# Supplementary material for: Catalyst-controlled stereodivergent synthesis of polysubstituted alkenes
Source: Nat Commun. 2025 Oct 14;16:9107. doi: 10.1038/s41467-025-64114-6 (PMC12521405; doi:10.1038/s41467-025-64114-6)
Supplement: Supplementary file 1 — Supplementary Information [file 41467_2025_64114_MOESM1_ESM.pdf]

Supplementary Information

Catalyst-controlled stereodivergent synthesis of polysubstituted alkenes

Chengmi Huang,[1,2†] Dong Wu,[1†] Yu-Qing Zheng,[3†] Lujin Wang,[1] Yuqiang Li,[4] Yangyang Li,[1,2]* Wen-Bo Liu,[3]* Guoyin Yin[1,2]*

[1] State Key Laboratory of Metabolism and Regulation in Complex Organisms, TaiKang Center for Life and Medical Sciences, The Institute for Advanced Studies, Wuhan University, Wuhan, Hubei, China.

[2] Shenzhen Research Institute, Wuhan University, Shenzhen, China.

[3] Hubei Research Center of Fundamental Science-Chemistry, Engineering Research Center of Organosilicon Compounds & Materials (Ministry of Education), Hubei Key Lab on Organic and Polymeric Opto-Electronic, Materials and College of Chemistry and Molecular Sciences, Wuhan University, Wuhan, Hubei, China.

[4] Shanghai Artificial Intelligence Laboratory, Shanghai, China.

[†] These authors contributed equally: Chengmi Huang, Dong Wu, Yu-Qing Zheng

*Emails: yinguoyin@whu.edu.cn; wenboliu@whu.edu.cn; yangyangl@whu.edu.cn

Content

1. General Method 1

1.1 General information 1

1.2 Ligand effect 2

2. General procedure 3

3. Analytical data of compounds 4

4. Synthetic applications 23

4.1 Synthesis of *E* and *Z*-allyl alcohols 23

4.2 Synthesis of a key pharmacophore in pharmaceutical compounds 24

4.3 Synthesis of (*E*, *E*)-α-homofarnesene 26

5. Mass Balance Analysis 27

6. Mechanistic studies 27

6.1 Isomerization experiments 27

6.2 Quenching experiment. 28

6.3 Proton-stoichiometric experiment. 29

6.4 BnBr-stoichiometric experiment. 30

6.5 Radical trapping experiments 31

6.6 Radical clock experiments 32

6.7 Cyclic voltammetry experiments 34

7. Computational analysis 39

8. X-ray characterization data 45

9. NMR spectra 49

10. References 169

1. General Method

1.1 General information

General information: All reactions were run under a dry argon atmosphere fitted on a glass tube or vial. All glassware was oven-dried at 120 ºC for 2 h and cooled down under vacuum. Thin layer chromatography (TLC) employed glass 0.25 mm silica gel plates. Flash chromatography columns were packed with 200-300 mesh silica gel in petroleum (bp. 60-90 ºC). GC-MS spectra were recorded on a Varian GC-MS 3900-2100 T. GC analysis was performed on a Shimadzu GC-2010pro instrument with a flame ionization detector and a SHRXI-5MS column (10 m, 0.1 mm inner diameter, 0.25 µm film thickness) The high-resolution mass spectra were measured on Thermo Fisher Scientific Exactive Plus (ESI or APCI). Optical rotation was determined using a Perkin Elmer 343 polarimeter. All new compounds were characterized by ^{1}H NMR, ^{13}C NMR, ^{11}B NMR, ^{29}Si NMR, ^{19}F NMR ^{31}P NMR and HRMS. The known compounds were characterized by ^{1}H NMR, ^{13}C NMR. ^{1}H, ^{13}C ^{11}B ^{19}F and ^{29}Si NMR ^{31}P NMR data were recorded with JNM-ECZ 400 and Bruker 600 MHz with tetramethylsilane as an internal standard. Data for ^{1}H, ^{13}C ^{11}B ^{29}Si ^{19}F and ^{31}P are reported as follows: chemical shift (δ ppm), multiplicity (s = singlet, d = doublet, t = triplet, q = quartet, dd = doublet of doublet, dt = doublet of triplet, dq = doublet of quartet, m = multiplet), integration, and coupling constant (Hz). All chemical shifts (δ) were reported in ppm and coupling constants (J) in Hz. All chemical shifts were reported relative to tetramethylsilane (0 ppm for ^{1}H), Chloroform-d (77.16 ppm for ^{13}C), respectively. The X-ray single crystal diffraction data were collected on a Bruker D8 VENTURE CMOS photon 100 diffractometer with helios mx multilayer monochrmator Cu Kα radiation (λ = 1.54178 Å) at the Core Facility of Wuhan University

Materials: $NiCl_2 \cdot$DME (CAS: 29046-78-4) and LiOMe were purchased from Energy Chemical and stored in the glove box. Anhydrous 1,4-dioxane was purchased from Adamas-beta®. Unless otherwise noted, alkynes, alkyl bromides and bis(pinacolato)diboron were obtained from commercial suppliers (Energy Chemical, Adamas-beta®, *J&K* and Innochem so on) and used without further purification.

1.2 Ligand effect

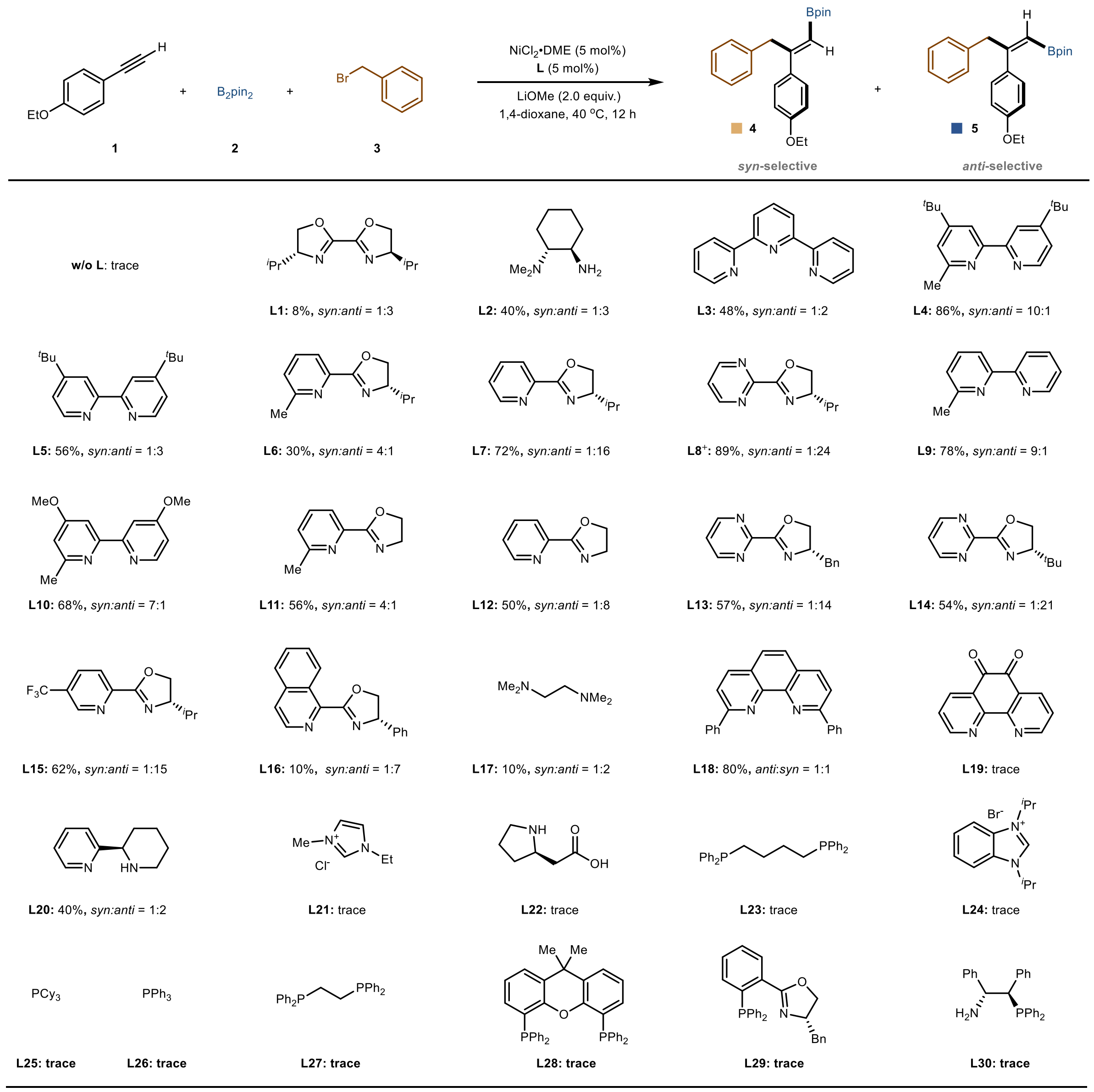

Figure S1 Reaction Optimization. $NiCl_2$·DME (5 mol%), **L** (5 mol%), **1** (0.4 mmol, 1.0 equiv.), **2** (0.8 mmol, 2.0 equiv.), **3** (0.6 mmol, 1.5 equiv.), and LiOMe (0.8 mmol, 2.0 equiv.) in 1,4-dioxane (0.2 M), stirred at 40 °C for 12 h. GC yields, *syn*:*anti* selectivities were determined by GC analysis of the crude reaction mixture. $^{+}$**3** (0.8 mmol, 2.0 equiv.).

2. General procedure

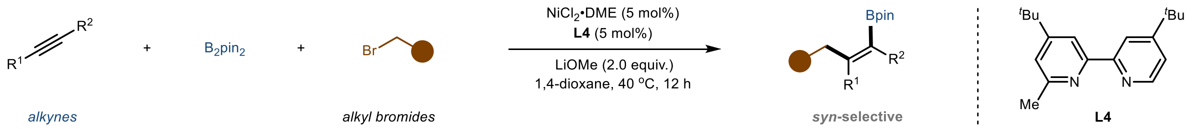

General procedure A: Under N_2 atmosphere, an oven-dried 10 mL reaction tube which equipped with a magnetic stir bar and sealed with a rubber stopper sequentially was added $NiCl_2 \cdot DME$ (0.02 mmol, 5 mol%), **L4** (0.02 mmol, 5 mol%), LiOMe (0.8 mmol, 2.0 equiv.) and B_2pin_2 (0.8 mmol, 2 equiv.). Then anhydrous 1,4-dioxane (1 mL), alkynes (0.4 mmol, 1.0 equiv.), alkylbromide (0.6 mmol, 1.5 equiv.), anhydrous 1,4-dioxane (1 mL) were added and the mixture was stirred. After 12 h of stirring at 40 °C, the resulting mixture was quenched with water (3 mL) and further diluted with ethyl acetate (3 mL). Then the mixture was extracted with ethyl acetate and the combined organic layers were dried over anhydrous Na_2SO_4, filtered, and concentrated under vacuum. The crude material was separated on a silica gel column affording the desired *syn*-product.

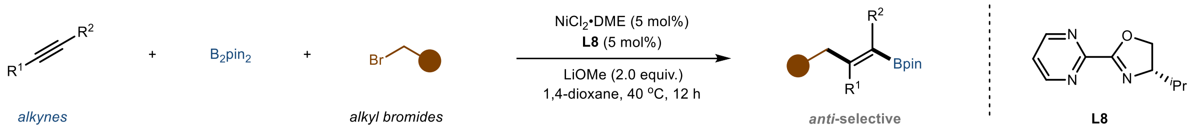

General procedure B: Under N_2 atmosphere, an oven-dried 10 mL reaction tube which equipped with a magnetic stir bar and sealed with a rubber stopper sequentially was added $NiCl_2 \cdot DME$ (0.02 mmol, 5 mol%), **L8** (0.02 mmol, 5 mol%), LiOMe (0.8 mmol, 2.0 equiv.) and B_2pin_2 (0.8 mmol, 2.0 equiv.). Then anhydrous 1,4-dioxane (1 mL), alkynes (0.4 mmol, 1.0 equiv.), alkylbromide (0.8 mmol, 2 equiv.), anhydrous 1,4-dioxane (1 mL) were added and the mixture was stirred. After 12 h of stirring at 40 °C, the resulting mixture was quenched with water (3 mL) and further diluted with ethyl acetate (3 mL). Then the mixture was extracted with ethyl acetate and the combined organic layers were dried over anhydrous Na_2SO_4, filtered, and concentrated under vacuum. The crude material was separated on a silica gel column affording the desired *anti*-product.

3. Analytical data of compounds

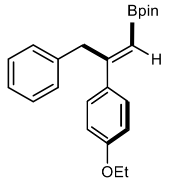

(*E*)-2-(2-(4-ethoxyphenyl)-3-phenylprop-1-en-1-yl)-4,4,5,5-tetramethyl-1,3,2-dioxaborolane (4): This compound was synthesized according to **General procedure A,** petroleum ether/ethyl acetate = 40:1, colorless oil, isolated yield: 115.0 mg, 79%; **^{1}H NMR** (600 MHz, Chloroform-*d*) δ 7.36 (d, *J* = 8.8 Hz, 2H), 7.22 - 7.16 (m, 4H), 7.11 - 7.07 (m, 1H), 6.74 (d, *J* = 8.8 Hz, 2H), 5.82 (s, 1H), 4.26 (s, 2H), 3.97 (q, *J* = 7.0 Hz, 2H), 1.36 (t, *J* = 7.0 Hz, 3H), 1.27 (s, 12H); **^{13}C NMR** (101 MHz, Chloroform-*d*) δ 159.8, 159.0, 140.7, 135.1, 128.6, 128.2, 128.0, 125.7, 114.1, 83.1, 63.4, 39.2, 24.9, 14.9 ppm; **^{11}B NMR** (193 MHz, Chloroform-*d*) δ 30.50 ppm; **HRMS (ESI)** calculated $[M+H]^+$ for $C_{23}H_{30}O_3B^+$ = 365.2283, found: 365.2288.

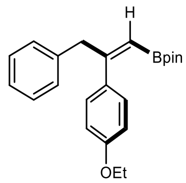

(*Z*)-2-(2-(4-ethoxyphenyl)-3-phenylprop-1-en-1-yl)-4,4,5,5-tetramethyl-1,3,2-dioxaborolane (5): This compound was synthesized according to **General procedure B**, petroleum ether/ethyl acetate = 40:1, colorless oil, isolated yield: 124.4 mg, 85%; **^{1}H NMR** (600 MHz, Chloroform-*d*) δ 7.24 - 7.19 (m, 4H), 7.16 - 7.12 (m, 3H), 6.77 (d, *J* = 8.6 Hz, 2H), 5.27 (s, 1H), 4.00 (q, *J* = 7.0 Hz, 2H), 3.76 (s, 2H), 1.39 (t, *J* = 7.0 Hz, 3H), 1.13 (s, 12H); **^{13}C NMR** (151 MHz, Chloroform-*d*) δ 160.1, 158.6, 139.2, 135.0, 129.5, 129.3, 128.3, 126.2, 113.6, 83.1, 63.5, 46.9, 24.7, 14.9 ppm; **^{11}B NMR** (193 MHz, Chloroform-*d*) δ 30.50 ppm; **HRMS (ESI)** calculated $[M+H]^+$ for $C_{23}H_{30}O_3B^+$ = 365.2283, found: 365.2286.

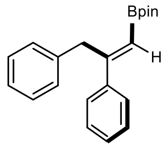

(*E*)-2-(2,3-diphenylprop-1-en-1-yl)-4,4,5,5-tetramethyl-1,3,2-dioxaborolane (6): This compound was synthesized according to **General procedure A**, petroleum ether/ethyl acetate = 50:1, colorless oil, isolated yield: 103.7 mg, 81%; **^{1}H NMR** (600 MHz, Chloroform-*d*) δ 7.40 (m, 1H), 7.39 (m, 1H), 7.24 - 7.16 (m, 7H), 7.11 - 7.08 (m, 1H), 5.85 (s, 1H), 4.27 (s, 2H), 1.28 (s, 12H); **^{13}C NMR** (151 MHz, Chloroform-*d*) δ 160.5, 143.1, 140.4, 128.7, 128.3, 128.2, 127.9, 126.8, 125.8, 83.2, 39.4, 25.0 ppm; **^{11}B NMR** (193 MHz, Chloroform-*d*) δ 30.42 ppm. **HRMS (ESI)** calculated $[M+H]^+$ for $C_{21}H_{26}O_{22}B^+$ = 321.2020, found: 321.2026.

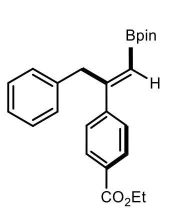

Ethyl (*E*)-4-(3-phenyl-1-(4,4,5,5-tetramethyl-1,3,2-dioxaborolan-2-yl)prop-1-en-2-yl)benzoate (7): This compound was synthesized according to **General procedure A**, petroleum ether/ethyl acetate = 30:1, colorless oil, isolated yield: 101.9 mg, 65%; **^{1}H NMR** (600 MHz, Chloroform-*d*) δ 7.93 - 7.88 (m, 2H), 7.47 - 7.40 (m, 2H), 7.18 - 7.15 (m, 4H), 7.11 - 7.08 (m, 1H), 5.89 (s, 1H), 4.33 (q, *J* = 7.1 Hz, 2H), 4.28 (s, 2H), 1.35 (t, *J* = 7.1 Hz, 3H), 1.30 (s, 12H); **^{13}C NMR** (151 MHz, Chloroform-*d*) δ 166.5, 159.6, 147.5, 139.8, 129.7, 129.5, 128.7, 128.3, 126.8, 126.0, 83.4, 61.0, 39.4, 24.9, 14.4 ppm; **^{11}B NMR** (193 MHz, Chloroform-*d*) δ 30.71 ppm; **HRMS (ESI)** calculated $[M+H]^+$ for $C_{24}H_{30}O_4B^+$ = 393.2232, found: 393.2219.

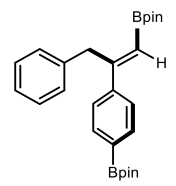

(*E*)-4,4,5,5-tetramethyl-2-(4-(3-phenyl-1-(4,4,5,5-tetramethyl-1,3,2-dioxaborolan-2-yl)prop-1-en-2-yl)phenyl)-1,3,2-dioxaborolane (8): This compound was synthesized according to **General procedure A**, petroleum ether/ethyl acetate = 20:1, white solid, isolated yield: 119.5 mg, 67%; **^{1}H NMR** (600 MHz, Chloroform-*d***)** δ 7.69 - 7.66 (m, 2H), 7.40 (d, *J* = 8.3 Hz, 2H), 7.26 (m, 1H), 7.17 - 7.14 (m, 4H), 5.86 (s, 1H), 4.27 (s, 2H), 1.31 (s, 12H), 1.29 (s, 12H); **^{13}C NMR** (151 MHz, Chloroform-*d*) δ 160.6, 145.8, 140.3, 134.7, 128.8, 128.3, 126.2, 125.8, 83.9, 83.3, 39.3, 25.00, 24.97 ppm; **^{11}B NMR** (193 MHz, Chloroform-*d*) δ 30.85 ppm; **HRMS (ESI)** calculated [M+H]$^+$ for $C_{27}H_{37}O_4B_2^+$ = 447.2873, found: 447.2861.

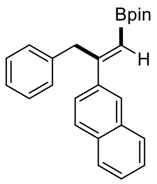

(*E*)-4,4,5,5-tetramethyl-2-(2-(naphthalen-1-yl)-3-phenylprop-1-en-1-yl)-1,3,2-dioxaborolane (9): This compound was synthesized according to **General procedure A**, petroleum ether/ethyl acetate = 40:1, white solid, isolated yield: 103.6 mg, 70%; **^{1}H NMR** (600 MHz, Chloroform-*d***)** δ 7.90 (d, *J* = 8.0 Hz, 1H), 7.79 (d, *J* = 6.4 Hz, 1H), 7.68 (d, *J* = 8.2 Hz, 1H), 7.43 - 7.37 (m, 2H), 7.27 (d, *J* = 7.8 Hz, 1H), 7.12 - 7.04 (m, 3H), 7.03 - 6.98 (m, 2H), 6.92 - 6.91 (d, *J* = 6.9 Hz, 1H), 5.51 (s, 1H), 4.24 (s, 2H), 1.38 (s, 12H); **^{13}C NMR** (151 MHz, Chloroform-*d*) δ 162.1, 142.9, 139.7, 133.7, 130.8, 129.2, 128.2, 128.0, 127.2, 126.1, 125.9, 125.8, 125.6, 125.0, 124.9, 83.4, 43.1, 25.1 ppm; **^{11}B NMR** (193 MHz, Chloroform-*d*) δ 30.31 ppm; **HRMS (ESI)** calculated [M+H]$^+$ for $C_{25}H_{28}O_2B^+$ = 371.2177, found: 371.2183.

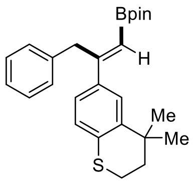

(*E*)-2-(2-(4,4-dimethylthiochroman-6-yl)-3-phenylprop-1-en-1-yl)-4,4,5,5-tetramethyl-1,3,2-dioxaborolane (10): This compound was synthesized according to **General procedure A**, petroleum ether/ethyl acetate = 20:1, white solid, isolated yield: 131.0 mg, 78%; **^{1}H NMR** (600 MHz, Chloroform-*d***)** δ 7.42 (d, *J* = 2.1 Hz, 1H), 7.22 - 7.17 (m, 4H), 7.12 - 7.10 (m, 2H), 6.93 (d, *J* = 8.3 Hz, 1H), 5.86 (s, 1H), 4.24 (s, 2H), 2.99 - 2.93 (m, 2H), 1.93 - 1.87 (m, 2H), 1.28 (s, 12H), 1.22 (s, 6H); **^{13}C NMR (**151 MHz, Chloroform-*d***)** δ 160.0, 141.5, 140.6, 138.4, 131.9, 128.7, 128.3, 126.3, 125.8, 125.2, 124.4, 83.2, 39.2, 37.8, 33.1, 30.3, 24.9, 23.2 ppm; **^{11}B NMR** (193 MHz, Chloroform-*d*) δ 30.56 ppm; **HRMS (ESI)** calculated [M+H]$^+$ for $C_{26}H_{34}O_2SB^+$ = 421.2367, found: 421.2363.

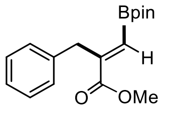

methyl (*E*)-2-benzyl-3-(4,4,5,5-tetramethyl-1,3,2-dioxaborolan-2-yl)acrylate (11): This compound was synthesized according to **General procedure A**, petroleum ether/ethyl acetate = 30:1, colorless oil, isolated yield: 61.6 mg, 51%; **^{1}H NMR** (600 MHz, Chloroform-*d***)** δ 7.29 – 7.27 (m, 2H), 7.25 – 7.23 (m, *J* = 7.6 Hz, 2H), 7.17 – 7.15 (m, 1H), 6.62 (s, 1H), 3.99 (s, 2H), 3.66 (s, 3H), 1.30 (s, 12H).; **^{13}C NMR (**151 MHz, Chloroform-*d***)** δ 167.8, 150.0, 139.8, 129.0, 128.3, 126.1, 83.9, 52.1, 36.5, 24.9 ppm; **^{11}B NMR** (193 MHz, Chloroform-*d*) δ 30.26 ppm. **HRMS (ESI)** calculated [M+H]$^+$ for $C_{17}H_{24}O_4B^+$ = 303.1762, found: 303.1757.

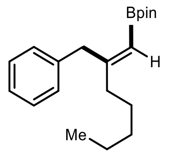

(*Z*)-2-(2-benzylhept-1-en-1-yl)-4,4,5,5-tetramethyl-1,3,2-dioxaborolanee (12): This compound was synthesized according to **General procedure A**, petroleum ether/ethyl acetate = 50:1, colorless oil, isolated yield: 87.9 mg, 70%; **^{1}H NMR (600 MHz,** Chloroform-*d***)** δ 7.19 - 7.14 (m, 4H), 7.11 - 7.08 (m, 1H), 5.18 (s, 1H), 3.69 (s, 2H), 1.92 - 1.87 (m, 2H), 1.34 - 1.31 (m, 2H), 1.20 (s, 12H), 1.16 - 1.09 (m, 4H), 0.76 (t, *J* = 7.1 Hz, 3H); **^{13}C NMR** (151 MHz, Chloroform-*d*) δ 165.3, 140.7, 129.0, 128.3, 125.9, 82.9, 41.1, 38.3, 31.6, 27.5, 25.0, 22.7, 14.1 ppm; **^{11}B NMR** (193 MHz, Chloroform-*d*) δ 30.57 ppm; **HRMS (ESI)** calculated $[M+H]^+$ for $C_{20}H_{32}O_2B^+$ = 315.2490, found: 315.2498.

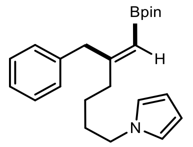

(*Z*)-1-(5-benzyl-6-(4,4,5,5-tetramethyl-1,3,2-dioxaborolan-2-yl)hex-5-en-1-yl)-1H-pyrrole (13): This compound was synthesized according to **General procedure A**, petroleum ether/ethyl acetate = 50:1, colorless oil, isolated yield: 75.9 mg, 52%; **^{1}H NMR** (600 MHz, Chloroform-*d*) δ 7.28 - 7.23 (m, 2H), 7.22 - 7.16 (m, 3H), 6.57 (t, *J* = 2.1 Hz, 2H), 6.10 (t, *J* = 2.1 Hz, 2H), 5.23 (s, 1H), 3.76 (t, *J* = 7.1 Hz, 2H), 3.74 (s, 2H), 2.03 - 1.95 (m, 2H), 1.66 (m, 2H), 1.43 -1.37 (m, 2H), 1.28 (s, 12H); **^{13}C NMR** (151 MHz, Chloroform-*d*) δ 164.0, 140.4, 129.0, 128.4, 126.1, 120.6, 107.9, 83.0, 49.5, 41.0, 37.6, 31.1, 25.0, 24.7 ppm; **^{11}B NMR** (193 MHz, Chloroform-*d*) δ 30.38 ppm; **HRMS (ESI)** calculated $[M+H]^+$ for $C_{23}H_{33}O_2NB^+$ = 366.2599, found: 366.2590.

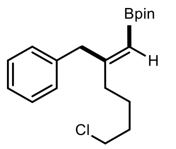

(*Z*)-2-(2-benzyl-6-chlorohex-1-en-1-yl)-4,4,5,5-tetramethyl-1,3,2-dioxaborolane (14): This compound was synthesized according to **General procedure A**, petroleum ether/ethyl acetate = 50:1, colorless oil, isolated yield: 64.1 mg, 48%; **^{1}H NMR** (600 MHz, Chloroform-*d***)** δ 7.28 - 7.25 (m, 2H), 7.24 - 7.22 (m, *J* = 6.6 Hz, 2H), 7.20 -7 .16 (m, 1H), 5.26 (s, 1H), 3.77 (s, 2H), 3.45 (t, *J* = 6.7 Hz, 2H), 2.06 -1.98 (m, 2H), 1.68 (p, *J* = 6.8 Hz, 2H), 1.57 -1.52 (m, 2H), 1.29 (s, 12H).; **^{13}C NMR** (151 MHz, Chloroform-*d*) δ 164.0, 140.4, 129.0, 128.4, 126.1, 83.0, 45.1, 41.0, 37.3, 32.1, 25.0, 24.9 ppm; **^{11}B NMR** (193 MHz, Chloroform-*d*) δ 29.84 ppm; **HRMS (ESI)** calculated $[M+H]^+$ for $C_{19}H_{29}O_2ClB^+$ = 335.1944, found: 335.1949.

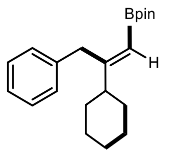

(*E*)-2-(2-cyclohexyl-3-phenylprop-1-en-1-yl)-4,4,5,5-tetramethyl-1,3,2-dioxaborolane (15): This compound was synthesized according to **General procedure A**, petroleum ether/ethyl acetate = 50:1, colorless oil, isolated yield: 97.8 mg, 75%; **^{1}H NMR** (600 MHz, Chloroform-*d***)** δ 7.28 -7.20 (m, 4H), 7.18 -7.15 (m, 1H), 5.29 (s, 1H), 3.80 (s, 2H), 1.83 - 1.80 (m, 1H), 1.68 - 1.56 (m, 5H), 1.26 (s, 12H), 1.16 -1.07 (m, 5H).; **^{13}C NMR** (151 MHz, Chloroform-*d*) δ 170.0, 140.8, 129.0, 128.2, 125.8, 82.9, 45.4, 40.7, 32.9, 26.9, 26.5, 25.0 ppm; **^{11}B NMR** (193 MHz, Chloroform-*d*) δ 31.36 ppm; **HRMS (ESI)** calculated $[M+H]^+$ for $C_{21}H_{32}O_2B^+$ = 327.2490, found: 327.2499.

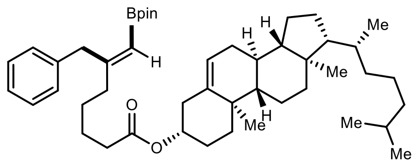

(3*S*,8*S*,9*S*,10*R*,13*R*,14*S*,17*R*)-10,13-dimethyl-17-((*R*)-6-methylheptan-2-yl)-2,3,4,7,8,9,10,11,12,13,14,15,16,17-tetradecahydro-1H-cyclopenta[a]phenanthren-3-yl (*Z*)-6-benzyl-7-(4,4,5,5-tetramethyl-1,3,2-dioxaborolan-2-yl)hept-6-enoate (16): This compound was synthesized according to **General procedure A**, petroleum ether/ethyl acetate = 30:1, colorless oil, isolated yield: 170.8 mg, 60%; **^{1}H NMR** (600 MHz, Chloroform-*d*) δ 7.27 - 7.21 (m, 4H), 7.19 - 7.15 (m, 1H), 5.37 (d, *J* = 5.6 Hz, 1H), 5.24 (s, 1H), 4.63 - 4.54 (m, 1H), 3.76 (s, 2H), 2.31 -2.26 (m, 2H), 2.20 (t, *J* = 7.4 Hz, 2H), 2.03 -1.93 (m, 4H), 1.84 - 1.83 (m, 3H), 1.61 -1 .37 (m, 12H), 1.37 -1.30 (m, 3H), 1.28 (s, 12H), 1.20 -1.04 (m, 7H), 1.03 -0.93 (m, 6H), 0.91 (d, *J* = 6.5 Hz, 3H), 0.86 (dd, *J* = 6.6, 2.7 Hz, 6H), 0.68 (s, 3H); **^{13}C NMR** (151 MHz, Chloroform-*d*) δ 173.1, 164.2, 140.4, 139.7, 128.9, 128.2, 125.9, 122.6, 82.8, 73.7, 56.7, 56.1, 50.0, 42.3, 40.9, 39.8, 39.5, 38.2, 37.7, 37.0, 36.6, 36.2, 35.8, 34.5, 31.92, 31.87, 28.3, 28.0, 27.8, 27.1, 24.9, 24.7, 24.3, 23.8, 22.9, 22.6, 21.0, 19.4, 18.7, 11.9 ppm; **^{11}B NMR** (193 MHz, Chloroform-*d*) δ 30.79 ppm; **HRMS (APCI)** calculated $[M+H]^+$ for $C_{47}H_{74}O_4B^+$ = 713.5675, found: 713.5687.

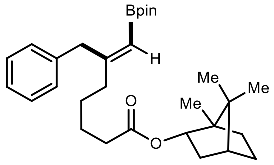

(1*S*,2*R*,4*S*)-1,7,7-trimethylbicyclo[2.2.1]heptan-2-yl (*Z*)-6-benzyl-7-(4,4,5,5-tetramethyl-1,3,2-dioxaborolan-2-yl)hept-6-enoate (17): This compound was synthesized according to **General procedure A**, petroleum ether/ethyl acetate = 30:1, colorless oil, isolated yield: 111.4 mg, 58%; **^{1}H NMR** (600 MHz, Chloroform-*d*) δ 7.27 -7.21 (m, 4H), 7.20 -7.15 (m, 1H), 5.25 (s, 1H), 4.87 -4.85 (m, 1H), 3.76 (s, 2H), 2.36 -2.29 (m, 1H), 2.24 (t, *J* = 7.5 Hz, 2H), 2.01 (t, *J* = 7.0 Hz, 2H), 1.93 -1.88 (m, 1H), 1.76 -1.70 (m, 1H), 1.67 -1.65 (m, 1H), 1.57 -1.53 (m, 2H), 1.47 -1.43 (m, 2H), 1.28 (s, 12H), 1.26 -1.25 (m, 2H), 0.92 (dd, *J* = 13.8, 3.5 Hz, 1H), 0.90 (s, 3H), 0.86 (s, 3H), 0.81 (s, 3H); **^{13}C NMR** (151 MHz, Chloroform-*d*) δ 174.0, 164.2, 140.4, 128.9, 128.3, 125.9, 82.8, 79.6, 48.7, 47.5, 44.9, 40.9, 37.8, 36.8, 34.6, 28.1, 27.13, 27.11, 24.9, 24.7, 19.7, 18.9, 13.5 ppm; **^{11}B NMR** (193 MHz, Chloroform-*d*) δ 30.22 ppm; **HRMS (APCI)** calculated $[M+NH_4]^+$ for $C_{30}H_{49}NO_4B^+$ =498.3749, found: 498.3764.

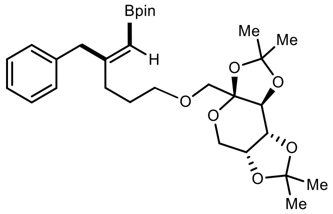

2-((*Z*)-2-benzyl-5-(((3a*S*,5a*R*,8a*R*,8b*S*)-2,2,7,7-tetramethyltetrahydro-3aH-bis([1,3]dioxolo)[4,5-b:4',5'-d]pyran-3a-yl)methoxy)pent-1-en-1-yl)-4,4,5,5-tetramethyl-1,3,2-dioxaborolane (18): This compound was synthesized according to **General procedure A**, petroleum ether/ethyl acetate = 30:1, colorless oil, isolated yield: 119.6 mg, 55%; **^{1}H NMR** (600 MHz, Chloroform-*d*) δ 7.27 -7.21 (m, 4H), 7.19 - 7.16 (m, 1H), 5.25 (s, 1H), 4.58 (dd, *J* = 7.9, 2.6 Hz, 1H), 4.34 (d, *J* = 2.6 Hz, 1H), 4.22 (dd, *J* = 7.9, 1.8 Hz, 1H), 3.89 (dd, *J* = 13.0, 1.9 Hz, 1H), 3.77 (d, *J* = 3.0 Hz, 2H), 3.70 (d, *J* = 12.9 Hz, 1H), 3.51 (d, *J* = 10.4 Hz, 1H), 3.48 -3.42 (m, 2H), 3.40 -3.37 (m, 1H), 2.05 (t, *J* = 7.8 Hz, 2H), 1.72 - 1.67 (m, 2H), 1.51 (s, 3H), 1.46 (s, 3H), 1.34 (s, 6H), 1.28 (s, 12H); **^{13}C NMR** (151 MHz, Chloroform-*d*) δ 164.3, 140.4, 129.0, 128.4, 126.0, 109.0, 108.6, 102.8, 83.0, 72.1, 71.7, 71.2, 70.4, 70.0, 61.1, 41.2, 34.8, 27.8, 26.7, 26.0, 25.5, 25.0, 24.2 ppm; **^{11}B NMR** (193 MHz, Chloroform-*d*) δ 30.28 ppm; **HRMS (ESI)** calculated $[M+H]^+$for $C_{30}H_{46}O_8B^+$ = 545.3271, found: 545.3271.

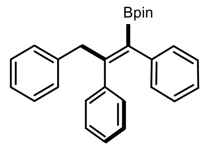

(*Z*)-4,4,5,5-tetramethyl-2-(1,2,3-triphenylprop-1-en-1-yl)-1,3,2-dioxaborolane (19): This compound was synthesized according to **General procedure A**, petroleum ether/ethyl acetate = 40:1, white solid, isolated yield: 130.0 mg, 82%; **^{1}H NMR (**600 MHz, Chloroform-*d***)** δ 7.23 - 7.18 (m, 4H), 7.13 - 7.10 (m, 1H), 7.06 -7.03 (m, 2H), 7.01 - 6.94 (m, 6H), 6.80 - 6.78 (m, 2H), 4.06 (s, 2H), 1.31 (s, 12H); **^{13}C NMR** (151 MHz, Chloroform-*d*) δ 151.8, 141.9, 141.6, 139.7, 129.7, 129.3, 129.1, 128.2, 127.54, 127.45, 126.3, 125.9, 125.4, 83.8, 44.5, 24.8 ppm; **^{11}B NMR** (193 MHz, Chloroform-*d*) δ 31.22 ppm; **HRMS (ESI)** calculated $[M+NH_4]^+$for $C_{27}H_{33}O_2NB^+$ = 414.2599, found: 414.2602.

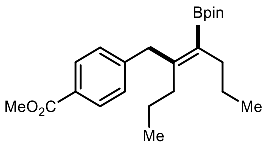

Methyl(*E*)-4-(2-propyl-3-(4,4,5,5-tetramethyl-1,3,2-dioxaborolan-2-yl)hex-2-en-1-yl)benzoate (20): This compound was synthesized according to **General procedure A**, petroleum ether/ethyl acetate = 20:1, colorless oil, isolated yield: 120.4 mg, 78%; **^{1}H NMR** (600 MHz, Chloroform-d**)** δ 7.93 (d, *J* = 8.3 Hz, 2H), 7.30 (d, *J* = 8.1 Hz, 2H), 3.89 (s, 3H), 3.72 (s, 2H), 2.22 - 2.13 (m, 2H), 1.96 - 1.87 (m, 2H), 1.42 - 1.35 (m, 2H), 1.34 - 1.29 (m, 2H), 1.24 (s, 12H), 0.91 (t, *J* = 7.3 Hz, 3H), 0.84 (t, *J* = 7.3 Hz, 3H); **^{13}C NMR** (151 MHz, Chloroform-*d*) δ 167.4, 151.0, 147.3, 129.6, 129.0, 127.8, 83.1, 52.0, 41.8, 34.0, 33.1, 24.9, 23.8, 22.0, 14.6, 14.4 ppm; **^{11}B NMR** (193 MHz, Chloroform-*d*) δ 31.91 ppm; **HRMS (ESI)** calculated $[M+H]^+$ for $C_{23}H_{36}O_4B^+$ = 387.2701, found: 387.2709.

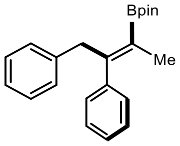

(*Z*)-2-(3,4-diphenylbut-2-en-2-yl)-4,4,5,5-tetramethyl-1,3,2-dioxaborolane (21): This compound was synthesized according to **General procedure A**, petroleum ether/ethyl acetate = 40:1, colorless oil, isolated yield: 74.8 mg, 56%; **^{1}H NMR** (600 MHz, Chloroform-d**)** δ 7.19 (m, 2H), 7.14 (d, *J* = 6.0 Hz, 3H), 7.09 (d, *J* = 5.9 Hz, 1H), 7.05 (d, *J* = 6.7 Hz, 2H), 6.86 -6.82 (m, 2H), 3.99 (s, 2H), 1.60 (s, 3H), 1.33 (s, 12H); **^{13}C NMR** (151 MHz, Chloroform-*d*) δ 153.7, 142.9, 140.3, 129.2, 128.3, 128.0, 127.9, 126.3, 125.7, 83.4, 44.1, 25.0, 18.5 ppm; **^{11}B NMR** (193 MHz, Chloroform-*d*) δ 31.56 ppm. Analytical data were identical to those previously reported.[1]

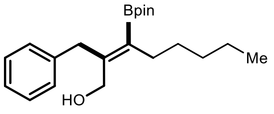

(*Z)-2-benzyl-3-(4,4,5,5-tetramethyl-1,3,2-dioxaborolan-2-yl)oct-2-en-1-ol* (22): This compound was synthesized according to **General procedure A**, petroleum ether/ethyl acetate = 10:1, colorless oil, isolated yield: 89.4 mg, 65%; **^{1}H NMR** (600 MHz, Chloroform-d**)** δ 7.30 (d, *J* = 6.6 Hz, 4H), 7.22 – 7.19 (m,), 4.10 (s, 2H), 3.82 (s, 2H), 2.27 (t, *J* = 7.7 Hz, 2H), 1.42 – 1.32 (m, 6H), 1.31 (s, 12H), 0.91 (t, *J* = 6.9 Hz, 3H); **^{13}C NMR** (151 MHz, Chloroform-*d*) δ 149.1, 140.8, 129.1, 128.5, 126.1, 83.4, 60.7, 39.9, 32.0, 30.6, 30.5, 24.9, 22.7, 14.2 ppm; **^{11}B NMR** (193 MHz, Chloroform-*d*) δ 31.12 ppm. **HRMS (ESI)** calculated $[M-OH]^+$ for $C_{21}H_{32}O_2B^+$ = 327.2490, found: 327.2488.

The regioselectivity and stereoselectivity of the product **22** are determined based on the ^{1}H NMR spectrum and the NOE of the product after deborylation product **22’**.

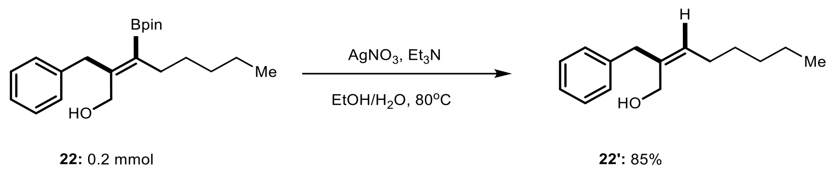

(Z)-2-benzyloct-2-en-1-ol (22'): This compound was synthesized according to literature procedure[2]**,** colorless oil, petroleum ether/ethyl acetate = 10:1, isolated yield: 0.2 mmol, 36.9 mg, 85%; **^{1}H NMR** (600 MHz, Chloroform-d**)** δ 7.23 – 7.19 (m, 2H), 7.15 – 7.12 (m, 3H), 5.35 (t, *J* = 7.5 Hz, 1H), 4.00 (s, 2H), 3.38 (s, 2H), 2.03 (q, *J* = 7.4 Hz, 2H), 1.33 – 1.28 (m, 2H), 1.27 – 1.21 (m, 4H), 0.82 (t, J = 7.0 Hz, 3H).; **^{13}C NMR** (151 MHz, Chloroform-*d*) δ 140.2, 137.7, 131.1, 129.0, 128.6, 126.3, 59.9, 41.9, 31.6, 29.8, 27.8, 22.7, 14.2 ppm.

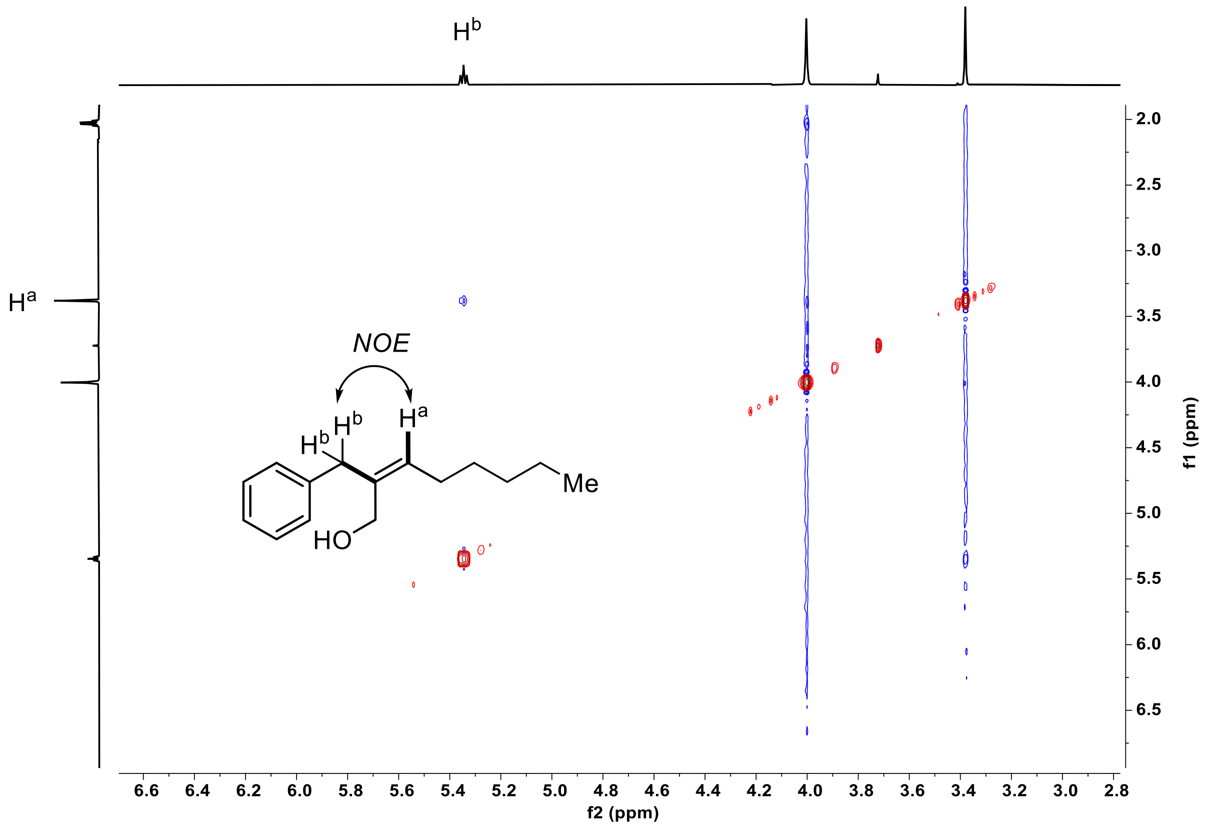

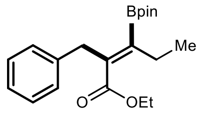

ethyl (*Z*)-2-benzyl-3-(4,4,5,5-tetramethyl-1,3,2-dioxaborolan-2-yl)pent-2-enoate (23): This compound was synthesized according to **General procedure A**, petroleum ether/ethyl acetate = 20:1, colorless oil, isolated yield: 75.7 mg, 55%; **^{1}H NMR** (600 MHz, Chloroform-d**)** δ 7.20 – 7.11 (m, 4H), 7.11 – 7.05 (m, 1H), 3.96 (q, *J* = 7.1 Hz, 2H), 3.80 (s, 2H), 2.26 (q, *J* = 7.5 Hz, 2H), 1.21 (s, 12H), 1.02 (t, *J* = 7.1 Hz, 3H), 0.97 (t, *J* = 7.5 Hz, 3H); **^{13}C NMR** (151 MHz, Chloroform-*d*) δ 169.2, 142.3, 139.5, 129.0, 128.3, 126.1, 83.8, 60.1, 39.3, 26.3, 24.9, 14.6, 14.2 ppm; **^{11}B NMR** (193 MHz, Chloroform-*d*) δ 31.25ppm. **HRMS (ESI)** calculated $[M+H]^+$ for $C_{20}H_{30}O_4B^+$ = 345.2232, found: 345.2225.

The regioselectivity and stereoselectivity of the product **22** are determined based on the ^{1}**H NMR** spectrum and the **NOE** of the product after deborylation product **22'**.

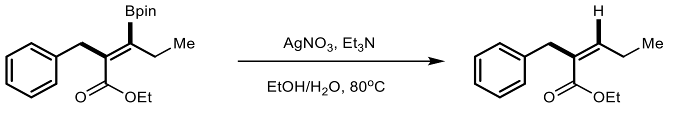

23: 0.2 mmol

23': 80%

ethyl (Z)-2-benzylpent-2-enoate (23'): This compound was synthesized according to literature procedure[2], colorless oil, petroleum ether/ethyl acetate = 20:1, isolated yield: 0.2 mmol 34.9 mg, 80%; ^{1}**H NMR** (600 MHz, Chloroform-d) δ 7.22 – 7.17 (m, 2H), 7.14 – 7.08 (m, 3H), 5.84 (t, *J* = 7.4 Hz, 1H), 4.05 (q, *J* = 7.1 Hz, 2H), 3.50 (s, 2H), 2.41 (p, *J* = 7.5 Hz, 2H), 1.13 (t, *J* = 7.1 Hz, 3H), 0.96 (t, J = 7.5 Hz, 3H); ^{13}**C NMR** (151 MHz, Chloroform-*d*) δ 167.7, 145.4, 139.8, 130.9, 128.9, 128.4, 126.3, 60.2, 40.6, 23.1, 14.3, 14.0 ppm.

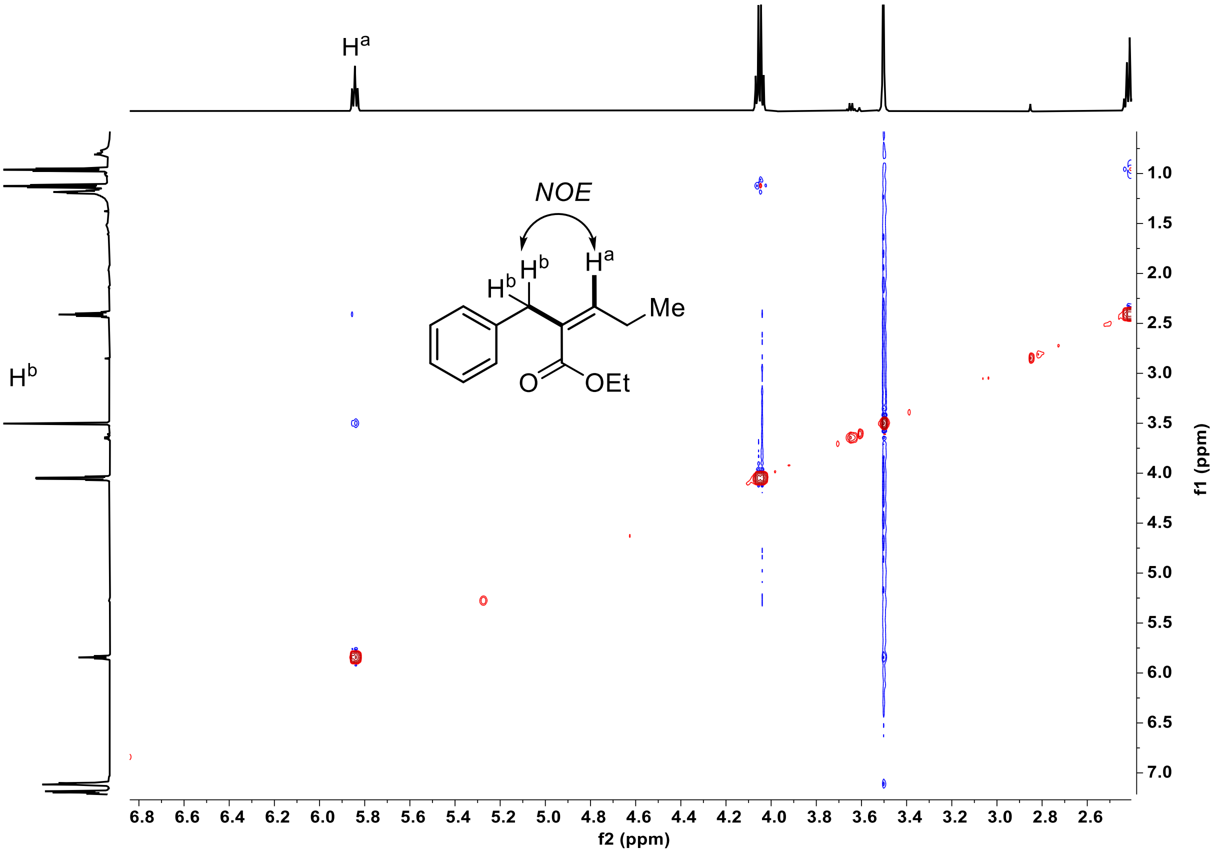

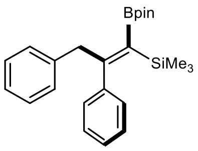

(*Z*)-(2,3-diphenyl-1-(4,4,5,5-tetramethyl-1,3,2-dioxaborolan-2-yl)prop-1-en-1-yl)trimethylsilane (24): This compound was synthesized according to **General procedure A**, petroleum ether/ethyl acetate = 40:1, white solid, isolated yield: 123.9 mg, 79%; ^{1}**H NMR** (600 MHz, Chloroform-d) δ 7.35 (m, 2H), 7.32 - 7.28 (m, 4H), 7.27 - 7.24 (m, 2H), 7.02 (m, 2H), 4.01 (s, 2H), 1.55 (s, 12H), -0.00 (s, 9H); ^{13}**C NMR** (151 MHz, Chloroform-*d*) δ 163.4, 144.8, 138.9, 129.4, 128.4, 128.1, 127.4, 126.7, 125.9, 83.7, 49.4, 25.5 0.86 ppm; ^{11}**B NMR** (193 MHz, Chloroform-*d*) δ 31.79 ppm;

^{29}Si NMR (119 MHz, Chloroform-*d*) δ -8.58 ppm; **HRMS (ESI)** calculated $[M+H]^+$ for $C_{24}H_{34}O_2BSi^+$ = 393.2416, found: 393.2433.

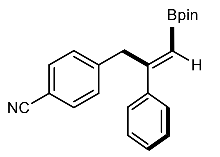

(*E*)-4-(2-phenyl-3-(4,4,5,5-tetramethyl-1,3,2-dioxaborolan-2-yl)allyl)benzonitrile (25): This compound was synthesized according to **General procedure A**, petroleum ether/ethyl acetate = 20:1, white solid, isolated yield: 85.6 mg, 62%; **^{1}H NMR** (600 MHz, Chloroform-d**)** δ 7.48 -7.46 (m, 1H), 7.47 - 7.46 (m, 1H), 7.36 (m, 1H), 7.35 (m, 1H), 7.30 - 7.27 (m, 2H), 7.27 - 7.23 (m, 3H), 5.91 (s, 1H), 4.32 (s, 2H), 1.28 (s, 12H); **^{13}C NMR** (151 MHz, Chloroform-*d*) δ 158.9, 146.3, 142.3, 132.1, 129.4, 128.5, 128.4, 126.6, 119.3, 109.7, 83.4, 39.5, 24.9 ppm; **^{11}B NMR** (193 MHz, Chloroform-*d*) δ 30.06 ppm; **HRMS (ESI)** calculated $[M+H]^+$ for $C_{22}H_{25}O_2NB^+$ = 346.1973, found: 346.1965.

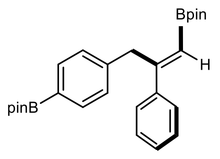

(*E*)-4,4,5,5-tetramethyl-2-(4-(2-phenyl-3-(4,4,5,5-tetramethyl-1,3,2-dioxaborolan-2-yl)allyl)phenyl)-1,3,2-dioxaborolane (26): This compound was synthesized according to **General procedure A**, petroleum ether/ethyl acetate = 20:1, white solid, isolated yield: 96.3 mg, 54%; **^{1}H NMR** (600 MHz, Chloroform-*d*) δ 7.63 (d, *J* = 8.0 Hz, 2H), 7.40 -7.36 (m, 2H), 7.23 -7.18 (m, 5H), 5.87 (s, 1H), 4.29 (s, 2H), 1.30 (s, 12H), 1.28 (s, 12H); **^{13}C NMR** (151 MHz, Chloroform-*d*) δ 160.2, 143.8, 142.9, 134.8, 128.2, 128.1, 128.0, 126.8, 83.7, 83.3, 39.7, 24.99, 24.95 ppm; **^{11}B NMR (193 MHz, Chloroform-*d*)** δ 30.44 ppm; **HRMS (ESI)** calculated $[M+H]^+$ for $C_{27}H_{37}O_4B_2^+$ = 447.2873, found: 447.2872.

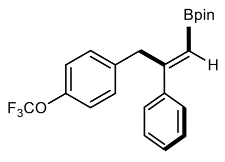

(*E*)-4,4,5,5-tetramethyl-2-(2-phenyl-3-(4-(trifluoromethoxy)phenyl)prop-1-en-1-yl)-1,3,2-dioxaborolane (27): This compound was synthesized according to **General procedure A**, petroleum ether/ethyl acetate = 40:1, colorless oil, isolated yield: 101.8 mg, 63%; **^{1}H NMR** (600 MHz, Chloroform-d**)** δ 7.41 - 7.36 (m, 2H), 7.27 - 7.22 (m, 3H), 7.22 - 7.18 (m, 2H), 7.03 -7.01 (m, 2H), 5.86 (s, 1H), 4.26 (s, 2H), 1.28 (s, 12H); **^{13}C NMR** (151 MHz, Chloroform-*d*) δ 160.0, 147.5, 142.8, 139.2, 129.9, 128.4, 128.2, 126.7, 120.8, 120.6 (q, *J* = 256.7 Hz), 83.3, 38.7, 24.9 ppm; **^{11}B NMR** (193 MHz, Chloroform-*d*) δ 30.11 ppm; **^{19}F NMR** (565 MHz, Chloroform-*d*) δ -57.87 ppm; **HRMS (ESI)** calculated $[M+H]^+$ for $C_{22}H_{25}O_3BF_3^+$ = 405.1843, found: 405.1833.

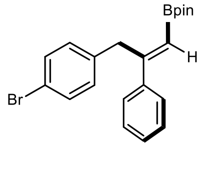

(*E*)-2-(3-(4-bromophenyl)-2-phenylprop-1-en-1-yl)-4,4,5,5-tetramethyl-1,3,2-dioxaborolane (28): This compound was synthesized according to **General procedure A**, petroleum ether/ethyl acetate = 40:1, colorless oil, isolated yield: 71.6 mg, 45%; **^{1}H NMR** (600 MHz, Chloroform-*d*) δ 7.37 - 7.36 (m, 2H), 7.29 (d, *J* = 8.4 Hz, 2H), 7.26 - 7.22 (m, 3H), 7.06 (d, *J* = 8.1 Hz, 2H), 5.85 (s, 1H), 4.21 (s, 2H), 1.28 (s, 12H); **^{13}C NMR** (151 MHz, Chloroform-*d*) δ

159.9, 142.7, 139.4, 131.3, 130.4, 128.3, 128.1, 126.7, 119.6, 83.3, 38.8, 25.0 ppm; **^{11}B NMR** (193 MHz, Chloroform-*d*) δ 30.46 ppm; **HRMS (ESI)** calculated $[M+H]^+$ for $C_{21}H_{25}O_2BrB^+$ = 399.1126, found: 399.1138.

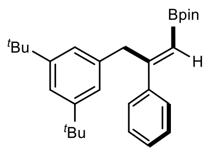

(*E*)-2-(3-(3,5-di-tert-butylphenyl)-2-phenylprop-1-en-1-yl)-4,4,5,5-tetramethyl-1,3,2-dioxaborolane (29): This compound was synthesized according to **General procedure A**, petroleum ether/ethyl acetate = 40:1, colorless oil, isolated yield: 108.9 mg, 63%; **^{1}H NMR** (600 MHz, Chloroform-d) δ 7.38 (d, *J* = 7.1 Hz, 2H), 7.26 -7.18 (m, 3H), 7.13 (s, 1H), 7.01 (d, *J* = 1.9 Hz, 2H), 5.74 (s, 1H), 4.23 (s, 2H), 1.30 (s, 12H), 1.22 (s, 18H); **^{13}C NMR** (151 MHz, Chloroform-*d*) δ 161.8, 150.2, 143.8, 139.2, 128.1, 127.7, 127.1, 123.3, 119.5, 83.2, 40.1, 34.8, 31.6, 25.0 ppm; **^{11}B NMR** (193 MHz, Chloroform-*d*) δ 30.54 ppm; **HRMS (ESI)** calculated $[M+H]^+$ for $C_{29}H_{42}O_2B^+$ = 433.3272, found: 433.3266.

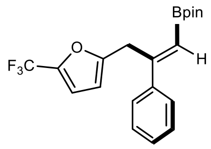

(*E*)-4,4,5,5-tetramethyl-2-(2-phenyl-3-(5-(trifluoromethyl)furan-2-yl)prop-1-en-1-yl)-1,3,2-dioxaborolane (30): This compound was synthesized according to **General procedure A**, petroleum ether/ethyl acetate = 40:1, colorless oil, isolated yield: 117.9 mg, 78%; **^{1}H NMR** (600 MHz, Chloroform-d) δ 7.49 - 7.41 (m, 2H), 7.32 - 7.25 (m, 3H), 6.58 (dd, *J* = 3.3, 1.4 Hz, 1H), 5.94 (s, 1H), 5.93 (d, *J* = 3.3 Hz, 1H), 4.30 (s, 2H), 1.27 (s, 12H); **^{13}C NMR** (151 MHz, Chloroform-*d*) δ 158.1, 156.1, 142.2, 140.1 (q, *J* = 42.3 Hz), 128.5, 128.4, 126.4, 119.4 (q, *J* = 267.3 Hz), 112.5 (q, *J* = 2.9 Hz), 106.9, 83.4, 32.3, 24.9 ppm; **^{11}B NMR** (193 MHz, Chloroform-*d*) δ 30.59 ppm; **^{19}F NMR** (565 MHz, Chloroform-*d*) δ -63.85 ppm; **HRMS (ESI)** calculated $[M+H]^+$ for $C_{20}H_{23}O_3F_3B^+$ = 379.1687, found: 379.1689.

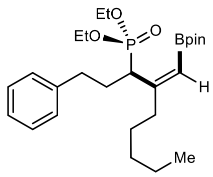

Diethyl (*Z*)-(1-phenyl-4-((4,4,5,5-tetramethyl-1,3,2-dioxaborolan-2-yl)methylene)nonan-3-yl)phosphonate (31): This compound was synthesized according to **General procedure A**, petroleum ether/ethyl acetate = 3:1, colorless oil, isolated yield: 87.9 mg, 46%; **^{1}H NMR** (600 MHz, Chloroform-d) δ 7.27 -7.24 (m, 2H), 7.19 -7.16 (m, 3H), 5.48 (s, 1H), 4.13 - 4.00 (m, 5H), 2.58 - 2.51 (m, 2H), 2.41 -2.29 (m, 1H), 2.23 -2.11 (m, 2H), 2.09 -2.00 (m, 1H), 1.59 - 1.48 (m, 2H), 1.34 - 1.32 (m, 4H), 1.29 - 1.24 (m, 18H), 0.91 -0.87 (m, 3H); **^{13}C NMR** (151 MHz, Chloroform-*d*) *δ* 160.3 (d, *J* = 7.5 Hz), 141.8, 128.4, 128.3, 125.9, 82.9, 61.9 (dd, *J* = 6.5, 6.4 Hz), 43.5 (d, *J* = 131.8 Hz), 34.0 (d, *J* = 15.1 Hz), 33.3, 31.7, 28.6 (d, *J* = 4.5 Hz), 27.6, 24.9, 24.7, 22.7, 16.4 (dd, *J* = 3.0 Hz, 1.5 Hz), 14.0 ppm; **^{11}B NMR** (193 MHz, Chloroform-*d*) δ 30.20 ppm; **^{31}P NMR** (243 MHz, Chloroform-*d*) δ 29.49 ppm; **HRMS (APCI)** calculated $[M+H]^+$ for $C_{26}H_{45}O_5BP^+$ = 479.3092, found: 479.3095.

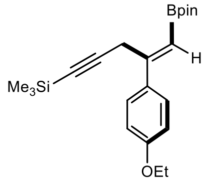

(*E*)-(4-(4-ethoxyphenyl)-5-(4,4,5,5-tetramethyl-1,3,2-dioxaborolan-2-yl)pent-4-en-1-yn-1-yl)trimethylsilane: (32): This compound was synthesized according to **General procedure A**, petroleum ether/ethyl acetate = 30:1, colorless oil, isolated yield: 98.0 mg, 65%; **^{1}H NMR** (600 MHz, Chloroform-*d*) δ 7.48 (d, *J* = 8.9 Hz, 2H), 6.79 (d, *J* = 8.8 Hz, 2H), 5.68 (s, 1H), 4.00 (q, *J* = 7.0 Hz, 2H), 3.79 (s, 2H), 1.36 (t, *J* = 7.0 Hz, 3H), 1.25 (s, 12H), 0.00 (s, 9H); **^{13}C NMR** (151 MHz, Chloroform-*d*) δ 159.2, 155.9, 134.0, 128.0, 114.0, 106.0, 85.3, 83.2, 63.6, 25.0, 24.5, 15.0, 0.13 ppm; **^{11}B NMR** (193 MHz, Chloroform-*d*) δ 30.36 ppm; **^{29}Si NMR** (119 MHz, Chloroform-*d*) δ -19.02 ppm; **HRMS (APCI)** calculated $[M+H-H_2O]^+$ for $C_{22}H_{33}O_3BSi^+$ = 385.2365, found: 385.2372.

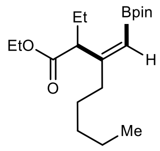

Ethyl (*Z*)-2-ethyl-3-((4,4,5,5-tetramethyl-1,3,2-dioxaborolan-2-yl)methylene)octanoate (33): This compound was synthesized according to **General procedure A**, petroleum ether/ethyl acetate = 30:1, colorless oil, isolated yield: 74.4 mg, 55%; **^{1}H NMR** (600 MHz, Chloroform-*d*) δ 5.35 (s, 1H), 4.16 (dd, *J* = 8.3, 6.7 Hz, 1H), 4.11 (q, *J* = 7.1 Hz, 2H), 2.06 - 1.99 (m, 2H), 1.96 - 1.87 (m, 1H), 1.58 - 1.50 (m, 1H), 1.43 - 1.45 (m, 2H), 1.27-1.30 (m, 16H), 1.23 (t, *J* = 7.1 Hz, 3H), 0.85 - 0.87 (m, 6H); **^{13}C NMR** (151 MHz, Chloroform-*d*) δ 174.2, 162.0, 82.9, 60.3, 52.2, 33.6, 31.7, 27.6, 24.9, 23.4, 22.7, 14.3, 14.0, 12.0 ppm; **^{11}B NMR** (193 MHz, Chloroform-*d*) 29.95 ppm; **HRMS (ESI)** calculated $[M+H]^+$ for $C_{19}H_{36}BO_4^+$ = 339.2710, found: 339.2701.

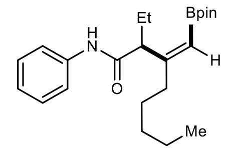

(*Z*)-2-ethyl-N-phenyl-3-((4,4,5,5-tetramethyl-1,3,2-dioxaborolan-2-yl)methylene)octanamide (34): This compound was synthesized according to **General procedure A**, petroleum ether/ethyl acetate = 5:1, colorless oil, isolated yield: 81.6 mg, 53%; **^{1}H NMR** (600 MHz, Chloroform-*d*) δ 8.62 (s, 1H), 7.57 -7.43 (m, 2H), 7.29 (m, 2H), 7.07 - 7.04 (m, 1H), 5.35 (s, 1H), 3.81 (t, *J* = 7.4 Hz, 1H), 2.21 - 2.04 (m, 2H), 2.04 - 1.93 (m, 1H), 1.66 - 1.61 (m, 1H), 1.46 - 1.42 (m, 1H), 1.38 (d, *J* = 17.4 Hz, 12H), 1.30 -1.23 (m, 5H), 0.87 (t, *J* = 7.4 Hz, 3H), 0.84 (t, *J* = 6.9 Hz, 3H); **^{13}C NMR** (151 MHz, Chloroform-*d*) δ 171.2, 164.5, 139.0, 129.1, 123.7, 119.2, 83.9, 55.8, 32.9, 31.7, 27.3, 25.5, 24.7, 22.7, 21.8, 14.1, 12.3 ppm; **^{11}B NMR** (193 MHz, Chloroform-*d*) δ 30.63 ppm; **HRMS (APCI)** calculated $[M+H]^+$ for $C_{23}H_{37}NO_3B^+$ = 386.2861, found: 386.2863.

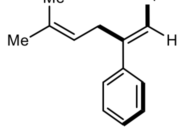

(*E*)-4,4,5,5-tetramethyl-2-(5-methyl-2-phenylhexa-1,4-dien-1-yl)-1,3,2-dioxaborolane (35): This compound was synthesized according to **General procedure A**, petroleum ether/ethyl acetate = 40:1, colorless oil, isolated yield: 85.8 mg, 72%; **^{1}H NMR** (600 MHz, Chloroform-d**)** δ 7.44 (d, *J* = 7.0 Hz, 2H), 7.30 - 7.29 (m, *J* = 7.4 Hz, 2H), 7.27 - 7.25 (m, 1H), 5.64 (s, 1H), 5.03 (t, *J* = 7.0 Hz, 1H), 3.63 (d, *J* = 6.9 Hz, 2H), 1.69 (s, 3H), 1.61 (s, 3H), 1.31 (s, 12H); **^{13}C NMR** (151 MHz, Chloroform-*d*) 162.0, 143.4, 132.0, 128.2, 127.9, 126.6, 123.1, 83.1, 32.9, 25.9, 25.0, 18.2 ppm; **^{11}B NMR** (193 MHz, Chloroform-*d*) δ 30.32 ppm; **HRMS (ESI)** calculated $[M+H]^+$ for $C_{19}H_{28}O_2B^+$ = 299.2177, found: 299.2169.

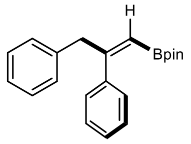

(*Z*)-2-(2,3-diphenylprop-1-en-1-yl)-4,4,5,5-tetramethyl-1,3,2-dioxaborolane (36): This compound was synthesized according to **General procedure B**, petroleum ether/ethyl acetate = 40:1, colorless oil, isolated yield: 116.5 mg, 91%; **^{1}H NMR** (600 MHz, Chloroform-*d*) δ 7.28 - 7.20 (m, 7H), 7.18 - 7.10 (m, 3H), 5.32 (t, *J* = 1.4 Hz, 1H), 3.77 (s, 2H), 1.10 (s, 12H); **^{13}C NMR** (151 MHz, Chloroform-*d*) δ 160.6, 142.8, 138.9, 129.6, 128.4, 128.1, 127.7, 127.5, 126.3, 83.1, 46.9, 24.7 ppm; **^{11}B NMR** (193 MHz, Chloroform-d) δ 30.07 ppm; **HRMS (ESI)** calculated $[M+H]^+$ for $C_{21}H_{26}O_2B^+$ = 321.2020, found: 321.2019.

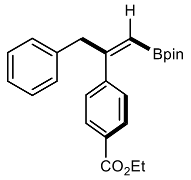

Ethyl (*Z*)-4-(3-phenyl-1-(4,4,5,5-tetramethyl-1,3,2-dioxaborolan-2-yl)prop-1-en-2-yl)benzoate (37): This compound was synthesized according to **General procedure B**, petroleum ether/ethyl acetate = 20:1, colorless oil, isolated yield: 92.5 mg, 59%; **^{1}H NMR** (600 MHz, Chloroform-*d*) δ 7.94 - 7.92 (m, 2H), 7.30 -7.28 (m, 2H), 7.25 - 7.21 (m, 2H), 7.18 - 7.15 (m, 1H), 7.14 - 7.10 (m, 2H), 5.43 (t, *J* = 1.4 Hz, 1H), 4.37 (q, *J* = 7.1 Hz, 2H), 3.77 (s, 2H), 1.39 (t, *J* = 7.1 Hz, 3H), 1.10 (s, 12H); **^{13}C NMR** (151 MHz, Chloroform-*d*) δ 166.8, 159.9, 147.4, 138.4, 129.6, 129.4, 129.1, 128.5, 128.2, 126.5, 83.4, 61.0, 47.1, 24.7, 14.5 ppm; **^{11}B NMR** (193 MHz, Chloroform-*d*) δ 30.51 ppm; **HRMS (ESI)** calculated $[M+H]^+$ for $C_{24}H_{30}O_4B^+$ = 393.2232, found: 393.2227.

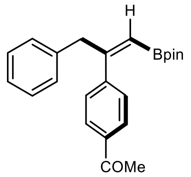

(*Z*)-1-(4-(3-phenyl-1-(4,4,5,5-tetramethyl-1,3,2-dioxaborolan-2-yl)prop-1-en-2-yl)phenyl)ethan-1-one. (38): This compound was synthesized according to **General procedure B**, petroleum ether/ethyl acetate = 20:1, colorless oil, isolated yield: 89.8 mg, 62%; **^{1}H NMR** (600 MHz, Chloroform-*d*) δ 7.80 - 7.75 (m, 2H), 7.25 (d, *J* = 8.3 Hz, 2H), 7.19 - 7.15 (m, 2H), 7.10 (t, *J* = 7.4 Hz, 1H), 7.07 - 7.02 (m, 2H), 5.38 (t, *J* = 1.4 Hz, 1H), 3.70 (s, 2H), 2.52 (s, 3H), 1.03 (s, 12H); **^{13}C NMR** (151 MHz, Chloroform-*d*) δ 198.1, 159.7, 147.7, 138.3, 136.1, 129.5, 128.5, 128.4, 127.9, 126.5, 83.4, 47.0, 26.8, 24.7 ppm; **^{11}B NMR** (193 MHz, Chloroform-*d*) δ 30.31 ppm; **HRMS (ESI)** calculated $[M+H]^+$ for $C_{23}H_{28}O_3B^+$ = 363.2126, found: 363.2117.

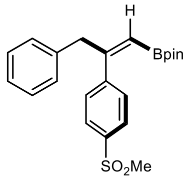

(*Z*)-4,4,5,5-tetramethyl-2-(2-(4-(methylsulfonyl)phenyl)-3-phenylprop-1-en-1-yl)-1,3,2-dioxaborolane (39): This compound was synthesized according to **General procedure B**, petroleum ether/ethyl acetate = 5:1, colorless oil, isolated yield: 89.2 mg, 56%; **^{1}H NMR** (600 MHz, Chloroform-*d*) δ 7.82 (d, *J* = 8.3 Hz, 2H), 7.39 (d, *J* = 8.3 Hz, 2H), 7.25 (m, 2H), 7.19 (m, 1H), 7.12 (d, *J* = 6.7 Hz, 2H), 5.50 (t, *J* = 1.4 Hz, 1H), 3.76 (s, 2H), 3.03 (s, 3H), 1.08 (s, 12H); **^{13}C NMR** (151 MHz, Chloroform-*d*) δ 159.1, 148.6, 139.2, 137.8, 129.5, 129.1, 128.6, 126.8, 126.6, 83.4, 47.1, 44.7, 24.7 ppm; **^{11}B NMR** (193 MHz, Chloroform-*d*) δ 31.91 ppm; **HRMS (APCI)** calculated $[M+H]^+$ for $C_{22}H_{28}O_4SB^+$ = 399.1796, found: 399.1804.

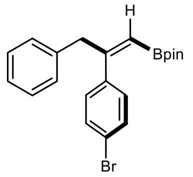

***(Z)*-2-(2-(4-bromophenyl)-3-phenylprop-1-en-1-yl)-4,4,5,5-tetramethyl-1,3,2-dioxaborolane (40)**: This compound was synthesized according to **General procedure B**, petroleum ether/ethyl acetate = 40:1, white solid, isolated yield: 101.8 mg, 64%; **^{1}H NMR** (600 MHz, Chloroform-*d*) δ 7.36 (d, *J* = 8.4 Hz, 2H), 7.23 (m, 2H), 7.17 (m, 1H), 7.12 - 7.10 (m, 4H), 5.38 (t, *J* = 1.4 Hz, 1H), 3.73 (s, 2H), 1.11 (s, 12H); **^{13}C NMR** (151 MHz, Chloroform-*d*) δ 159.6, 141.5, 138.5, 130.8, 129.9, 129.5, 128.5, 126.4, 121.5, 83.3, 47.1, 24.7 ppm; **^{11}B NMR** (193 MHz, Chloroform-*d*) δ 30.22 ppm; **HRMS (ESI)** calculated $[M+H]^+$ for $C_{21}H_{25}O_2BrB^+$ = 399.1126, found: 399.1135.

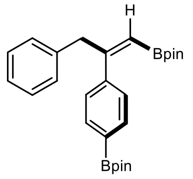

(*Z*)-4,4,5,5-tetramethyl-2-(4-(3-phenyl-1-(4,4,5,5-tetramethyl-1,3,2-dioxaborolan-2-yl)prop-1-en-2-yl)phenyl)-1,3,2-dioxaborolane (41): This compound was synthesized according to **General procedure B**, petroleum ether/ethyl acetate = 20:1, white solid, isolated yield: 132.0 mg, 74%; **^{1}H NMR** (600 MHz, Chloroform-*d*) δ 7.70 (d, *J* = 8.1 Hz, 2H), 7.28 - 7.26 (m, 2H), 7.24 - 7.21 (m, 2H), 7.16 -7.12 (m, 3H), 5.34 (t, *J* = 1.4 Hz, 1H), 3.77 (s, 2H), 1.34 (s, 12H), 1.11 (s, 12H); **^{13}C NMR** (151 MHz, Chloroform-*d*) δ 160.4, 145.5, 138.9, 134.3, 129.6, 128.4, 127.5, 126.3, 83.8, 83.3, 46.9, 25.0, 24.7 ppm; **^{11}B NMR** (193 MHz, Chloroform-*d*) δ 30.56 ppm; **HRMS (ESI)** calculated $[M+H]^+$ for $C_{27}H_{37}O_4B_2^+$ = 447.2873, found: 447.2871.

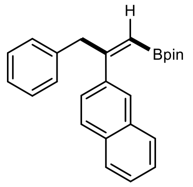

***(Z)*-4,4,5,5-tetramethyl-2-(2-(naphthalen-1-yl)-3-phenylprop-1-en-1-yl)-1,3,2-dioxaborolane (42)**: This compound was synthesized according to **General procedure B**, petroleum ether/ethyl acetate = 40:1, white solid, isolated yield: 90.3 mg, 61%; **^{1}H NMR** (600 MHz, Chloroform-*d*) δ 7.98 -7.91 (m, 1H), 7.84 - 7.78 (m, 1H), 7.73 (d, *J* = 8.2 Hz, 1H), 7.46 -7.40 (m, 2H), 7.38 (dd, *J* = 8.2, 7.0 Hz, 1H), 7.29 - 7.24 (m, 2H), 7.21 - 7.15 (m, 4H), 5.61 (t, *J* = 1.5 Hz, 1H), 3.86 -3.71 (m, 2H), 0.82 (s, 6H), 0.66 (s, 6H); **^{13}C NMR** (151 MHz, Chloroform-*d*) δ 160.1, 141.5, 138.6, 133.6, 131.8, 130.0, 128.4, 128.1, 127.0, 126.4, 125.8, 125.7, 125.4, 125.1, 125.0, 82.7, 48.3, 24.4, 24.2 ppm; **^{11}B NMR** (193 MHz, Chloroform-*d*) δ 30.08 ppm; **HRMS (ESI)** calculated $[M+H]^+$ for $C_{25}H_{28}O_2B^+$ = 371.2177, found: 371.2178.

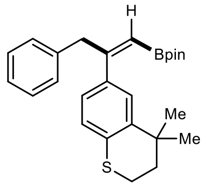

(*Z*)-2-(2-(4,4-dimethylthiochroman-6-yl)-3-phenylprop-1-en-1-yl)-4,4,5,5-tetramethyl-1,3,2-dioxaborolane (43): This compound was synthesized according to **General procedure B**, petroleum ether/ethyl acetate = 20:1, colorless oil, isolated yield: 117.6 mg, 70%; **^{1}H NMR** (600 MHz, Chloroform-*d*) 7.26 -7.19 (m, 3H), 7.17 - 7.11 (m, 3H), 6.97 - 6.92 (m, 2H), 5.30 (t, *J* = 1.4 Hz, 1H), 3.72 (s, 2H), 3.02 - 2.97 (m, 2H), 1.94 - 1.90 (m, 2H), 1.27 (s, 6H), 1.12 (s, 12H); **^{13}C NMR** (151 MHz, Chloroform-*d*) δ 160.6, 140.9, 139.0, 138.3, 130.6, 129.6, 128.3, 126.3, 126.2, 126.0, 125.7, 83.1, 47.5, 37.8, 33.1, 30.2, 24.7, 23.2 ppm; **^{11}B NMR** (193 MHz, Chloroform-*d*) δ 30.48 ppm; **HRMS (ESI)** calculated $[M+H]^+$ for $C_{26}H_{34}O_2BS^+$ = 421.2367, found: 421.2368.

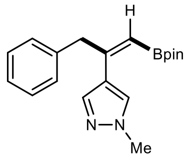

(*Z*)-1-methyl-4-(3-phenyl-1-(4,4,5,5-tetramethyl-1,3,2-dioxaborolan-2-yl)prop-1-en-2-yl)-1H-pyrazole (44): This compound was synthesized according to **General procedure B**, petroleum ether/ethyl acetate = 5:1, colorless oil, isolated yield: 99.8 mg, 77%; **^{1}H NMR** (600 MHz, Chloroform-*d*) δ 7.74 (s, 1H), 7.60 (s, 1H), 7.27 - 7.24 (m, 2H), 7.22 - 7.16 (m, 3H), 5.20 (t, *J* = 1.3 Hz, 1H), 3.82 (s, 3H), 3.74 (s, 2H), 1.27 (s, 12H); **^{13}C NMR** (151 MHz, Chloroform-*d*) δ 150.1, 139.6, 139.2, 130.0, 129.2, 128.5, 126.3, 122.8, 83.3, 47.1, 39.0, 25.0 ppm; **^{11}B NMR** (193 MHz, Chloroform-*d*) δ 30.91 ppm; **HRMS (ESI)** calculated $[M+H]^+$ for $C_{19}H_{26}O_2N_2B^+$ =325.2082, found: 325.2075.

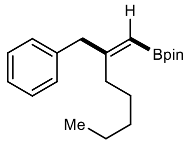

(*E*)-2-(2-benzylhept-1-en-1-yl)-4,4,5,5-tetramethyl-1,3,2-dioxaborolane (45): This compound was synthesized according to **General procedure B**, petroleum ether/ethyl acetate = 40:1, colorless oil, isolated yield: 119.3 mg, 95%; **^{1}H NMR** (600 MHz, Chloroform-*d*) δ 7.28 - 7.24 (m, 2H), 7.20 -7.13 (m, 3H), 5.03 (t, *J* = 1.4 Hz, 1H), 3.42 (s, 2H), 2.40 - 2.33 (m, 2H), 1.47 - 1.42 (m, 2H), 1.33 - 1.27 (m, 4H), 1.24 (s, 12H), 0.88 (t, *J* = 7.1 Hz, 3H); **^{13}C NMR** (151 MHz, Chloroform-*d*) δ 166.1, 139.6, 129.6, 128.4, 126.2, 82.7, 45.9, 34.4, 31.8, 29.2, 25.0, 22.6, 14.2 ppm; **^{11}B NMR** (193 MHz, Chloroform-*d*) δ 30.07 ppm; **HRMS (ESI)** calculated $[M+H]^+$ for $C_{20}H_{32}O_2B^+$ = 315.2490, found:315.2493.

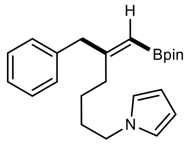

(*E*)-1-(5-benzyl-6-(4,4,5,5-tetramethyl-1,3,2-dioxaborolan-2-yl)hex-5-en-1-yl)-1H-pyrrole (46): This compound was synthesized according to **General procedure B**, petroleum ether/ethyl acetate = 40:1, colorless oil, isolated yield: 118.3 mg, 81%; **^{1}H NMR** (600 MHz, Chloroform-*d*) δ 7.27 - 7.25 (m, 2H), 7.19 - 7.17 (m, 1H), 7.13 - 7.12 (m, 2H), 6.64 (t, *J* = 2.1 Hz, 2H), 6.13 (t, *J* = 2.1 Hz, 2H), 5.09 (t, *J* = 1.3 Hz, 1H), 3.87 (t, *J* = 7.2 Hz, 2H), 3.38 (s, 2H), 2.44 - 2.40 (m, 2H), 1.78 - 1.73 (m, 2H), 1.46 - 1.41 (m, 2H), 1.23 (s, 12H); **^{13}C NMR** (151 MHz, Chloroform-*d*) δ 165.0, 139.2, 129.5, 128.4, 126.3, 120.6, 107.9, 82.8, 49.3, 45.7, 33.5, 31.1, 26.0, 25.0 ppm; **^{11}B NMR** (193 MHz, Chloroform-*d*) δ 30.98 ppm; **HRMS (ESI)** calculated $[M+H]^+$ for $C_{23}H_{33}O_2NB^+$ = 366.2599, found: 366.2594.

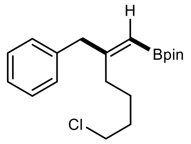

(*E*)-2-(2-benzyl-6-chlorohex-1-en-1-yl)-4,4,5,5-tetramethyl-1,3,2-dioxaborolane (47): This compound was synthesized according to **General procedure B**, petroleum ether/ethyl acetate = 40:1, colorless oil, isolated yield: 102.9 mg, 77%; **^{1}H NMR** (600 MHz, Chloroform-*d*) δ 7.28 - 7.26 (m, 2H), 7.20 - 7.18 (m, 1H), 7.15 (d, *J* = 6.7 Hz, 2H), 5.10 (t, *J* = 1.4 Hz, 1H), 3.55 (t, *J* = 6.8 Hz, 2H), 3.42 (s, 2H), 2.44 - 2.37 (m, 2H), 1.80 - 1.75 (m, 2H), 1.61 - 1.56 (m, 2H), 1.25 (s, 12H); **^{13}C NMR** (151 MHz, Chloroform-*d*) δ 164.9, 139.3, 129.5, 128.5, 126.3, 82.9, 45.8, 45.1, 33.3, 32.3, 26.4, 25.0 ppm; **^{11}B NMR** (193 MHz, Chloroform-*d*) δ 30.05 ppm; **HRMS (ESI)** calculated $[M+H]^+$ for $C_{19}H_{29}O_2ClB^+$ = 335.1944, found: 335.1951.

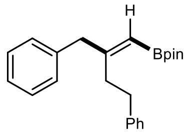

(*E*)-2-(2-benzyl-4-phenylbut-1-en-1-yl)-4,4,5,5-tetramethyl-1,3,2-dioxaborolane (48): This compound was synthesized according to **General procedure B**, petroleum ether/ethyl acetate = 40:1, colorless oil, isolated yield: 100.2 mg, 72%; **^{1}H NMR** (600 MHz, Chloroform-*d*) δ 7.28 - 7.25 (m, 4H), 7.21 - 7.14 (m, 6H), 5.11 (t, *J* = 1.4 Hz, 1H), 3.44 (s, 2H), 2.72 - 2.69 (m, 2H), 2.68 - 2.62 (m, 2H), 1.25 (s, 12H); **^{13}C NMR** (151 MHz, Chloroform-*d*) δ 165.2, 142.5, 139.2, 129.6, 128.6, 128.5, 128.3, 126.3, 125.8, 82.8, 46.6, 37.0, 36.6, 25.0 ppm; **^{11}B NMR** (193 MHz, Chloroform-*d*) δ 30.03 ppm; **HRMS (ESI)** calculated $[M+H]^+$ for $C_{23}H_{30}O_2B^+$ = 349.2333, found: 349.2334.

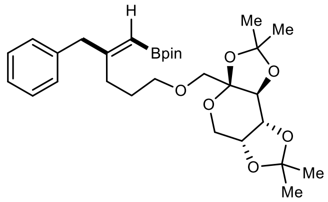

2-((*E*)-2-benzyl-5-(((3aS,5aR,8aR,8bS)-2,2,7,7-tetramethyltetrahydro-3aH-bis([1,3]dioxolo)[4,5-b:4',5'-d]pyran-3a-yl)methoxy)pent-1-en-1-yl)-4,4,5,5-tetramethyl-1,3,2-dioxaborolane (49): This compound was synthesized according to **General procedure B**, petroleum ether/ethyl acetate = 20:1, colorless oil, isolated yield: 178.7 mg, 82%; **^{1}H NMR** (600 MHz, Chloroform-*d*) δ 7.27 -7.24 (m, 2H), 7.20 - 7.16 (m, 1H), 7.16 - 7.12 (m, 2H), 5.06 (t, *J* = 1.4 Hz, 1H), 4.60 (dd, *J* = 7.9, 2.6 Hz, 1H), 4.41 (d, *J* = 2.6 Hz, 1H), 4.25 - 4.22 (m, 1H), 3.91 (dd, *J* = 13.0, 1.9 Hz, 1H), 3.72 (d, *J* = 12.2 Hz, 1H), 3.59 - 3.54 (m, 2H), 3.53 -3.51 (m, 1H), 3.49 - 3.45 (m, 1H), 3.42 (s, 2H), 2.45 - 2.38 (m, 2H), 1.78 - 1.73 (m, 2H), 1.54 (s, 3H), 1.47 (s, 3H), 1.41 (s, 3H), 1.34 (s, 3H), 1.24 (s, 12H); **^{13}C NMR** (101 MHz, $CDCl_3$) δ 165.1, 139.2, 129.4, 128.3, 126.1, 108.9, 108.5, 102.7, 82.7, 72.13, 72.05, 71.1, 70.3, 70.0, 61.0, 45.9, 31.0, 29.4, 26.6, 25.9, 25.4, 24.9, 24.1 ppm; **^{11}B NMR** (193 MHz, Chloroform-*d*) δ 30.48 ppm; **HRMS (ESI)** calculated $[M+H]^+$ for $C_{30}H_{46}O_8B^+$ = 545.3280, found: 545.3271.

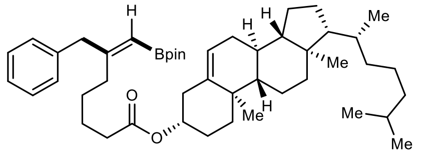

(3*S*,8*S*,9*S*,10*R*,13*R*,14*S*,17*R*)-10,13-dimethyl-17-((*R*)-6-methylheptan-2-yl)-2,3,4,7,8,9,10,11,12,13,14,15,16,17-tetradecahydro-1H-cyclopenta[a]phenanthren-3-yl (*E*)-6-benzyl-7-(4,4,5,5-tetramethyl-1,3,2-dioxaborolan-2-yl)hept-6-enoate (50): This compound was synthesized according to **General procedure B**, petroleum ether/ethyl acetate = 20:1, colorless oil, isolated yield: 213.6 mg, 75%; **^{1}H NMR** (600 MHz, Chloroform-*d*) δ 7.25 (m, 2H), 7.19 -7.15 (m, 1H), 7.15 -7.11 (m, 2H), 5.37 (d, *J* = 5.6 Hz, 1H), 5.07 (s, 1H), 4.66 - 4.58 (m, 1H), 3.41 (s, 2H), 2.44 - 2.36 (m, 2H), 2.30 (q, *J* = 7.7, 6.8 Hz, 4H), 2.04 - 1.93 (m, 2H), 1.86 - 1.84 (m, 3H), 1.67 - 1.39 (m, 12H), 1.38 - 1.30 (m, 3H), 1.24 (s, 12H), 1.19 -1.05 (m, 7H), 1.01 (s, 6H), 0.92 (d, *J* = 6.5 Hz, 3H), 0.86 (dd, *J* = 6.6, 2.7 Hz, 6H), 0.68 (s, 3H); **^{13}C NMR** (151 MHz, Chloroform-*d*) δ 173.2, 165.2, 139.7, 139.3, 129.4, 128.3, 126.1, 122.6, 82.7, 73.7, 56.7, 56.2, 50.1, 45.7, 42.3, 39.8, 39.6, 38.2, 37.0, 36.6, 36.2, 35.8, 34.5, 33.7, 31.9, 31.9, 28.7, 28.3, 28.0, 27.9, 24.9, 24.7, 24.3, 23.9, 22.9, 22.6, 21.1, 19.4, 18.8, 11.9 ppm; **^{11}B NMR** (193 MHz, Chloroform-*d*) δ 30.23 ppm; **HRMS (APCI)** calculated $[M+H]^+$ for $C_{47}H_{74}O_4B^+$ = 713.5675, found: 713.5683.

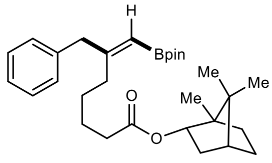

(1*S*,2*R*,4*S*)-1,7,7-trimethylbicyclo[2.2.1]heptan-2-yl (*E*)-6-benzyl-7-(4,4,5,5-tetramethyl-1,3,2-dioxaborolan-2-yl)hept-6-enoate (51): This compound was synthesized according to **General procedure B**, petroleum ether/ethyl acetate = 20:1, colorless oil, isolated yield: 136.3 mg, 71%; ^{1}**H NMR** (600 MHz, Chloroform-*d*) δ 7.28 -7.26 (m, 1H), 7.26 - 7.25 (m, 1H), 7.20 - 7.13 (m, 3H), 5.07 (s, 1H), 4.90 - 4.87 (m, 1H), 3.42 (s, 2H), 2.43 -2.38 (m, 2H), 2.36 - 2.29 (m, 3H), 1.97 -1.92 (m, 1H), 1.78 -1.71 (m, 1H), 1.68 - 1.62 (m, 3H), 1.52 - 1.48 (m, 2H), 1.31 - 1.27 (m, 2H), 1.25 (s, 12H), 0.95 (dd, *J* = 13.7, 3.5 Hz, 1H), 0.91 (s, 3H), 0.87 (s, 3H), 0.83 (s, 3H); ^{13}**C NMR** (151 MHz, Chloroform-*d*) δ 174.3, 165.3, 139.4, 129.5, 128.4, 126.3, 82.8, 79.7, 48.9, 47.9, 45.8, 45.0, 37.0, 34.7, 33.9, 28.9, 28.2, 27.3, 25.0, 24.9, 19.9, 13.7 ppm; ^{11}**B NMR (193 MHz, Chloroform-*d*)** δ 30.03 ppm. **HRMS (APCI)** calculated $[M+NH_4]^+$ for $C_{30}H_{49}NO_4B^+$ =498.3749, found: 498.3763.

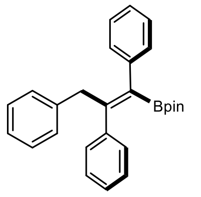

(*E*)-4,4,5,5-tetramethyl-2-(1,2,3-triphenylprop-1-en-1-yl)-1,3,2-dioxaborolane (52): This compound was synthesized according to **General procedure B**, petroleum ether/ethyl acetate = 40:1, white solid, isolated yield: 87.1 mg, 55%; ^{1}**H NMR** (600 MHz, Chloroform-*d*) δ 7.35 - 7.28 (m, 6H), 7.24 - 7.17 (m, 4H), 7.11 (m, 2H), 7.13 - 7.05 (m, 1H), 6.99 - 6.98 (m, 2H), 3.81 (s, 2H), 1.01 (s, 12H); ^{13}**C NMR** (151 MHz, Chloroform-d) δ 149.3, 143.5, 141.2, 140.0, 129.0, 128.8, 128.5, 128.4, 128.1, 127.9, 127.2, 126.4, 125.8, 83.6, 39.8, 24.5 ppm; ^{11}**B NMR** (193 MHz, Chloroform-*d*) δ 30.54 ppm; **HRMS (ESI)** calculated $[M+H]^+$ for $C_{27}H_{30}O_2B^+$ = 397.2333, found: 397.2333.

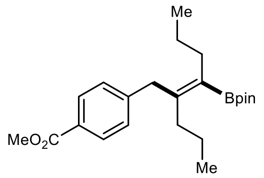

Methyl (*Z*)-4-(2-propyl-3-(4,4,5,5-tetramethyl-1,3,2-dioxaborolan-2-yl)hex-2-en-1-yl)benzoate (53): This compound was synthesized according to **General procedure B**, petroleum ether/ethyl acetate = 20:1, colorless oil, isolated yield: 74.1 mg, 48%; ^{1}**H NMR (6**00 MHz, Chloroform-*d*) δ 7.92 (d, *J* = 8.3 Hz, 2H), 7.26 -7.21 (m, 2H), 3.89 (s, 3H), 3.53 (s, 2H), 2.22 - 2.13 (m, 4H), 1.38 - 1.31 (m, 4H), 1.29 (s, 12H), 0.88 (t, *J* = 7.3 Hz, 3H), 0.82 (t, *J* = 7.3 Hz, 3H); ^{13}**C NMR** (151 MHz, Chloroform-*d*) δ 167.3, 149.7, 146.2, 129.7, 128.8, 127.9, 83.1, 52.1, 38.1, 37.5, 33.7, 24.9, 23. 7, 23.2, 14.4, 14.2 ppm; ^{11}**B NMR** (193 MHz, Chloroform-*d*) δ 31.44 ppm; **HRMS (ESI)** calculated $[M+H]^+$ for $C_{23}H_{36}O_4B^+$ = 387.2701, found: 387.2709.

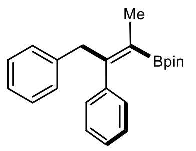

(*E*)-2-(3,4-diphenylbut-2-en-2-yl)-4,4,5,5-tetramethyl-1,3,2-dioxaborolane (54): This compound was synthesized according to **General procedure B**, petroleum ether/ethyl acetate = 40:1, colorless oil, isolated yield: 87.1 mg, 65%; ^{1}**H NMR** (600 MHz, Chloroform-*d*) δ 7.20 - 7.17 (m, 2H), 7.17 - 7.11 (m, 5H), 7.11 -7.03 (m, 3H), 3.85 (s, 2H), 1.95 (s, 3H), 1.07 (s, 12H); ^{13}**C NMR** (151 MHz, Chloroform-*d*) δ 149.3, 145.0, 139.2, 128.7, 128.6, 128.3, 127.8, 126.7, 125.8, 83.3, 39.6, 24.6, 17.4 ppm; ^{11}**B NMR** (193 MHz, Chloroform-*d*) δ 31.04 ppm; **HRMS (ESI)** calculated $[M+H]^+$ for $C_{22}H_{28}O_2B^+$ = 335.2177, found: 335.2189.

The regioselectivity of the product **54** are determined based on the ^{1}**H NMR** spectrum of the product after oxidation product **54'**.

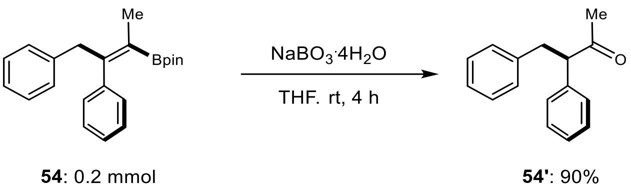

3,4-diphenylbutan-2-one (54'): This compound was synthesized according to literature procedure[3], colorless oil, petroleum ether/ethyl acetate = 20:1, isolated yield: 40.3 mg, 90%; **^{1}H NMR (400 MHz, Chloroform-*d*)** δ 7.34 – 7.15 (m, 8H), 7.10 – 7.04 (m, 2H), 3.95 (t, *J* = 7.3 Hz, 1H), 3.46 (dd, *J* = 13.9, 7.5 Hz, 1H), 2.93 (dd, *J* = 13.8, 7.3 Hz, 1H), 2.04 (s, 3H); **^{13}C NMR (101 MHz, Chloroform-*d*)** δ 207.7, 139.8, 138.5, 129.1, 129.0, 128.4, 128.3, 127.4, 126.2, 61.6, 38.4, 29.6 ppm. Analytical data were identical to those previously reported[4].

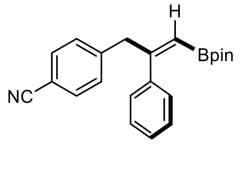

(*Z*)-4-(2-phenyl-3-(4,4,5,5-tetramethyl-1,3,2-dioxaborolan-2-yl)allyl)benzonitrile (55): This compound was synthesized according to **General procedure B**, petroleum ether/ethyl acetate = 20:1, white solid, isolated yield: 99.4 mg, 72%; **^{1}H NMR** (400 MHz, Chloroform-*d*) δ 7.52 (d, *J* = 8.1 Hz, 2H), 7.28 -7.20 (m, 7H), 5.35 (t, *J* = 1.4 Hz, 1H), 3.83 (s, 2H), 1.11 (s, 12H); **^{13}C NMR** (101 MHz, Chloroform-*d*) δ 158.8, 144.6, 141.9, 132.3, 130.3, 128.0, 127.92, 127.87, 119.2, 110.2, 83.4, 47.0, 24.7 ppm; **^{11}B NMR** (193 MHz, Chloroform-*d*) δ 30.02 ppm; **HRMS (ESI)** calculated $[M+H]^+$ for $C_{22}H_{25}O_2NB^+$ = 346.1973, found: 346.1968.

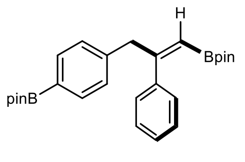

(*Z*)-4,4,5,5-tetramethyl-2-(4-(2-phenyl-3-(4,4,5,5-tetramethyl-1,3,2-dioxaborolan-2-yl)allyl)phenyl)-1,3,2-dioxaborolane (56): This compound was synthesized according to **General procedure B**, petroleum ether/ethyl acetate = 20:1, white solid, isolated yield: 130.2 mg, 73%; **^{1}H NMR** (600 MHz, Chloroform-*d*) δ 7.69 (d, *J* = 7.9 Hz, 2H), 7.25 - 7.23 (m, 5H), 7.16 (d, *J* = 7.9 Hz, 2H), 5.32 (t, *J* = 1.4 Hz, 1H), 3.79 (s, 2H), 1.33 (s, 12H), 1.10 (s, 12H); **^{13}C NMR** (151 MHz, Chloroform-*d*) δ 160.3, 142.7, 142.3, 134.9, 129.1, 128.1, 127.7, 127.5, 83.8, 83.2, 47.1, 25.0, 24.7 ppm; **^{11}B NMR** (193 MHz, Chloroform-*d*) δ 30.98 ppm; **HRMS (ESI)** calculated $[M+H]^+$ for $C_{27}H_{37}O_4B_2^+$ = 447.2873, found: 447.2873.

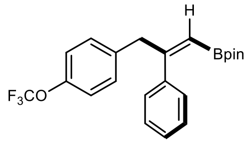

(*Z*)-4,4,5,5-tetramethyl-2-(2-phenyl-3-(4-(trifluoromethoxy)phenyl)prop-1-en-1-yl)-1,3,2-dioxaborolane (57): This compound was synthesized according to **General procedure B**, petroleum ether/ethyl acetate = 40:1, colorless oil, isolated yield: 100.2 mg, 62%; **^{1}H NMR** (600 MHz, Chloroform-*d*) δ 7.28 - 7.21 (m, 5H), 7.18 - 7.13 (m, 2H), 7.10 - 7.06 (m, 2H), 5.38 - 5.28 (m, 1H), 3.76 (s, 2H), 1.11 (s, 12H); **^{13}C NMR** (151 MHz, Chloroform-*d*) δ 160.0, 147.8, 142.4, 137.7, 130.8, 128.0, 127.8, 127.7, 120.9, 120.62 (q, *J* = 256.7 Hz), 83.3 46.2 24.7ppm; **^{11}B NMR** (193 MHz, Chloroform-*d*) δ 30.41 ppm; **^{19}F NMR** (565 MHz, Chloroform-*d*) δ -57.86 ppm; **HRMS (ESI)** calculated $[M+H]^+$ for $C_{22}H_{25}O_3F_3B^+$ = 405.1843, found: 405.1852.

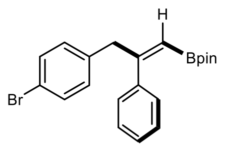

(Z)-2-(3-(4-bromophenyl)-2-phenylprop-1-en-1-yl)-4,4,5,5-tetramethyl-1,3,2-dioxaborolane (58): This compound was synthesized according to **General procedure B**, petroleum ether/ethyl acetate = 40:1, white solid, isolated yield: 124.2 mg, 78%; **^{1}H NMR** (600 MHz, Chloroform-*d*) δ 7.35 (d, *J* = 8.4 Hz, 2H), 7.27 - 7.21 (m, 5H), 7.01 (d, *J* = 8.4 Hz, 2H), 5.31 (t, *J* = 1.4 Hz, 1H), 3.72 (s, 2H), 1.11 (s, 12H); **^{13}C NMR** (151 MHz, Chloroform-*d*) δ 159.9, 142.4, 137.9, 131.5, 131.3, 128.1, 127.8, 127.7, 120.2, 83.3, 46.3, 24.7 ppm; **^{11}B NMR** (193 MHz, Chloroform-*d*) δ 29.97 ppm; **HRMS (ESI)** calculated [M+H]$^+$ for $C_{21}H_{25}O_2BrB^+$ = 399.1126, found: 399.1123.

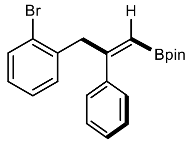

(*Z*)-2-(3-(2-bromophenyl)-2-phenylprop-1-en-1-yl)-4,4,5,5-tetramethyl-1,3,2-dioxaborolane (59): This compound was synthesized according to **General procedure B**, petroleum ether/ethyl acetate = 40:1, white solid, isolated yield: 79.6 mg, 50%; **^{1}H NMR** (600 MHz, Chloroform-*d*) δ 7.52 (d, *J* = 8.7 Hz, 1H), 7.36 - 7.33 (m, 2H), 7.31 - 7.26 (m, 3H), 7.22 - 7.19 (m, 2H), 7.07 - 7.05 (m, 1H), 5.13 (t, *J* = 1.5 Hz, 1H), 3.89 (d, *J* = 1.5 Hz, 2H), 1.09 (s, 12H); **^{13}C NMR** (151 MHz, Chloroform-*d*) δ 158.8, 142.9, 138.5, 133.0, 131.9, 128.2, 128.0, 127.8, 127.6, 127.4, 125.5, 83.1, 46.4, 24.7 ppm; **^{11}B NMR** (193 MHz, Chloroform-*d*) δ 30.57 ppm; **HRMS (ESI)** calculated [M+H]$^+$ for $C_{21}H_{25}O_2BrB^+$ = 399.1126, found: 399.1125.

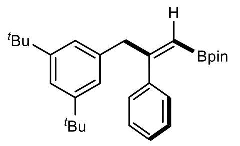

(*Z*)-2-(3-(3,5-di-tert-butylphenyl)-2-phenylprop-1-en-1-yl)-4,4,5,5-tetramethyl-1,3,2-dioxaborolane (60): This compound was synthesized according to **General procedure B**, petroleum ether/ethyl acetate = 40:1, colorless oil, isolated yield: 150.3 mg, 87%; **^{1}H NMR** (600 MHz, Chloroform-*d*) δ 7.28 - 7.23 (m, 5H), 7.21 (m, 1H), 6.94 (d, *J* = 1.9 Hz, 2H), 5.33 (t, *J* = 1.4 Hz, 1H), 3.76 (s, 2H), 1.26 (s, 18H), 1.10 (s, 12H); **^{13}C NMR** (151 MHz, Chloroform-*d*) δ 161.5, 150.5, 143.2, 137.8, 128.2, 127.7, 127.3, 123.9, 120.1, 83.1, 47.6, 34.8, 31.6, 24.7 ppm; **^{11}B NMR** (193 MHz, Chloroform-*d*) δ 29.66 ppm; **HRMS (ESI)** calculated [M+H]$^+$ for $C_{29}H_{42}O_2B^+$ = 433.3272, found: 433.3270.

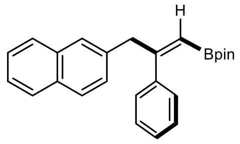

(*Z*)-4,4,5,5-tetramethyl-2-(3-(naphthalen-2-yl)-2-phenylprop-1-en-1-yl)-1,3,2-dioxaborolane (61): This compound was synthesized according to **General procedure B**, petroleum ether/ethyl acetate = 40:1, white solid, isolated yield: 120.0 mg, 81%; **^{1}H NMR** (600 MHz, Chloroform-*d*) δ 7.80 - 7.71 (m, 3H), 7.59 (d, *J* = 1.1 Hz, 1H), 7.43 - 7.38 (m, 2H), 7.33 -7.27 (m, 3H), 7.27 -7.23 (m, 3H), 5.35 (t, *J* = 1.4 Hz, 1H), 3.93 (s, 2H), 1.09 (s, 12H); **^{13}C NMR** (151 MHz, Chloroform-*d*) δ 160.5, 142.8, 136.5, 133.7, 132.3, 128.2, 128.08, 128.07, 128.0, 127.8, 127.7, 127.6, 125.9, 125.4, 83.2, 47.0, 24.7 ppm; **^{11}B NMR** (193 MHz, Chloroform-*d*) δ 30.11 ppm; HRMS (ESI) calculated [M+H]$^+$ for $C_{25}H_{28}O_2B^+$ = 371.2177, found: 371.2183.

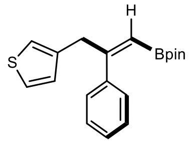

(*Z*)-4,4,5,5-tetramethyl-2-(2-phenyl-3-(thiophen-3-yl)prop-1-en-1-yl)-1,3,2-dioxaborolane (62): This compound was synthesized according to **General procedure B**, petroleum ether/ethyl acetate = 40:1, colorless oil, isolated yield: 71.7 mg, 55%; **^{1}H NMR** (600 MHz, Chloroform-*d*) δ 7.28 -7.23 (m, 5H), 7.19 (m, 1H), 6.94 - 6.91 (m, 1H), 6.89 (dd, *J* = 4.9, 1.3 Hz, 1H), 5.38 (t, *J* = 1.4 Hz, 1H), 3.78 (s, 2H), 1.11 (s, 12H); **^{13}C NMR** (151 MHz, Chloroform-*d*) δ 160.0, 142.7, 139.2, 129.0, 128.0, 127.8, 127.6, 125.3, 122.2, 83.2, 41.3, 24.7 ppm; **^{11}B NMR** (193 MHz, Chloroform-*d*) δ 30.04 ppm; **HRMS (ESI)** calculated [M+H]$^+$ for $C_{19}H_{24}O_2SB^+$ = 327.1585, found: 327.1581.

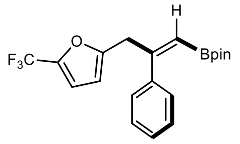

(Z)-4,4,5,5-tetramethyl-2-(2-phenyl-3-(5-(trifluoromethyl)furan-2-yl)prop-1-en-1-yl)-1,3,2-dioxaborolane (63): This compound was synthesized according to **General procedure B**, petroleum ether/ethyl acetate = 40:1, yellow oil, isolated yield: 107.4 mg, 71%; **^{1}H NMR** (600 MHz, Chloroform-*d*) δ 7.27 (m, 5H), 6.62 (d, *J* = 3.4 Hz, 1H), 6.04 (d, *J* = 3.4 Hz, 1H), 5.47 (t, *J* = 1.3 Hz, 1H), 3.83 (s, 2H), 1.12 (s, 12H); **^{13}C NMR** (151 MHz, Chloroform-*d*) δ 156.06, 156.05, 155.8, 141.6, 140.71 (q, *J* = 42.8 Hz), 128.0, 127.9, 119.3 (q, *J* = 267.3 Hz), 112.53 (q, *J* = 2.9 Hz), 108.3, 83.4, 39.1, 24.7 ppm; **^{11}B NMR** (193 MHz, Chloroform-*d*) δ 29.77 ppm; **^{19}F NMR** (565 MHz, Chloroform-*d*) δ -63.86 ppm; **HRMS (ESI)** calculated [M+H]$^+$ for $C_{20}H_{23}F_3BO_3^+$ = 379.1687, found: 379.1681.

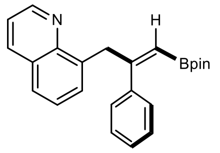

(*Z*)-8-(2-phenyl-3-(4,4,5,5-tetramethyl-1,3,2-dioxaborolan-2-yl)allyl)quinoline (64): This compound was synthesized according to **General procedure B**, petroleum ether/ethyl acetate = 10:1, white solid, isolated yield: 130.6 mg, 88%; **^{1}H NMR** (600 MHz, Chloroform-*d*) δ 8.91 (m, 1H), 8.10 (m, 1H), 7.66 (m, 1H), 7.61 - 7.56 (m, 1H), 7.47 -7.39 (m, 3H), 7.36 (m, 1H), 7.27 - 7.22 (m, 3H), 5.28 (t, *J* = 1.4 Hz, 1H), 4.53 (s, 2H), 1.11 (s, 12H); **^{13}C NMR** (151Hz, Chloroform-*d*) δ 160.9, 149.6, 147.0, 143.2, 138.1, 136.3, 130.3, 128.5, 128.3, 127.7, 127.4, 126.5, 126.4, 121.0, 83.1, 41.0, 24.7 ppm; **^{11}B NMR** (193 MHz, Chloroform-*d*) δ 30.76 ppm; **HRMS (ESI)** calculated [M+H]$^+$ for $C_{24}H_{27}O_2NB^+$ = 372.2129, found: 372.2124.

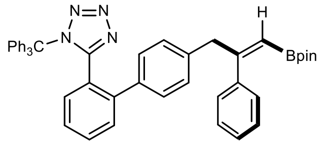

(*Z*)-5-(4'-(2-phenyl-3-(4,4,5,5-tetramethyl-1,3,2-dioxaborolan-2-yl)allyl)-[1,1'-biphenyl]-2-yl)-1-trityl-1H-tetrazole (65): This compound was synthesized according to **General procedure B**, petroleum ether/ethyl acetate = 10:1, white solid, isolated yield: 225.9 mg, 80%; **^{1}H NMR** (600 MHz, Chloroform-*d*) δ 7.88 (d, *J* = 7.7 Hz, 1H), 7.49 - 7.44 (m, 1H), 7.43 - 7.37 (m, 2H), 7.31 (m, 3H), 7.26 - 7.21 (m, 11H), 7.01 -7.69 (m, 2H), 6.93 -6.89 (m, 8H), 5.32 (t, *J* = 1.4 Hz, 1H), 3.66 (s, 2H), 1.10 (s, 12H); **^{13}C NMR** (151 MHz, Chloroform-*d*) δ 164.3, 160.2, 142.9, 142.3, 141.4, 139.0, 137.5, 130.9, 130.4, 130.3, 130.0, 129.3, 129.1, 128.3, 128.1, 127.8, 127.7, 127.5, 127.4, 126.5, 83.1, 83.0, 46.6, 24.7 ppm; **^{11}B NMR** (193 MHz, Chloroform-*d*) δ 30.73 ppm; **HRMS (ESI)** calculated [M+Na]$^+$ for $C_{47}H_{43}O_2N_4BNa^+$ = 729.3371, found: 729.3374.

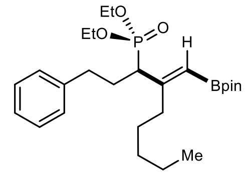

Diethyl(*E*)-(1-phenyl-4-((4,4,5,5-tetramethyl-1,3,2-dioxaborolan-2-yl)methylene)nonan-3-yl)phosphonate (66): This compound was synthesized according to **General procedure B** (**L5** instead of **L8**.), petroleum ether/ethyl acetate = 3:1, colorless oil, isolated yield: 108.7 mg, 57%; ^{1}**H NMR** (600 MHz, Chloroform-*d*) δ 7.29 -7.24 (m, 2H), 7.19 - 7.16 (m, 3H), 5.45 (d, *J* = 5.2 Hz, 1H), 4.09 - 4.02 (m, 4H), 2.82 -2.69 (m, 2H), 2.65 - 2.59 (m, 1H), 2.53 - 2.49 (m, 1H), 2.25 - 2.19 (m, 2H), 2.09 - 2.01 (m, 1H), 1.45 - 1.41 (m, 1H), 1.34 -1.23 (m, 23H), 0.88 (t, *J* = 6.9 Hz, 3H); ^{13}**C NMR** (151 MHz, Chloroform-*d*) δ 160.9 (d, *J* = 9.0 Hz), 141.7, 128.6, 128.5, 126.1, 82.9, 62.3 (t, *J* = 6 Hz), 46.0 (d, *J* = 132.9 Hz), 36.7 (d, *J* = 4.5 HZ), 34.0 (d, *J* = 15 Hz), 32.0, 31.1 (d, *J* = 4.5 Hz), 29.1 (d, *J* = 3 Hz), 25.0 (d, *J* = 28.5), 22.6, 16.6 (t, *J* = 6 HZ), 14.2 ppm; ^{11}**B NMR** (193 MHz, Chloroform-*d*) δ 29.84 ppm; ^{31}**P NMR (243 MHz, Chloroform-*d*)** δ 28.11 ppm; **HRMS (APCI)** calculated $[M+H]^+$ for $C_{26}H_{45}O_5BP^+$ = 479.3092, found: 479.3100.

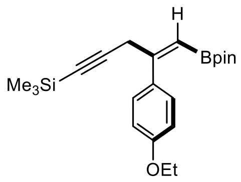

(*Z*)-(4-(4-ethoxyphenyl)-5-(4,4,5,5-tetramethyl-1,3,2-dioxaborolan-2-yl)pent-4-en-1-yn-1-yl)trimethylsilane (67): This compound was synthesized according to **General procedure B**, petroleum ether/ethyl acetate = 40:1, colorless oil, isolated yield: 84.3 mg, 55%; ^{1}**H NMR** (600 MHz, Chloroform-*d*) δ 7.03 (d, *J* = 8.5 Hz, 2H), 6.65 (d, *J* = 8.6 Hz, 2H), 5.68 (s, 1H), 3.87 (q, *J* = 7.0 Hz, 2H), 3.21 (s, 2H), 1.25 (t, *J* = 7.0 Hz, 3H), 1.00 (s, 12H), -0.00 (s, 9H); ^{13}**C NMR** (151 MHz, Chloroform-*d*) δ 158.8, 154.2, 134.3, 129.1, 113.8, 103.5, 88.7, 83.2, 63.6, 31.3, 24.8, 15.0 ppm; ^{11}**B NMR** (193 MHz, Chloroform-*d*) δ 30.80 ppm; ^{29}**Si NMR** (119 MHz, Chloroform-*d*) δ -18.75 ppm; **HRMS (APCI)** calculated $[M+H-H_2O]^+$ for $C_{22}H_{33}O_3BSi^+$ = 385.2365, found: 385.2377.

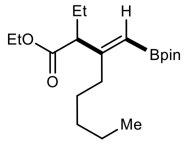

Ethyl(*E*)-2-ethyl-3-((4,4,5,5-tetramethyl-1,3,2-dioxaborolan-2-yl)methylene)octanoate (68): This compound was synthesized according to **General procedure B** (**L5** instead of **L8**.), petroleum ether/ethyl acetate = 40:1, colorless oil, isolated yield: 128.1 mg, 95%; ^{1}**H NMR** (400 MHz, Chloroform-*d*) δ 5.28 (s, 1H), 4.18 - 4.01 (m, 2H), 2.90 (dd, *J* = 8.9, 6.1 Hz, 1H), 2.44 - 2.31 (m, 2H), 1.85 - 1.77 (m, 1H), 1.64 - 1.49 (m, 1H), 1.44 - 1.35 (m, 2H), 1.34 - 1.06 (m, 21H), 0.85 (td, *J* = 7.1, 3.0 Hz, 6H); ^{13}**C NMR** (101 MHz, Chloroform-*d*) δ 173.6, 163.1, 80.8, 62.1, 54.7, 35.1, 31.8, 29.4, 25.1, 24.90, 24.87, 22.5, 14.2, 14.1, 12.4 ppm; ^{11}**B NMR** (193 MHz, Chloroform-*d*) 30.69 ppm; **HRMS (ESI)** calculated $[M+H]^+$ for $C_{19}H_{36}BO_4^+$ = 339.2710, found: 339.2708.

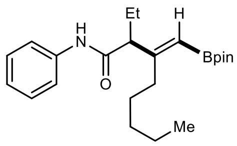

(E) -2-ethyl-N-phenyl-3-((4,4,5,5-tetramethyl-1,3,2-dioxaborolan-2-yl)methylene)octanamide (69): This compound was synthesized according to **General procedure B** , petroleum ether/ethyl acetate = 10:1, colorless oil, isolated yield: 95.5 mg, 62%; ^{1}**H NMR** (600 MHz, Chloroform-*d*) δ 7.52 - 7.42 (m, 2H), 7.33 - 7.26 (m, 3H), 7.08 (m, 1H), 5.44 (s, 1H), 2.98 (t, *J* = 7.4 Hz, 1H), 2.58 - 2.53 (m, 1H), 2.31 - 2.26 (m, 1H), 2.02 - 1.92 (m, 1H), 1.75 - 1.68 (m, 1H), 1.62 (m, 1H), 1.52 -1.34 (m, 2H), 1.28 (d, *J* = 1.6 Hz, 15H), 0.96 (t, *J* = 7.4 Hz, 3H), 0.88 -0.78 (m, 3H); ^{13}**C NMR** (151 MHz, Chloroform-*d*) δ 171.0, 164.6, 138.0, 129.1, 124.3, 119.9, 83.2, 60.0, 34.7, 32.1, 30.2, 25.1, 25.0, 24.2, 22.5, 14.2, 12.6 ppm; ^{11}**B NMR** (193 MHz, Chloroform-*d*) δ 30.45 ppm; **HRMS (ESI)** calculated $[M + H]^+$ for $C_{23}H_{37}NO_3B^+$ = 386.2861, found: 386.2863.

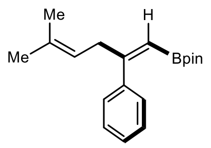

(Z)-4,4,5,5-tetramethyl-2-(5-methyl-2-phenylhexa-1,4-dien-1-yl)-1,3,2-dioxaborolane (70): This compound was synthesized according to **General procedure B** (**L5** instead of **L8**.), petroleum ether/ethyl acetate = 40:1, colorless oil, isolated yield: 47.7 mg, 40%; **^{1}H NMR** (600 MHz, Chloroform-*d*) δ 7.27 - 7.25 (m, 5H), 5.45 (s, 1H), 5.21 (t, *J* = 6.5 Hz, 1H), 3.12 (d, *J* = 7.3 Hz, 2H), 1.71 (s, 3H), 1.58 (s, 3H), 1.12 (s, 12H); **^{13}C NMR** (101 MHz, Chloroform-*d*) δ 161.0, 143.5, 134.0, 127.9, 127.7, 127.4, 121.3, 83.1, 38.9, 25.9, 24.7, 17.8 ppm; **^{11}B NMR** (193 MHz, Chloroform-*d*) δ 30.69 ppm; **HRMS (ESI)** calculated $[M+H]^+$ for $C_{19}H_{28}BO_2^+$ = 299.2177, found: 299.2171.

4. Synthetic applications

4.1 Synthesis of *E* and *Z*-allyl alcohols

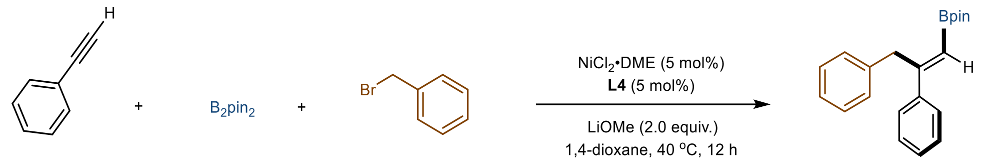

79 **2** **3** **6:** 65%, *syn:anti* = 9:1

This compound was synthesized according to **General procedure A**, 10 mmol scale, colorless oil, isolated yield: 2.2 g, 69%.

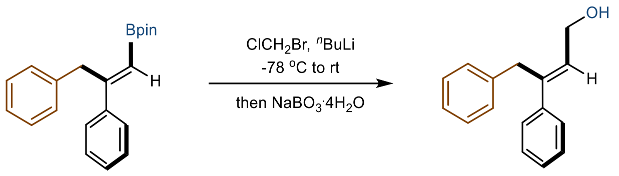

6 **71:** 58%

According to the following procedure[5]: To an oven-dried round bottom flask containing a stirring bar was added a solution of **6** (160.0 mg, 0.5 mmol, 1.0 equiv.) and bromochloromethane (194.9 μL, 3.0 mmol, 6.0 equiv.) in THF (4.0 mL) under N_2 atmosphere. The reaction mixture was cooled to -78 °C. ^{n}BuLi (1.00 mL,2.5 mmol, 5.0 equiv., 2.5 M in hexane) was slowly added and the solution was stirred for 10 min at the same temperature. The reaction mixture was warmed to room temperature and additionally stirred for 12 h. Then the reaction was quenched with saturated aqueous solution of NaCl (5 mL) and extracted with EtOAc (3 ×5 mL), dried over Na_2SO_4, concentrated under reduced pressure. Then the crude material and $NaBO_3{\cdot}4H_2O$ (461.6 mg, 3.0 mmol, 6.0 equiv.) were dissolved in THF/H_2O (12mL, 2:1). After stirred at ambient temperature 16 h, the reaction was quenched by saturated aqueous solution of NaCl (5 mL). The mixture was extracted with Et_2O, dried over Na_2SO_4, concentrated under reduced pressure. The mixture was purified by flash column chromatography to provide the title compound **71** as a colorless oil.

^{1}H NMR (600 MHz, Chloroform-*d*) δ 7.37 (dd, *J* = 8.3, 1.4 Hz, 2H), 7.28 -7.21 (m, 5H), 7.16 -7.15 (m, 3H), 6.16 (t, *J* = 6.7 Hz, 1H), 4.39 (d, *J* = 6.7 Hz, 2H), 3.91 (s, 2H), 1.44 (s, 1H); **^{13}C NMR** (151 MHz, Chloroform-*d*) δ 142.1, 140.5, 139.4, 128.9, 128.7, 128.5, 128.2, 127.5, 126.6, 126.3, 60.2, 36.2 ppm.

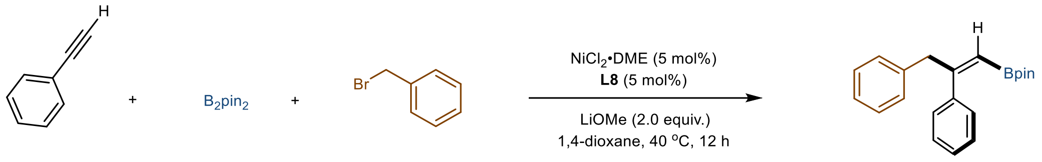

79 **2** **3** **36:** 75%, *anti:syn* = 12:1

This compound was synthesized according to **General procedure B**, 10 mmol scale, colorless oil, isolated yield: 2.4 g, 75%.

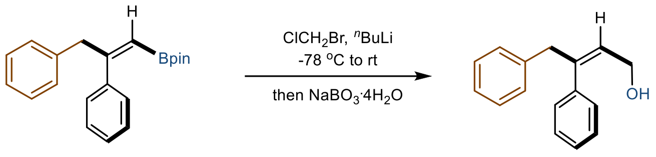

36 **72:** 56%

To an oven-dried round bottom flask containing a stirring bar was added a solution of **36** (160.0 mg, 0.5 mmol, 1.0 equiv.) and bromochloromethane (194.9 μL, 3.0 mmol, 6.0 equiv.) in THF (4.0 mL) under N_2 atmosphere. The reaction mixture was cooled to -78 °C. ^{n}BuLi (1.00 mL,2.5 mmol, 5.0 equiv., 2.5 M in hexane) was slowly added and the solution was stirred for 10 min at the same temperature. The reaction mixture was warmed to room temperature and additionally stirred for 12 h. Then the reaction was quenched with saturated aqueous solution of NaCl (5 mL) and extracted with EtOAc (3 ×5 mL), dried over Na_2SO_4, concentrated under reduced pressure. Then the crude material and $NaBO_3{\cdot}4H_2O$ (461.6 mg, 3.0 mmol, 6.0 equiv.) were dissolved in THF/H_2O (12mL, 2:1). After stirred at ambient temperature 16 h, the reaction was quenched by saturated aqueous solution of NaCl (5 mL). The mixture was extracted with Et_2O, dried over Na_2SO_4, concentrated under reduced pressure. The mixture was purified by flash column chromatography (petroleum ether/ethyl acetate = 10:1) to provide the title compound **72** as a colorless oil.

^{1}H NMR (600 MHz, Chloroform-*d*) δ 7.29 - 7.22 (m, 5H), 7.17 (m, 1H), 7.13 (d, *J* = 6.9 Hz, 2H), 7.07 (d, *J* = 6.8 Hz, 2H), 5.66 (t, *J* = 6.9 Hz, 1H), 4.06 (d, *J* = 6.9 Hz, 2H), 3.68 (s, 2H), 1.25 (s, 1H); **^{13}C NMR** (151 MHz, Chloroform-*d*) δ 143.9, 140.0, 139.0, 129.3, 128.43, 128.37, 128.2, 127.6, 127.3, 126.3, 60.5, 45.4 ppm.

4.2 Synthesis of a key pharmacophore in pharmaceutical compounds

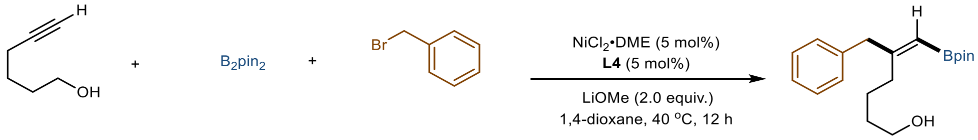

73 **2** **3** **74a:** 74%

(*E*)-5-benzyl-6-(4,4,5,5-tetramethyl-1,3,2-dioxaborolan-2-yl)hex-5-en-1-ol (74a): This compound was synthesized according to **General procedure B**, 10 mmol scale, colorless oil, petroleum ether/ethyl acetate = 10:1, isolated yield: 2.3 g, 74%;

^{1}H NMR (600 MHz, Chloroform-*d*) δ 7.26 (m, 2H), 7.18 (m, 1H), 7.16 -7.12 (m, 2H), 5.08 (s, 1H), 3.65 (t, *J* = 5.9 Hz, 2H), 3.43 (s, 2H), 2.38 -2.32 (m, 2H), 2.08 (s, 1H), 1.60 -1.52 (m, 4H), 1.25 (s, 12H); **^{13}C NMR** (151 MHz, Chloroform-*d*) δ 165.9, 139.2, 129.5, 128.4, 126.2, 83.0, 62.0, 46.1, 33.6, 31.8, 25.5, 24.8 ppm; **^{11}B NMR**

(193 MHz, Chloroform-*d*) δ 29.92 ppm; **HRMS (ESI)** calculated $[M+H]^+$ for $C_{19}H_{30}O_3B^+$ = 317.2283, found: 317.2289.

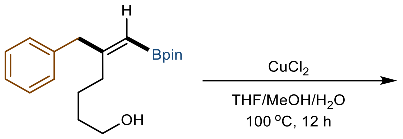

74a **74b**: 82%

According to the following procedure[6]: a 25 mL Schlenk tube was charged with **74a** (134 mg, 0.4 mmol), $CuCl_2$ (16.2 mg, 1.2 mmol, 3.0 equiv.), THF (2 mL), MeOH (2 mL) and H_2O (2 mL). The mixture was heated at 100 °C and monitored by TLC. 36 h later, stop heating and the reaction was cooled to room temperature. The organic phase was washed with brine and dried over anhydrous $MgSO_4$. The mixture was purified by flash column chromatography (petroleum ether/ethyl acetate = 10:1) to provide the title compound **74b** (72.4 mg, 82%) as a colorless oil.

^{1}H NMR (600 MHz, Chloroform-*d*) δ 7.30 (m, 2H), 7.22 (m, 1H), 7.16 (d, J = 7.4 Hz, 2H), 5.85 (s, 1H), 3.63 (t, J = 6.5 Hz, 2H), 3.37 (s, 2H), 2.18 (t, J = 7.7 Hz, 2H), 1.59 -1.54 (m, 2H), 1.50 -1.45 (m, 2H), 1.34 (s, 1H); **^{13}C NMR** (151 MHz, Chloroform-*d*) δ 141.9, 138.3, 129.1, 128.6, 126.7, 114.5, 62.8, 41.2, 32.4, 29.7, 23.2 ppm; **HRMS (ESI)** calculated $[M+H]^+$ for $C_{13}H_{18}ClO_2^+$ = 225.1041, not found.

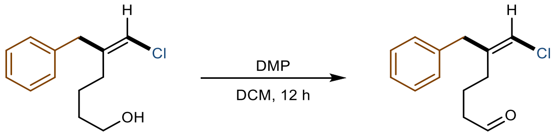

74b **74c**: 80%

According to the following procedure[7]: To a solution of **74b** (67.2 mg, 0.3 mmol) in DCM (2 mL) was added Dess-Martin periodinane (168.9 mg, 11.2 mmol). The mixture was stirred at room temperature for 12 h, then poured into saturated aqueous $NaHCO_3/Na_2S_2O_3$, and stirring was continued for another 1 h. The insoluble material was removed by filtration. The DCM layer was separated from the filtrate, washed with saturated aqueous $NaHCO_3$ and brine, dried over Na_2SO_4, filtered, and concentrated in vacuo to give **74c** (52.8 mg, 80%) as a colorless oil

^{1}H NMR (600 MHz, Chloroform-*d*) δ 9.75 (s, 1H), 7.30 (m, 2H), 7.23 (m, 1H), 7.16 (d, J = 7.4 Hz, 2H), 5.90 (s, 1H), 3.38 (s, 2H), 2.46 -2.41 (m, 2H), 2.19 (t, J = 7.8 Hz, 2H), 1.75 (q, J = 7.6 Hz, 2H); **^{13}C NMR** (151 MHz, Chloroform-*d*) δ 202.3, 141.2, 138.1, 129.1, 128.7, 126.8, 115.2, 43.3, 41.1, 29.2, 19.5 ppm; **HRMS (ESI)** calculated $[M+H]^+$ for $C_{13}H_{16}ClO_2^+$ = 223.0884, not found.

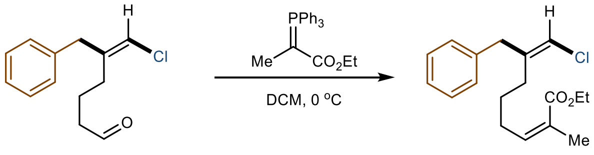

74c **74**: 85%

According to the following procedure[8]: Carbethoxyethylidene triphenylphosphorane (153 mg, 0.4 mmol) was added to a solution of **74c** (44.4 mg, 0.2 mmol) in dichloromethane (3 mL) at 0 °C and stirred for 3 h at room temperature. The resulting reaction mixture was then concentrated under reduced pressure and the crude residue was finally purified by flash column chromatography over silica gel to yield the desired ester **74** as a colorless oil (52.0 mg, 85 %).

^{1}H NMR (600 MHz, Chloroform-*d*) δ 7.29 (d, *J* = 7.6 Hz, 2H), 7.24 (d, *J* = 7.5 Hz, 1H), 7.16 (d, *J* = 7.4 Hz, 2H), 6.78 -6.67 (m, 1H), 5.87 (s, 1H), 4.19 (q, *J* = 7.4 Hz, 2H), 3.37 (s, 2H), 2.17 (dt, *J* = 14.8, 7.9 Hz, 4H), 1.81 (s, 3H), 1.54 (t, *J* = 7.9 Hz, 2H), 1.30 (t, *J* = 7.3 Hz, 3H); **^{13}C NMR** (151 MHz, Chloroform-*d*) δ 168.3, 141.7, 138.2, 129.1, 128.7, 128.3, 126.8, 114.7, 60.6, 41.3, 29.9, 28.5, 26.1, 14.4, 12.5 ppm.

4.3 Synthesis of (*E*, *E*)-*α*-homofarnesene

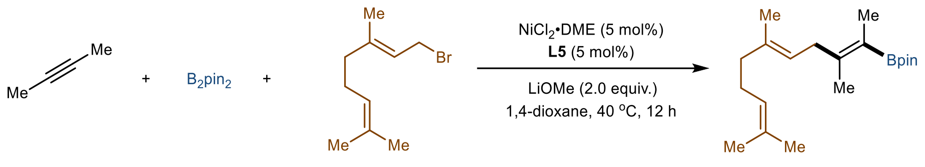

75 **2** **76** **77**: 55%, *anti:syn* = 6:1

4,4,5,5-tetramethyl-2-((2*Z*,5*E*)-3,6,10-trimethylundeca-2,5,9-trien-2-yl)-1,3,2-dioxaborolane (77): This compound was synthesized according to **General procedure B** (**L5** instead of **L8**), 0.4 mmol scale, colorless oil, petroleum ether/ethyl acetate = 40:1, isolated yield: 69.7 mg, 55%; **^{1}H NMR** (600 MHz, Chloroform-*d*) δ 5.09 - 5.04 (m, 2H), 2.80 (d, *J* = 7.1 Hz, 2H), 2.07 (d, *J* = 7.8 Hz, 2H), 2.01 -1.97 (m, 2H), 1.92 (s, 3H), 1.70 (s, 3H), 1.67 (s, 3H), 1.65 (s, 3H), 1.60 (s, 3H), 1.27 (s, 12H); **^{13}C NMR** (151 MHz, Chloroform-*d*) δ 151.2, 135.8, 131.5, 124.5, 121.8, 82.9, 39.9, 34.0, 26.8, 25.8, 25.0, 22.4, 17.8, 16.3, 16.3 ppm; **^{11}B NMR** (193 MHz, Chloroform-*d*) δ 31.05 ppm; **HRMS (ESI)** calculated $[M+H]^+$ for $C_{20}H_{36}O_2B^+$ = 319.2803, found: 319.2806.

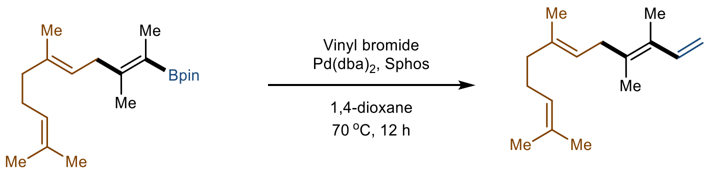

77 **78**: 69%

According to the following procedure[9]: In a Schlenk tube, $Pd(dba)_2$ (0.01 mmol) and S-Phos (0.01 mmol) were dissolved in 1,4-dioxane (1 mL). Next, and in this order, product **77** (31.7 mg, 0.1 mmol), vinyl bromide 1.0 M in THF (0.2 mL, 0.20 mmol) and aq. NaOH 2.0 M (0.17 mL, 0.33 mmol) were added and the resulting mixture was heated at 70 °C for 12 h. The solvent was removed under vacuum and the residue was diluted in Et_2O (10 mL), washed with aq. NH_4Cl (2 x 5 mL) and water (1 x 5 mL). The combined organic phases were dried with anhydrous Na_2SO_4 and concentrated in vacuo to afford the corresponding product **74** (14.9 mg, 69%).

^{1}H NMR (600 MHz, Chloroform-d) δ 6.84 (m, 1H), 5.13 (d, *J* = 17.2 Hz, 1H), 5.06 (dd, *J* = 18.2, 8.0 Hz, 2H), 4.99 (d, *J* = 10.7 Hz, 1H), 2.84 (d, *J* = 7.1 Hz, 2H), 2.09 -2.08 (m, 2H), 2.01 -1.98 (m, 2H), 1.79 (s, 3H), 1.67 (s, 6H), 1.59 (s, 3H), 1.55 (s, 3H); **^{13}C NMR** (151 MHz, Chloroform-*d*) δ 136.2, 135.9, 135.3, 131.5, 126.8, 124.4, 122.0, 111.5, 39.9, 34.4, 26.8, 25.9, 18.3, 17.8, 16.3, 13.5 ppm.

5. Mass Balance Analysis

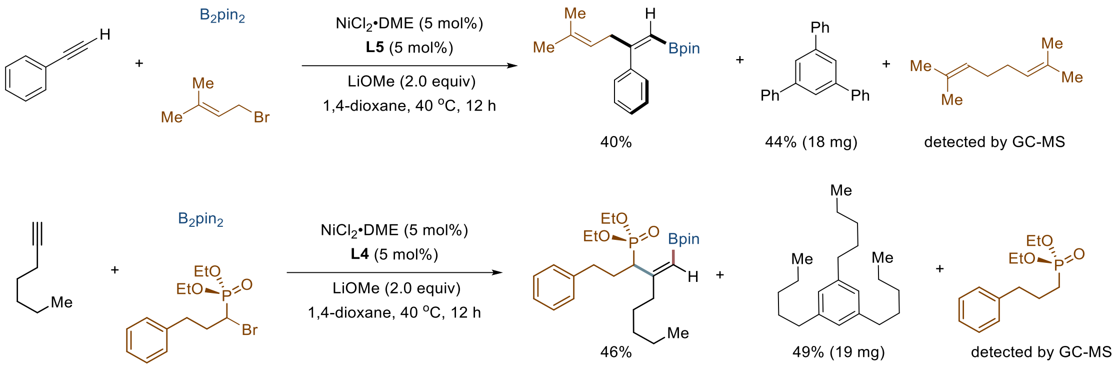

As shown above, we isolated alkyne trimers as the main side products from two representative reactions. We also observed protodehalogenation products or homocoupling products from electrophiles.

6. Mechanistic studies

6.1 Isomerization experiments

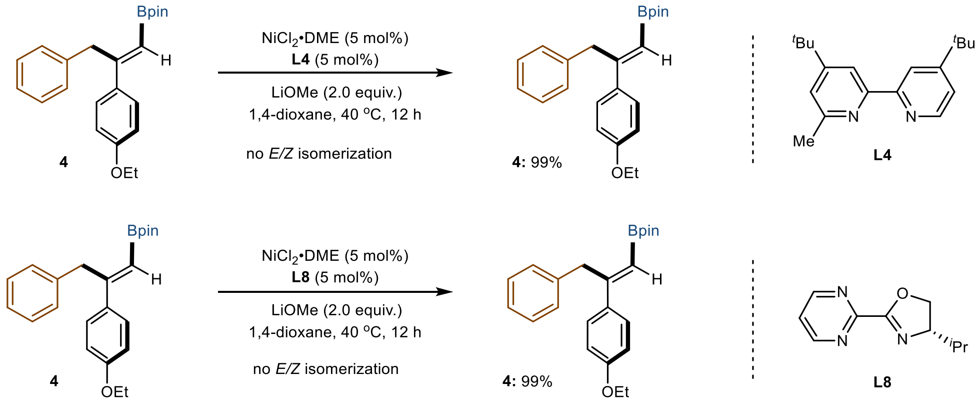

Under N_2 atmosphere, an oven-dried 10 mL reaction tube which equipped with a magnetic stir bar and sealed with a rubber stopper sequentially was added $NiCl_2$·DME (0.02 mmol, 5 mol%), **L4 or L8** (0.02 mmol, 5 mol %), LiOMe (30.2 mg, 0.8 mmol, 2.0 equiv.). Then anhydrous 1,4-dioxane (2 mL), **4** (0.4 mmol, 1.0 equiv.) were added and the mixture was stirred. After 12 h of stirring at 40 °C. The reaction mixture was analyzed by **^{1}H NMR** with an internal standard.

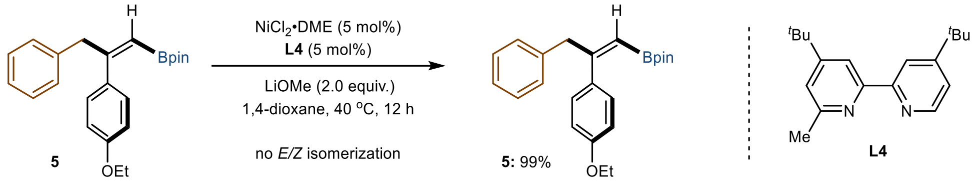

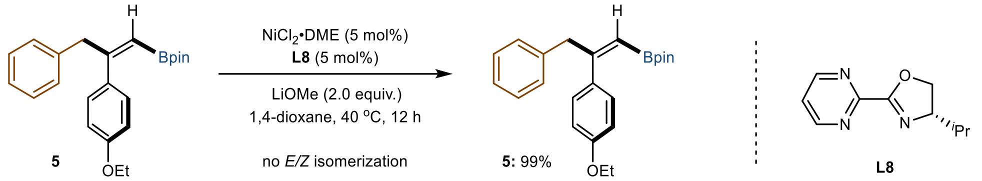

Under N_2 atmosphere, an oven-dried 10 mL reaction tube which equipped with a magnetic stir bar and sealed with a rubber stopper sequentially was added $NiCl_2$·DME (0.02 mmol, 5 mol%), **L4 or L8** (0.02 mmol, 5 mol %), LiOMe (30.2 mg, 0.8 mmol, 2.0 equiv.). Then anhydrous 1,4-dioxane (2 mL), **5** (0.4 mmol, 1.0 equiv.) were added and the mixture was stirred. After 12 h of stirring at 40 °C. The reaction mixture was analyzed by **^{1}H NMR** with an internal standard.

6.2 Quenching experiment.

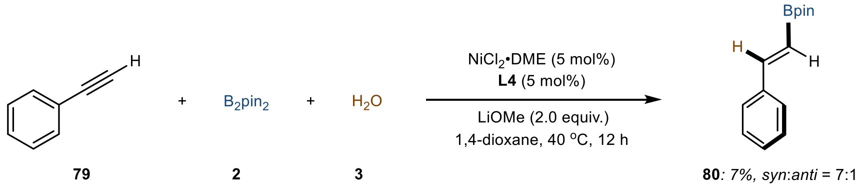

Under N_2 atmosphere, an oven-dried 10 mL reaction tube which equipped with a magnetic stir bar and sealed with a rubber stopper. Sequentially $NiCl_2$·DME (0.02 mmol, 5 mol%), **L4** (0.02 mmol, 5 mol%), B_2pin_2 (0.8 mmol, 2.0 equiv.), LiOMe (0.8 mmol, 2.0 equiv.), H_2O (2.0 mmol, 5.0 equiv.) was added. Then anhydrous 1,4-dioxane (2 mL), Phenylacetylene (0.4 mmol, 1.0 equiv.) were added and the mixture was stirred. After 12 h of stirring at 40 °C. Then the reaction mixture was cooled to room temperature, quenched with water (3 mL) and further diluted with ethyl acetate (3 mL). Finally, the mixture was extracted with ethyl acetate and the combined organic layers were dried over anhydrous Na_2SO_4, filtered, and concentrated under vacuum. The crude material was separated on a silica gel column affording the desired product.

(*E*)-2-(2,3-diphenylprop-1-en-1-yl)-4,4,5,5-tetramethyl-1,3,2-dioxaborolane (80): ^{1}H NMR (600 MHz, Chloroform-*d*) δ 7.49 (d, *J* = 7.6 Hz, 2H), 7.40 (d, *J* = 18.4 Hz, 1H), 7.34 (t, *J* = 7.5 Hz, 2H), 7.29 (t, *J* = 7.3 Hz, 1H), 6.17 (d, *J* = 18.4 Hz, 1H), 1.32 (s, 12H); **^{13}C NMR (**151 MHz, Chloroform-*d*) δ 149.7, 137.6, 129.0, 128.7, 127.2, 83.5, 25.0 ppm.

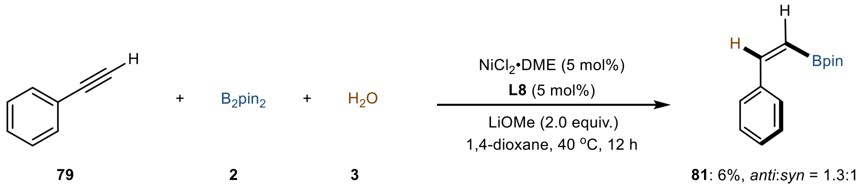

Under N_2 atmosphere, an oven-dried 10 mL reaction tube which equipped with a magnetic stir bar and sealed with a rubber stopper. Sequentially, $NiCl_2$·DME (0.02 mmol, 5 mol %), **L8** (0.02 mmol, 5 mol %), B_2pin_2 (0.8 mmol,

2.0 equiv.), LiOMe (0.8 mmol, 2.0 equiv.), H_2O (2.0 mmol, 5.0 equiv.) was added. Then anhydrous 1,4-dioxane (2 mL), phenylacetylene (0.4 mmol, 1.0 equiv.) were added and the mixture was stirred. After 12 h of stirring at 40 °C. The yields and *anti:syn* selectivities of product **81** were determined by measuring **^{1}H NMR** of the crude reaction mixture on the basis of 1,3,5-trimethoxybenzene as an internal standard and comparing with the data reported in the literature[10,11]. The **^{1}H NMR** of the crude mixture is shown in **Fig. S2.**

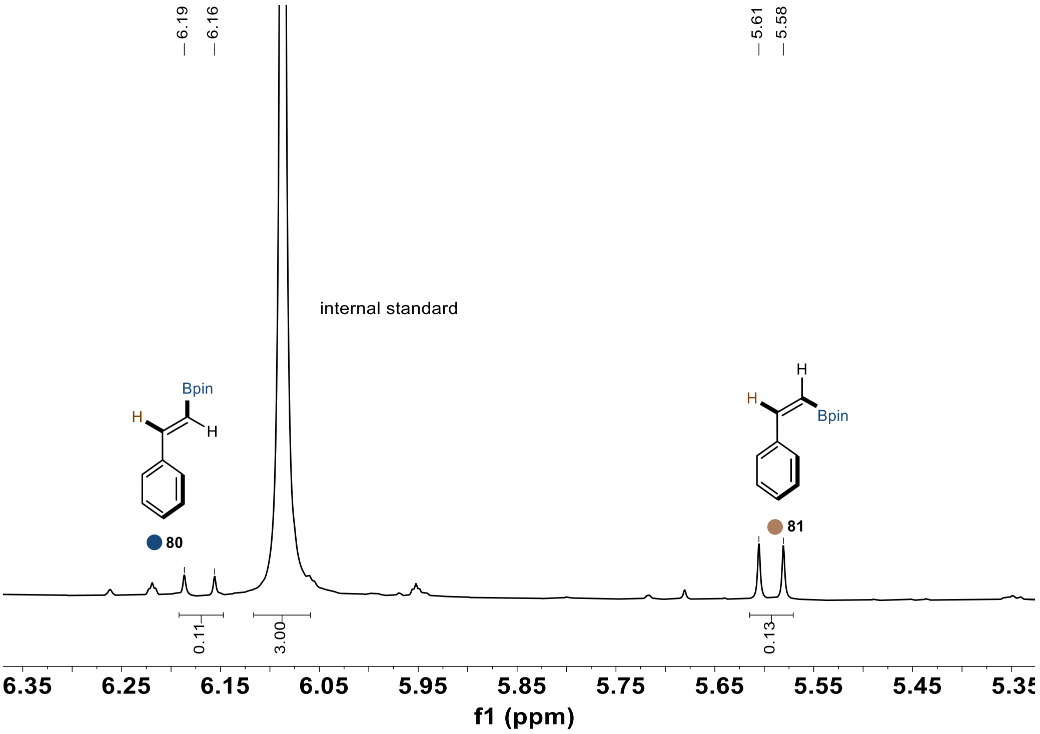

Figure S2 ^{1}H NMR of the crude reaction mixture

6.3 Proton-stoichiometric experiment.

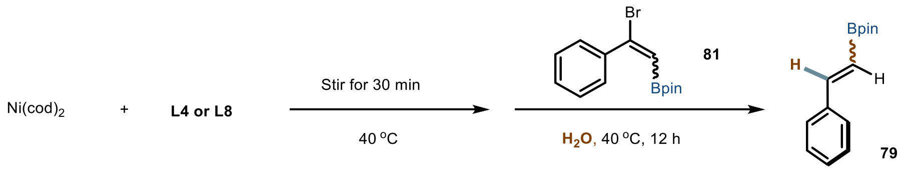

entry	**81** (*syn*:*anti*)	Ligand	**79** (yield)	**79** (*syn*:*anti*)
1	100:0	**L4**	22%	2.2: 1
2	100:0	**L8**	18%	1: 2.2
3	2:1	**L4**	25%	1.2: 1
4	2:1	**L8**	20%	1: 1
5	1:3	**L4**	30%	1.5: 1
6	1:3	**L8**	15%	1: 1.3

Under N_2 atmosphere, an oven-dried 10 mL reaction tube which equipped with a magnetic stir bar and sealed with a rubber stopper sequentially was added Ni(cod)2 (110.8 mg, 0.4 mmol, 1.0 equiv.), **L4** (112.8 mg, 0.4 mmol, 1.0 equiv.) or L8 (76.0 mg, 0.4 mmol, 1.0 equiv.), anhydrous 1,4-dioxane (1.0 mL), stirred at 30 °C for 30 min. Then 2-(2-bromo-2-phenylvinyl)-4,4,5,5-tetramethyl-1,3,2-dioxaborolane **81** (0.4 mmol, 1.0 equiv.), H_2O (2.0 mmol, 5 equiv..) were added in this order and the mixture was stirred. After stirring at 40 °C for 12 h, the resulting mixture was quenched with water (3 mL) and further diluted with ethyl acetate (3 mL). The yields and *anti:syn* selectivities of product **79** were determined by measuring **GC** of the crude reaction mixture on the basis of naphthalene as an internal standard.

6.4 BnBr-stoichiometric experiment.

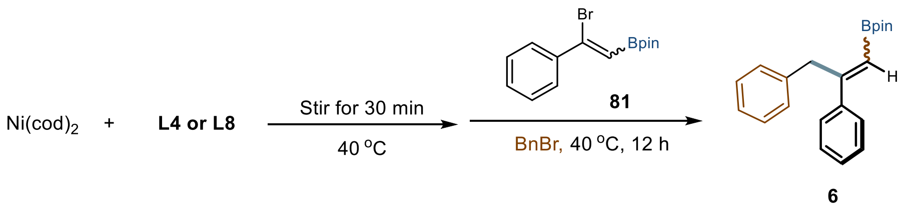

entry	**81** (*syn*:*anti*)	Ligand	**6** (yield)	**6** (*syn*:*anti*)
1	100:0	**L4**	52%	10:1
2	100:0	**L8**	69%	1:12
3	2:1	**L4**	57%	10:1
4	2:1	**L8**	64%	1:11
5	1:3	**L4**	55%	10:1
6	1:3	**L8**	68%	1:12

Under N_2 atmosphere, an oven-dried 10 mL reaction tube which equipped with a magnetic stir bar and sealed with a rubber stopper sequentially was added Ni(cod)2 (110.8 mg, 0.4 mmol, 1.0 equiv.), **L4** (112.8 mg, 0.4 mmol, 1.0 equiv.) or L8 (76.0 mg, 0.4 mmol, 1.0 equiv.), anhydrous 1,4-dioxane (1.0 mL), stirred at 30 °C for 30 min. Then 2-(2-bromo-2-phenylvinyl)-4,4,5,5-tetramethyl-1,3,2-dioxaborolane **81** (0.4 mmol, 1.0 equiv.), BnBr (0.8 mmol, 2 equiv..) were added in this order and the mixture was stirred. After stirring at 40 °C for 12 h, the resulting mixture was quenched with water (3 mL) and further diluted with ethyl acetate (3 mL). The yields and *anti:syn* selectivities of product **6** were determined by measuring **GC** of the crude reaction mixture on the basis of naphthalene as an internal standard.

6.5 Radical trapping experiments

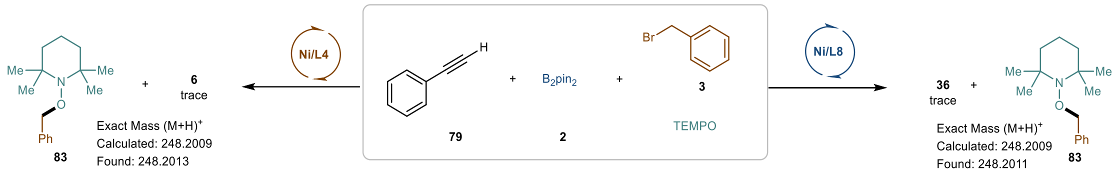

Following procedure **A** or **B**, using **79**, **2**, and **3** the addition of radical inhibitor TEMPO (1 equiv) to the reaction shut down the productive reactivity. And the TEMPO – captured benzyl radical **83** was detected by HRMS – ESI.

Target Mass

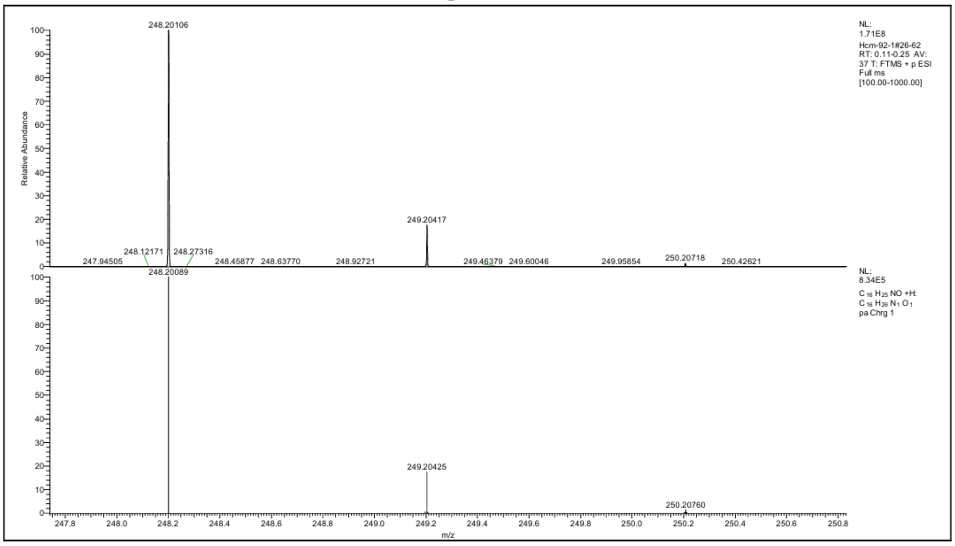

Formula:	*RDB:*	*Delta ppm:*	*Adduct ion:*
C16 H26 O N	4.5	0.681	*M+H*

Target Mass

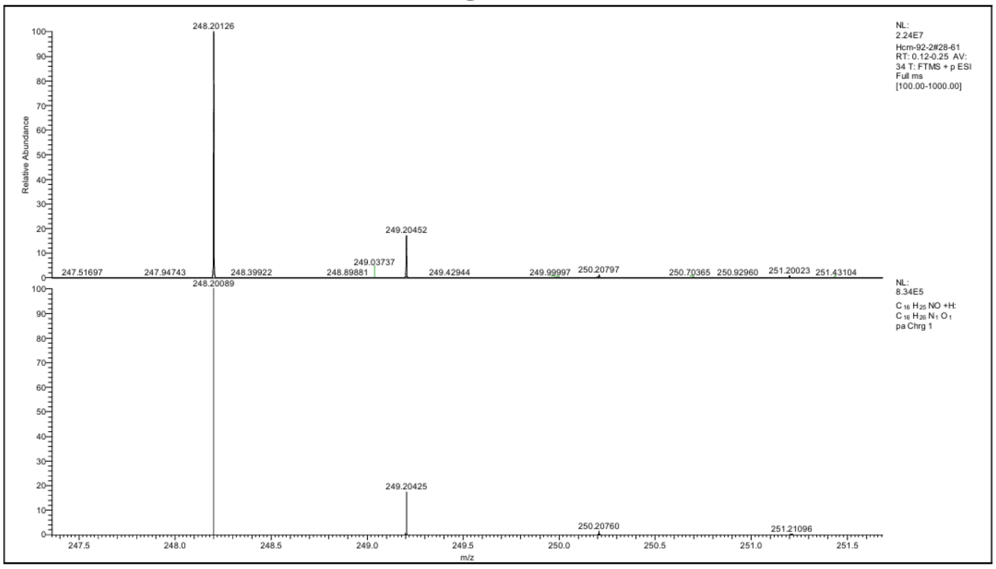

Formula:	*RDB:*	*Delta ppm:*	*Adduct ion:*
C16 H26 O N	4.5	1.487	*M+H*

6.6 Radical clock experiments

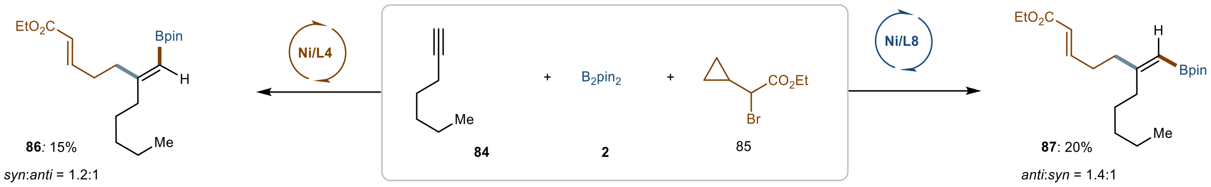

Under N_2 atmosphere, an oven-dried 10 mL reaction tube which equipped with a magnetic stir bar and sealed with a rubber stopper. Sequentially $NiCl_2 \cdot DME$ (0.02 mmol, 5 mol%), **L4/L8** (0.02 mmol, 5 mol%), B_2pin_2 (0.8 mmol, 2.0 equiv.), LiOMe (0.8 mmol, 2.0 equiv.), H_2O (2.0 mmol, 5.0 equiv.) was added. Then anhydrous 1,4-dioxane (2 mL), 1-Heptyne (0.4 mmol, 1.0 equiv.) and Ethyl 2-bromo-2-cyclopropylacetate (0.8 mmol, 2.0 equiv.) were added and the mixture was stirred. After 12 h of stirring at 40 °C. Then the reaction mixture was cooled to room temperature, quenched with water (3 mL) and further diluted with ethyl acetate (3 mL). Finally, the mixture was extracted with ethyl acetate and the combined organic layers were dried over anhydrous Na_2SO_4, filtered, and concentrated under vacuum. The crude material was separated on a silica gel column affording the desired product.

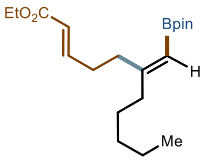

ethyl (2E,6Z)-6-((4,4,5,5-tetramethyl-1,3,2-dioxaborolan-2-yl)methylene)undec-2-enoate (86): ^{1}**H NMR** (600 MHz, Chloroform-*d*)δ 6.97 (dt, *J* = 15.6, 7.0 Hz, 1H), 5.81 (dt, *J* = 15.6, 1.5 Hz, 1H), 5.16 (s, 1H), 4.17 (q, *J* = 7.2 Hz, 2H), 2.57 – 2.47 (m, 2H), 2.30 (q, *J* = 8.8, 7.7 Hz, 2H), 2.07 (t, *J* = 7.7 Hz, 2H), 1.45 – 1.40 (m, 2H), 1.30 – 1.26 (m, 7H), 1.25 (s, 12H), 0.86 (d, *J* = 7.2 Hz, 3H); ^{13}**C NMR (**151 MHz, Chloroform-*d*) δ 166.9, 165.7, 149.2, 121.4, 82.8, 60.2, 39.2, 33.4, 32.4, 31.7, 27.6, 25.0, 22.7, 14.4, 14.1 ppm. Analytical data were identical to those previously reported.[12]

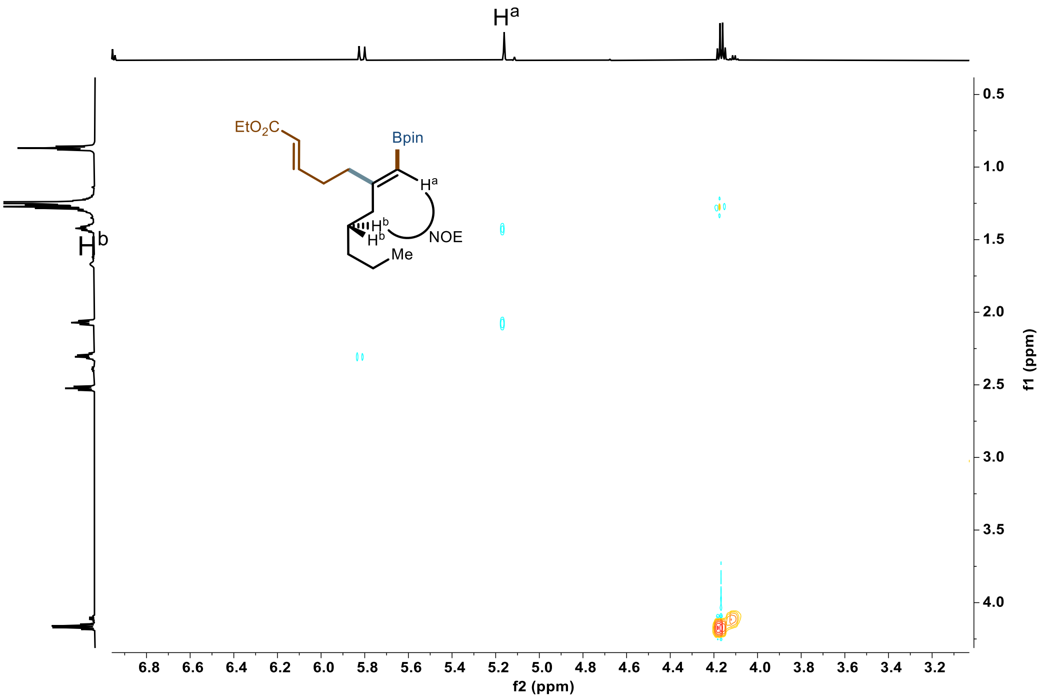

Figure S3 NOE of the compound **86**

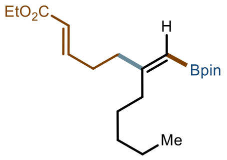

ethyl (2E,6E)-6-((4,4,5,5-tetramethyl-1,3,2-dioxaborolan-2-yl)methylene)undec-2-enoate (87): ^{1}**H NMR (600 MHz, Chloroform-*d*)** δ 6.97 (dt, *J* = 15.6, 6.7 Hz, 1H), 5.83 (d, *J* = 15.6 Hz, 1H), 5.12 (s, 1H), 4.18 (q, *J* = 7.1 Hz, 2H), 2.41 – 2.32 (m, 4H), 2.27 – 2.20 (m, 2H), 1.44 – 1.37 (m, 2H), 1.35 – 1.31 (m, 2H), 1.29 – 1.27 (m, 5H), 1.26 (s, 12H), 0.89 (t, *J* = 7.1 Hz, 3H); ^{13}**C NMR (101 MHz, Chloroform-*d*)** δ 166.8, 165.5, 148.8, 121.6, 82.8, 60.3, 37.1, 35.0, 31.8, 30.6, 29.2, 25.0, 22.6, 14.4, 14.2 ppm; Analytical data were identical to those previously reported[12]

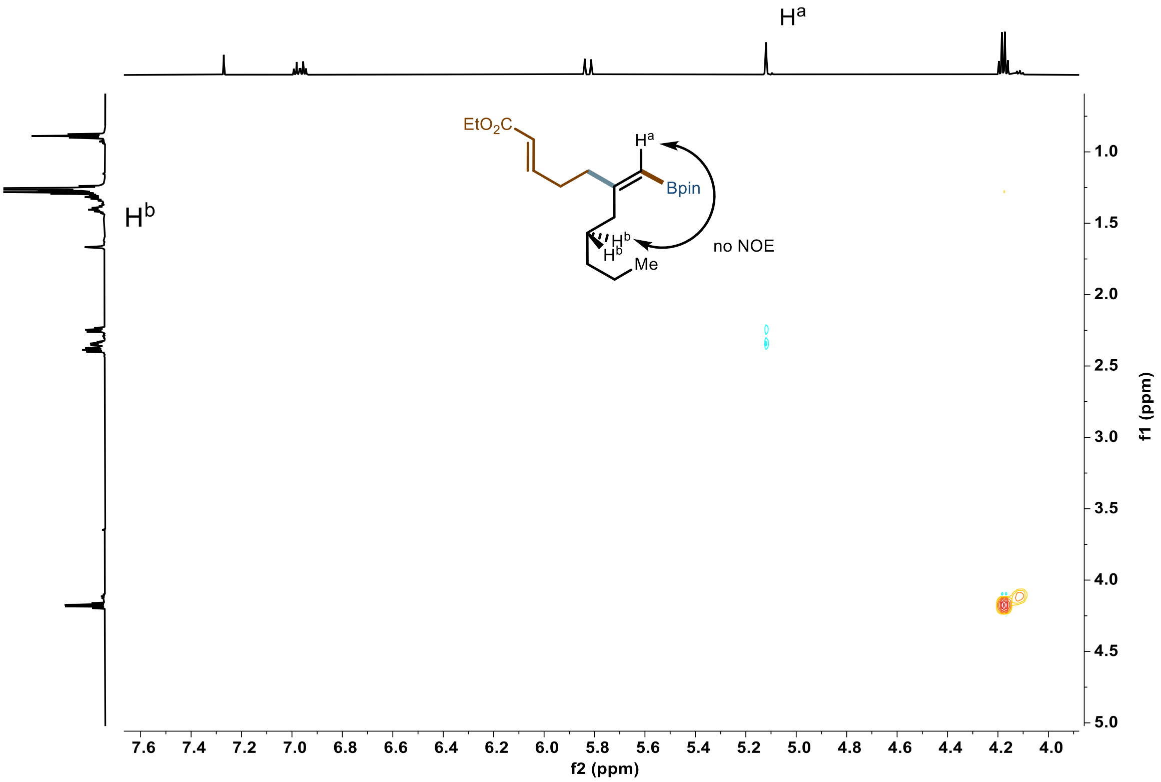

Figure S4 NOE of the compound **87**

6.7 Cyclic voltammetry experiments

Cyclic voltammetry (CV) experiments were conducted in a 20 mL three-necked cell set-up fitted with a glassy carbon working electrode (3 mm in diameter), an Ag/AgCl reference electrode, and a platinum wire counter electrode. All measurements were carried out in 5 mL anhydrous NMP with an electrolyte (nBu_4NPF_6, 0.1 mmol) and stirred for 3 min, using a scan rate of 200 mV/s. These samples were measured at room temperature and CV spectrum were shown in Figure S3-S4.

Blank:

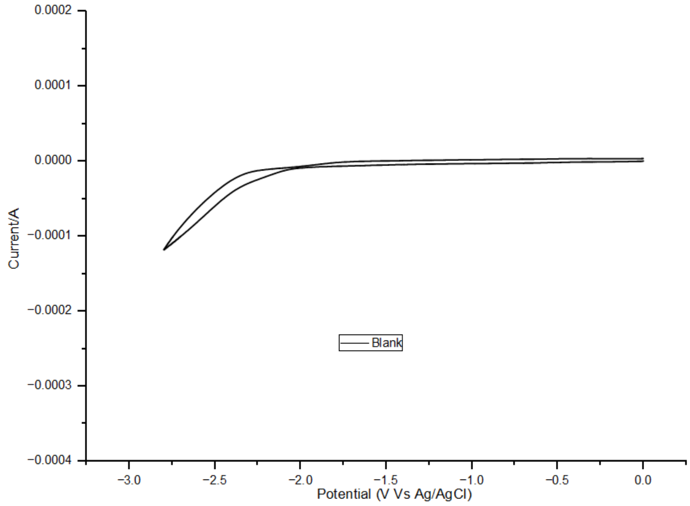

Figure S5 nBu_4NPF_6 (0.1 mmol) in 5.0 mL NMP

$NiCl_2$·DME:

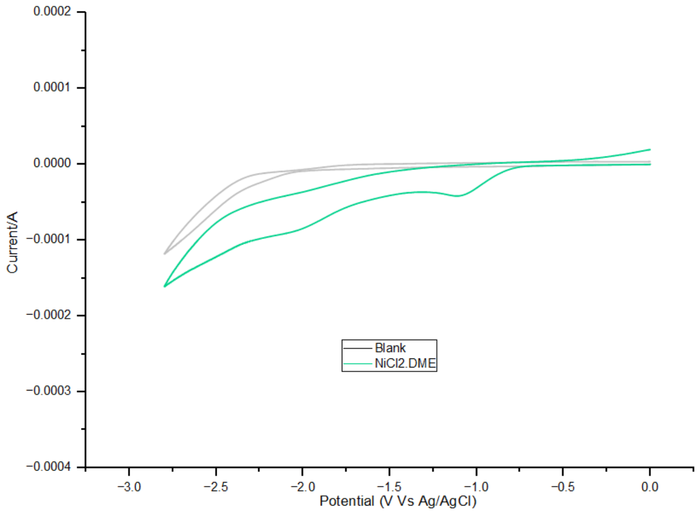

Figure S6 Blank line: nBu_4NPF_6 (0.1 mmol) in 5.0 mL NMP. Green line: $NiCl_2$·DME (0.05 mmol), nBu_4NPF_6, (0.1 mmol) in 5.0 mL NMP.

NiCl$_2$·DME+L4:

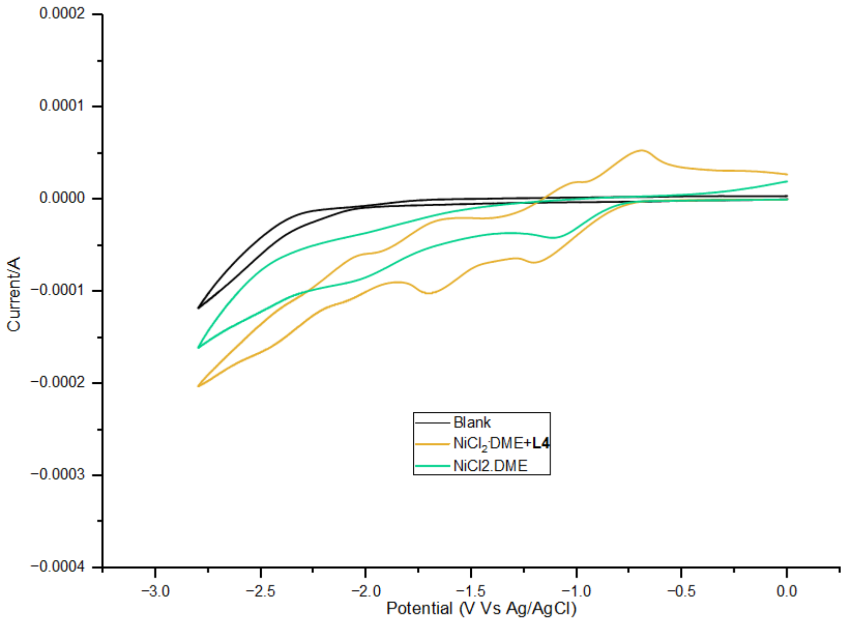

Figure S7 Blank line: nBu$_4$NPF$_6$ (0.1 mmol) in 5.0 mL NMP. Green line: NiCl$_2$·DME (0.05 mmol), nBu$_4$NPF$_6$, (0.1 mmol) in 5.0 mL NMP. Yellow line: NiCl$_2$·DME (0.05 mmol), **L4** (0.05 mmol), nBu$_4$NPF$_6$, (0.1 mmol) in 5.0 mL NMP.

NiCl$_2$·DME+L4+B$_2$pin$_2$+LiOMe:

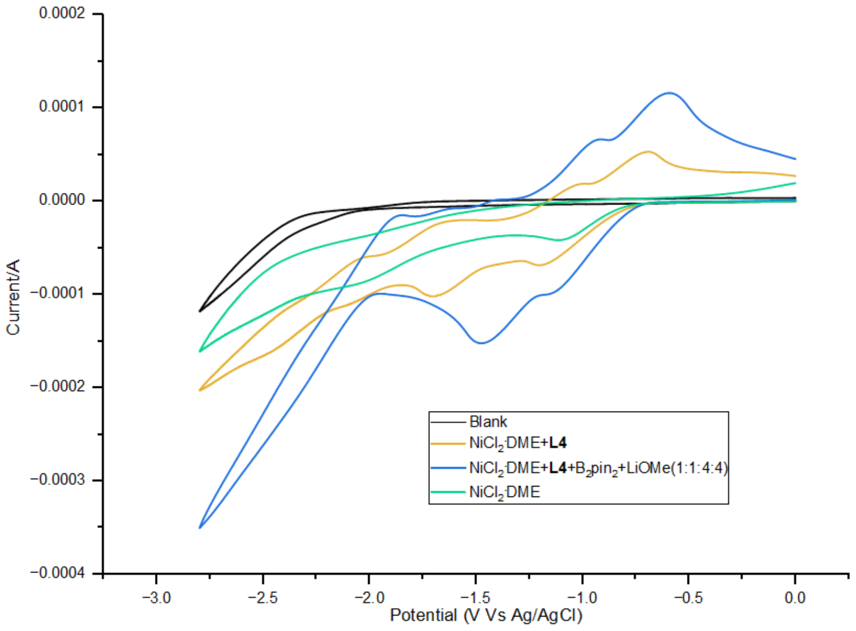

Figure S8 Blank line: nBu$_4$NPF$_6$ (0.1 mmol) in 5.0 mL NMP. Green line: NiCl$_2$·DME (0.05 mmol), nBu$_4$NPF$_6$, (0.1 mmol) in 5.0 mL NMP. Yellow line: NiCl$_2$·DME (0.05 mmol), **L4** (0.05 mmol), nBu$_4$NPF$_6$, (0.1 mmol) in 5.0 mL NMP. Blue line: NiCl$_2$·DME (0.05 mmol), **L4** (0.05 mmol), B$_2$pin$_2$ (0.2 mmol), LiOMe (0.2 mmol), nBu$_4$NPF$_6$, (0.1 mmol) in 5.0 mL NMP.

L4

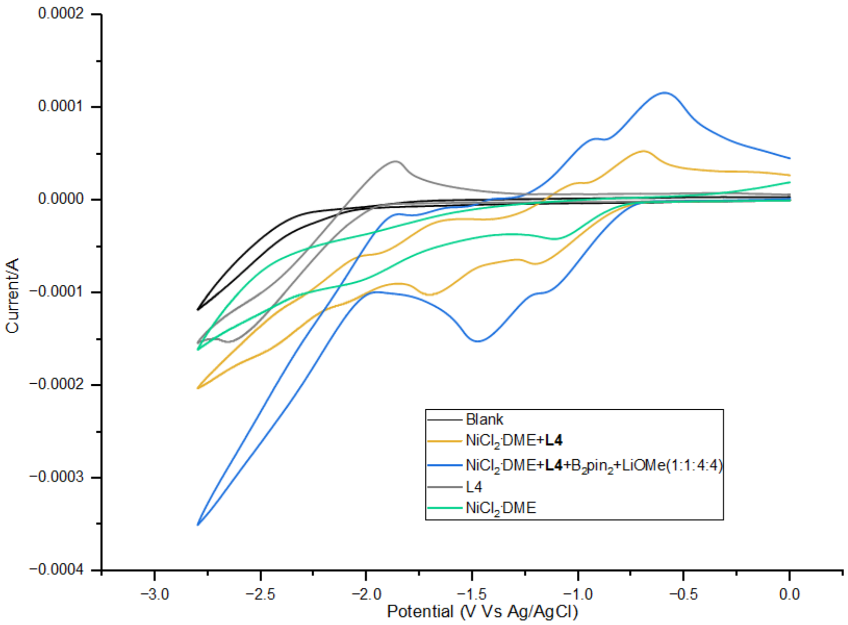

Figure S9 Blank line: nBu_4NPF_6 (0.1 mmol) in 5.0 mL NMP. Green line: $NiCl_2$·DME (0.05 mmol), nBu_4NPF_6, (0.1 mmol) in 5.0 mL NMP. Yellow line: $NiCl_2$·DME (0.05 mmol), **L4** (0.05 mmol), nBu_4NPF_6, (0.1 mmol) in 5.0 mL NMP. Blue line: $NiCl_2$·DME (0.05 mmol), **L4** (0.05 mmol), B_2pin_2 (0.2 mmol), LiOMe (0.2 mmol), nBu_4NPF_6, (0.1 mmol) in 5.0 mL NMP. Gray Line **L4** (0.05 mmol)

$NiCl_2$·DME+L8

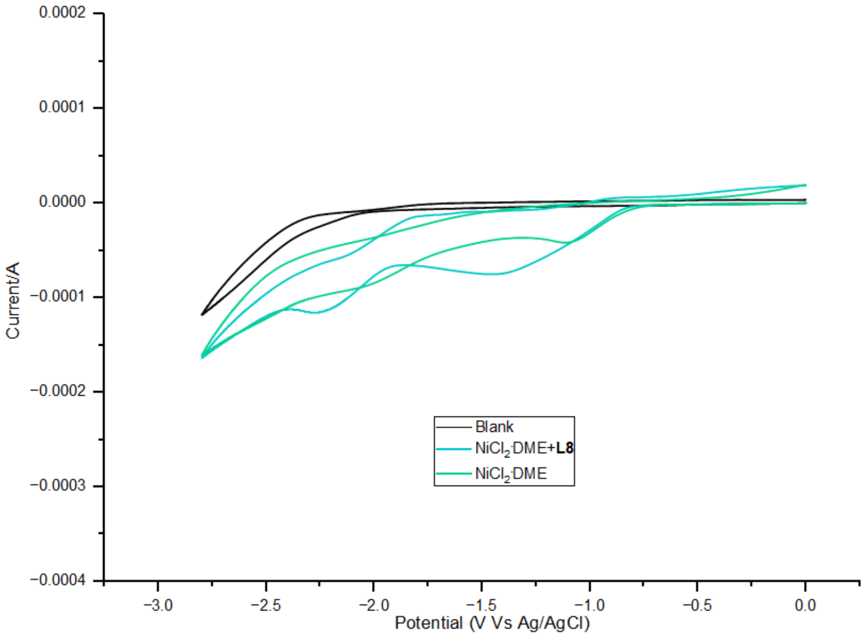

Figure S10 Blank line: nBu_4NPF_6 (0.1 mmol) in 5.0 mL NMP. Green line: $NiCl_2$·DME (0.05 mmol), nBu_4NPF_6, (0.1 mmol) in 5.0 mL NMP. Cyan line: $NiCl_2$·DME (0.05 mmol), **L8** (0.05 mmol), nBu_4NPF_6, (0.1 mmol) in 5.0 mL NMP.

$NiCl_2$·DME+L8+B_2pin_2+LiOMe:

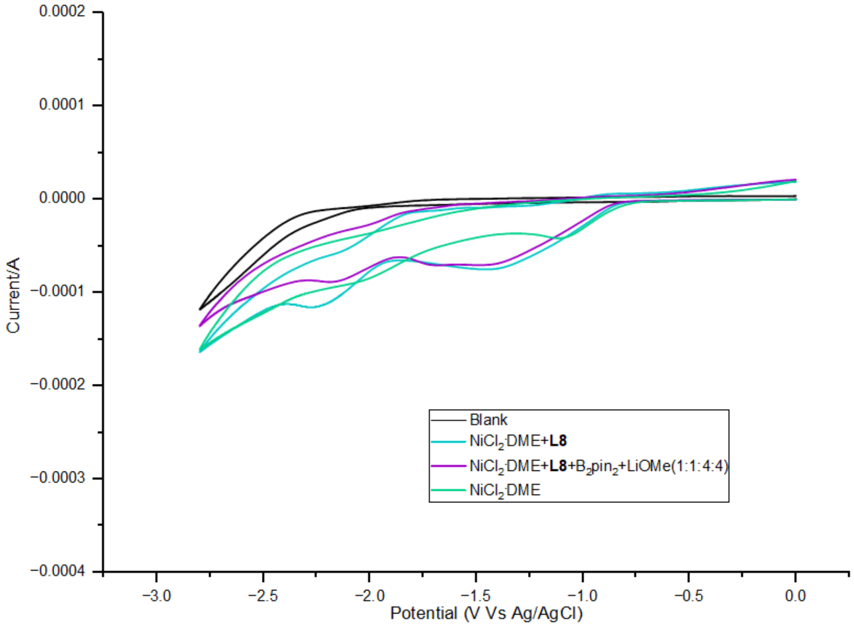

Figure S11 Blank line: nBu_4NPF_6 (0.1 mmol) in 5.0 mL NMP. Green line: $NiCl_2$·DME (0.05 mmol), nBu_4NPF_6, (0.1 mmol) in 5.0 mL NMP. Cyan line: $NiCl_2$·DME (0.05 mmol), **L8** (0.05 mmol), nBu_4NPF_6, (0.1 mmol) in 5.0 mL NMP. Purple line: $NiCl_2$·DME (0.05 mmol), **L8** (0.05 mmol), B_2pin_2 (0.2 mmol), LiOMe (0.2 mmol), nBu_4NPF_6, (0.1 mmol) in 5.0 mL NMP.

L8:

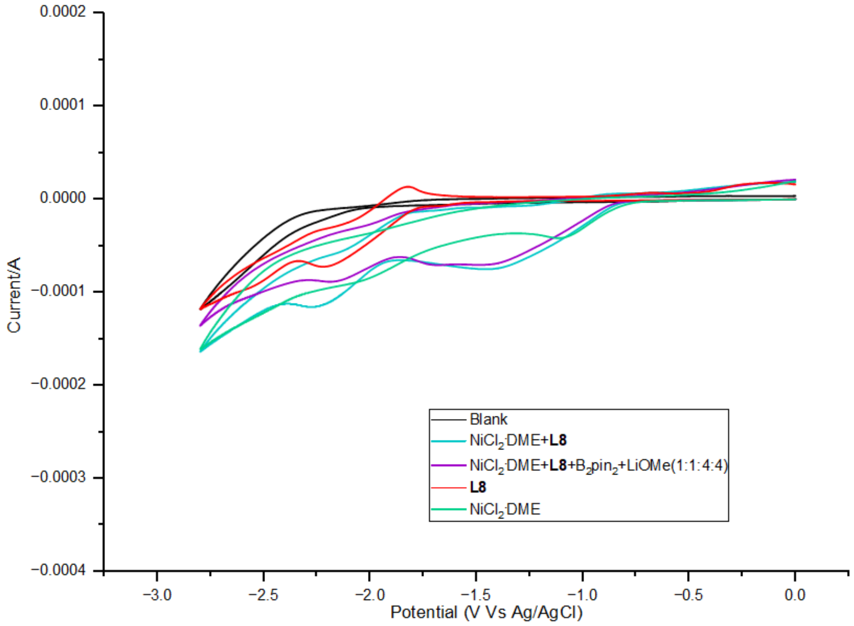

Figure S12 Blank line: nBu_4NPF_6 (0.1 mmol) in 5.0 mL NMP. Green line: $NiCl_2$·DME (0.05 mmol), nBu_4NPF_6, (0.1 mmol) in 5.0 mL NMP. Cyan line: $NiCl_2$·DME (0.05 mmol), **L8** (0.05 mmol), nBu_4NPF_6, (0.1 mmol) in 5.0 mL NMP. Purple line: $NiCl_2$·DME (0.05 mmol), **L8** (0.05 mmol), B_2pin_2 (0.2 mmol), LiOMe (0.2 mmol), nBu_4NPF_6, (0.1 mmol) in 5.0 mL NMP. Red line: **L8** (0.05 mmol).

Summary (Reduction potential):

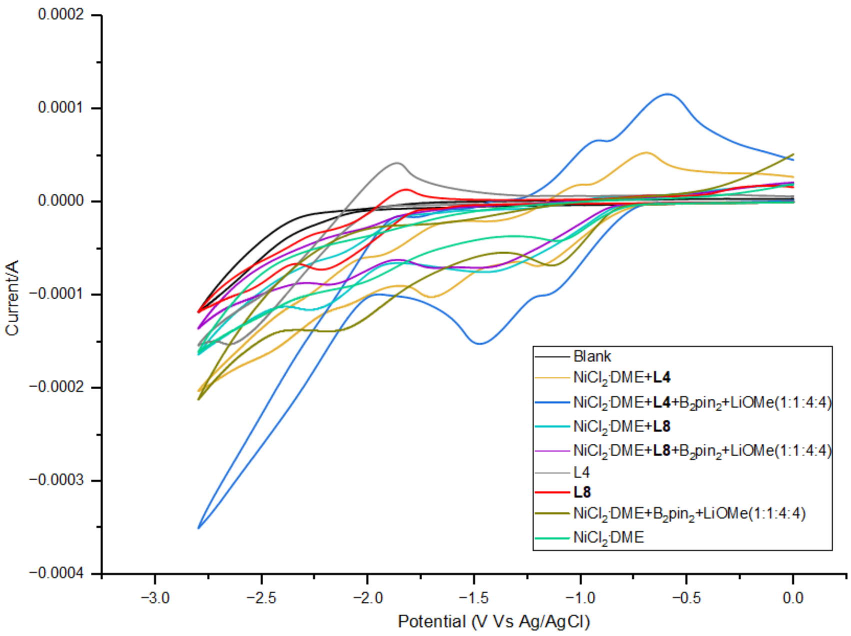

Figure S13 Blank line: nBu_4NPF_6 (0.1 mmol) in 5.0 mL NMP. Green line: $NiCl_2$·DME (0.05 mmol), nBu_4NPF_6, (0.1 mmol) in 5.0 mL NMP. Gray Line **L4** (0.05 mmol). Red line: **L8** (0.05 mmol). Cyan line: $NiCl_2$·DME (0.05 mmol), **L8** (0.05 mmol), nBu_4NPF_6, (0.1 mmol) in 5.0 mL NMP. Yellow line: $NiCl_2$·DME (0.05 mmol), **L4** (0.05 mmol), nBu_4NPF_6, (0.1 mmol) in 5.0 mL NMP. Purple line: $NiCl_2$·DME (0.05 mmol), **L8** (0.05 mmol), B_2pin_2 (0.2 mmol), LiOMe (0.2 mmol), nBu_4NPF_6, (0.1 mmol) in 5.0 mL NMP. Blue line: $NiCl_2$·DME (0.05 mmol), **L4** (0.05 mmol), B_2pin_2 (0.2 mmol), LiOMe (0.2 mmol), nBu_4NPF_6, (0.1 mmol) in 5.0 mL NMP. Deep yellow: $NiCl_2$·DME (0.05 mmol), B_2pin_2 (0.2 mmol), LiOMe (0.2 mmol), nBu_4NPF_6, (0.1 mmol) in 5.0 mL NMP.

7. Computational analysis

Computational Methods: All of the density functional theory (DFT) calculations were performed with the Gaussian 16[13] series of programs. The B3LYP functional[14,15] including Grimme empirical dispersion correction (GD3BJ)[16] with the standard def2-SVP basis set[17] was used for the geometry optimizations in the gas phase. Harmonic vibrational frequency calculations were performed for all of the stationary points to determine whether they are local minima or transition structures and to derive the thermochemical corrections for free energies. Intrinsic reaction coordinate (IRC) calculations were performed to ensure that the saddle points located were transition states connecting the reactants and the products. The M06 functional[18] proposed by Truhlar et al. with the def2-TZVP basis set[19] was used to calculate the single-point energies. The solvent effect was considered by applying the implicit SMD[20] solvation model with 1,4-dioxane as solvent in single-point energy calculations. The 3D images of the calculated structures were prepared using CYLview[21]. The surface distance projection maps were created using Multiwfn[22]. The distortion/interaction analysis[23,24] was performed at the same level of single-point energies. All conformations of each structure were carefully explored by the assistance of xtb-6.5.1[25], Molclus (version 1.12)[26]. The structures with the lowest energy were applied.

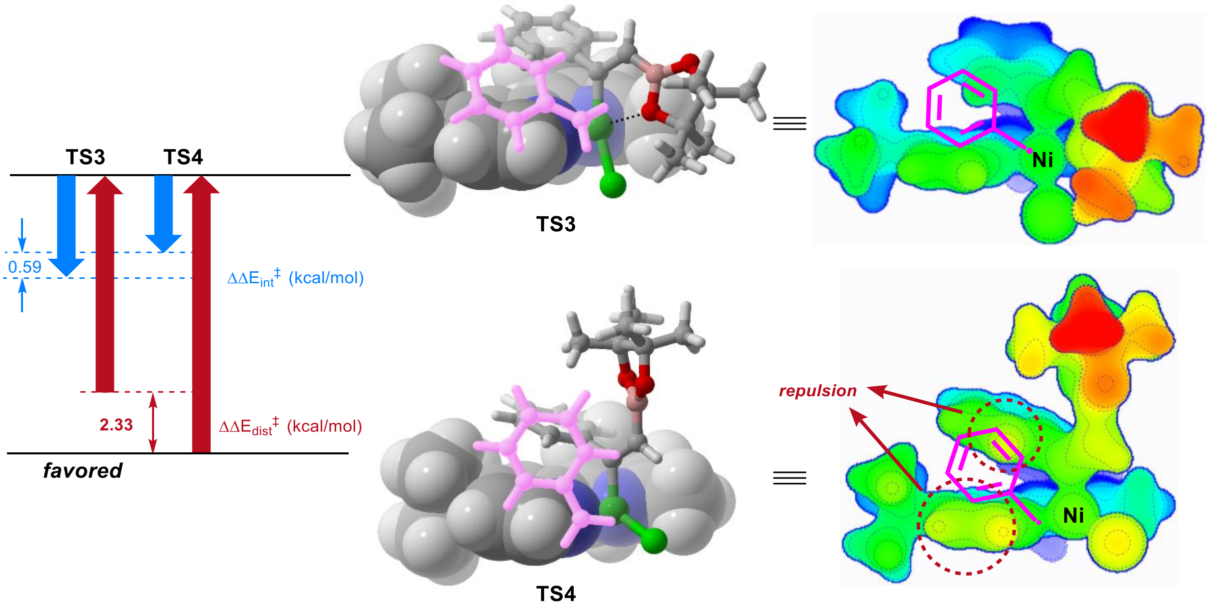

Figure S14 Distortion/Interaction energy analysis and steric environment of benzyl radical addition in **L4**. **TS3** is preferred in both distortion energy and interaction energy. The difference of distortion energy is more dominant, which can be further implied from surface distance projection map that benzyl radical in **TS3** orients in the minimal repulsion site. In contrast, no matter in which direction, the benzyl radical in **TS4** inevitably repels with the aryl rings from the ligand as well as the alkenyl group.

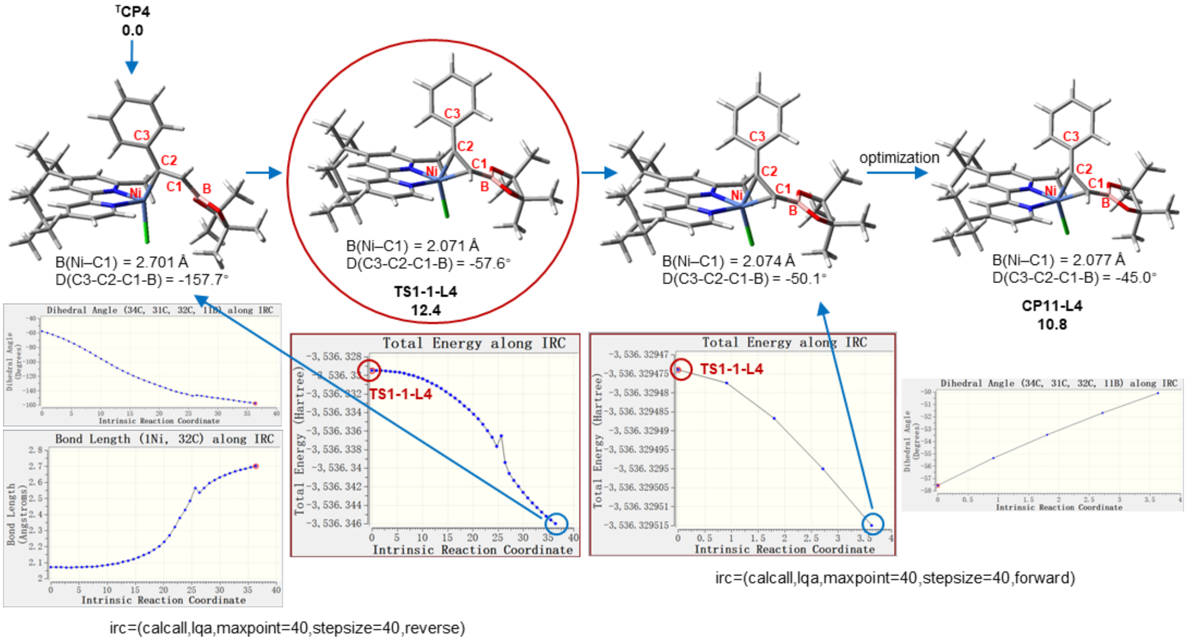

Figure S15 Intrinsic reaction coordinate study of the syn/anti-isomerization step in **L4**. The E/Z-isomerization from **^{T}CP4** to **^{T}CP3** is a stepwise process, which undergoes the coordination of C1 and the flip of phenyl group first (**TS1-1-L4**) to construct a three-membered η²-vinylnickel intermediate **CP11-L4**. Then the Ni-C1 bond dissociates through **TS1-L4** and forms **^{T}CP3**.

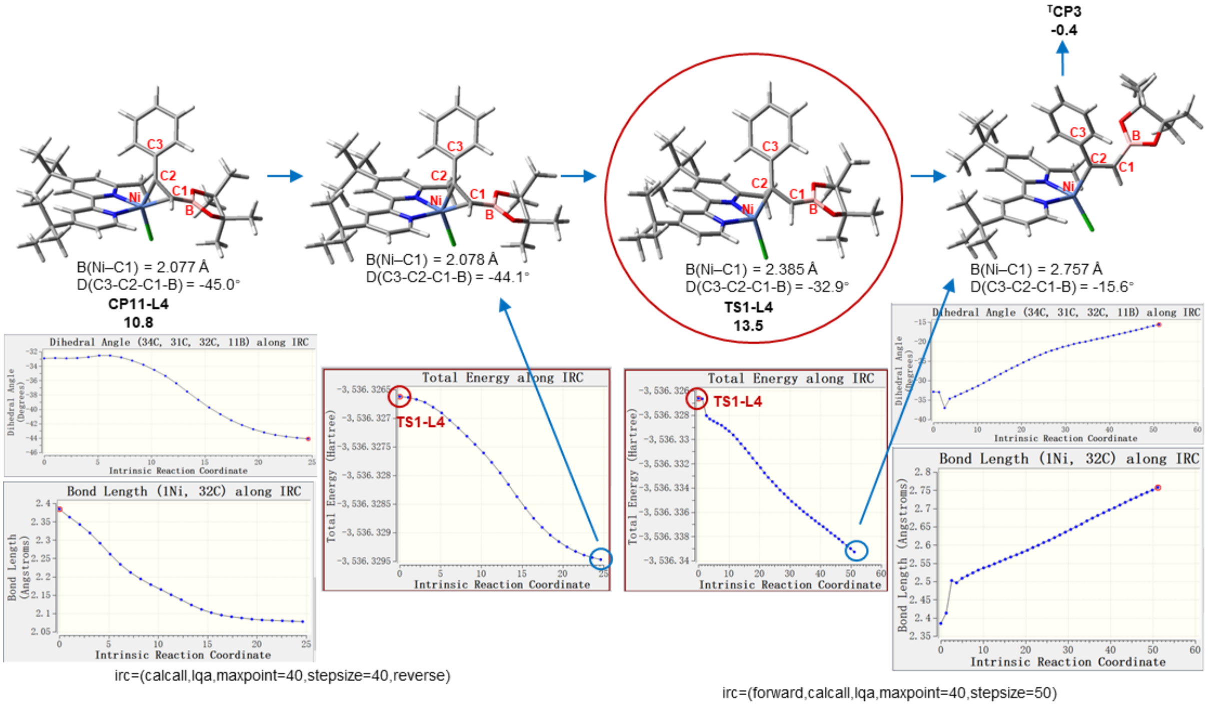

Figure S16 Intrinsic reaction coordinate study of the syn/anti-isomerization step in **L4**. After the generation of **CP11-L4**, the Ni-C1 bond dissociates through **TS1-L4** to form **^{T}CP3**.

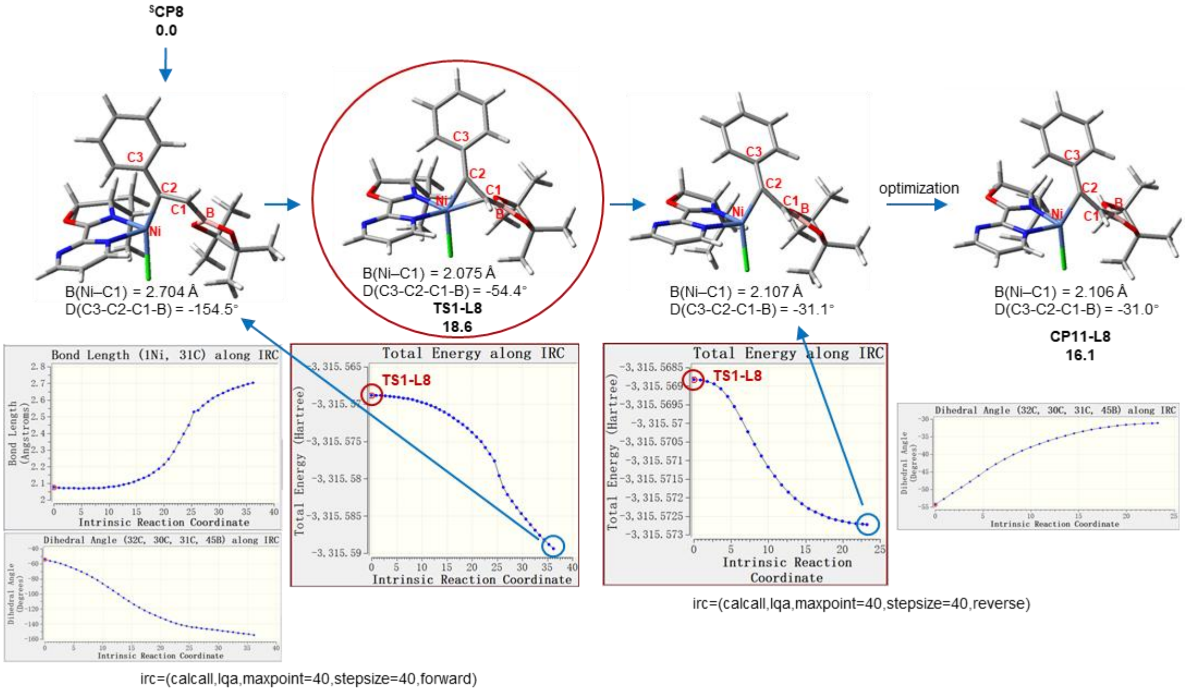

Figure S17 Intrinsic reaction coordinate study of the syn/anti -isomerization step in **L8**. Similar to **L4**, the E/Z-isomerization from **^{S}CP8** to **^{S}CP7** is also a stepwise process. It should be noticed that according to our DFT calculation, high spin pathways are much more favored in syn/anti -isomerization in both **L4** and **L8**. So the singlet *syn*-intermediate **^{S}CP8** will initially experiences a spin state transition, then undergoes the coordination of C1 and the flip of phenyl group first (**TS1-L8**). After constructing the three-membered η²-vinylnickel intermediate **CP11-L8**, the Ni-C1 bond dissociates through **TS1-1-L8** to form **^{S}CP7**.

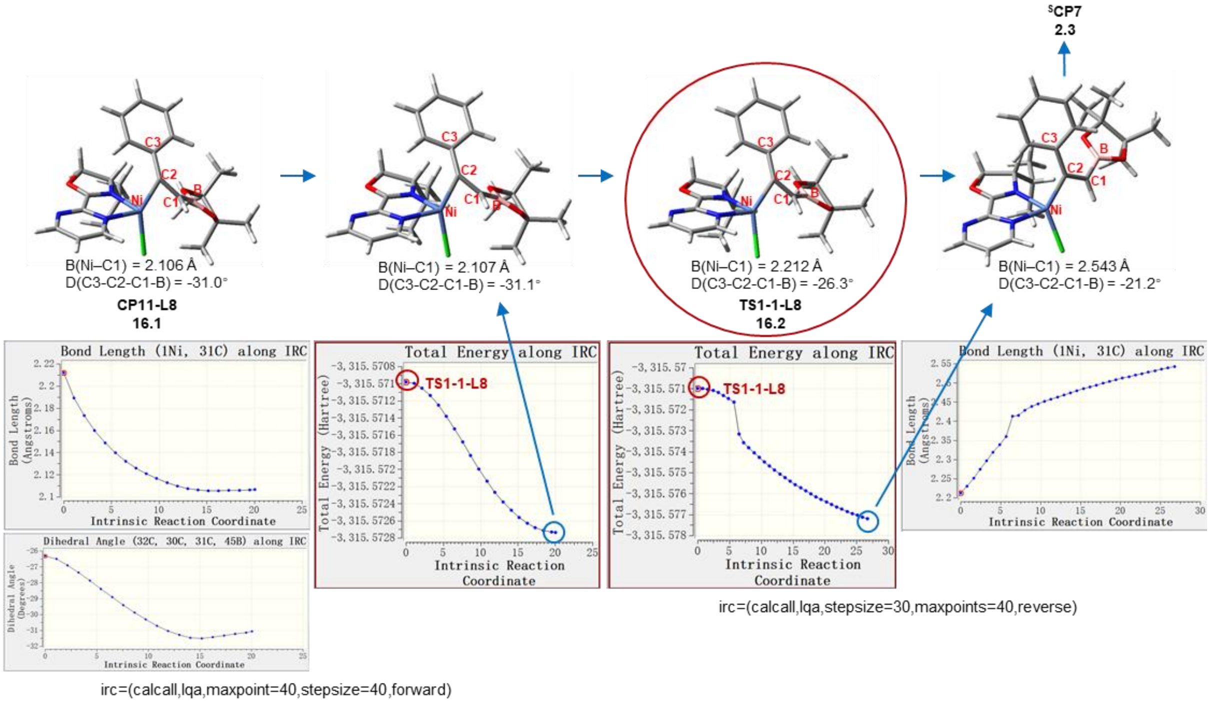

Figure S18 Intrinsic reaction coordinate study of the syn/anti-isomerization step in **L8**. After constructing the three-membered η^2-vinylnickel intermediate **CP11-L8**, the Ni-C1 bond dissociates through **TS1-1-L8** to form $^{\mathbf{S}}$**CP7**. It should be noticed that both **CP11-L8** and **TS1-1-L8** are also triplet.

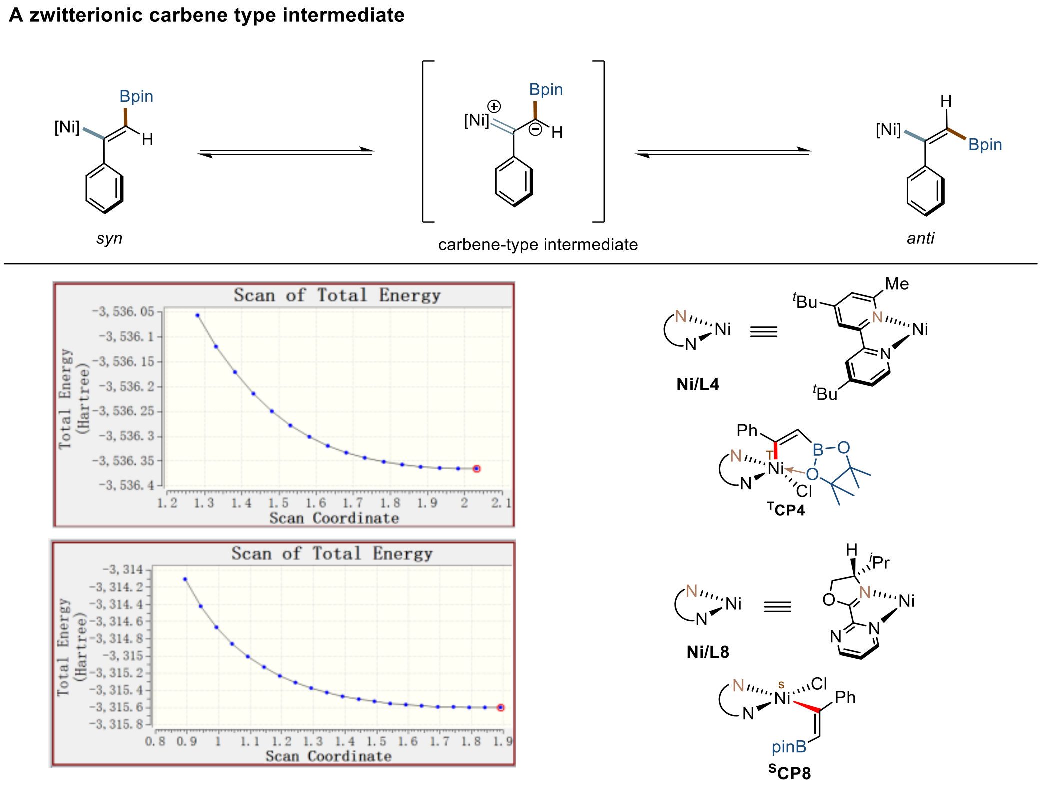

Figure S19 Exploration for metal-carbene species in syn/anti-isomerization process. Sicne metal-carbene mechanism of E/Z-isomerization has been proposed in many previous work, we tried to located the key metal-carbene species by scanning the Ni-C bond in both $^{\mathbf{T}}$**CP4** and $^{\mathbf{S}}$**CP8**. The total energy of nickel complex rises monotonically when the Ni-C bond becomes shorter, and no stable intermediate is found. So we exclude this possibility.

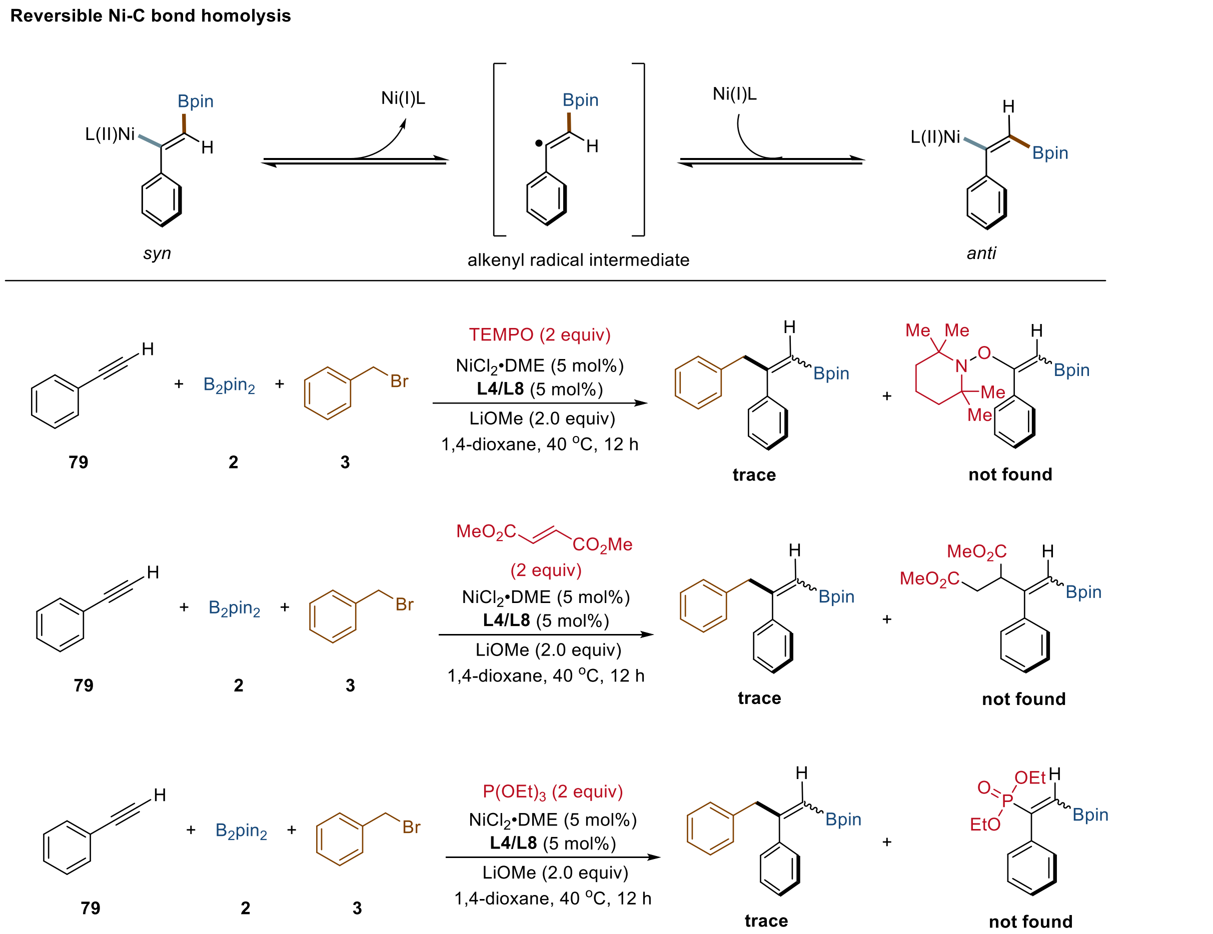

Figure S20 The radical inhibition experiment did not detect the alkenyl radical intermediate by high-resolution mass spectrometry (HRMS). This result does not support the reversible Ni - C homolysis pathway.

Table S1 Absolute Calculation Energies, Enthalpies, and Free Energies

Geometry	Imaginary freq	$E_{(elec\text{-}B3LYP\text{-}D3)}$[1]	$E_{(solv,\ M06)}$[2]	$G_{(corr\text{-}B3LYP\text{-}D3)}$[3]
**CP1-L4**		-2817.050859	-2817.467018	0.354665
**CP1-L4+BnBr**		-5661.745818	-5662.383616	0.461648
**TS2-L4**	-220.10	-5661.741058	-5662.370000	0.463086
**CP2-L4**		-5390.991403	-5391.581224	0.352305
**CP1-L8**		-2596.293723	-2596.751481	0.179773
**CP1-L8+BnBr**		-5440.990470	-5441.667764	0.287153
**TS2-L8**	-216.00	-5440.984130	-5441.652519	0.288436
**CP2-L8**		-5170.229481	-5170.863739	0.178003
**BnBr**		-2844.669589	-2844.910583	0.085400
**Bn radical**		-270.746467	-270.789209	0.085035
**^{T}CP3**		-3536.352212	-3536.936992	0.626344
**^{S}CP3**		-3536.342335	-3536.935260	0.630181

**TS3**	-23.52	-3807.124519	-3807.740273	0.738592
**TS3-1**	-13.18	-3807.108962	-3807.731604	0.733835
**^{T}CP4**		-3536.371692	-3536.944236	0.634248
**^{S}CP4**		-3536.358513	-3536.942305	0.633515
**TS4**	-20.71	-3807.133696	-3807.744923	0.740597
**TS4-1**	-16.01	-3807.123591	-3807.730561	0.739598
**CP5**		-3807.148229	-3807.765834	0.741990
**TS5**	-283.18	-3807.133729	-3807.753660	0.743435
**CP6**		-3807.147308	-3807.761329	0.744797
**TS6**	-323.24	-3807.133315	-3807.751040	0.744824
**^{S}CP7**		-3315.588977	-3316.231584	0.453905
**^{T}CP7**		-3315.587059	-3316.217900	0.449742
**TS7**	-26.20	-3586.366700	-3587.036271	0.562907
**TS7-1**	-30.74	-3586.358673	-3587.021431	0.560763
**^{S}CP8**		-3315.603743	-3316.239182	0.457806
**^{T}CP8**		-3315.610119	-3316.229991	0.456920
**TS8**	-31.66	-3586.376609	-3587.039370	0.566107
**TS8-1**	-39.67	-3586.371768	-3587.025549	0.563408
**CP9**		-3586.384668	-3587.049948	0.565113
**TS9**	-276.02	-3586.373211	-3587.041765	0.564405
**CP10**		-3586.384517	-3587.041515	0.567213
**TS10**	-288.42	-3586.377200	-3587.038518	0.568221
***syn*-product**		-990.098699	-990.339043	0.362585
***anti*-product**		-990.095103	-990.337237	0.360249
**TS1-L4**	-25.59	-3536.326624	-3536.916752	0.628275
**TS1-1-L4**	-16.18	-3536.329474	-3536.918688	0.628540
**CP11-L4**		-3536.329548	-3536.919155	0.626436
**TS1-L8**	-25.02	-3315.568828	-3316.204713	0.452963
**TS1-1-L8**	-31.02	-3315.570979	-3316.209541	0.453988
**CP11-L8**		-3315.572714	-3316.209573	0.453785

[1]The electronic energy (in Hartree) calculated by B3LYP-D3(BJ)/def2-SVP in gas phase. [2]The electronic energy (in Hartree) calculated by M06/def2-TZVP in 1,4-dioxane. [3]The thermal correction to Gibbs free energy (in Hartree) calculated by B3LYP-D3(BJ)/def2-SVP in gas phase.

8. X-ray characterization data

Crystals suitable for X-ray single-crystal diffraction analysis were obtained from Hexane/$CHCl_3$ using slow evaporation under air at room temperature.

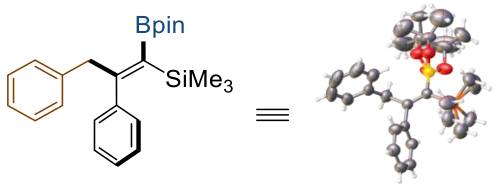

24 *CCDC: 2320520*

Table S2 Crystal data and structure refinement for **24**.

Identification code	**24**
Empirical formula	C24H33BO2Si
Formula weight	392.40
Temperature/K	295(2)
Crystal system	monoclinic
Space group	I2/a
a/Å	25.3212(15)
b/Å	6.3279(5)
c/Å	29.8444(16)
α/°	90
β/°	94.871(5)
γ/°	90
Volume/Å^3	4764.7(5)
Z	8
ρ_{calc}g/cm^3	1.094
μ/mm^{-1}	0.974
F(000)	1696.0
Crystal size/mm^3	0.12 × 0.06 × 0.04
Radiation	CuKα (λ = 1.54184)
2Θ range for data collection/°	5.944 to 140.116
Index ranges	-30 ≤ h ≤ 30, -6 ≤ k ≤ 7, -34 ≤ l ≤ 36
Reflections collected	16498
Independent reflections	4484 [R_{int} = 0.0478, R_{sigma} = 0.0442]
Data/restraints/parameters	4484/122/366
Goodness-of-fit on F^2	1.043
Final R indexes [I>=2σ (I)]	R_1 = 0.0590, wR_2 = 0.1590
Final R indexes [all data]	R_1 = 0.0833, wR_2 = 0.1824
Largest diff. peak/hole / e Å^{-3}	0.19/-0.39

Crystals suitable for X-ray single-crystal diffraction analysis were obtained from Hexane/EA using slow evaporation under air at room temperature.

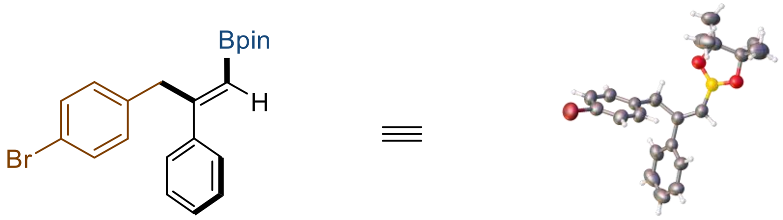

28 *CCDC: 2261903*

Table S3 Crystal data and structure refinement for **28**.

Identification code	**28**
Empirical formula	C21H24BBrO2
Formula weight	399.12
Temperature/K	298.74(10)
Crystal system	monoclinic
Space group	P21/c
a/Å	6.0056(3)
b/Å	15.9159(11)
c/Å	20.4438(10)
α/°	90
β/°	91.539(5)
γ/°	90
Volume/$Å^3$	1953.4(2)
Z	4
ρcalcg/cm^3	1.357
μ/mm^{-1}	2.939
F(000)	824.0
Crystal size/mm^3	0.25 × 0.04 × 0.04
Radiation	Cu Kα (λ = 1.54184)
2Θ range for data collection/°	7.04 to 151.942
Index ranges	-4 ≤ h ≤ 7, -19 ≤ k ≤ 19, -25 ≤ l ≤ 24
Reflections collected	14460
Independent reflections	3850 [Rint = 0.0289, Rsigma = 0.0279]
Data/restraints/parameters	3850/0/231
Goodness-of-fit on F^2	1.120
Final R indexes [I>=2σ (I)]	R1 = 0.0505, wR2 = 0.1340
Final R indexes [all data]	R1 = 0.0769, wR2 = 0.1700
Largest diff. peak/hole / e $Å^{-3}$	0.41/-0.66

Crystals suitable for X-ray single-crystal diffraction analysis were obtained from Hexane/EA using slow evaporation under air at room temperature.

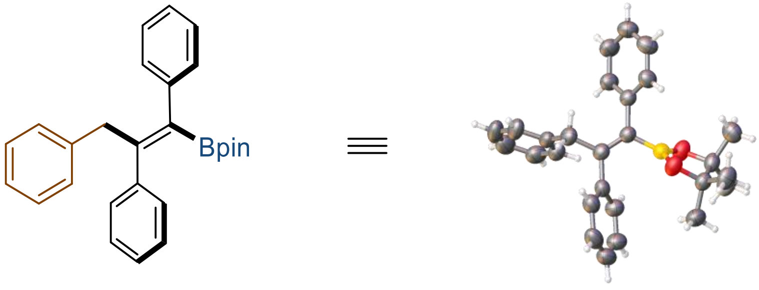

52

CCDC: 2279115

Table S4 Crystal data and structure refinement for **52**.

Identification code	**52**
Empirical formula	C27H29BO2
Formula weight	396.31
Temperature/K	299.85(10)
Crystal system	monoclinic
Space group	P21
a/Å	6.2130(5)
b/Å	19.8565(12)
c/Å	9.4147(7)
α/°	90
β/°	97.115(8)
γ/°	90
Volume/Å^3	1152.53(15)
Z	2
ρcalcg/cm^3	1.142
μ/mm^{-1}	0.537
F(000)	424.0
Crystal size/mm^3	0.12 × 0.04 × 0.04
Radiation	Cu Kα (λ = 1.54184)
2Θ range for data collection/°	8.906 to 151.042
Index ranges	$-7 \leq h \leq 4$, $-23 \leq k \leq 24$, $-11 \leq l \leq 11$
Reflections collected	8117
Independent reflections	3599 [Rint = 0.0414, Rsigma = 0.0555]
Data/restraints/parameters	3599/1/276
Goodness-of-fit on F^2	0.980
Final R indexes [I>=2σ (I)]	R1 = 0.0525, wR2 = 0.1444
Final R indexes [all data]	R1 = 0.0721, wR2 = 0.1592
Largest diff. peak/hole / e Å^{-3}	0.17/-0.20

Crystals suitable for X-ray single-crystal diffraction analysis were obtained from Hexane/$CHCl_3$ using slow evaporation under air at room temperature.

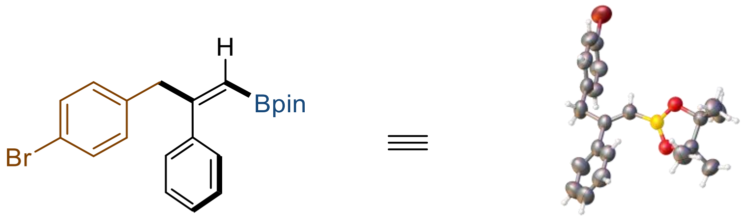

58 CCDC: 2248596

Table S5 Crystal data and structure refinement for **58**

Identification code	**58**
Empirical formula	C21H24BBrO2
Formula weight	399.12
Temperature/K	287(1)
Crystal system	monoclinic
Space group	P2/c
a/Å	13.4468(6)
b/Å	6.1100(3)
c/Å	25.0384(11)
α/°	90
β/°	104.918(4)
γ/°	90
Volume/Å^3	1987.82(16)
Z	4
ρcalcg/cm^3	1.334
μ/mm^{-1}	2.888
F(000)	824.0
Crystal size/mm^3	0.28 × 0.15 × 0.08
Radiation	Cu Kα (λ = 1.54184)
2Θ range for data collection/°	6.802 to 150.808
Index ranges	$-16 \le h \le 15$, $-5 \le k \le 7$, $-31 \le l \le 30$
Reflections collected	14341
Independent reflections	3934 [Rint = 0.0344, Rsigma = 0.0217]
Data/restraints/parameters	3934/0/230
Goodness-of-fit on F^2	1.095
Final R indexes [I>=2σ (I)]	R1 = 0.0597, wR2 = 0.1721
Final R indexes [all data]	R1 = 0.0715, wR2 = 0.1807
Largest diff. peak/hole / e Å^{-3}	0.67/-0.6

9. NMR spectra

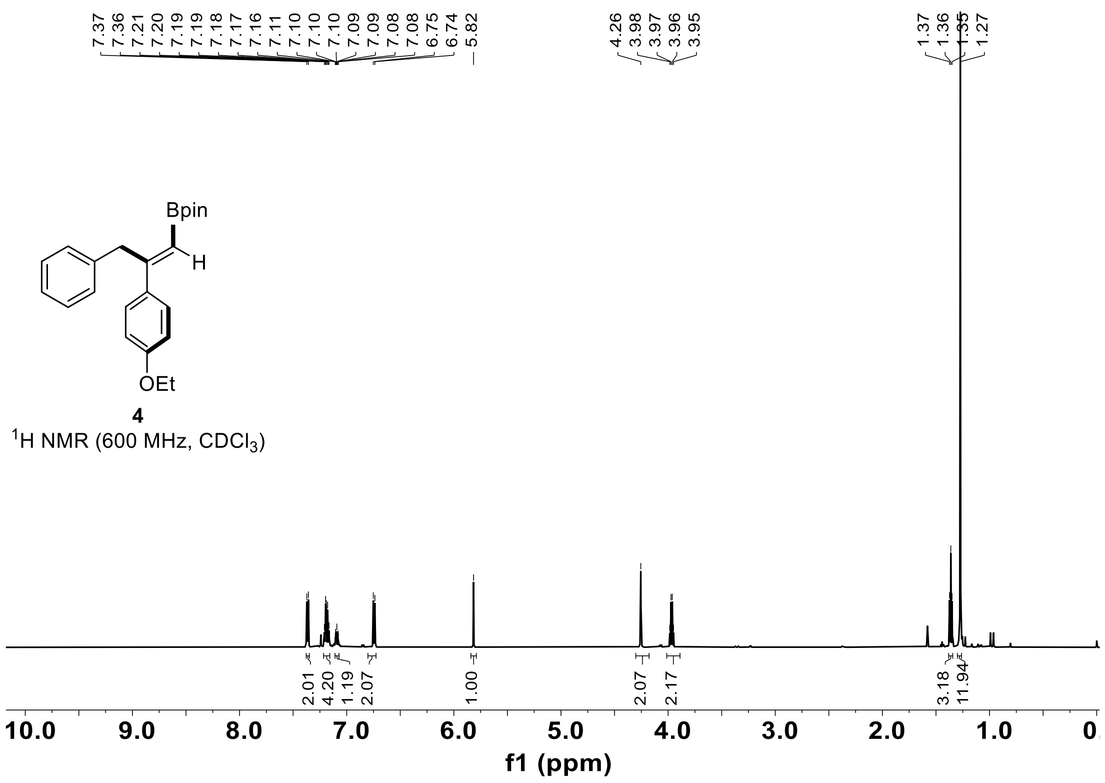

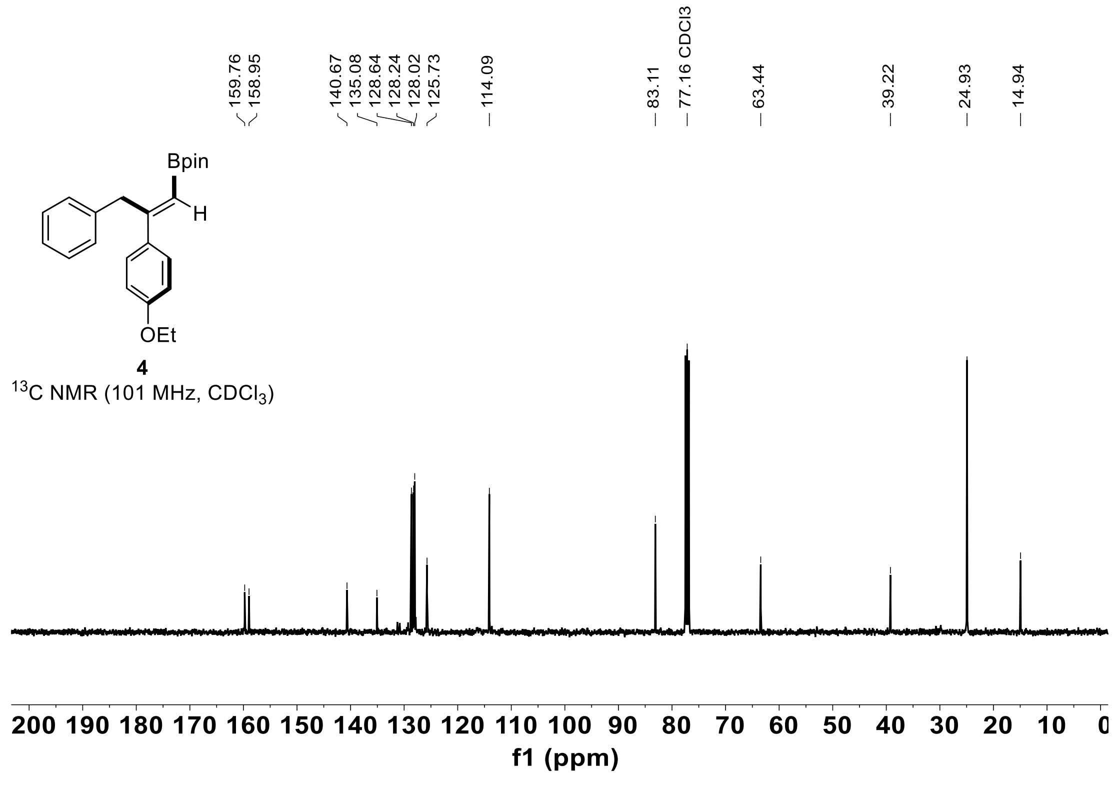

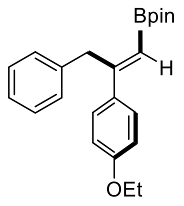

4
^{11}B NMR (193 MHz, $CDCl_3$)

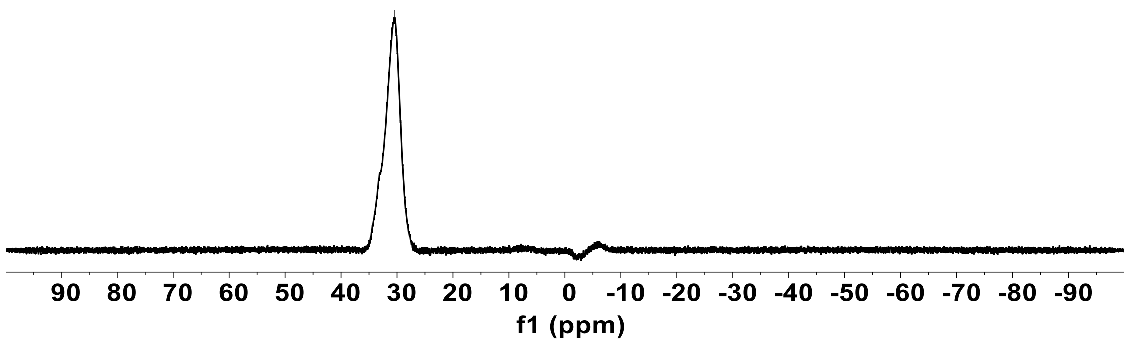

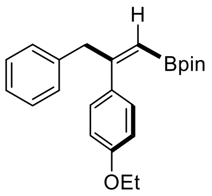

5
^{1}H NMR (600 MHz, $CDCl_3$)

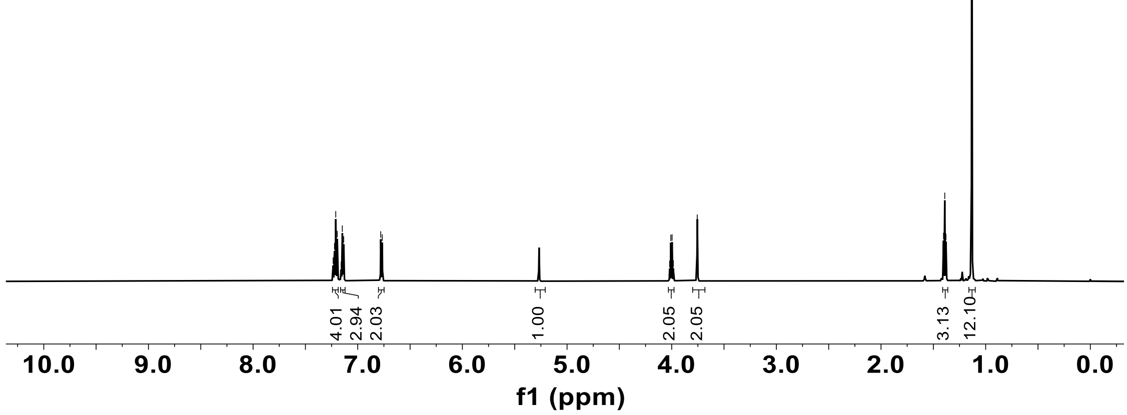

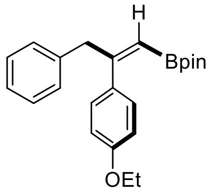

5

^{13}C NMR (151 MHz, $CDCl_3$)

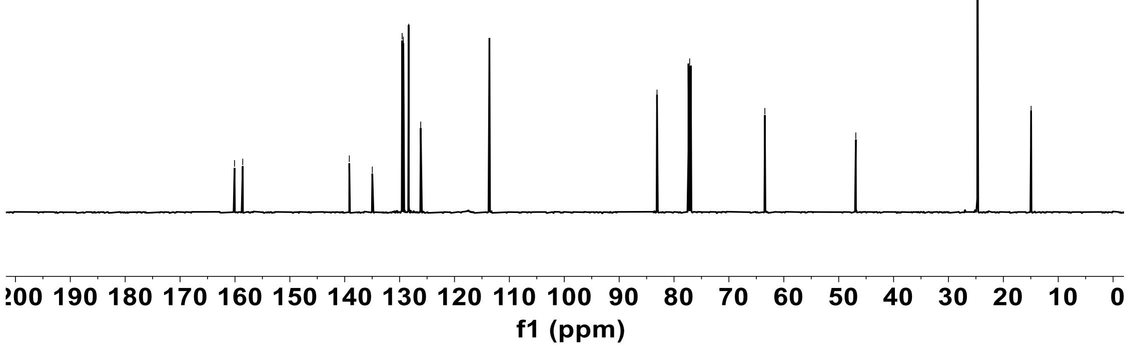

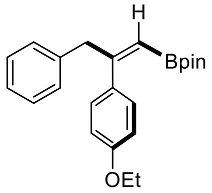

5

^{11}B NMR (193 MHz, $CDCl_3$)

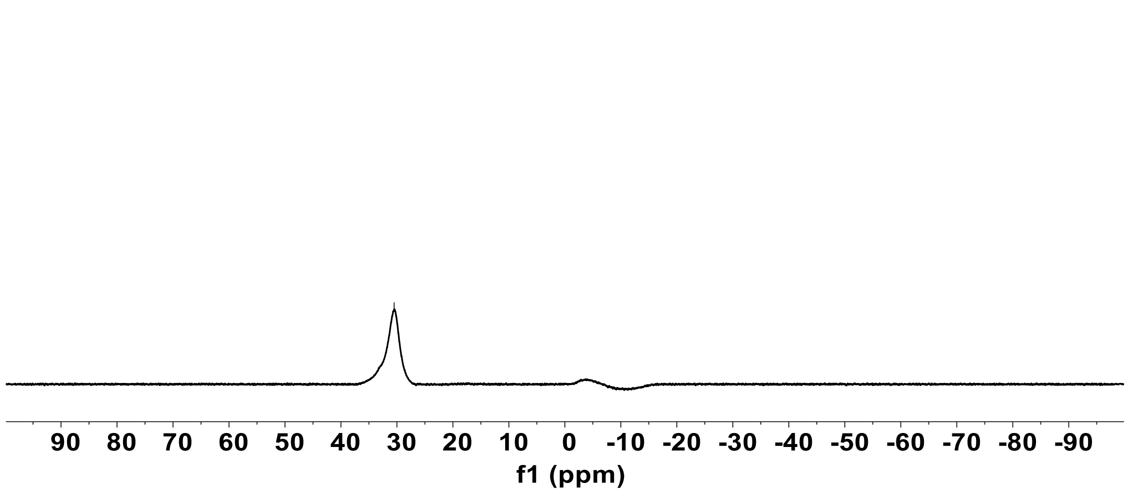

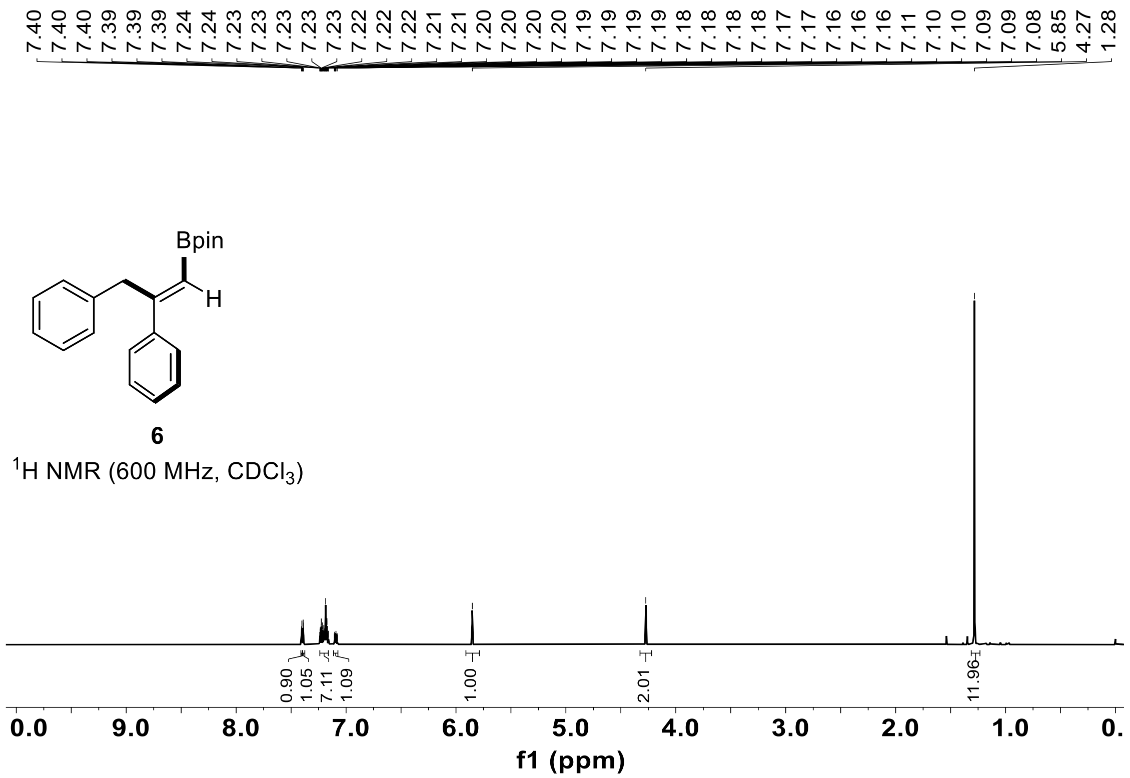

6

^{1}H NMR (600 MHz, $CDCl_3$)

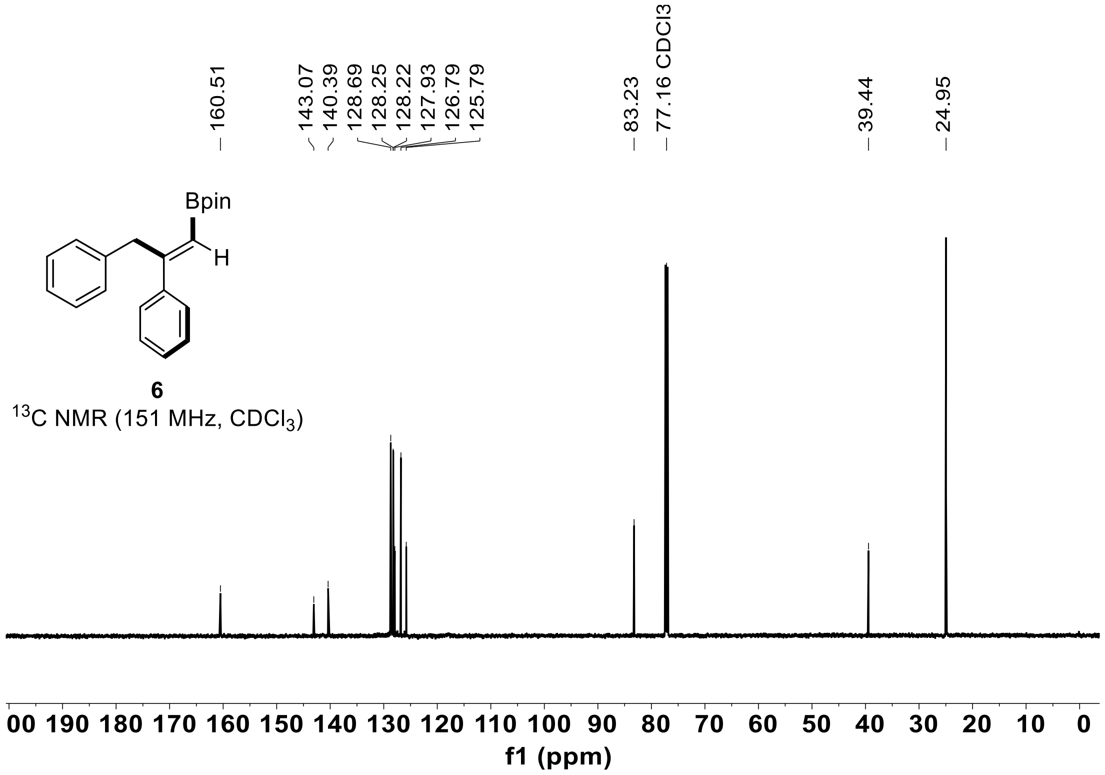

6

^{13}C NMR (151 MHz, $CDCl_3$)

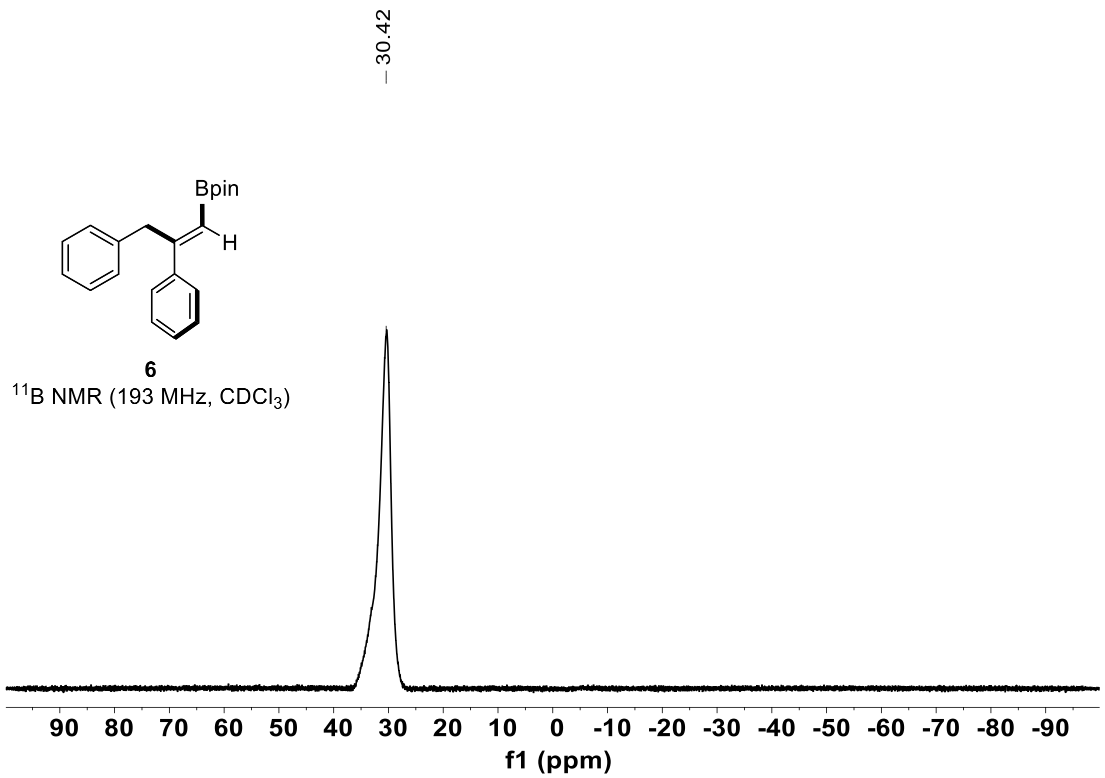

30.42
Bpin
H
6
^{11}B NMR (193 MHz, CDCl$_3$)
90 80 70 60 50 40 30 20 10 0 -10 -20 -30 -40 -50 -60 -70 -80 -90
f1 (ppm)

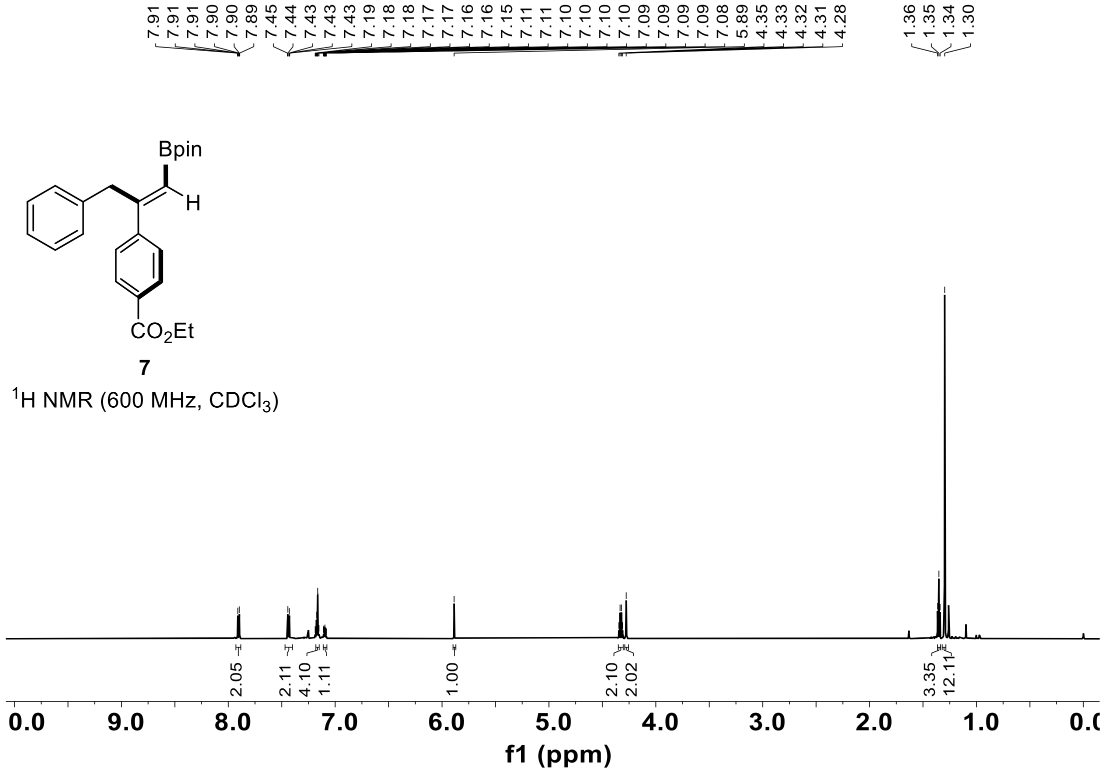

7.91 7.91 7.91 7.90 7.90 7.89 7.45 7.44 7.43 7.43 7.43 7.19 7.18 7.18 7.17 7.17 7.16 7.16 7.15 7.11 7.11 7.10 7.10 7.10 7.10 7.09 7.09 7.09 7.09 7.08 5.89 4.35 4.33 4.32 4.31 4.28
1.36 1.35 1.34 1.30
Bpin
H
CO$_2$Et
7
^{1}H NMR (600 MHz, CDCl$_3$)
2.05 2.11 4.10 1.11 1.00 2.10 2.02 3.35 12.11
0.0 9.0 8.0 7.0 6.0 5.0 4.0 3.0 2.0 1.0 0.0
f1 (ppm)

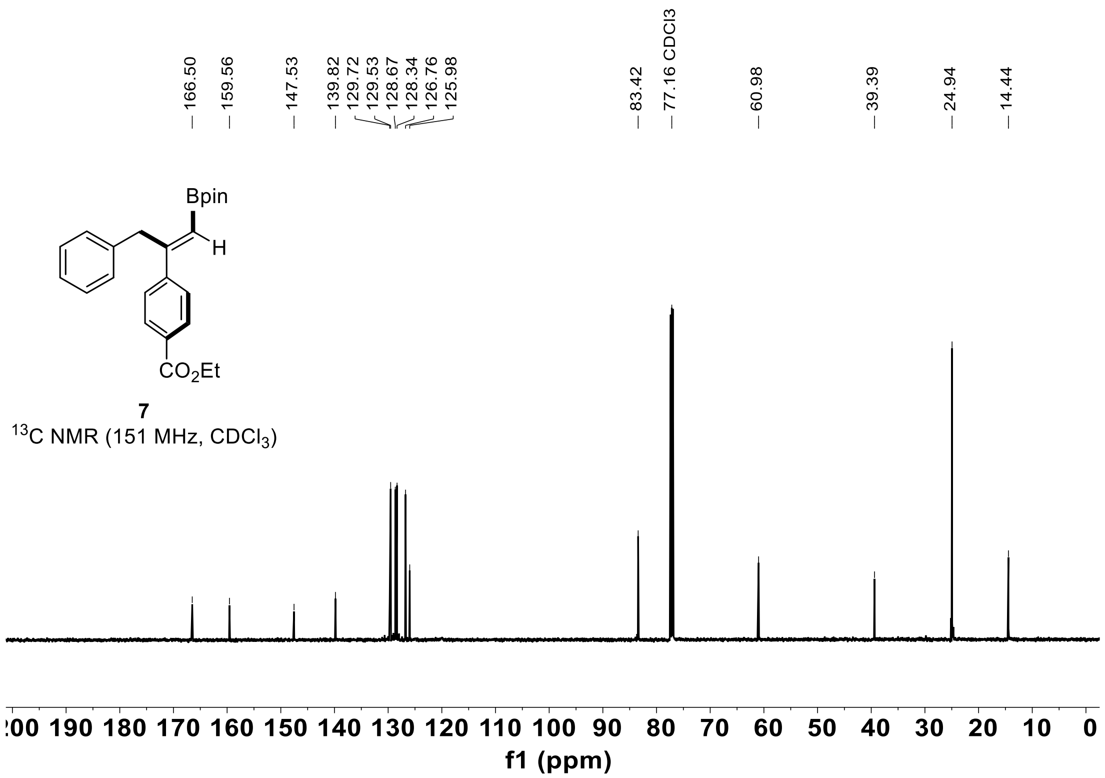
166.50
159.56
147.53
139.82
129.72
129.53
128.67
128.34
126.76
125.98
83.42
77.16 CDCl3
60.98
39.39
24.94
14.44
Bpin
H
CO_2Et
7
^{13}C NMR (151 MHz, $CDCl_3$)
200 190 180 170 160 150 140 130 120 110 100 90 80 70 60 50 40 30 20 10 0
f1 (ppm)

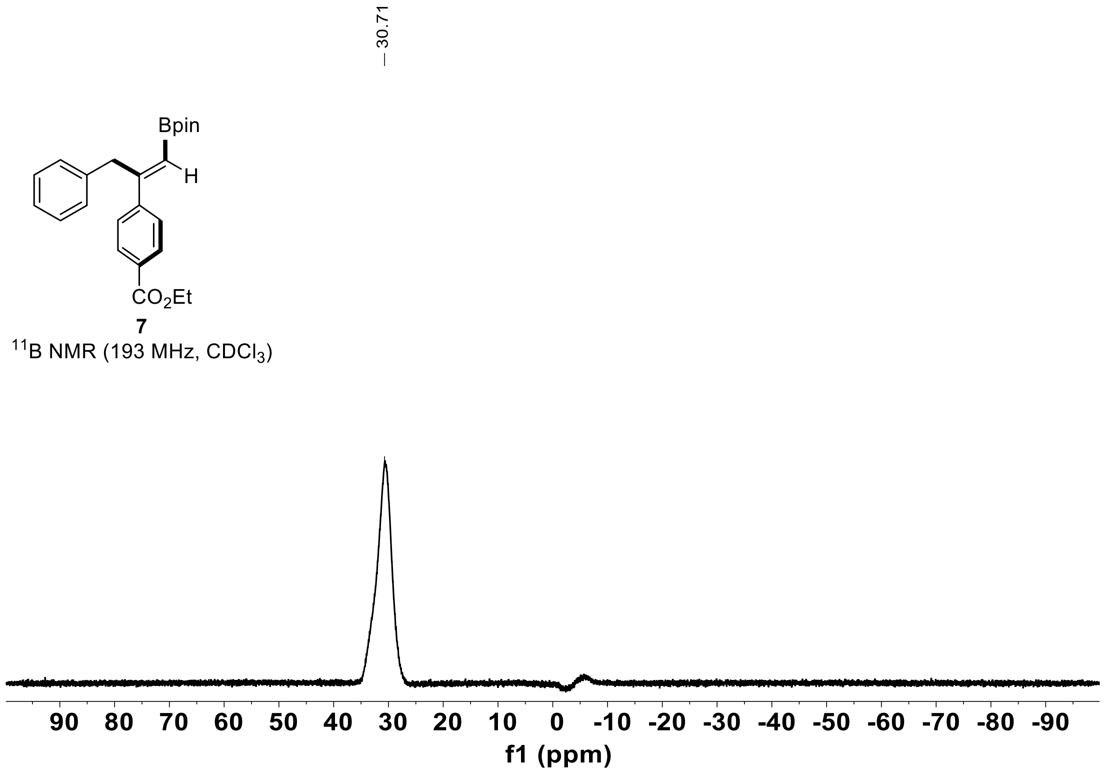
30.71
Bpin
H
CO_2Et
7
^{11}B NMR (193 MHz, $CDCl_3$)
90 80 70 60 50 40 30 20 10 0 -10 -20 -30 -40 -50 -60 -70 -80 -90
f1 (ppm)

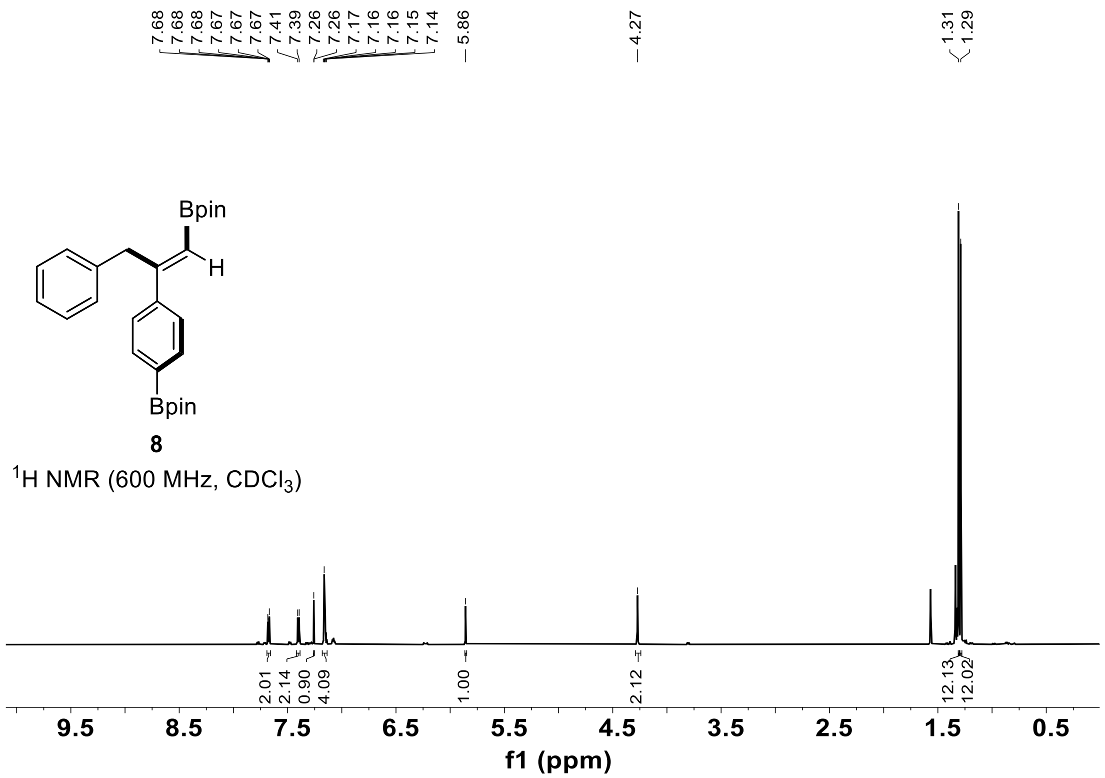
7.68 7.68 7.68 7.67 7.67 7.67 7.41 7.39 7.26 7.26 7.17 7.16 7.16 7.15 7.14
5.86
4.27
1.31 1.29
Bpin
H
Bpin
8
^{1}H NMR (600 MHz, $CDCl_3$)
2.01 2.14 0.90 4.09
1.00
2.12
12.13 12.02
9.5 8.5 7.5 6.5 5.5 4.5 3.5 2.5 1.5 0.5
f1 (ppm)

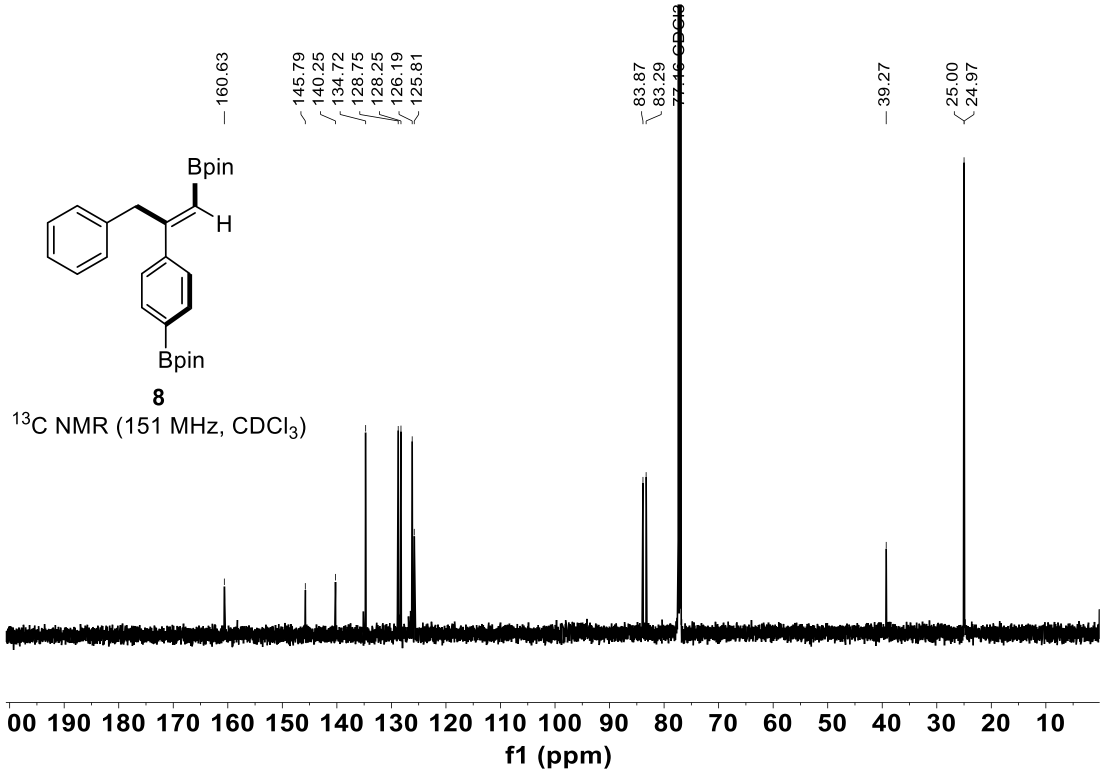
160.63
145.79 140.25 134.72 128.75 128.25 126.19 125.81
83.87 83.29 77.16 CDCl3
39.27
25.00 24.97
Bpin
H
Bpin
8
^{13}C NMR (151 MHz, $CDCl_3$)
00 190 180 170 160 150 140 130 120 110 100 90 80 70 60 50 40 30 20 10
f1 (ppm)

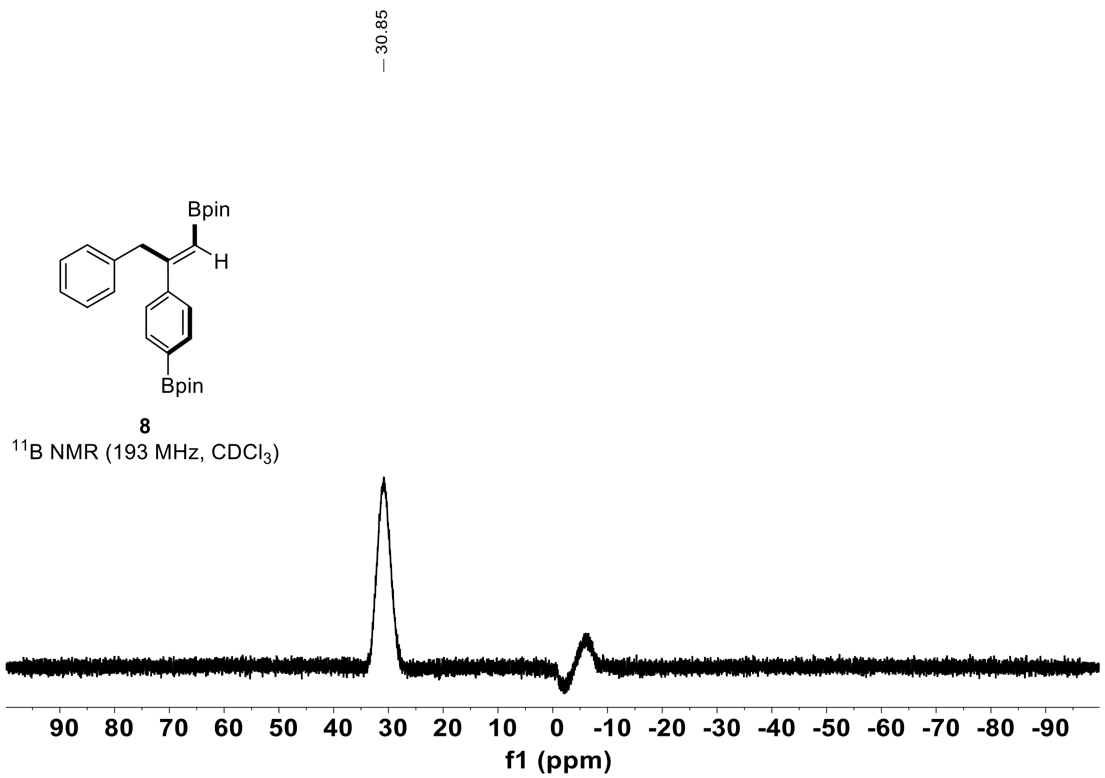

8

^{11}B NMR (193 MHz, $CDCl_3$)

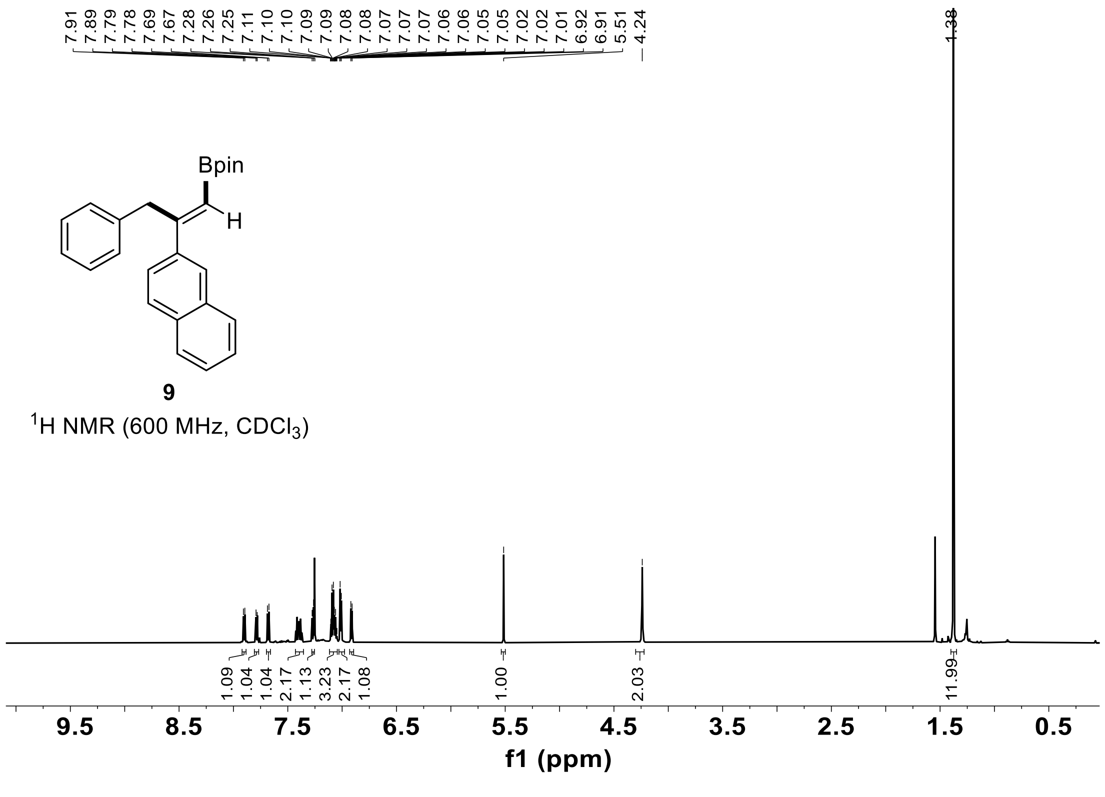

9

^{1}H NMR (600 MHz, $CDCl_3$)

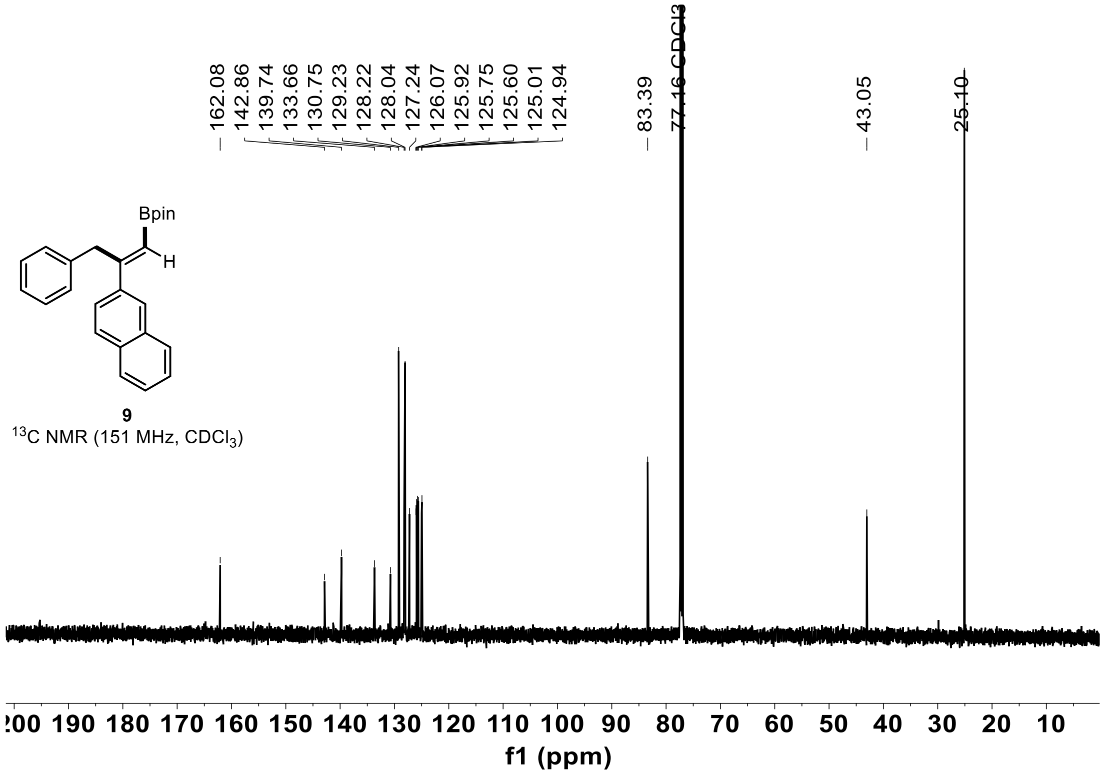

162.08
142.86
139.74
133.66
130.75
129.23
128.22
128.04
127.24
126.07
125.92
125.75
125.60
125.01
124.94
83.39
77.16 CDCl3
43.05
25.10
Bpin
H
9
^{13}C NMR (151 MHz, CDCl$_3$)
200 190 180 170 160 150 140 130 120 110 100 90 80 70 60 50 40 30 20 10
f1 (ppm)

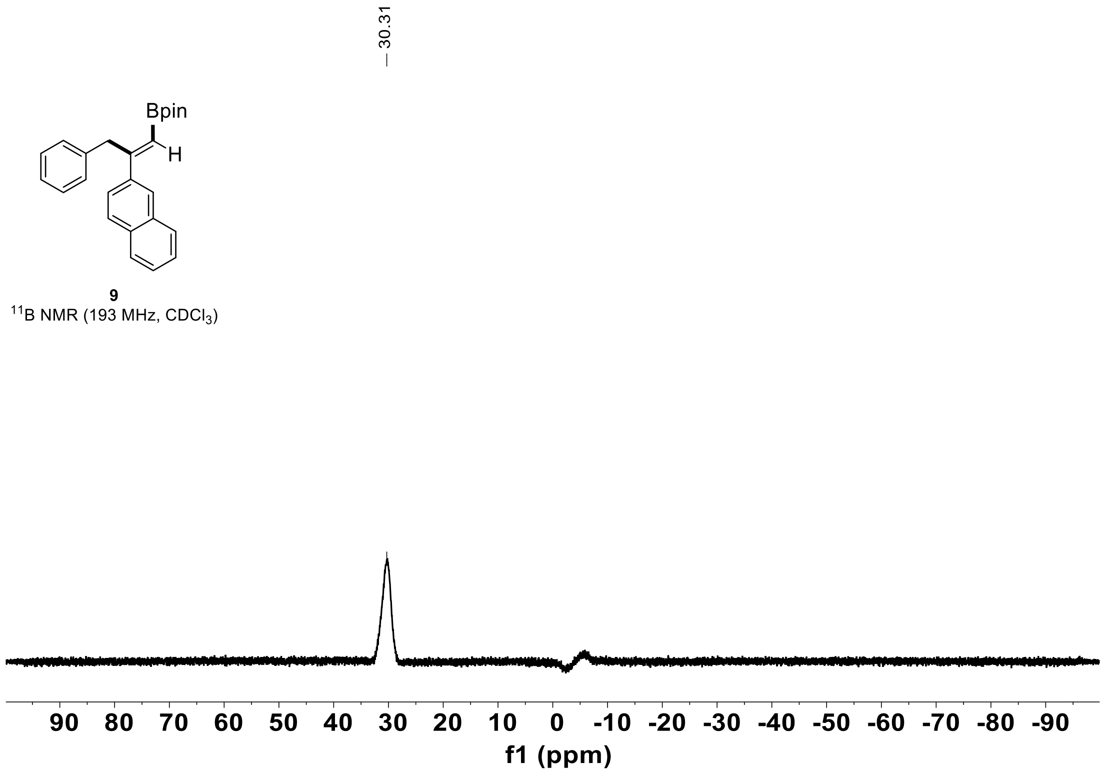

30.31
Bpin
H
9
^{11}B NMR (193 MHz, CDCl$_3$)
90 80 70 60 50 40 30 20 10 0 -10 -20 -30 -40 -50 -60 -70 -80 -90
f1 (ppm)

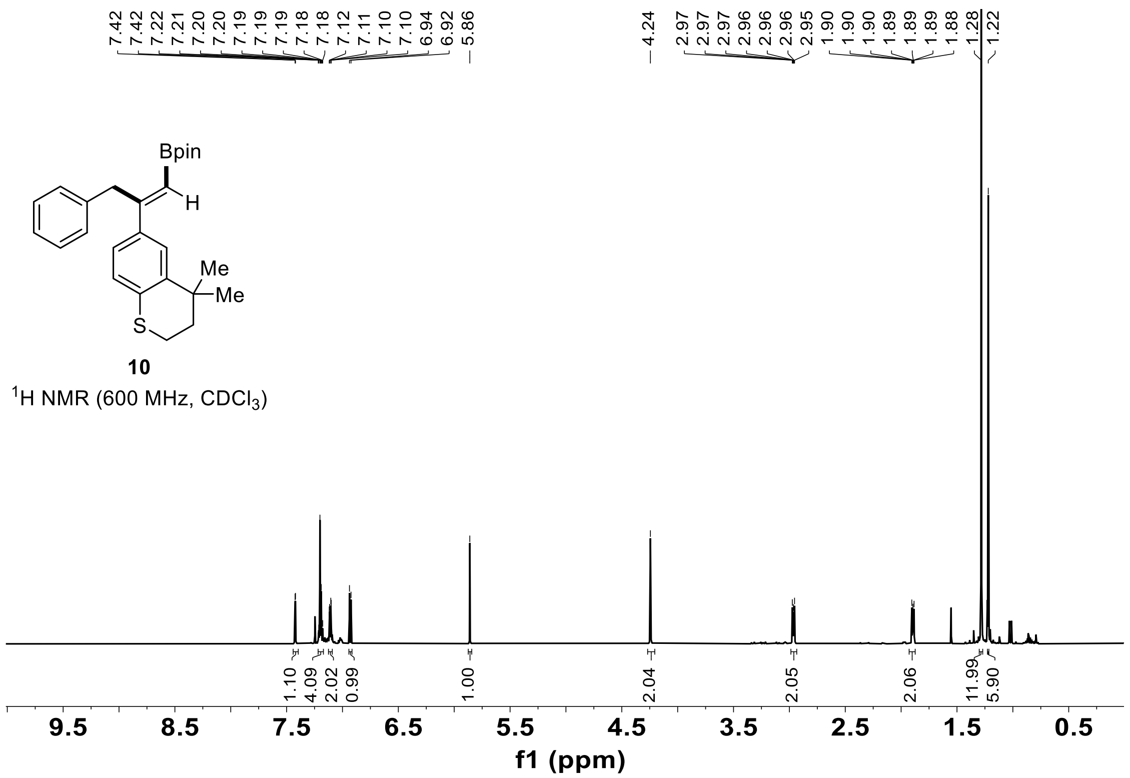
7.42
7.42
7.22
7.21
7.20
7.20
7.19
7.19
7.19
7.18
7.18
7.12
7.11
7.10
7.10
6.94
6.92
5.86
4.24
2.97
2.97
2.97
2.96
2.96
2.96
2.95
1.90
1.90
1.90
1.89
1.89
1.89
1.88
1.28
1.22
Bpin
H
Me
Me
S
10
^{1}H NMR (600 MHz, CDCl$_3$)
1.10
4.09
2.02
0.99
1.00
2.04
2.05
2.06
11.99
5.90
9.5
8.5
7.5
6.5
5.5
4.5
3.5
2.5
1.5
0.5
f1 (ppm)

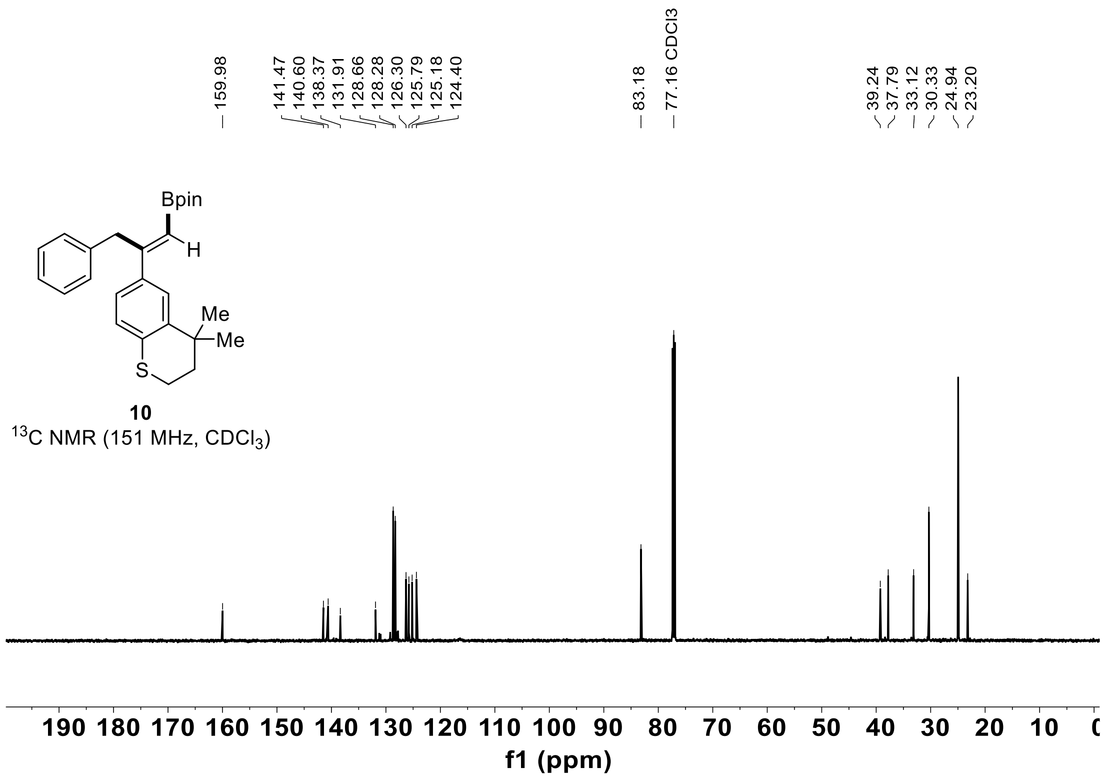
159.98
141.47
140.60
138.37
131.91
128.66
128.28
126.30
125.79
125.18
124.40
83.18
77.16 CDCl3
39.24
37.79
33.12
30.33
24.94
23.20
Bpin
H
Me
Me
S
10
^{13}C NMR (151 MHz, CDCl$_3$)
190 180 170 160 150 140 130 120 110 100 90 80 70 60 50 40 30 20 10 0
f1 (ppm)

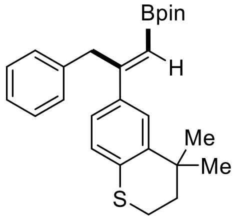

10

^{11}B NMR (193 MHz, $CDCl_3$)

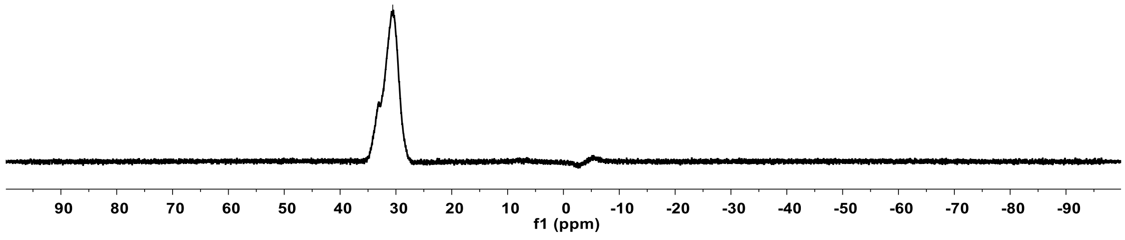

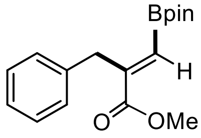

11

^{1}H NMR (600 MHz, $CDCl_3$)

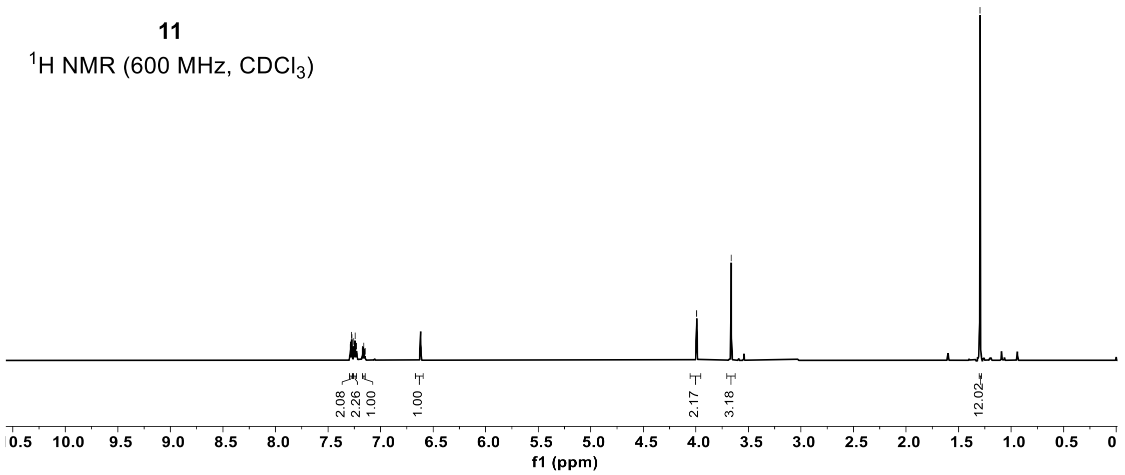

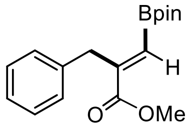

11

^{13}C NMR (151 MHz, $CDCl_3$)

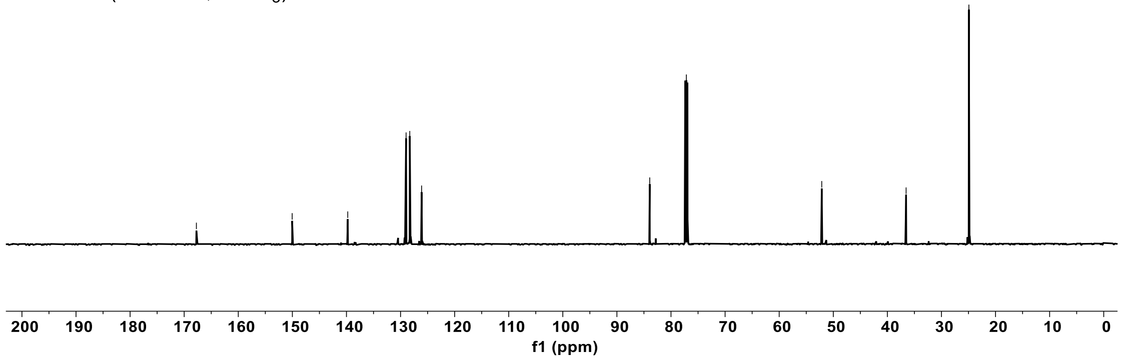

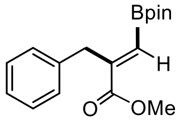

11

^{11}B NMR (193 MHz, $CDCl_3$)

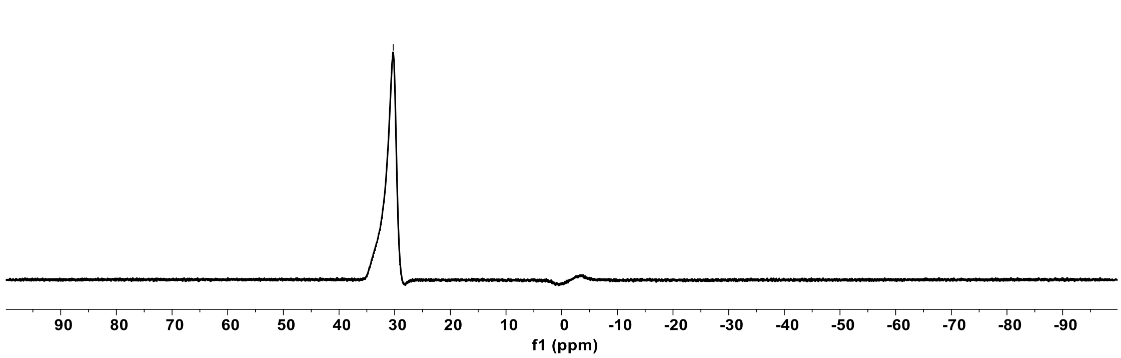

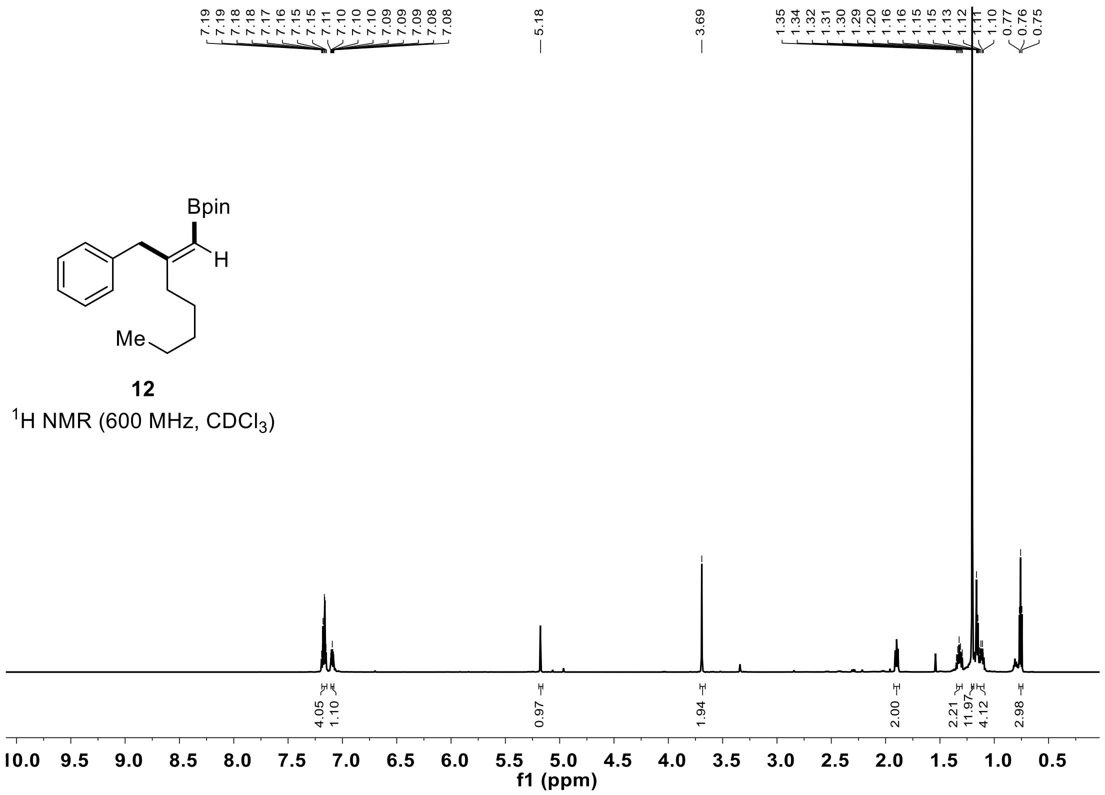
7.19 7.19 7.18 7.18 7.17 7.16 7.15 7.15 7.11 7.10 7.10 7.09 7.09 7.09 7.08 7.08
5.18
3.69
1.35 1.34 1.32 1.31 1.30 1.29 1.20 1.16 1.16 1.15 1.15 1.13 1.12 1.11 1.10 0.77 0.76 0.75
Bpin
H
Me
12
^{1}H NMR (600 MHz, CDCl$_3$)
4.05 1.10 0.97 1.94 2.00 2.21 11.97 4.12 2.98
f1 (ppm)

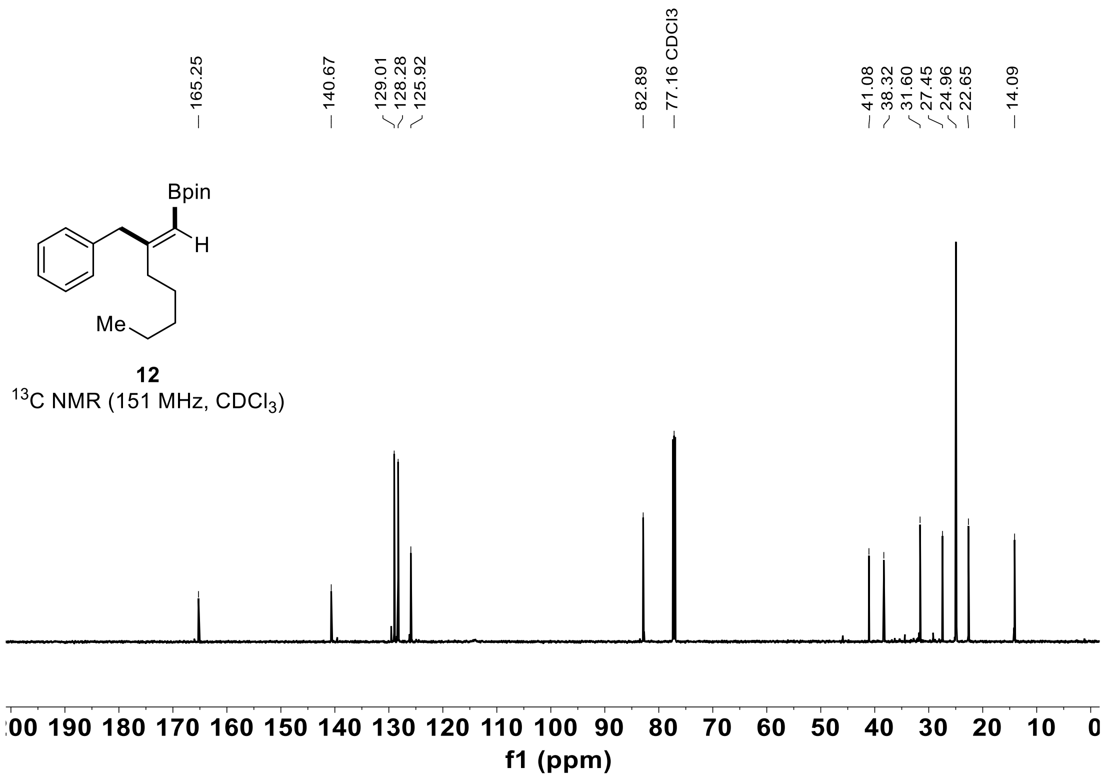
165.25
140.67
129.01 128.28 125.92
82.89
77.16 CDCl3
41.08 38.32 31.60 27.45 24.96 22.65
14.09
Bpin
H
Me
12
^{13}C NMR (151 MHz, CDCl$_3$)
f1 (ppm)

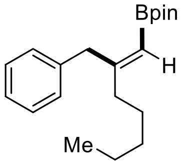

12

^{11}B NMR (193 MHz, $CDCl_3$)

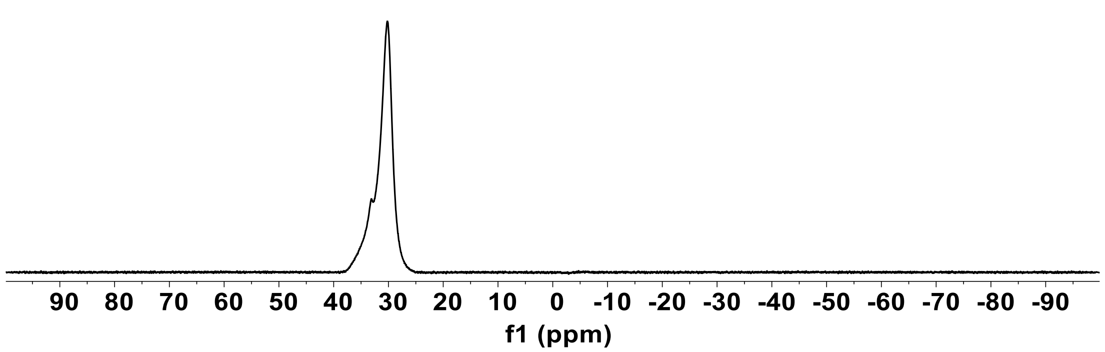

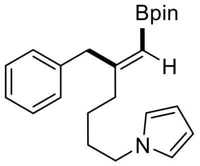

13

^{1}H NMR (600 MHz, $CDCl_3$)

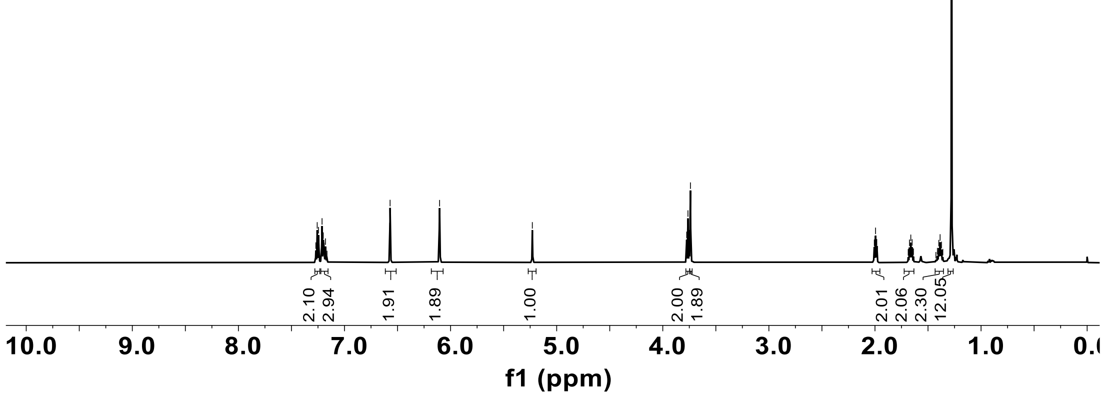

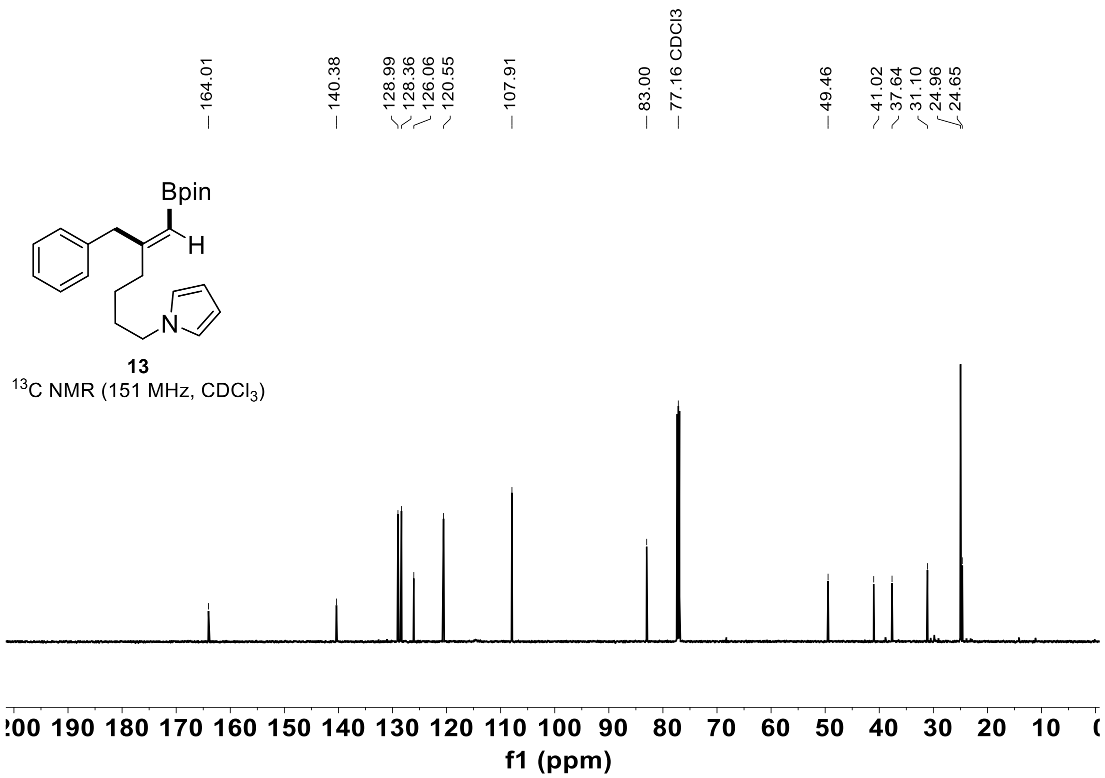
164.01
140.38
128.99
128.36
126.06
120.55
107.91
83.00
77.16 CDCl3
49.46
41.02
37.64
31.10
24.96
24.65
Bpin
H
N
13
^{13}C NMR (151 MHz, $CDCl_3$)
200 190 180 170 160 150 140 130 120 110 100 90 80 70 60 50 40 30 20 10 0
f1 (ppm)

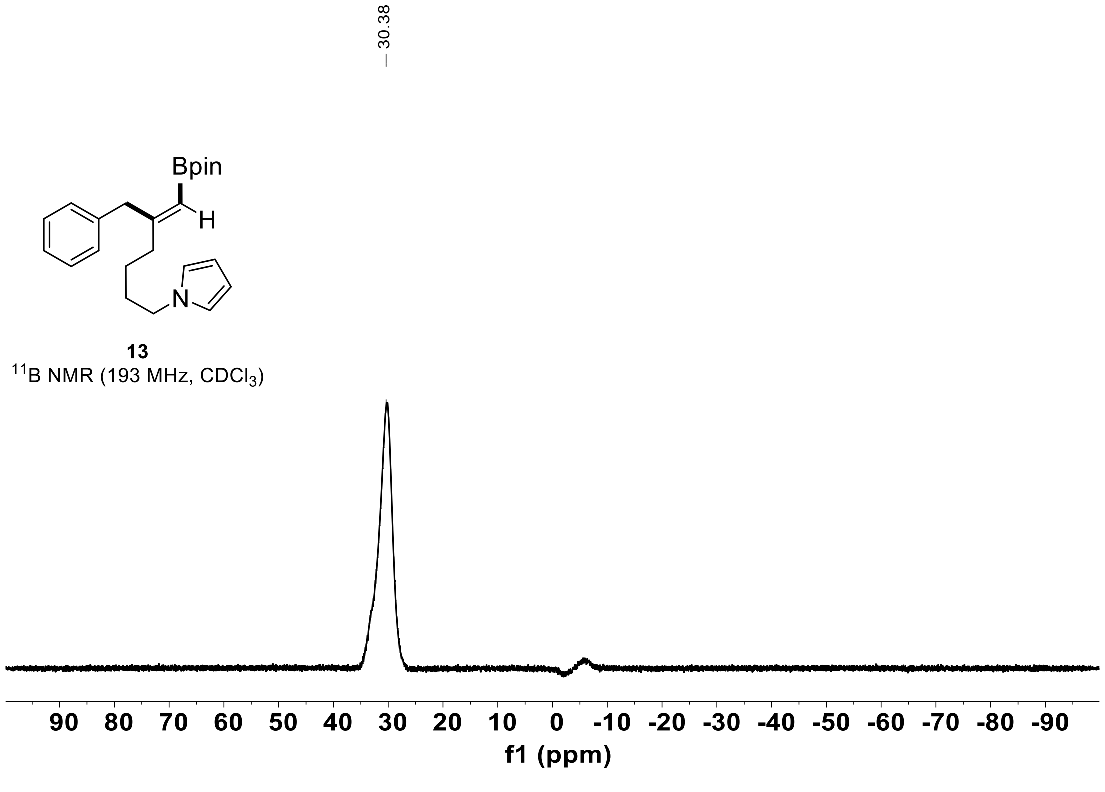
30.38
Bpin
H
N
13
^{11}B NMR (193 MHz, $CDCl_3$)
90 80 70 60 50 40 30 20 10 0 -10 -20 -30 -40 -50 -60 -70 -80 -90
f1 (ppm)

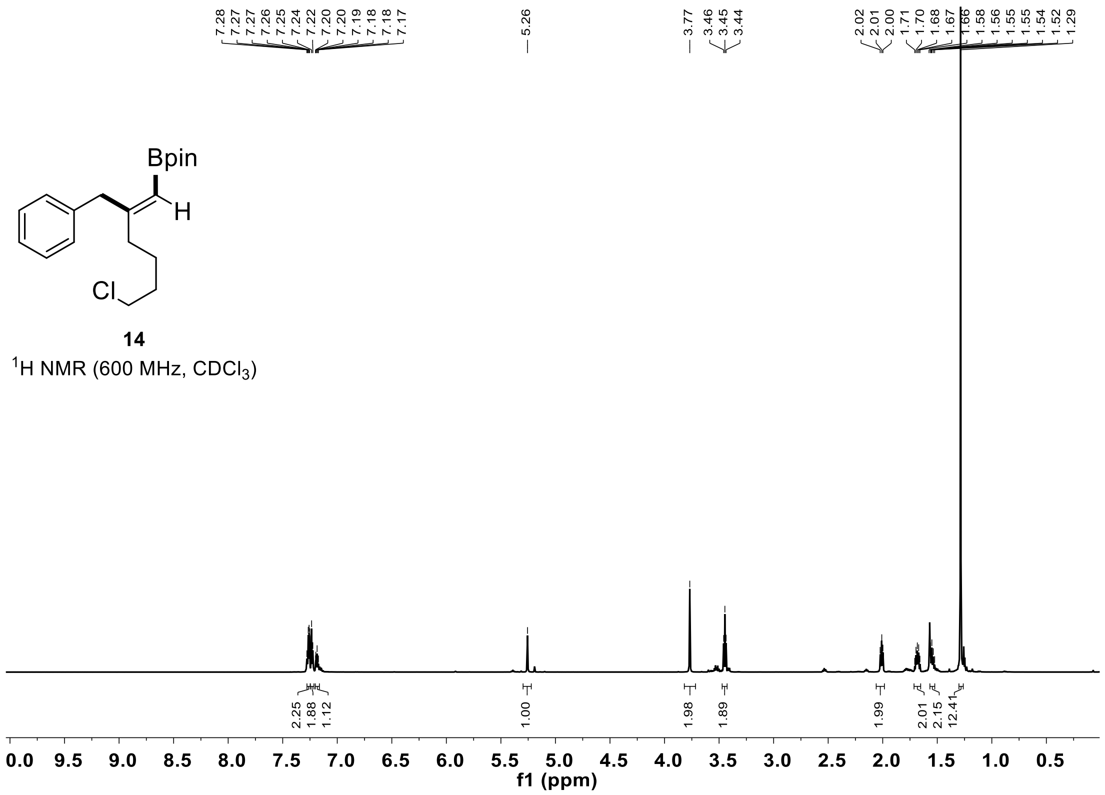
Bpin
H
Cl
14
^{1}H NMR (600 MHz, CDCl$_3$)
f1 (ppm)

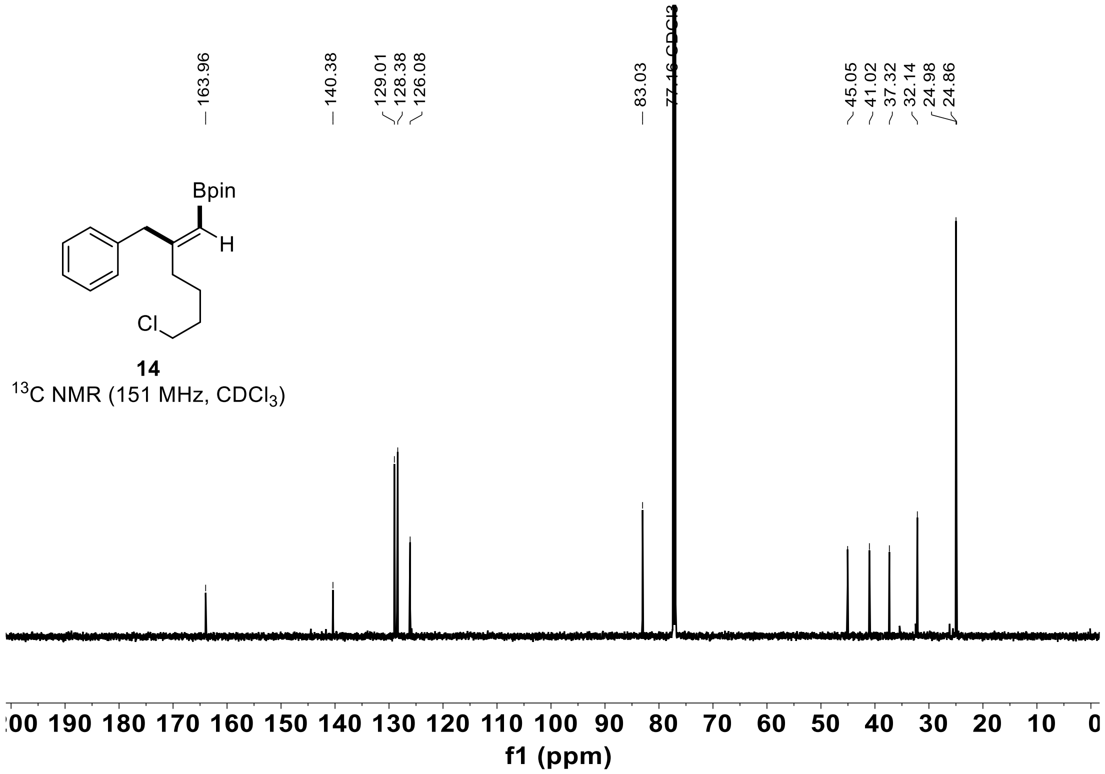
163.96
140.38
129.01
128.38
126.08
83.03
77.16 CDCl3
45.05
41.02
37.32
32.14
24.98
24.86
Bpin
H
Cl
14
^{13}C NMR (151 MHz, CDCl$_3$)
f1 (ppm)

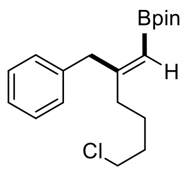

14

^{11}B NMR (193 MHz, $CDCl_3$)

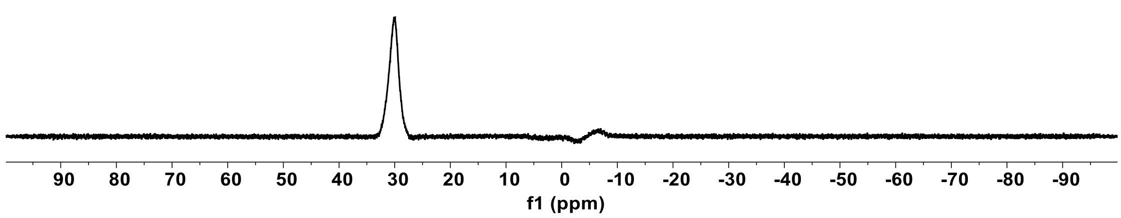

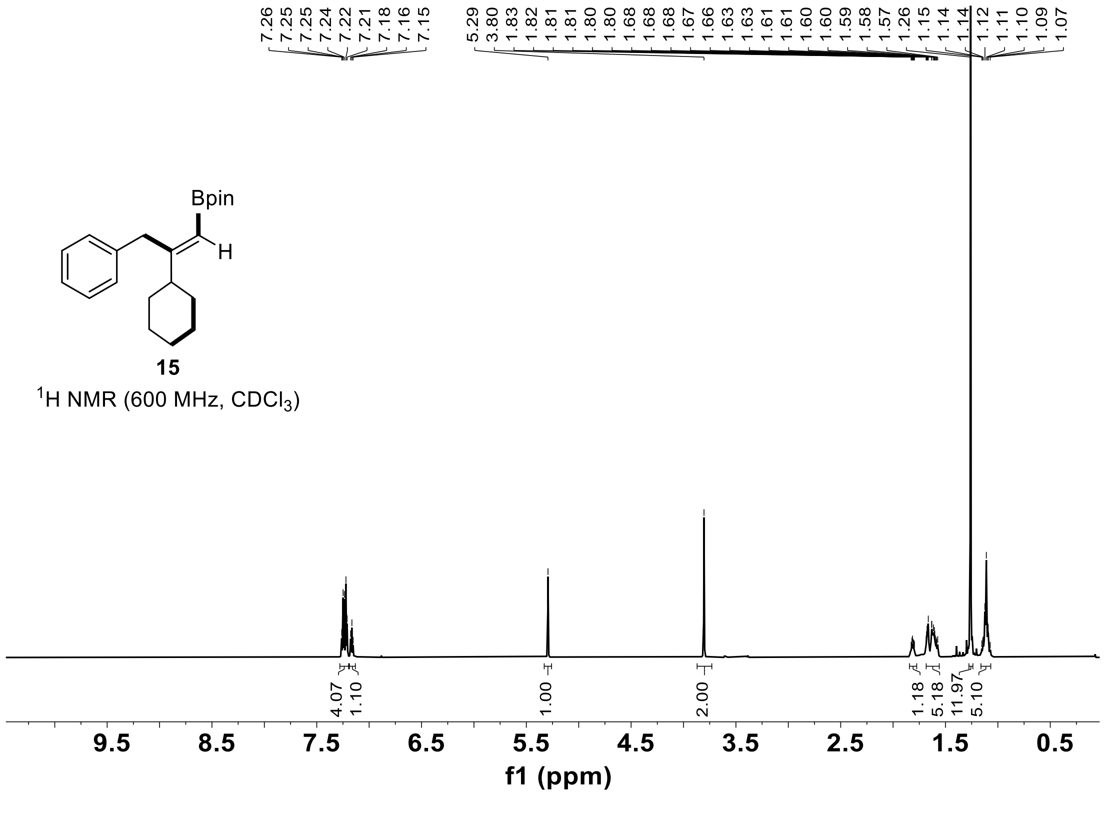

15

^{1}H NMR (600 MHz, $CDCl_3$)

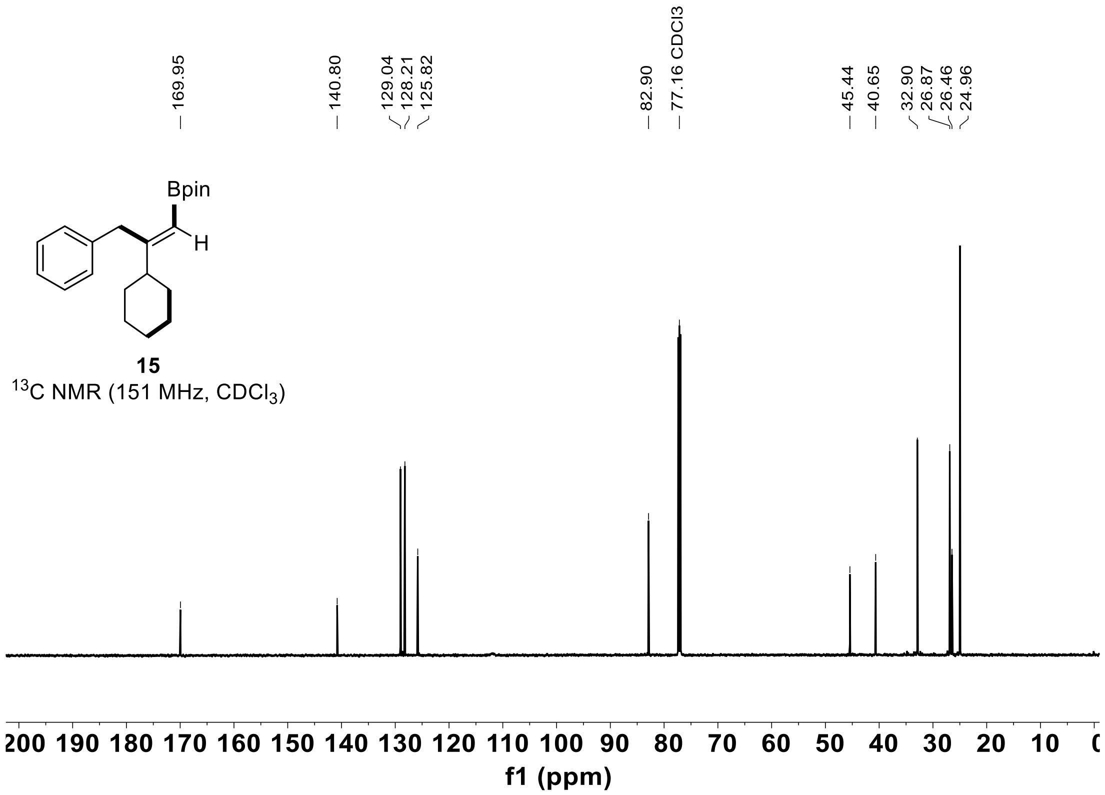

15
^{13}C NMR (151 MHz, $CDCl_3$)

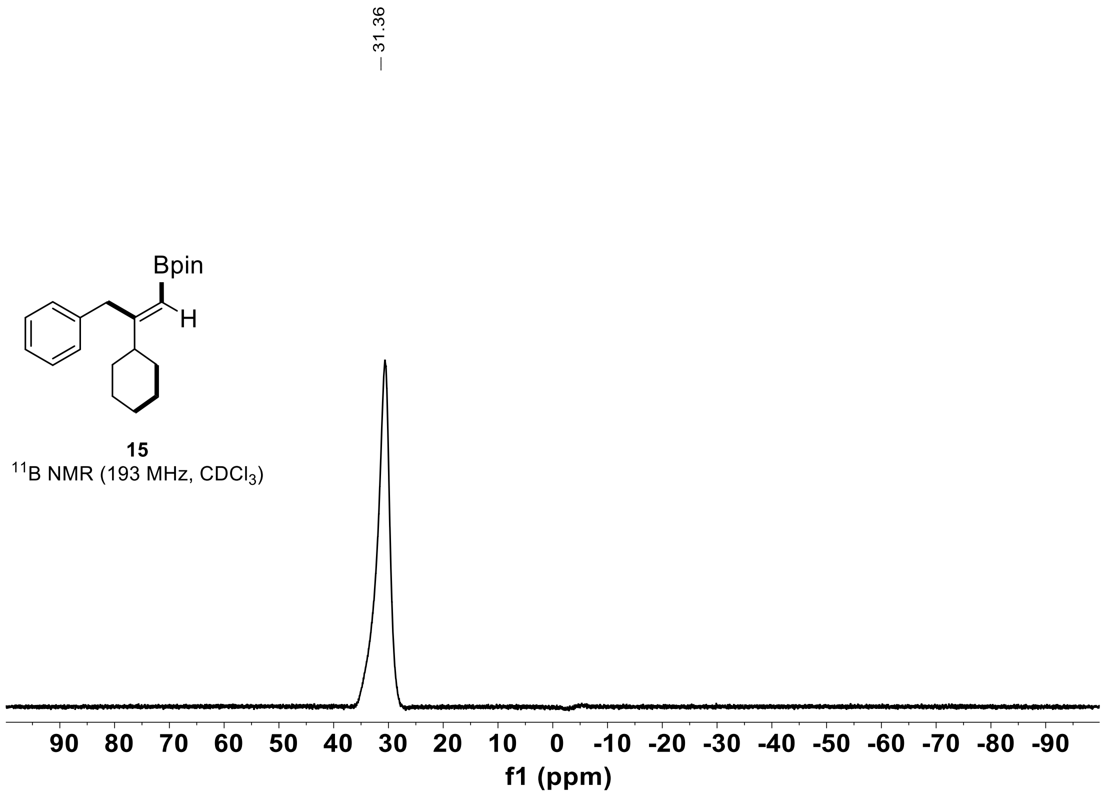

15
^{11}B NMR (193 MHz, $CDCl_3$)

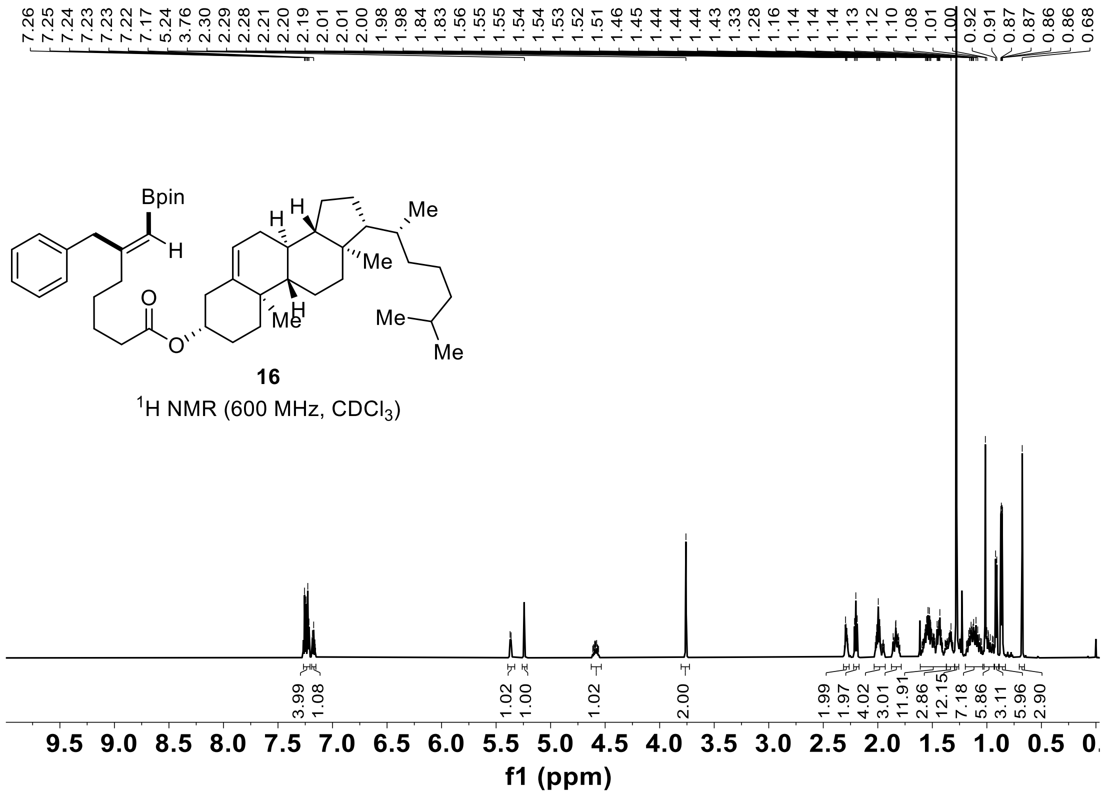
16
^{1}H NMR (600 MHz, $CDCl_3$)
f1 (ppm)

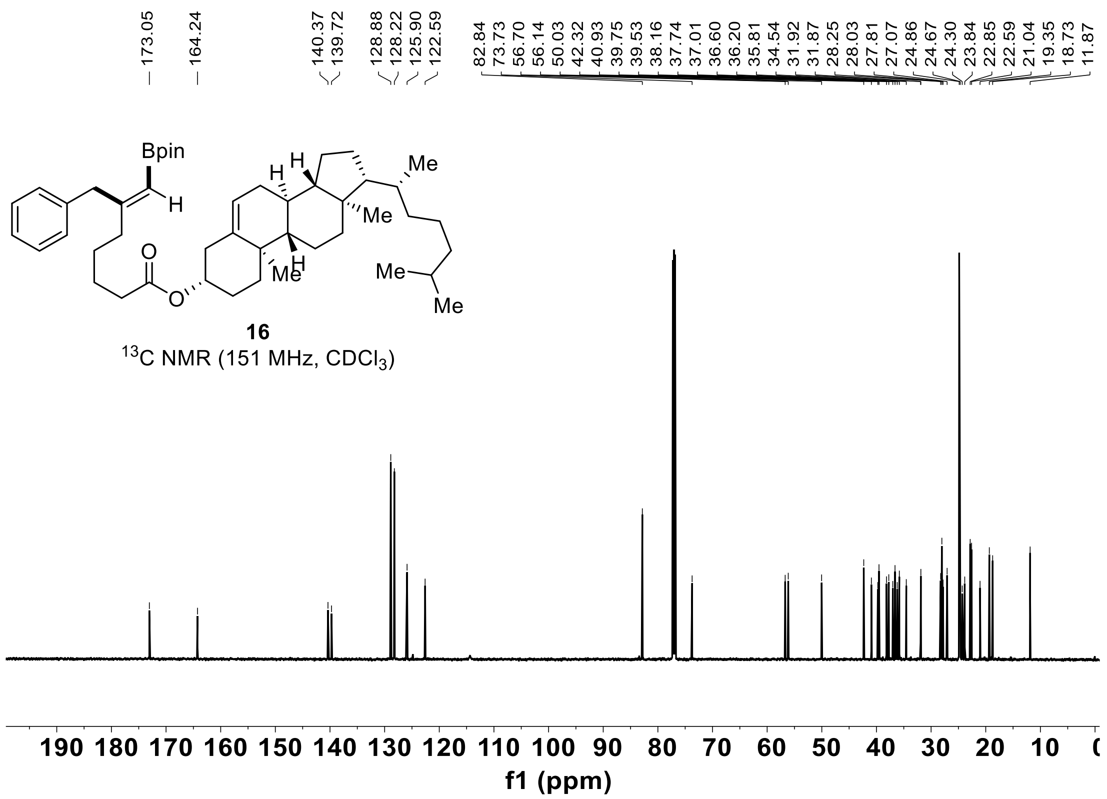
16
^{13}C NMR (151 MHz, $CDCl_3$)
f1 (ppm)

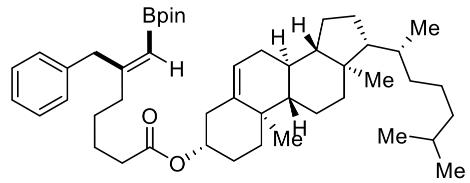

16

^{11}B NMR (193 MHz, CDCl$_3$)

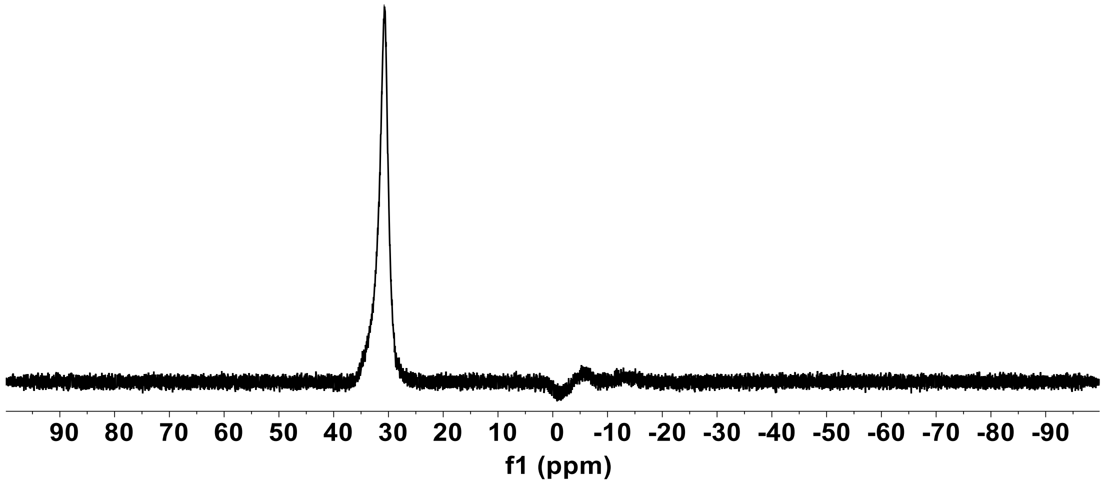

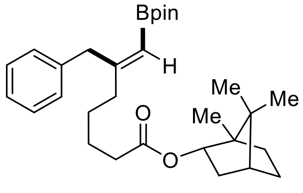

17

^{1}H NMR (600 MHz, CDCl$_3$)

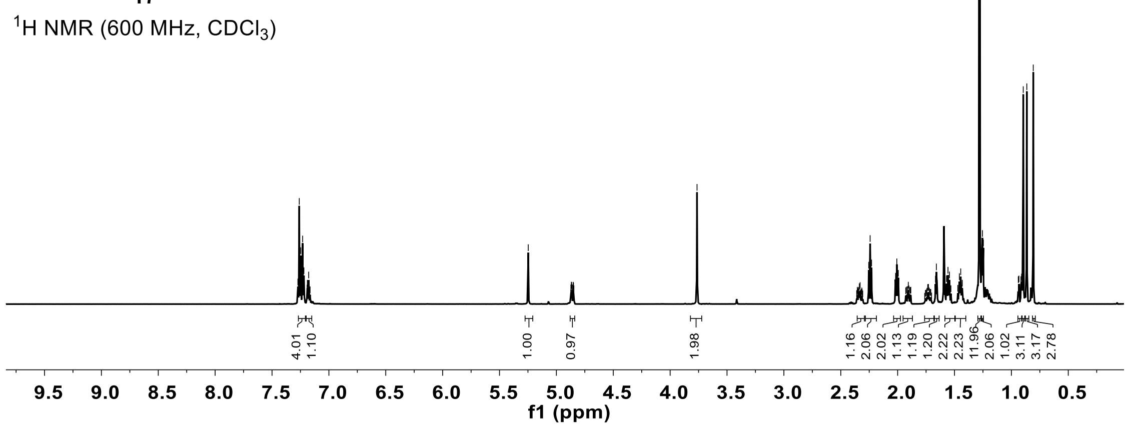

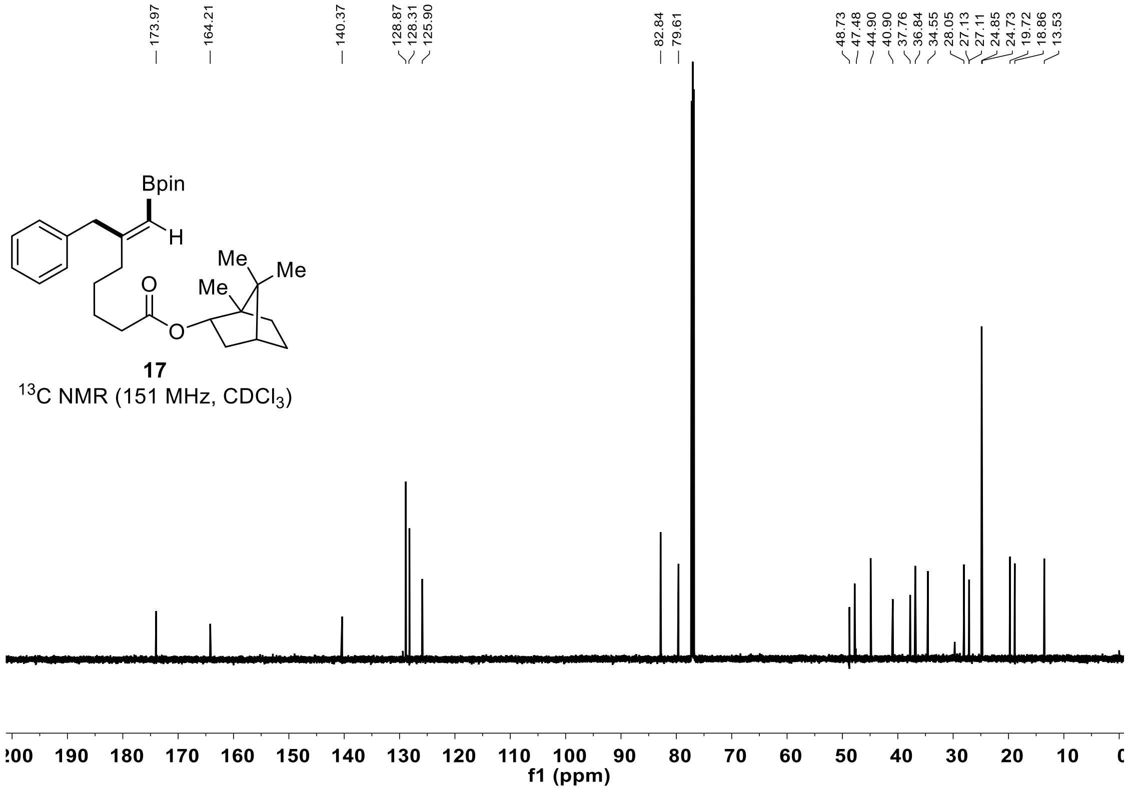

17

^{13}C NMR (151 MHz, $CDCl_3$)

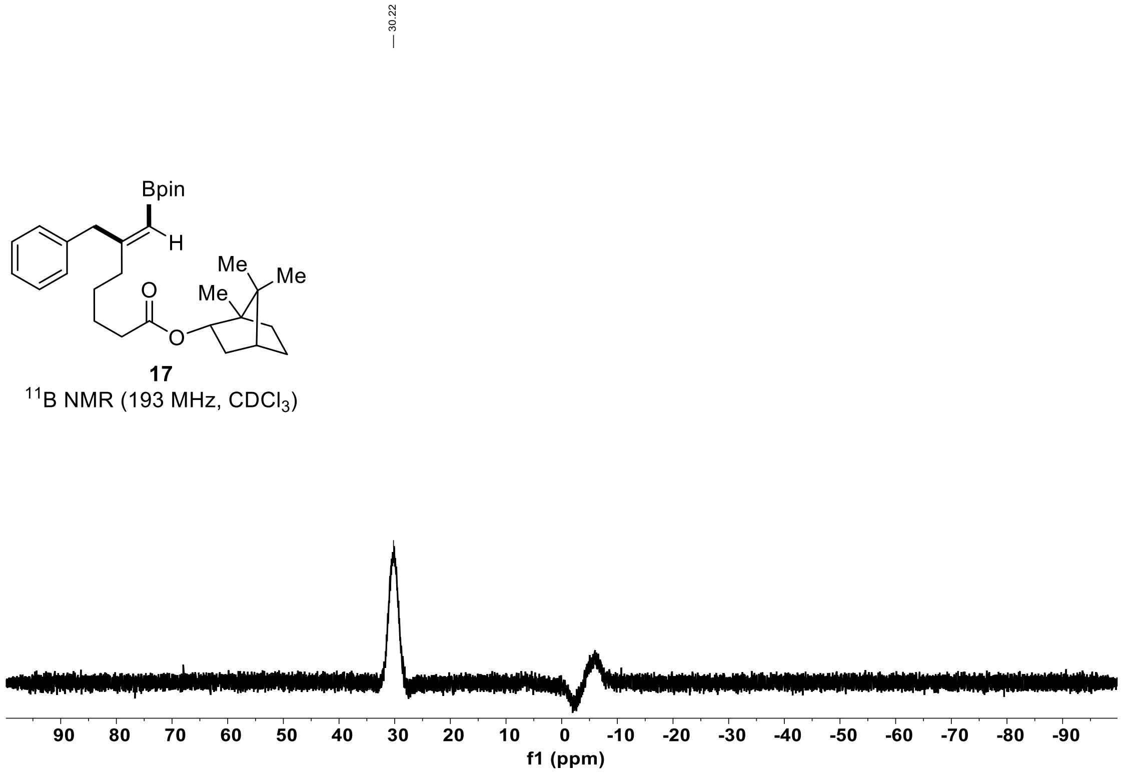

17

^{11}B NMR (193 MHz, $CDCl_3$)

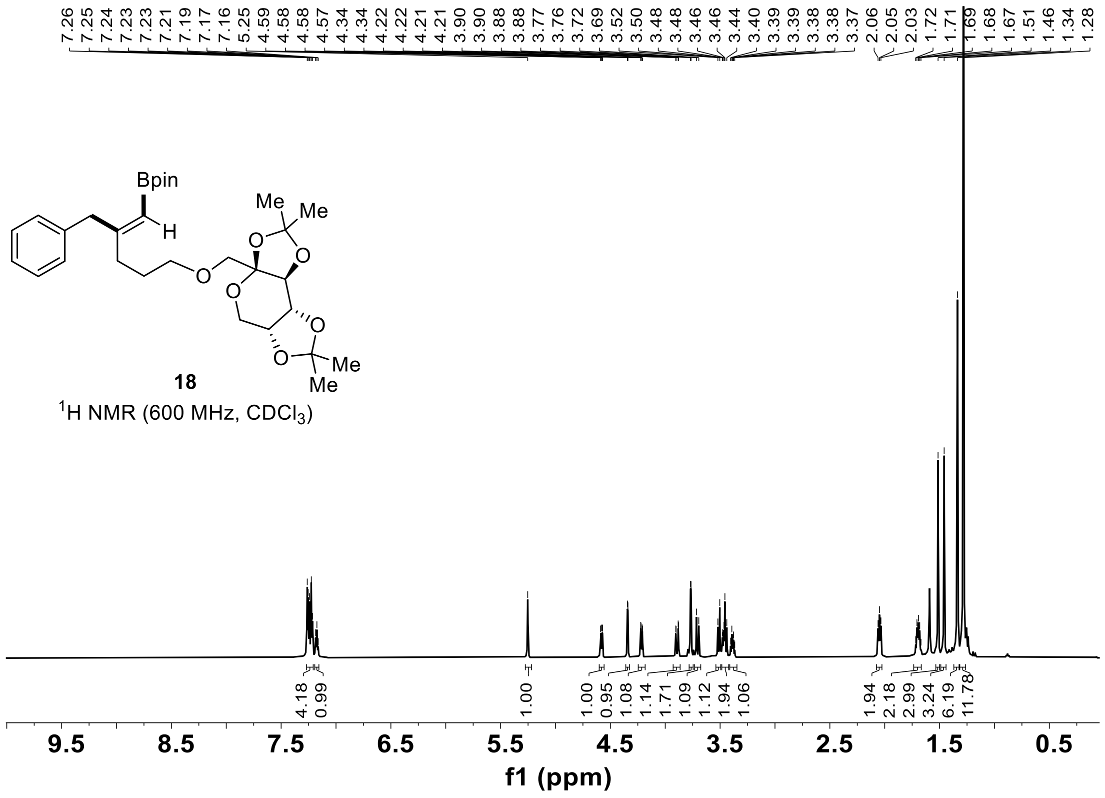

7.26 7.25 7.24 7.23 7.23 7.21 7.19 7.17 7.16 5.25 4.59 4.58 4.58 4.57 4.34 4.34 4.22 4.22 4.21 4.21 3.90 3.90 3.88 3.88 3.77 3.76 3.72 3.69 3.52 3.50 3.48 3.48 3.46 3.44 3.40 3.39 3.39 3.38 3.38 3.37 2.06 2.05 2.03 1.72 1.71 1.69 1.68 1.67 1.51 1.46 1.34 1.28
Bpin
H
Me
Me
O
O
O
O
O
O
Me
Me
18
^{1}H NMR (600 MHz, CDCl$_3$)
4.18 0.99 1.00 1.00 0.95 1.08 1.14 1.71 1.09 1.12 1.94 1.06 1.94 2.18 2.99 3.24 6.19 11.78
9.5 8.5 7.5 6.5 5.5 4.5 3.5 2.5 1.5 0.5
f1 (ppm)

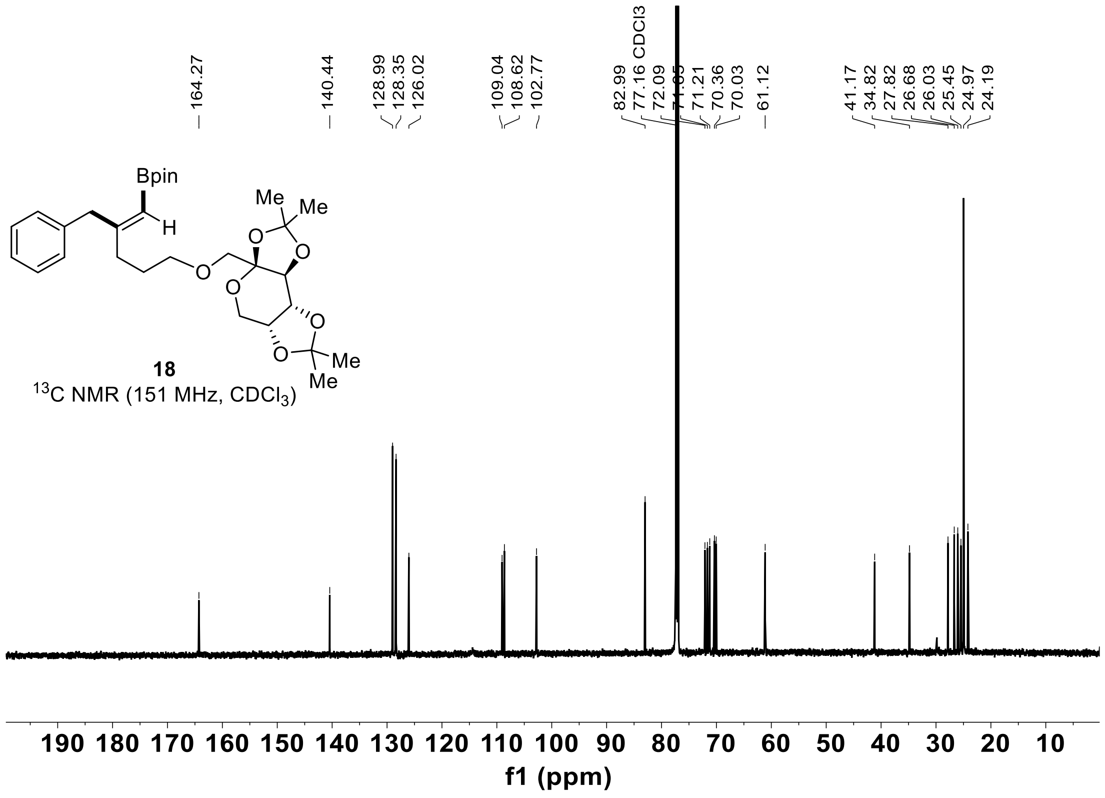

164.27 140.44 128.99 128.35 126.02 109.04 108.62 102.77 82.99 77.16 CDCl3 72.09 71.21 70.36 70.03 61.12 41.17 34.82 27.82 26.68 26.03 25.45 24.97 24.19
Bpin
H
Me
Me
18
^{13}C NMR (151 MHz, CDCl$_3$)
Me
Me
190 180 170 160 150 140 130 120 110 100 90 80 70 60 50 40 30 20 10
f1 (ppm)

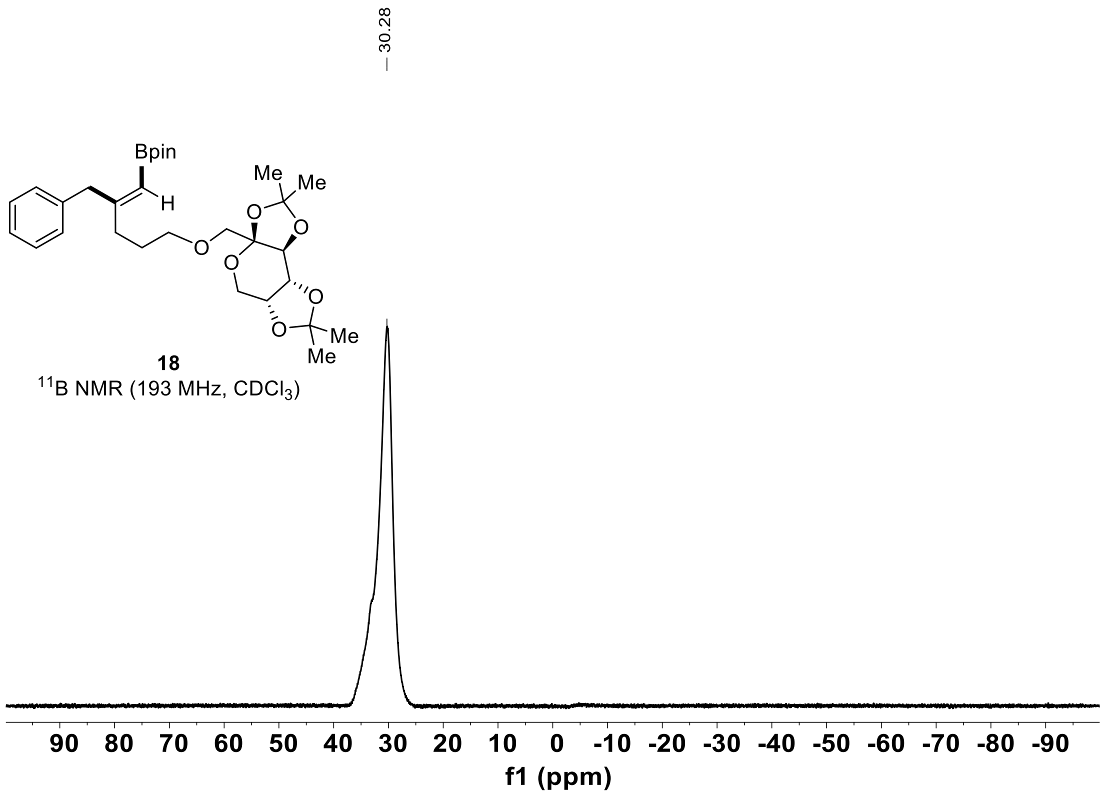
30.28
Bpin
H
Me
Me
O
O
O
O
O
O
O
Me
Me
18
^{11}B NMR (193 MHz, $CDCl_3$)
90 80 70 60 50 40 30 20 10 0 -10 -20 -30 -40 -50 -60 -70 -80 -90
f1 (ppm)

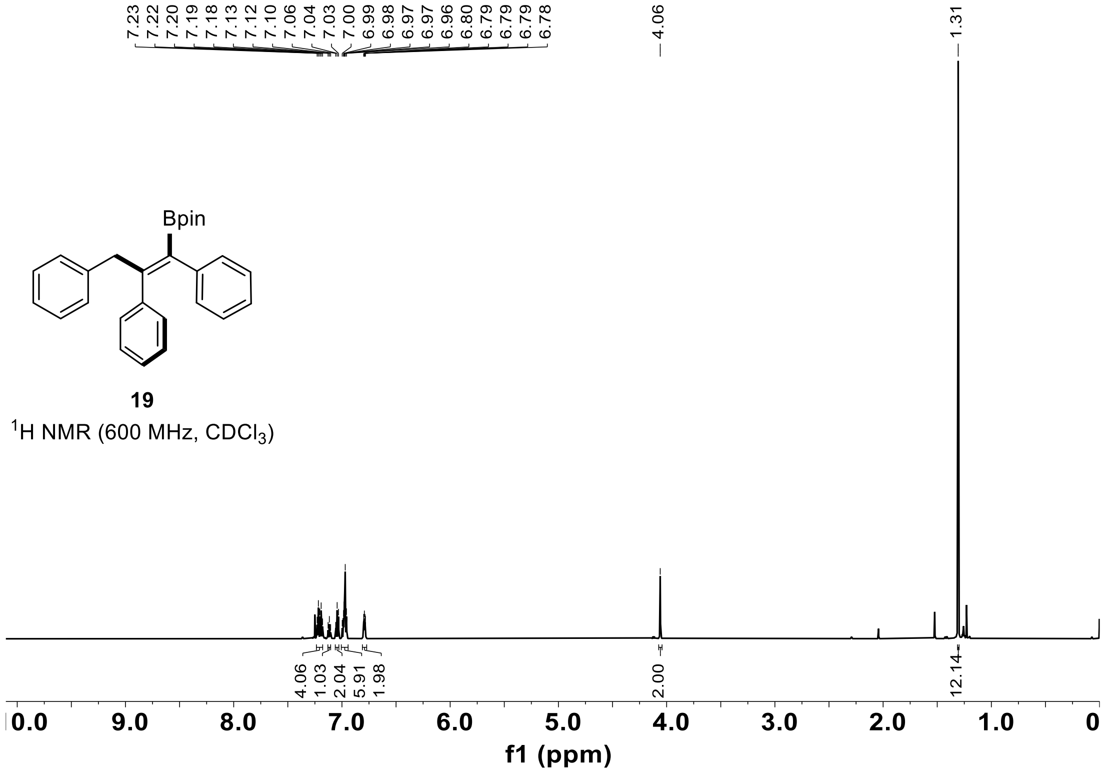
7.23 7.22 7.20 7.19 7.18 7.13 7.12 7.10 7.06 7.04 7.03 7.00 6.99 6.98 6.97 6.97 6.96 6.80 6.79 6.79 6.78
4.06
1.31
Bpin
19
^{1}H NMR (600 MHz, $CDCl_3$)
4.06 1.03 2.04 5.91 1.98
2.00
12.14
10.0 9.0 8.0 7.0 6.0 5.0 4.0 3.0 2.0 1.0 0
f1 (ppm)

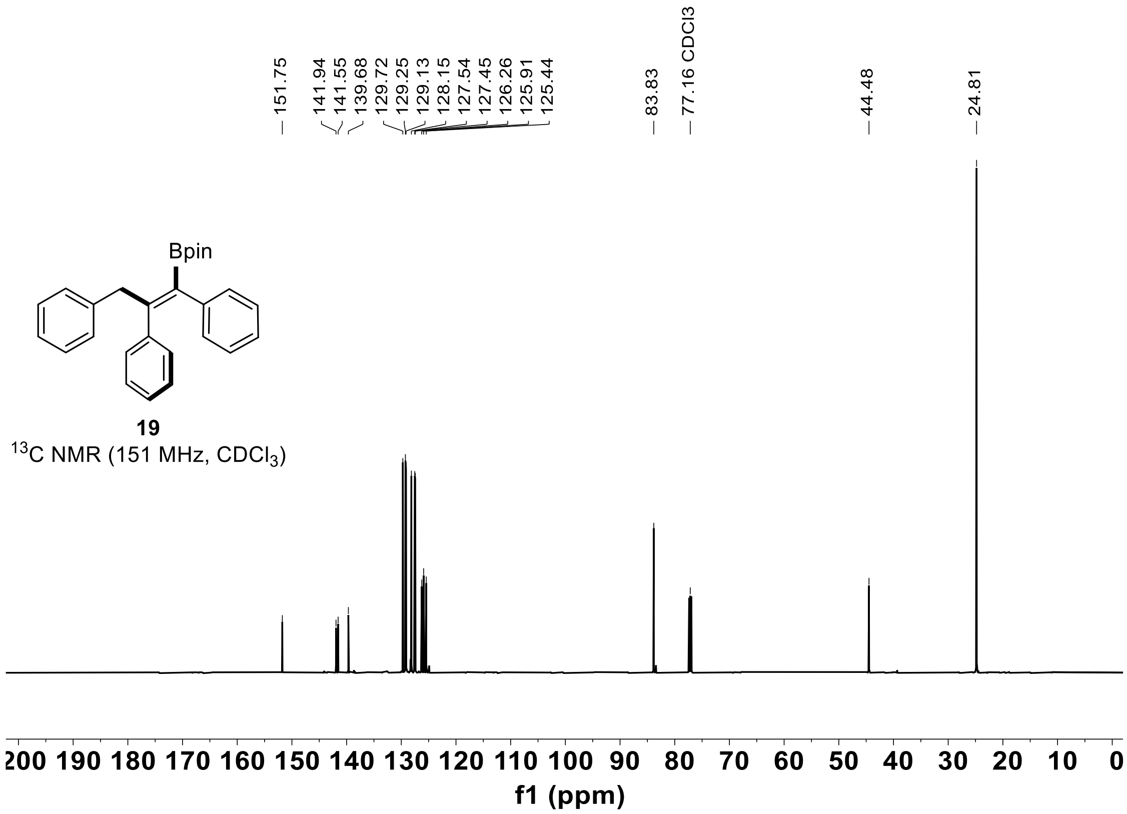

19

^{13}C NMR (151 MHz, $CDCl_3$)

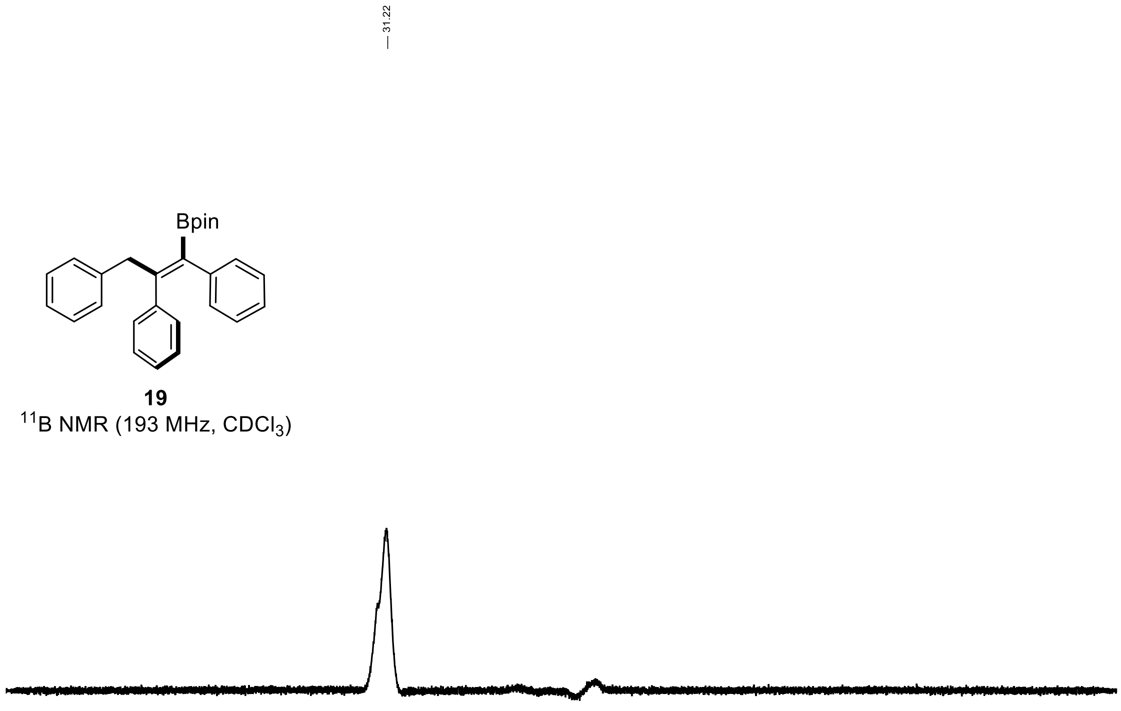

19

^{11}B NMR (193 MHz, $CDCl_3$)

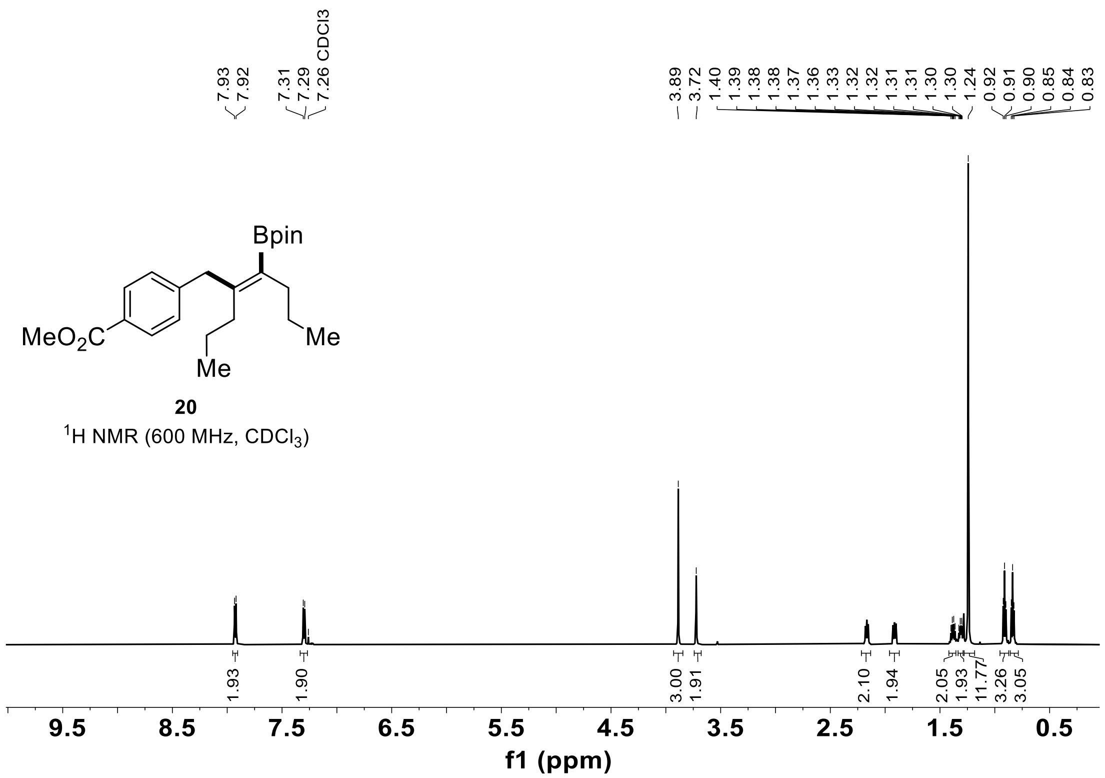
7.93
7.92
7.31
7.29
7.26 CDCl3
3.89
3.72
1.40
1.39
1.38
1.38
1.37
1.36
1.33
1.32
1.32
1.31
1.31
1.30
1.30
1.24
0.92
0.91
0.90
0.85
0.84
0.83
Bpin
MeO2C
Me
Me
20
1H NMR (600 MHz, CDCl3)
1.93
1.90
3.00
1.91
2.10
1.94
2.05
1.93
11.77
3.26
3.05
9.5
8.5
7.5
6.5
5.5
4.5
3.5
2.5
1.5
0.5
f1 (ppm)

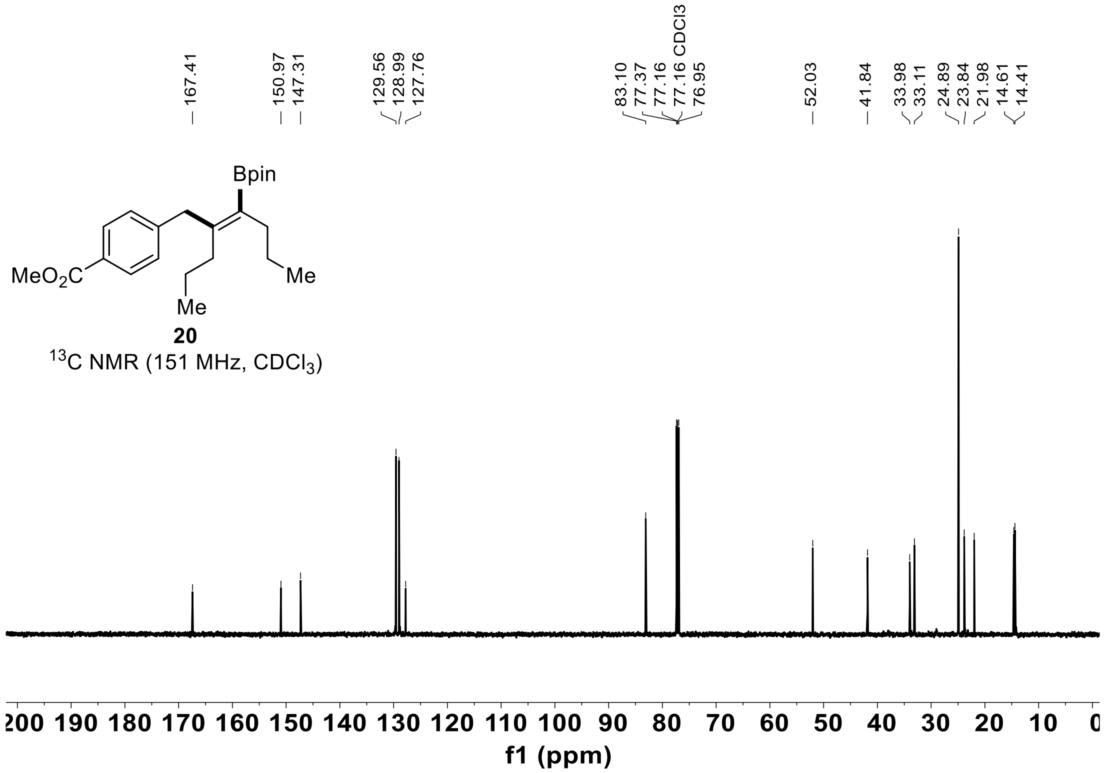
167.41
150.97
147.31
129.56
128.99
127.76
83.10
77.37
77.16
77.16 CDCl3
76.95
52.03
41.84
33.98
33.11
24.89
23.84
21.98
14.61
14.41
Bpin
MeO2C
Me
Me
20
13C NMR (151 MHz, CDCl3)
200 190 180 170 160 150 140 130 120 110 100 90 80 70 60 50 40 30 20 10 0
f1 (ppm)

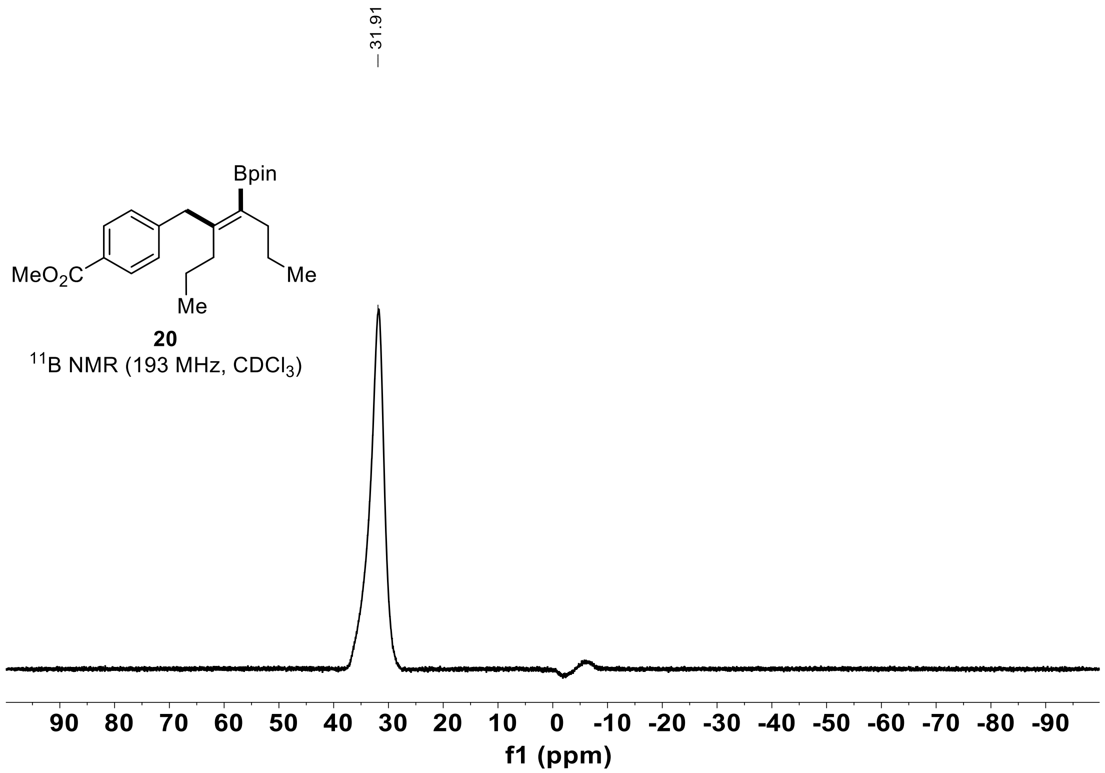

31.91
Bpin
MeO2C
Me
Me
20
11B NMR (193 MHz, CDCl3)
90 80 70 60 50 40 30 20 10 0 -10 -20 -30 -40 -50 -60 -70 -80 -90
f1 (ppm)

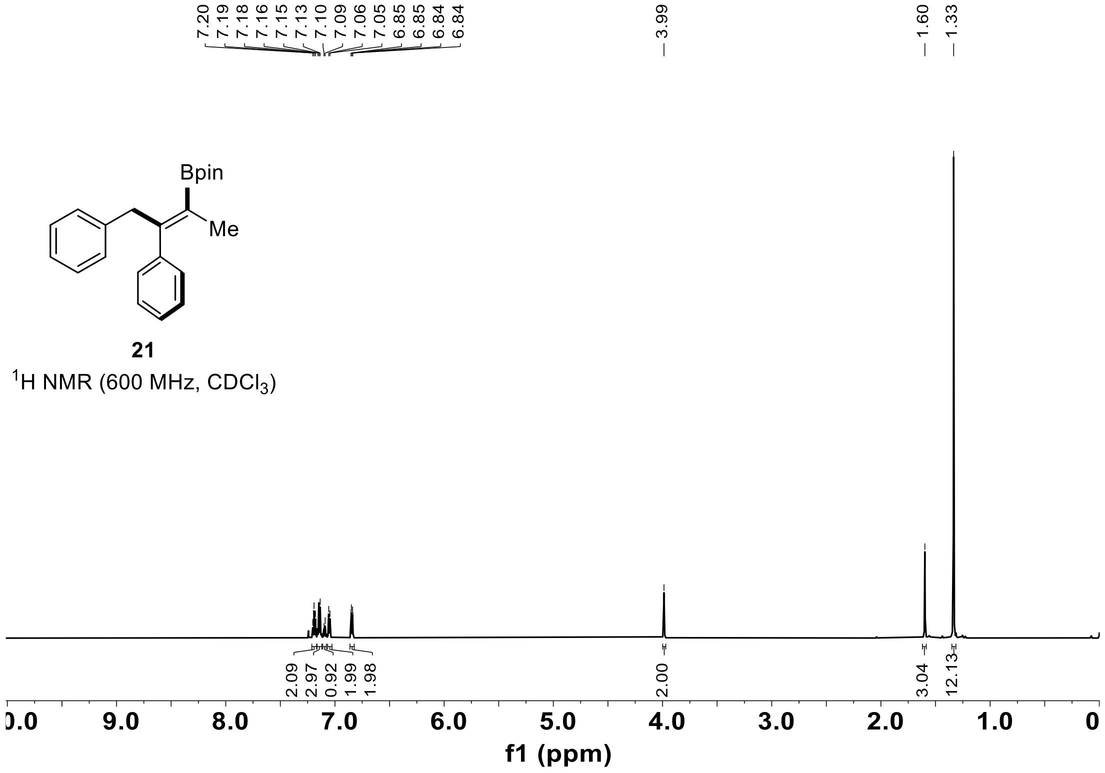

7.20 7.19 7.18 7.16 7.15 7.13 7.10 7.09 7.06 7.05 6.85 6.85 6.84 6.84
3.99
1.60 1.33
Bpin
Me
21
1H NMR (600 MHz, CDCl3)
2.09 2.97 0.92 1.99 1.98
2.00
3.04 12.13
9.0 8.0 7.0 6.0 5.0 4.0 3.0 2.0 1.0 0
f1 (ppm)

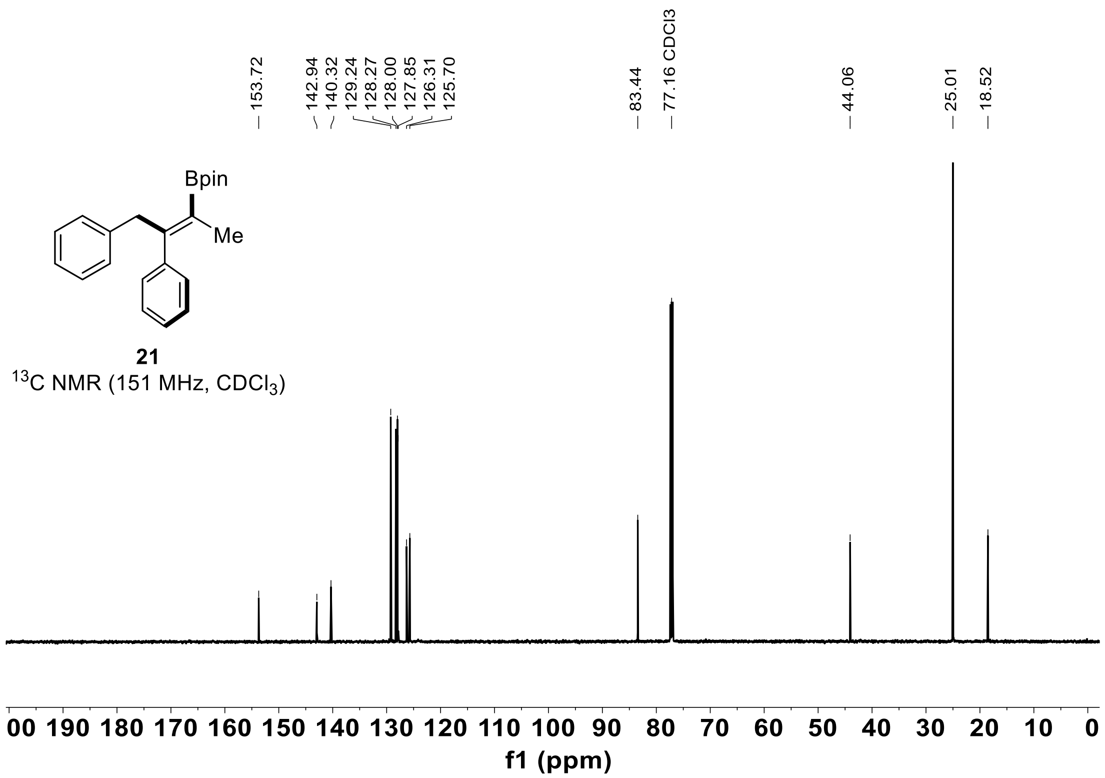

21
^{13}C NMR (151 MHz, $CDCl_3$)

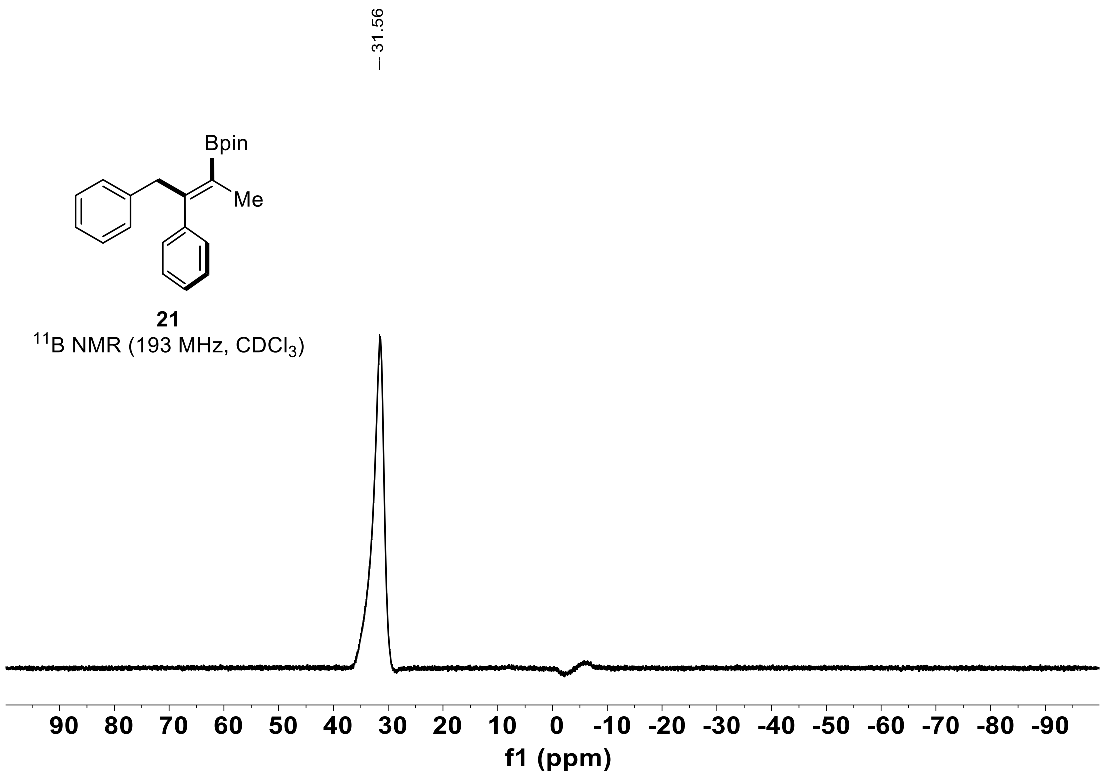

21
^{11}B NMR (193 MHz, $CDCl_3$)

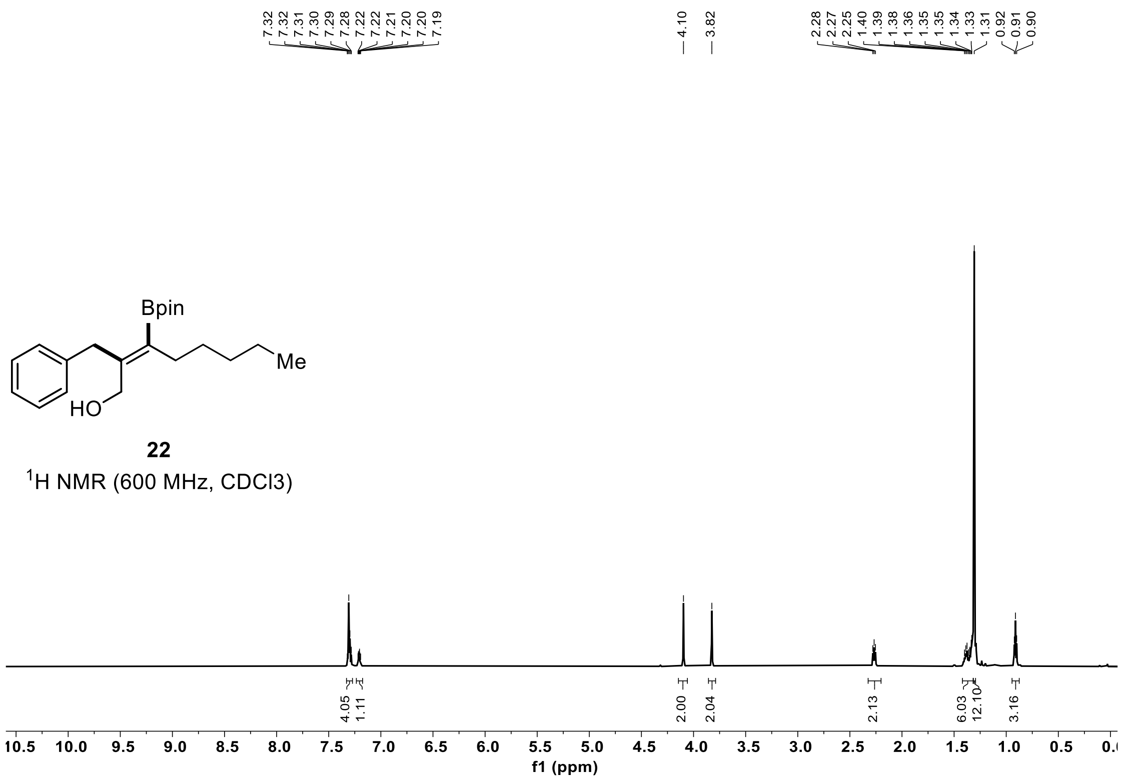

22

^{1}H NMR (600 MHz, CDCl3)

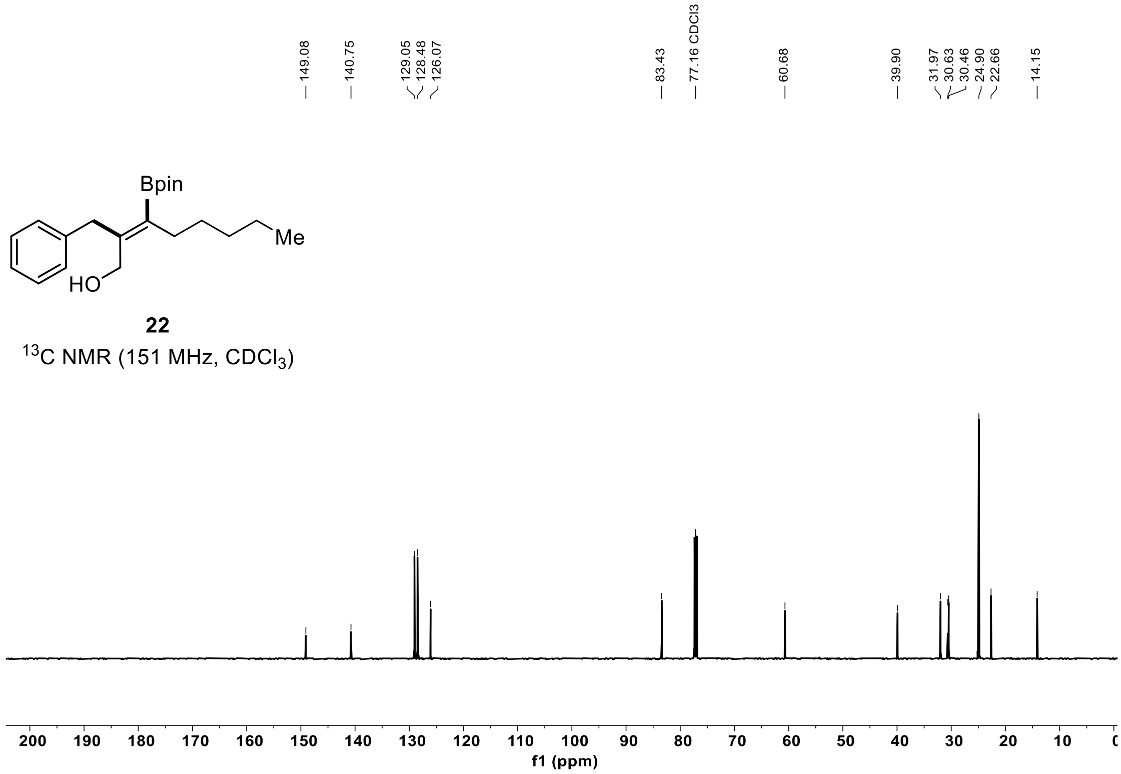

22

^{13}C NMR (151 MHz, $CDCl_3$)

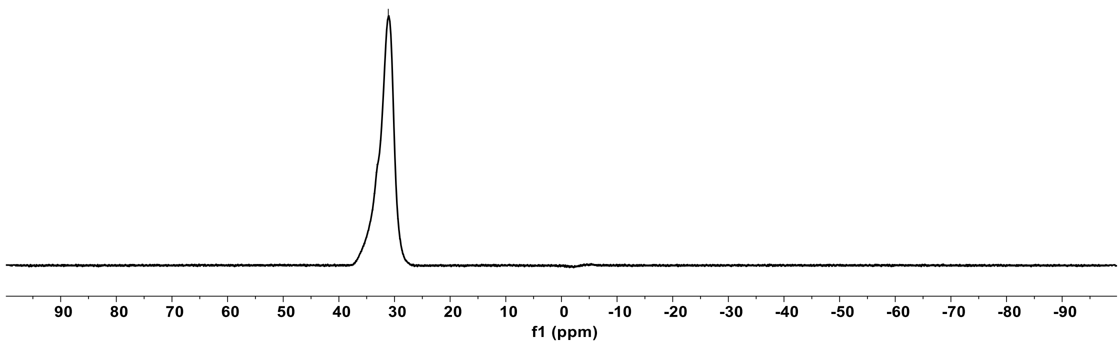

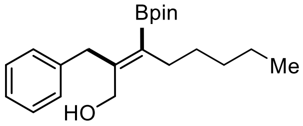

22

^{11}B NMR (193 MHz, $CDCl_3$)

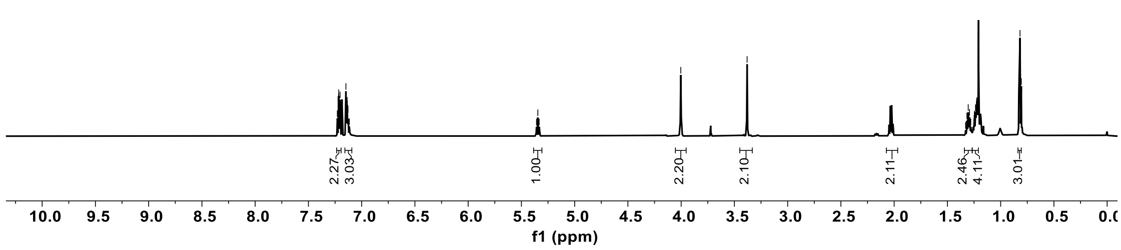

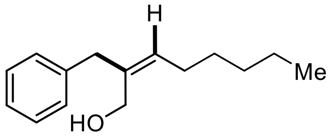

22'

^{1}H NMR (600 MHz, $CDCl_3$)

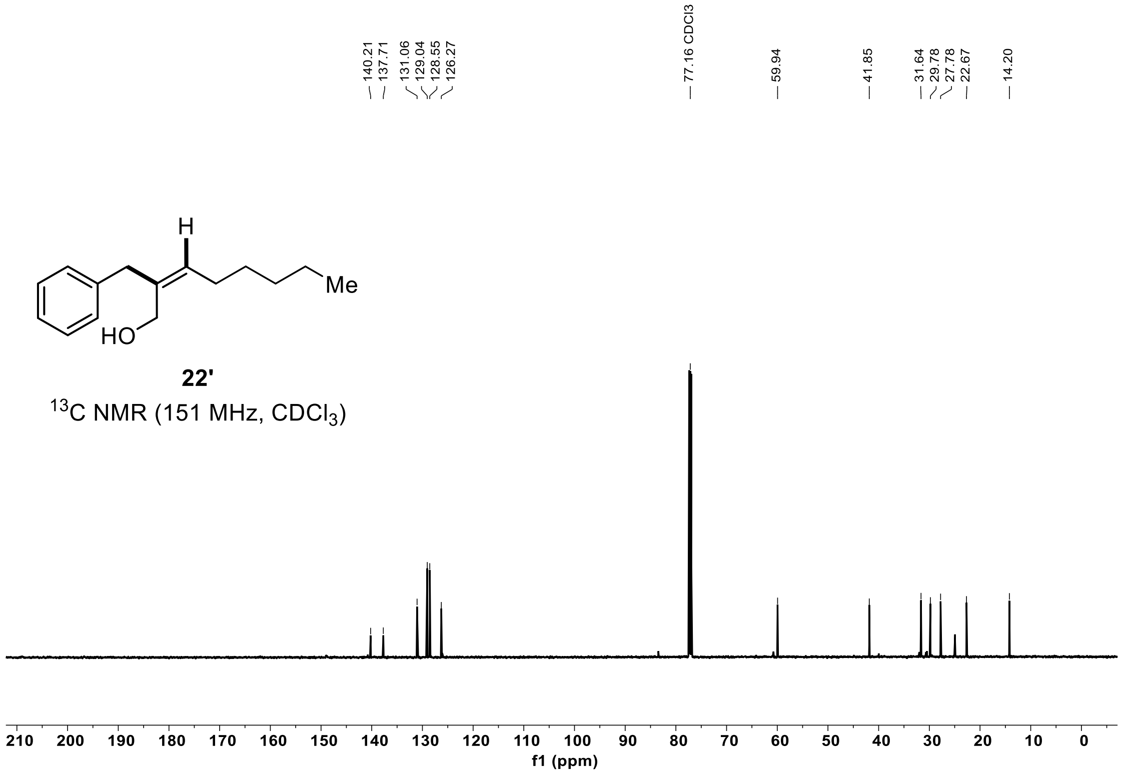

22'

^{13}C NMR (151 MHz, $CDCl_3$)

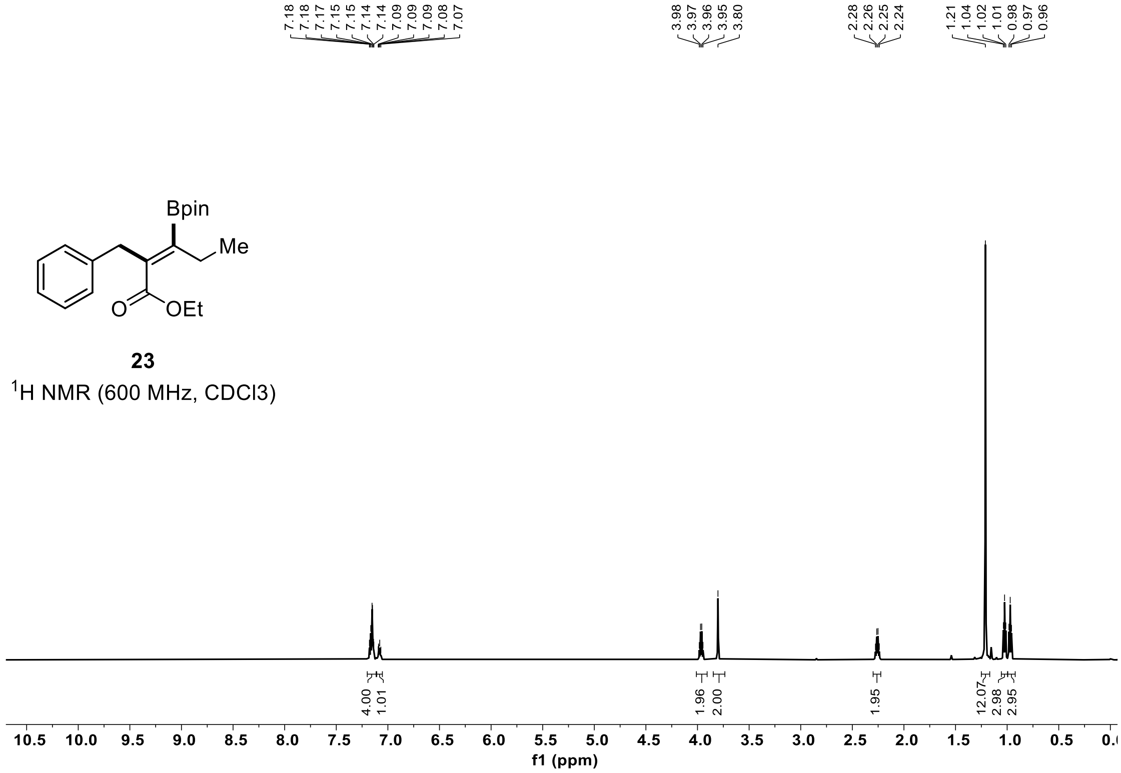

23

^{1}H NMR (600 MHz, CDCl3)

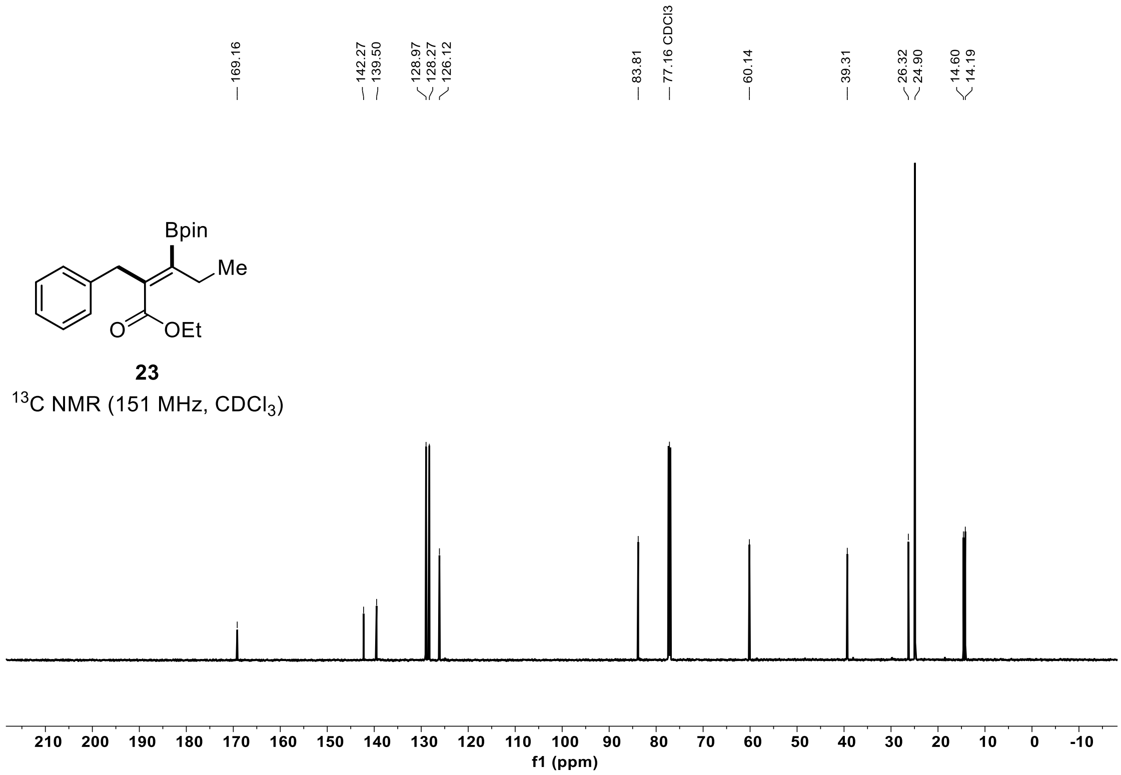
169.16
142.27
139.50
128.97
128.27
126.12
83.81
77.16 CDCl3
60.14
39.31
26.32
24.90
14.60
14.19
Bpin
Me
O
OEt
23
^{13}C NMR (151 MHz, $CDCl_3$)
210 200 190 180 170 160 150 140 130 120 110 100 90 80 70 60 50 40 30 20 10 0 -10
f1 (ppm)

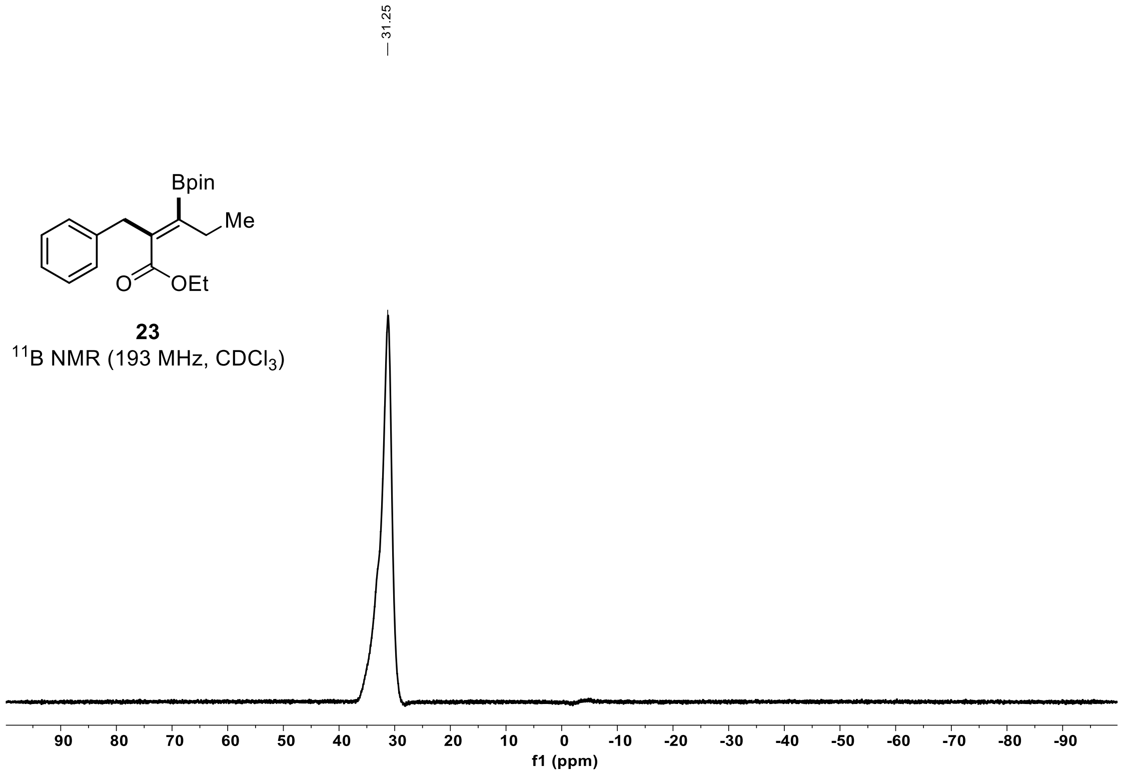
31.25
Bpin
Me
O
OEt
23
^{11}B NMR (193 MHz, $CDCl_3$)
90 80 70 60 50 40 30 20 10 0 -10 -20 -30 -40 -50 -60 -70 -80 -90
f1 (ppm)

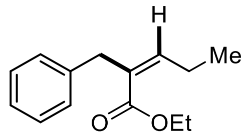

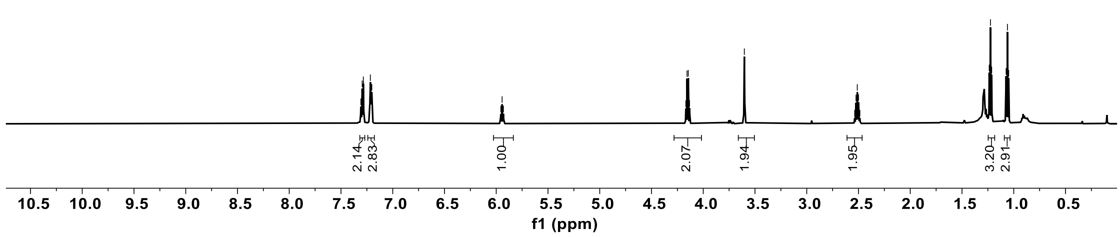

23'

^{1}H NMR (600 MHz, $CDCl_3$)

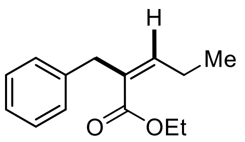

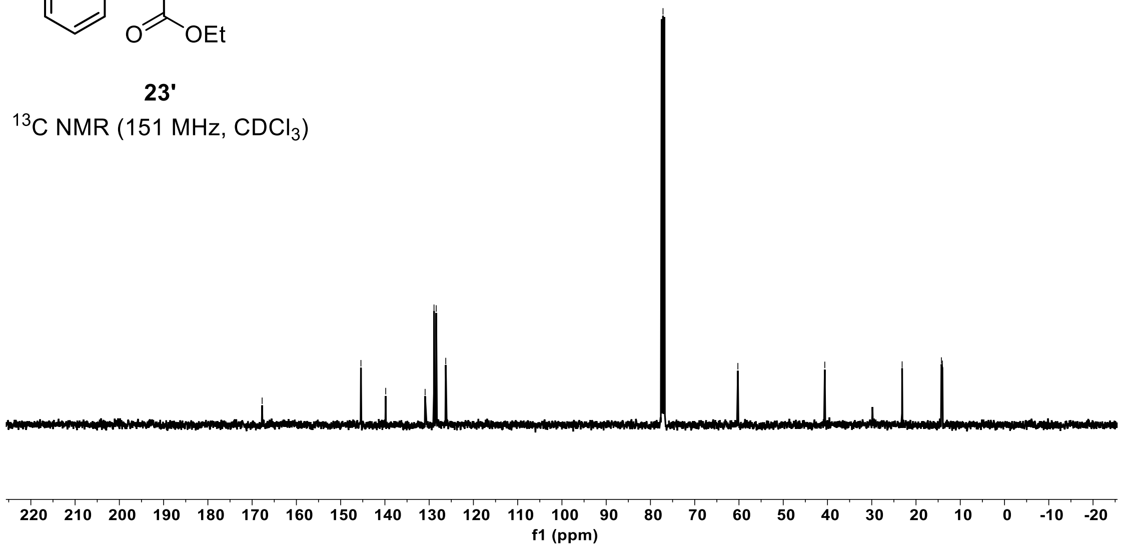

23'

^{13}C NMR (151 MHz, $CDCl_3$)

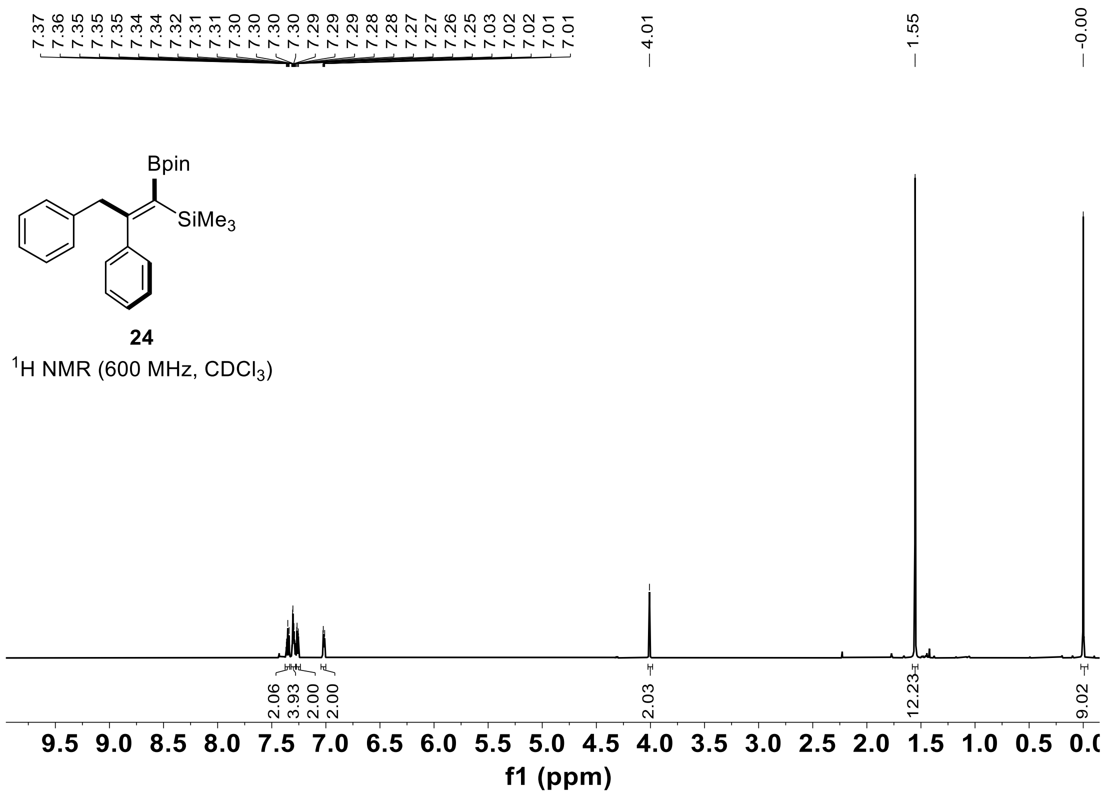
7.37 7.36 7.35 7.35 7.35 7.34 7.34 7.32 7.31 7.31 7.30 7.30 7.30 7.30 7.29 7.29 7.29 7.28 7.28 7.27 7.27 7.26 7.25 7.03 7.02 7.02 7.01 7.01
4.01
1.55
-0.00
Bpin
SiMe3
24
1H NMR (600 MHz, CDCl3)
2.06 3.93 2.00 2.00
2.03
12.23
9.02
9.5 9.0 8.5 8.0 7.5 7.0 6.5 6.0 5.5 5.0 4.5 4.0 3.5 3.0 2.5 2.0 1.5 1.0 0.5 0.0
f1 (ppm)

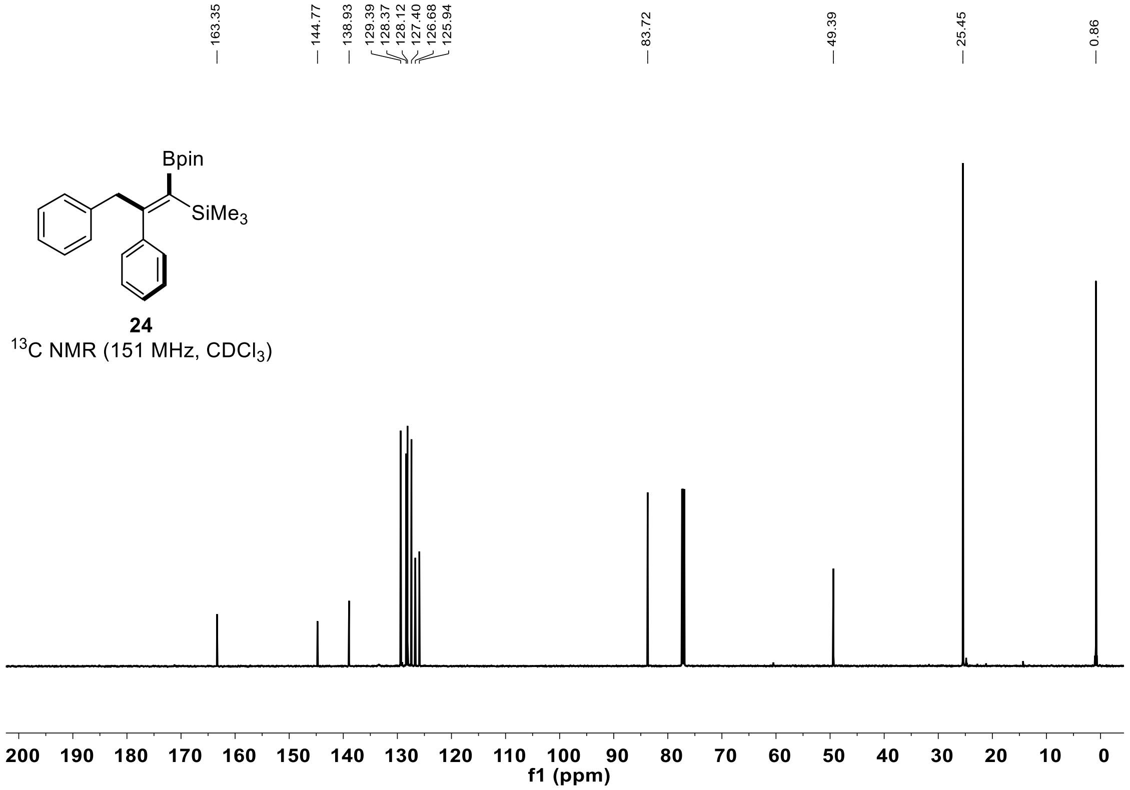
163.35
144.77
138.93
129.39 128.37 128.12 127.40 126.68 125.94
83.72
49.39
25.45
0.86
Bpin
SiMe3
24
13C NMR (151 MHz, CDCl3)
200 190 180 170 160 150 140 130 120 110 100 90 80 70 60 50 40 30 20 10 0
f1 (ppm)

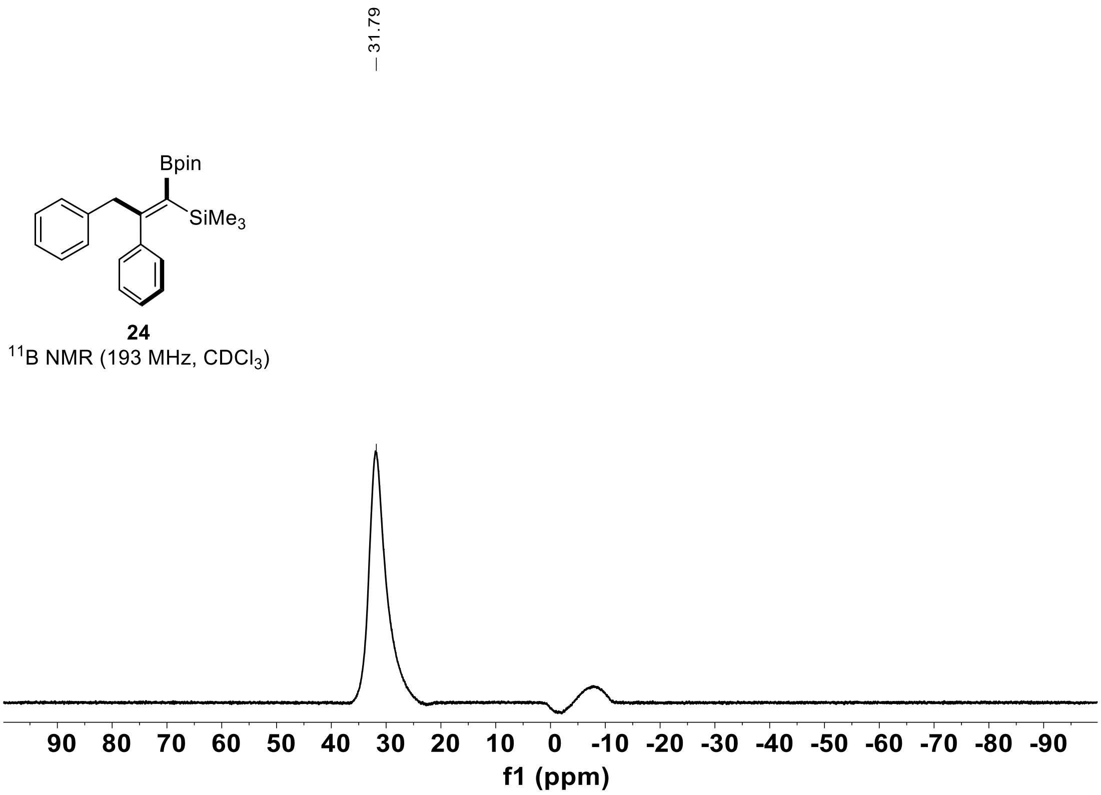
31.79
Bpin
SiMe3
24
11B NMR (193 MHz, CDCl3)
90 80 70 60 50 40 30 20 10 0 -10 -20 -30 -40 -50 -60 -70 -80 -90
f1 (ppm)

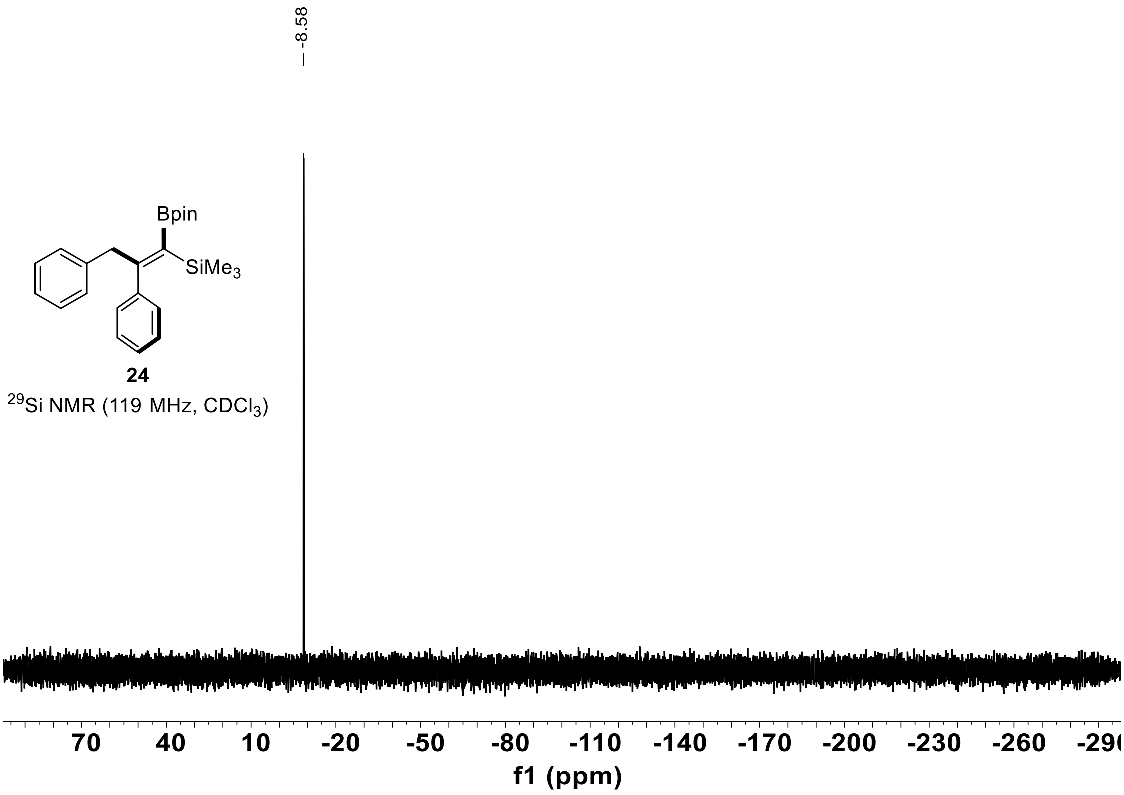
-8.58
Bpin
SiMe3
24
29Si NMR (119 MHz, CDCl3)
70 40 10 -20 -50 -80 -110 -140 -170 -200 -230 -260
f1 (ppm)

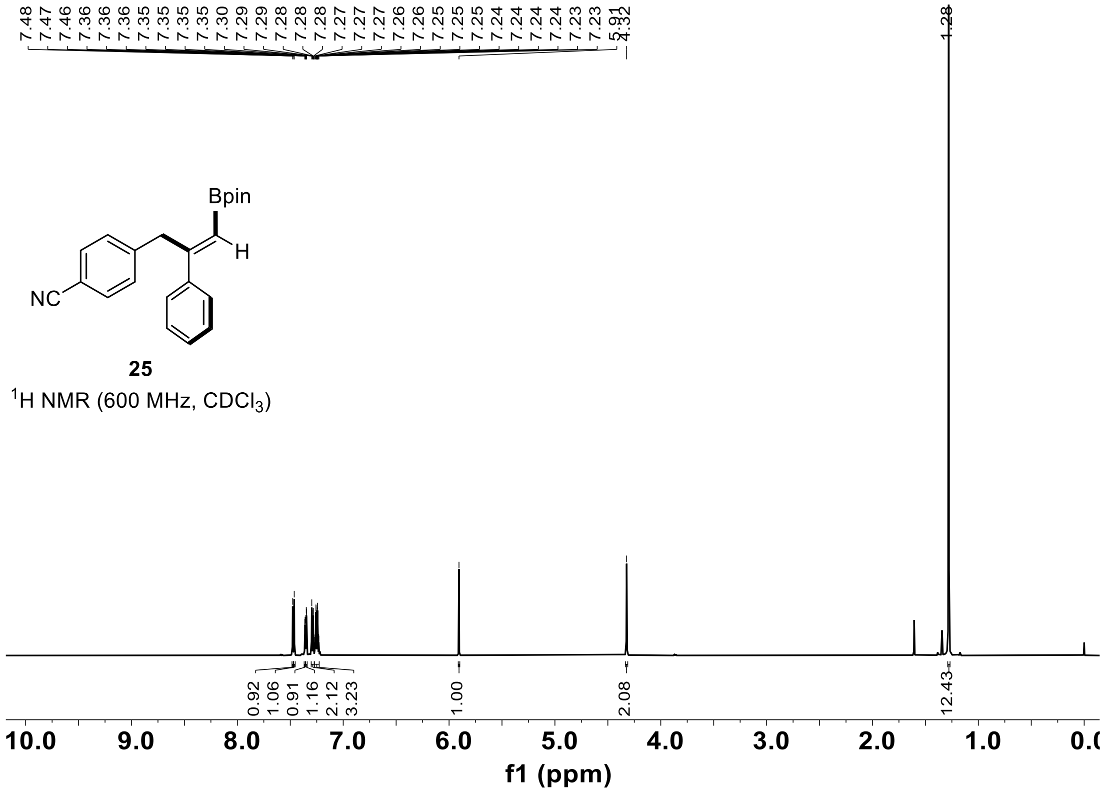
7.48 7.47 7.46 7.36 7.36 7.36 7.35 7.35 7.35 7.30 7.29 7.29 7.28 7.28 7.28 7.27 7.27 7.26 7.26 7.25 7.25 7.25 7.24 7.24 7.24 7.24 7.23 7.23 5.91 4.32 1.28
Bpin
H
NC
25
^{1}H NMR (600 MHz, $CDCl_3$)
0.92 1.06 0.91 1.16 2.12 3.23 1.00 2.08 12.43
10.0 9.0 8.0 7.0 6.0 5.0 4.0 3.0 2.0 1.0 0.0
f1 (ppm)

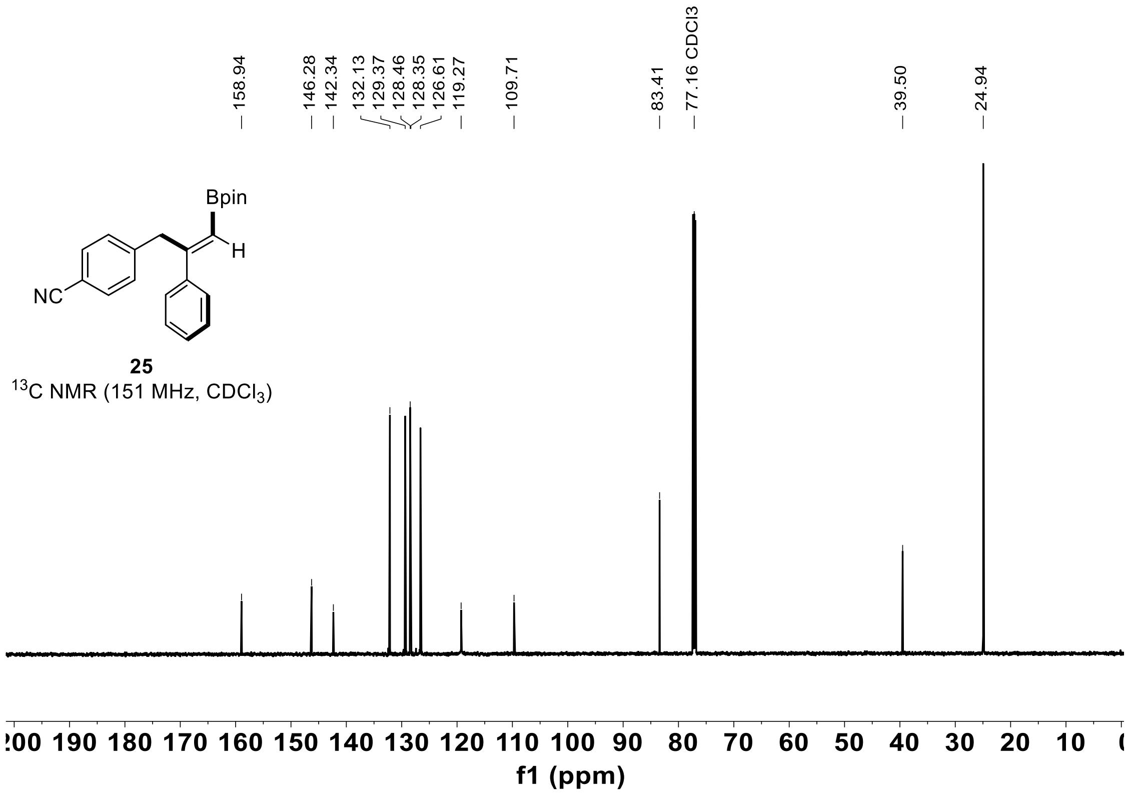
158.94 146.28 142.34 132.13 129.37 128.46 128.35 126.61 119.27 109.71 83.41 77.16 CDCl3 39.50 24.94
Bpin
H
NC
25
^{13}C NMR (151 MHz, $CDCl_3$)
200 190 180 170 160 150 140 130 120 110 100 90 80 70 60 50 40 30 20 10 0
f1 (ppm)

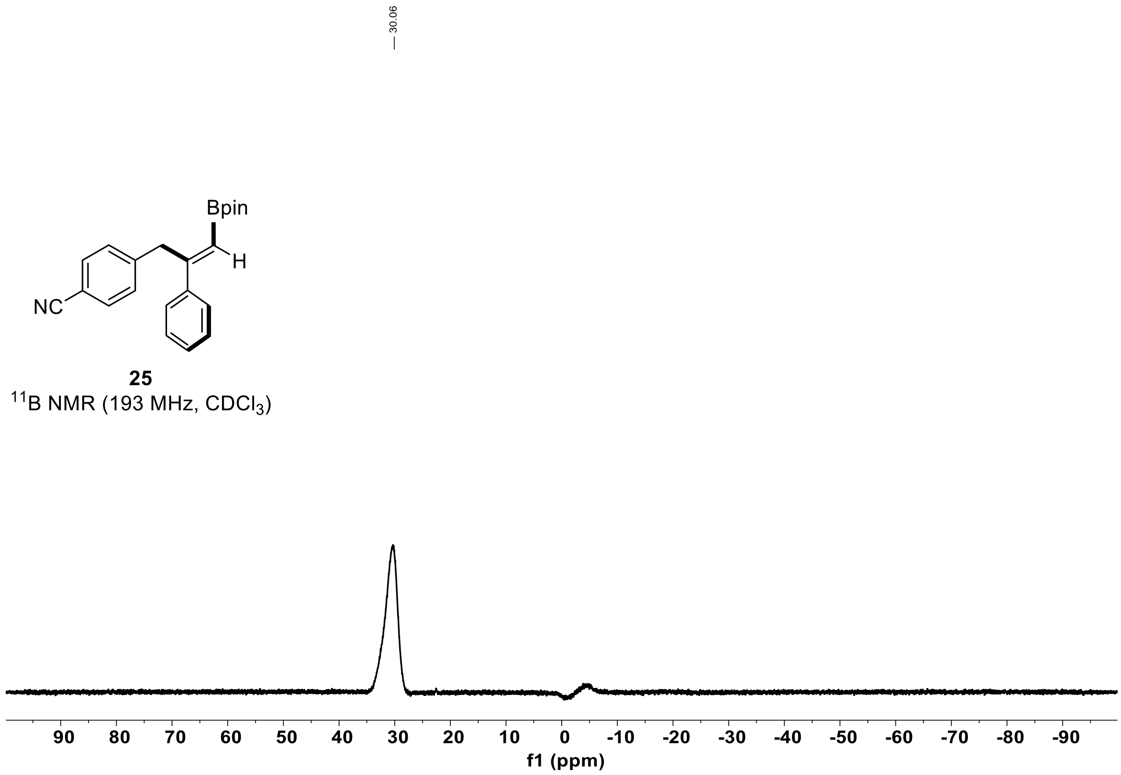
30.06
Bpin
H
NC
25
11B NMR (193 MHz, CDCl3)
90 80 70 60 50 40 30 20 10 0 -10 -20 -30 -40 -50 -60 -70 -80 -90
f1 (ppm)

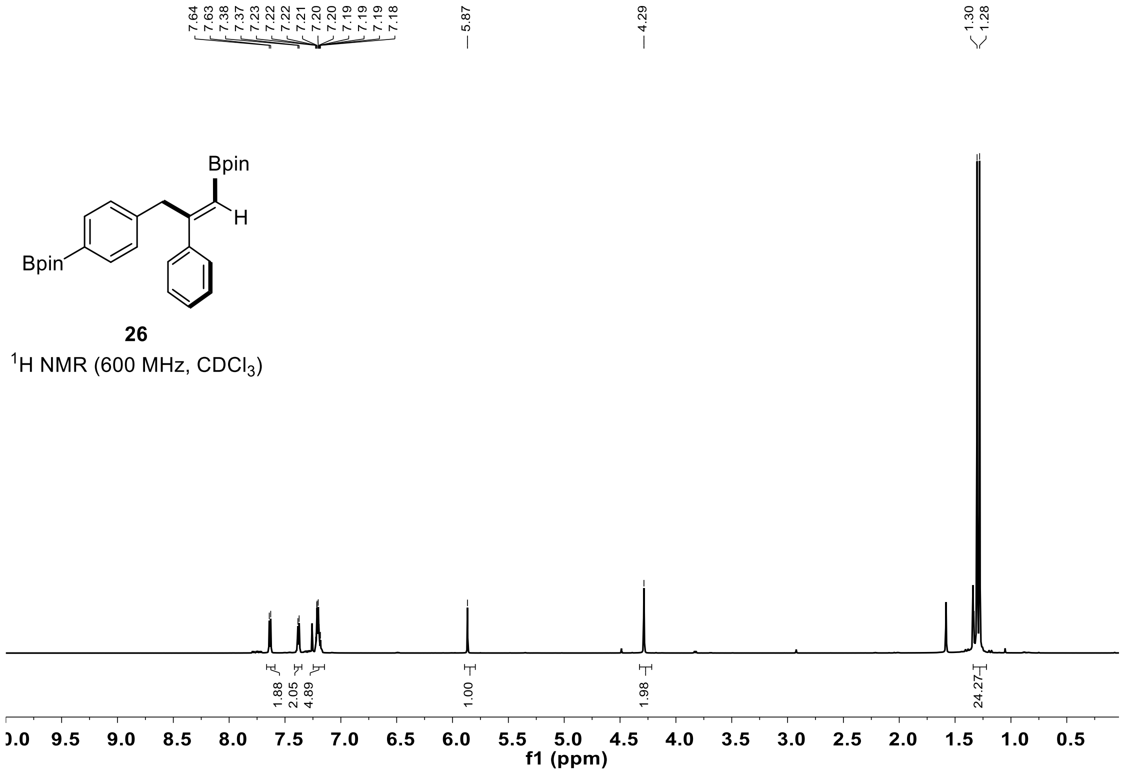
7.64 7.63 7.38 7.37 7.23 7.22 7.22 7.21 7.20 7.20 7.19 7.19 7.19 7.18
5.87
4.29
1.30 1.28
Bpin
H
Bpin
26
1H NMR (600 MHz, CDCl3)
1.88 2.05 4.89
1.00
1.98
24.27
9.5 9.0 8.5 8.0 7.5 7.0 6.5 6.0 5.5 5.0 4.5 4.0 3.5 3.0 2.5 2.0 1.5 1.0 0.5
f1 (ppm)

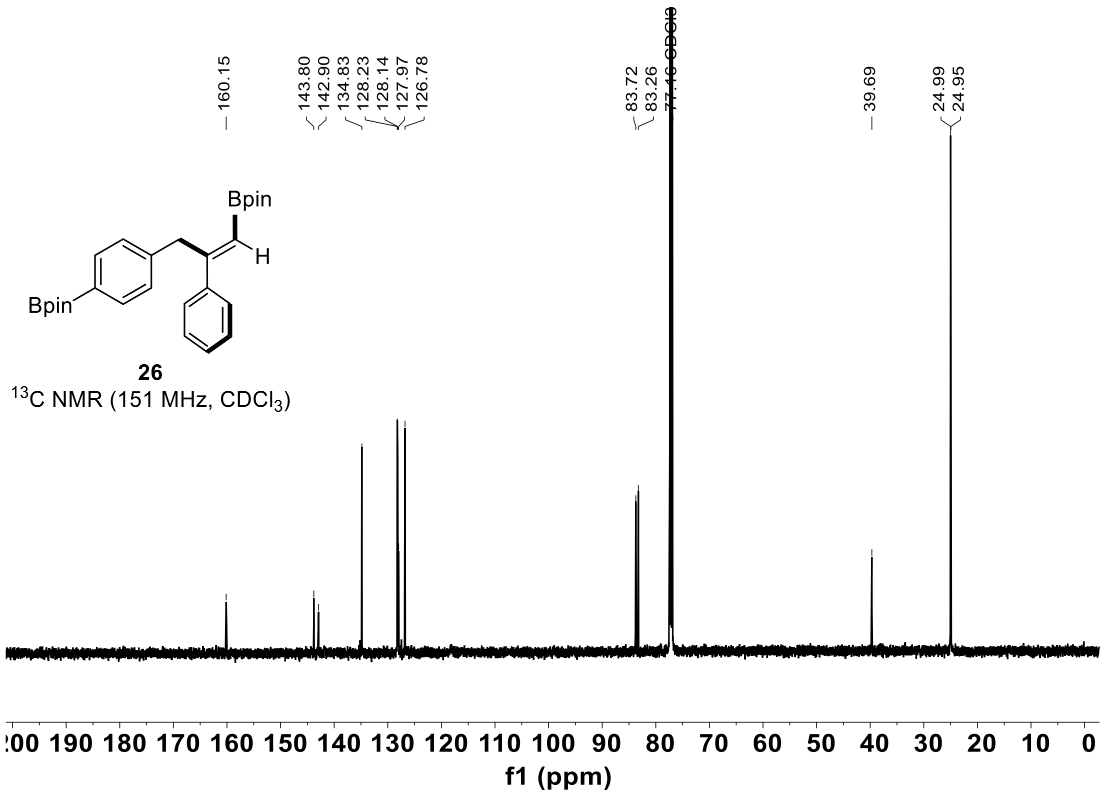
160.15
143.80
142.90
134.83
128.23
128.14
127.97
126.78
83.72
83.26
77.16 CDCl3
39.69
24.99
24.95
Bpin
H
Bpin
26
^{13}C NMR (151 MHz, CDCl$_3$)
200 190 180 170 160 150 140 130 120 110 100 90 80 70 60 50 40 30 20 10 0
f1 (ppm)

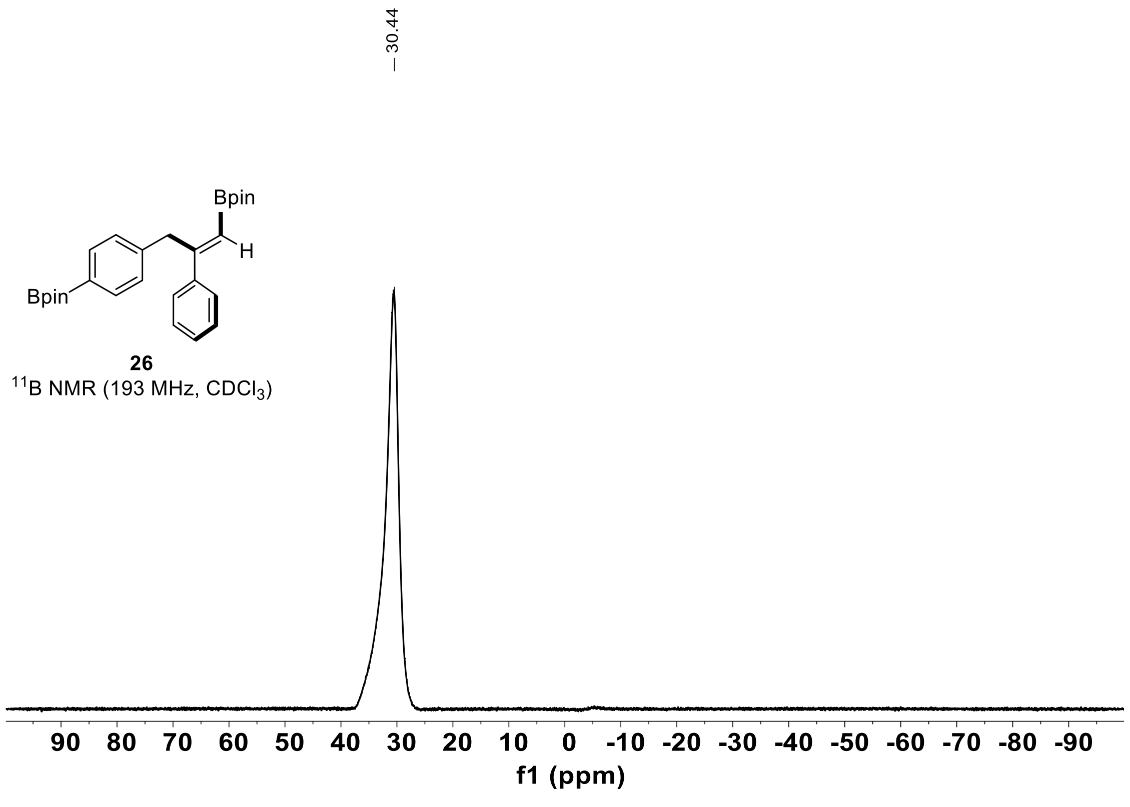
30.44
Bpin
H
Bpin
26
^{11}B NMR (193 MHz, CDCl$_3$)
90 80 70 60 50 40 30 20 10 0 -10 -20 -30 -40 -50 -60 -70 -80 -90
f1 (ppm)

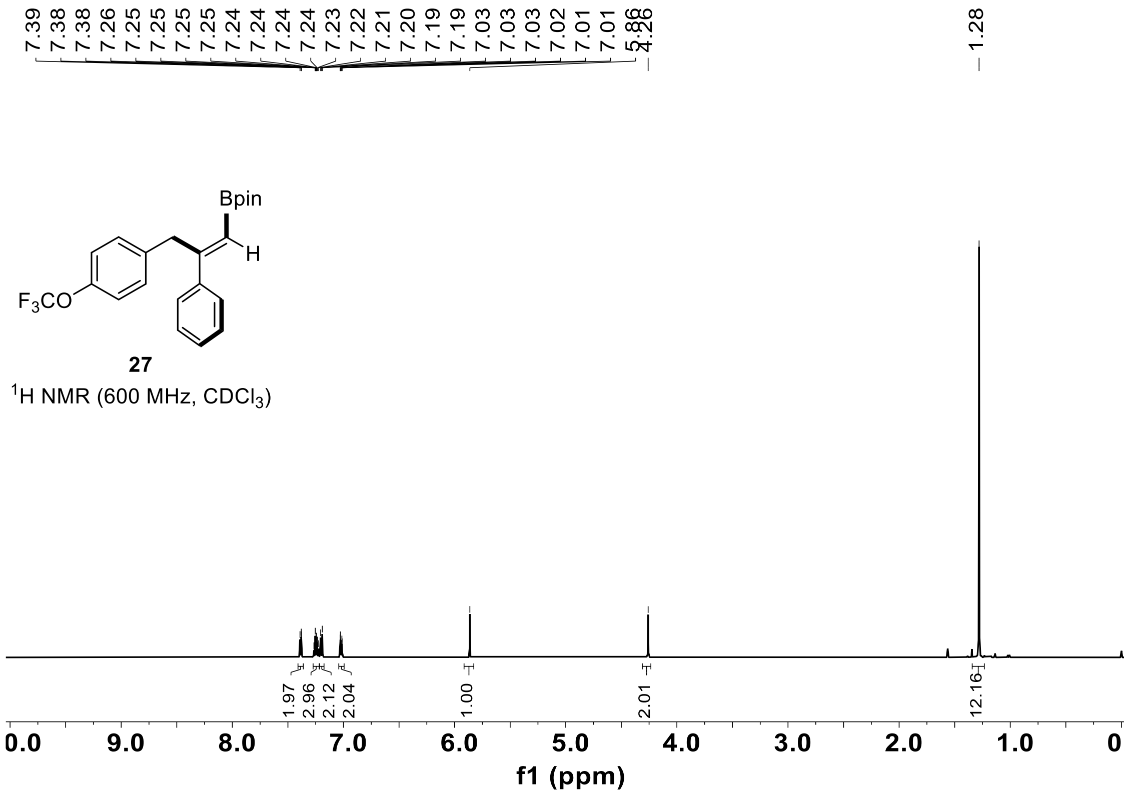
7.39 7.38 7.38 7.26 7.25 7.25 7.25 7.25 7.24 7.24 7.24 7.24 7.23 7.22 7.21 7.20 7.19 7.19 7.03 7.03 7.03 7.02 7.01 7.01 5.86 4.26
1.28
Bpin
H
F3CO
27
1H NMR (600 MHz, CDCl3)
1.97 2.96 2.12 2.04
1.00
2.01
12.16
0.0 9.0 8.0 7.0 6.0 5.0 4.0 3.0 2.0 1.0 0
f1 (ppm)

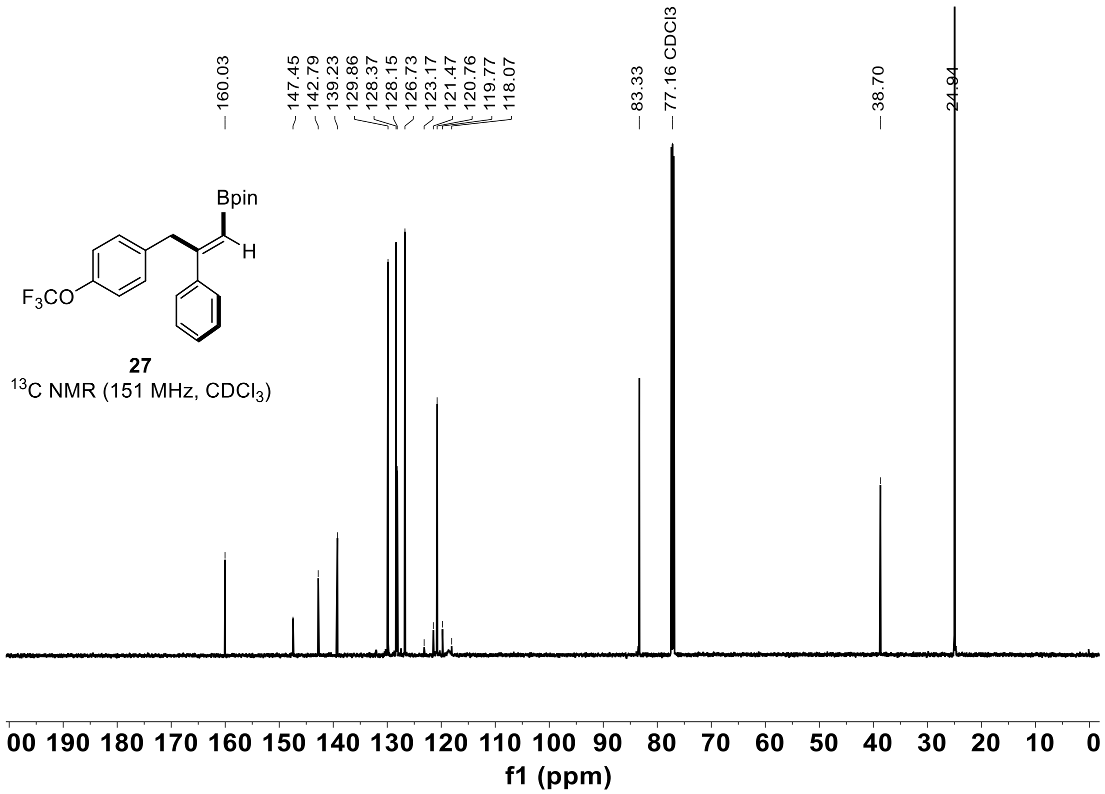
160.03
147.45 142.79 139.23 129.86 128.37 128.15 126.73 123.17 121.47 120.76 119.77 118.07
83.33
77.16 CDCl3
38.70
24.94
Bpin
H
F3CO
27
13C NMR (151 MHz, CDCl3)
00 190 180 170 160 150 140 130 120 110 100 90 80 70 60 50 40 30 20 10 0
f1 (ppm)

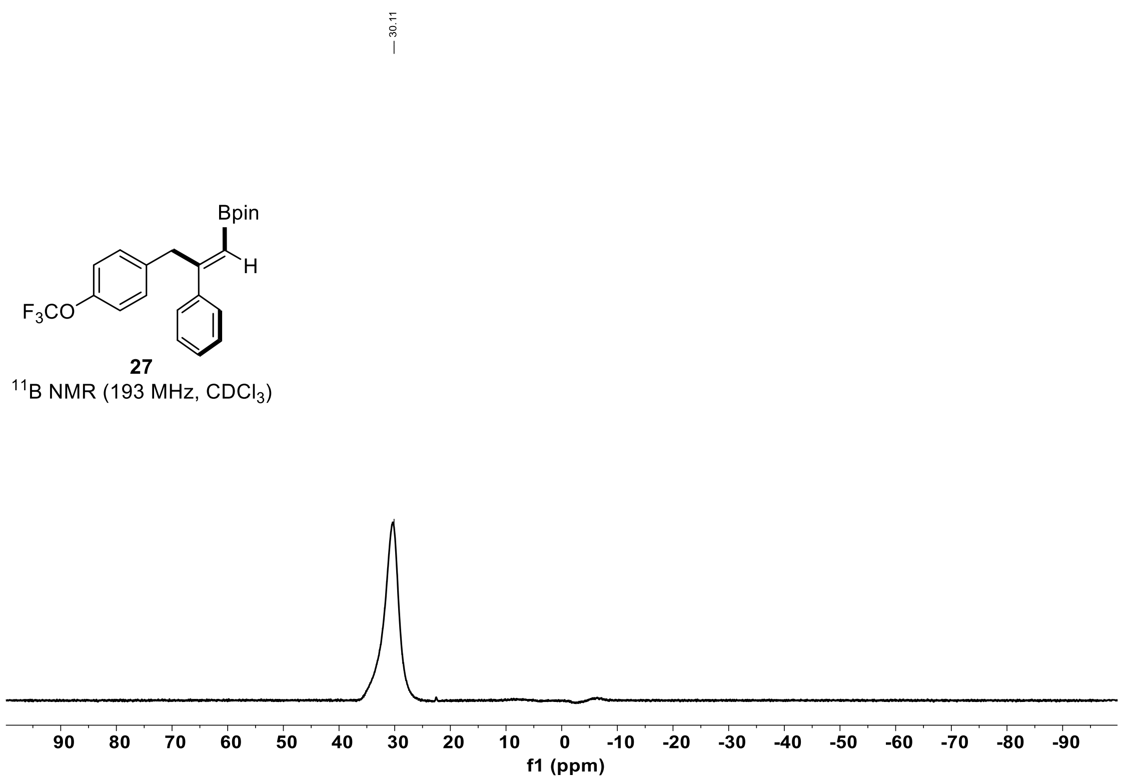
30.11
Bpin
H
F3CO
27
11B NMR (193 MHz, CDCl3)
90
80
70
60
50
40
30
20
10
0
-10
-20
-30
-40
-50
-60
-70
-80
-90
f1 (ppm)

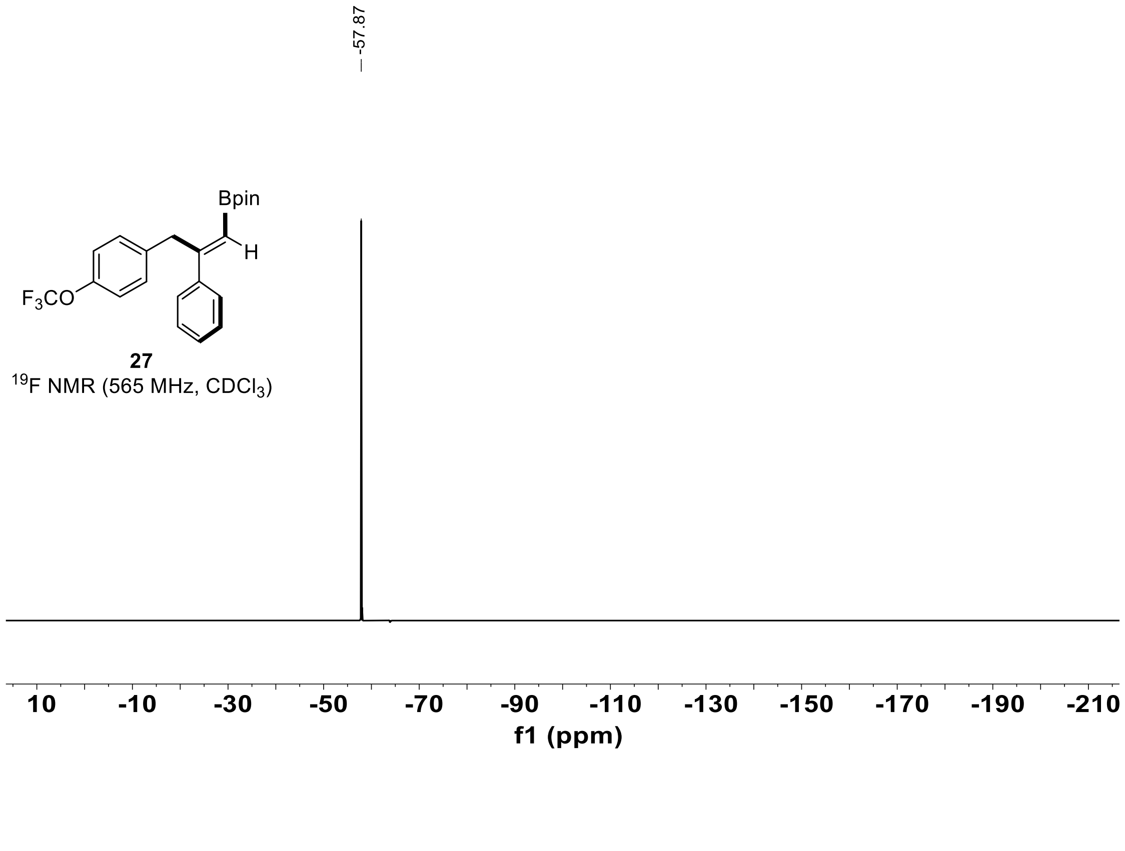
-57.87
Bpin
H
F3CO
27
19F NMR (565 MHz, CDCl3)
10
-10
-30
-50
-70
-90
-110
-130
-150
-170
-190
-210
f1 (ppm)

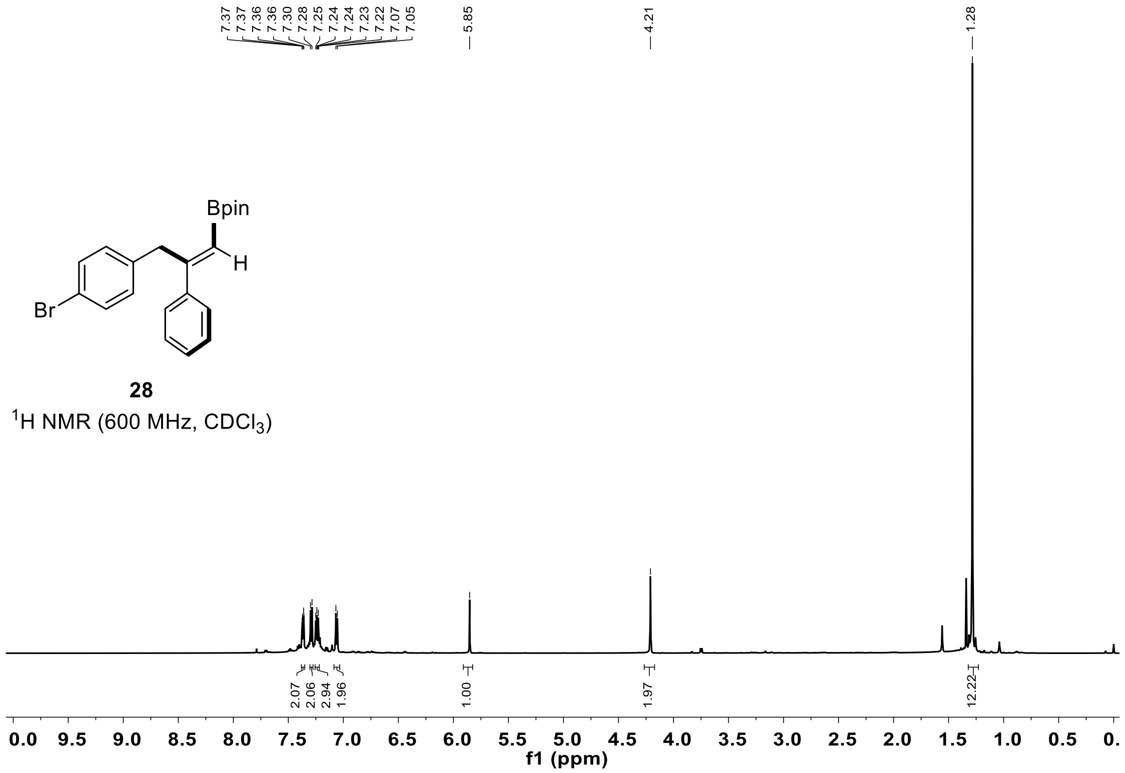
7.37 7.37 7.36 7.36 7.30 7.28 7.25 7.24 7.24 7.23 7.22 7.07 7.05
5.85
4.21
1.28
Bpin
H
Br
28
^{1}H NMR (600 MHz, $CDCl_3$)
2.07 2.06 2.94 1.96
1.00
1.97
12.22
0.0 9.5 9.0 8.5 8.0 7.5 7.0 6.5 6.0 5.5 5.0 4.5 4.0 3.5 3.0 2.5 2.0 1.5 1.0 0.5 0.
f1 (ppm)

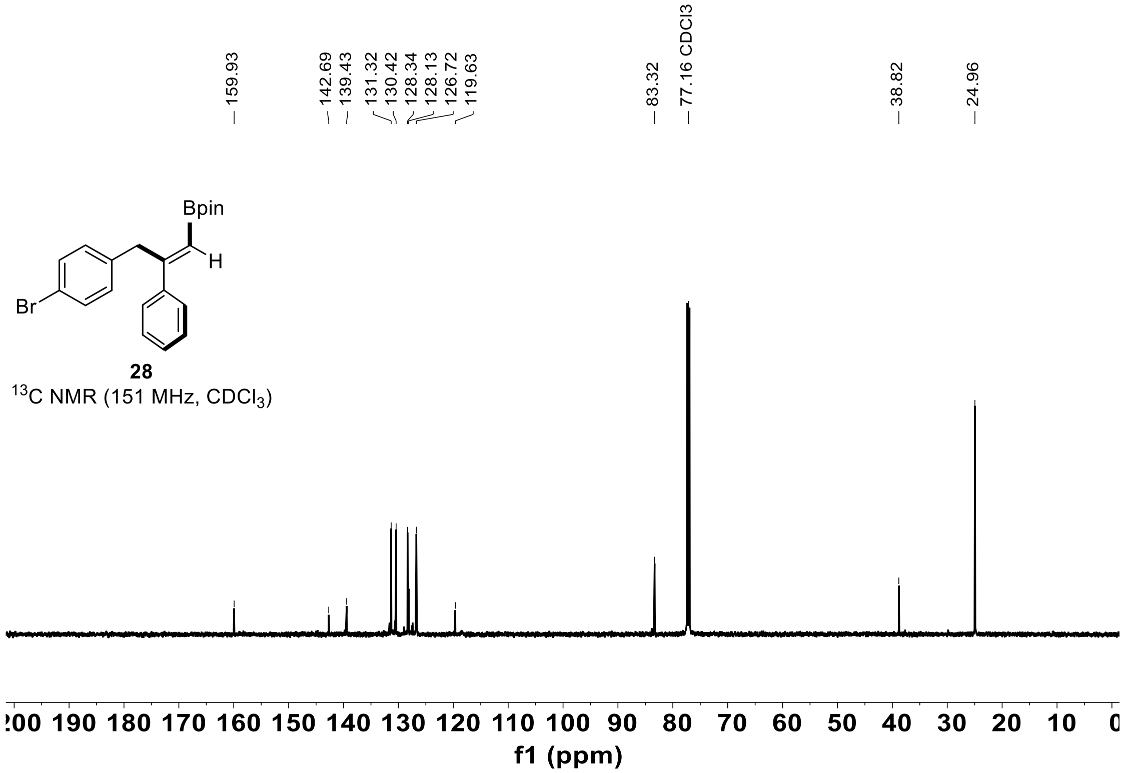
159.93
142.69 139.43 131.32 130.42 128.34 128.13 126.72 119.63
83.32
77.16 CDCl3
38.82
24.96
Bpin
H
Br
28
^{13}C NMR (151 MHz, $CDCl_3$)
200 190 180 170 160 150 140 130 120 110 100 90 80 70 60 50 40 30 20 10 0
f1 (ppm)

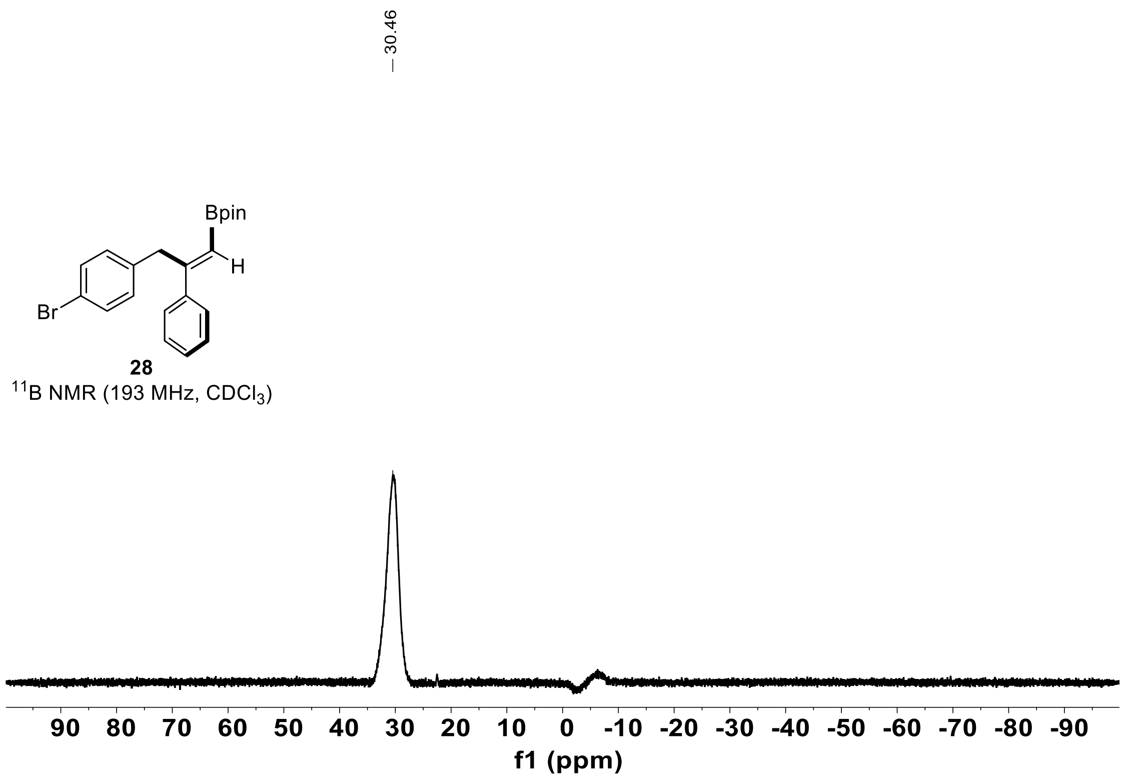

28
^{11}B NMR (193 MHz, $CDCl_3$)

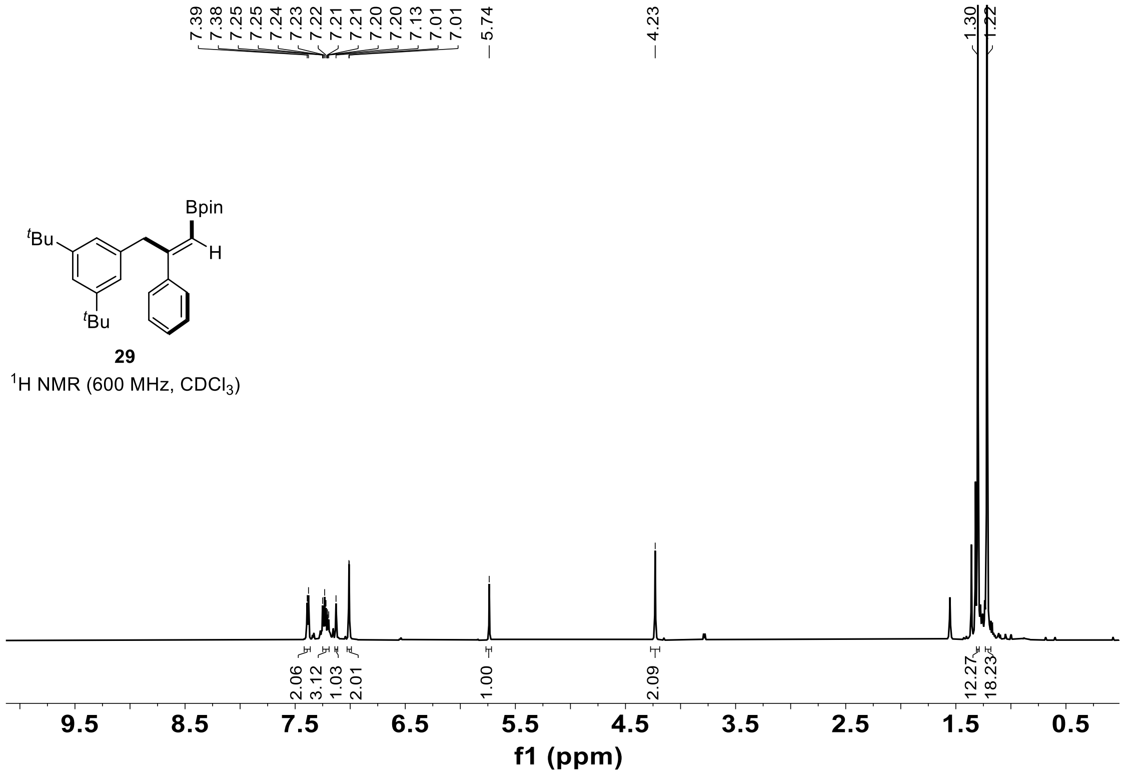

29
^{1}H NMR (600 MHz, $CDCl_3$)

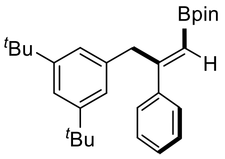

29

^{13}C NMR (151 MHz, $CDCl_3$)

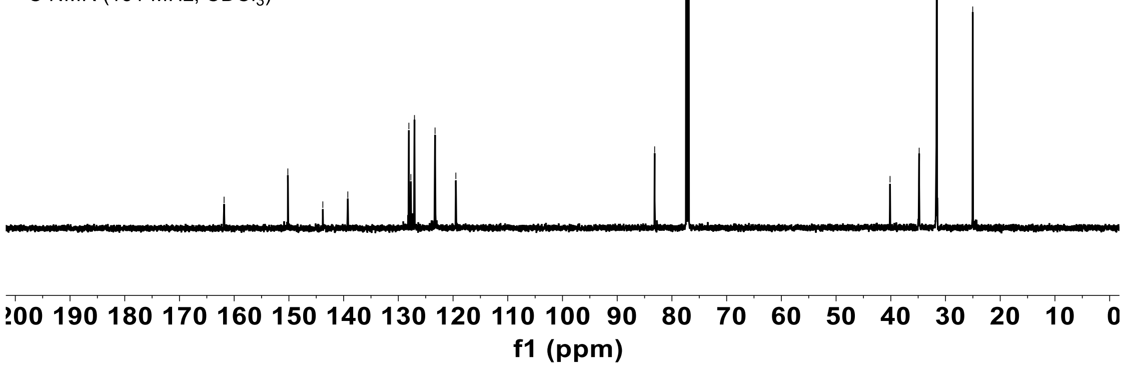

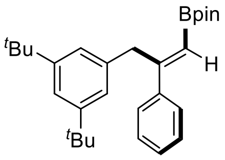

29

^{11}B NMR (193 MHz, $CDCl_3$)

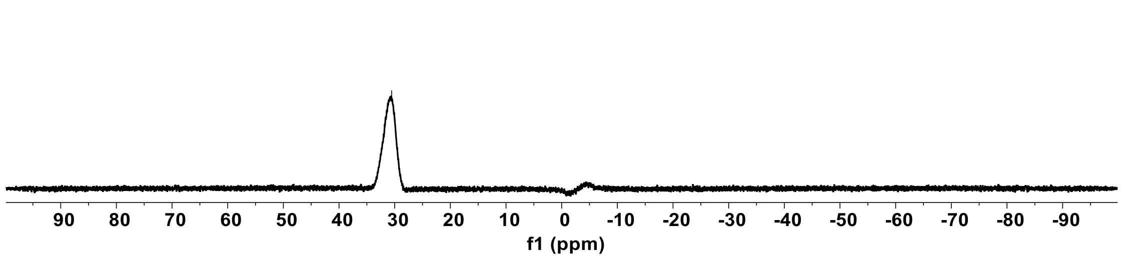

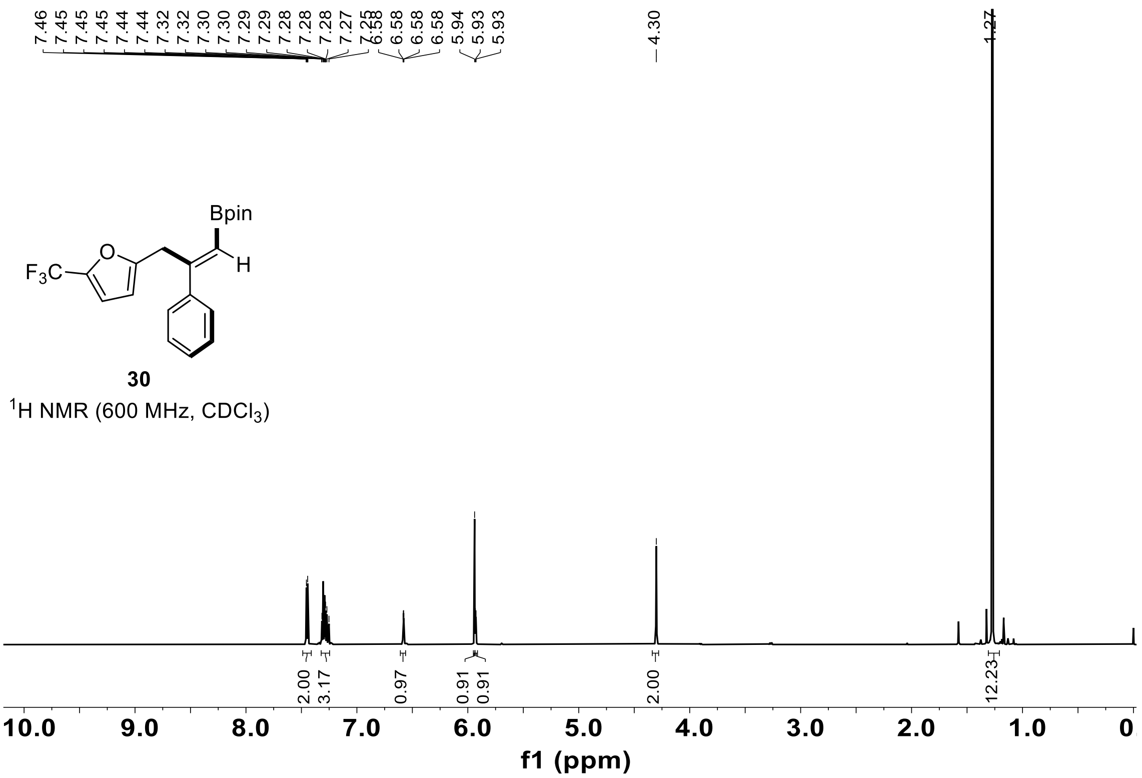

30

^{1}H NMR (600 MHz, $CDCl_3$)

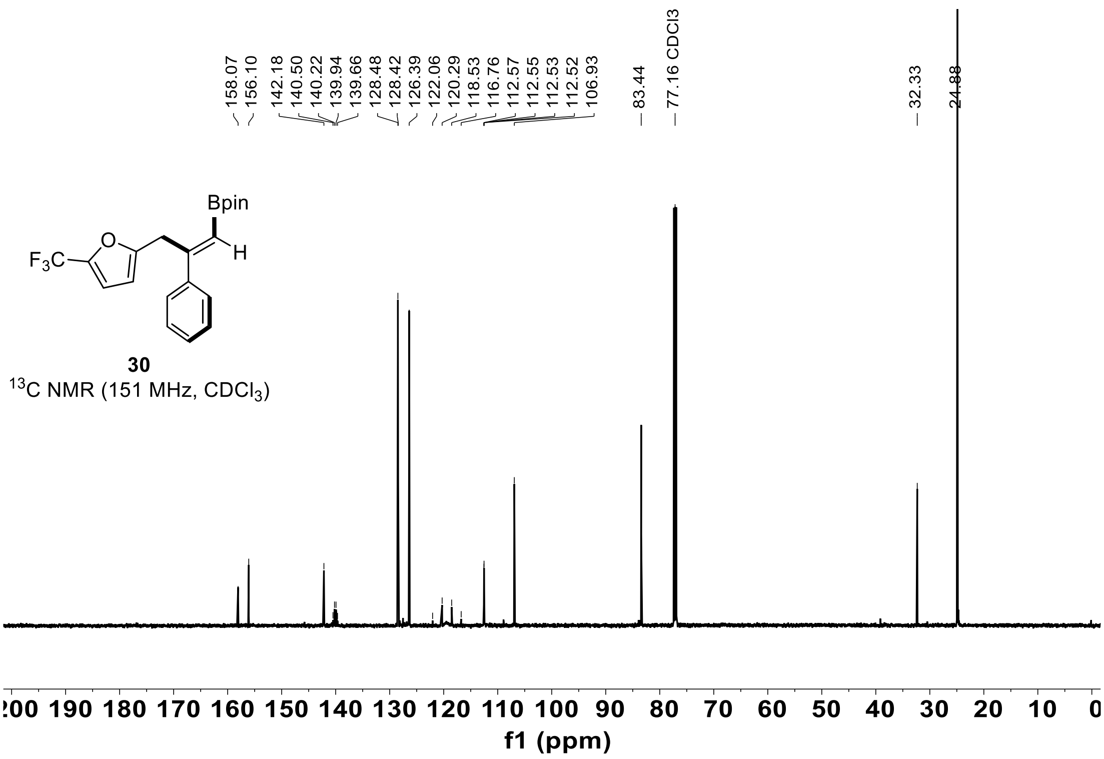

30

^{13}C NMR (151 MHz, $CDCl_3$)

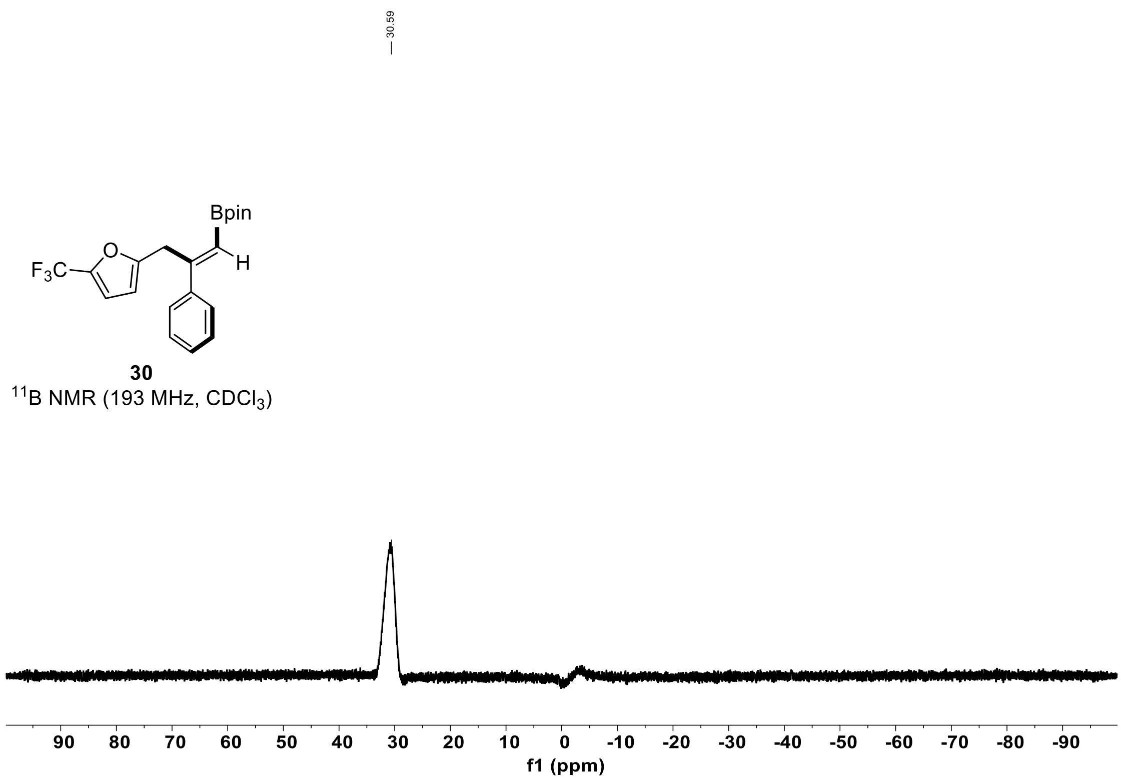
30.59
Bpin
F_3C
O
H
30
^{11}B NMR (193 MHz, $CDCl_3$)
90 80 70 60 50 40 30 20 10 0 -10 -20 -30 -40 -50 -60 -70 -80 -90
f1 (ppm)

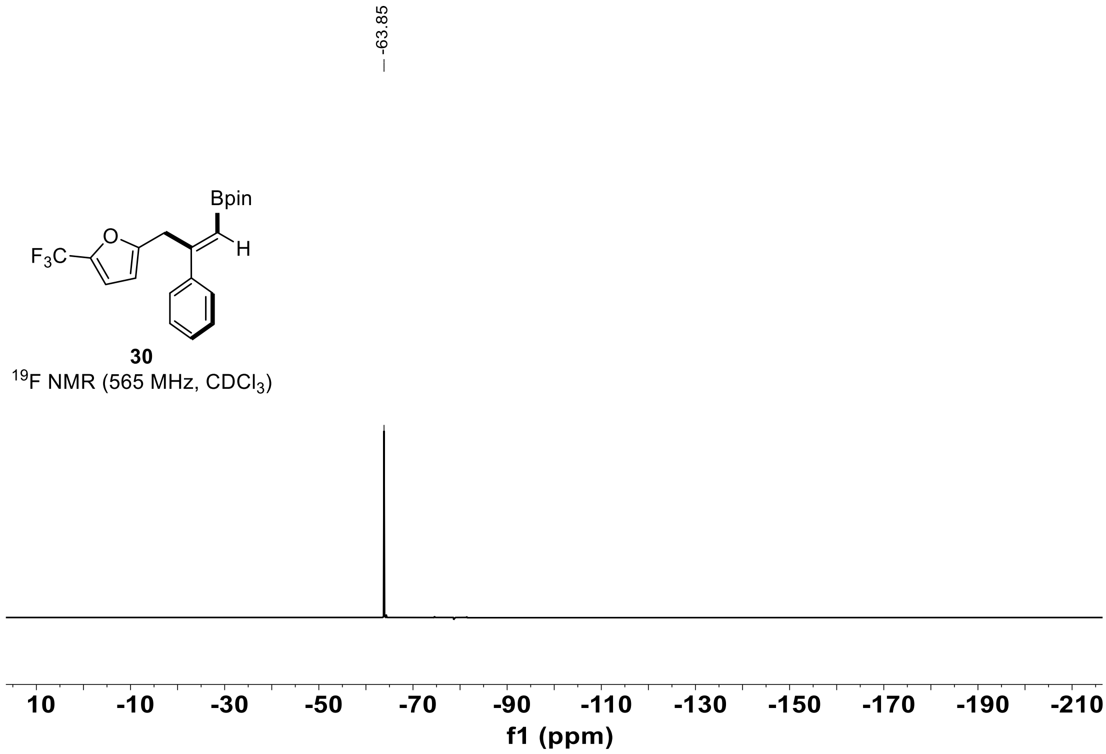
-63.85
Bpin
F_3C
O
H
30
^{19}F NMR (565 MHz, $CDCl_3$)
10 -10 -30 -50 -70 -90 -110 -130 -150 -170 -190 -210
f1 (ppm)

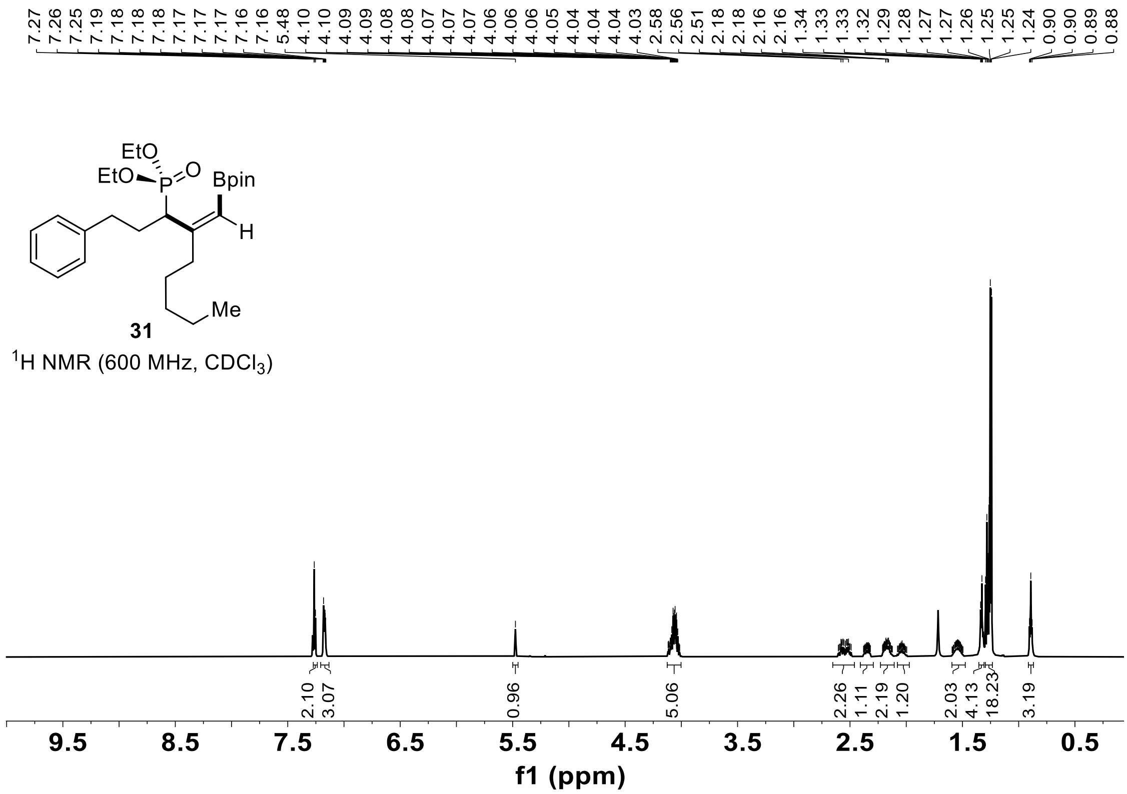
7.27 7.26 7.25 7.19 7.18 7.18 7.18 7.17 7.17 7.17 7.16 7.16 5.48 4.10 4.10 4.09 4.09 4.08 4.08 4.07 4.07 4.07 4.06 4.06 4.06 4.05 4.04 4.04 4.04 4.03 2.58 2.56 2.51 2.18 2.18 2.16 2.16 1.34 1.33 1.33 1.32 1.29 1.28 1.27 1.27 1.26 1.25 1.25 1.24 0.90 0.89 0.88
EtO
EtO
P
O
Bpin
H
Me
31
^{1}H NMR (600 MHz, CDCl$_3$)
2.10 3.07 0.96 5.06 2.26 1.11 2.19 1.20 2.03 4.13 18.23 3.19
9.5 8.5 7.5 6.5 5.5 4.5 3.5 2.5 1.5 0.5
f1 (ppm)

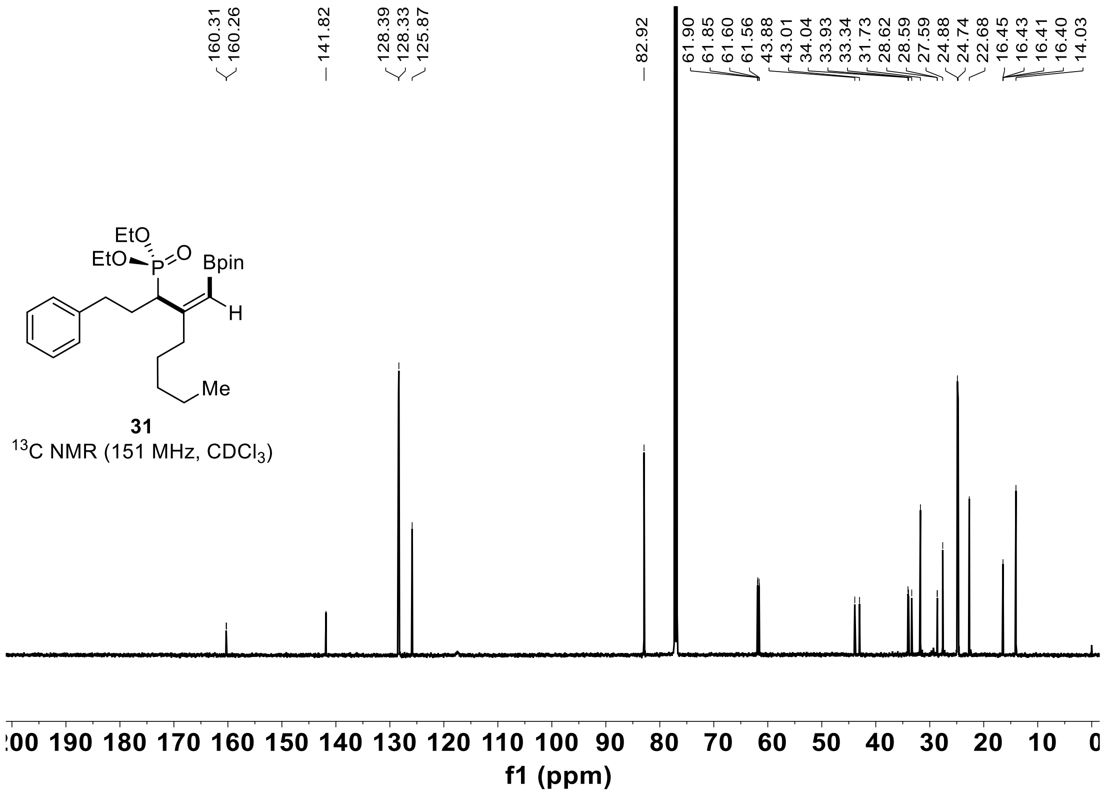
160.31 160.26 141.82 128.39 128.33 125.87 82.92 61.90 61.85 61.60 61.56 43.88 43.01 34.04 33.93 33.34 31.73 28.62 28.59 27.59 24.88 24.74 22.68 16.45 16.43 16.41 16.40 14.03
EtO
EtO
P
O
Bpin
H
Me
31
^{13}C NMR (151 MHz, CDCl$_3$)
200 190 180 170 160 150 140 130 120 110 100 90 80 70 60 50 40 30 20 10 0
f1 (ppm)

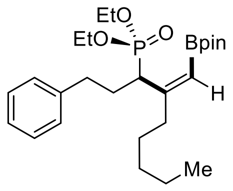

31

^{11}B NMR (193 MHz, $CDCl_3$)

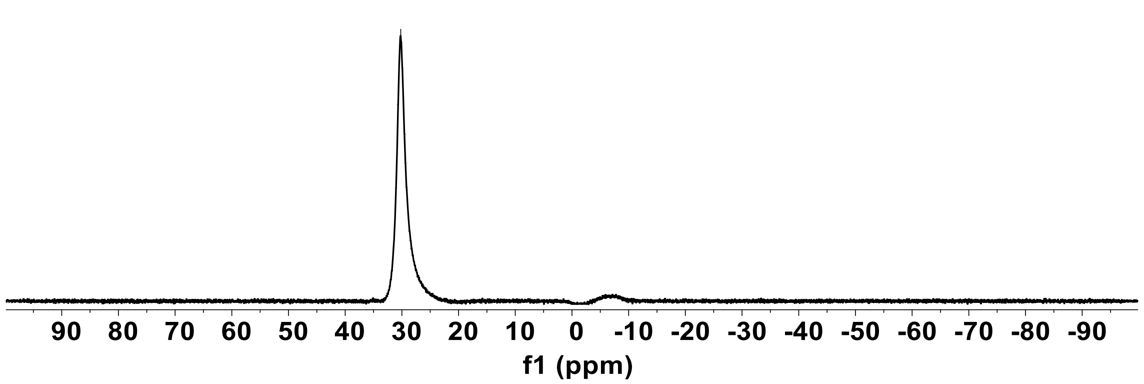

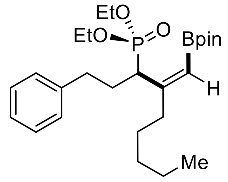

31

^{31}P NMR (243 MHz, $CDCl_3$)

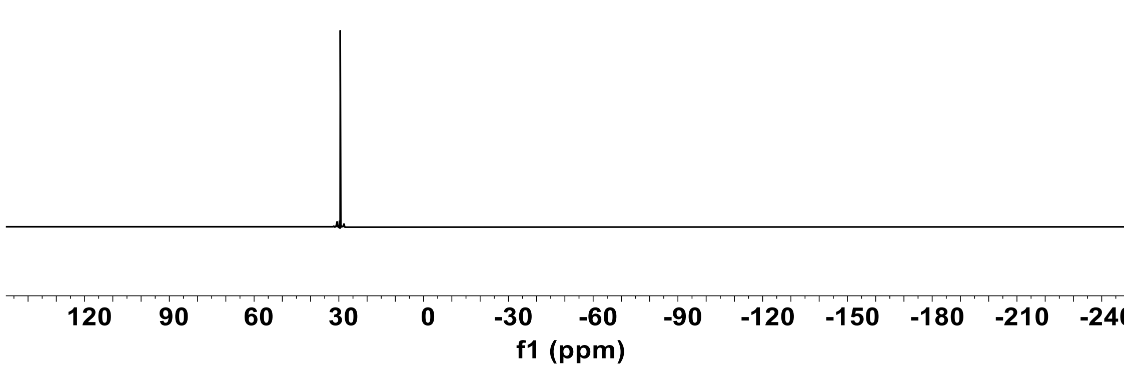

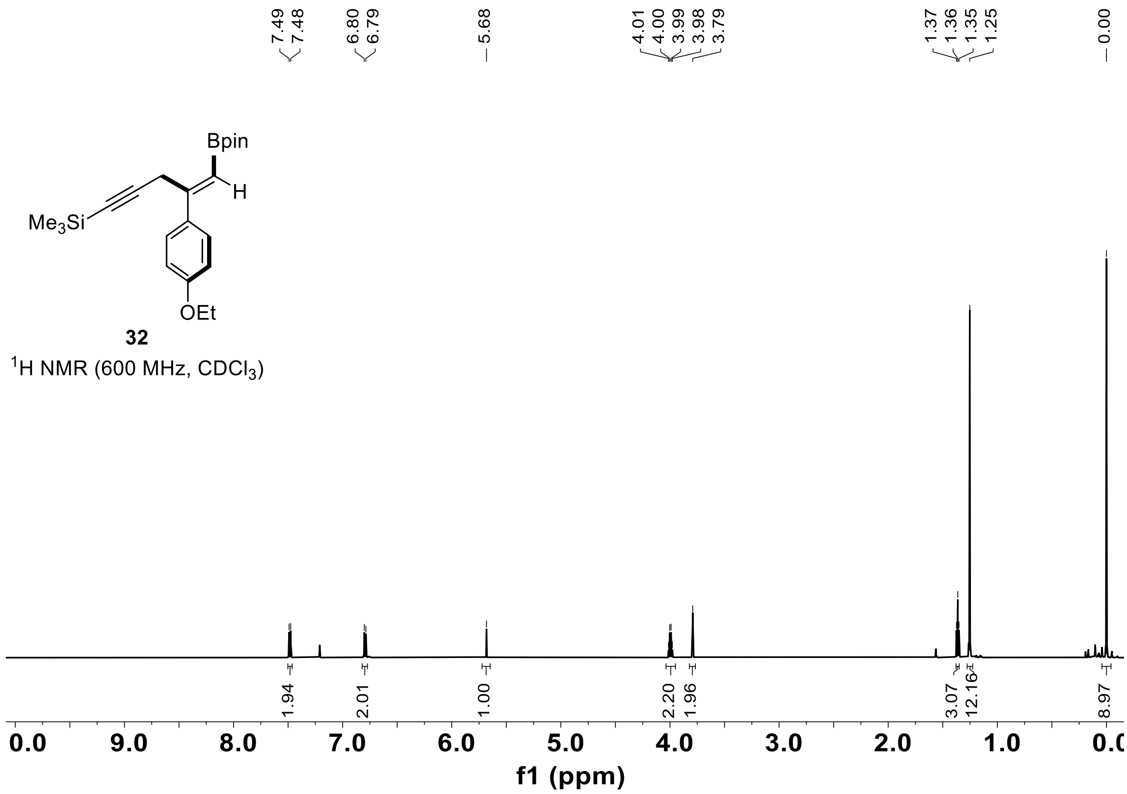
7.49
7.48
6.80
6.79
5.68
4.01
4.00
3.99
3.98
3.79
1.37
1.36
1.35
1.25
0.00
Bpin
H
Me_3Si
OEt
32
^{1}H NMR (600 MHz, $CDCl_3$)
1.94
2.01
1.00
2.20
1.96
3.07
12.16
8.97
f1 (ppm)

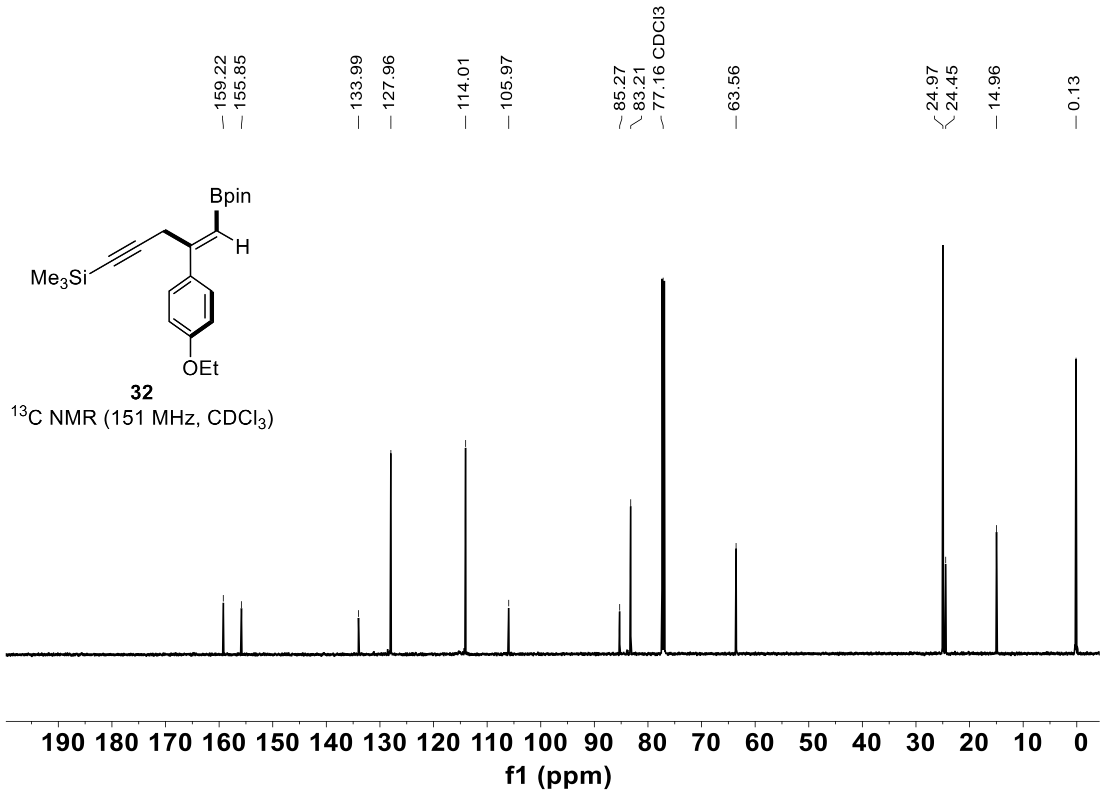
159.22
155.85
133.99
127.96
114.01
105.97
85.27
83.21
77.16 CDCl3
63.56
24.97
24.45
14.96
0.13
Bpin
H
Me_3Si
OEt
32
^{13}C NMR (151 MHz, $CDCl_3$)
f1 (ppm)

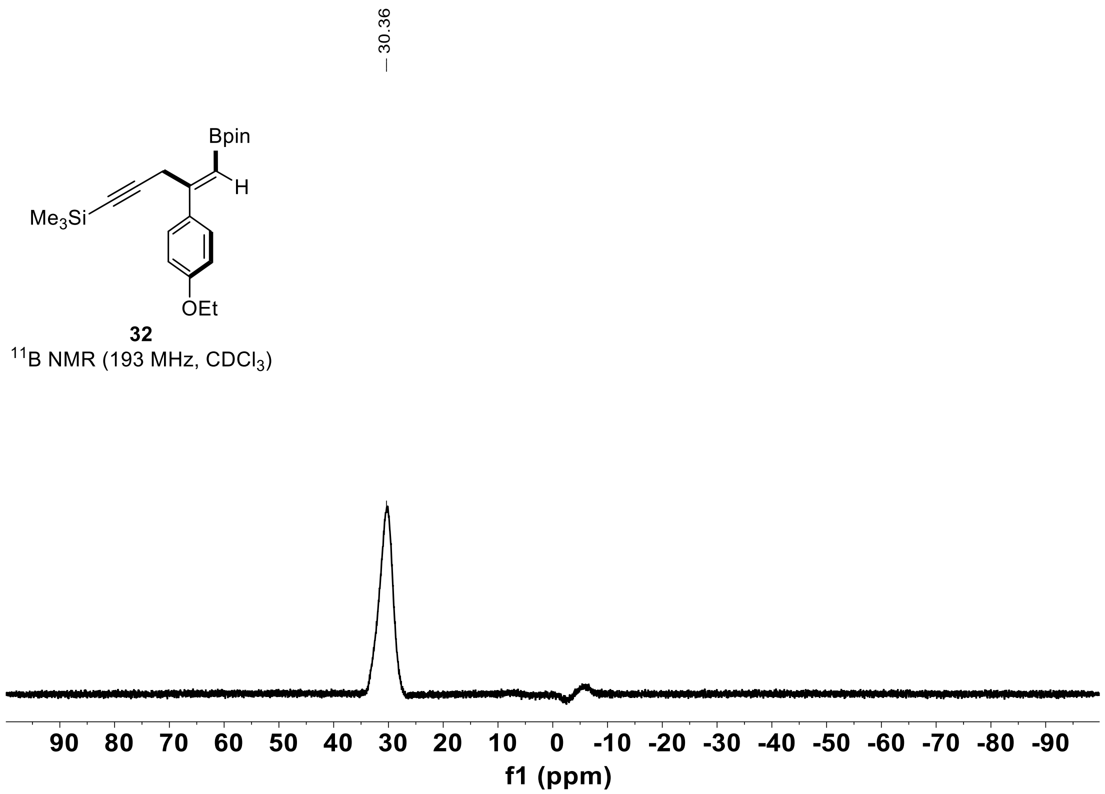
30.36
Bpin
H
Me3Si
OEt
32
11B NMR (193 MHz, CDCl3)
90 80 70 60 50 40 30 20 10 0 -10 -20 -30 -40 -50 -60 -70 -80 -90
f1 (ppm)

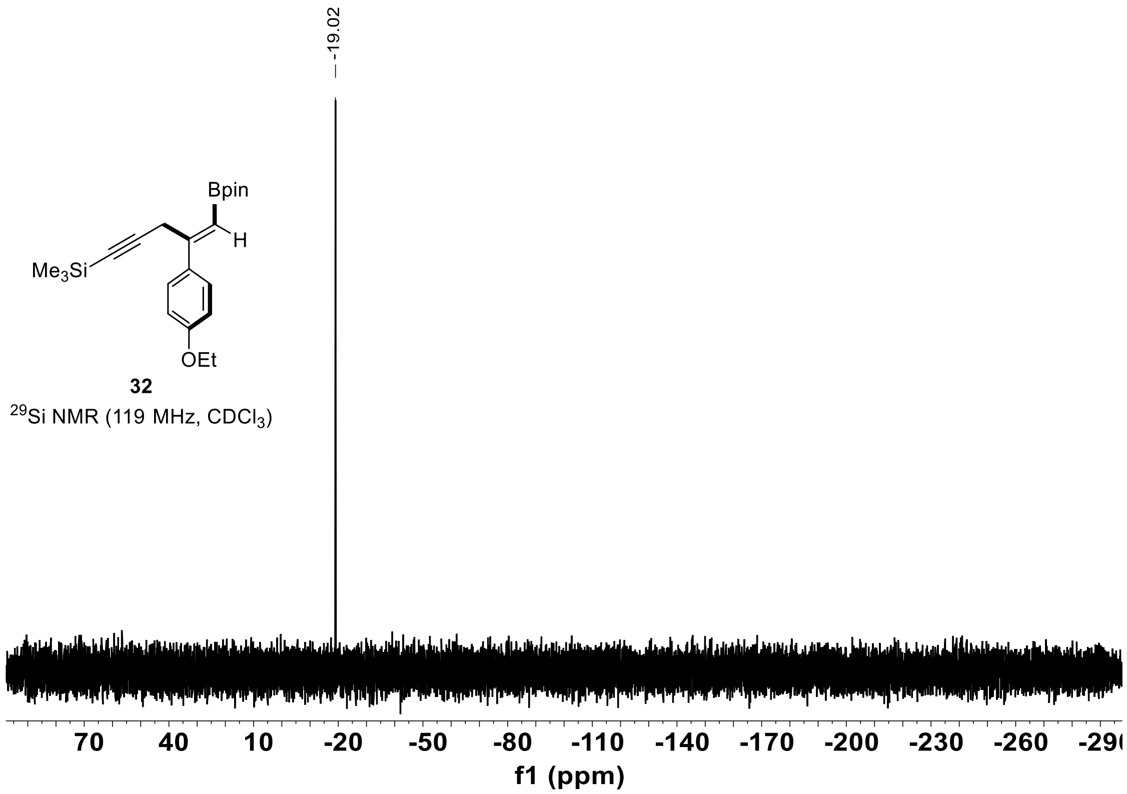
-19.02
Bpin
H
Me3Si
OEt
32
29Si NMR (119 MHz, CDCl3)
70 40 10 -20 -50 -80 -110 -140 -170 -200 -230 -260
f1 (ppm)

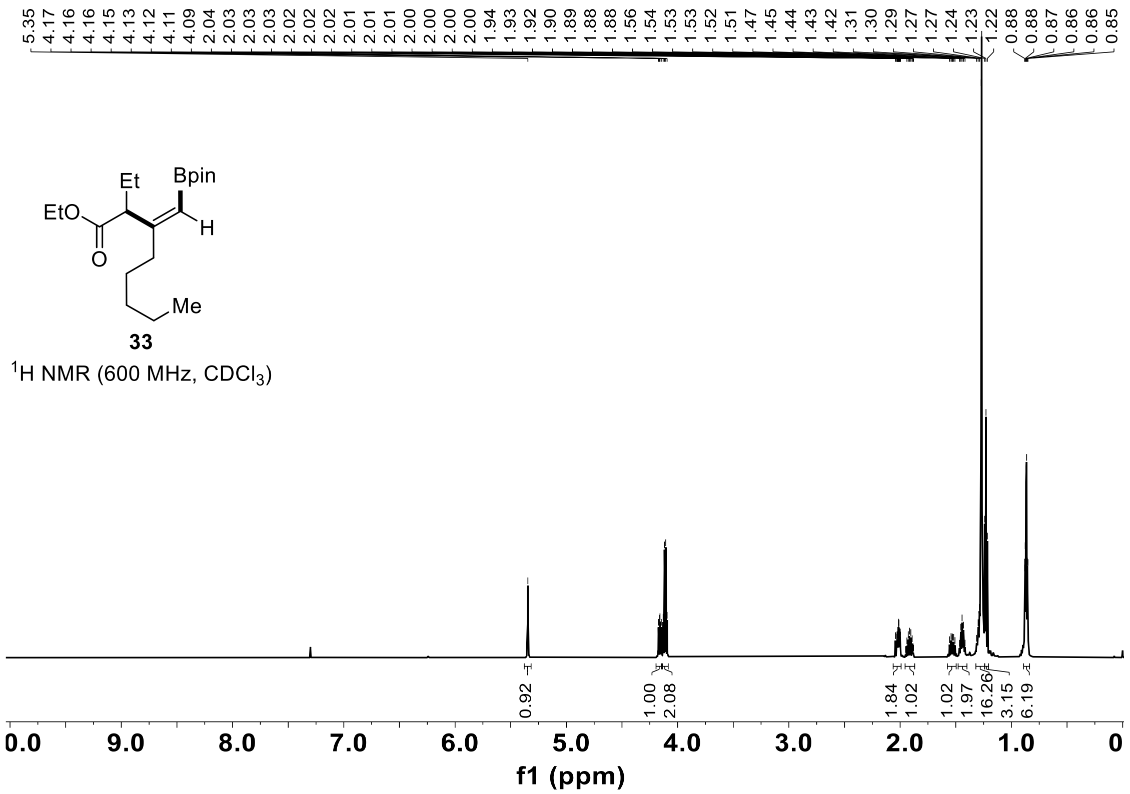
5.35
4.17
4.16
4.16
4.15
4.13
4.12
4.11
4.09
2.04
2.03
2.03
2.03
2.02
2.02
2.02
2.01
2.01
2.01
2.00
2.00
2.00
2.00
1.94
1.93
1.92
1.90
1.89
1.88
1.88
1.56
1.54
1.53
1.53
1.52
1.51
1.47
1.45
1.44
1.43
1.42
1.31
1.30
1.29
1.27
1.27
1.24
1.23
1.22
0.88
0.88
0.87
0.86
0.86
0.85
Et
Bpin
EtO
H
O
Me
33
^{1}H NMR (600 MHz, CDCl$_3$)
0.92
1.00
2.08
1.84
1.02
1.02
1.97
16.26
3.15
6.19
0.0
9.0
8.0
7.0
6.0
5.0
4.0
3.0
2.0
1.0
0
f1 (ppm)

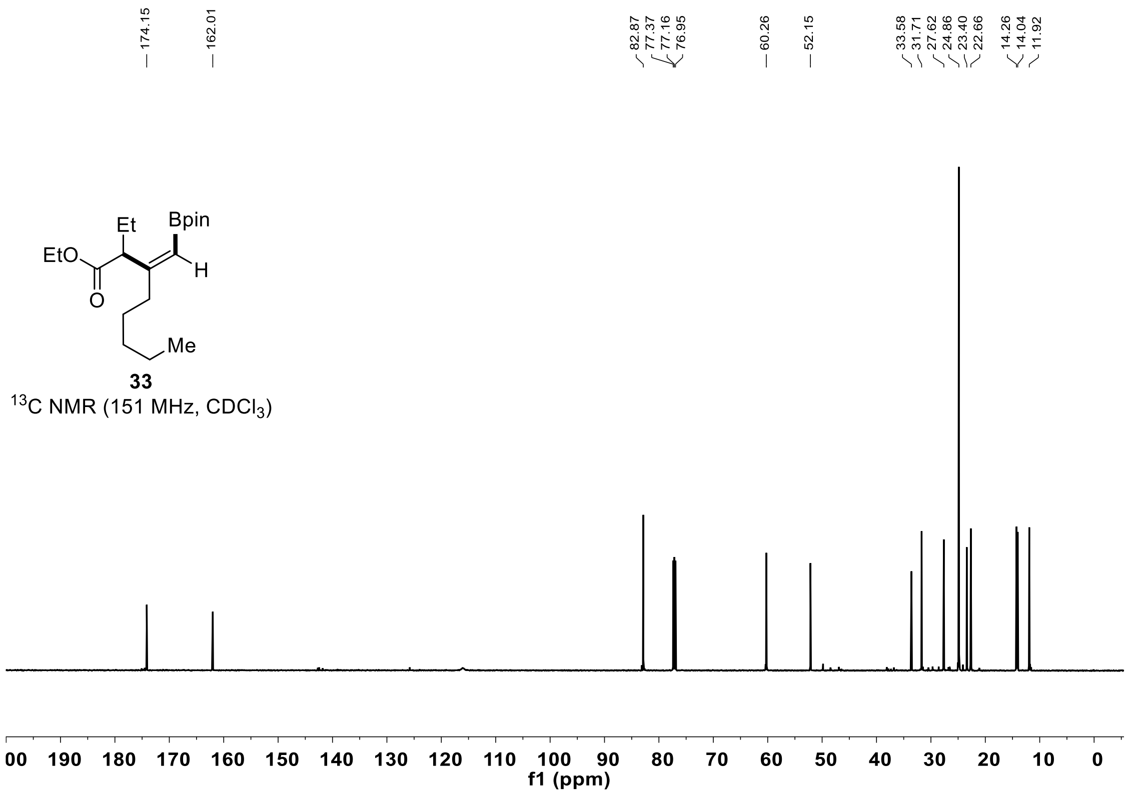
174.15
162.01
82.87
77.37
77.16
76.95
60.26
52.15
33.58
31.71
27.62
24.86
23.40
22.66
14.26
14.04
11.92
Et
Bpin
EtO
H
O
Me
33
^{13}C NMR (151 MHz, CDCl$_3$)
00
190
180
170
160
150
140
130
120
110
100
90
80
70
60
50
40
30
20
10
0
f1 (ppm)

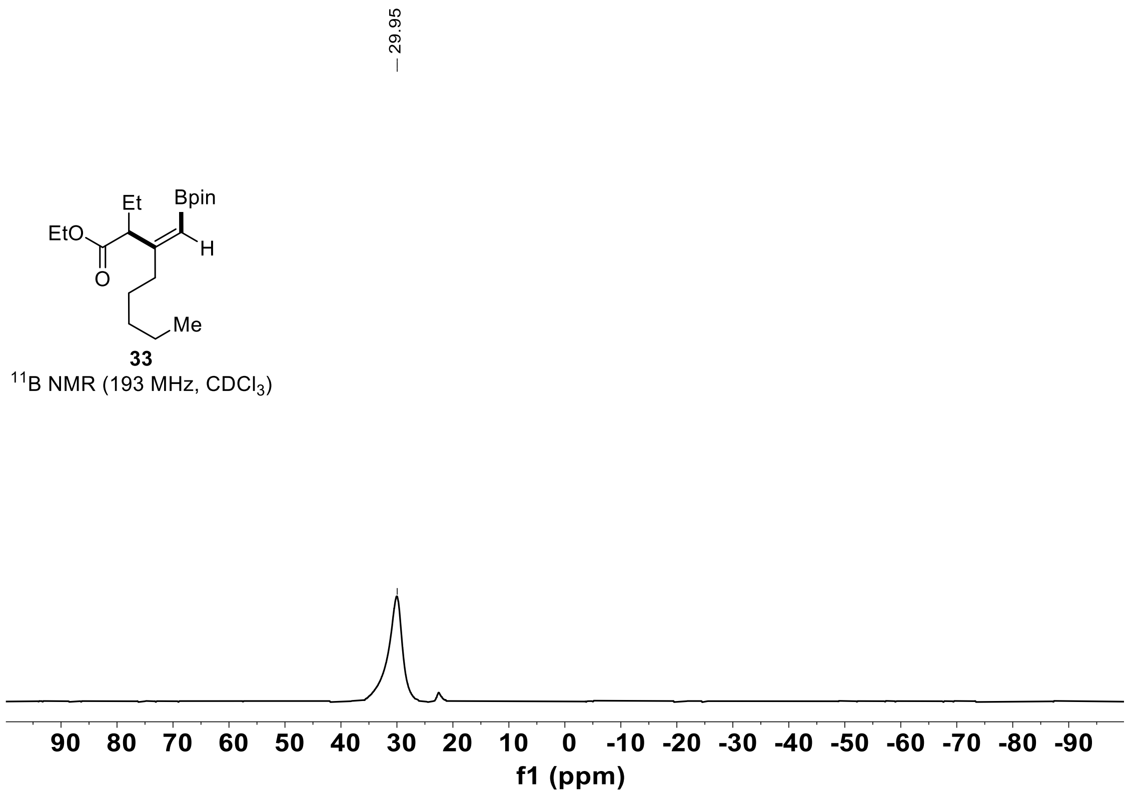
29.95
Et
Bpin
EtO
H
O
Me
33
11B NMR (193 MHz, CDCl3)
f1 (ppm)

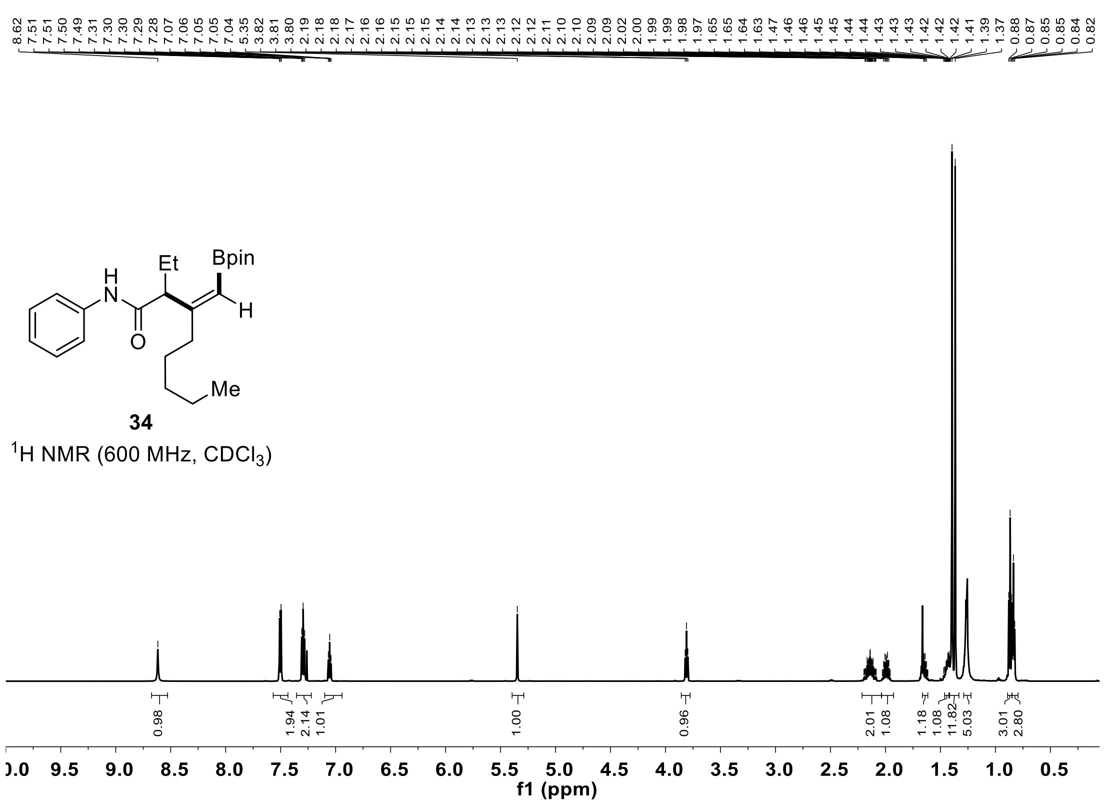
H
N
Et
Bpin
H
O
Me
34
1H NMR (600 MHz, CDCl3)
f1 (ppm)

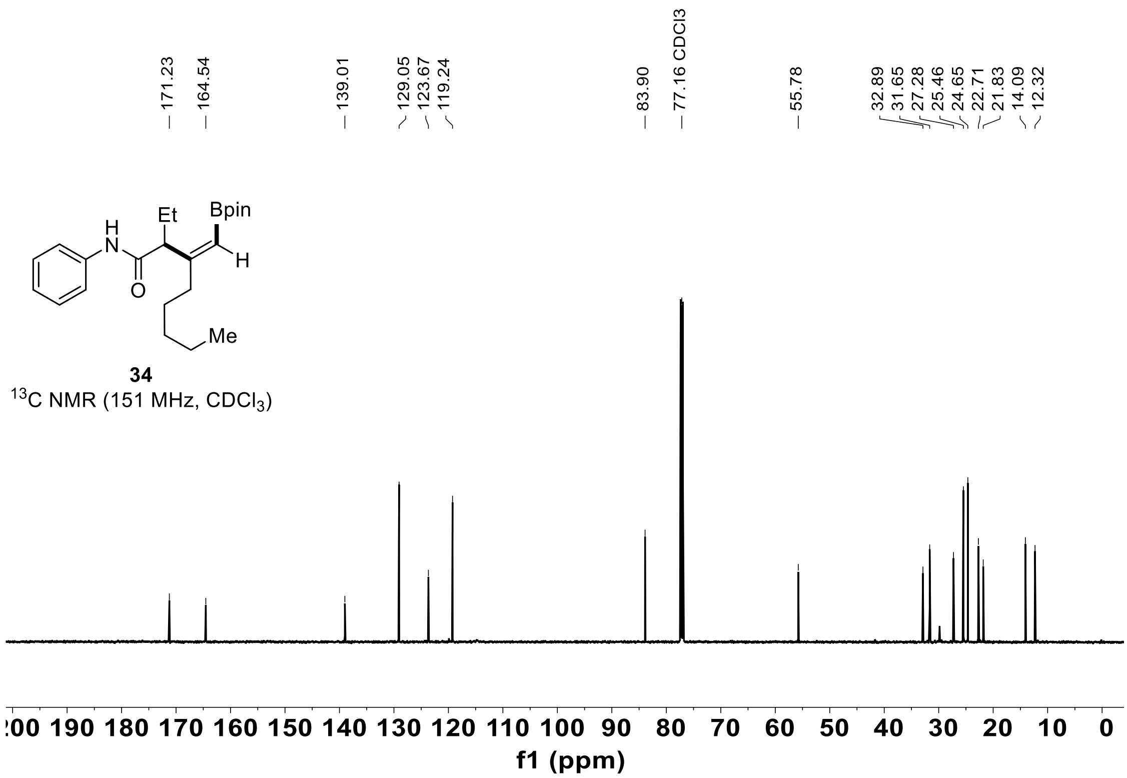

34

^{13}C NMR (151 MHz, $CDCl_3$)

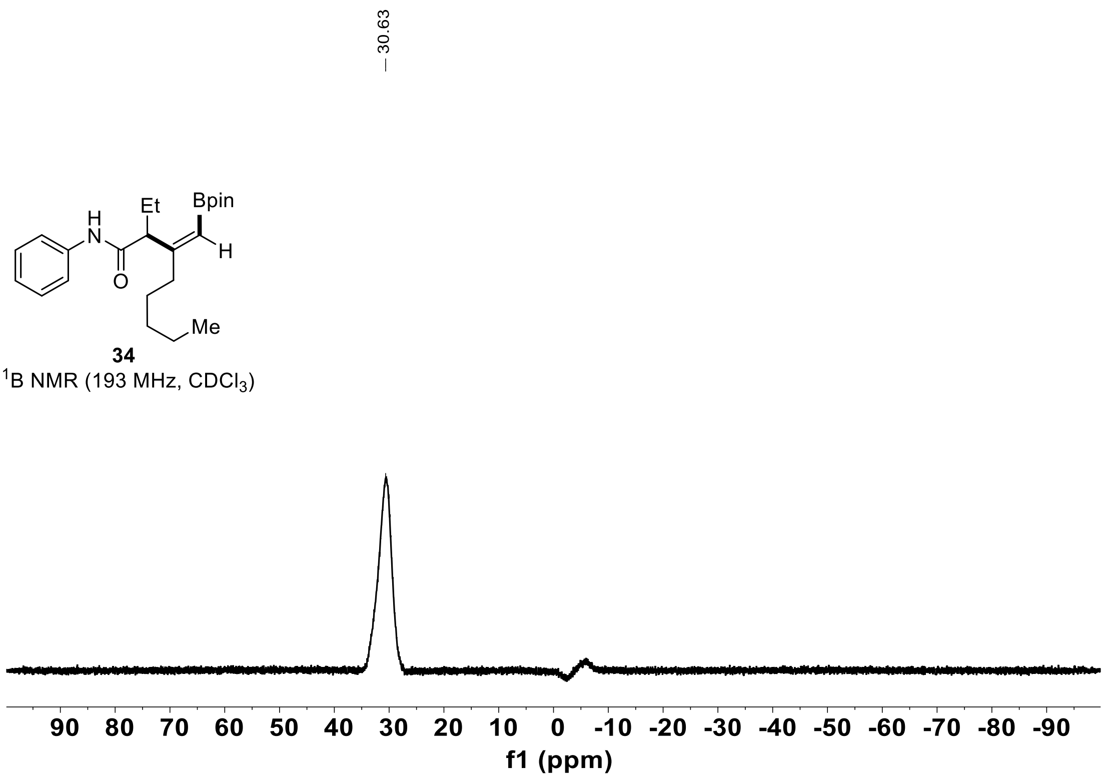

34

^{11}B NMR (193 MHz, $CDCl_3$)

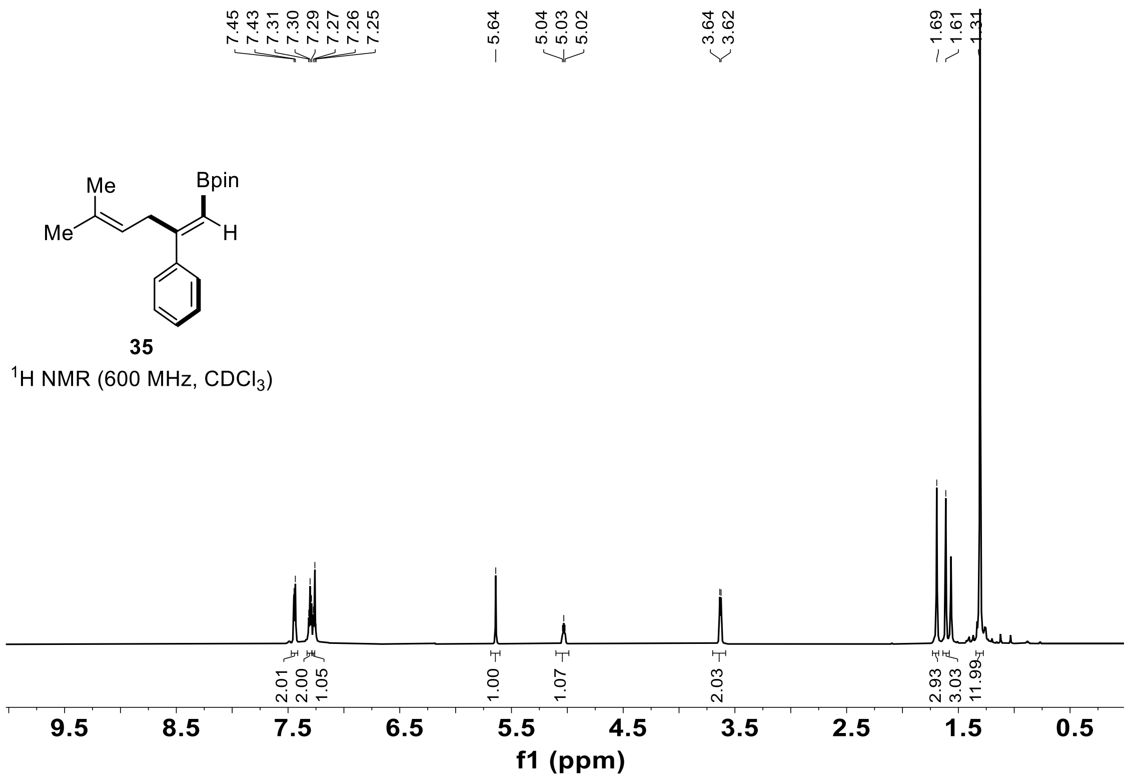

Me
Me
Bpin
H
35
¹H NMR (600 MHz, CDCl₃)
7.45
7.43
7.31
7.30
7.29
7.27
7.26
7.25
5.64
5.04
5.03
5.02
3.64
3.62
1.69
1.61
1.31
2.01
2.00
1.05
1.00
1.07
2.03
2.93
3.03
11.99
9.5
8.5
7.5
6.5
5.5
4.5
3.5
2.5
1.5
0.5
f1 (ppm)

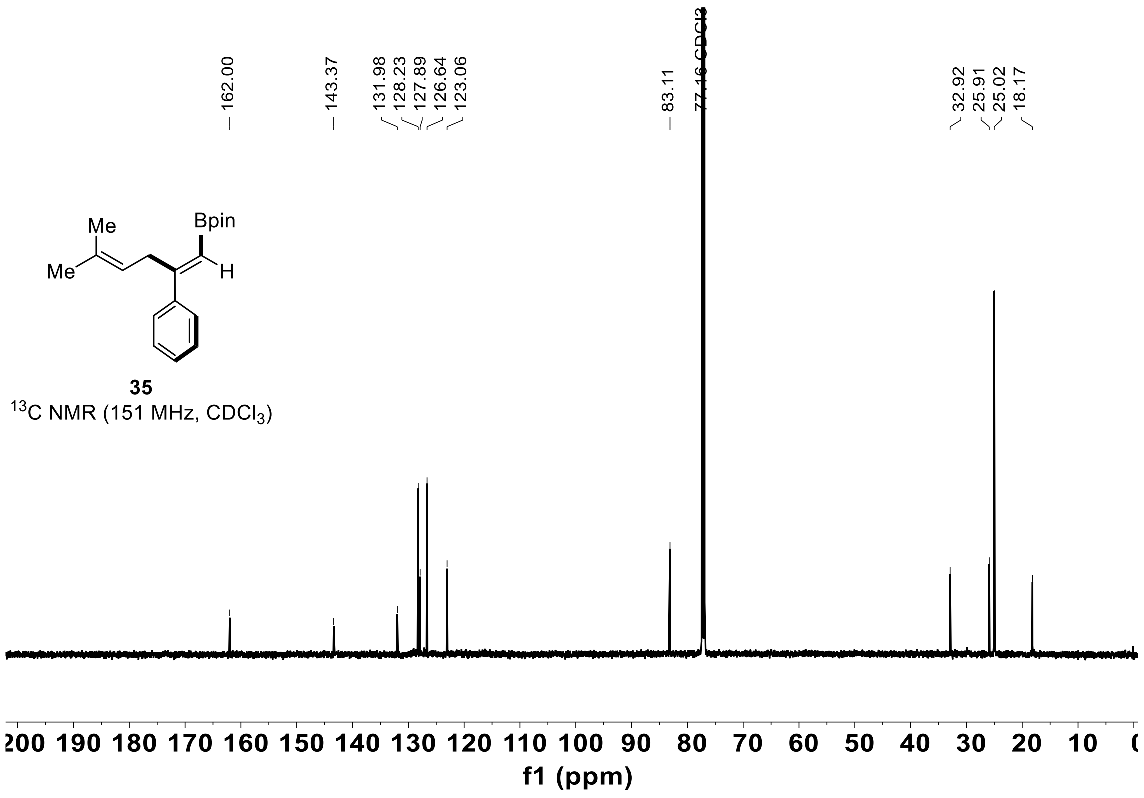

Me
Me
Bpin
H
35
¹³C NMR (151 MHz, CDCl₃)
162.00
143.37
131.98
128.23
127.89
126.64
123.06
83.11
77.16 CDCl3
32.92
25.91
25.02
18.17
200 190 180 170 160 150 140 130 120 110 100 90 80 70 60 50 40 30 20 10
f1 (ppm)

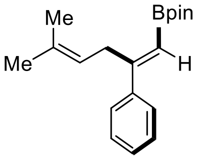

35

^{11}B NMR (193 MHz, $CDCl_3$)

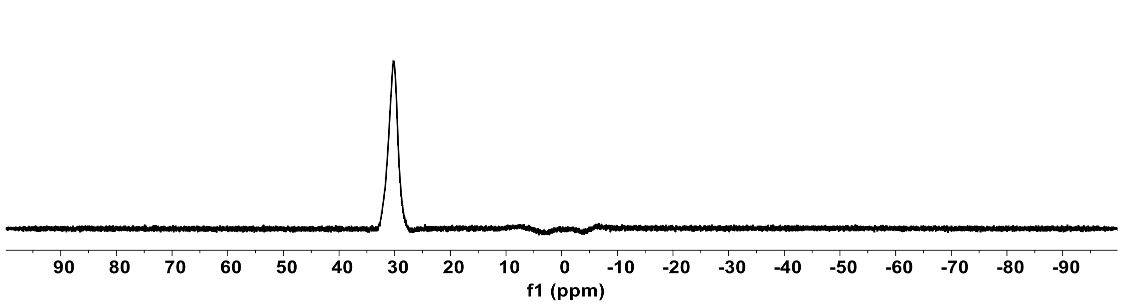

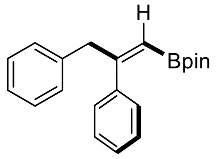

36

^{1}H NMR (600 MHz, $CDCl_3$)

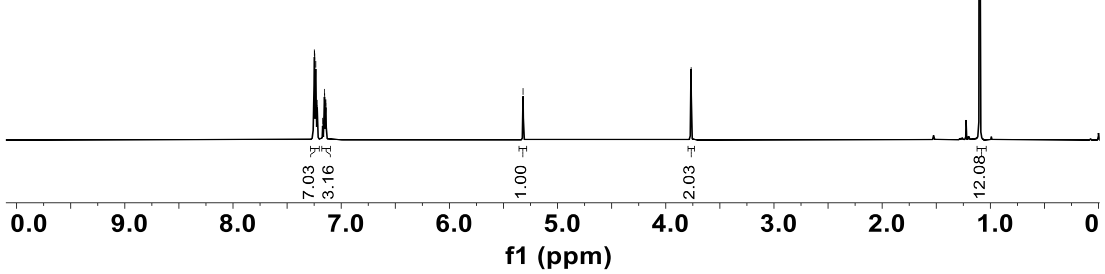

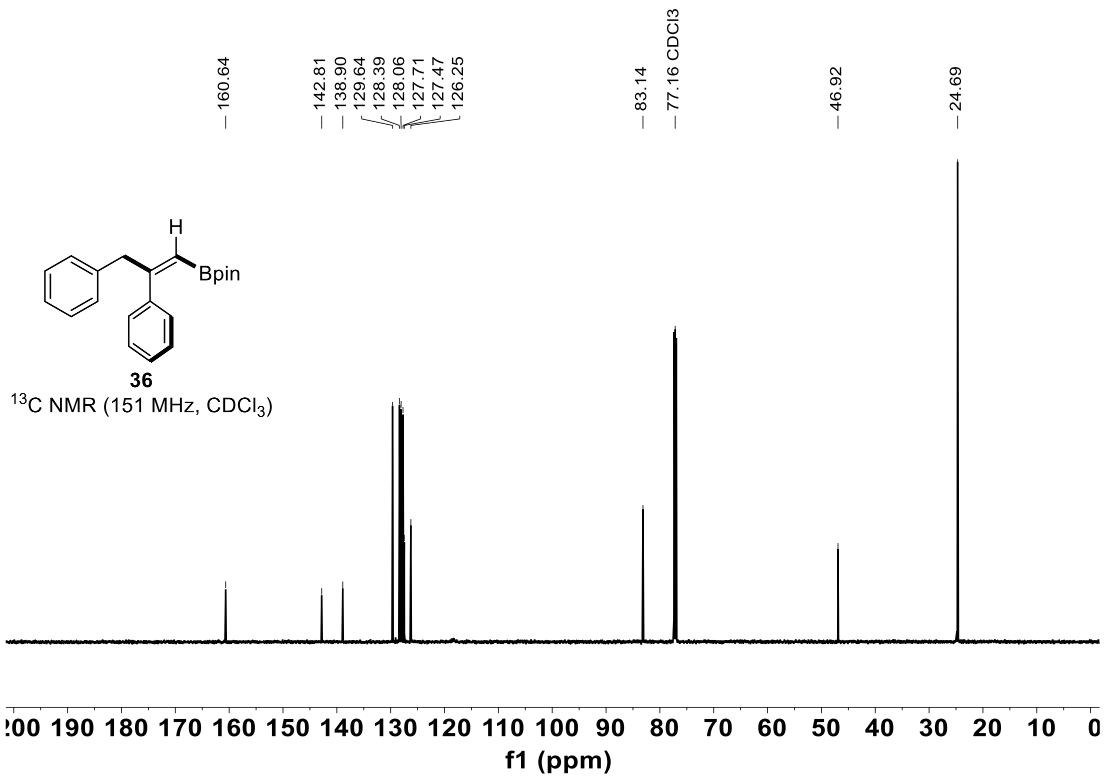

160.64
142.81
138.90
129.64
128.39
128.06
127.71
127.47
126.25
83.14
77.16 CDCl3
46.92
24.69
H
Bpin
36
^{13}C NMR (151 MHz, $CDCl_3$)
200 190 180 170 160 150 140 130 120 110 100 90 80 70 60 50 40 30 20 10 0
f1 (ppm)

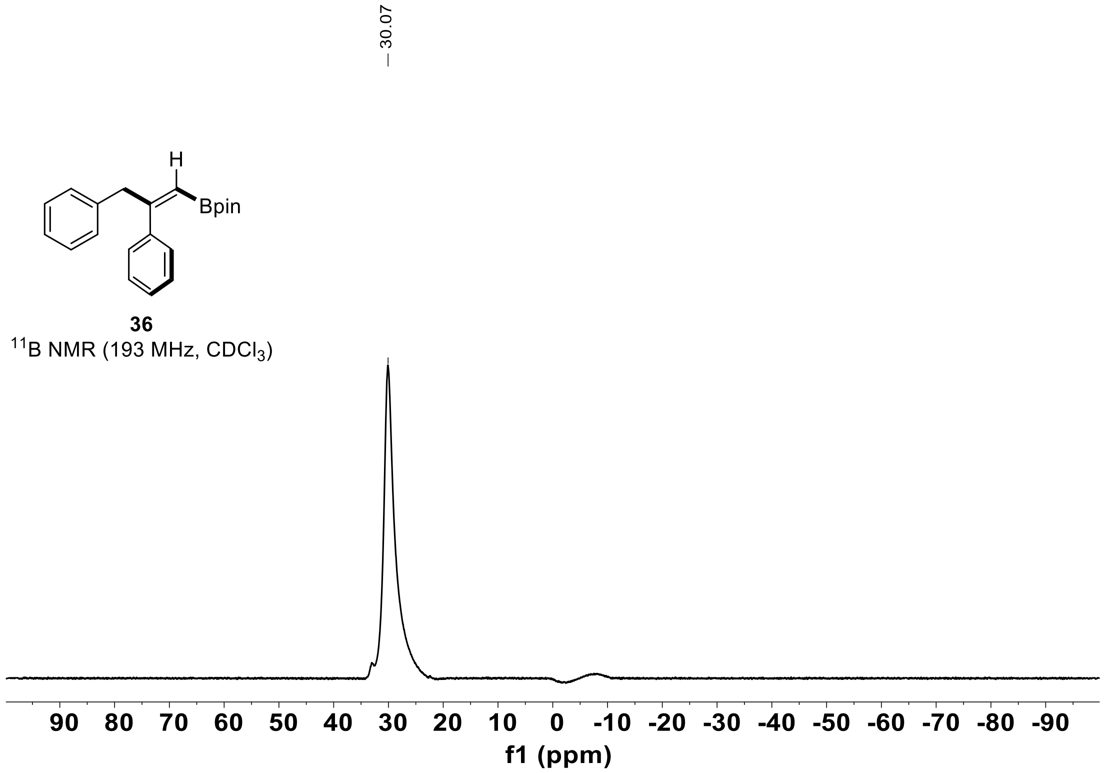

30.07
H
Bpin
36
^{11}B NMR (193 MHz, $CDCl_3$)
90 80 70 60 50 40 30 20 10 0 -10 -20 -30 -40 -50 -60 -70 -80 -90
f1 (ppm)

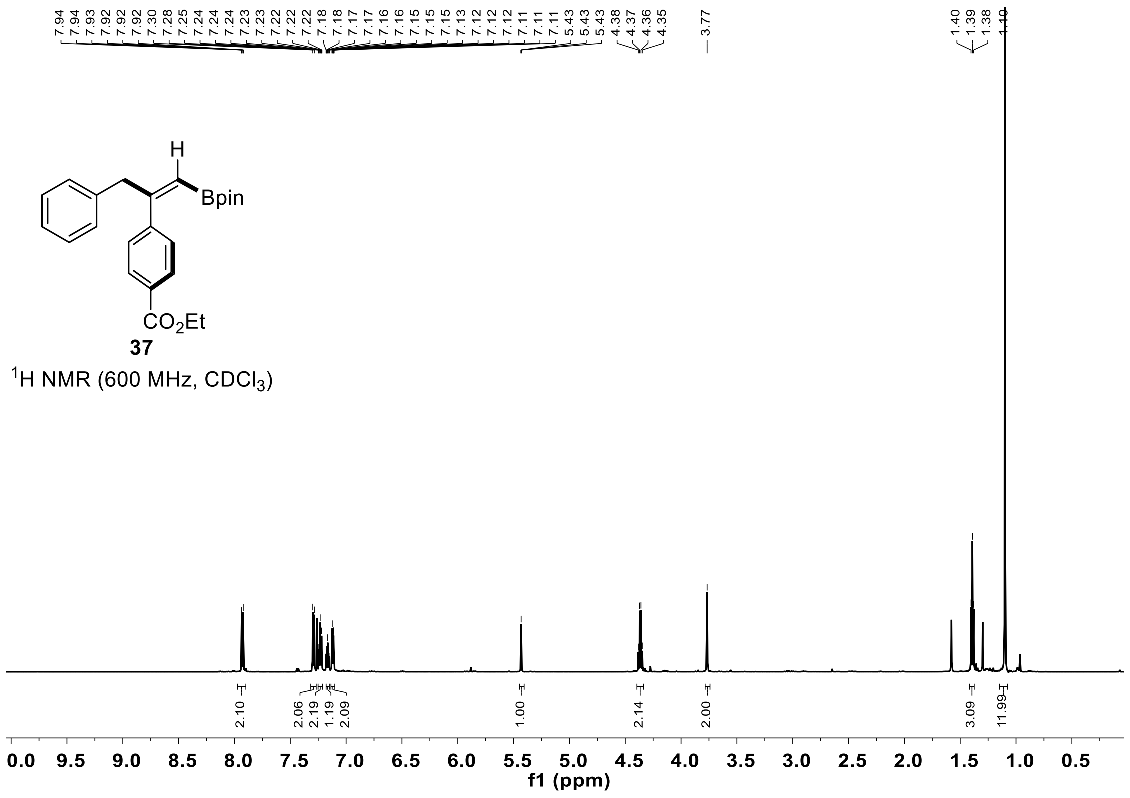
Bpin
CO2Et
37
1H NMR (600 MHz, CDCl3)
f1 (ppm)

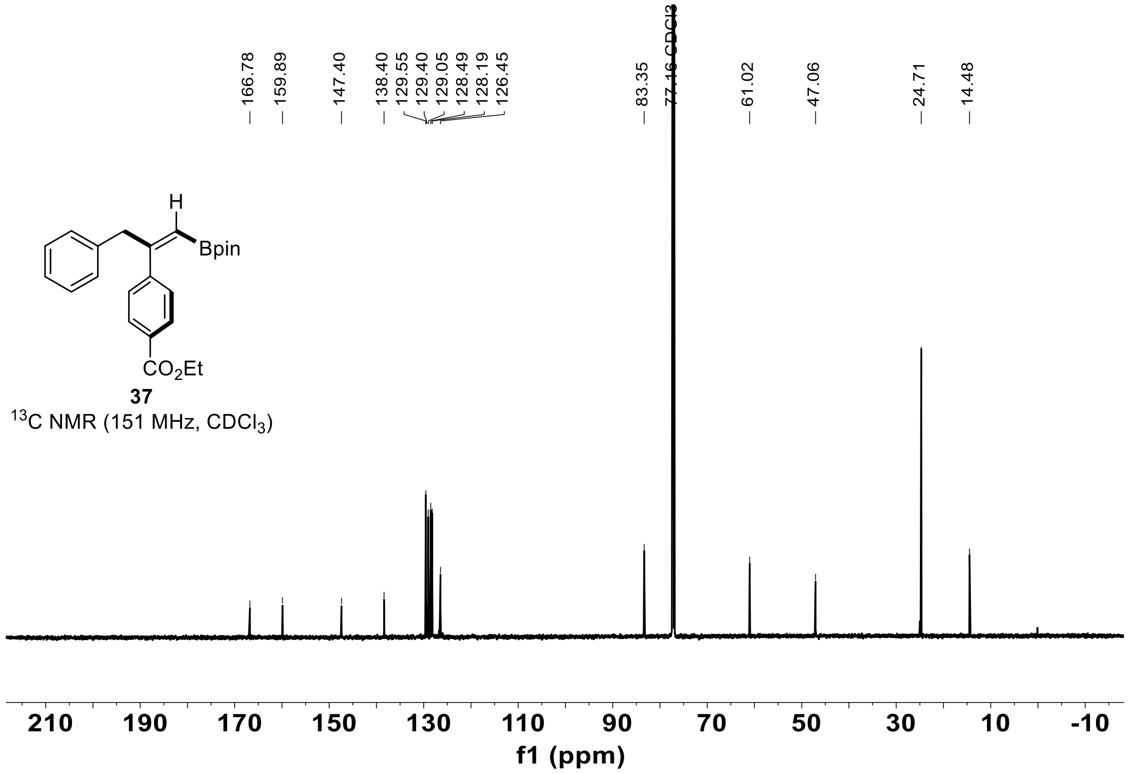
Bpin
CO2Et
37
13C NMR (151 MHz, CDCl3)
f1 (ppm)

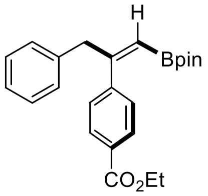

37

^{11}B NMR (193 MHz, $CDCl_3$)

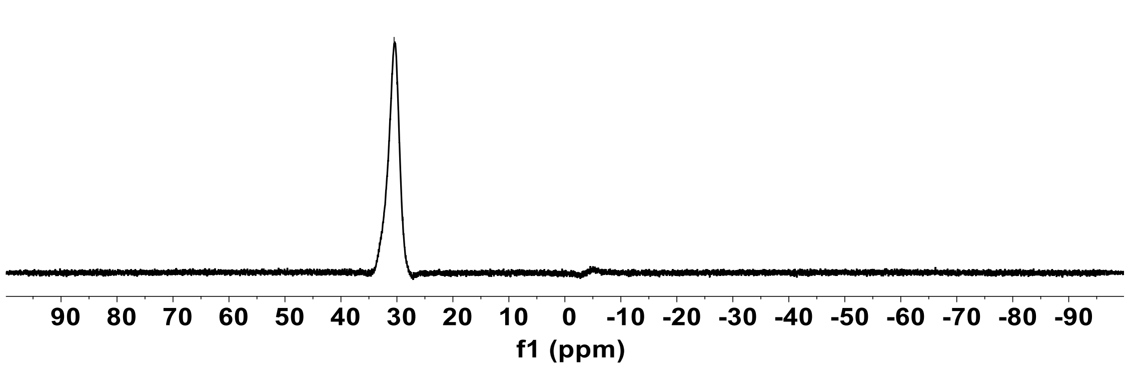

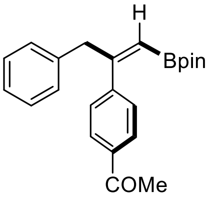

38

^{1}H NMR (600 MHz, $CDCl_3$)

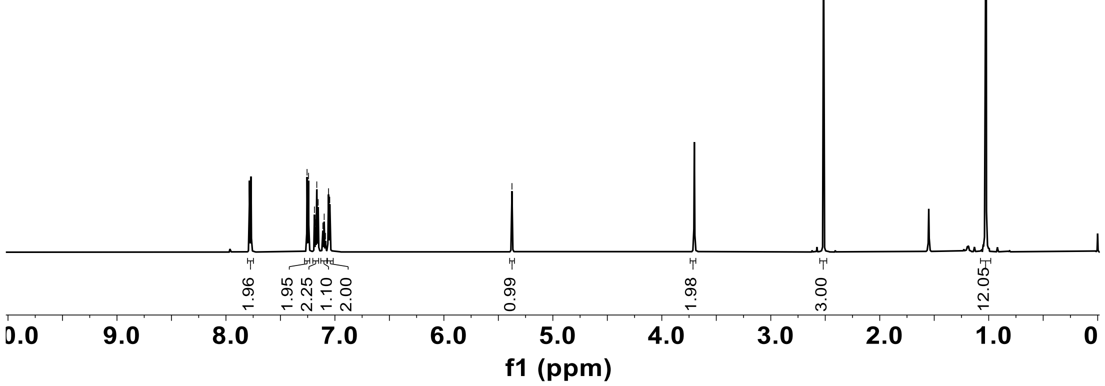

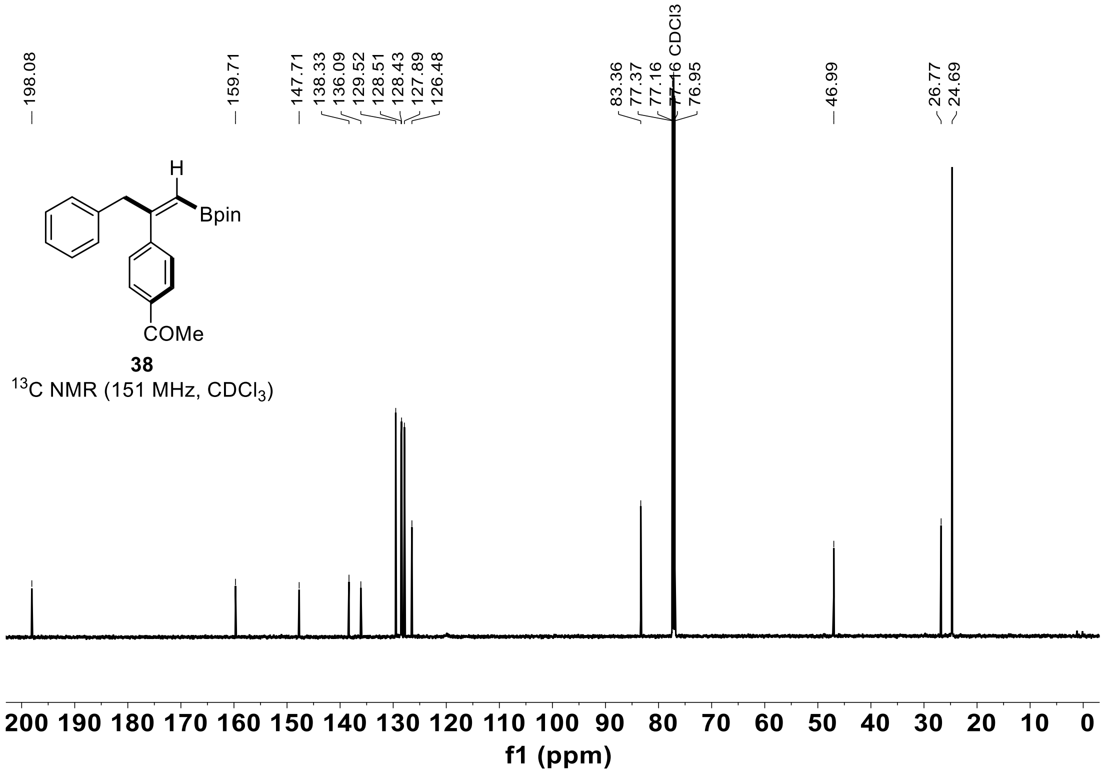
198.08
159.71
147.71
138.33
136.09
129.52
128.51
128.43
127.89
126.48
83.36
77.37
77.16
77.16 CDCl3
76.95
46.99
26.77
24.69
H
Bpin
COMe
38
^{13}C NMR (151 MHz, $CDCl_3$)
200 190 180 170 160 150 140 130 120 110 100 90 80 70 60 50 40 30 20 10 0
f1 (ppm)

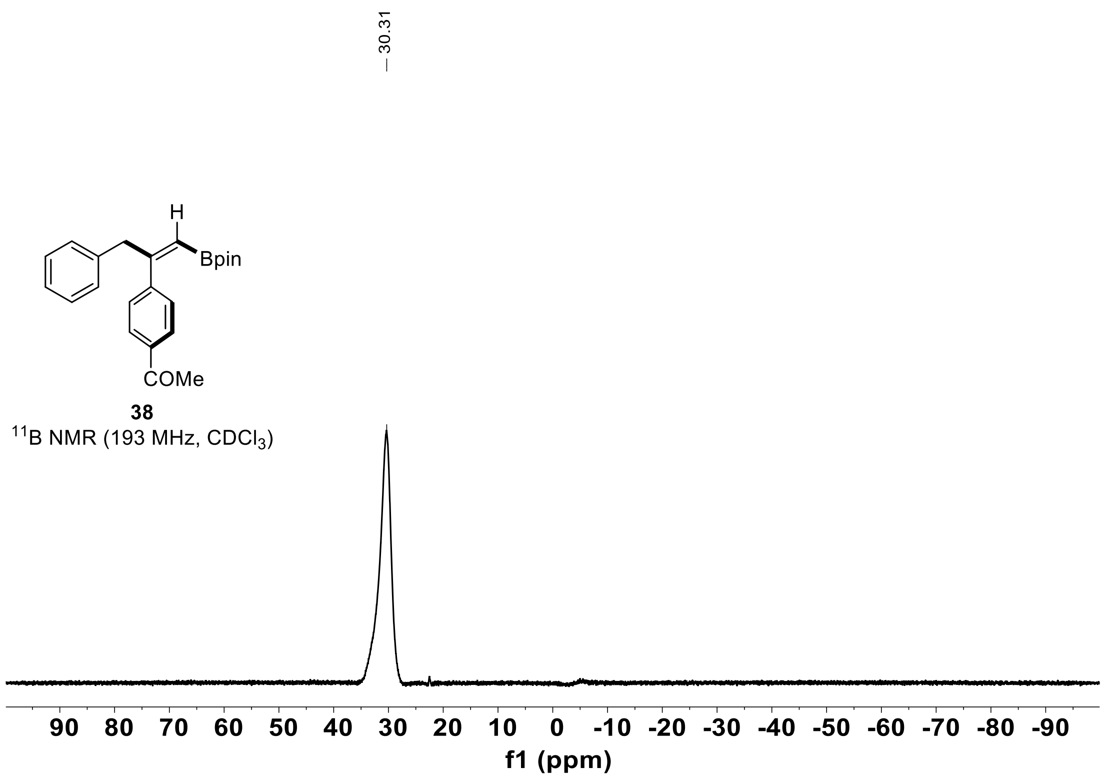
30.31
H
Bpin
COMe
38
^{11}B NMR (193 MHz, $CDCl_3$)
90 80 70 60 50 40 30 20 10 0 -10 -20 -30 -40 -50 -60 -70 -80 -90
f1 (ppm)

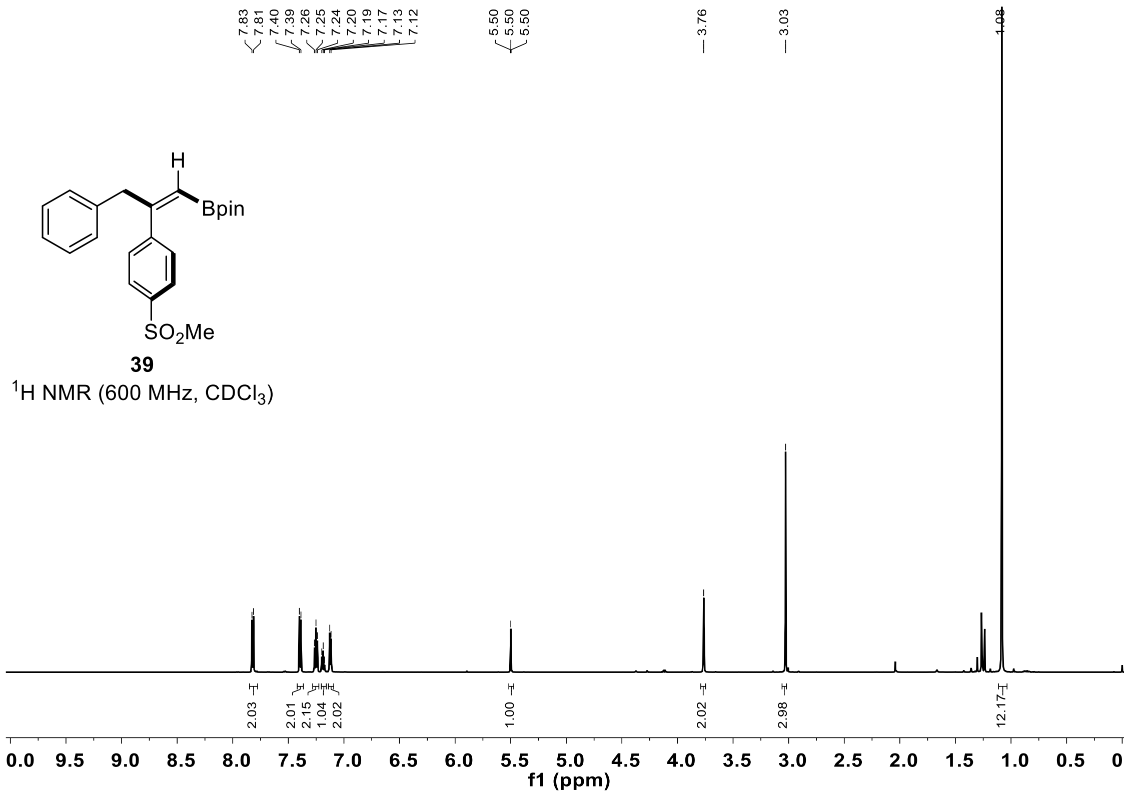
7.83
7.81
7.40
7.39
7.26
7.25
7.24
7.20
7.19
7.17
7.13
7.12
5.50
5.50
5.50
3.76
3.03
1.08
H
Bpin
SO2Me
39
1H NMR (600 MHz, CDCl3)
2.03
2.01
2.15
1.04
2.02
1.00
2.02
2.98
12.17
0.0 9.5 9.0 8.5 8.0 7.5 7.0 6.5 6.0 5.5 5.0 4.5 4.0 3.5 3.0 2.5 2.0 1.5 1.0 0.5 0
f1 (ppm)

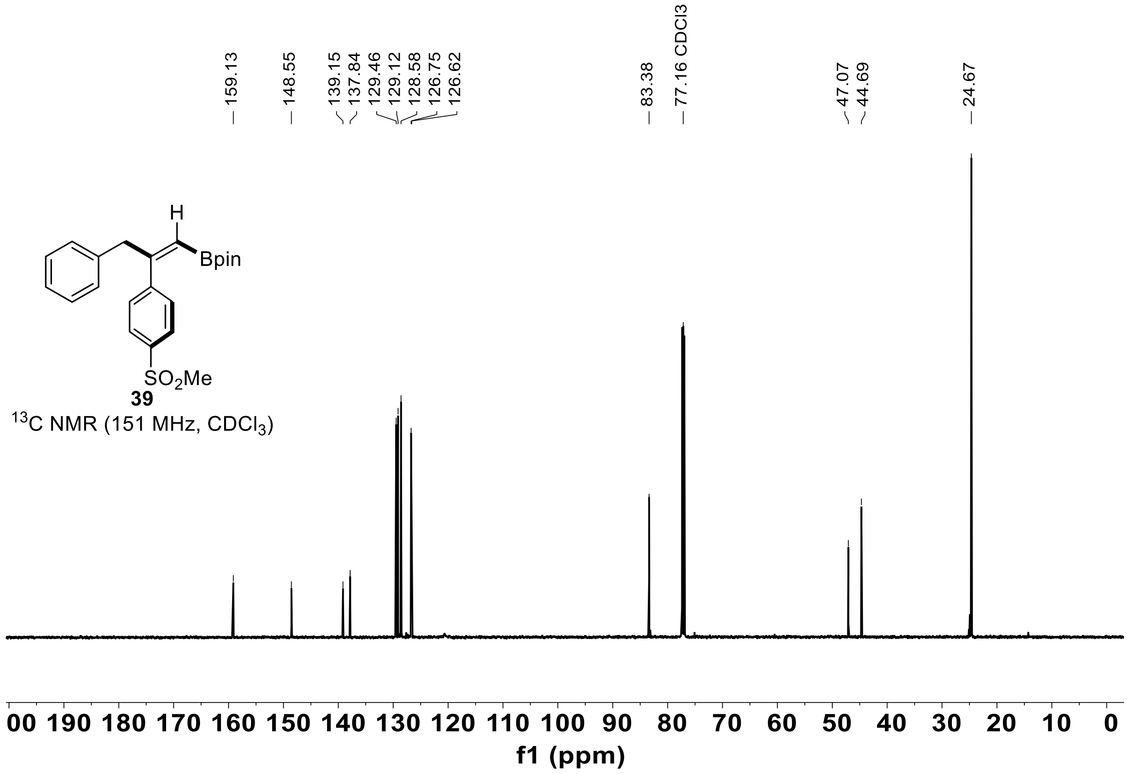
159.13
148.55
139.15
137.84
129.46
129.12
128.58
126.75
126.62
83.38
77.16 CDCl3
47.07
44.69
24.67
H
Bpin
SO2Me
39
13C NMR (151 MHz, CDCl3)
00 190 180 170 160 150 140 130 120 110 100 90 80 70 60 50 40 30 20 10 0
f1 (ppm)

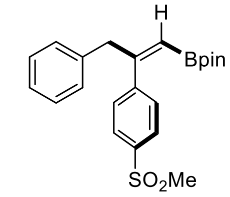

39

^{11}B NMR (193 MHz, $CDCl_3$)

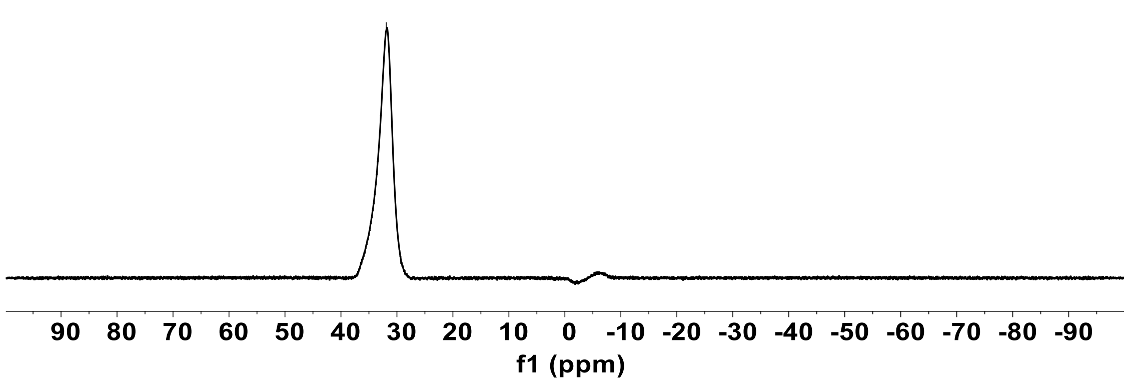

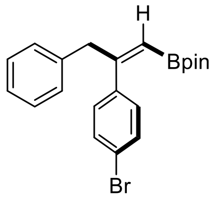

40

^{1}H NMR (600 MHz, $CDCl_3$)

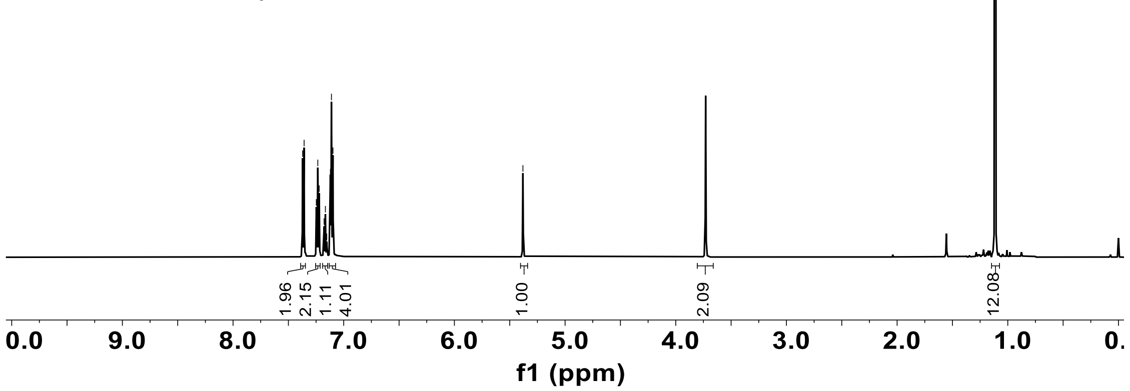

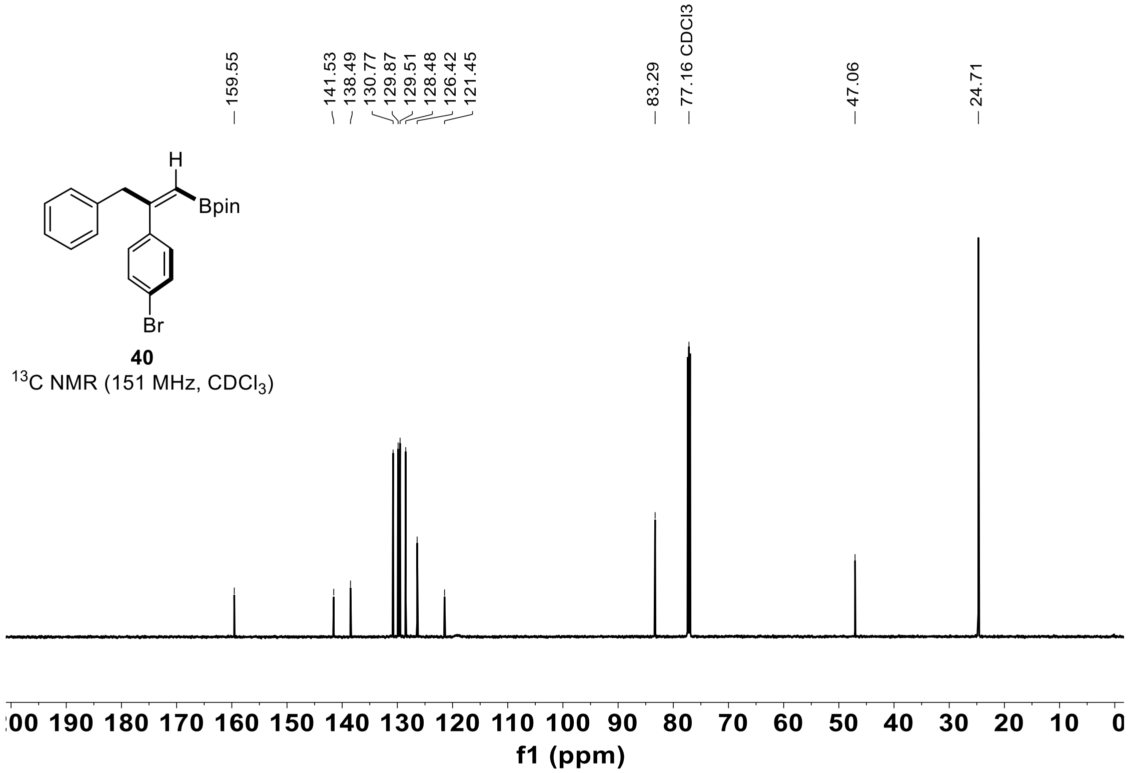
159.55
141.53
138.49
130.77
129.87
129.51
128.48
126.42
121.45
83.29
77.16 CDCl3
47.06
24.71
H
Bpin
Br
40
^{13}C NMR (151 MHz, CDCl$_3$)
00 190 180 170 160 150 140 130 120 110 100 90 80 70 60 50 40 30 20 10 0
f1 (ppm)

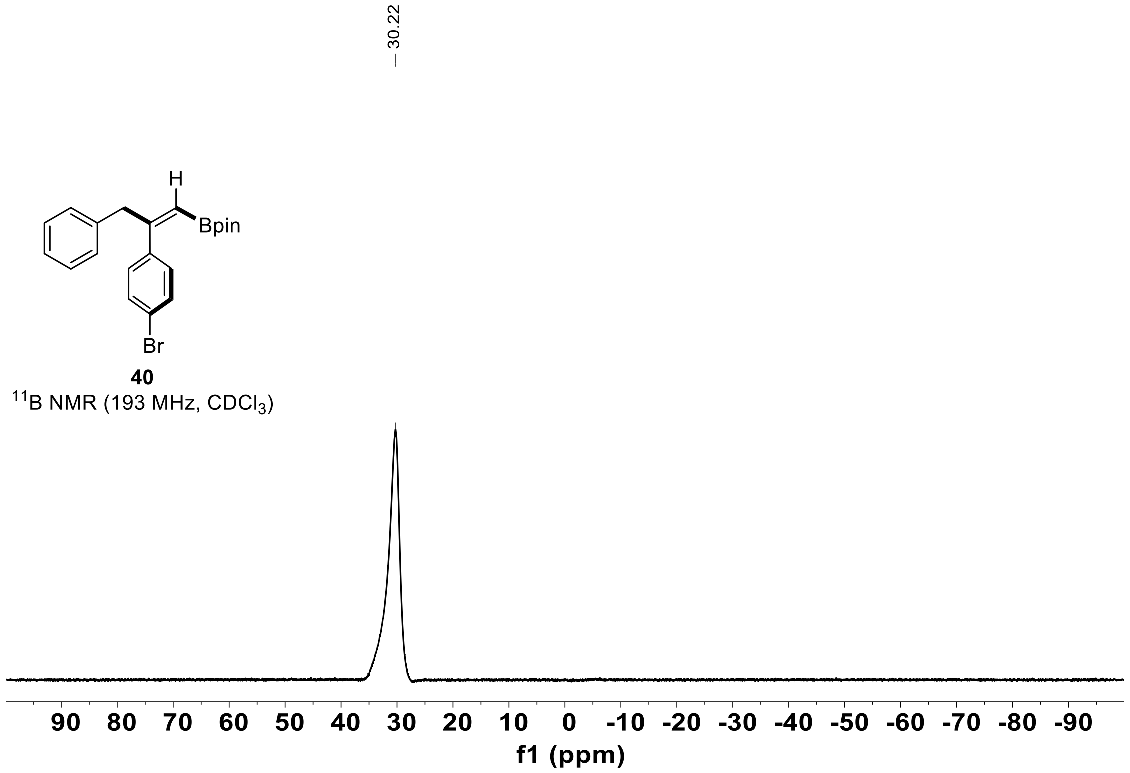
30.22
H
Bpin
Br
40
^{11}B NMR (193 MHz, CDCl$_3$)
90 80 70 60 50 40 30 20 10 0 -10 -20 -30 -40 -50 -60 -70 -80 -90
f1 (ppm)

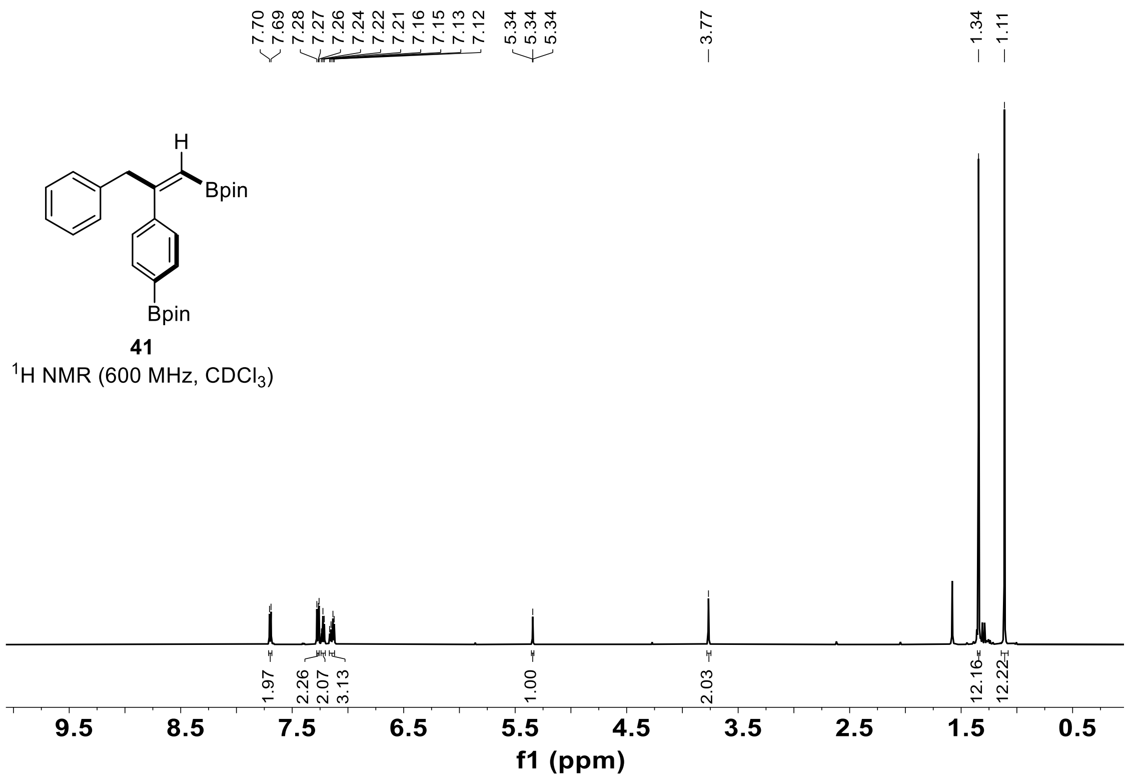
7.70
7.69
7.28
7.27
7.26
7.24
7.22
7.21
7.16
7.15
7.13
7.12
5.34
5.34
5.34
3.77
1.34
1.11
H
Bpin
Bpin
41
^{1}H NMR (600 MHz, $CDCl_3$)
1.97
2.26
2.07
3.13
1.00
2.03
12.16
12.22
9.5
8.5
7.5
6.5
5.5
4.5
3.5
2.5
1.5
0.5
f1 (ppm)

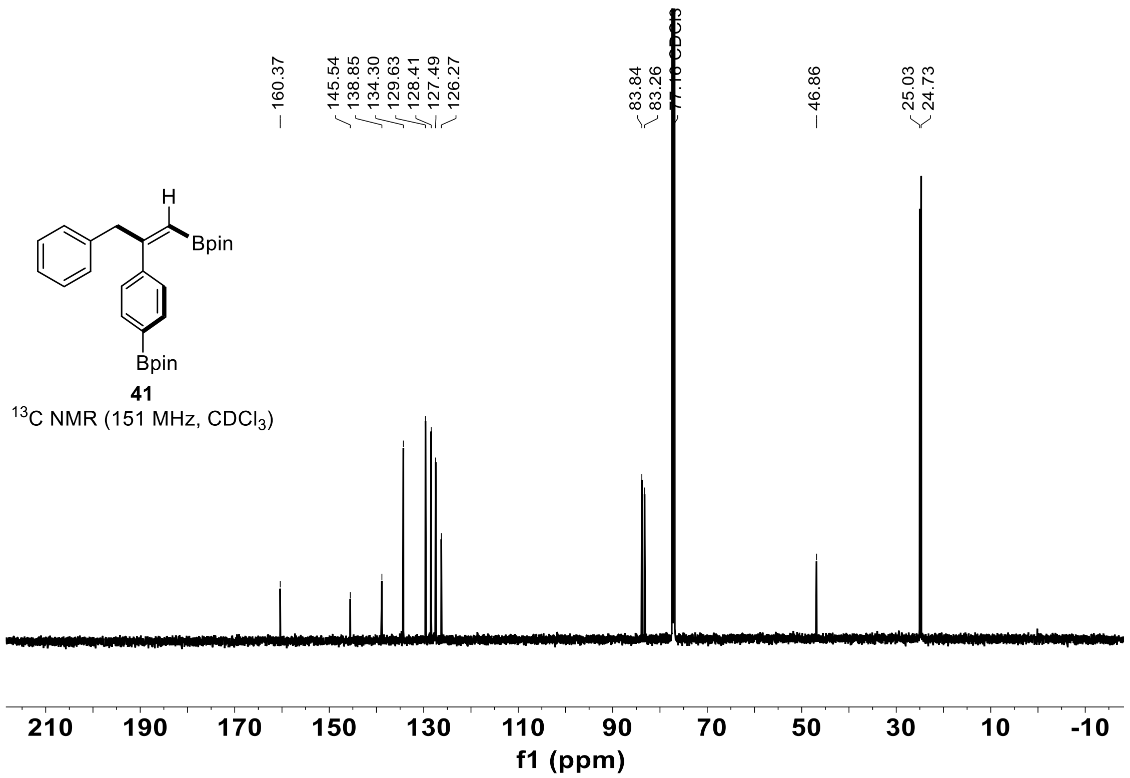
160.37
145.54
138.85
134.30
129.63
128.41
127.49
126.27
83.84
83.26
77.16 CDCl3
46.86
25.03
24.73
H
Bpin
Bpin
41
^{13}C NMR (151 MHz, $CDCl_3$)
210
190
170
150
130
110
90
70
50
30
10
-10
f1 (ppm)

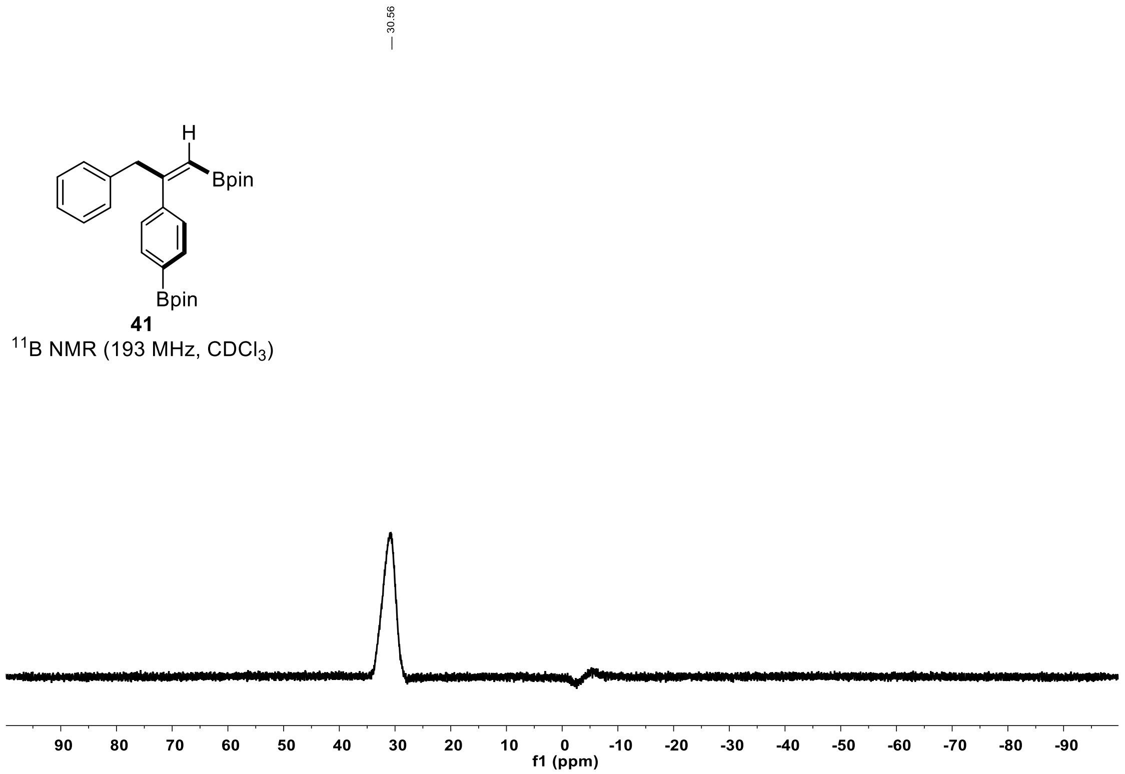

30.56
Bpin
Bpin
41
^{11}B NMR (193 MHz, $CDCl_3$)
f1 (ppm)

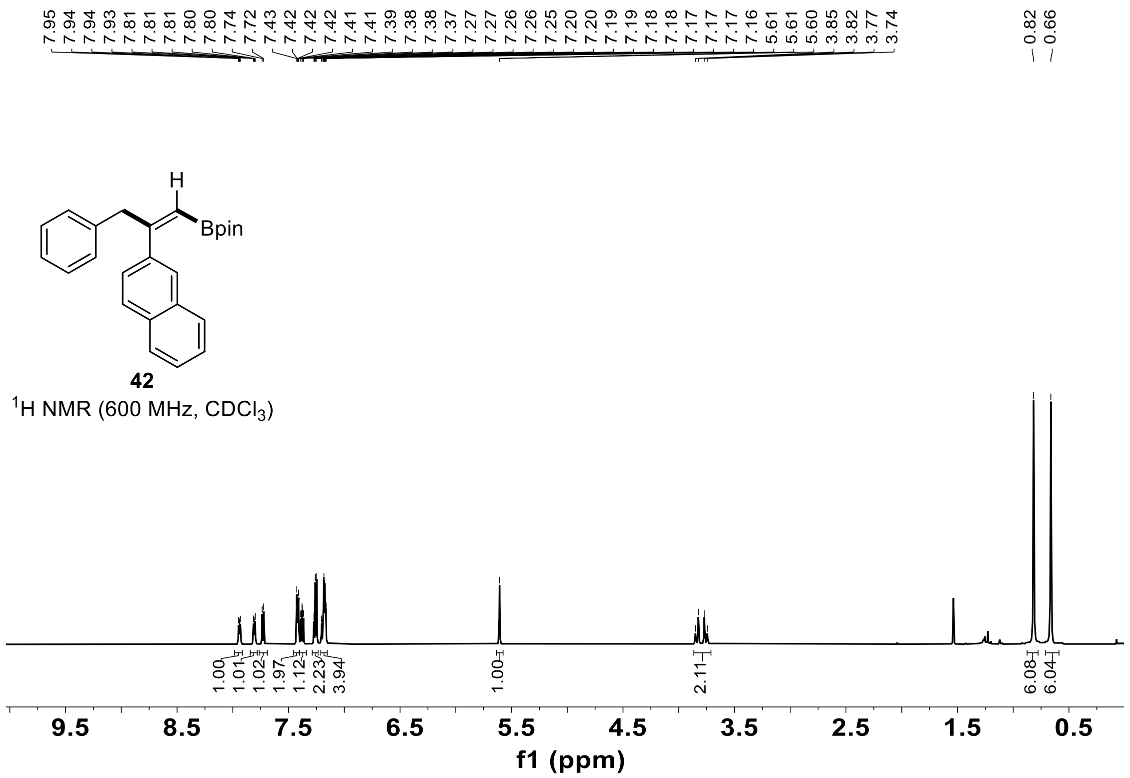

Bpin
42
^{1}H NMR (600 MHz, $CDCl_3$)
f1 (ppm)

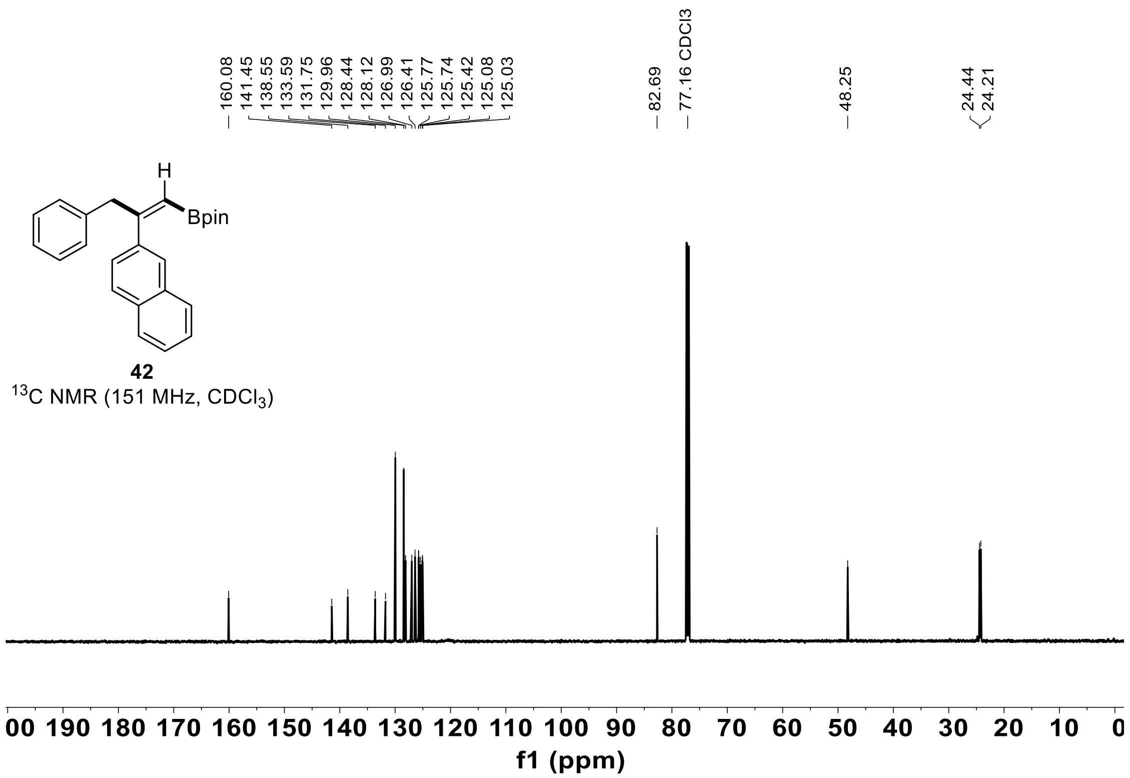

42
^{13}C NMR (151 MHz, $CDCl_3$)

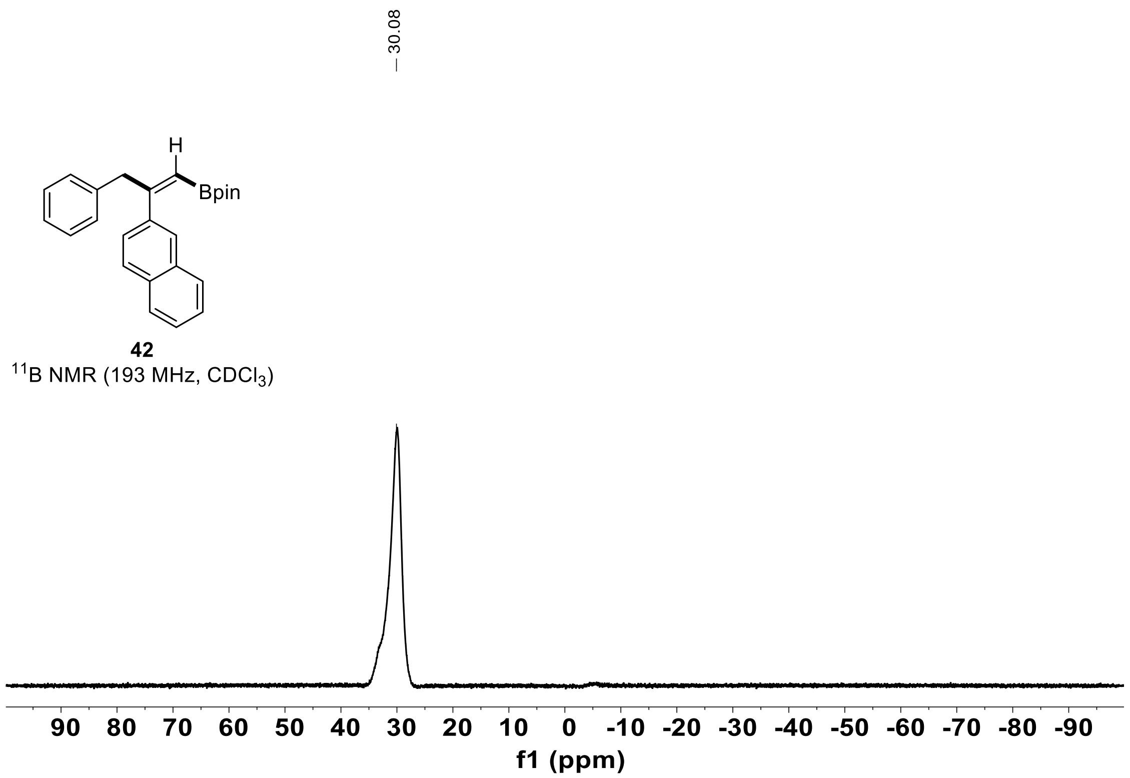

42
^{11}B NMR (193 MHz, $CDCl_3$)

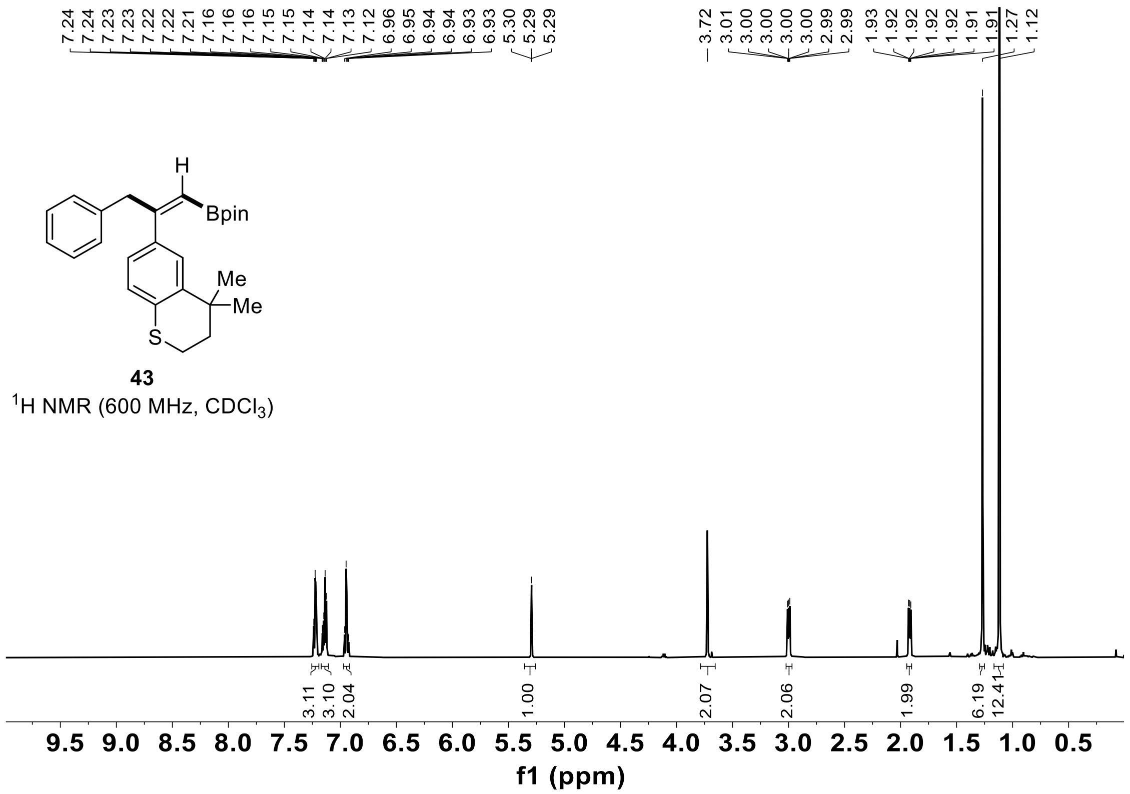

Bpin
Me
Me
S
43
¹H NMR (600 MHz, CDCl₃)
f1 (ppm)

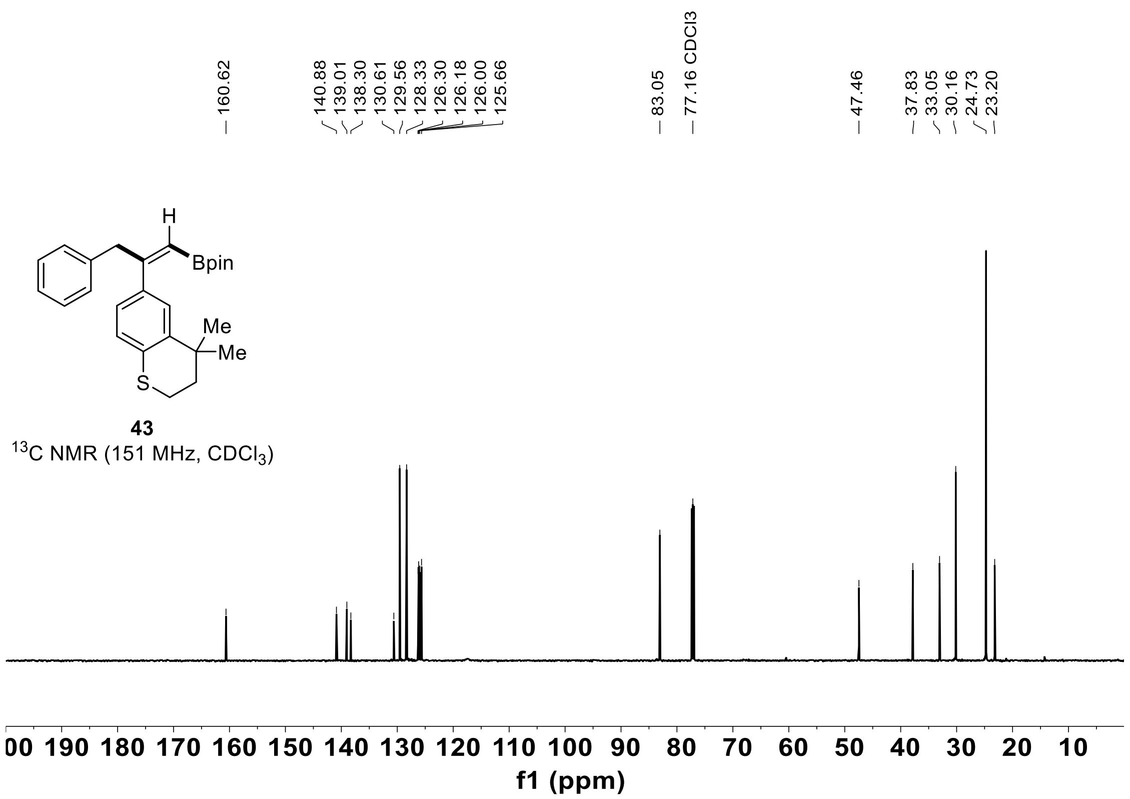

77.16 CDCl3
Bpin
Me
Me
S
43
¹³C NMR (151 MHz, CDCl₃)
f1 (ppm)

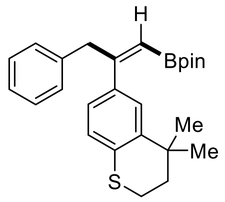

43

^{11}B NMR (193 MHz, $CDCl_3$)

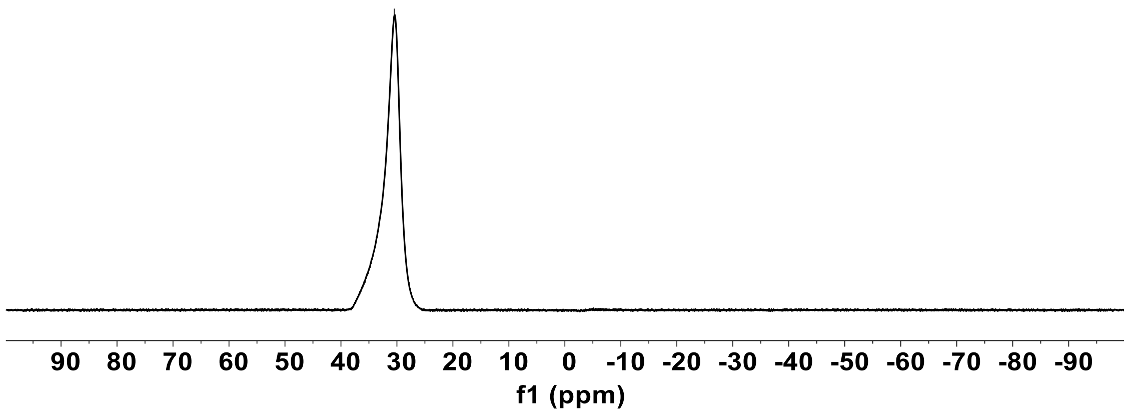

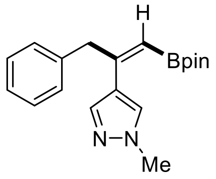

44

^{1}H NMR (600 MHz, $CDCl_3$)

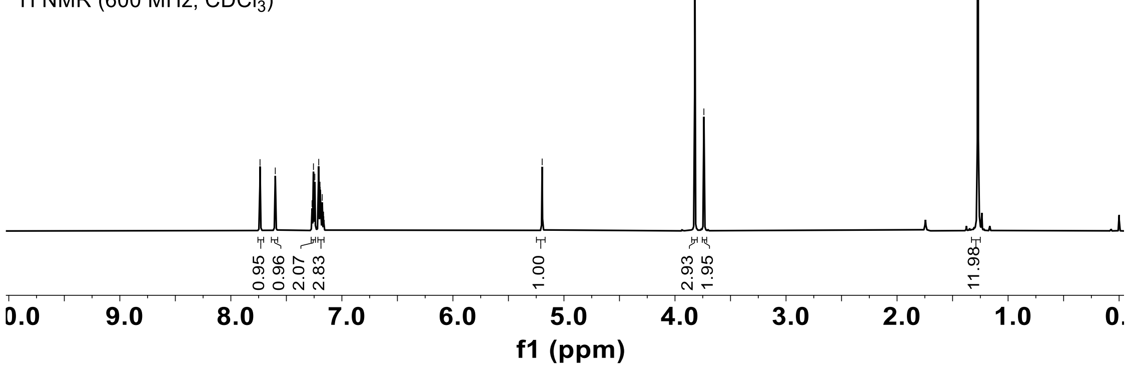

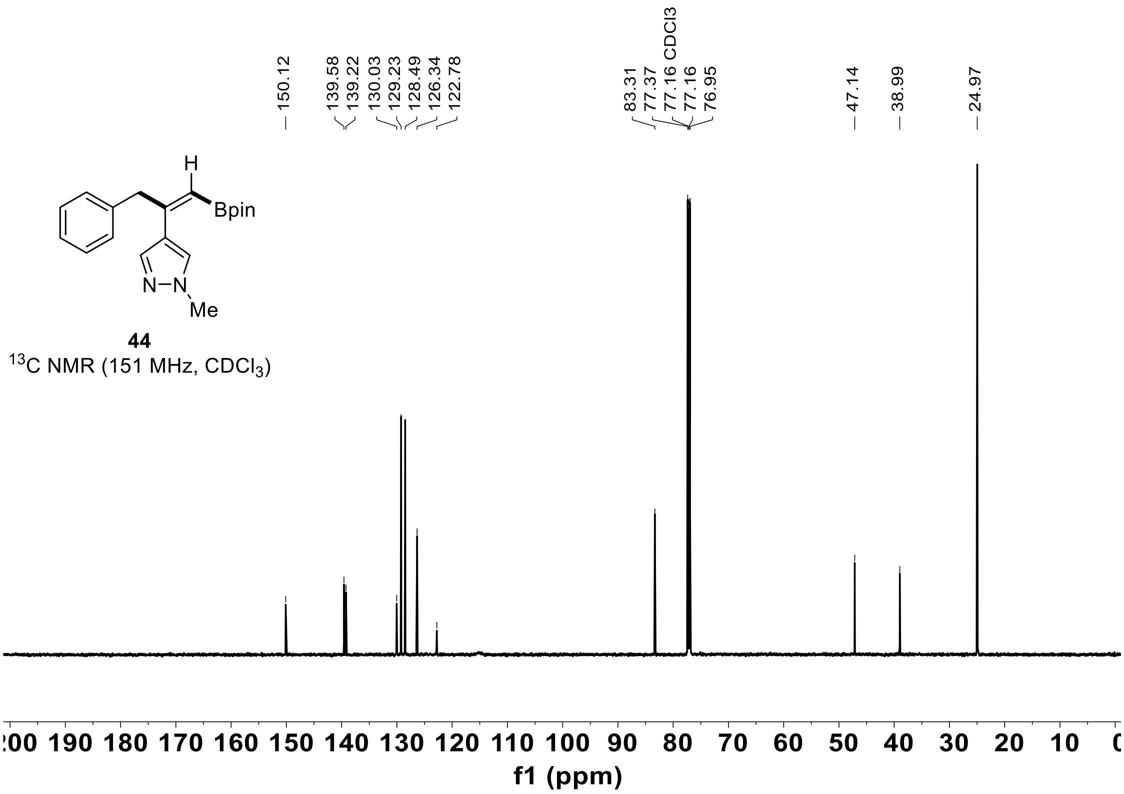

44

^{13}C NMR (151 MHz, $CDCl_3$)

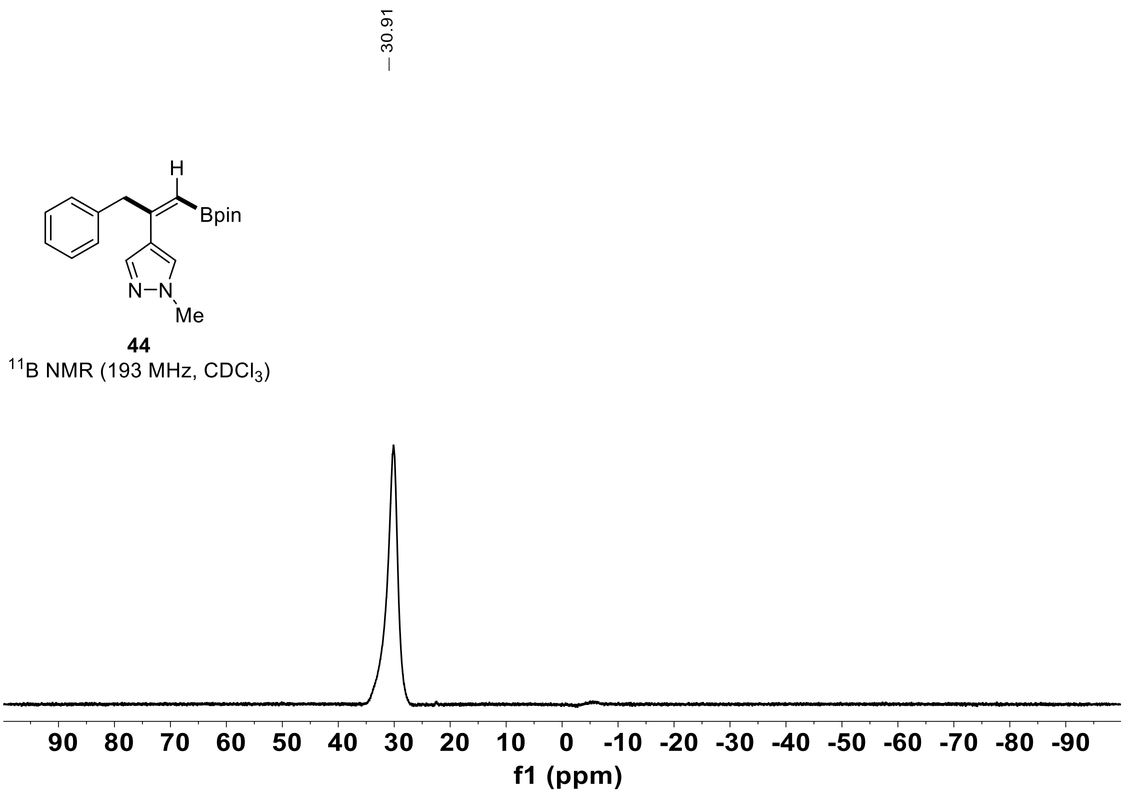

44

^{11}B NMR (193 MHz, $CDCl_3$)

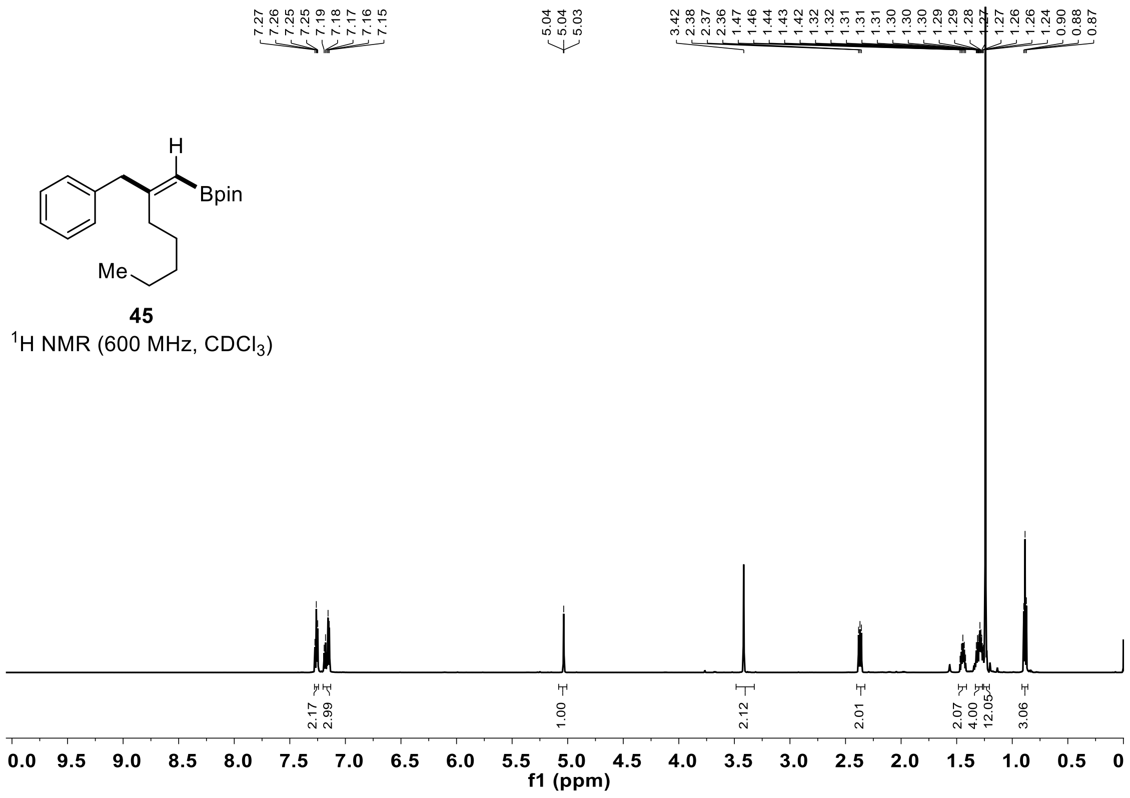
7.27 7.26 7.25 7.25 7.19 7.18 7.17 7.16 7.15
5.04 5.04 5.03
3.42 2.38 2.37 2.36 1.47 1.46 1.44 1.43 1.42 1.32 1.32 1.31 1.31 1.31 1.30 1.30 1.30 1.29 1.29 1.28 1.27 1.27 1.26 1.26 1.24 0.90 0.88 0.87
H
Bpin
Me
45
1H NMR (600 MHz, CDCl3)
2.17 2.99 1.00 2.12 2.01 2.07 4.00 12.05 3.06
f1 (ppm)

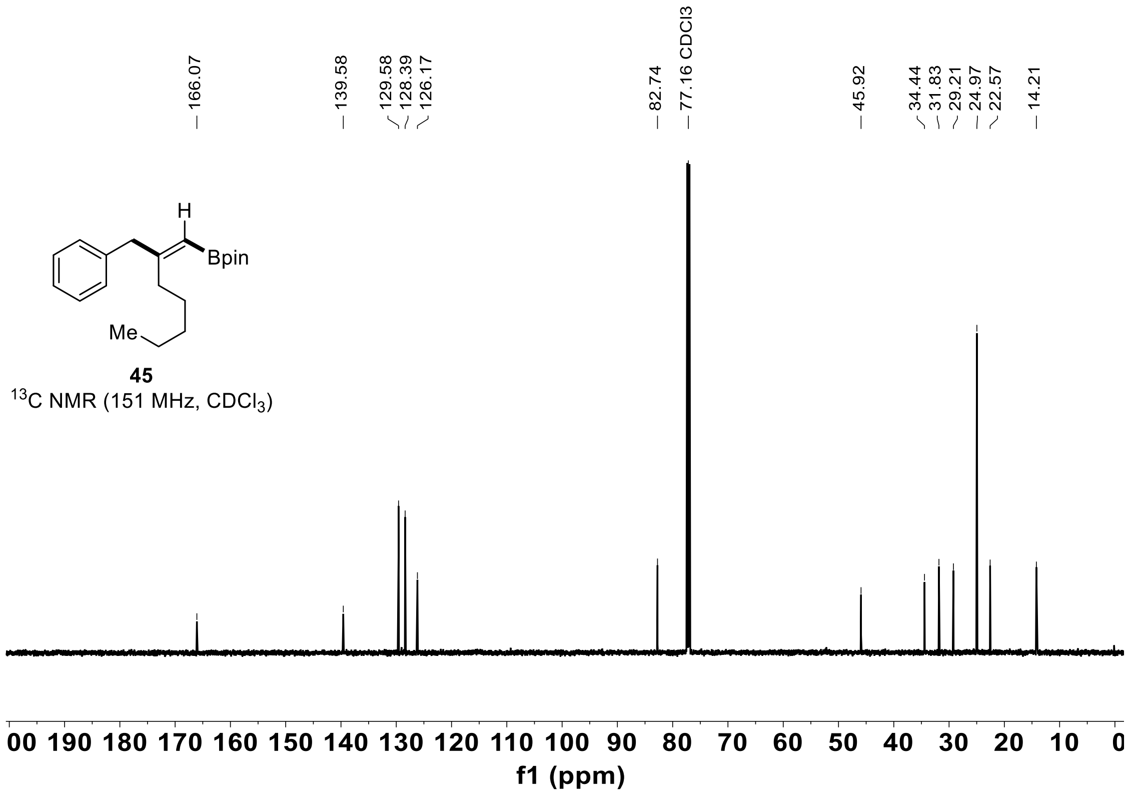
166.07
139.58 129.58 128.39 126.17
82.74 77.16 CDCl3
45.92 34.44 31.83 29.21 24.97 22.57 14.21
H
Bpin
Me
45
13C NMR (151 MHz, CDCl3)
f1 (ppm)

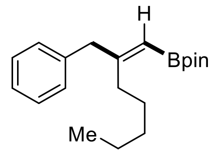

45

^{11}B NMR (193 MHz, $CDCl_3$)

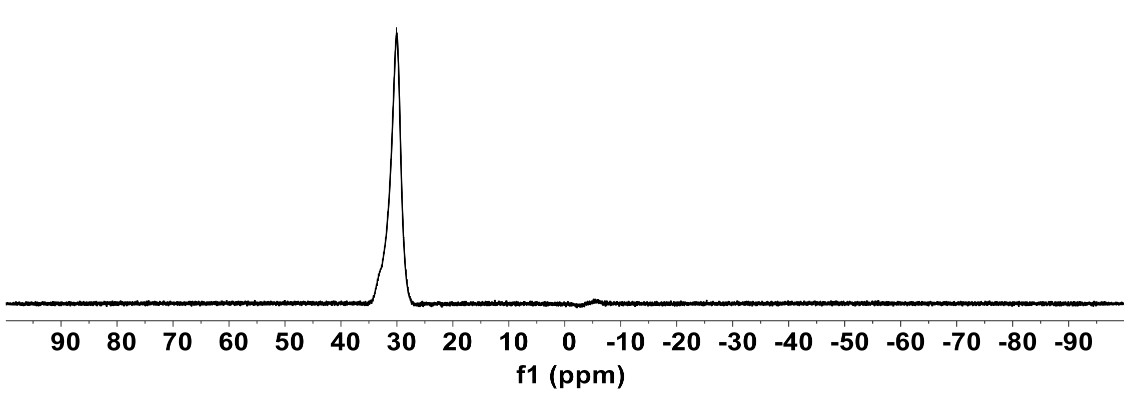

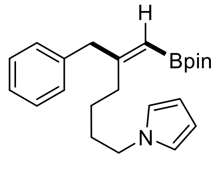

46

^{1}H NMR (600 MHz, $CDCl_3$)

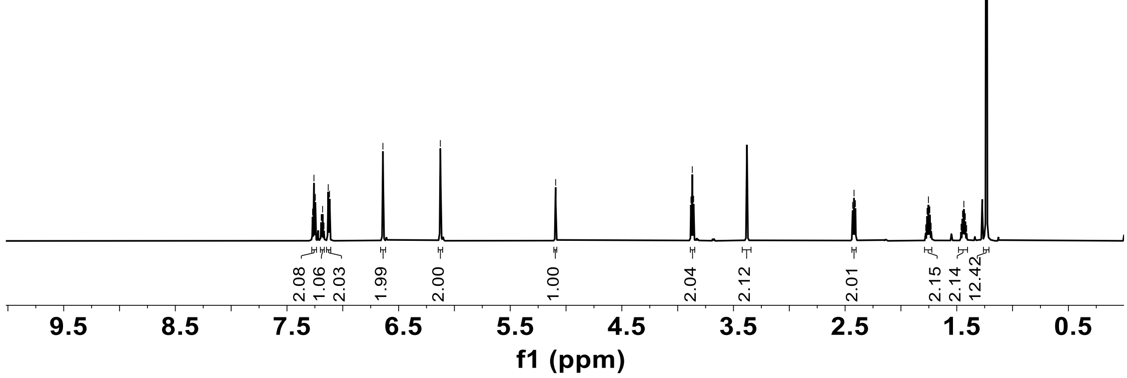

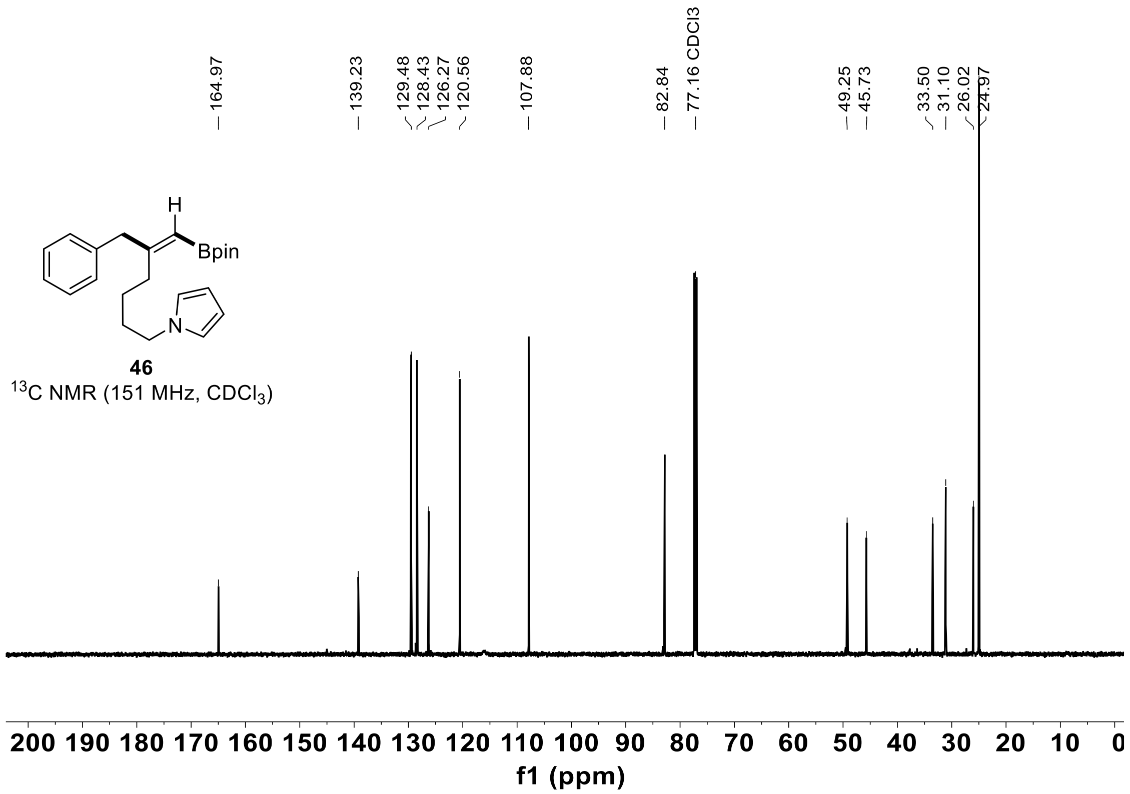
164.97
139.23
129.48
128.43
126.27
120.56
107.88
82.84
77.16 CDCl3
49.25
45.73
33.50
31.10
26.02
24.97
H
Bpin
N
46
^{13}C NMR (151 MHz, $CDCl_3$)
200 190 180 170 160 150 140 130 120 110 100 90 80 70 60 50 40 30 20 10 0
f1 (ppm)

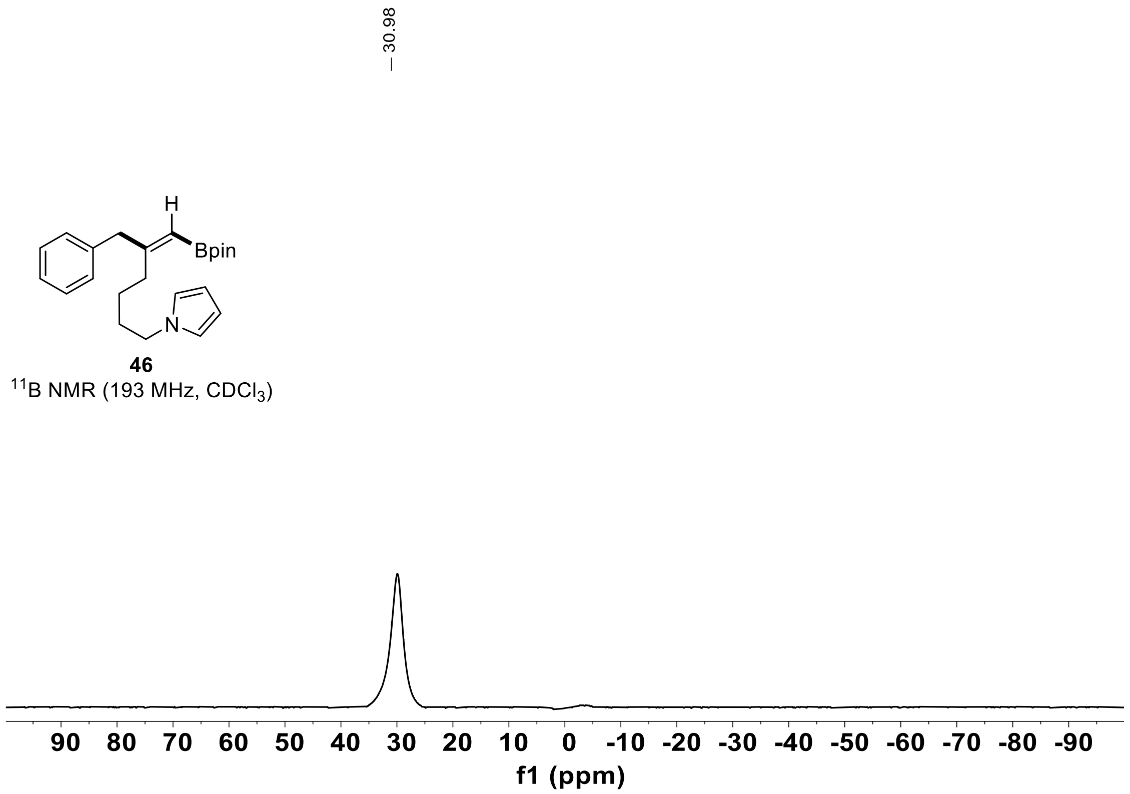
30.98
H
Bpin
N
46
^{11}B NMR (193 MHz, $CDCl_3$)
90 80 70 60 50 40 30 20 10 0 -10 -20 -30 -40 -50 -60 -70 -80 -90
f1 (ppm)

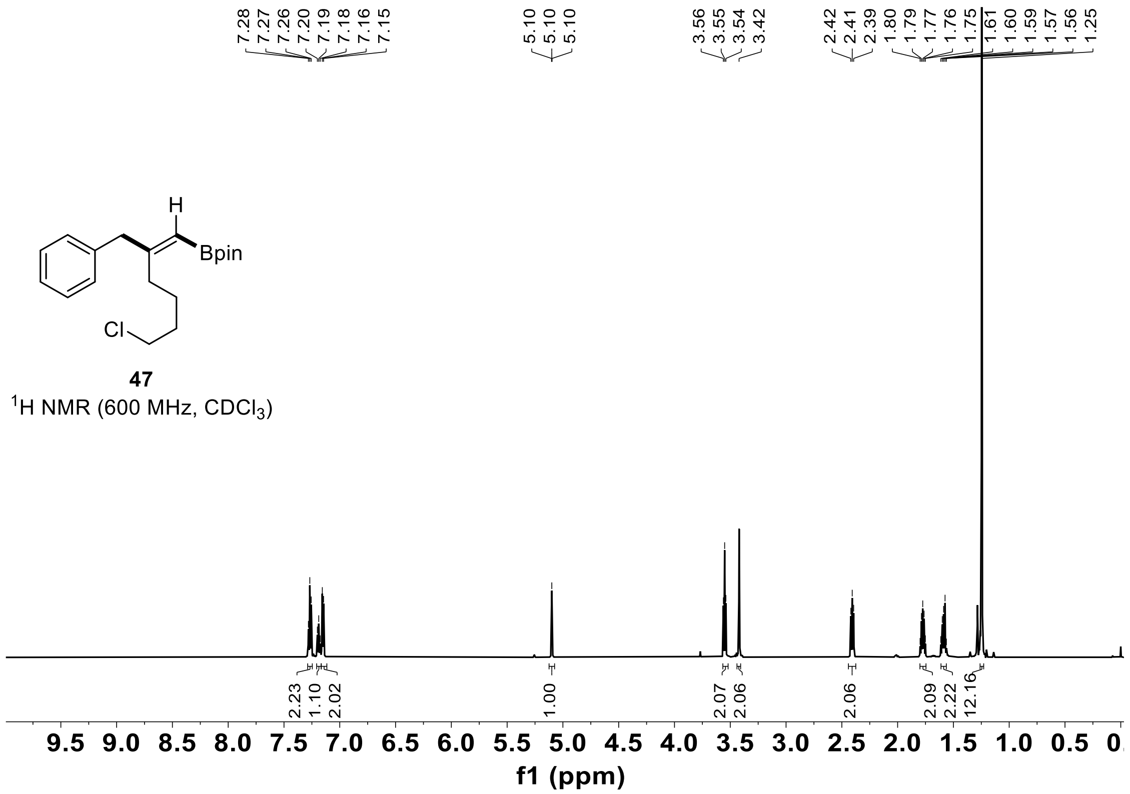
7.28 7.27 7.26 7.20 7.19 7.18 7.16 7.15
5.10 5.10 5.10
3.56 3.55 3.54 3.42
2.42 2.41 2.39 1.80 1.79 1.77 1.76 1.75 1.61 1.60 1.59 1.57 1.56 1.25
H
Bpin
Cl
47
^{1}H NMR (600 MHz, $CDCl_3$)
2.23 1.10 2.02
1.00
2.07 2.06
2.06
2.09 2.22 12.16
9.5 9.0 8.5 8.0 7.5 7.0 6.5 6.0 5.5 5.0 4.5 4.0 3.5 3.0 2.5 2.0 1.5 1.0 0.5 0.
f1 (ppm)

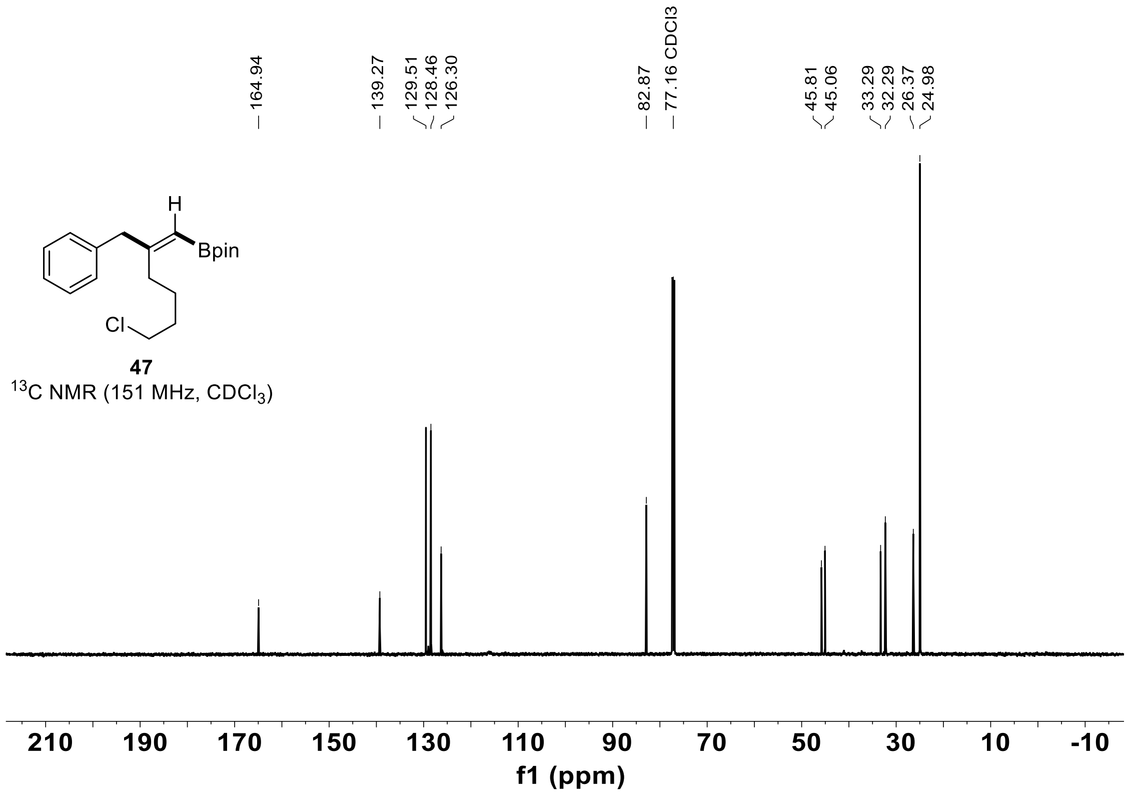
164.94
139.27
129.51 128.46 126.30
82.87
77.16 CDCl3
45.81 45.06
33.29 32.29 26.37 24.98
H
Bpin
Cl
47
^{13}C NMR (151 MHz, $CDCl_3$)
210 190 170 150 130 110 90 70 50 30 10 -10
f1 (ppm)

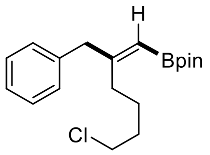

47

^{11}B NMR (193 MHz, $CDCl_3$)

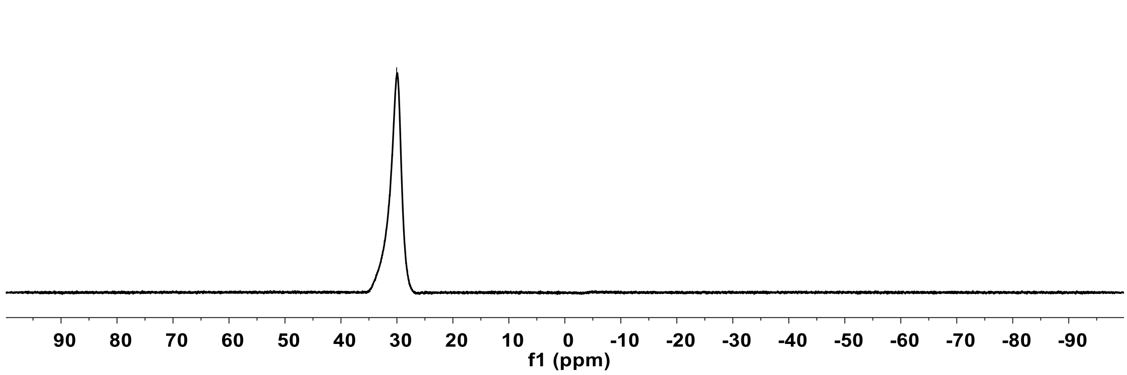

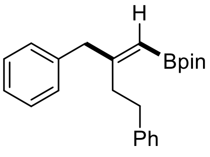

48

^{1}H NMR (600 MHz, $CDCl_3$)

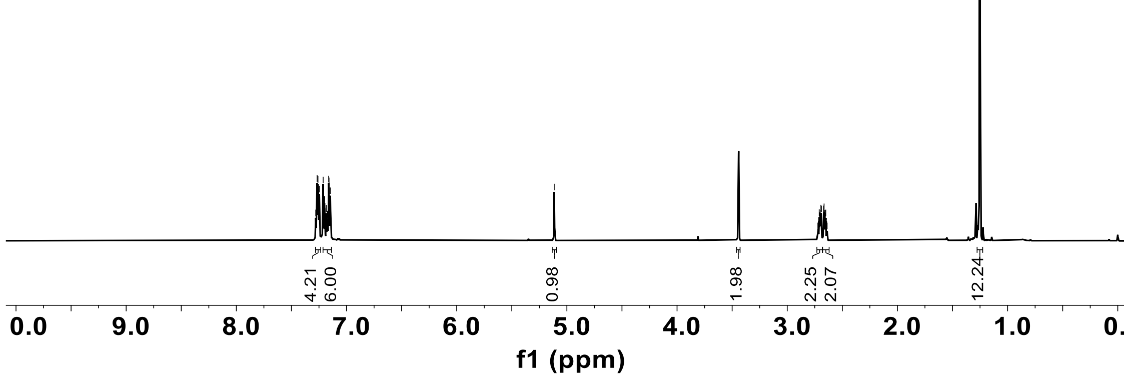

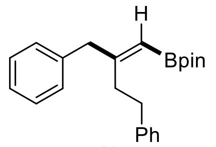

48

^{13}C NMR (151 MHz, $CDCl_3$)

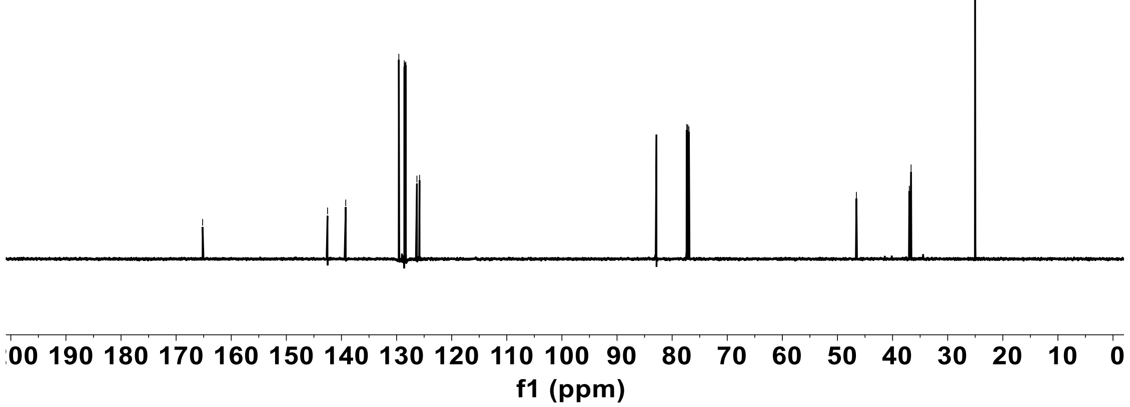

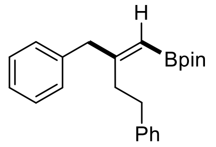

48

^{11}B NMR (193 MHz, $CDCl_3$)

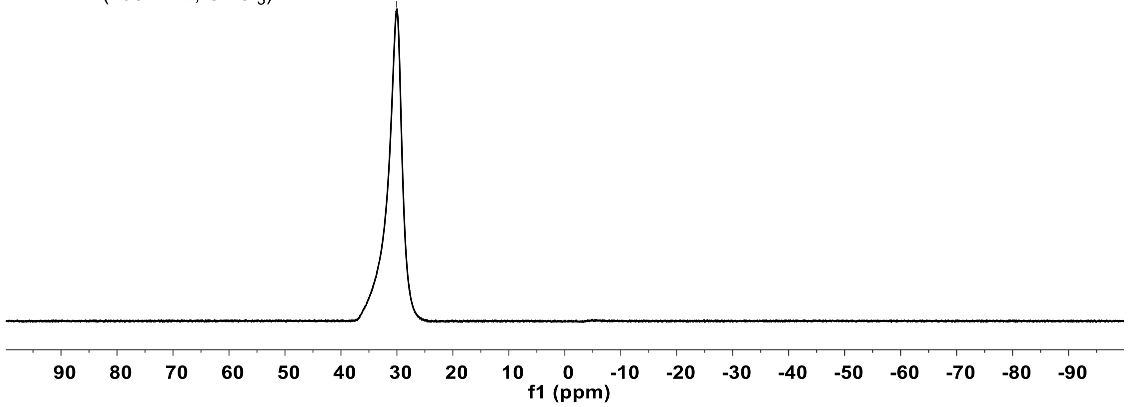

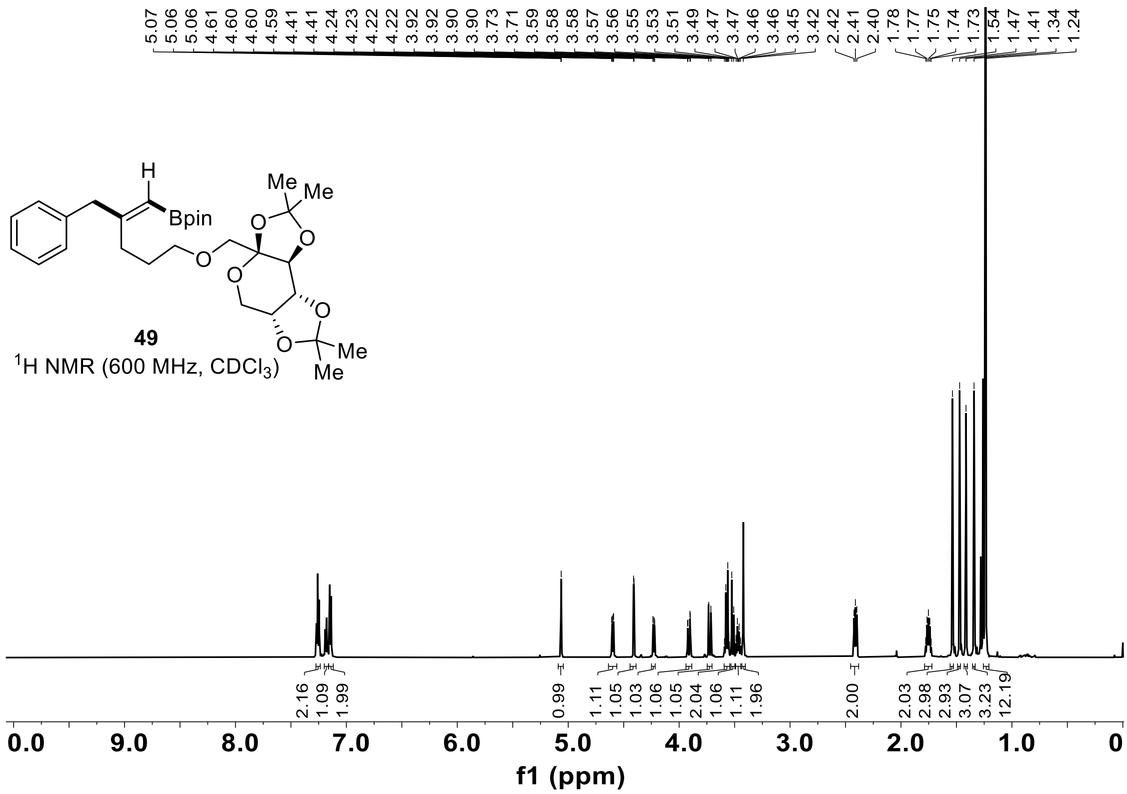

49
^{1}H NMR (600 MHz, $CDCl_3$)

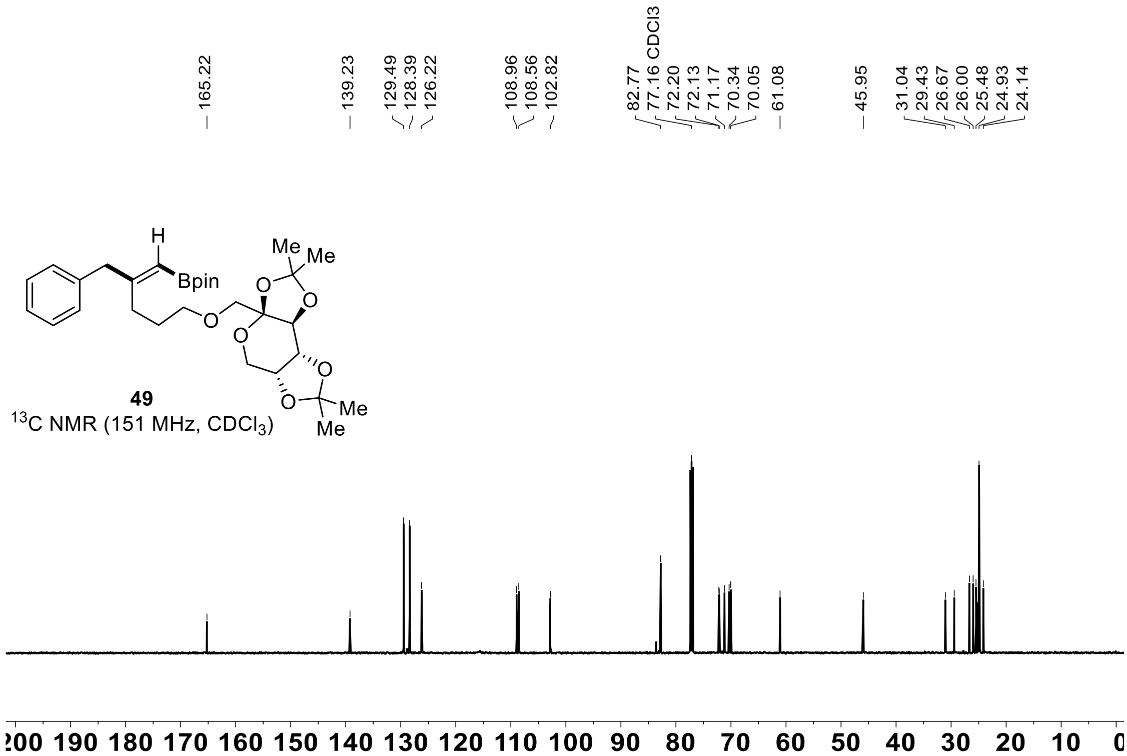

49
^{13}C NMR (151 MHz, $CDCl_3$)

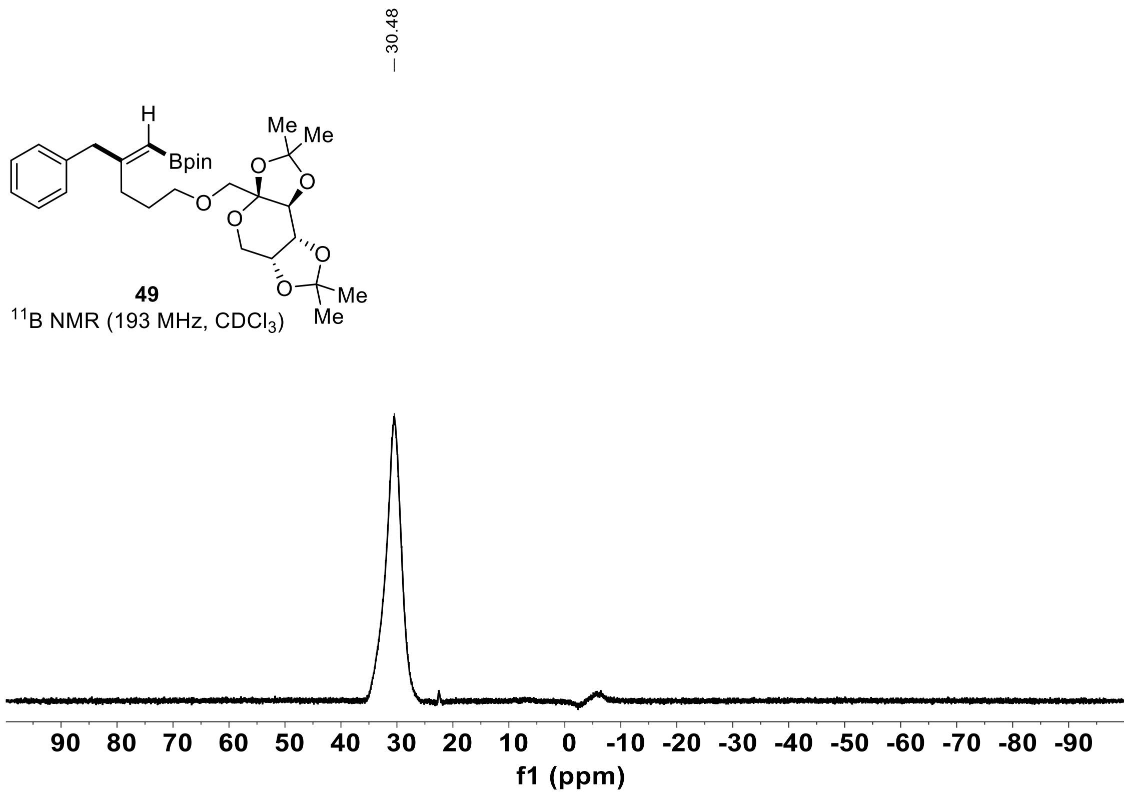

30.48
H
Bpin
Me
Me
O
O
O
O
O
O
Me
Me
49
^{11}B NMR (193 MHz, CDCl$_3$)
f1 (ppm)

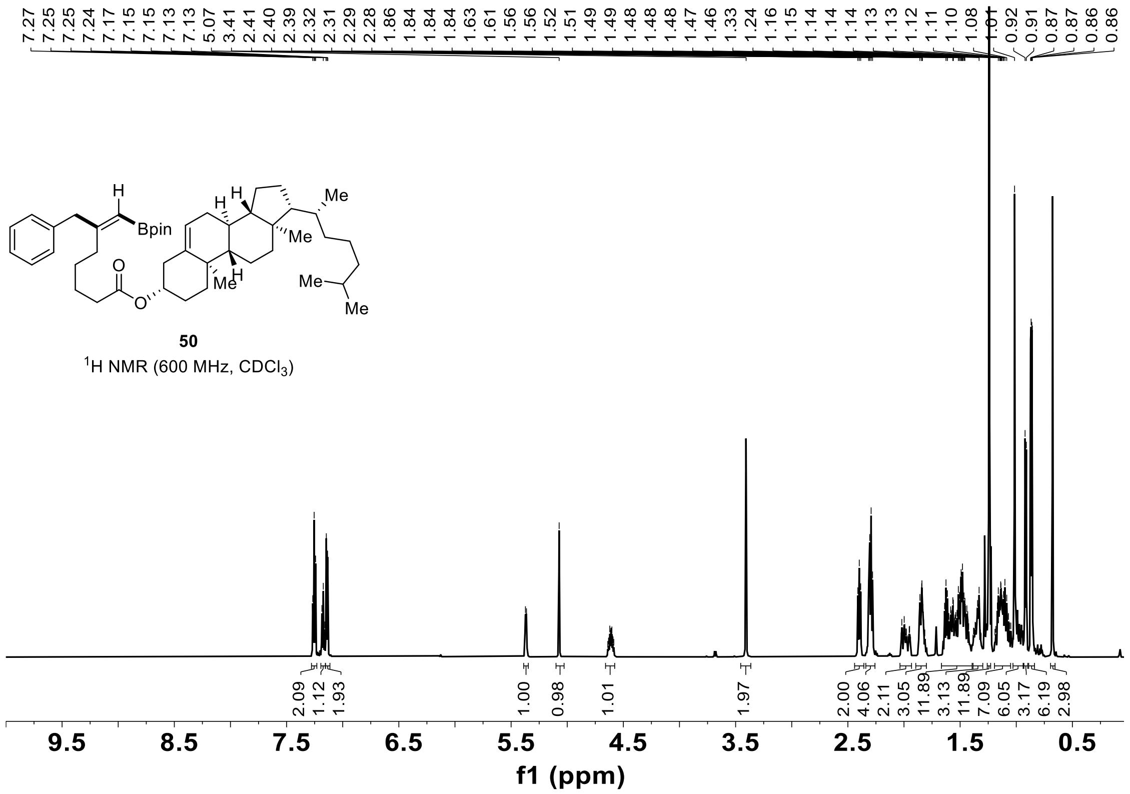

7.27 7.25 7.25 7.24 7.17 7.15 7.15 7.13 7.13 7.13 5.07 3.41 2.41 2.40 2.39 2.32 2.31 2.29 2.28 1.86 1.84 1.84 1.84 1.63 1.61 1.56 1.56 1.52 1.51 1.49 1.48 1.48 1.48 1.47 1.46 1.33 1.24 1.16 1.15 1.14 1.14 1.14 1.13 1.13 1.12 1.11 1.10 1.08 1.01 0.92 0.91 0.87 0.86 0.86
H
Bpin
O
O
H
H
H
H
Me
Me
Me
Me
Me
50
^{1}H NMR (600 MHz, CDCl$_3$)
2.09 1.12 1.93 1.00 0.98 1.01 1.97 2.00 4.06 2.11 3.05 11.89 3.13 11.89 7.09 6.05 3.17 6.19 2.98
f1 (ppm)

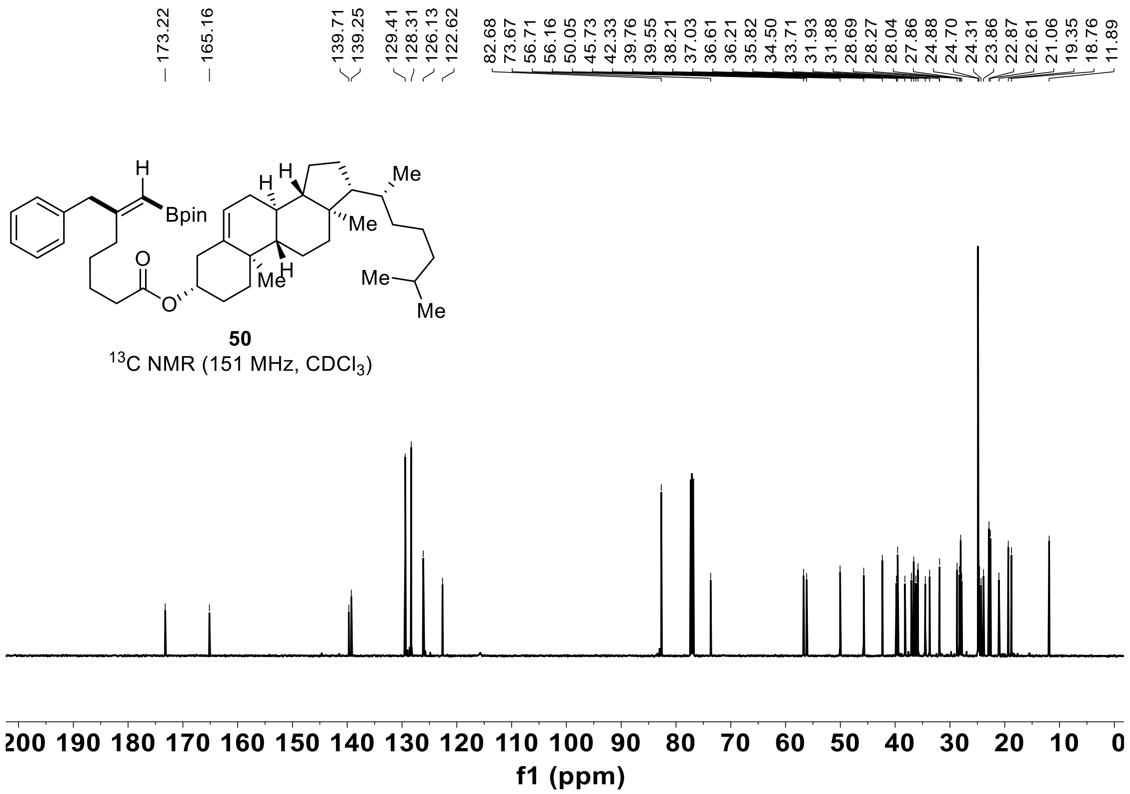
173.22
165.16
139.71
139.25
129.41
128.31
126.13
122.62
82.68
73.67
56.71
56.16
50.05
45.73
42.33
39.76
39.55
38.21
37.03
36.61
36.21
35.82
34.50
33.71
31.93
31.88
28.69
28.27
28.04
27.86
24.88
24.70
24.31
23.86
22.87
22.61
21.06
19.35
18.76
11.89
H
Bpin
H
H
Me
Me
O
O
Me
H
Me
Me
50
^{13}C NMR (151 MHz, $CDCl_3$)
200 190 180 170 160 150 140 130 120 110 100 90 80 70 60 50 40 30 20 10 0
f1 (ppm)

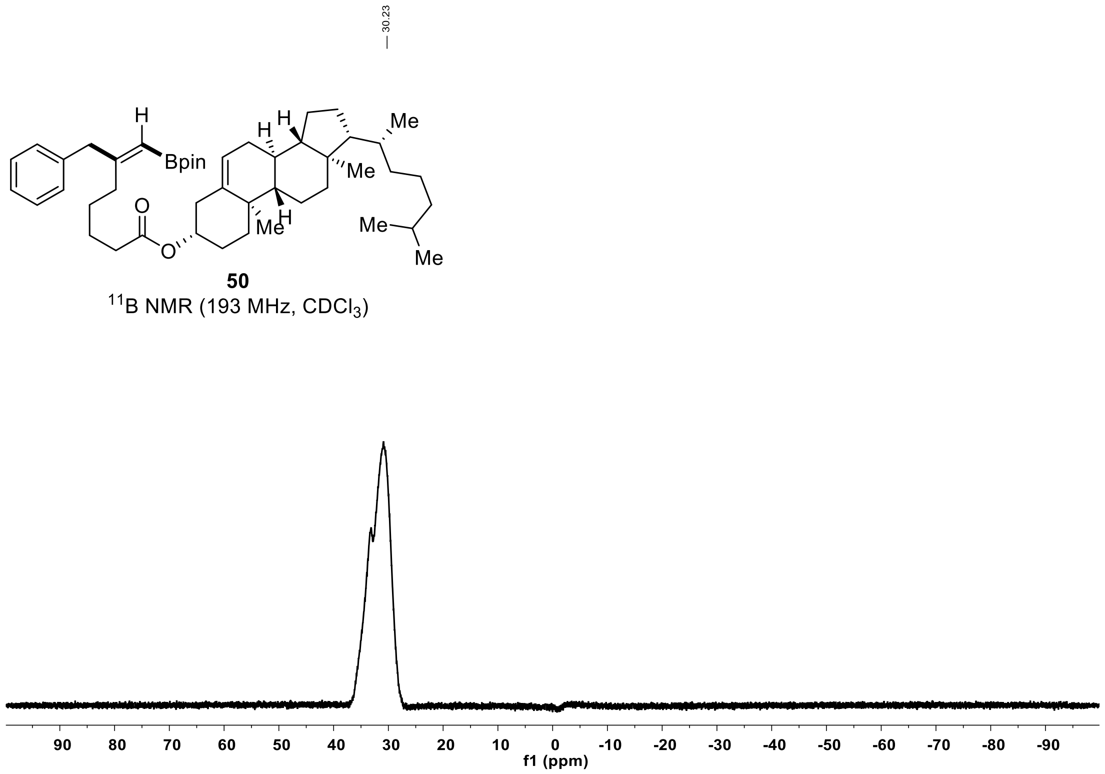
30.23
H
Bpin
H
H
Me
Me
O
O
Me
H
Me
Me
50
^{11}B NMR (193 MHz, $CDCl_3$)
90 80 70 60 50 40 30 20 10 0 -10 -20 -30 -40 -50 -60 -70 -80 -90
f1 (ppm)

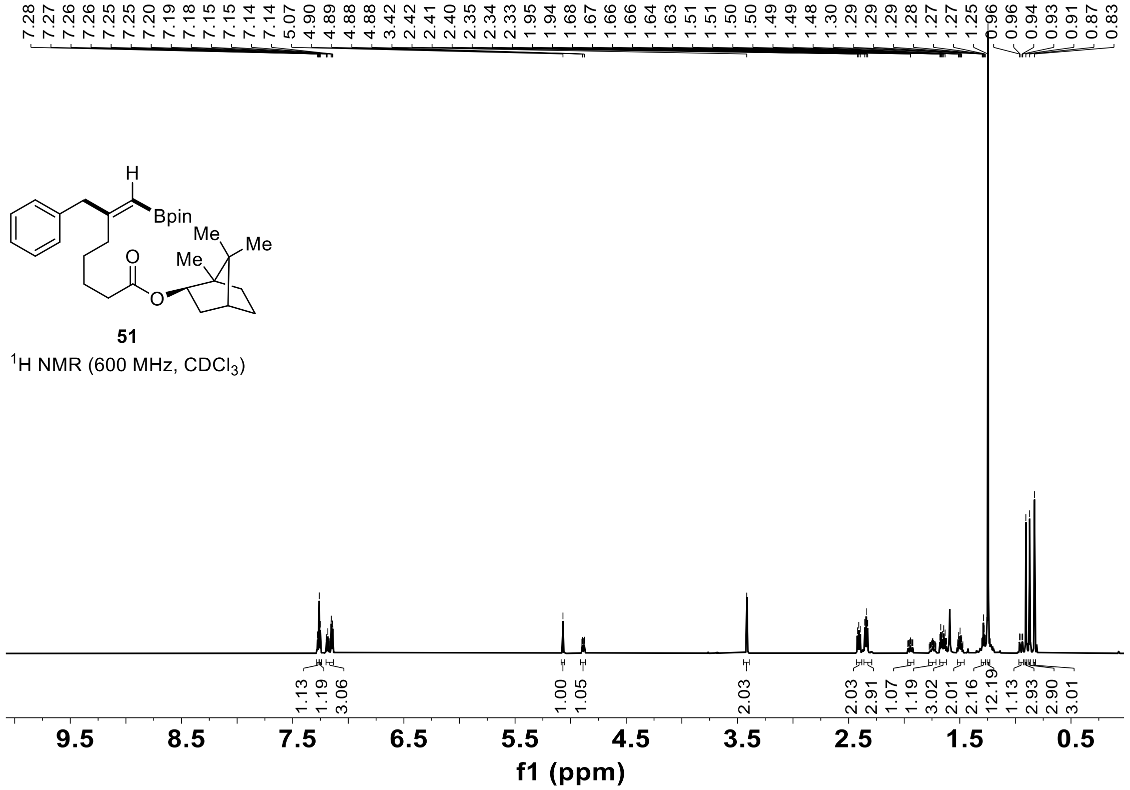

51

^{1}H NMR (600 MHz, $CDCl_3$)

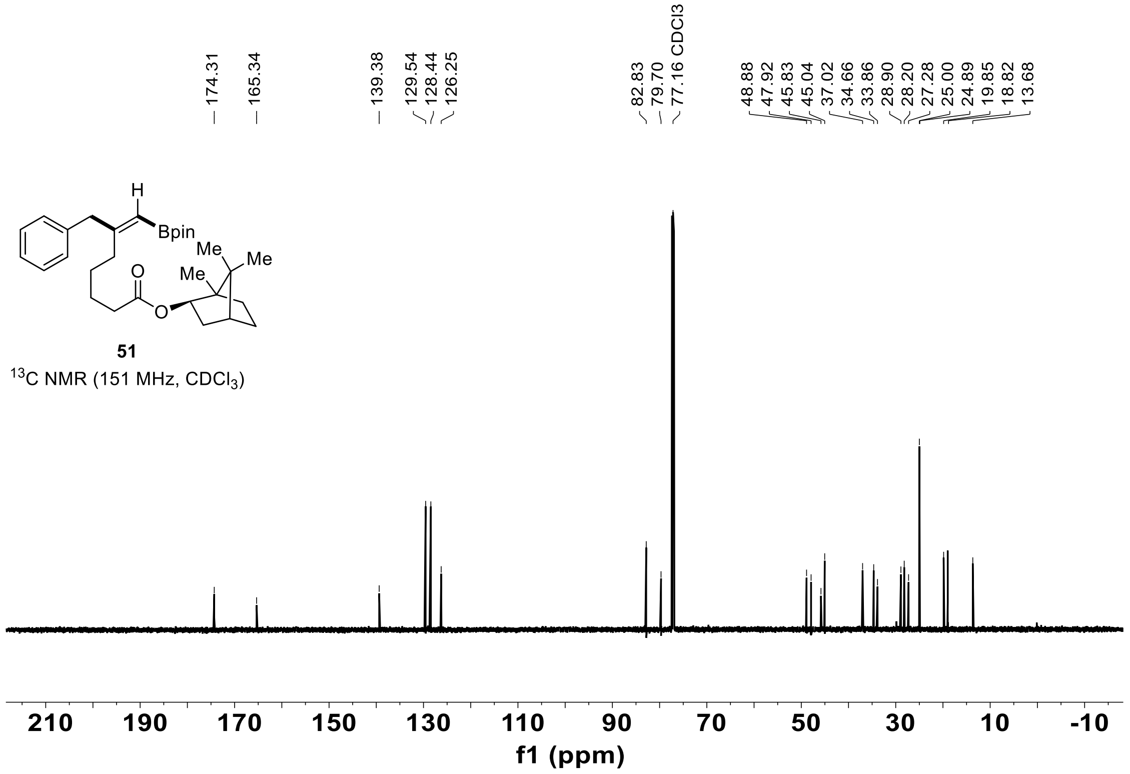

51

^{13}C NMR (151 MHz, $CDCl_3$)

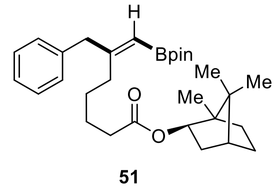

51
^{11}B NMR (193 MHz, $CDCl_3$)

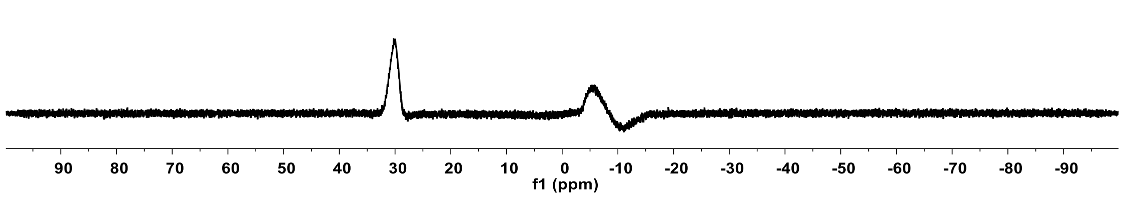

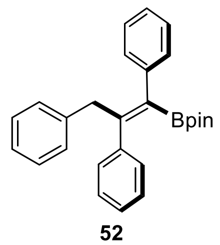

52
^{1}H NMR (600 MHz, $CDCl_3$)

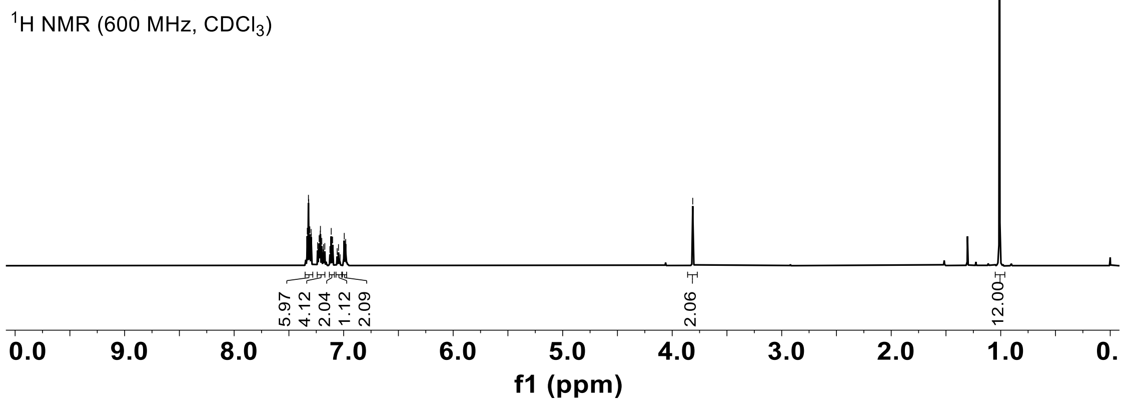

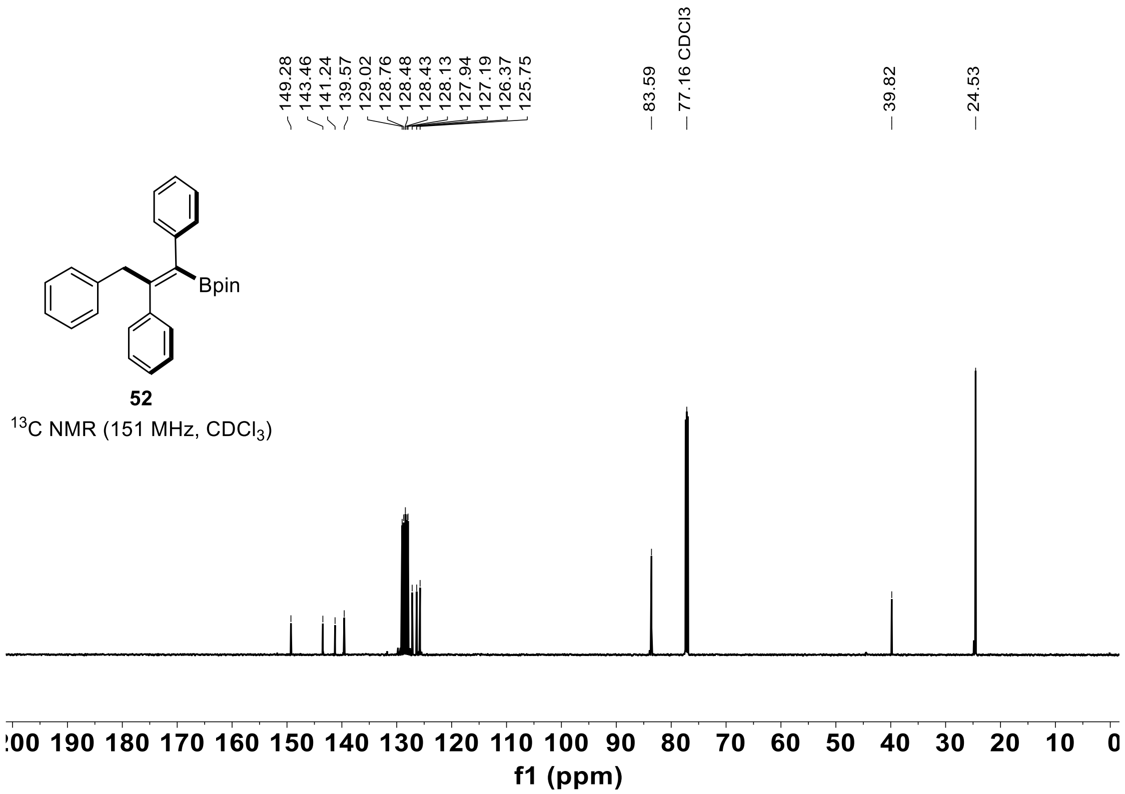
149.28
143.46
141.24
139.57
129.02
128.76
128.48
128.43
128.13
127.94
127.19
126.37
125.75
83.59
77.16 CDCl3
39.82
24.53
Bpin
52
^{13}C NMR (151 MHz, $CDCl_3$)
200 190 180 170 160 150 140 130 120 110 100 90 80 70 60 50 40 30 20 10 0
f1 (ppm)

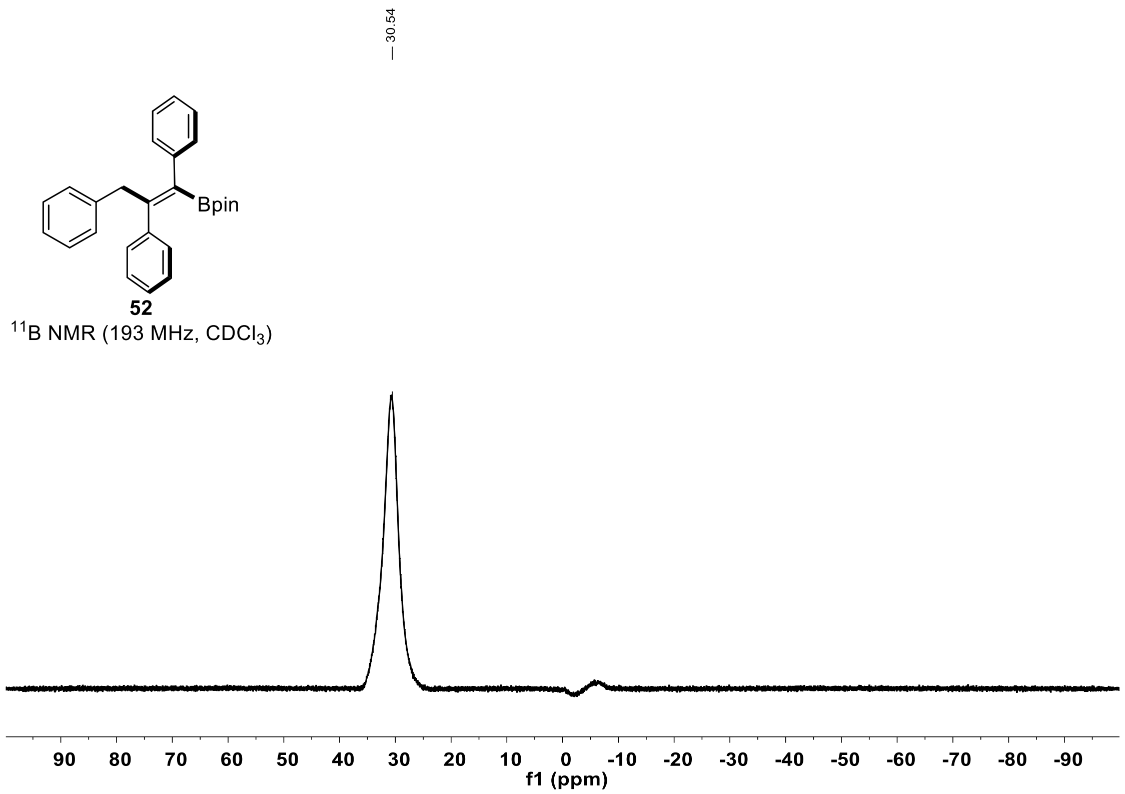
30.54
Bpin
52
^{11}B NMR (193 MHz, $CDCl_3$)
90 80 70 60 50 40 30 20 10 0 -10 -20 -30 -40 -50 -60 -70 -80 -90
f1 (ppm)

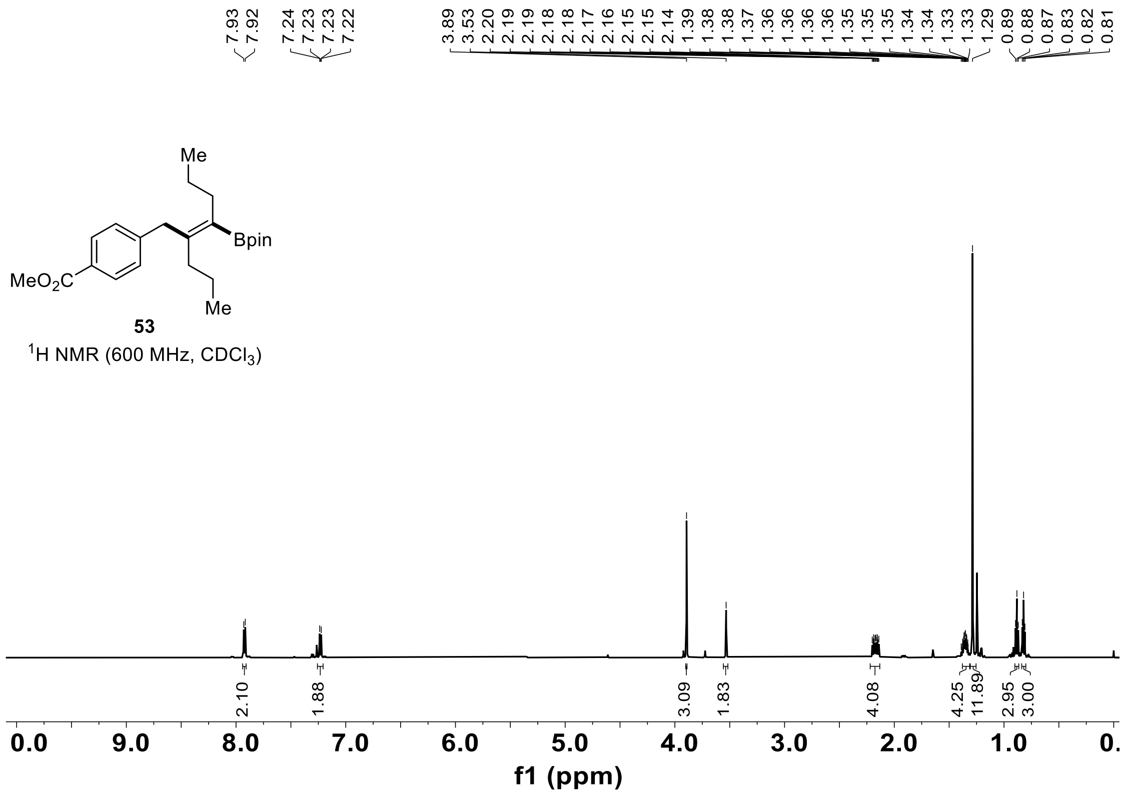
7.93 7.92 7.24 7.23 7.23 7.22
3.89 3.53 2.20 2.19 2.19 2.18 2.18 2.17 2.16 2.15 2.15 2.14 1.39 1.38 1.38 1.37 1.36 1.36 1.36 1.36 1.35 1.35 1.35 1.34 1.34 1.33 1.33 1.29 0.89 0.88 0.87 0.83 0.82 0.81
Me
Bpin
MeO2C
Me
53
1H NMR (600 MHz, CDCl3)
2.10 1.88 3.09 1.83 4.08 4.25 11.89 2.95 3.00
f1 (ppm)

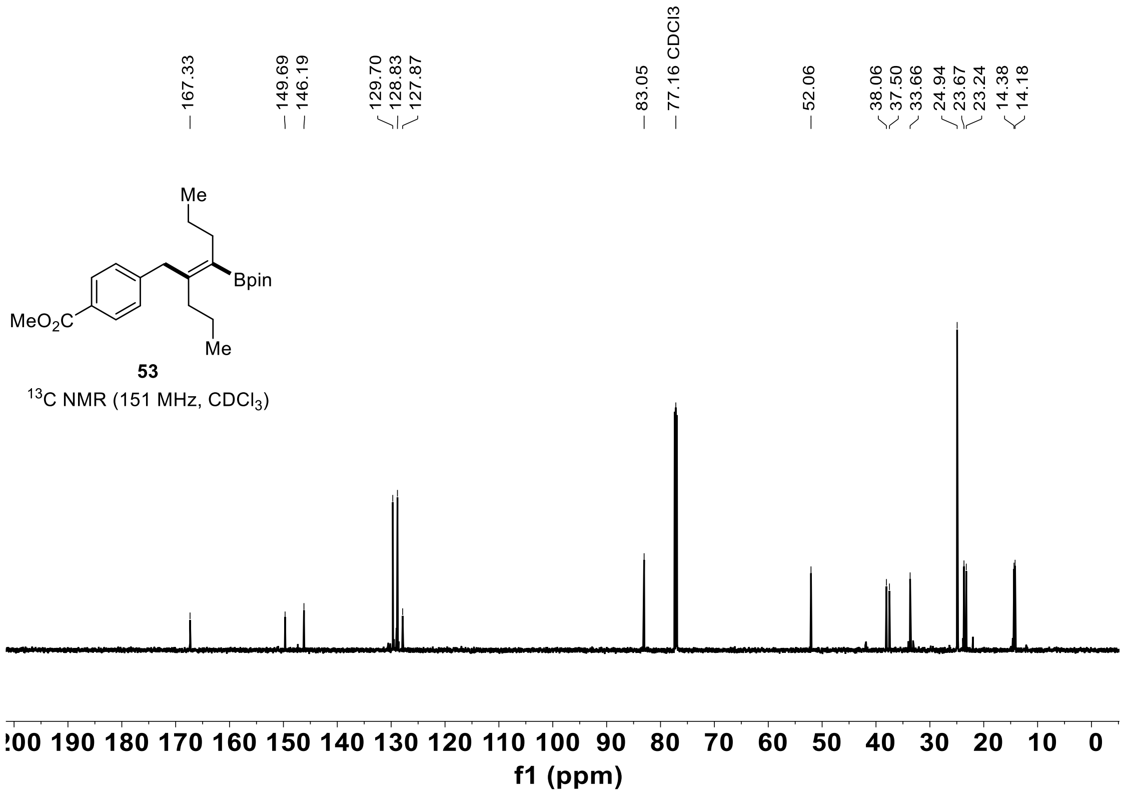
167.33 149.69 146.19 129.70 128.83 127.87 83.05 77.16 CDCl3 52.06 38.06 37.50 33.66 24.94 23.67 23.24 14.38 14.18
Me
Bpin
MeO2C
Me
53
13C NMR (151 MHz, CDCl3)
f1 (ppm)

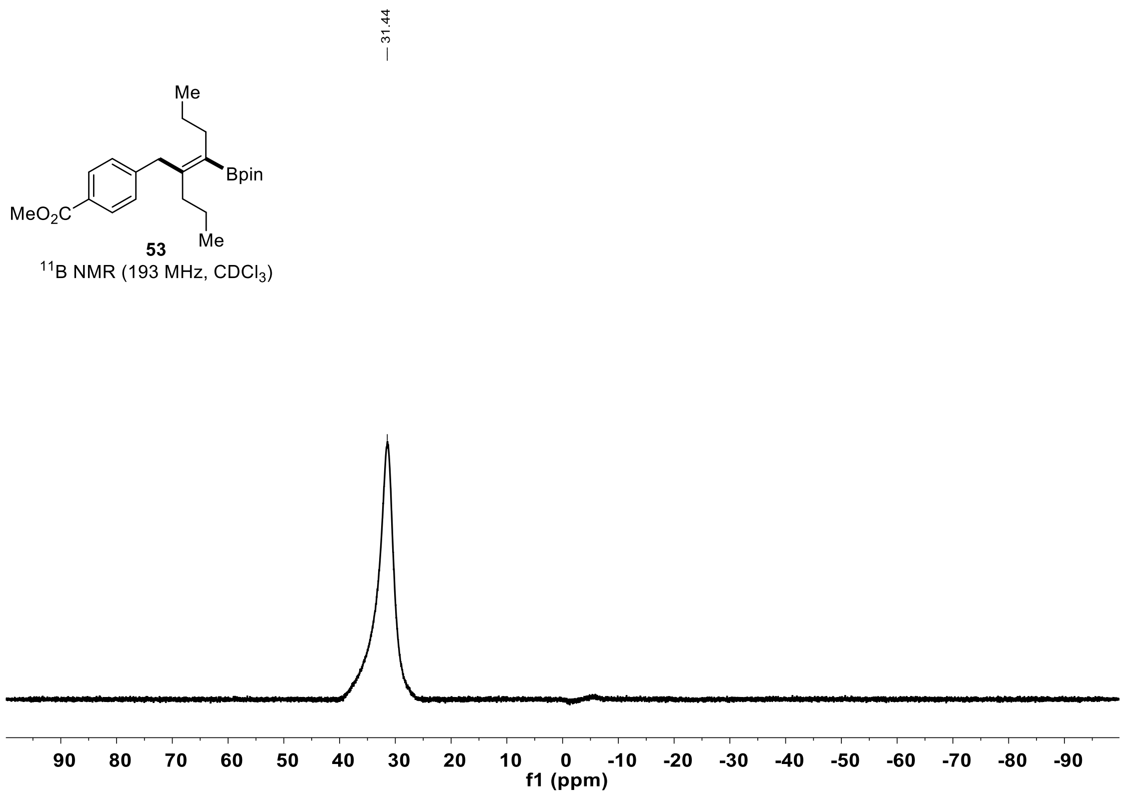
31.44
Me
Bpin
MeO_2C
Me
53
^{11}B NMR (193 MHz, $CDCl_3$)
90 80 70 60 50 40 30 20 10 0 -10 -20 -30 -40 -50 -60 -70 -80 -90
f1 (ppm)

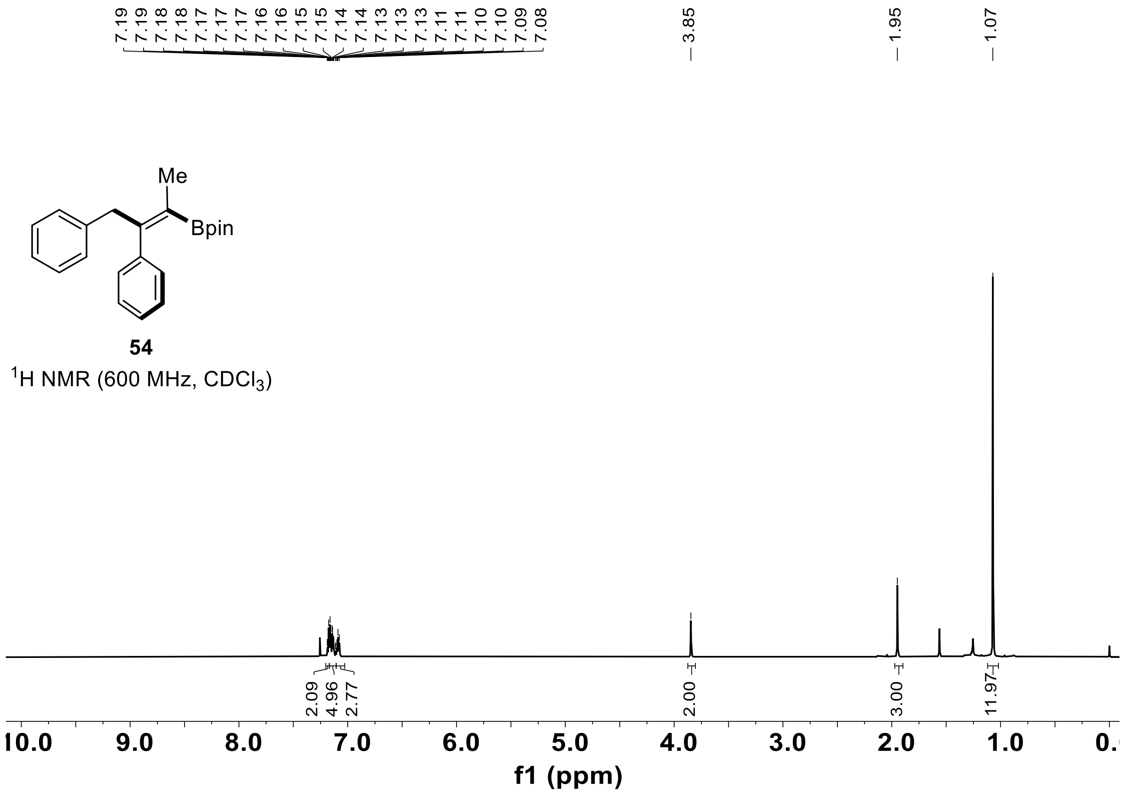
7.19 7.19 7.18 7.18 7.17 7.17 7.17 7.16 7.16 7.15 7.15 7.14 7.14 7.13 7.13 7.13 7.11 7.11 7.10 7.10 7.09 7.08
3.85
1.95
1.07
Me
Bpin
54
^1H NMR (600 MHz, $CDCl_3$)
2.09
4.96
2.77
2.00
3.00
11.97
10.0 9.0 8.0 7.0 6.0 5.0 4.0 3.0 2.0 1.0 0.
f1 (ppm)

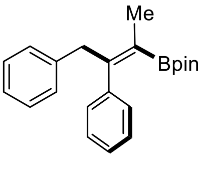

54

^{13}C NMR (151 MHz, $CDCl_3$)

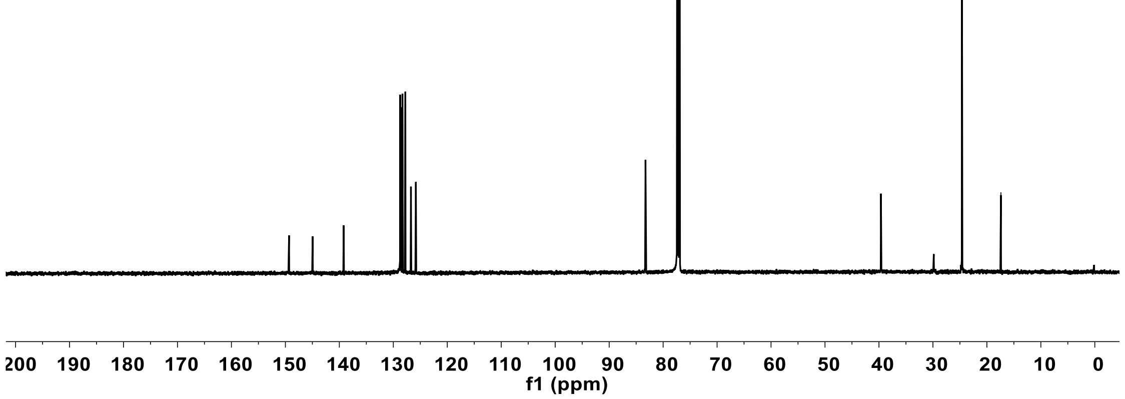

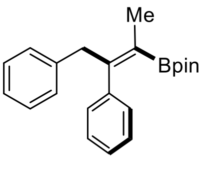

54

^{11}B NMR (193 MHz, $CDCl_3$)

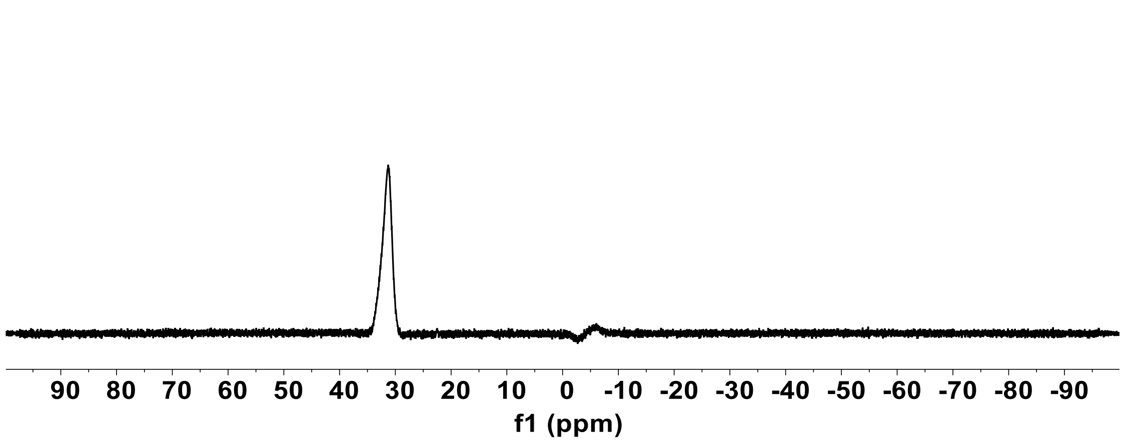

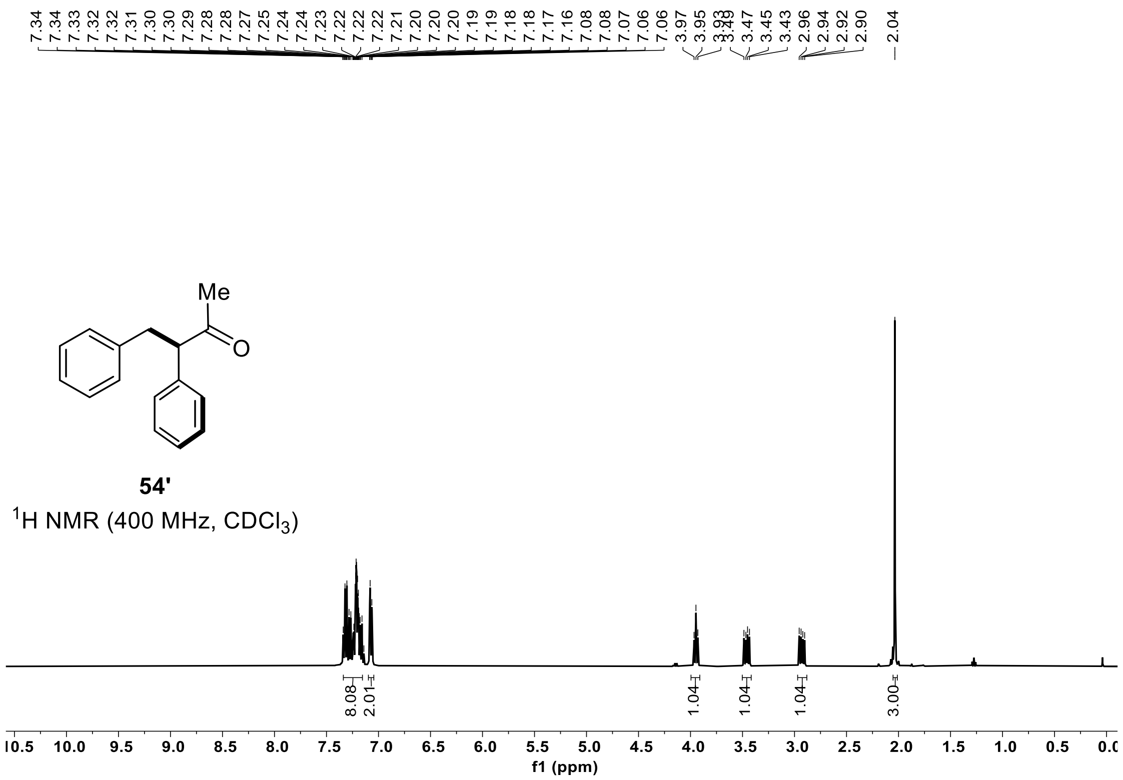
7.34 7.34 7.33 7.32 7.32 7.31 7.30 7.30 7.29 7.28 7.28 7.27 7.25 7.24 7.24 7.23 7.22 7.22 7.22 7.22 7.21 7.20 7.20 7.20 7.19 7.19 7.18 7.18 7.17 7.16 7.08 7.08 7.07 7.06 7.06 3.97 3.95 3.93 3.49 3.47 3.45 3.43 2.96 2.94 2.92 2.90 2.04
Me
O
54'
1H NMR (400 MHz, CDCl3)
8.08
2.01
1.04
1.04
1.04
3.00
10.5 10.0 9.5 9.0 8.5 8.0 7.5 7.0 6.5 6.0 5.5 5.0 4.5 4.0 3.5 3.0 2.5 2.0 1.5 1.0 0.5 0.0
f1 (ppm)

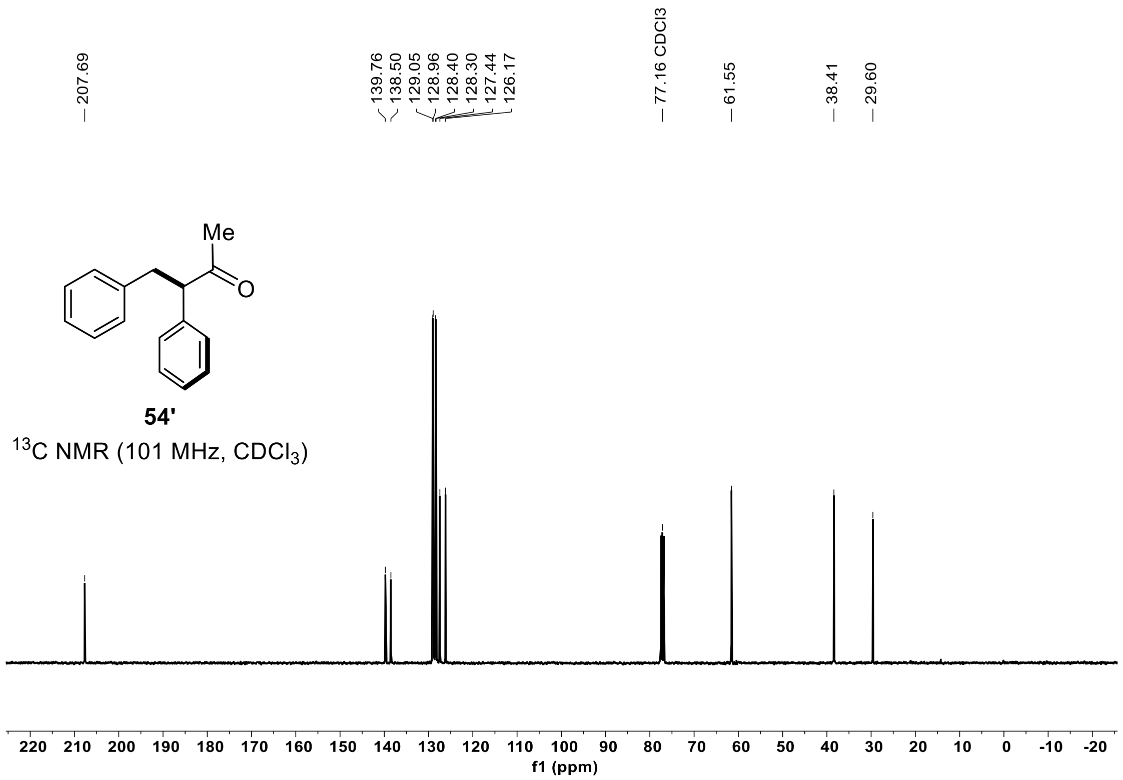
207.69
139.76 138.50 129.05 128.96 128.40 128.30 127.44 126.17
77.16 CDCl3
61.55
38.41
29.60
Me
O
54'
13C NMR (101 MHz, CDCl3)
220 210 200 190 180 170 160 150 140 130 120 110 100 90 80 70 60 50 40 30 20 10 0 -10 -20
f1 (ppm)

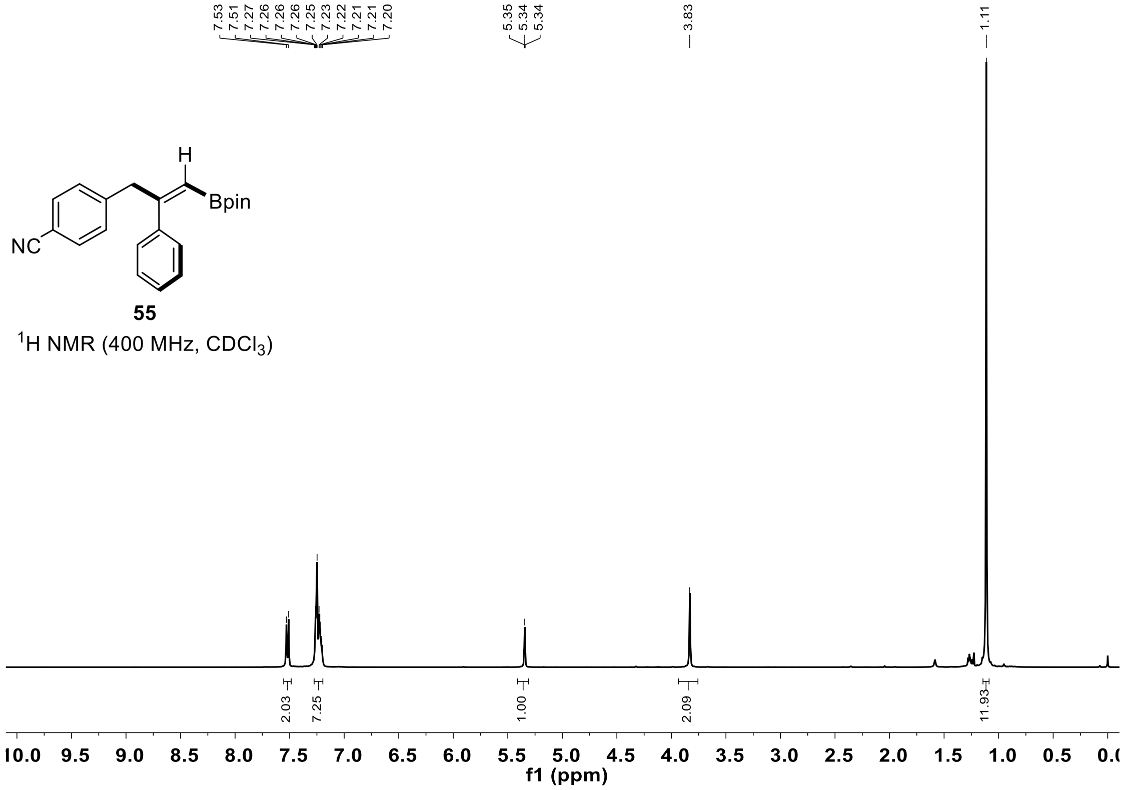

55

^{1}H NMR (400 MHz, $CDCl_3$)

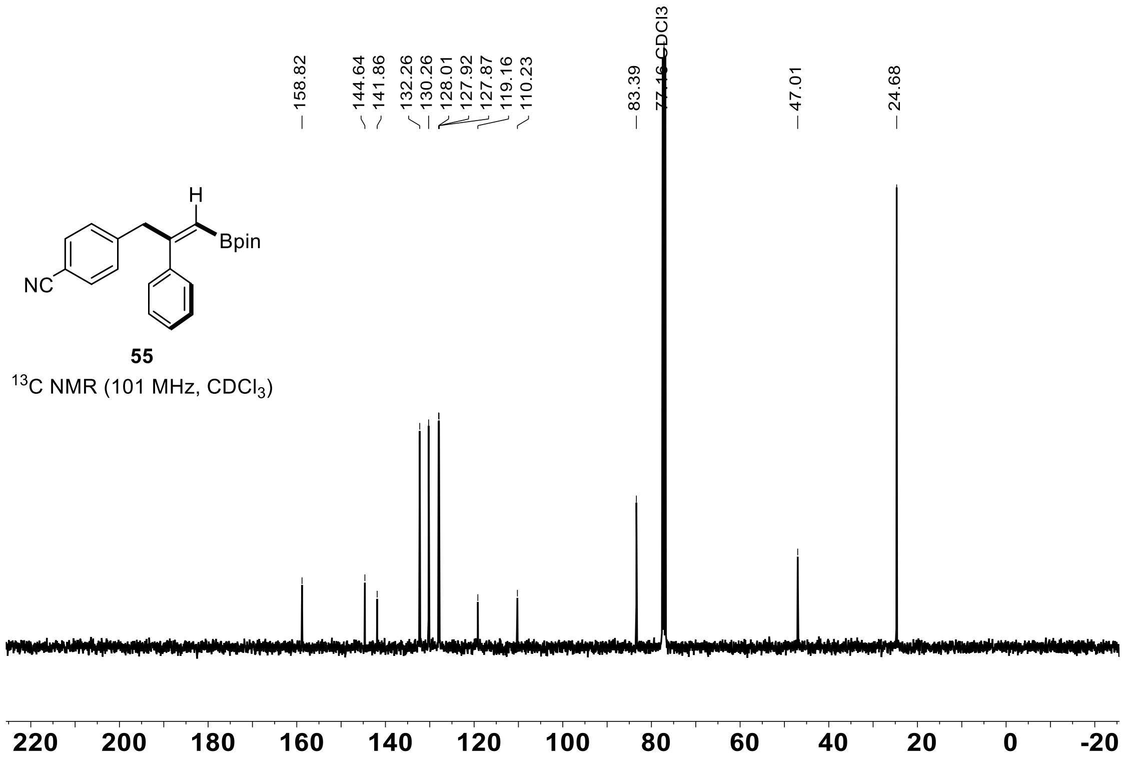

55

^{13}C NMR (101 MHz, $CDCl_3$)

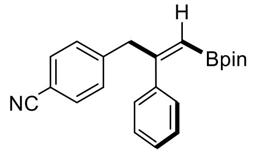

55

^{11}B NMR (193 MHz, $CDCl_3$)

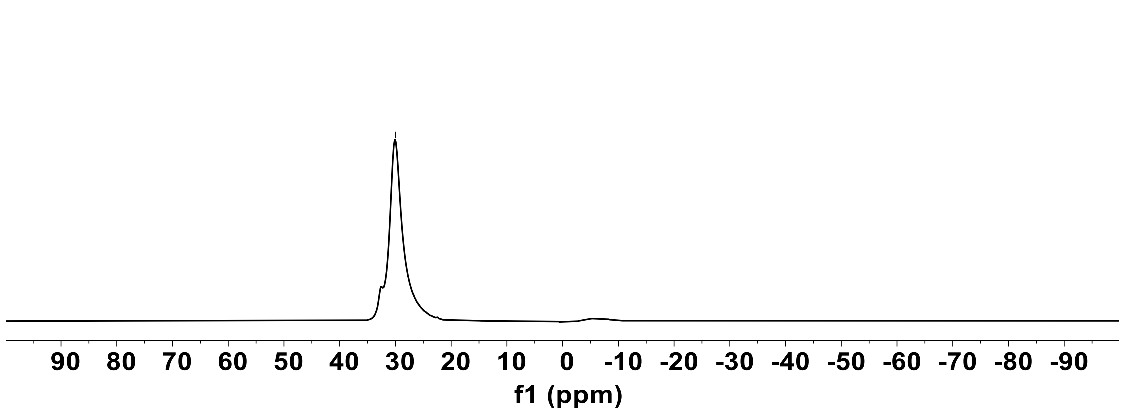

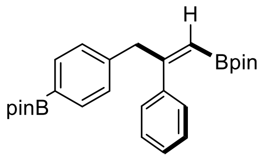

56

^{1}H NMR (600 MHz, $CDCl_3$)

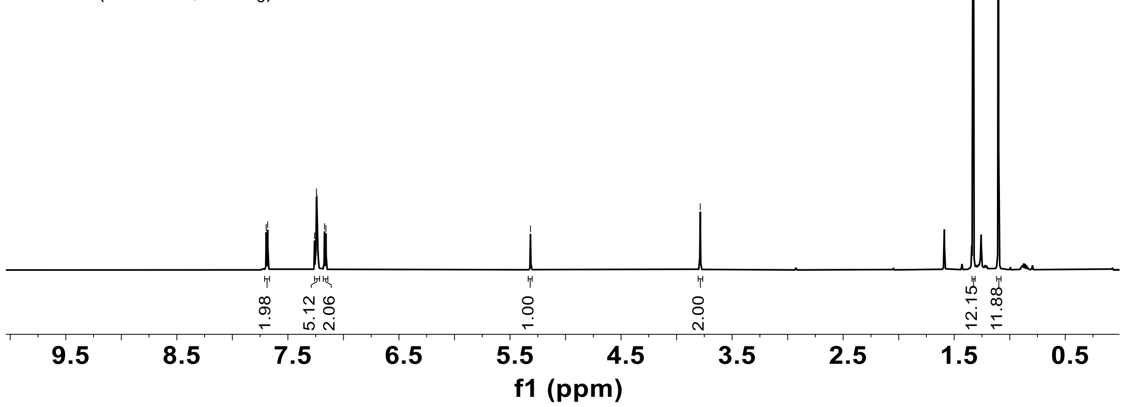

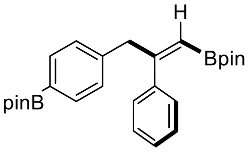

56

^{13}C NMR (151 MHz, $CDCl_3$)

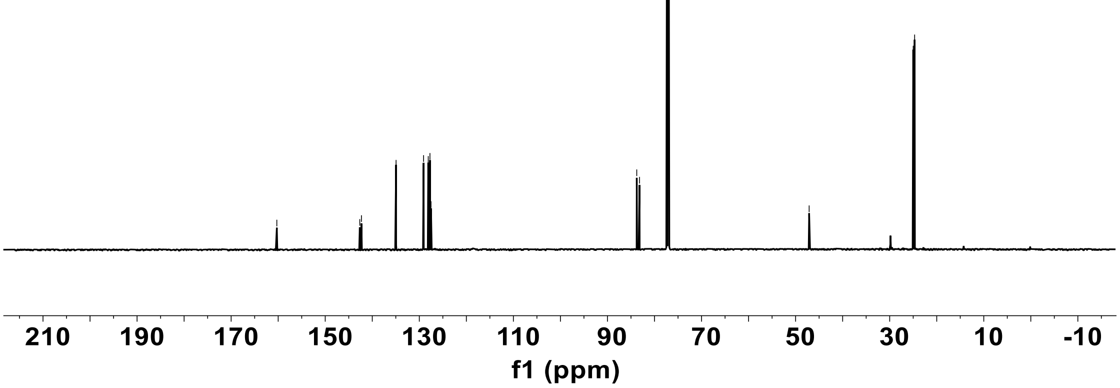

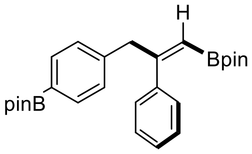

56

^{11}B NMR (193 MHz, $CDCl_3$)

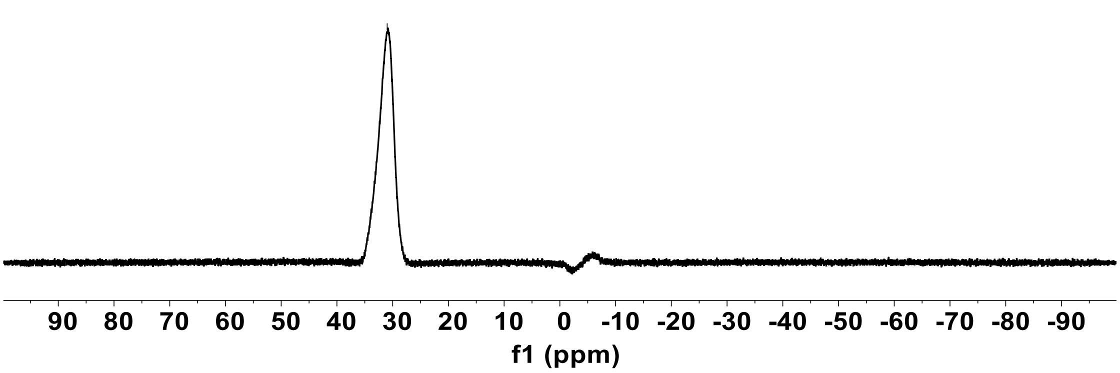

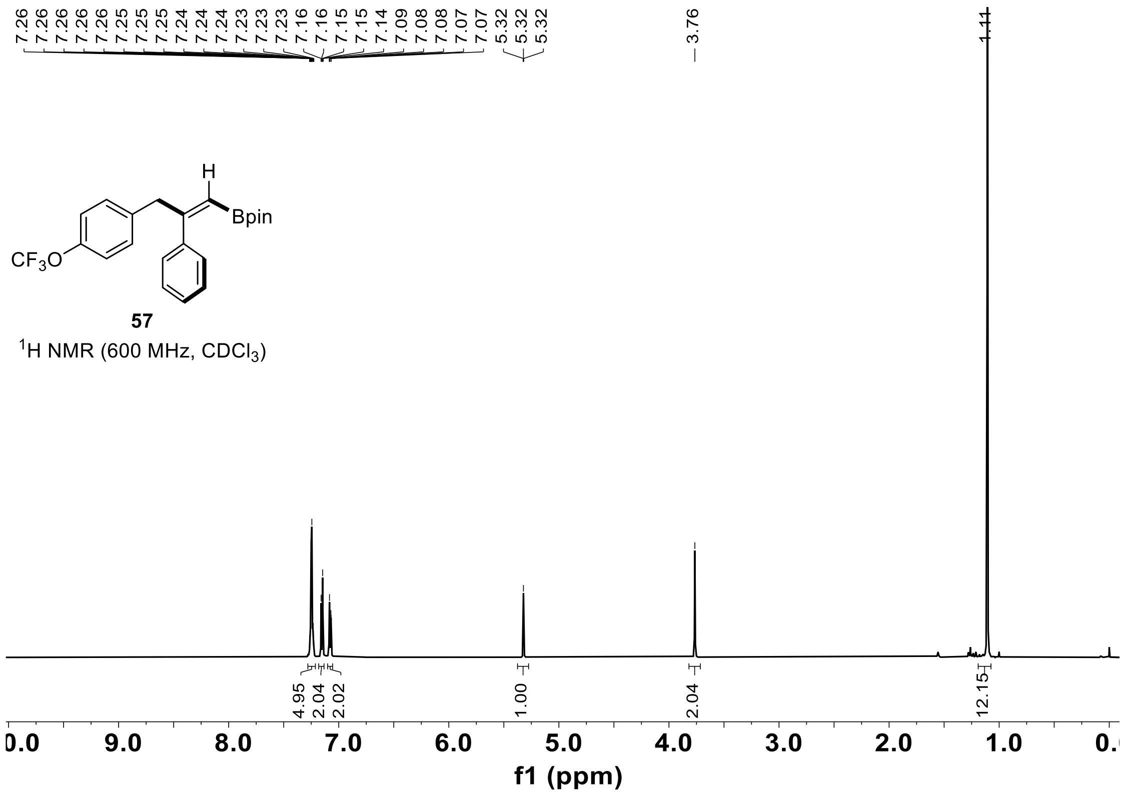

57

^{1}H NMR (600 MHz, $CDCl_3$)

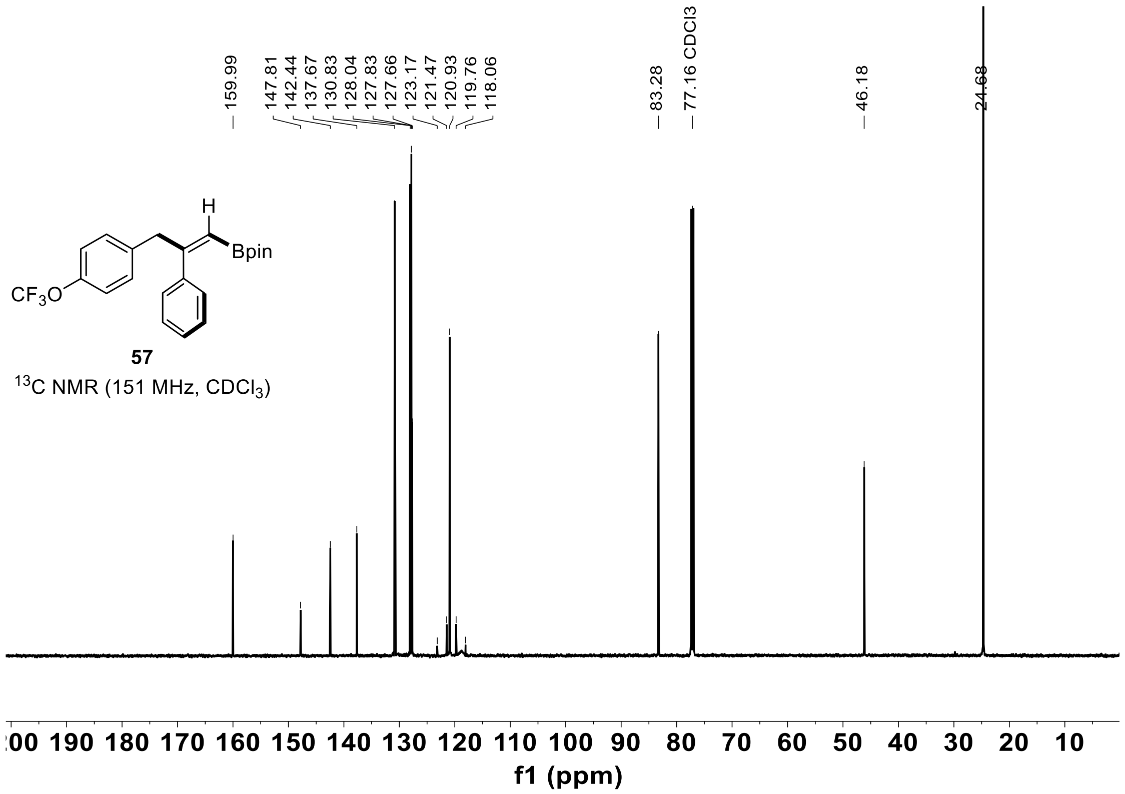

57

^{13}C NMR (151 MHz, $CDCl_3$)

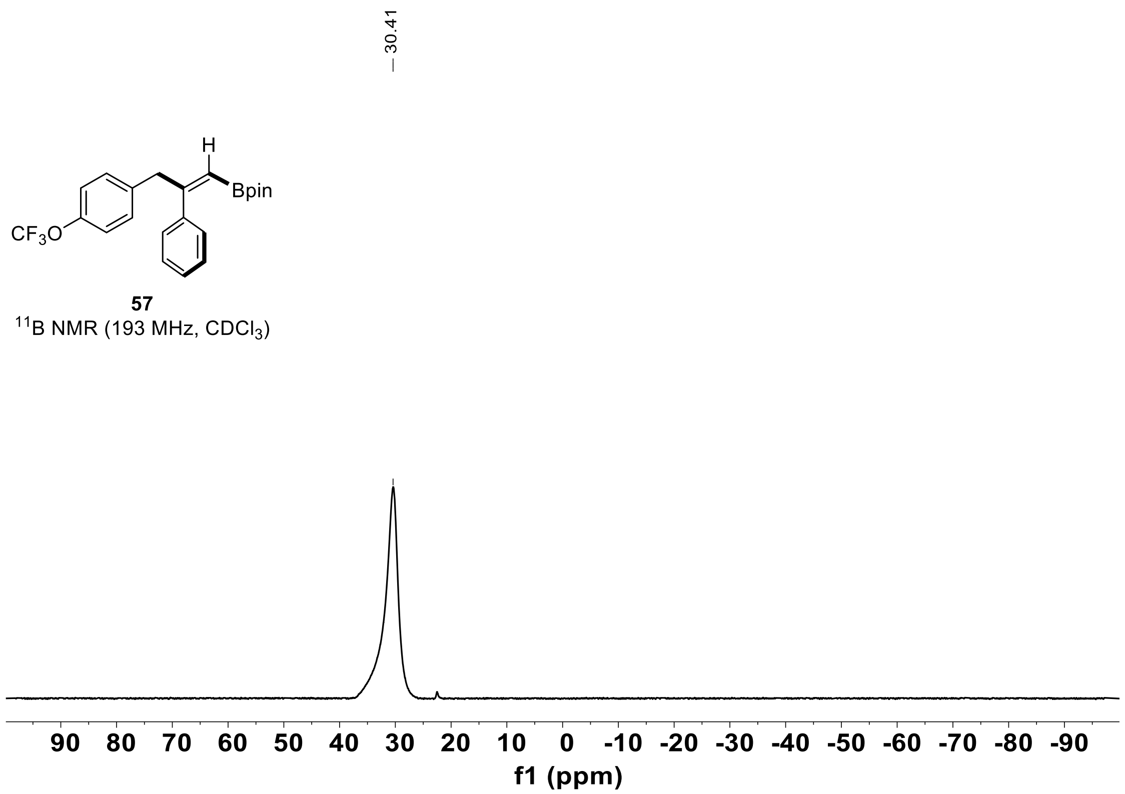
30.41
H
Bpin
CF_3O
57
^{11}B NMR (193 MHz, $CDCl_3$)
90 80 70 60 50 40 30 20 10 0 -10 -20 -30 -40 -50 -60 -70 -80 -90
f1 (ppm)

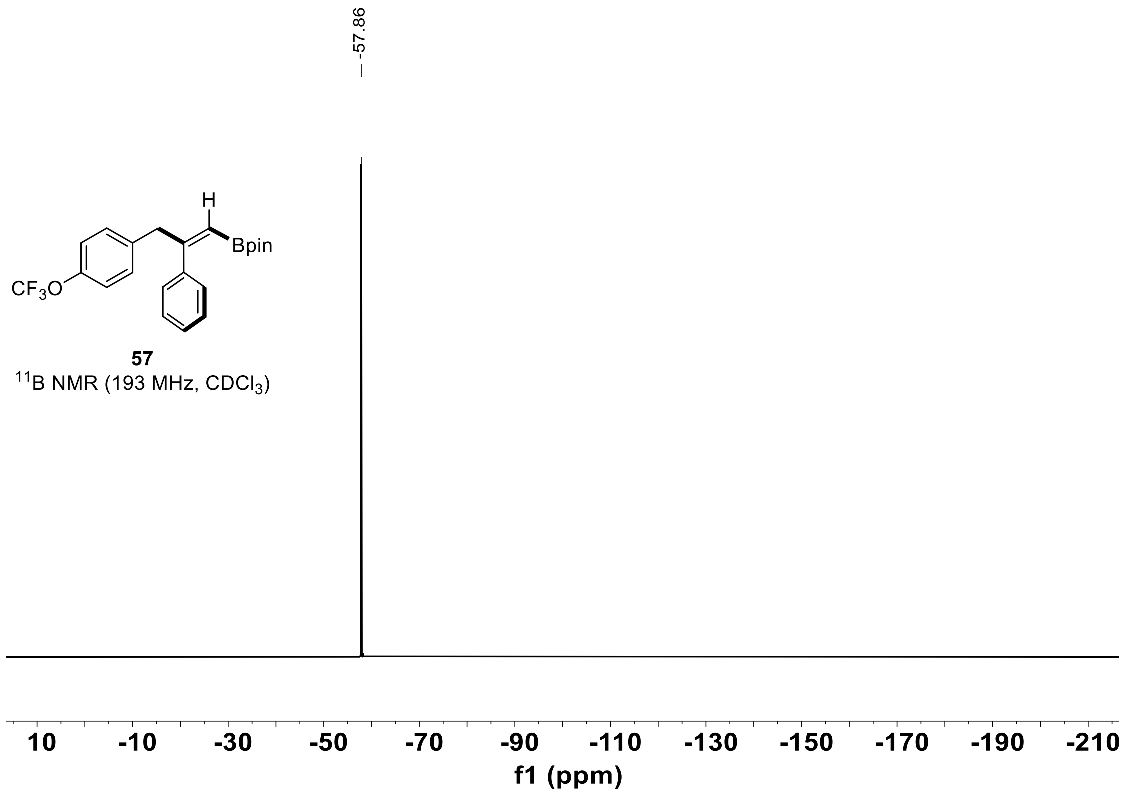
-57.86
H
Bpin
CF_3O
57
^{11}B NMR (193 MHz, $CDCl_3$)
10 -10 -30 -50 -70 -90 -110 -130 -150 -170 -190 -210
f1 (ppm)

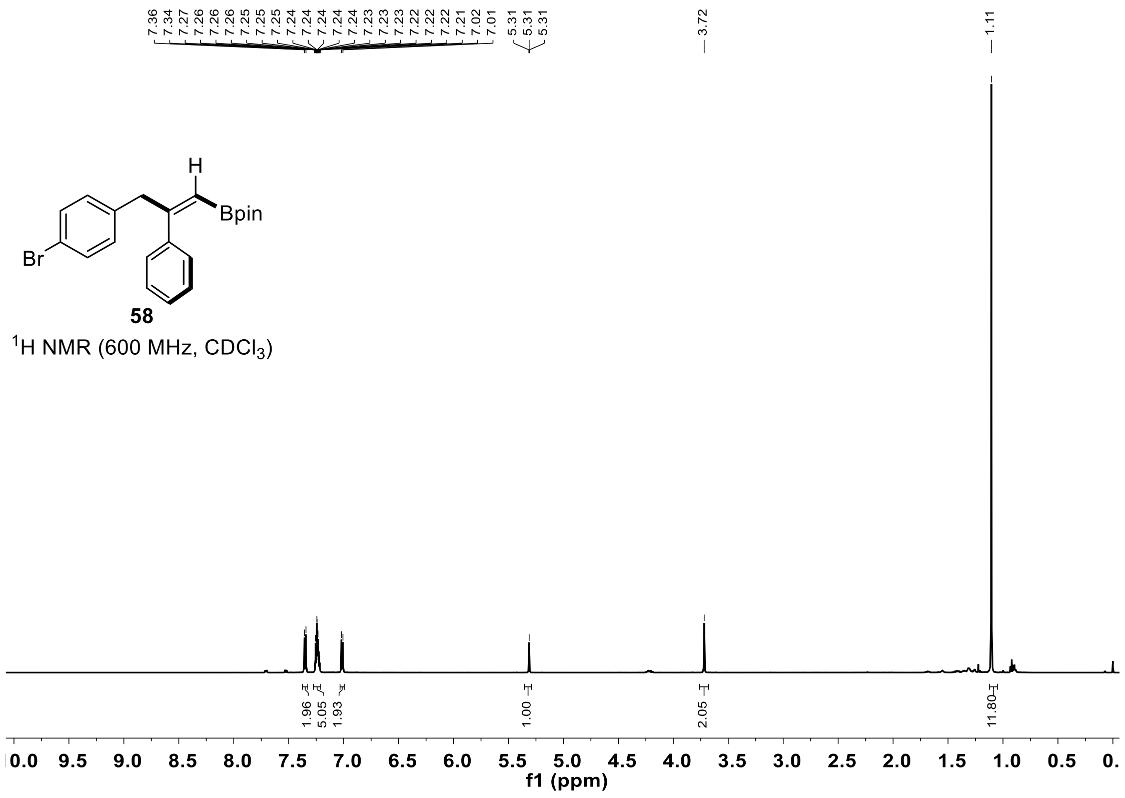
7.36 7.34 7.27 7.26 7.26 7.26 7.25 7.25 7.25 7.24 7.24 7.24 7.24 7.24 7.23 7.23 7.23 7.22 7.22 7.22 7.21 7.02 7.01
5.31 5.31 5.31
3.72
1.11
H
Bpin
Br
58
^{1}H NMR (600 MHz, $CDCl_3$)
1.96
5.05
1.93
1.00
2.05
11.80
10.0 9.5 9.0 8.5 8.0 7.5 7.0 6.5 6.0 5.5 5.0 4.5 4.0 3.5 3.0 2.5 2.0 1.5 1.0 0.5 0.
f1 (ppm)

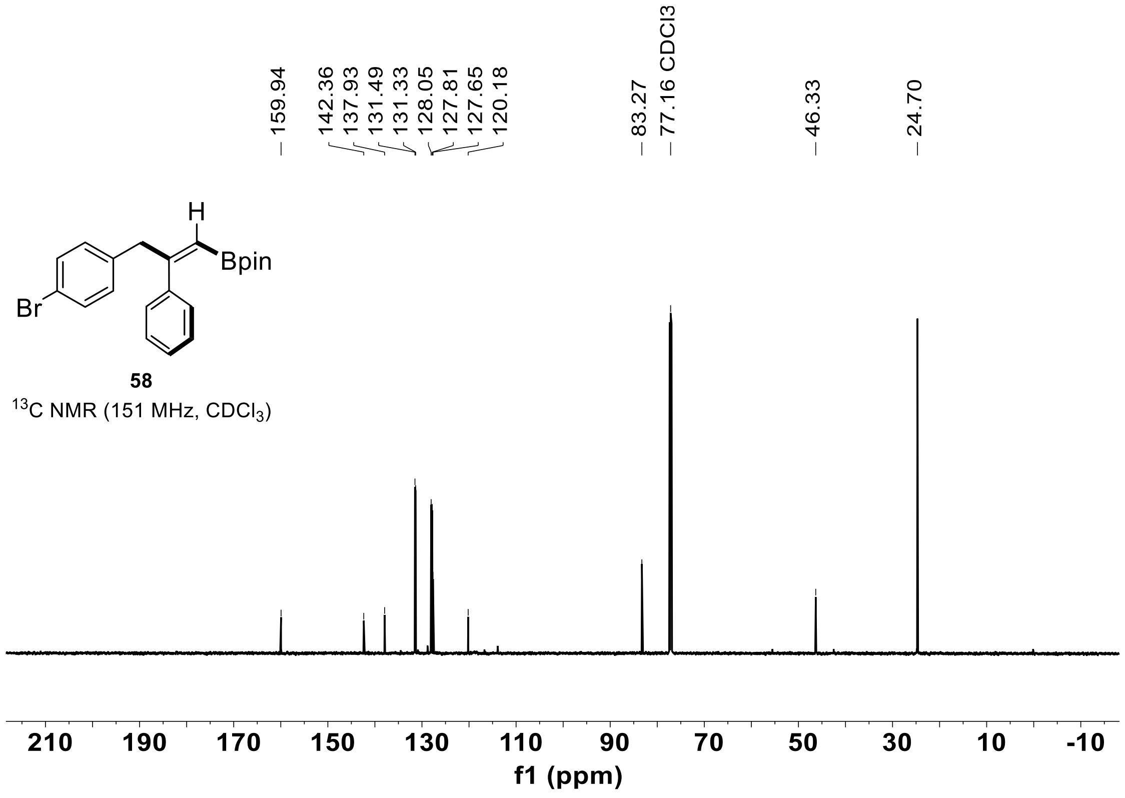
159.94
142.36
137.93
131.49
131.33
128.05
127.81
127.65
120.18
83.27
77.16 CDCl3
46.33
24.70
H
Bpin
Br
58
^{13}C NMR (151 MHz, $CDCl_3$)
210 190 170 150 130 110 90 70 50 30 10 -10
f1 (ppm)

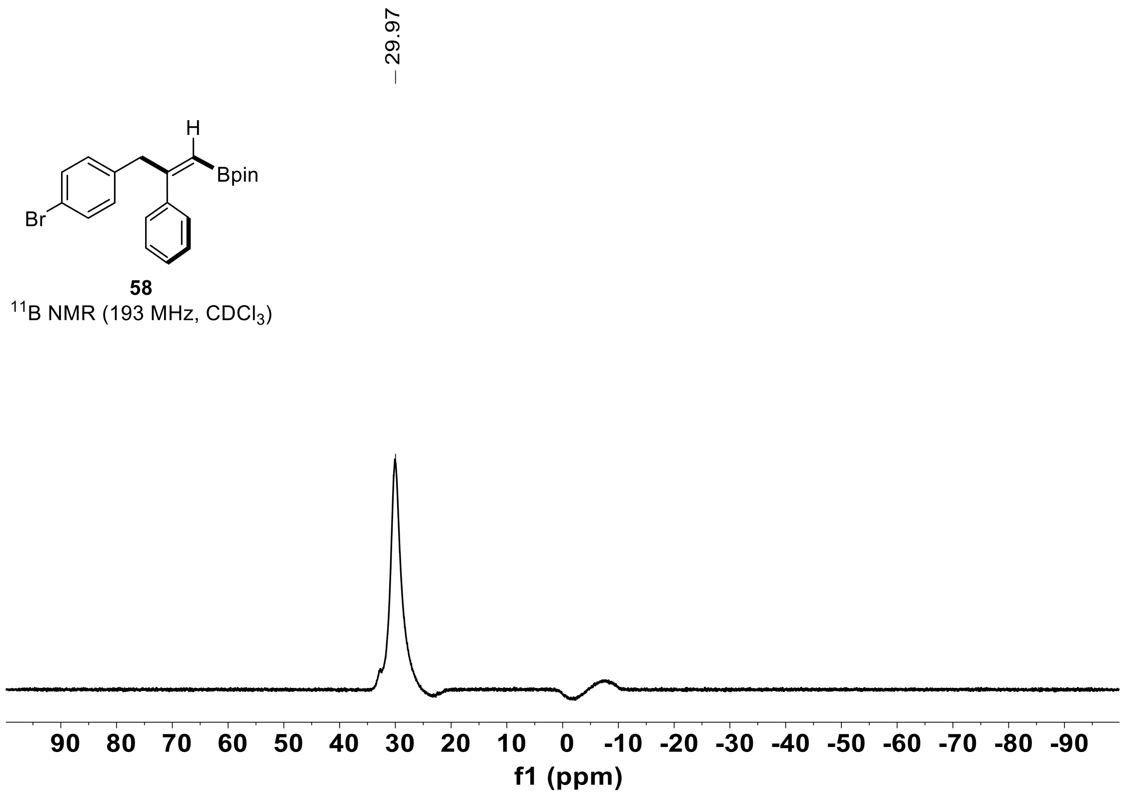
29.97
Bpin
Br
58
^{11}B NMR (193 MHz, $CDCl_3$)
90 80 70 60 50 40 30 20 10 0 -10 -20 -30 -40 -50 -60 -70 -80 -90
f1 (ppm)

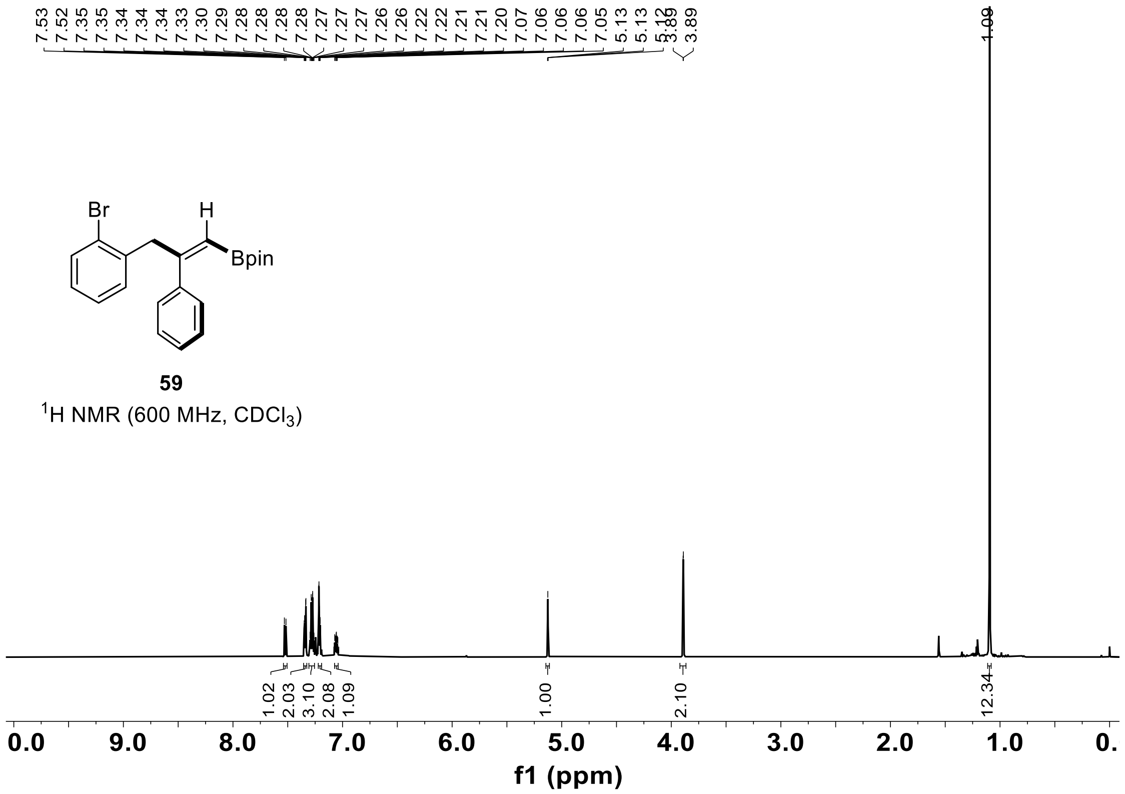
7.53 7.52 7.35 7.35 7.34 7.34 7.33 7.30 7.29 7.28 7.28 7.28 7.27 7.27 7.26 7.26 7.22 7.22 7.21 7.21 7.20 7.07 7.06 7.06 7.05 5.13 5.13 5.12 3.89 3.89
1.09
Br
Bpin
59
^{1}H NMR (600 MHz, $CDCl_3$)
1.02 2.03 3.10 2.08 1.09
1.00
2.10
12.34
0.0 9.0 8.0 7.0 6.0 5.0 4.0 3.0 2.0 1.0 0.
f1 (ppm)

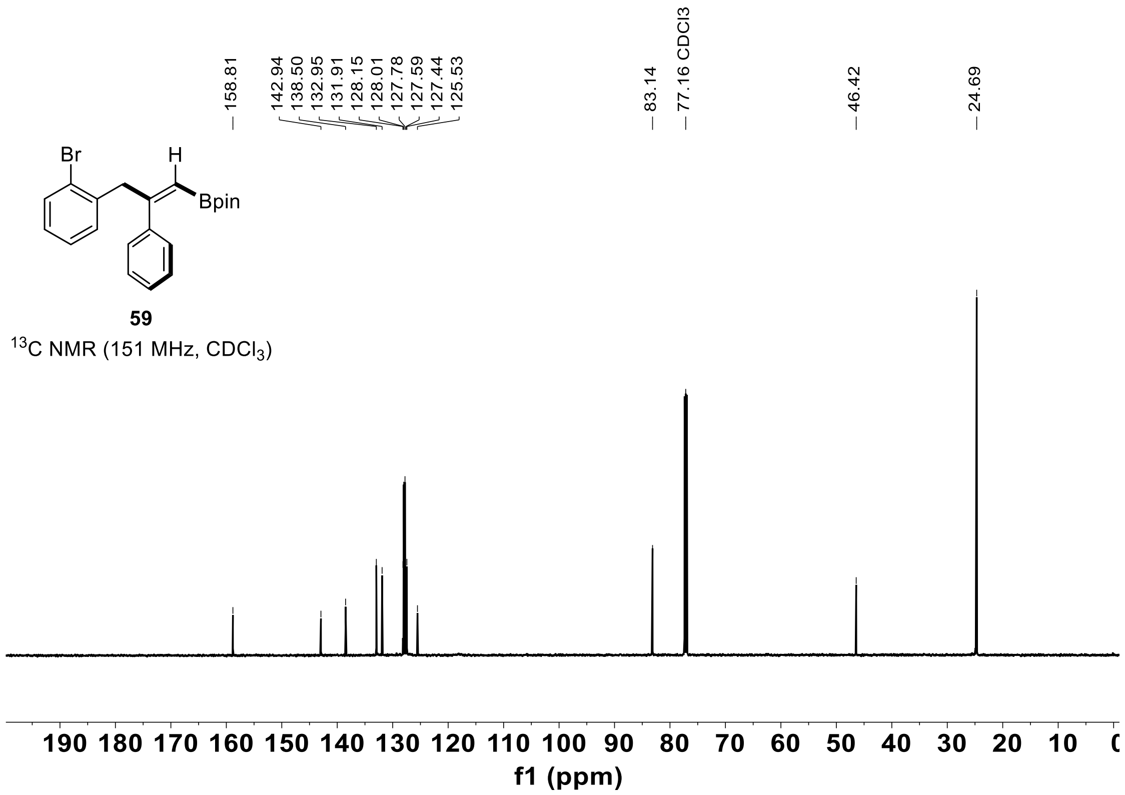

59

^{13}C NMR (151 MHz, $CDCl_3$)

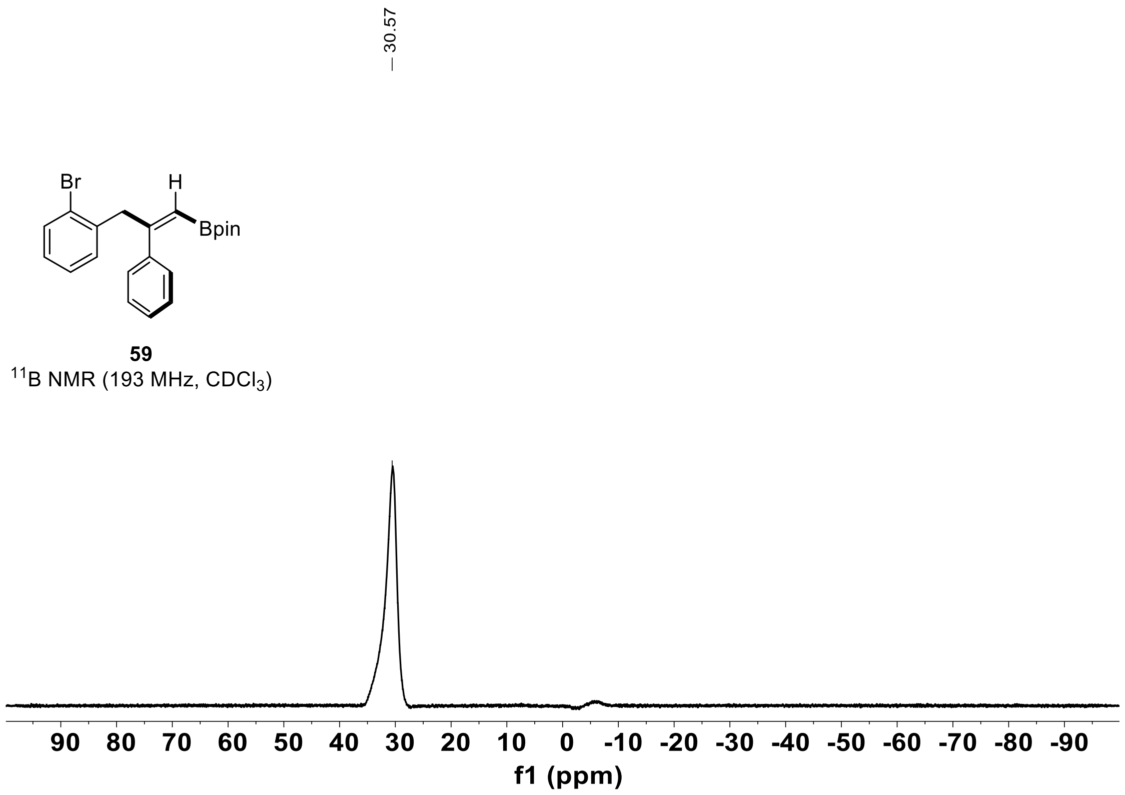

59

^{11}B NMR (193 MHz, $CDCl_3$)

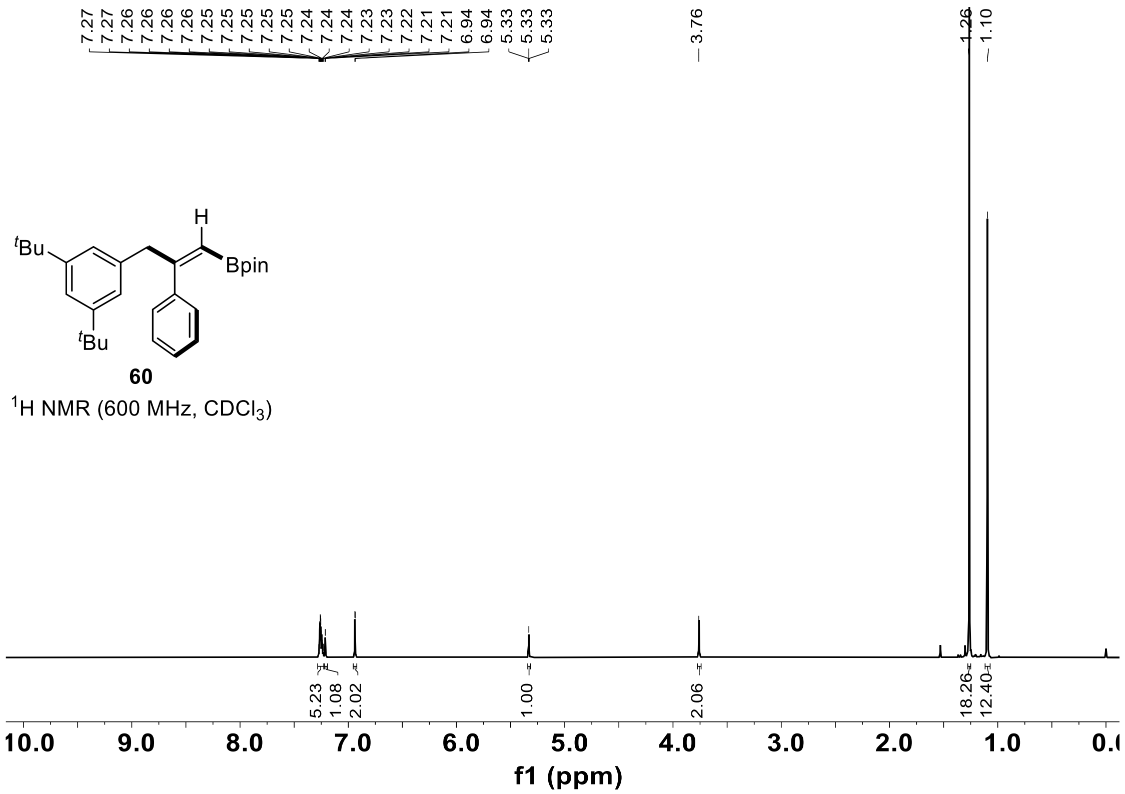

60

^{1}H NMR (600 MHz, $CDCl_3$)

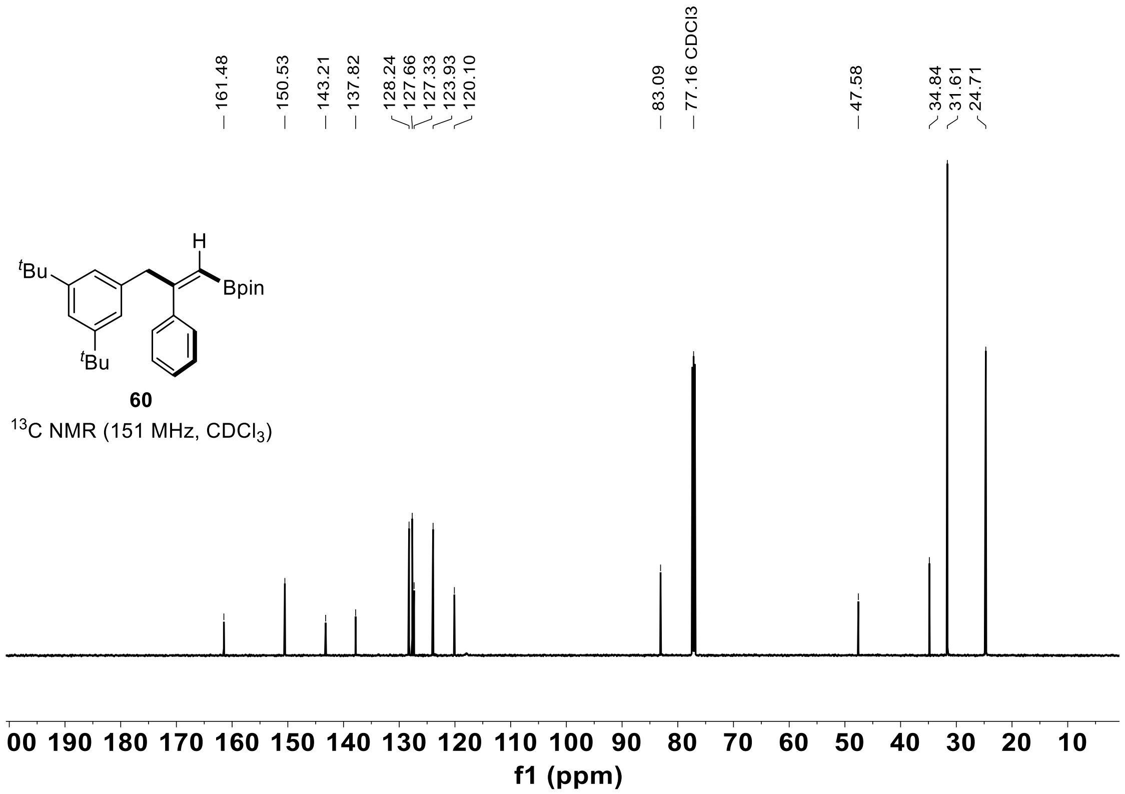

60

^{13}C NMR (151 MHz, $CDCl_3$)

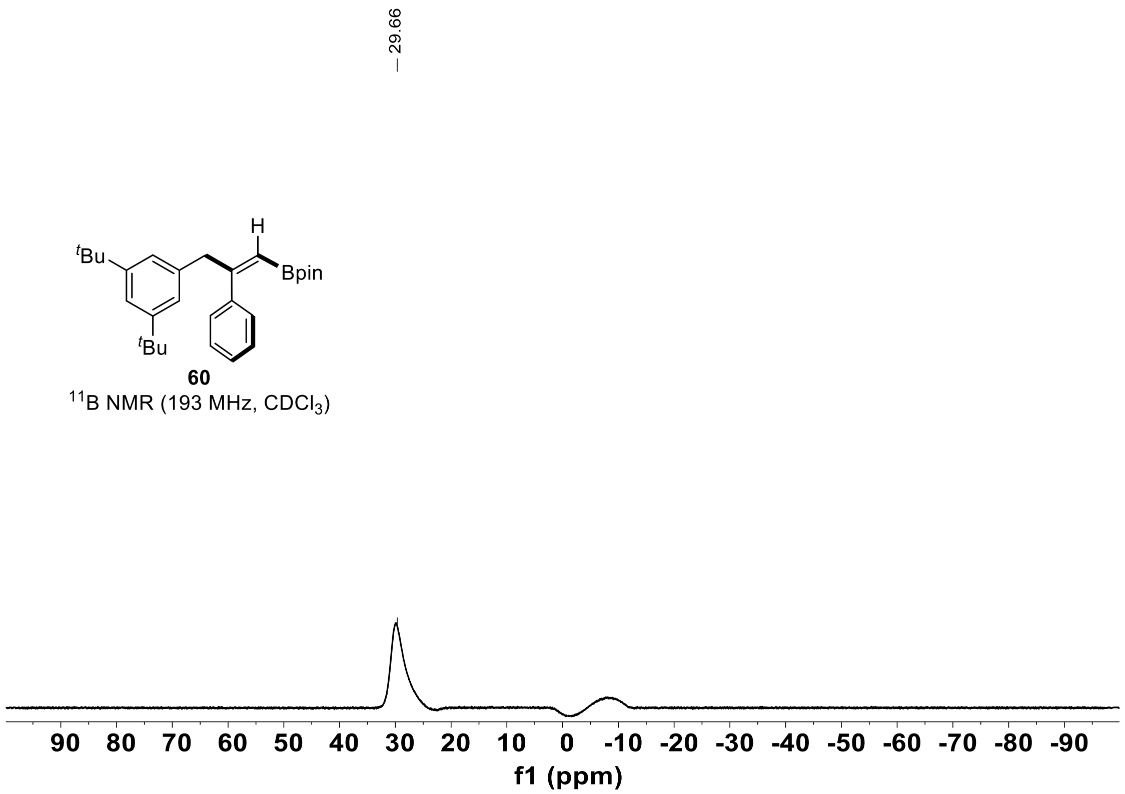

29.66
^tBu
^tBu
H
Bpin
60
^{11}B NMR (193 MHz, $CDCl_3$)
90 80 70 60 50 40 30 20 10 0 -10 -20 -30 -40 -50 -60 -70 -80 -90
f1 (ppm)

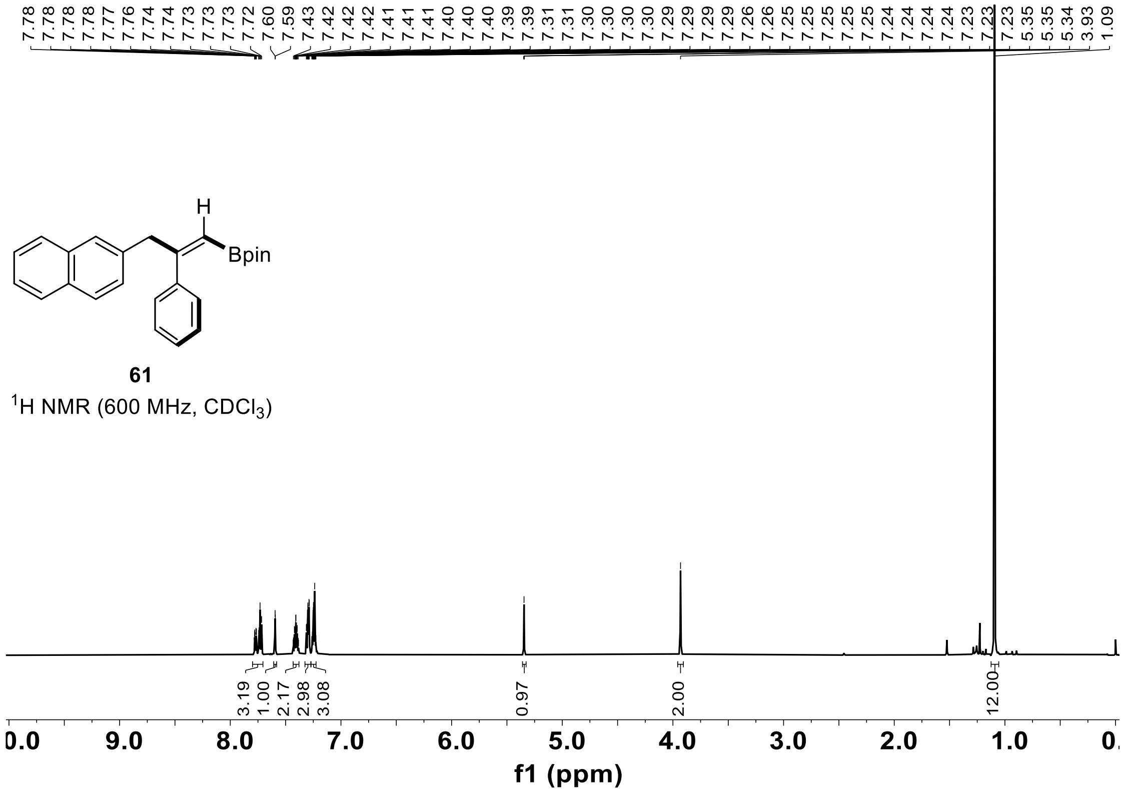

7.78 7.78 7.78 7.78 7.77 7.76 7.74 7.74 7.73 7.73 7.72 7.60 7.59 7.43 7.42 7.42 7.42 7.41 7.41 7.41 7.40 7.40 7.40 7.39 7.31 7.31 7.30 7.30 7.30 7.29 7.29 7.29 7.29 7.26 7.26 7.25 7.25 7.25 7.25 7.25 7.24 7.24 7.24 7.24 7.23 7.23 7.23 5.35 5.35 5.34 3.93 1.09
H
Bpin
61
^1H NMR (600 MHz, $CDCl_3$)
3.19 1.00 2.17 2.98 3.08 0.97 2.00 12.00
0.0 9.0 8.0 7.0 6.0 5.0 4.0 3.0 2.0 1.0 0.
f1 (ppm)

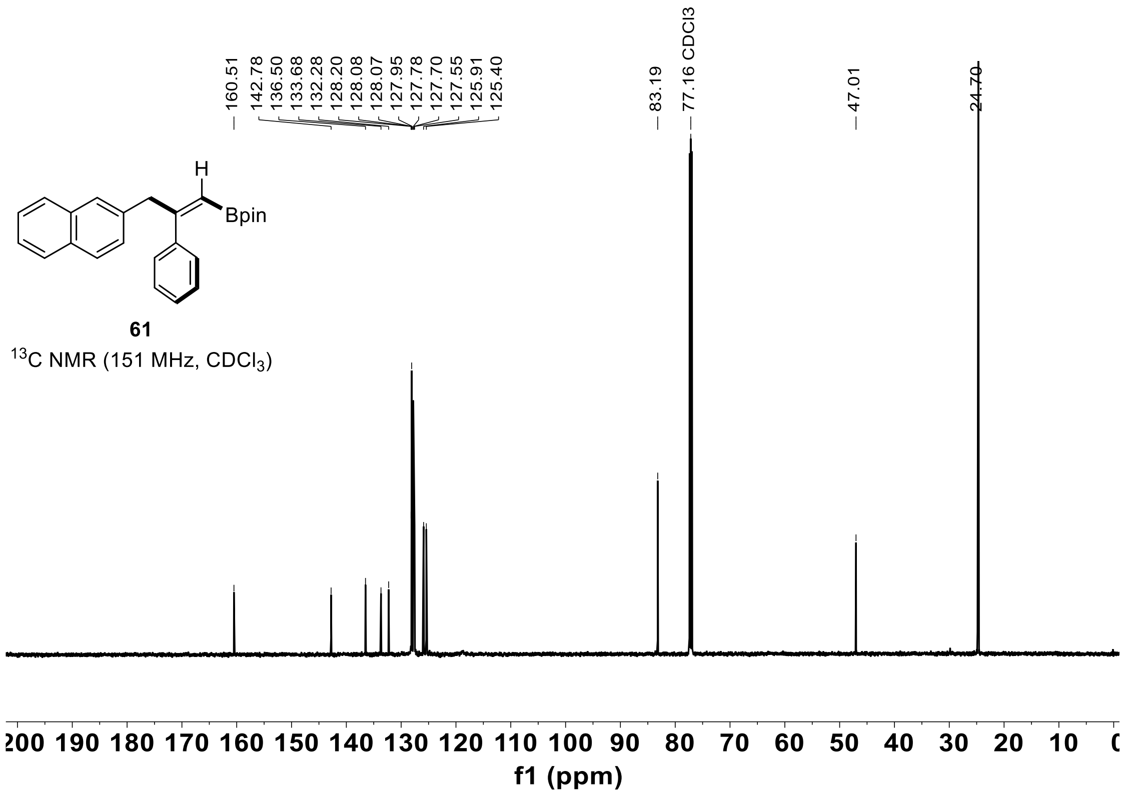

61

^{13}C NMR (151 MHz, $CDCl_3$)

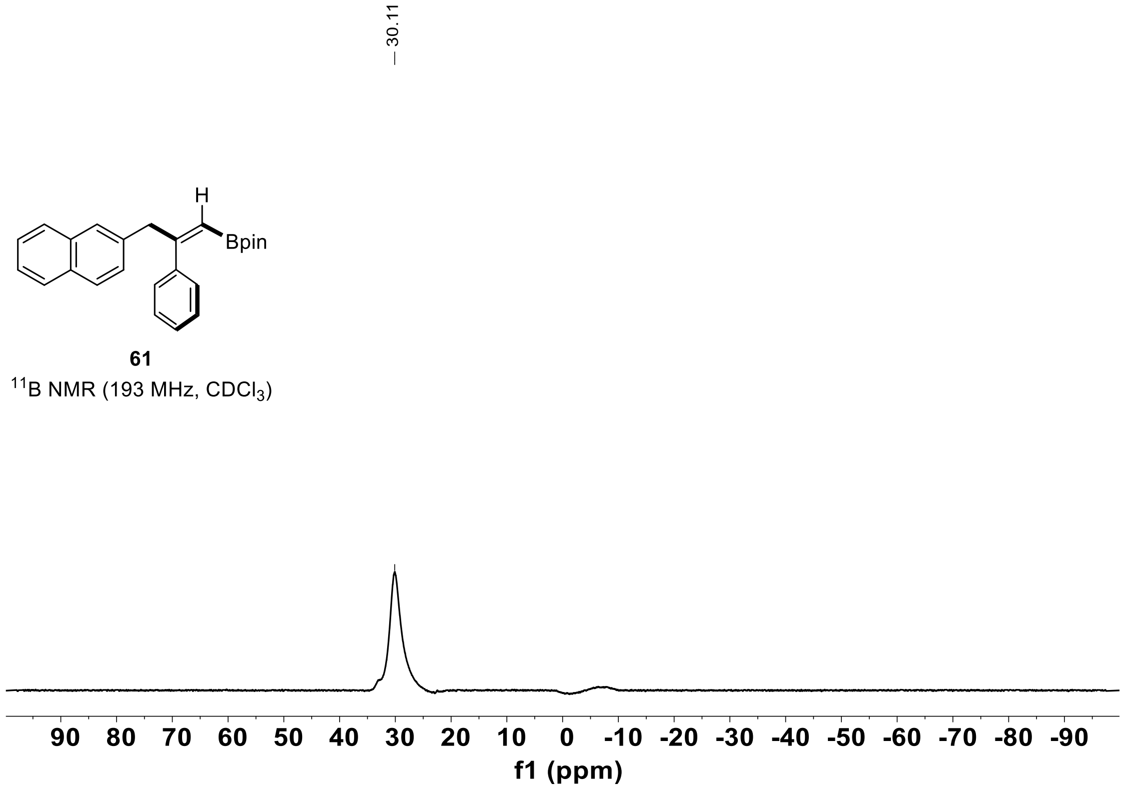

61

^{11}B NMR (193 MHz, $CDCl_3$)

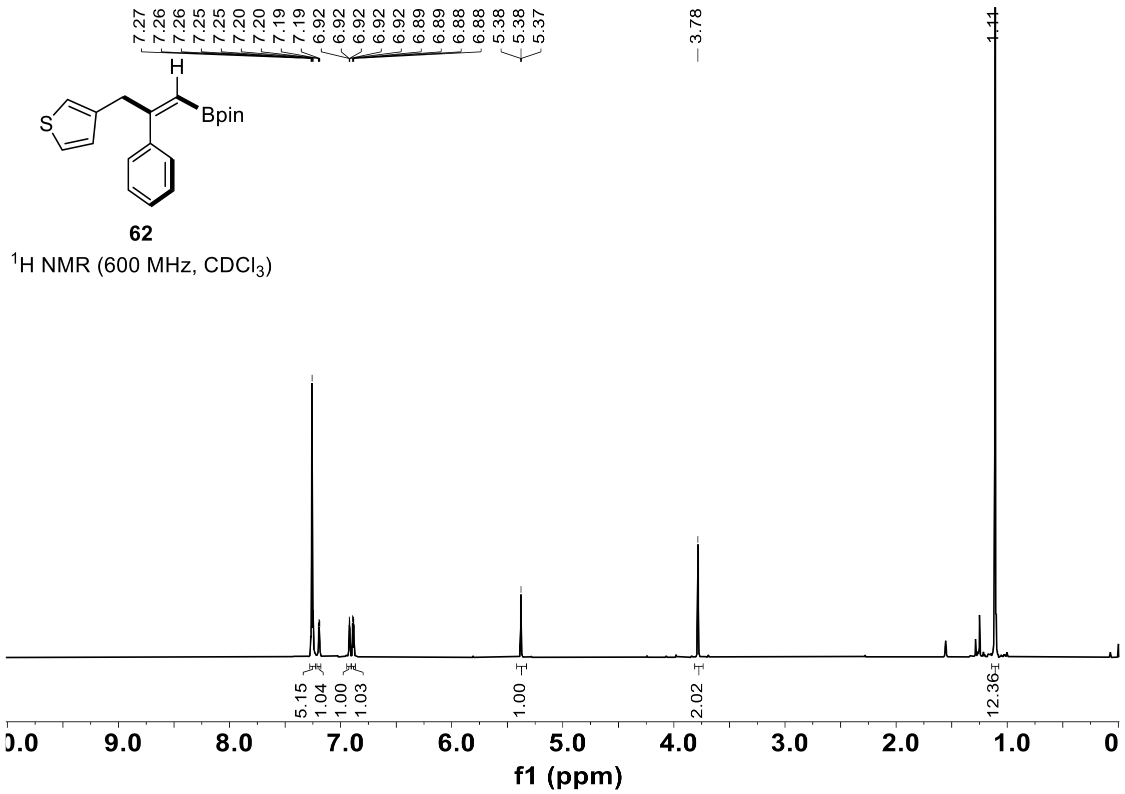

62

^{1}H NMR (600 MHz, $CDCl_3$)

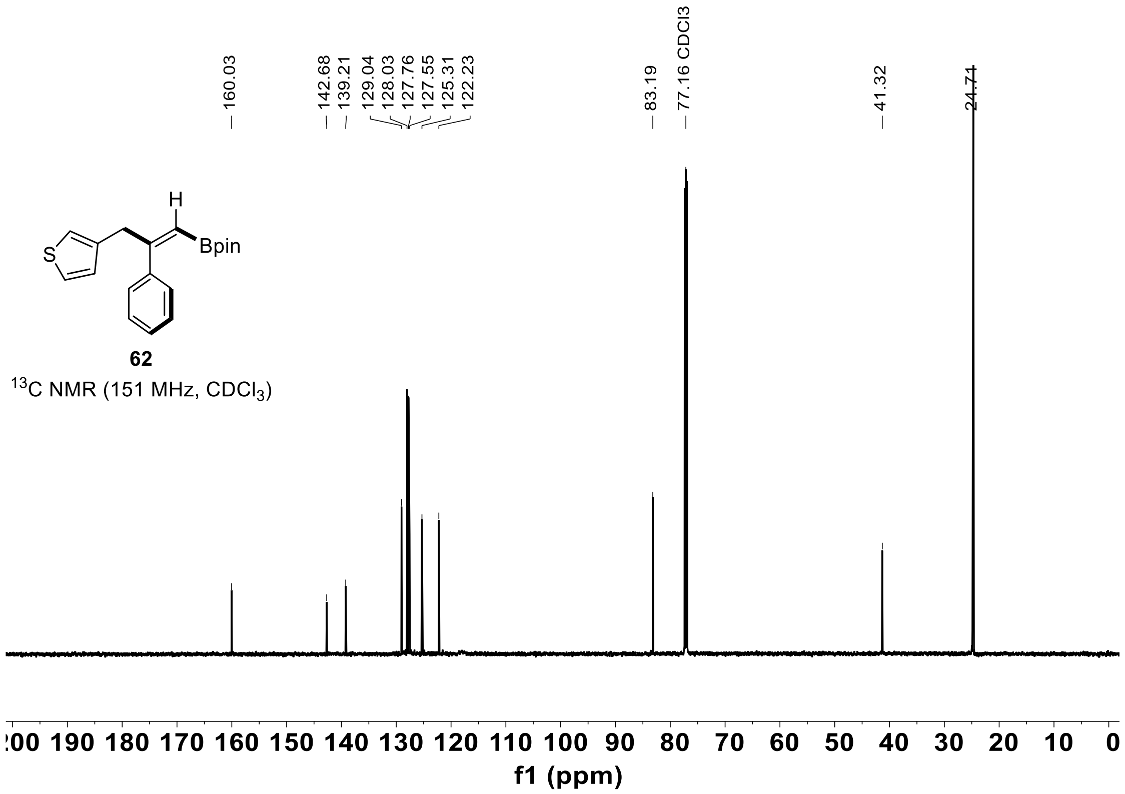

62

^{13}C NMR (151 MHz, $CDCl_3$)

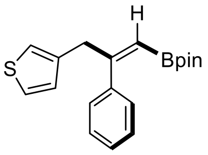

62

^{11}B NMR (193 MHz, $CDCl_3$)

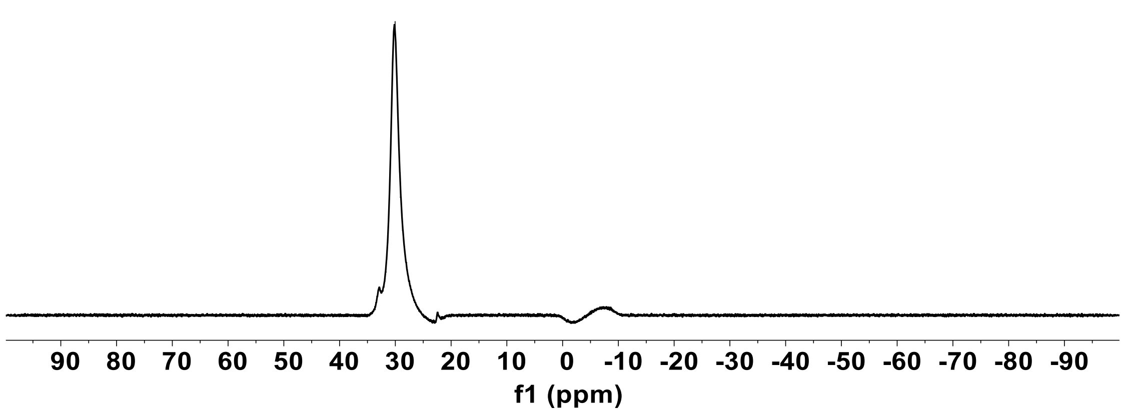

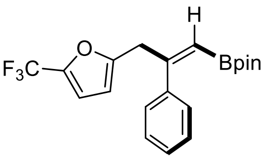

63

^{1}H NMR (600 MHz, $CDCl_3$)

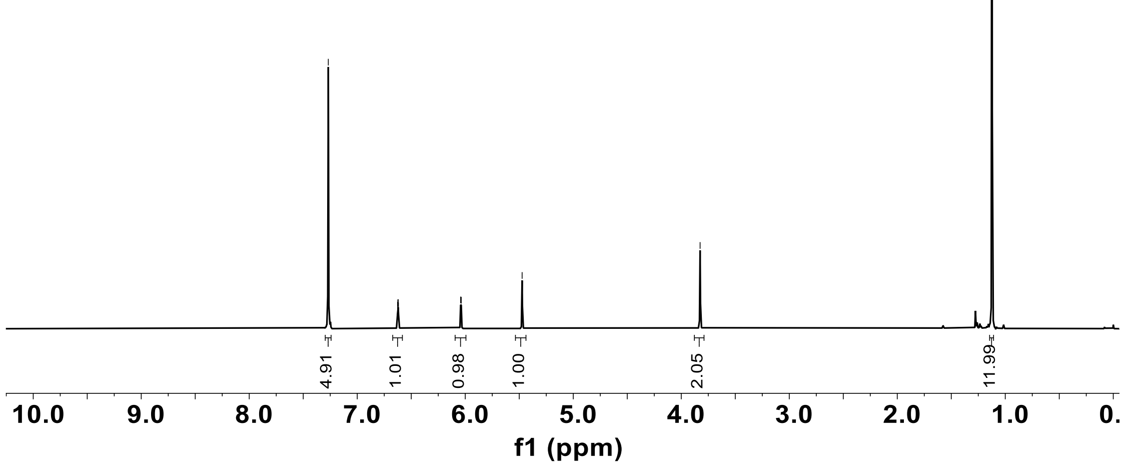

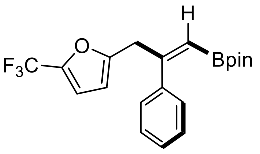

63
^{13}C NMR (151 MHz, $CDCl_3$)

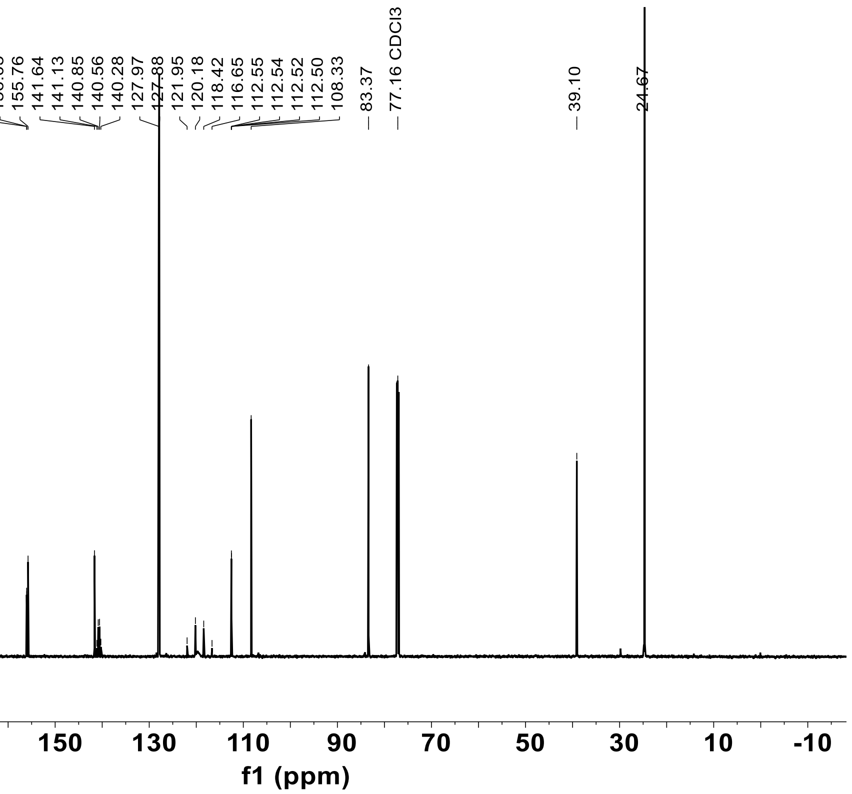

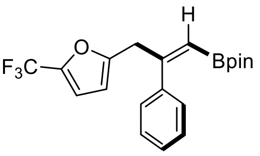

63
^{11}B NMR (193 MHz, $CDCl_3$)

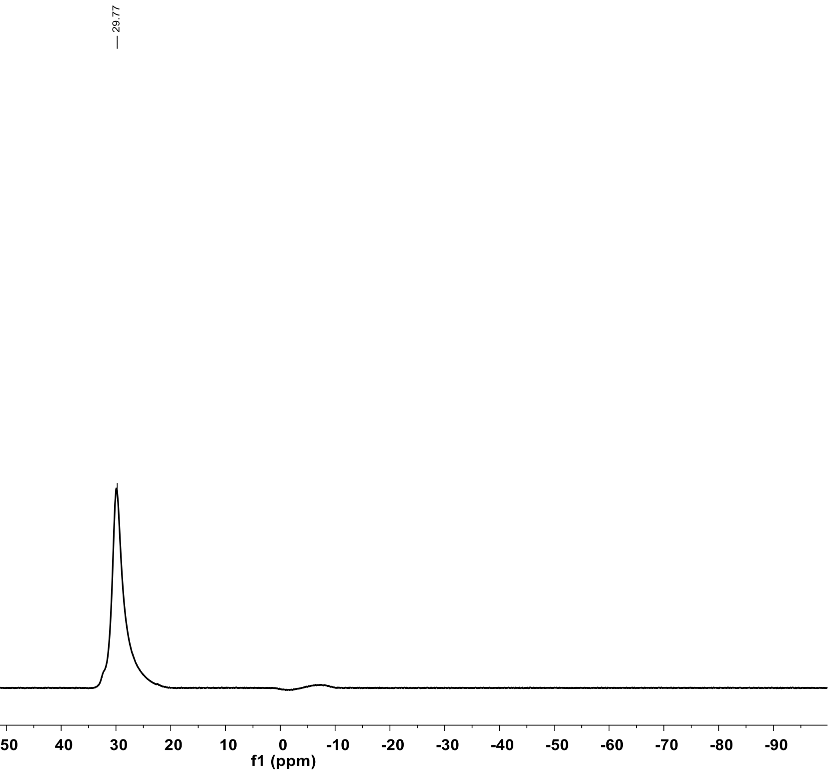

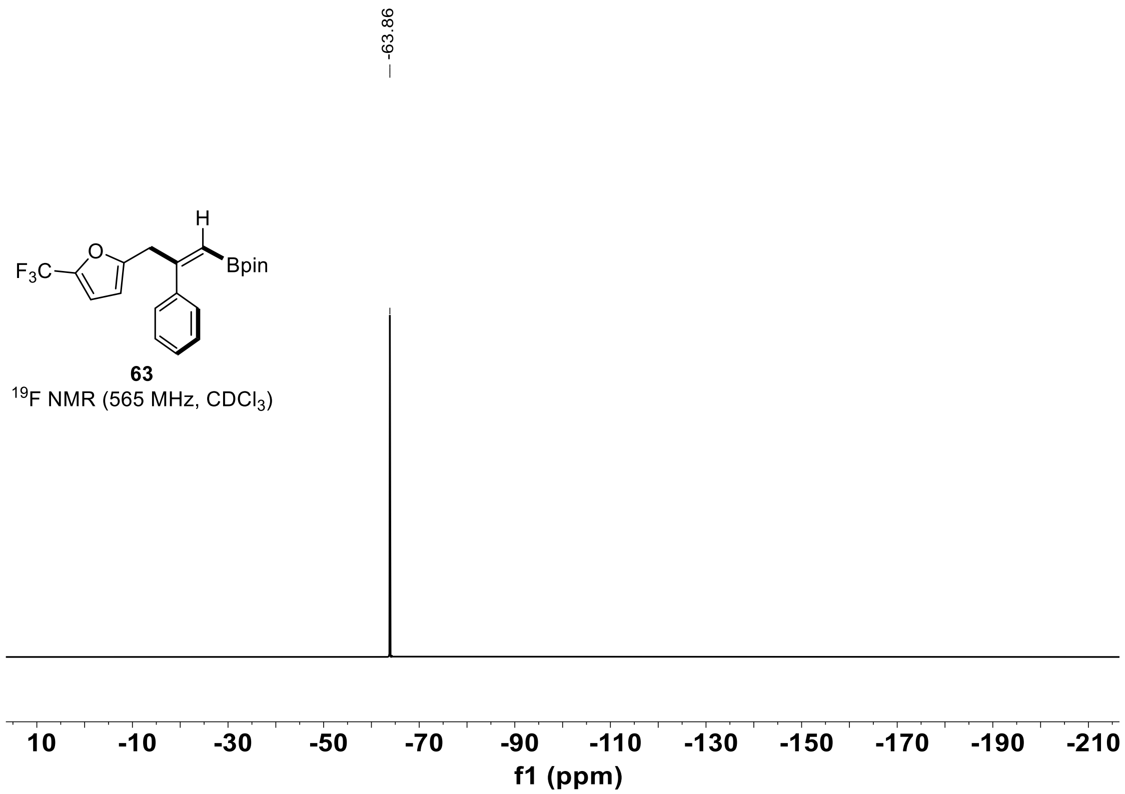

63

^{19}F NMR (565 MHz, $CDCl_3$)

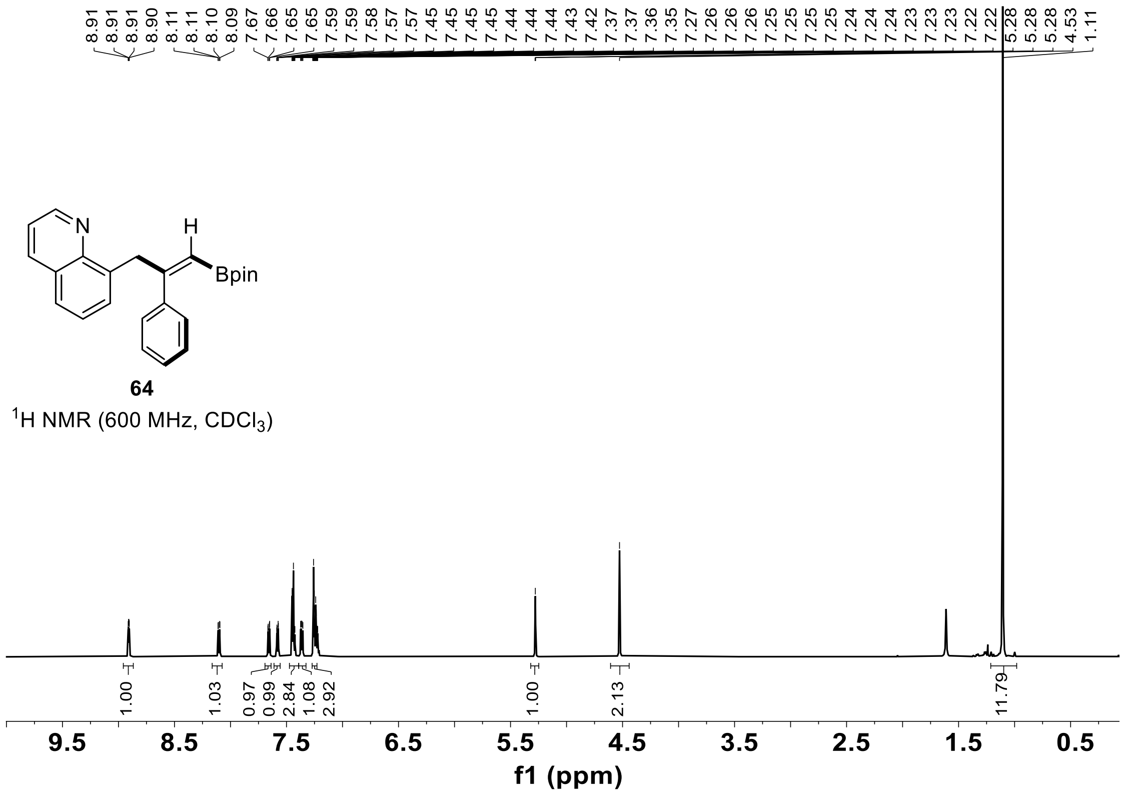

64

^{1}H NMR (600 MHz, $CDCl_3$)

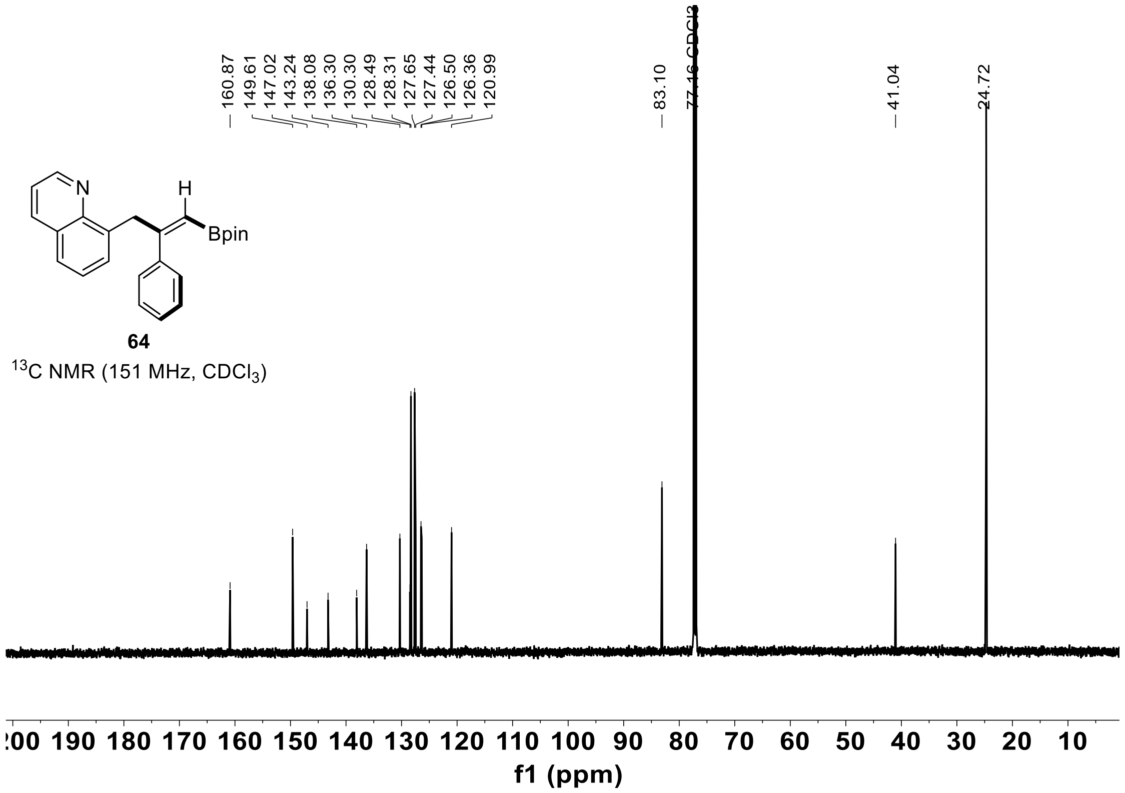

64

^{13}C NMR (151 MHz, $CDCl_3$)

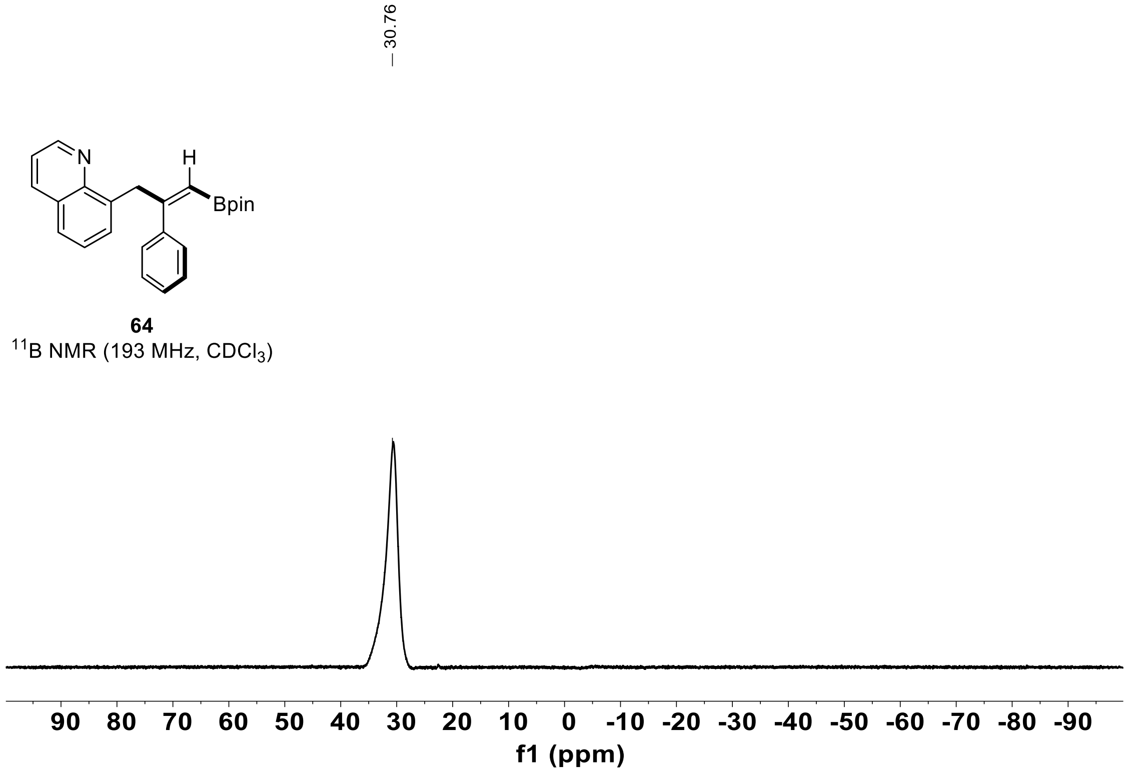

64

^{11}B NMR (193 MHz, $CDCl_3$)

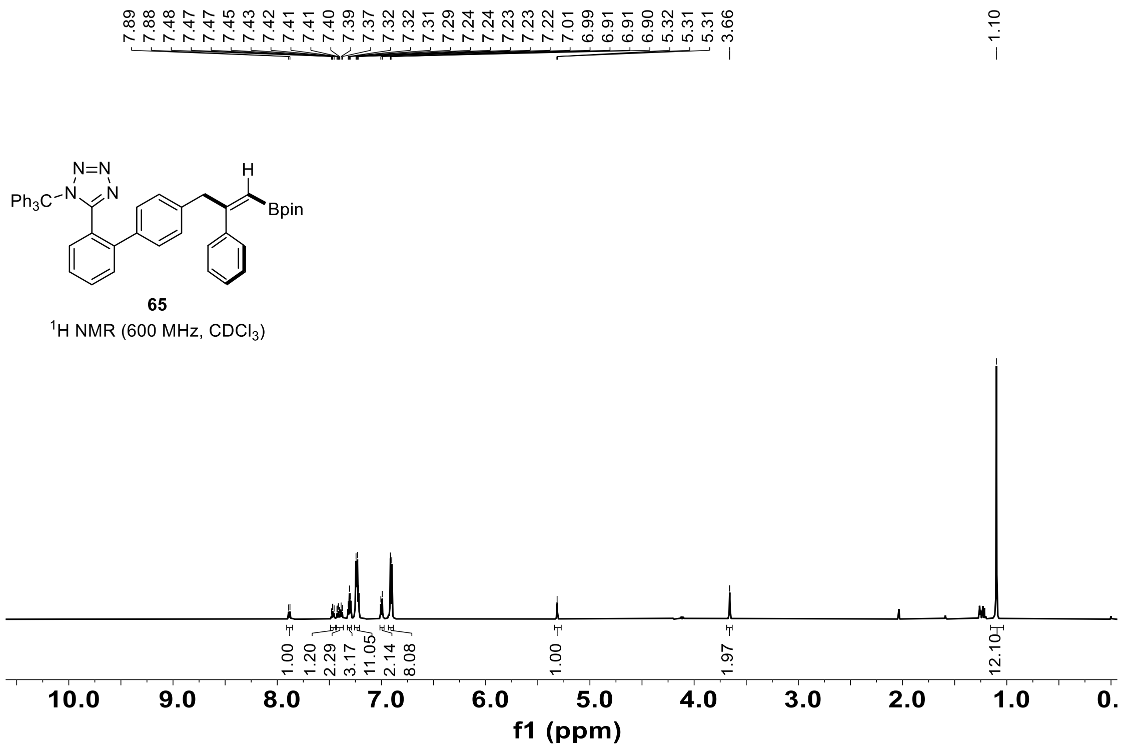

65

^{1}H NMR (600 MHz, $CDCl_3$)

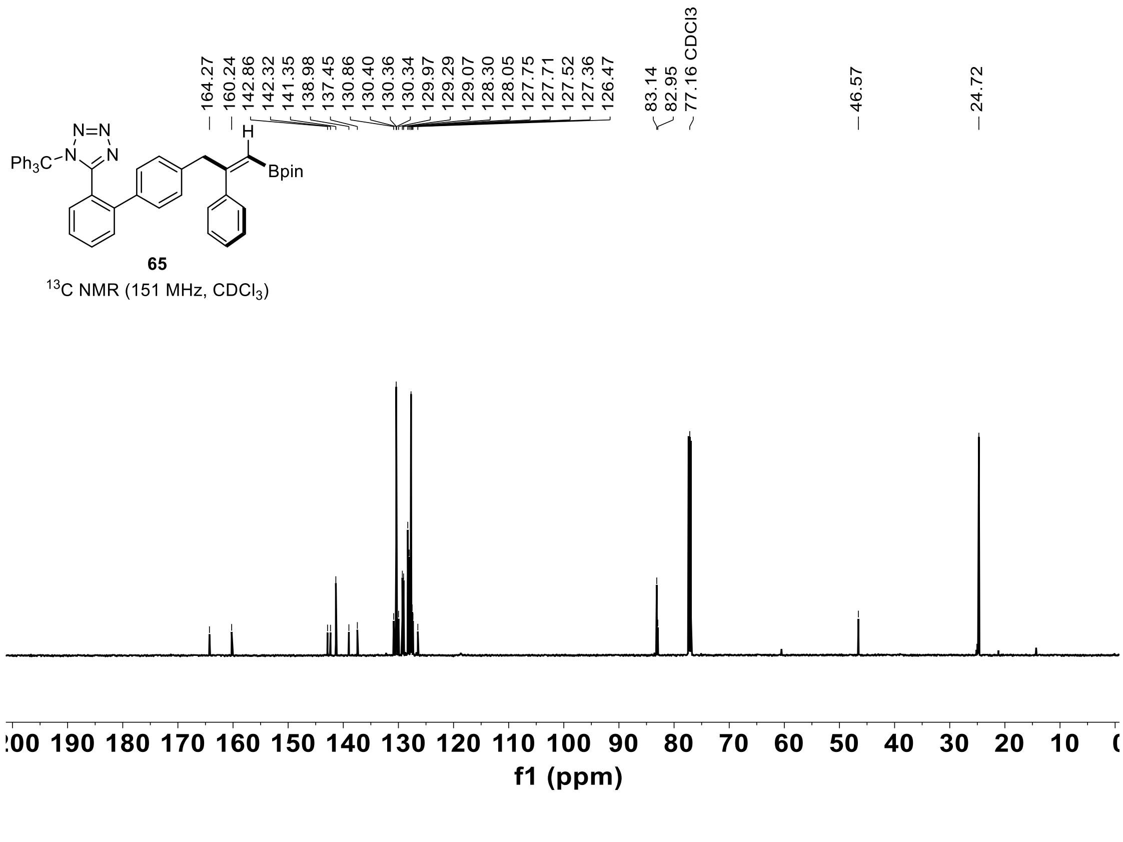

65

^{13}C NMR (151 MHz, $CDCl_3$)

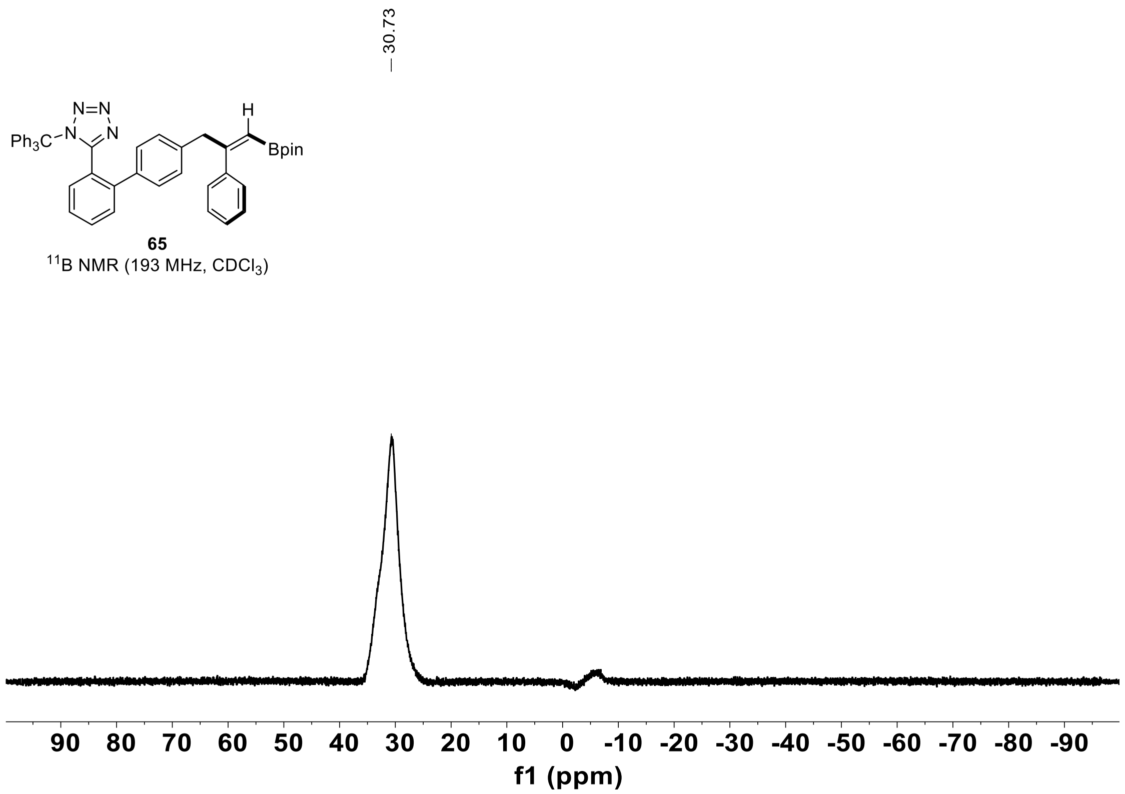

65
11B NMR (193 MHz, CDCl3)
f1 (ppm)

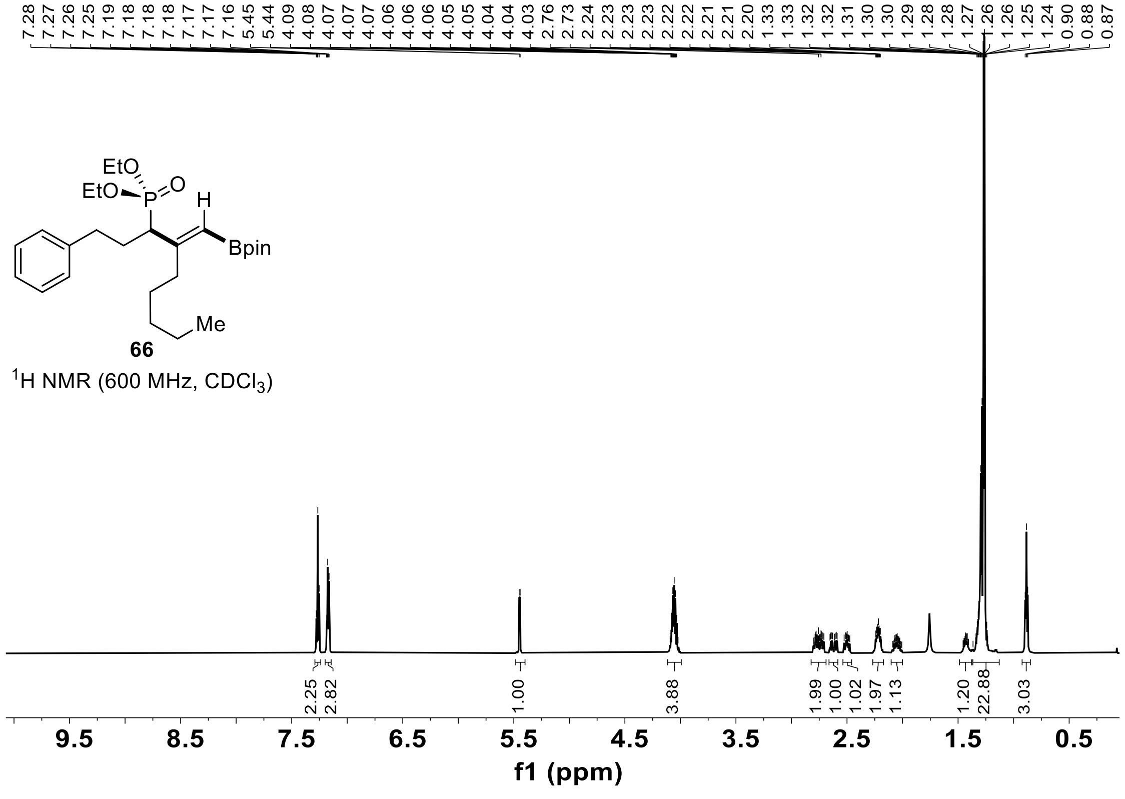

66
1H NMR (600 MHz, CDCl3)
f1 (ppm)

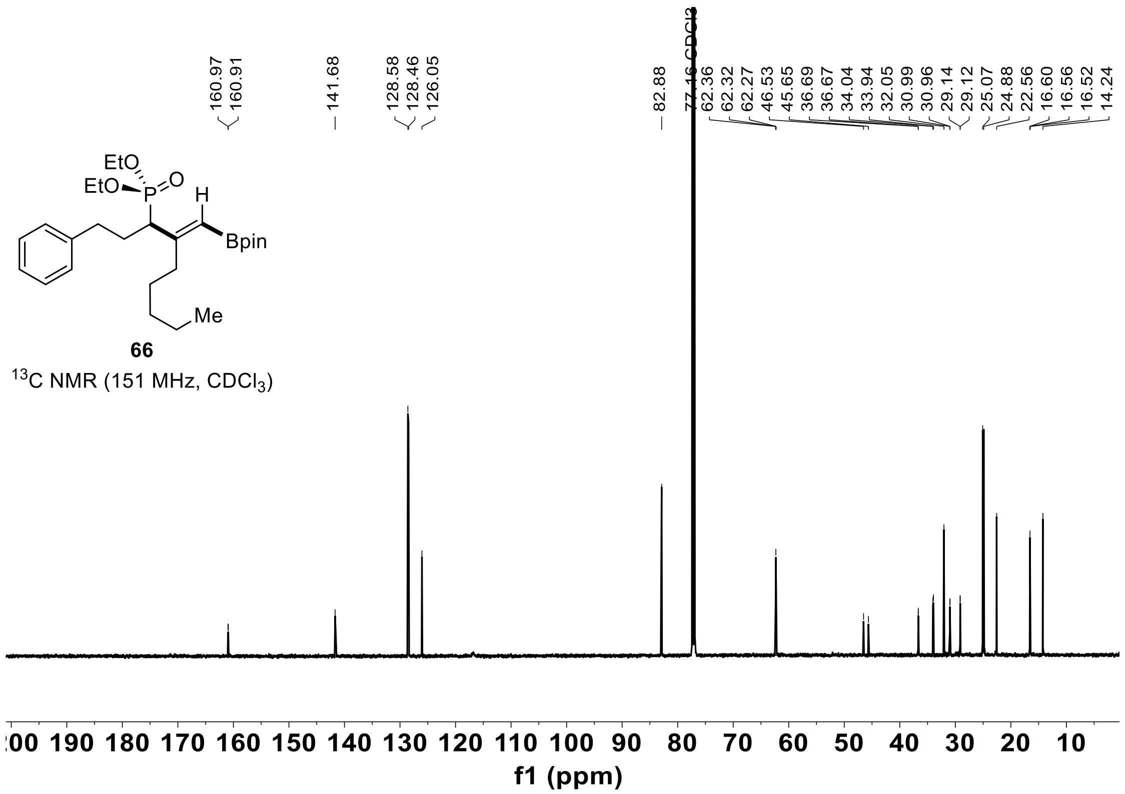
160.97
160.91
141.68
128.58
128.46
126.05
82.88
77.16 CDCl3
62.36
62.32
62.27
46.53
45.65
36.69
36.67
34.04
33.94
32.05
30.99
30.96
29.14
29.12
25.07
24.88
22.56
16.60
16.56
16.52
14.24
EtO
EtO
P
O
H
Bpin
Me
66
^{13}C NMR (151 MHz, CDCl$_3$)
f1 (ppm)

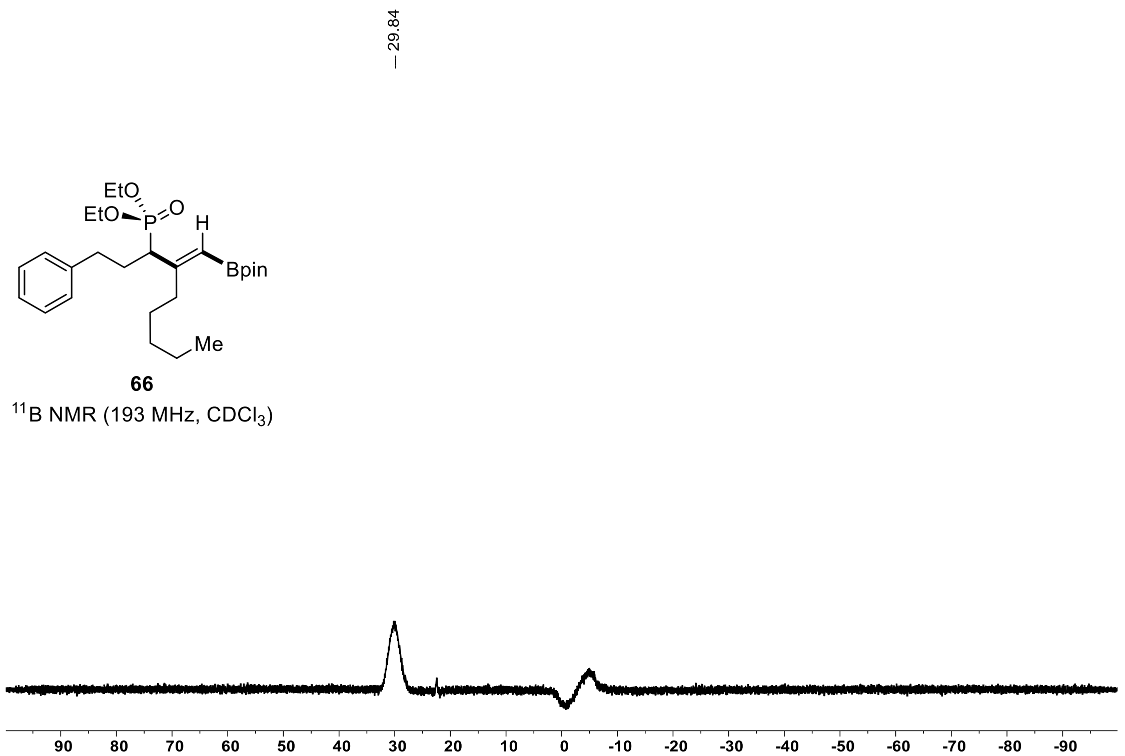
29.84
EtO
EtO
P
O
H
Bpin
Me
66
^{11}B NMR (193 MHz, CDCl$_3$)
f1 (ppm)

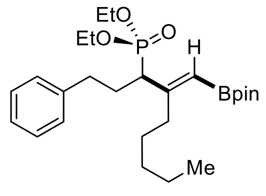

66

^{31}P NMR (243 MHz, $CDCl_3$)

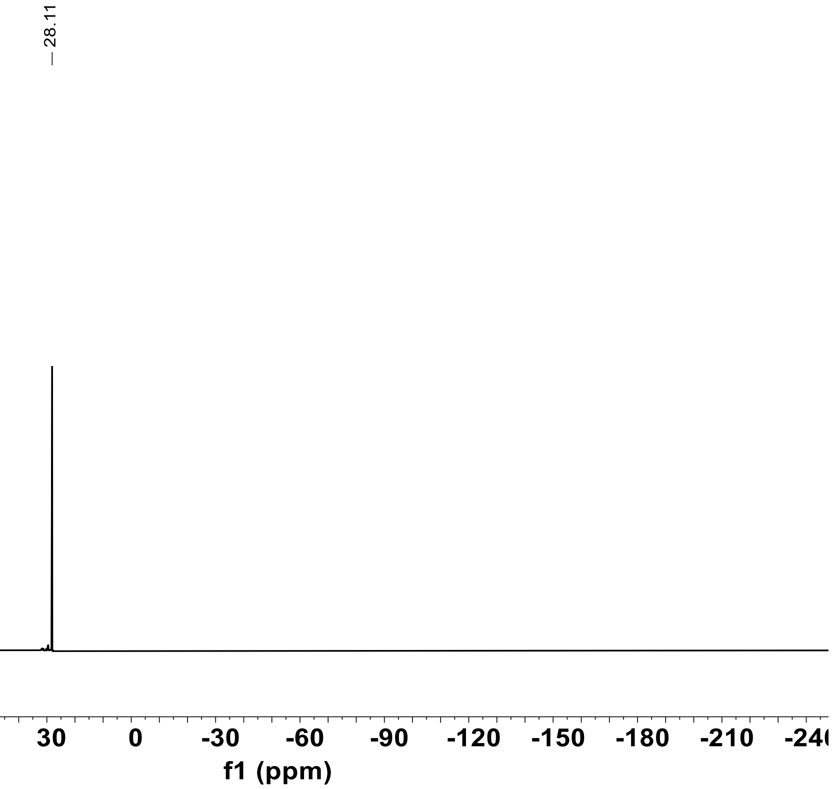

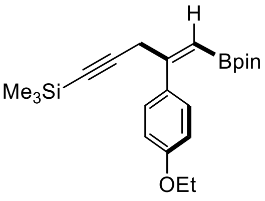

67

^{1}H NMR (600 MHz, $CDCl_3$)

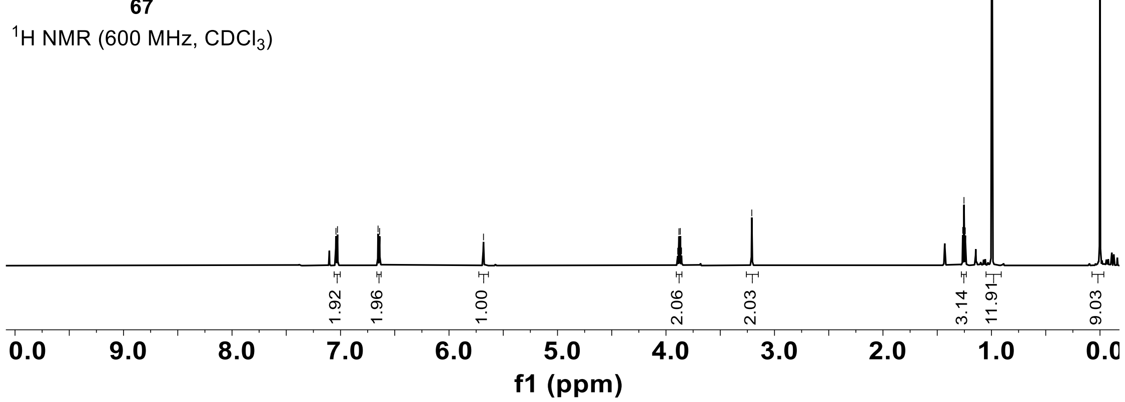

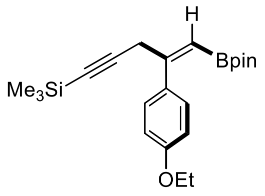

67

^{13}C NMR (151 MHz, $CDCl_3$)

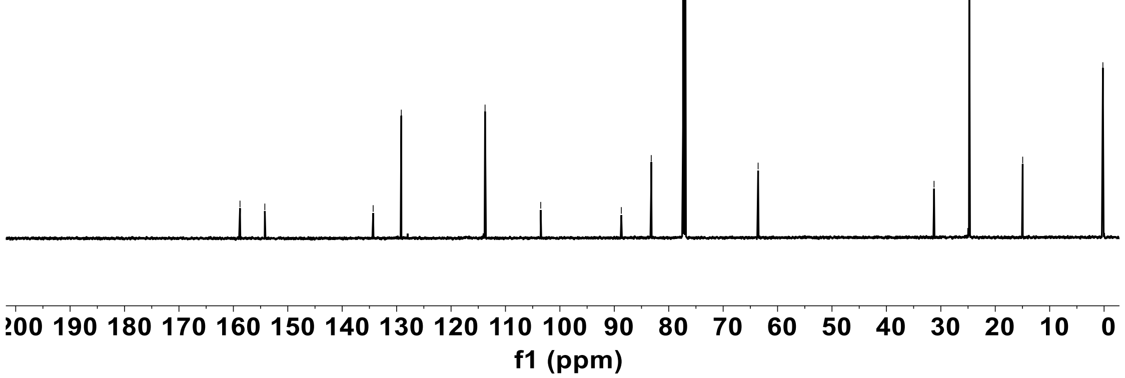

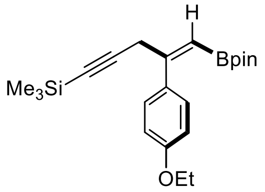

67

^{11}B NMR (193 MHz, $CDCl_3$)

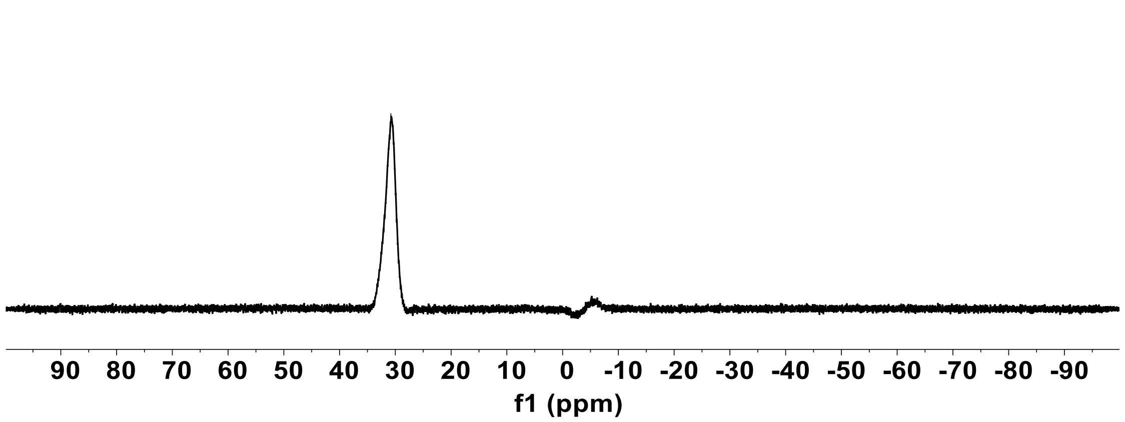

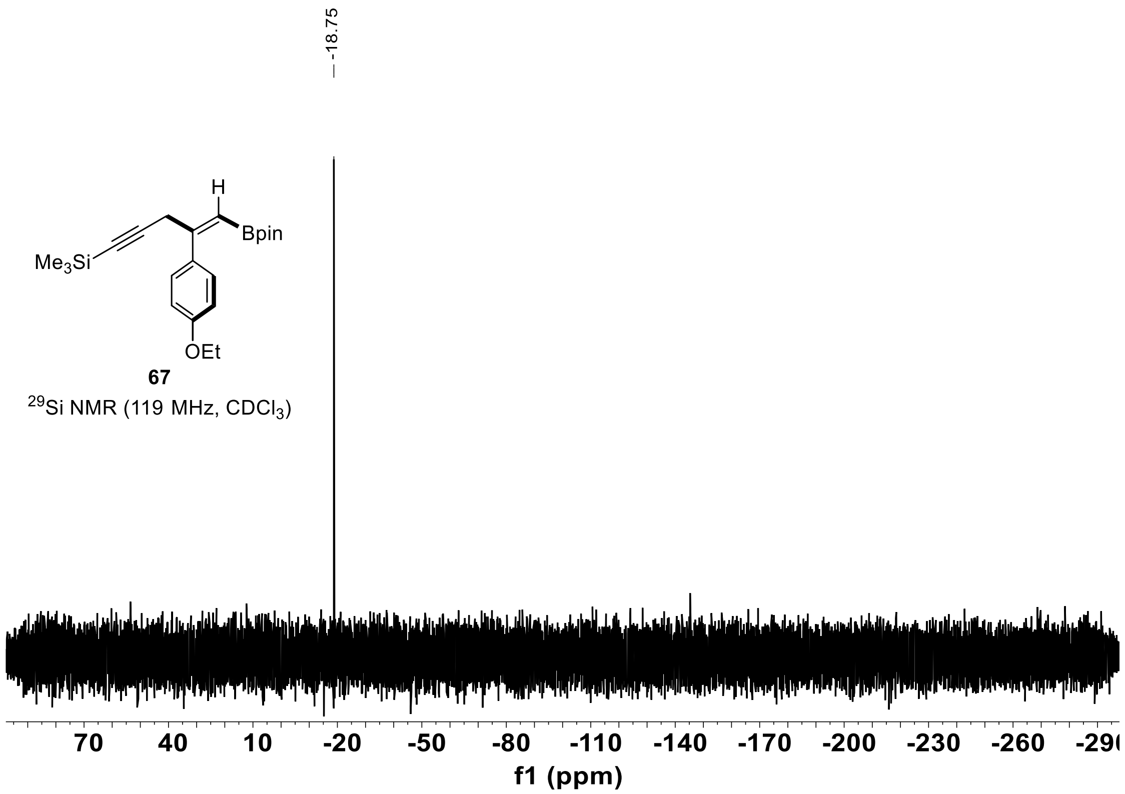

-18.75
H
Bpin
Me3Si
OEt
67
29Si NMR (119 MHz, CDCl3)
70 40 10 -20 -50 -80 -110 -140 -170 -200 -230 -260 -29(
f1 (ppm)

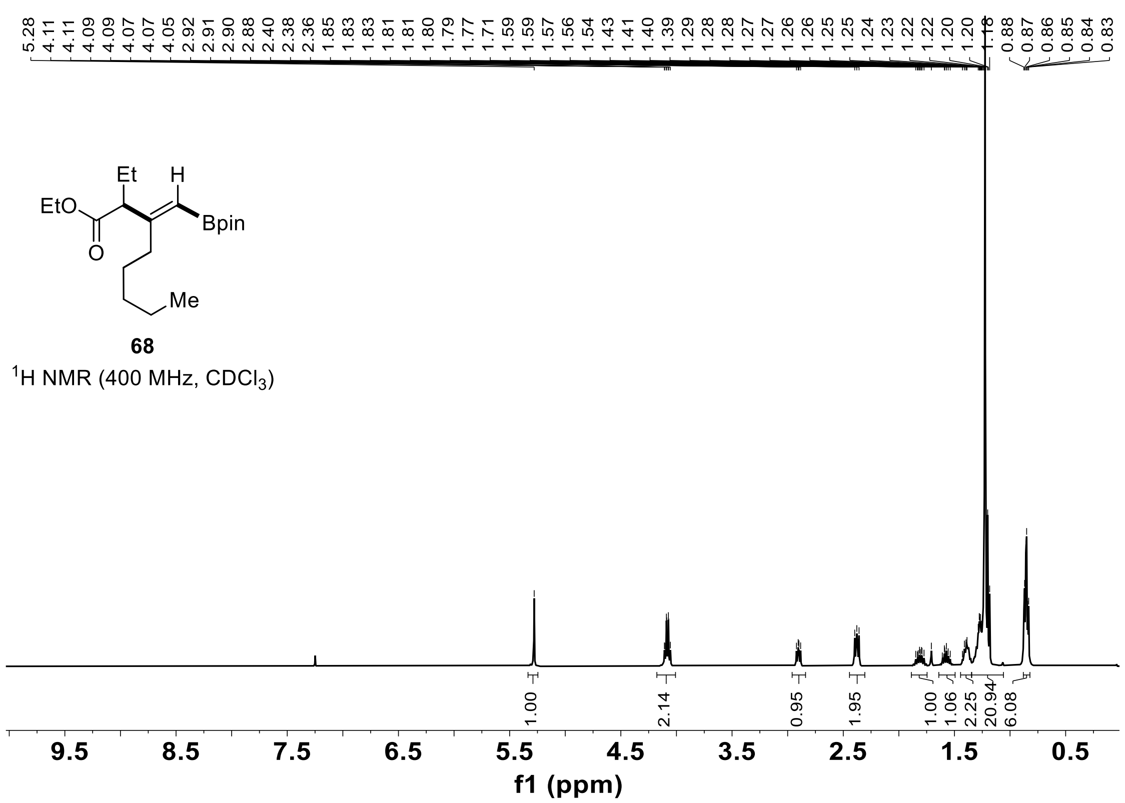

5.28 4.11 4.11 4.09 4.07 4.07 4.05 2.92 2.91 2.90 2.88 2.40 2.38 2.36 1.85 1.83 1.83 1.81 1.81 1.80 1.79 1.77 1.71 1.59 1.57 1.56 1.54 1.43 1.41 1.40 1.39 1.29 1.28 1.28 1.27 1.27 1.26 1.26 1.25 1.25 1.24 1.23 1.22 1.22 1.20 1.20 1.18 0.88 0.87 0.86 0.85 0.84 0.83
Et
H
EtO
Bpin
O
Me
68
1H NMR (400 MHz, CDCl3)
1.00 2.14 0.95 1.95 1.00 1.06 2.25 20.94 6.08
9.5 8.5 7.5 6.5 5.5 4.5 3.5 2.5 1.5 0.5
f1 (ppm)

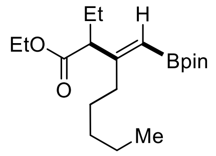

68

^{13}C NMR (101 MHz, $CDCl_3$)

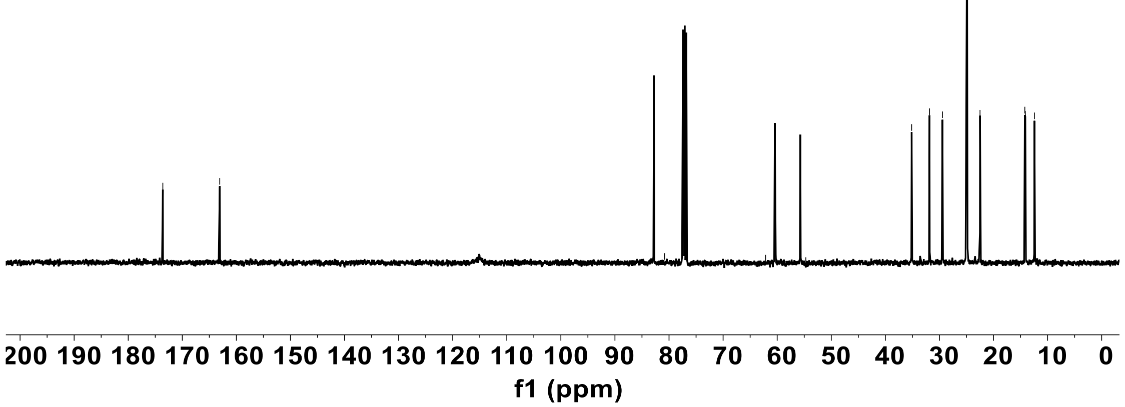

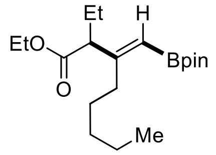

68

^{11}B NMR (193 MHz, $CDCl_3$)

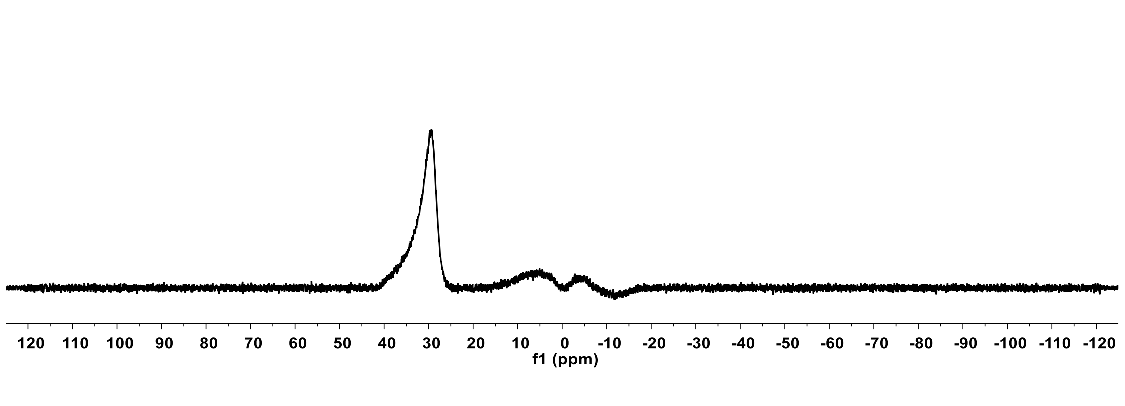

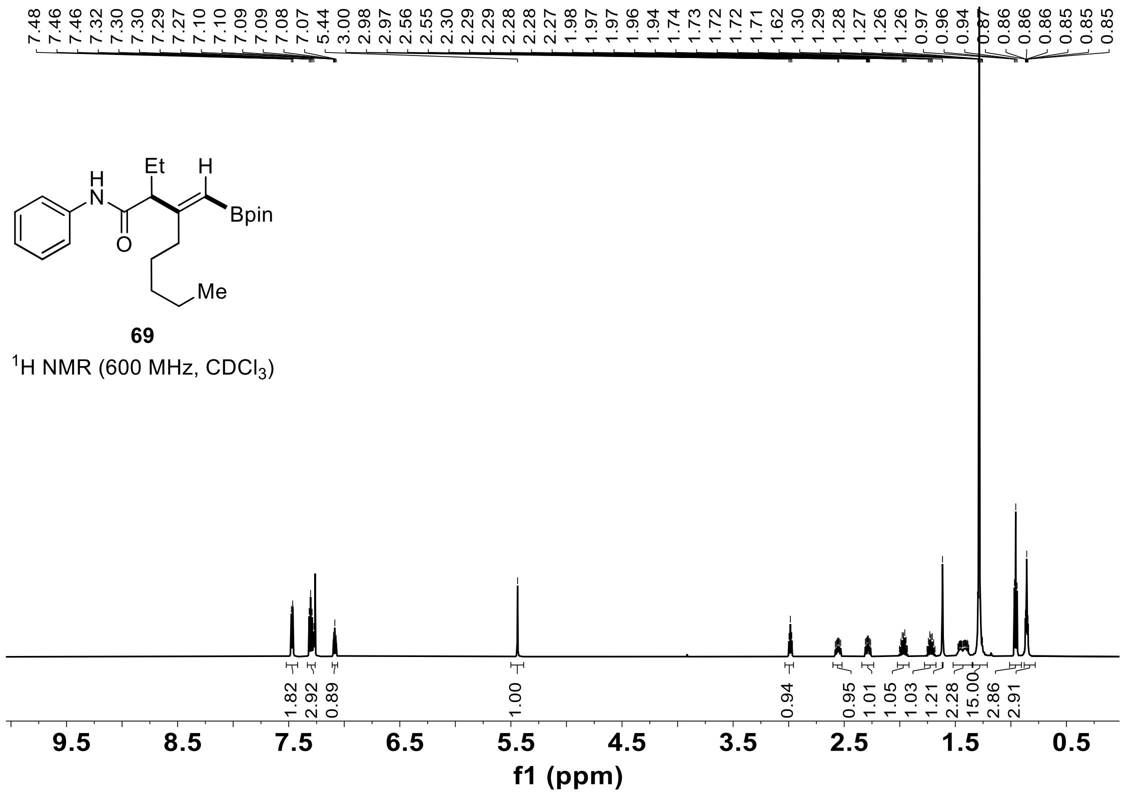
69
^{1}H NMR (600 MHz, CDCl$_3$)
f1 (ppm)

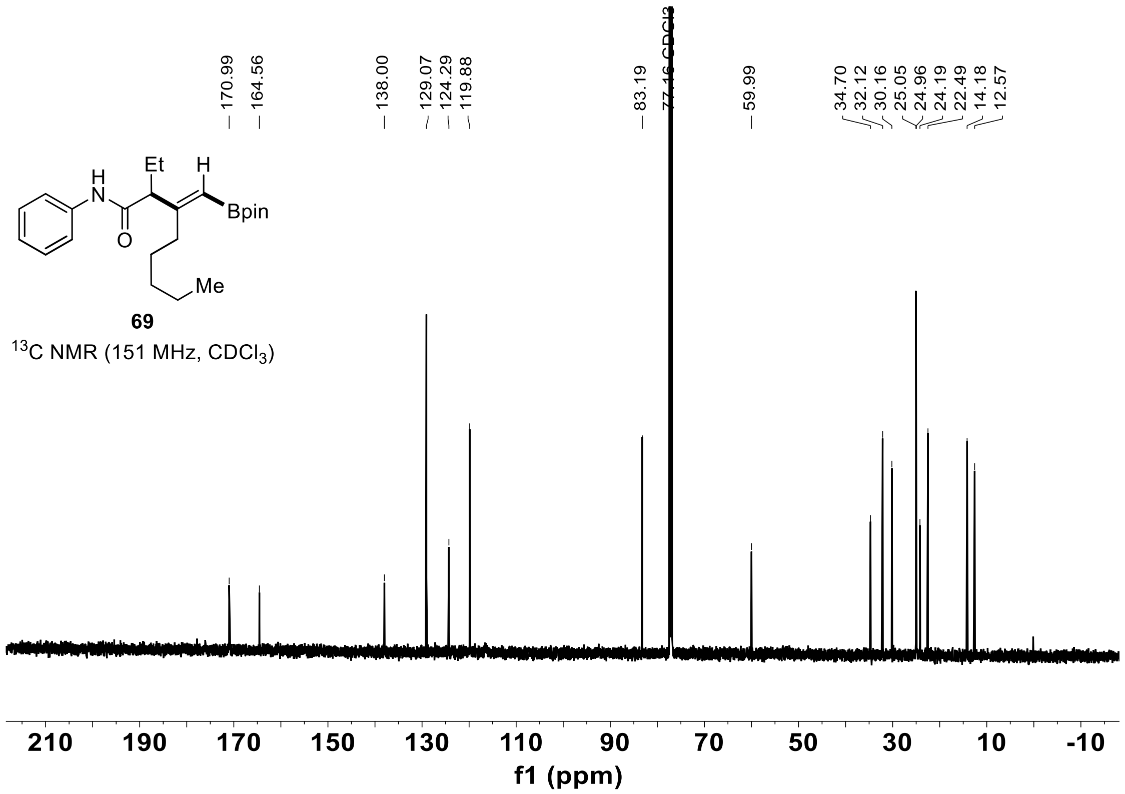
69
^{13}C NMR (151 MHz, CDCl$_3$)
f1 (ppm)

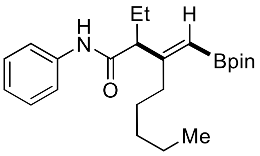

69

^{11}B NMR (193 MHz, $CDCl_3$)

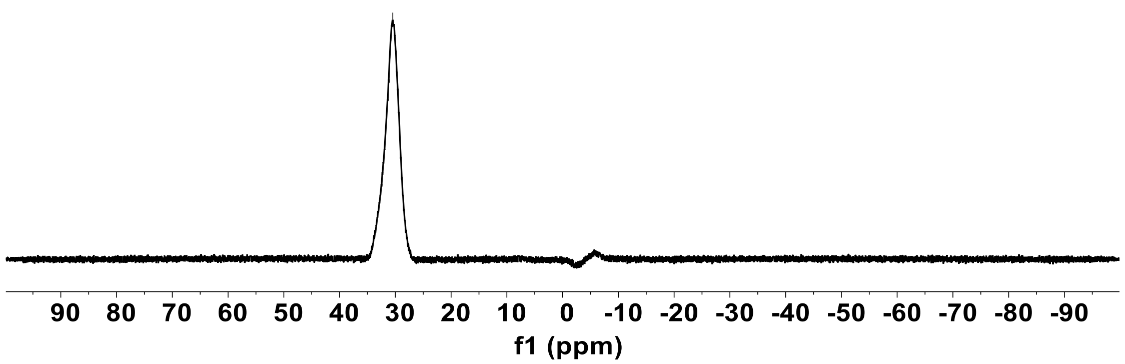

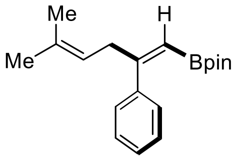

70

^{1}H NMR (600 MHz, $CDCl_3$)

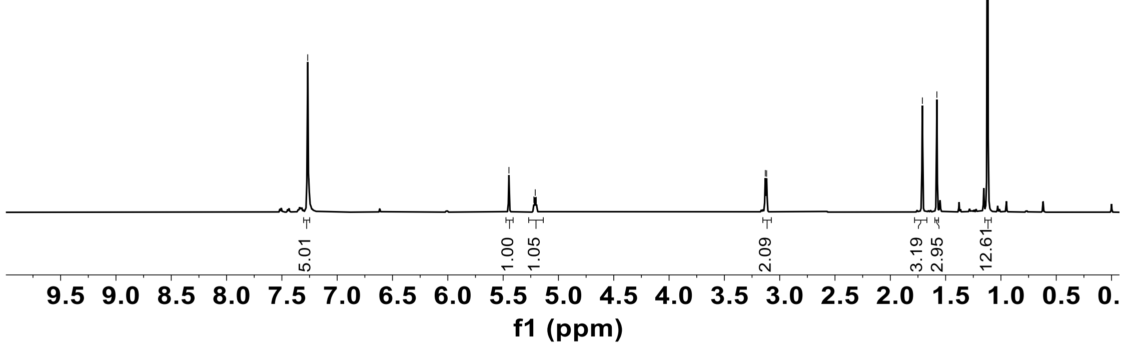

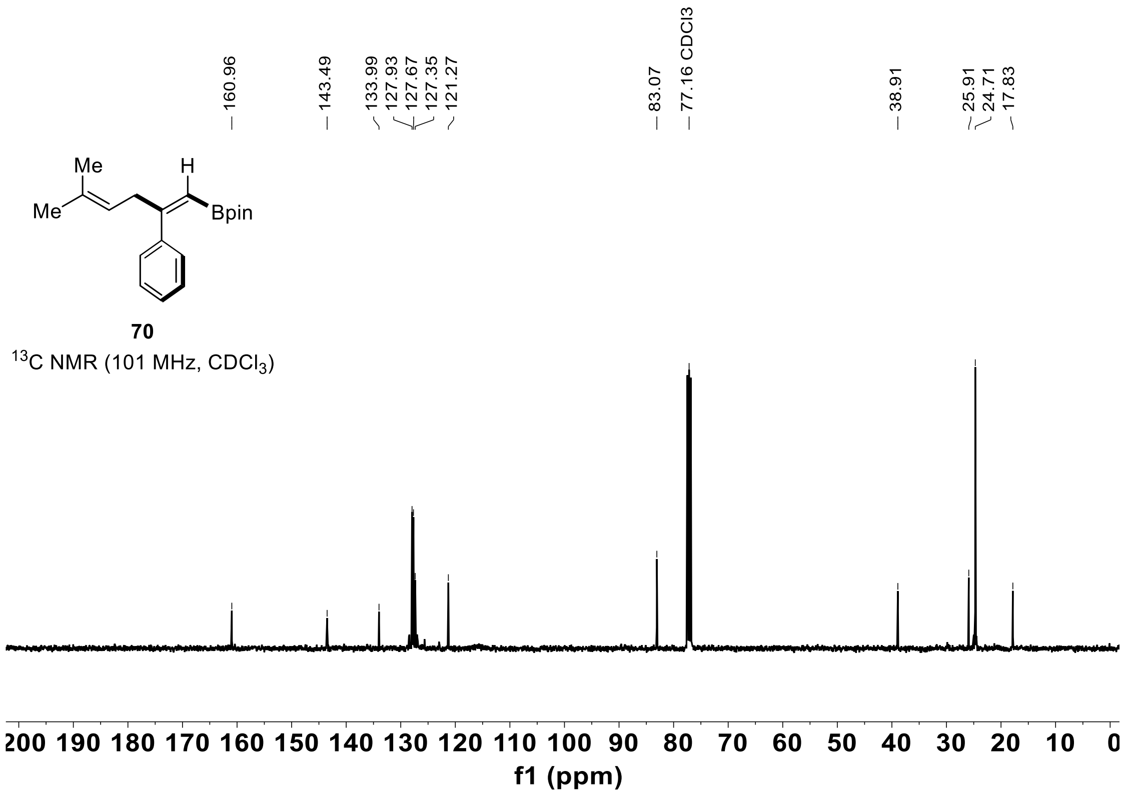
160.96
143.49
133.99
127.93
127.67
127.35
121.27
83.07
77.16 CDCl3
38.91
25.91
24.71
17.83
Me
Me
H
Bpin
70
^{13}C NMR (101 MHz, CDCl$_3$)
200 190 180 170 160 150 140 130 120 110 100 90 80 70 60 50 40 30 20 10 0
f1 (ppm)

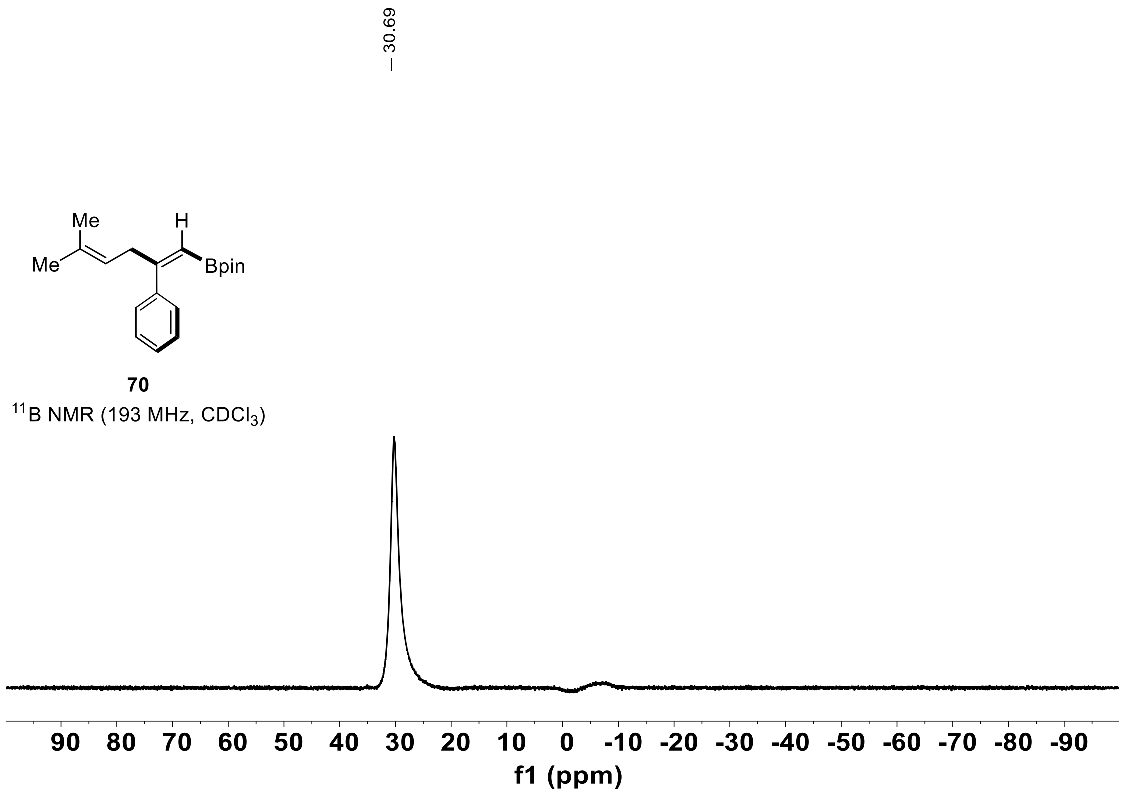
30.69
Me
Me
H
Bpin
70
^{11}B NMR (193 MHz, CDCl$_3$)
90 80 70 60 50 40 30 20 10 0 -10 -20 -30 -40 -50 -60 -70 -80 -90
f1 (ppm)

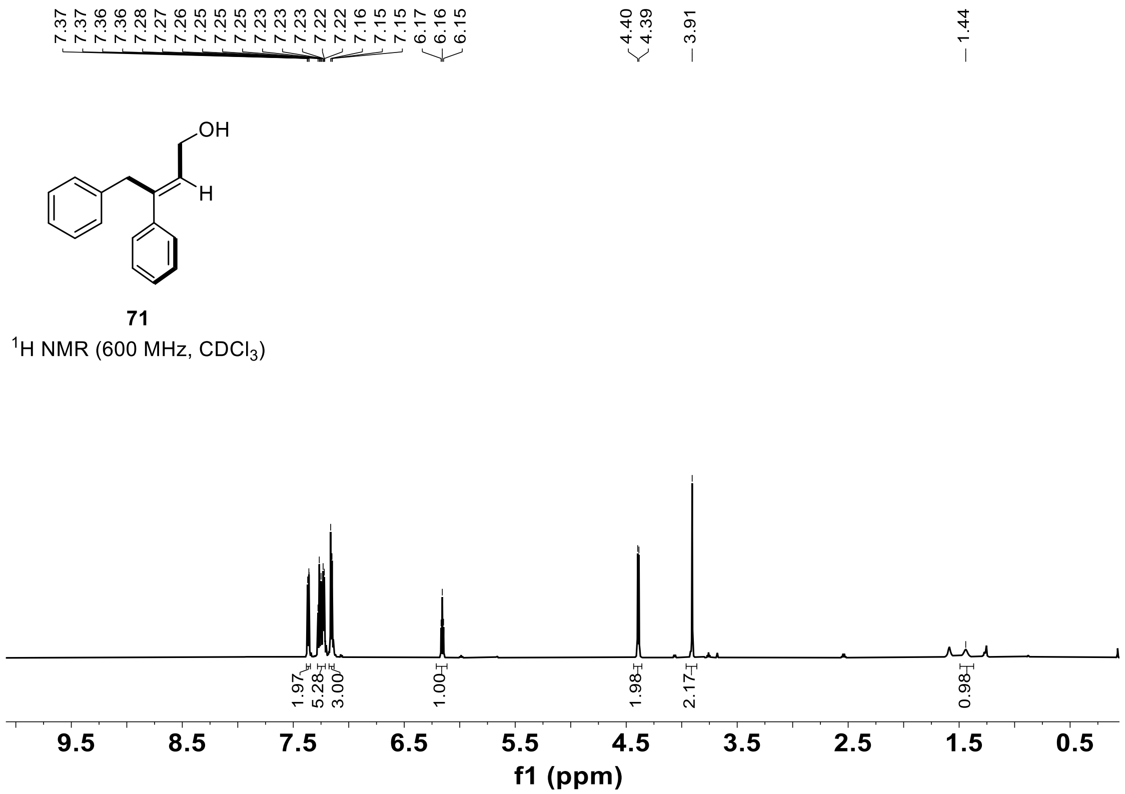
7.37
7.37
7.36
7.36
7.28
7.27
7.26
7.25
7.25
7.25
7.23
7.23
7.23
7.22
7.22
7.22
7.16
7.15
7.15
6.17
6.16
6.15
4.40
4.39
3.91
1.44
OH
H
71
^{1}H NMR (600 MHz, $CDCl_3$)
1.97
5.28
3.00
1.00
1.98
2.17
0.98
9.5
8.5
7.5
6.5
5.5
4.5
3.5
2.5
1.5
0.5
f1 (ppm)

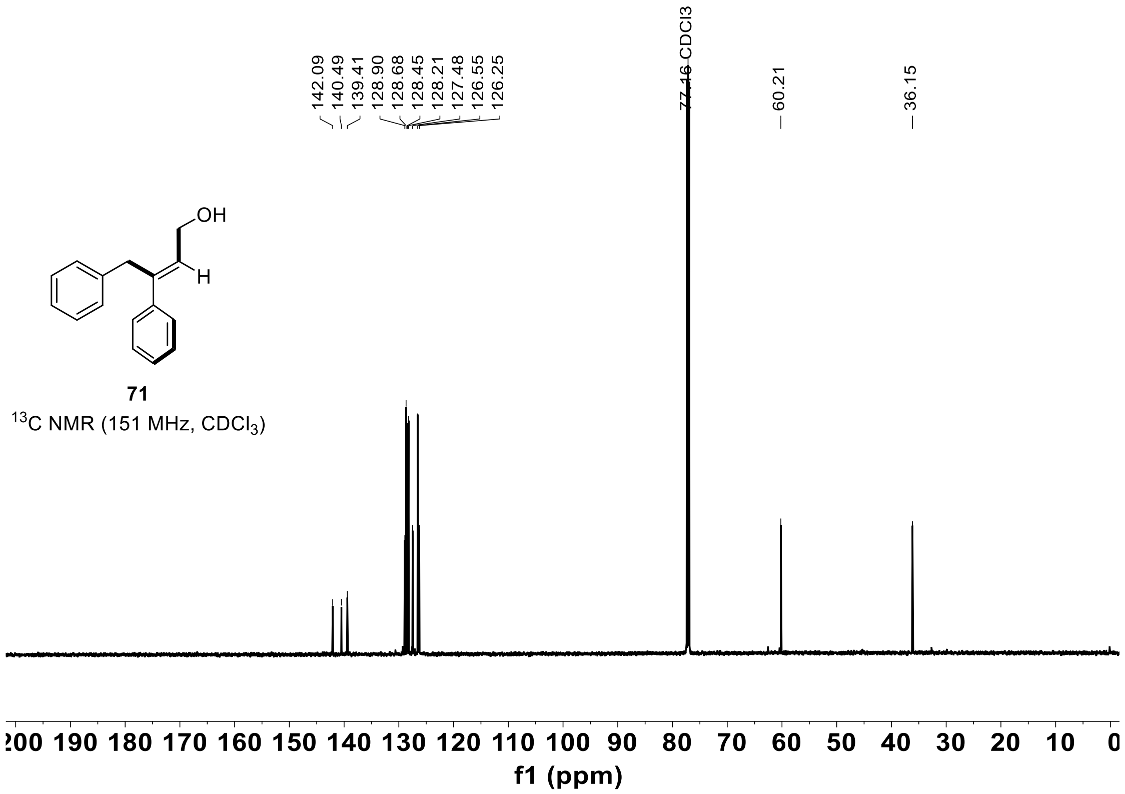
142.09
140.49
139.41
128.90
128.68
128.45
128.21
127.48
126.55
126.25
77.16 CDCl3
60.21
36.15
OH
H
71
^{13}C NMR (151 MHz, $CDCl_3$)
200 190 180 170 160 150 140 130 120 110 100 90 80 70 60 50 40 30 20 10 0
f1 (ppm)

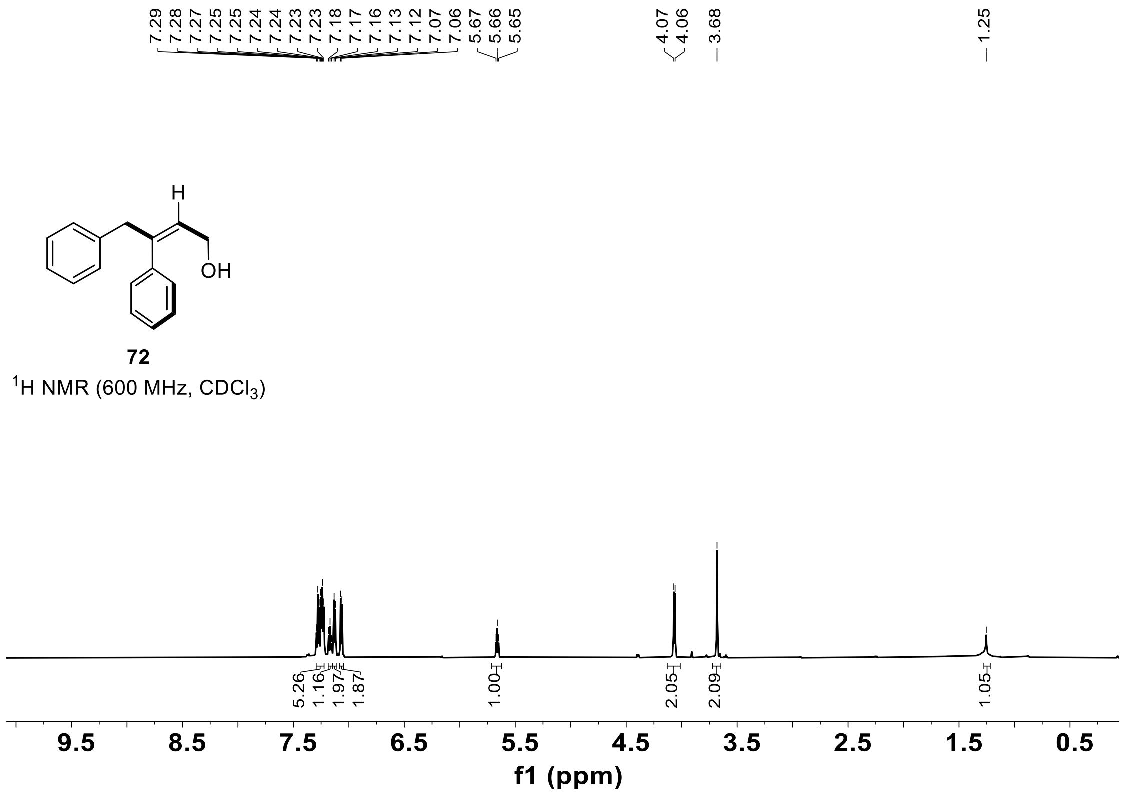

72

^{1}H NMR (600 MHz, $CDCl_3$)

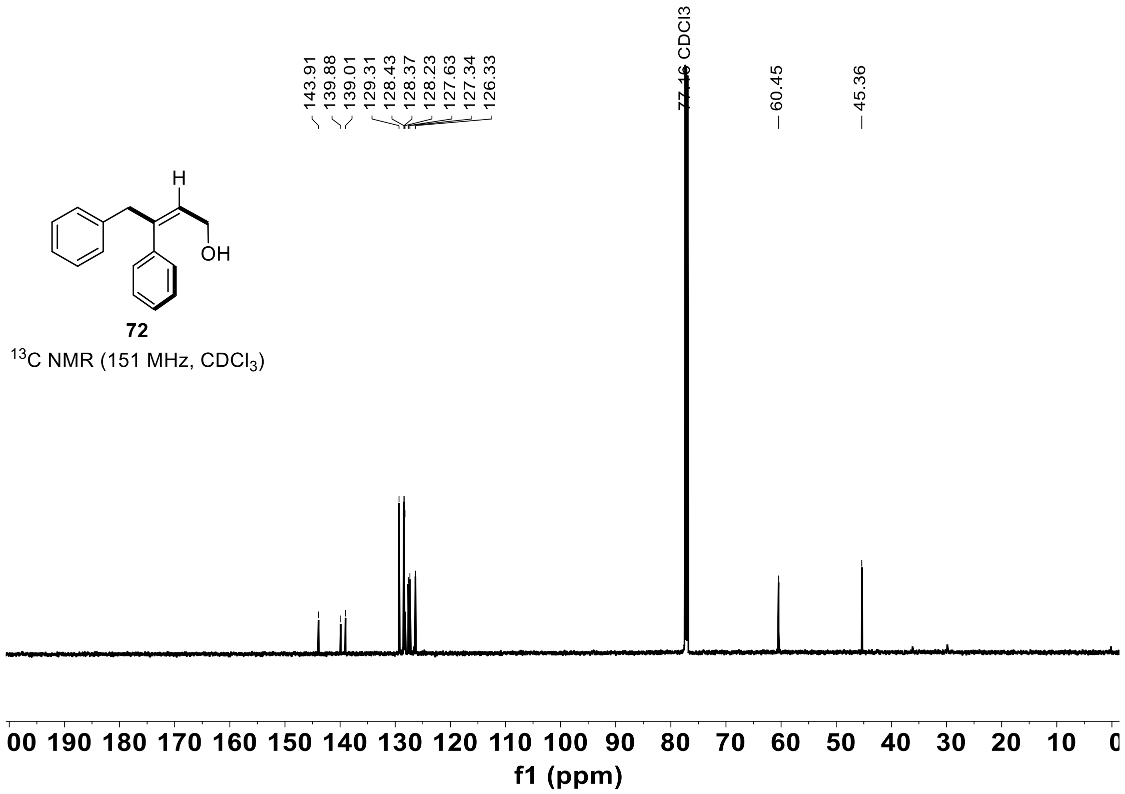

72

^{13}C NMR (151 MHz, $CDCl_3$)

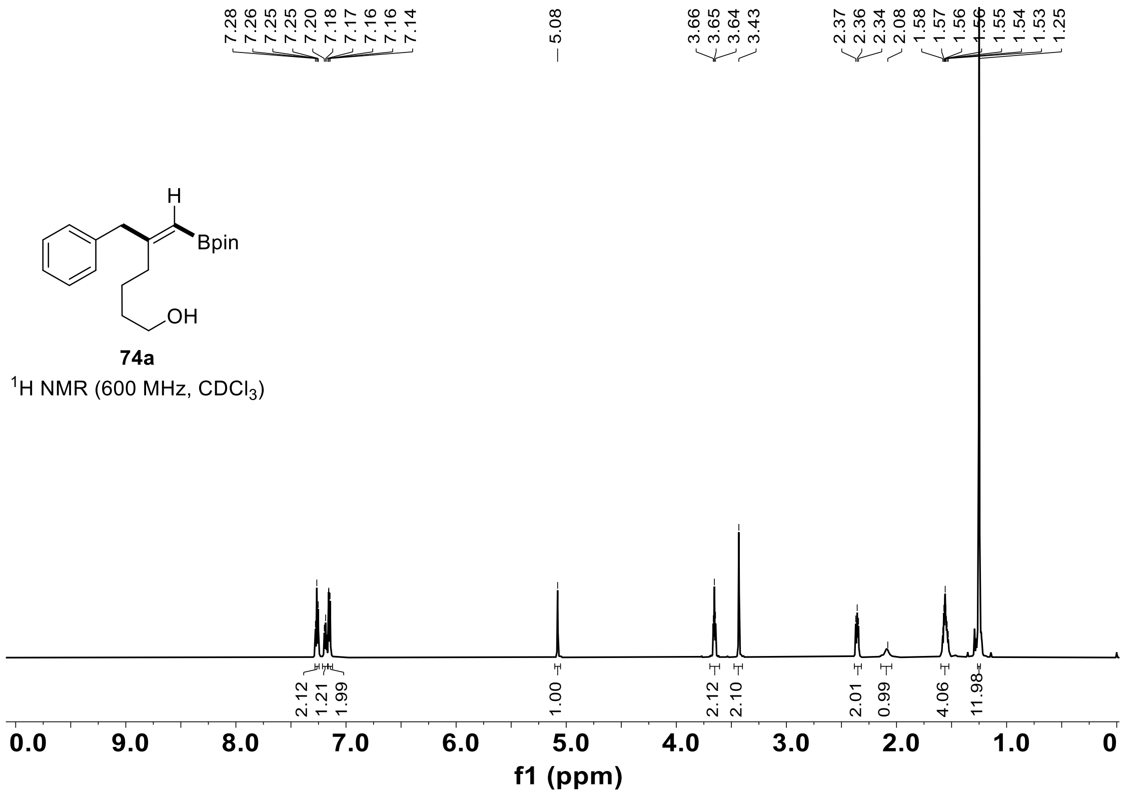

7.28 7.26 7.25 7.25 7.20 7.18 7.17 7.16 7.16 7.14
5.08
3.66 3.65 3.64 3.43
2.37 2.36 2.34 2.08 1.58 1.57 1.56 1.56 1.55 1.54 1.53 1.25
H
Bpin
OH
74a
^{1}H NMR (600 MHz, $CDCl_3$)
2.12 1.21 1.99 1.00 2.12 2.10 2.01 0.99 4.06 11.98
0.0 9.0 8.0 7.0 6.0 5.0 4.0 3.0 2.0 1.0 0
f1 (ppm)

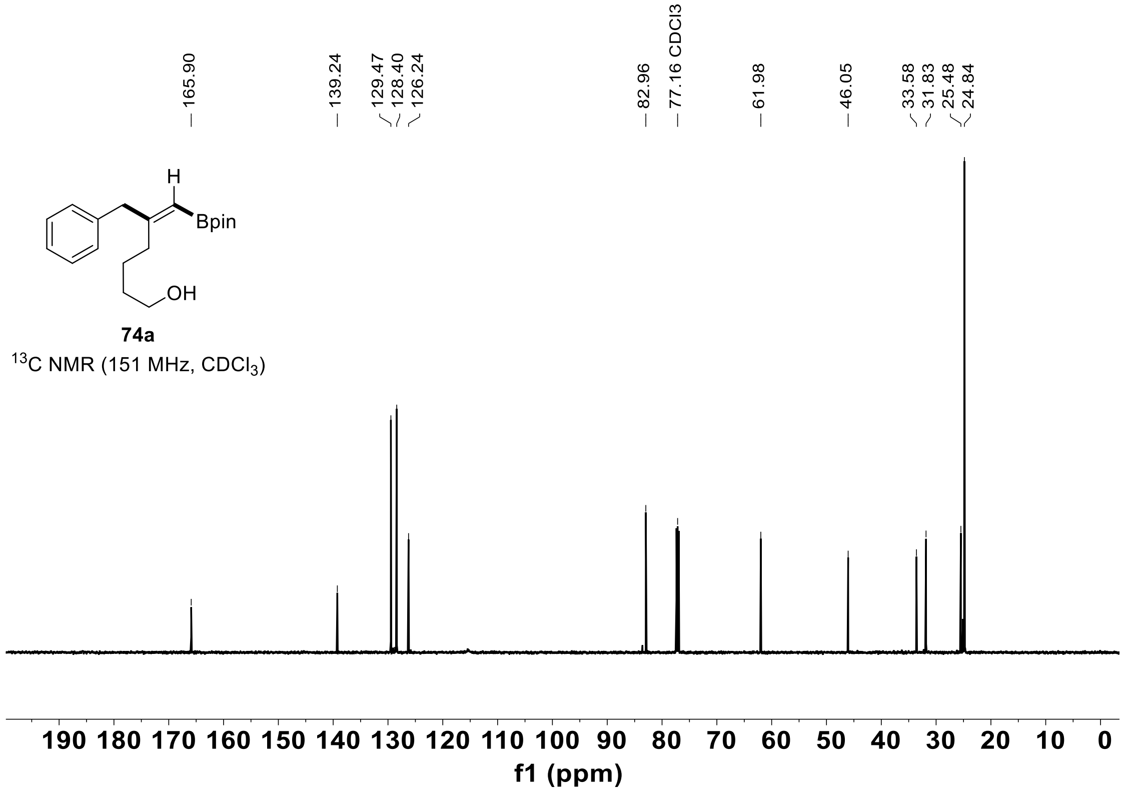

165.90
139.24
129.47 128.40 126.24
82.96
77.16 CDCl3
61.98
46.05
33.58 31.83 25.48 24.84
H
Bpin
OH
74a
^{13}C NMR (151 MHz, $CDCl_3$)
190 180 170 160 150 140 130 120 110 100 90 80 70 60 50 40 30 20 10 0
f1 (ppm)

29.92

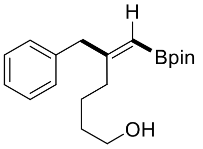

74a

^{11}B NMR (193 MHz, $CDCl_3$)

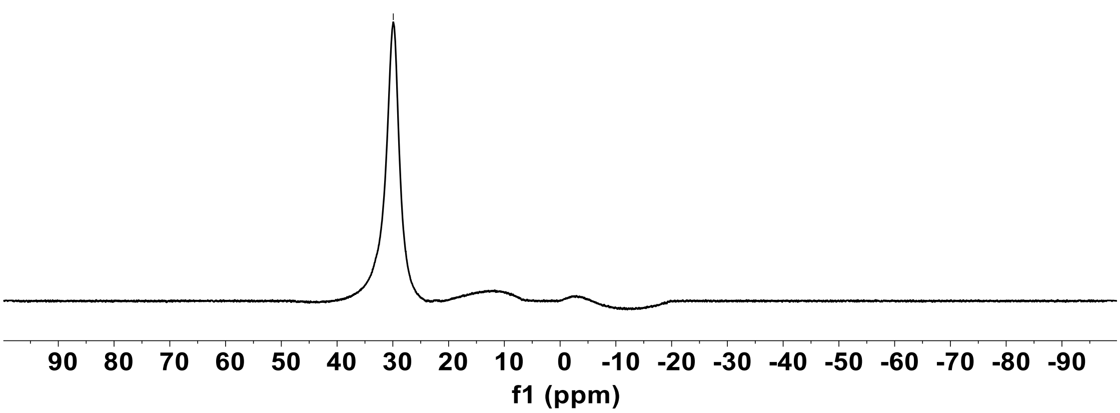

7.31 7.30 7.28 7.24 7.22 7.21 7.17 7.16 5.85 3.64 3.63 3.62 3.37 1.59 1.57 1.56 1.55 1.54 1.50 1.49 1.48 1.46 1.45 1.34

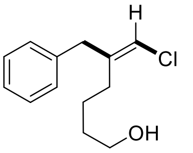

74b

^{1}H NMR (600 MHz, $CDCl_3$)

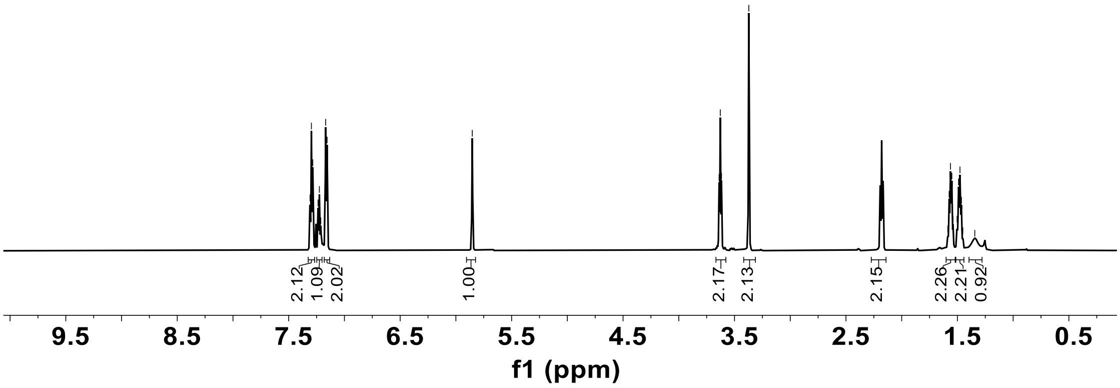

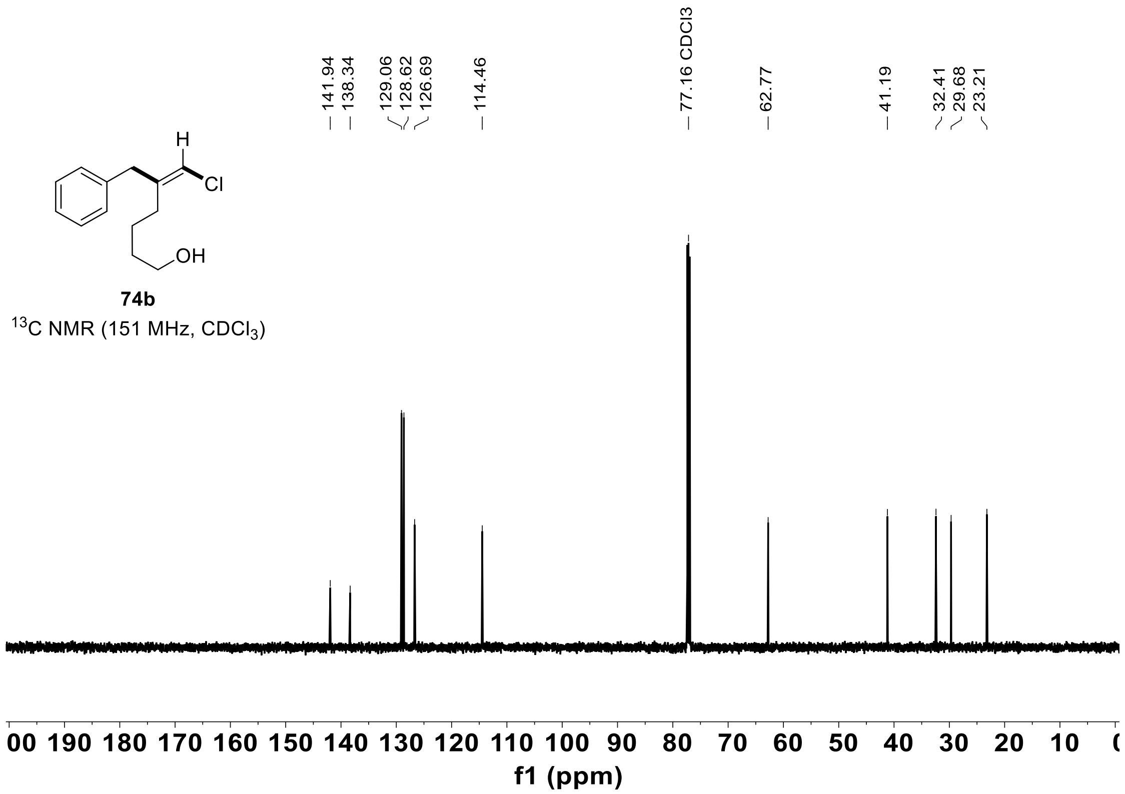

141.94
138.34
129.06
128.62
126.69
114.46
77.16 CDCl3
62.77
41.19
32.41
29.68
23.21
H
Cl
OH
74b
^{13}C NMR (151 MHz, $CDCl_3$)
f1 (ppm)

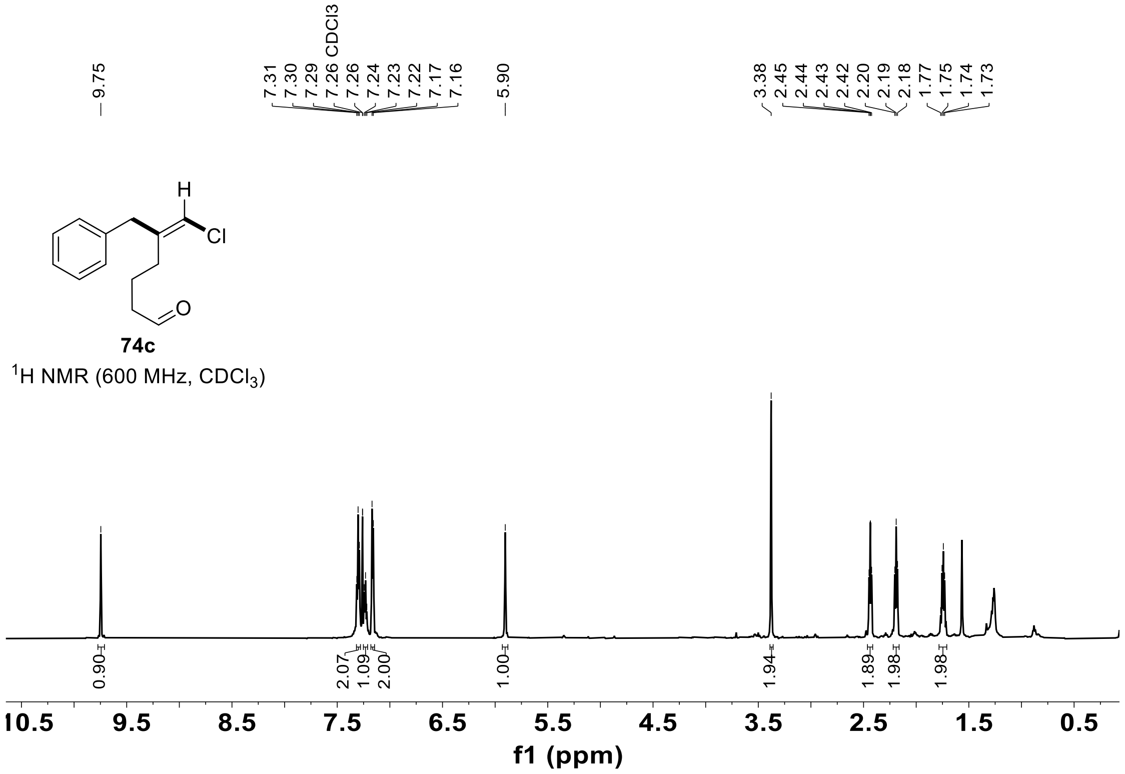

9.75
7.31
7.30
7.29
7.26 CDCl3
7.26
7.24
7.23
7.22
7.17
7.16
5.90
3.38
2.45
2.44
2.43
2.42
2.20
2.19
2.18
1.77
1.75
1.74
1.73
H
Cl
O
74c
^{1}H NMR (600 MHz, $CDCl_3$)
0.90
2.07
1.09
2.00
1.00
1.94
1.89
1.98
1.98
f1 (ppm)

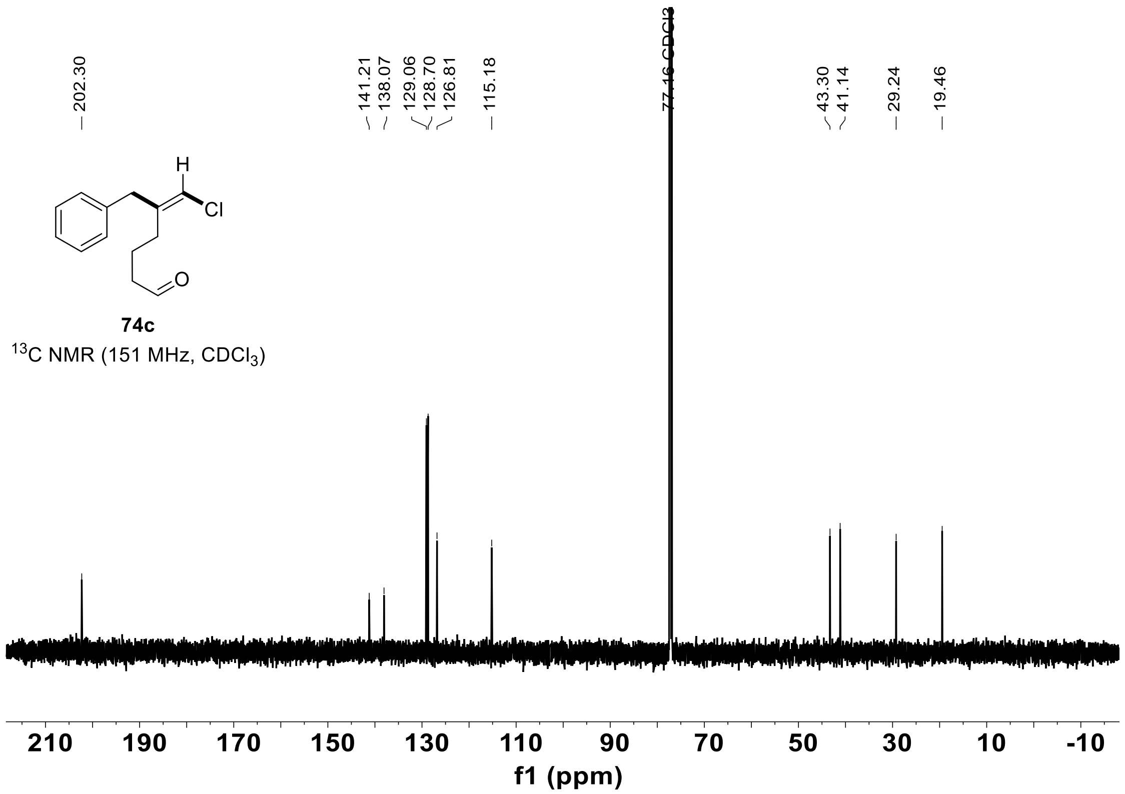
202.30
141.21
138.07
129.06
128.70
126.81
115.18
77.16 CDCl3
43.30
41.14
29.24
19.46
H
Cl
O
74c
^{13}C NMR (151 MHz, $CDCl_3$)
210
190
170
150
130
110
90
70
50
30
10
-10
f1 (ppm)

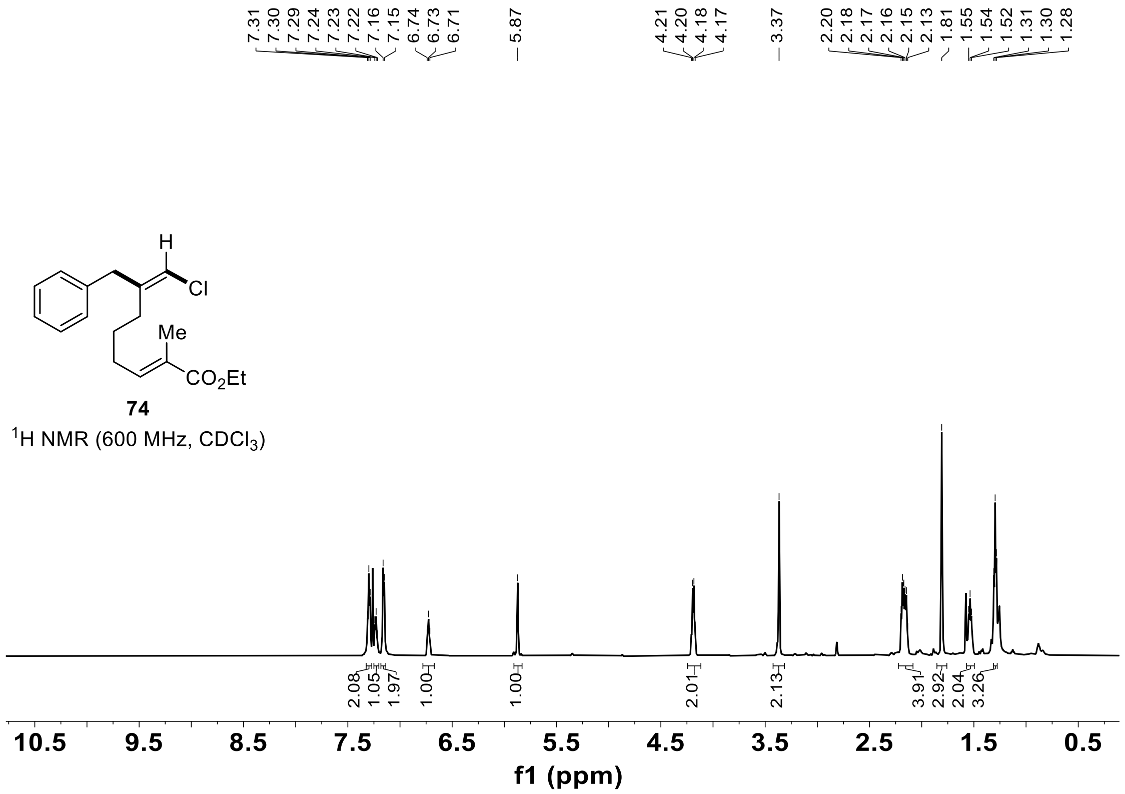
7.31
7.30
7.29
7.24
7.23
7.22
7.16
7.15
6.74
6.73
6.71
5.87
4.21
4.20
4.18
4.17
3.37
2.20
2.18
2.17
2.16
2.15
2.13
1.81
1.55
1.54
1.52
1.31
1.30
1.28
H
Cl
Me
CO_2Et
74
^1H NMR (600 MHz, $CDCl_3$)
2.08
1.05
1.97
1.00
1.00
2.01
2.13
3.91
2.92
2.04
3.26
10.5
9.5
8.5
7.5
6.5
5.5
4.5
3.5
2.5
1.5
0.5
f1 (ppm)

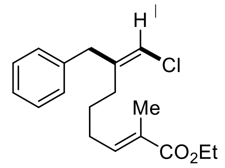

74

^{13}C NMR (151 MHz, $CDCl_3$)

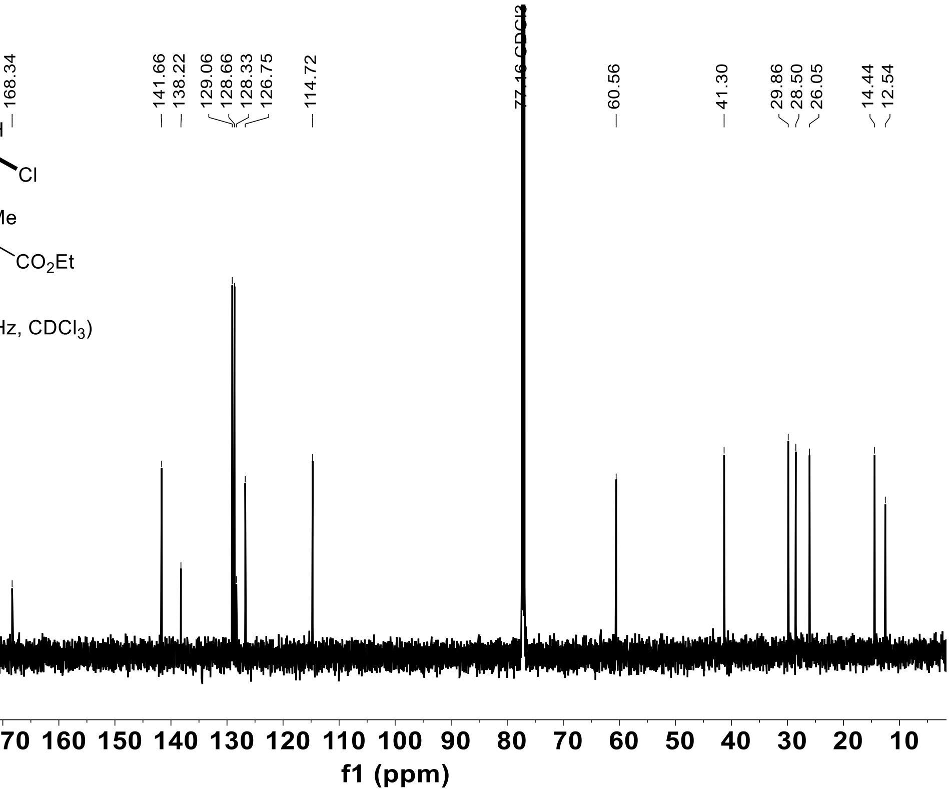

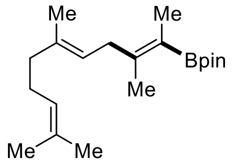

77

^{1}H NMR (600 MHz, $CDCl_3$)

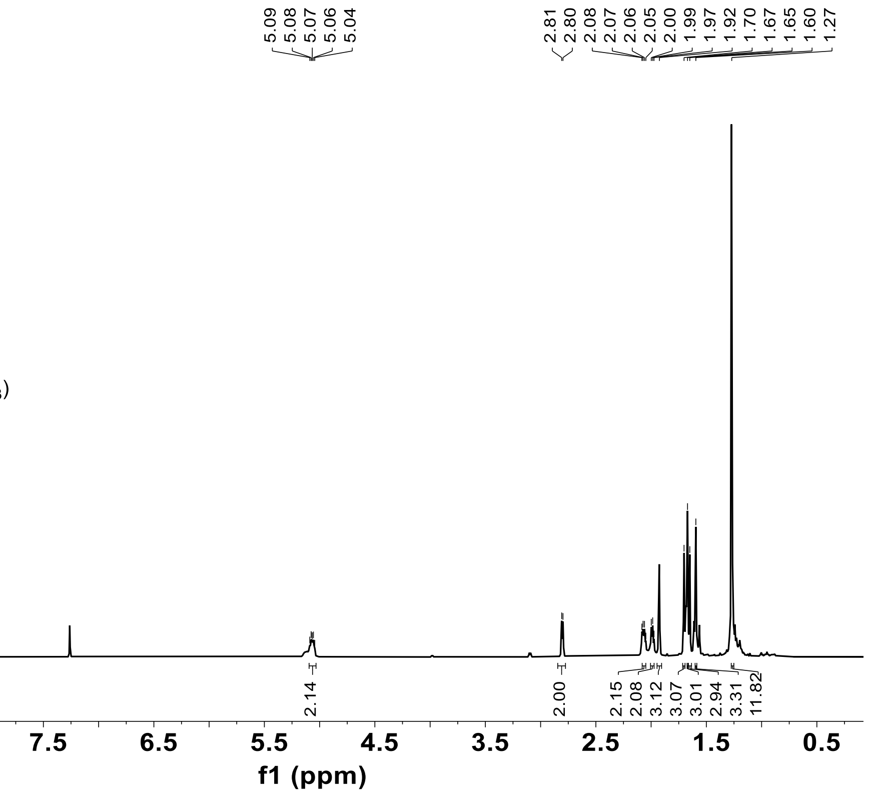

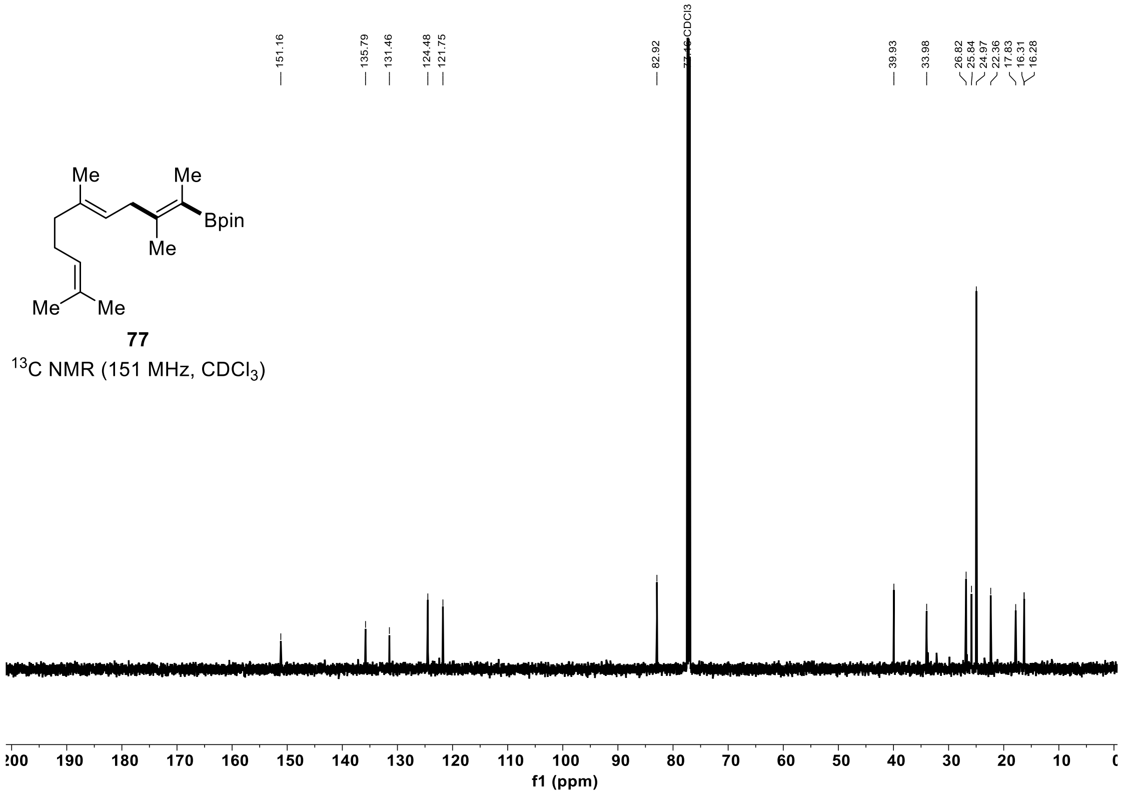
151.16
135.79
131.46
124.48
121.75
82.92
77.16 CDCl3
39.93
33.98
26.82
25.84
24.97
22.36
17.83
16.31
16.28
Me
Me
Bpin
Me
Me
Me
77
^{13}C NMR (151 MHz, $CDCl_3$)
200 190 180 170 160 150 140 130 120 110 100 90 80 70 60 50 40 30 20 10 0
f1 (ppm)

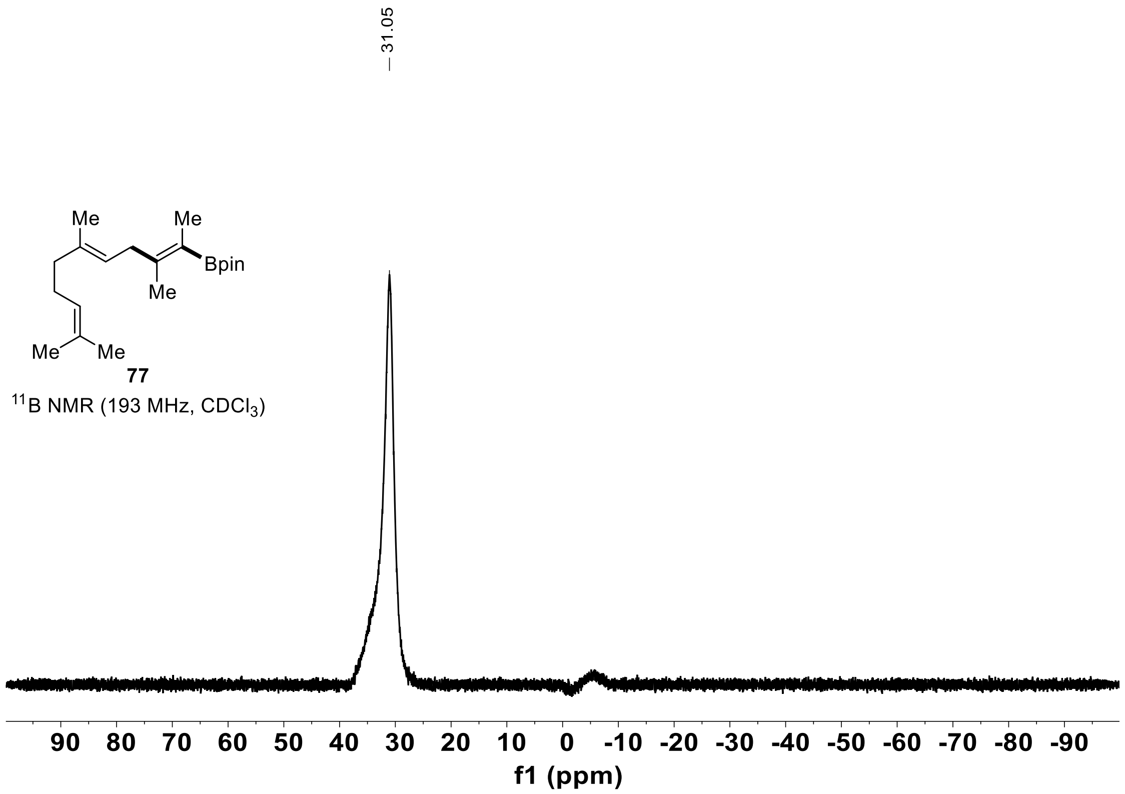
31.05
Me
Me
Bpin
Me
Me
Me
77
^{11}B NMR (193 MHz, $CDCl_3$)
90 80 70 60 50 40 30 20 10 0 -10 -20 -30 -40 -50 -60 -70 -80 -90
f1 (ppm)

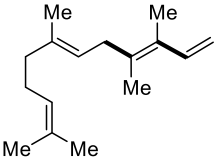

78

^{1}H NMR (600 MHz, $CDCl_3$)

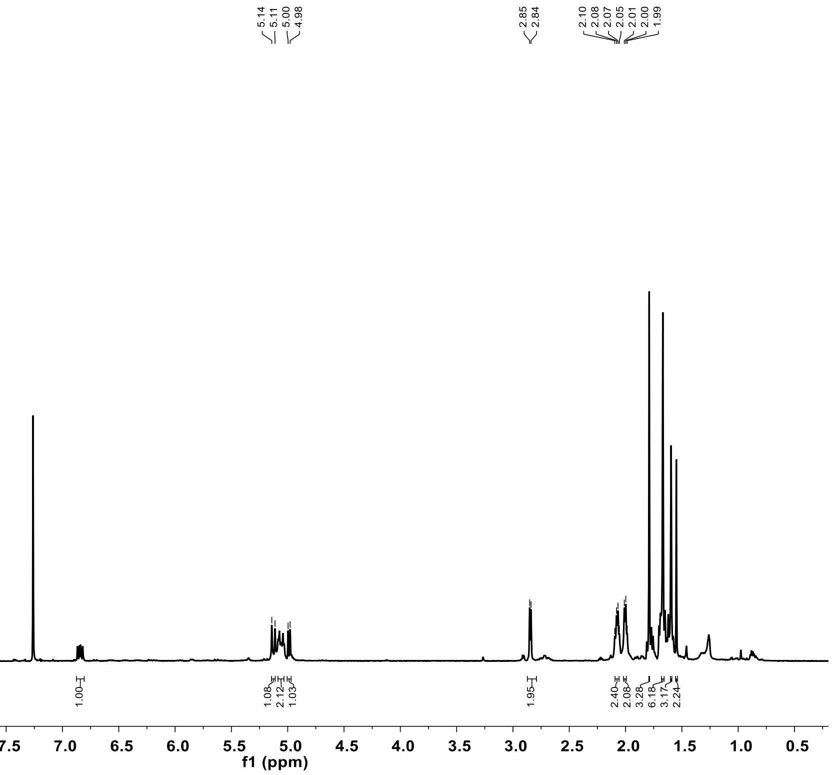

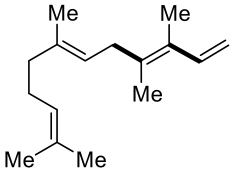

78

^{13}C NMR (151 MHz, $CDCl_3$)

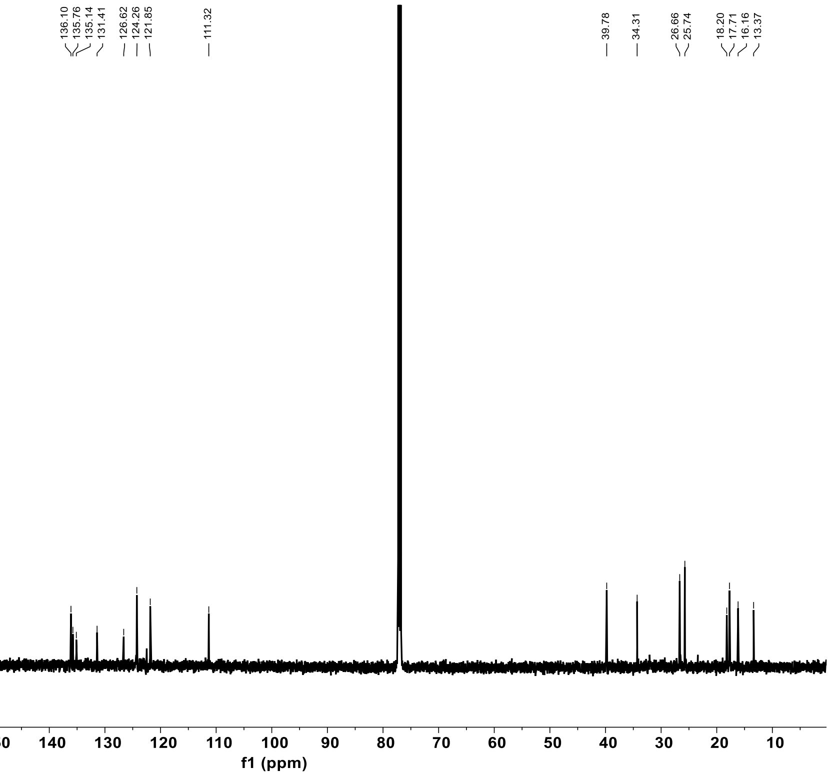

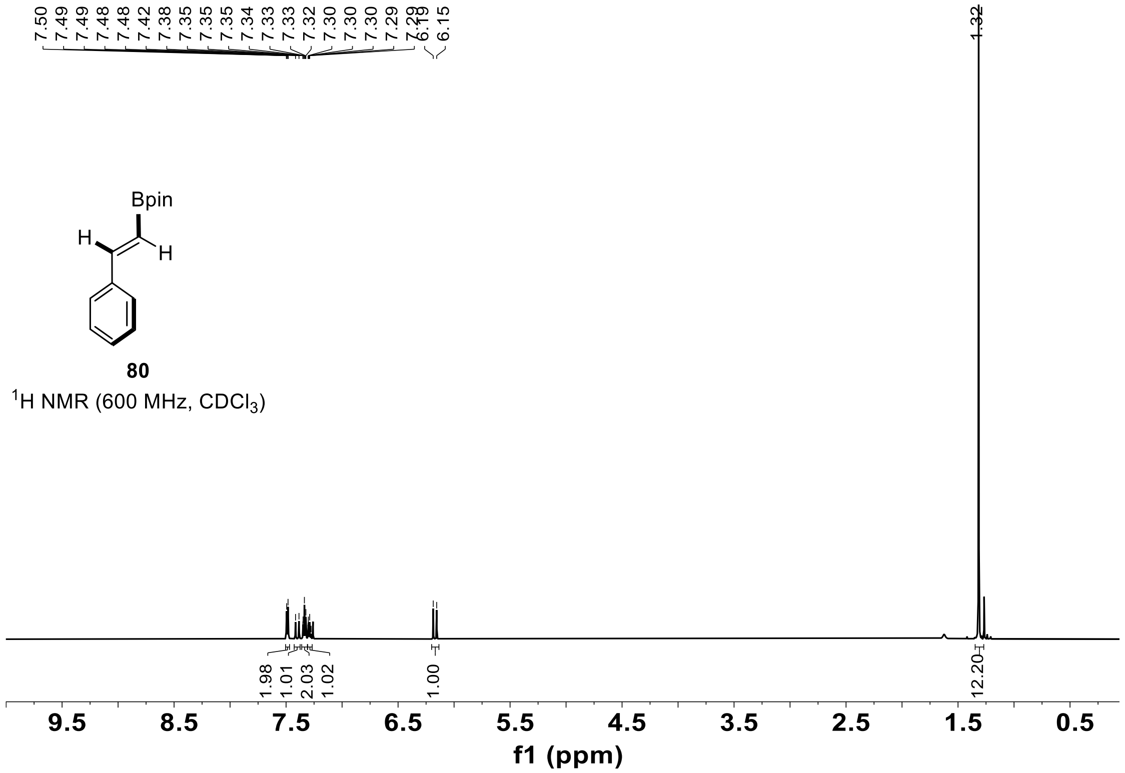
7.50 7.49 7.49 7.48 7.48 7.42 7.38 7.35 7.35 7.34 7.33 7.33 7.32 7.30 7.30 7.30 7.29 7.29 6.19 6.15
1.32
Bpin
H
H
80
^{1}H NMR (600 MHz, CDCl$_3$)
1.98 1.01 2.03 1.02
1.00
12.20
9.5 8.5 7.5 6.5 5.5 4.5 3.5 2.5 1.5 0.5
f1 (ppm)

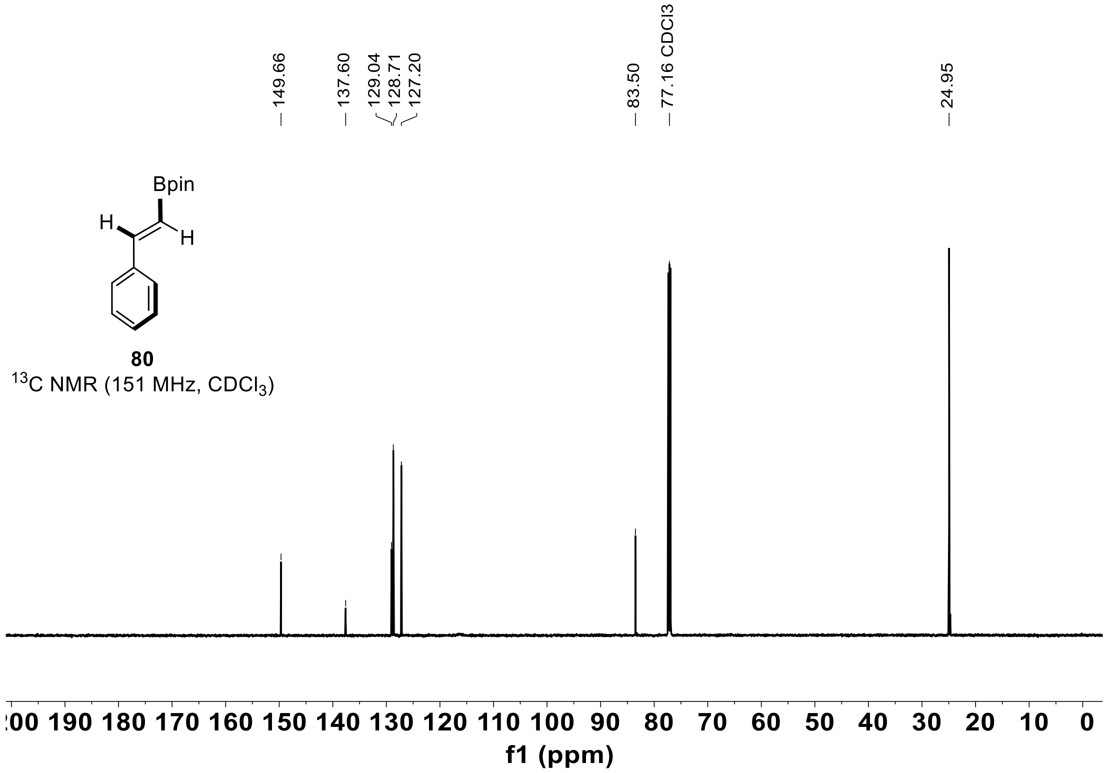
149.66
137.60
129.04 128.71 127.20
83.50
77.16 CDCl3
24.95
Bpin
H
H
80
^{13}C NMR (151 MHz, CDCl$_3$)
200 190 180 170 160 150 140 130 120 110 100 90 80 70 60 50 40 30 20 10 0
f1 (ppm)

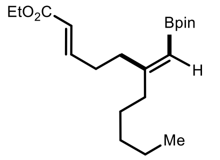

86

^{1}H NMR (600 MHz, $CDCl_3$)

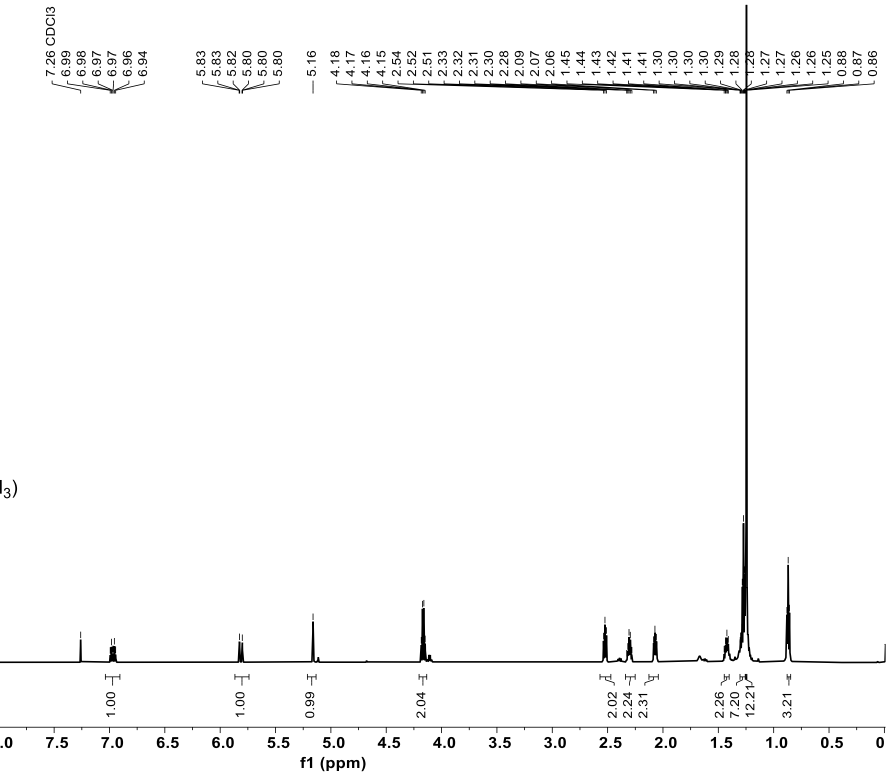

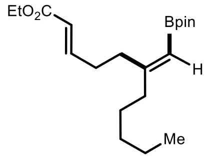

86

^{13}C NMR (151 MHz, $CDCl_3$)

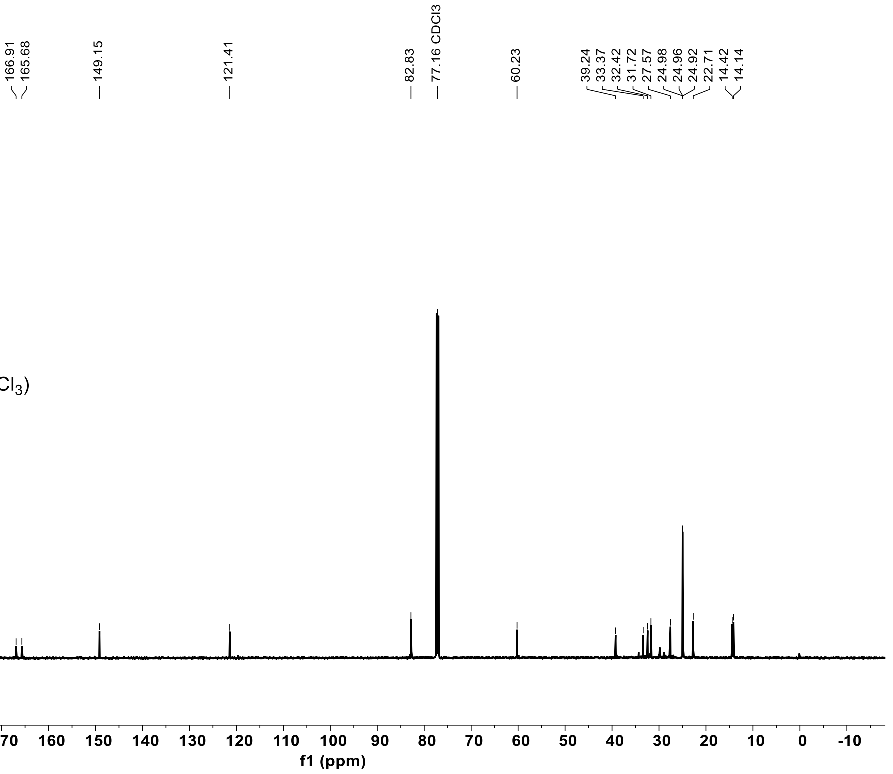

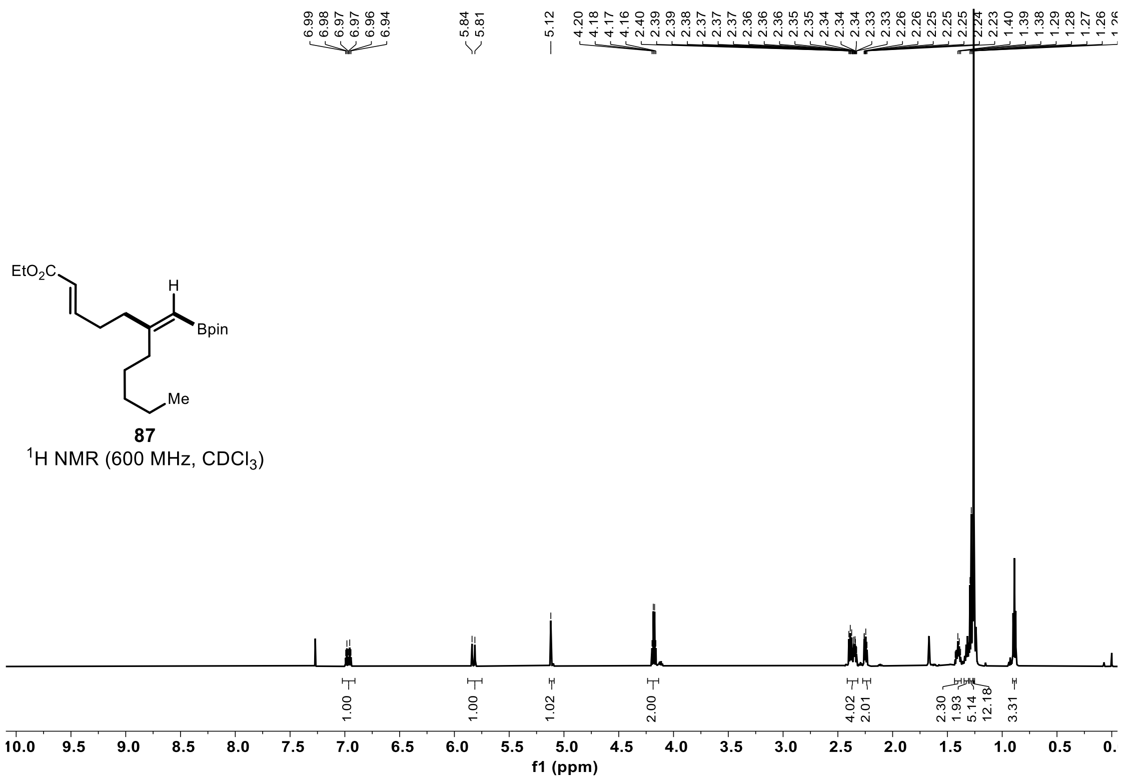

87

^{1}H NMR (600 MHz, $CDCl_3$)

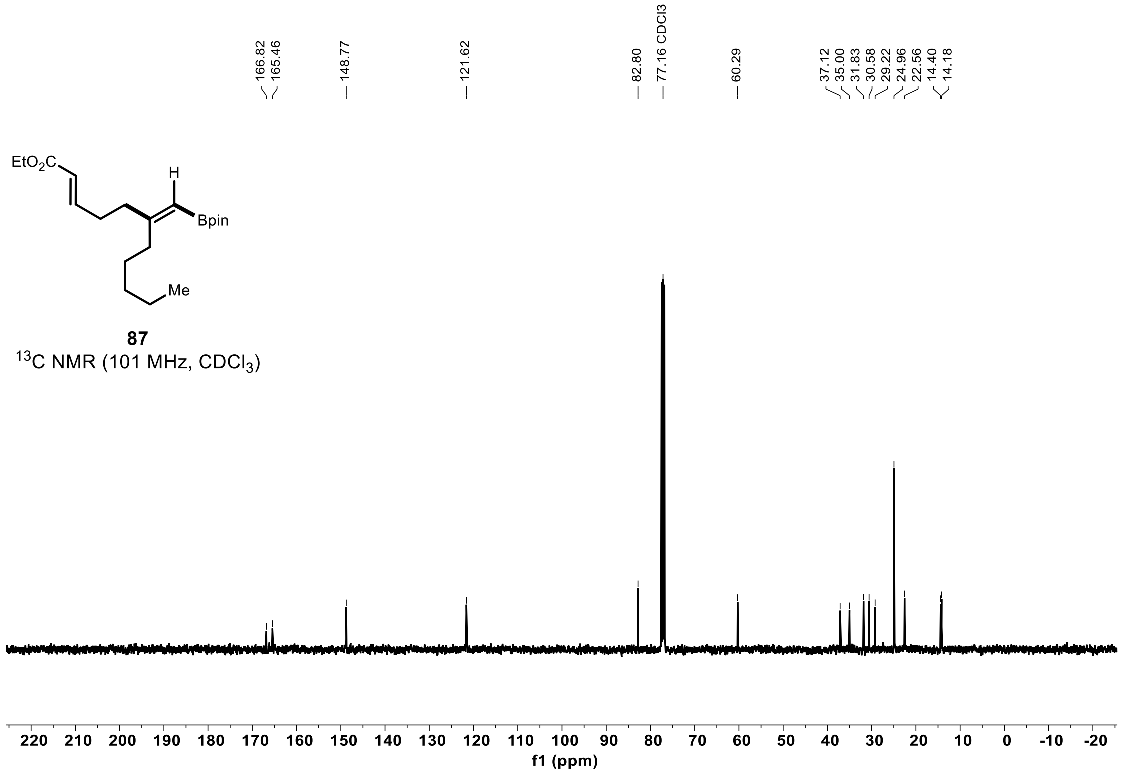

87

^{13}C NMR (101 MHz, $CDCl_3$)

10. References

1. Alfaro, R., Parra, A., Alemán, J., García Ruano, J. L. & Tortosa, M. Copper(I)-Catalyzed Formal Carboboration of Alkynes: Synthesis of Tri- and Tetrasubstituted Vinylboronates. *J. Am. Chem. Soc.* **134**, 15165-15168 (2012).
2. Zhang, J., Dai, W., Liu, Q. & Cao, S. Cu-Catalyzed Stereoselective Borylation of gem-Difluoroalkenes with B_2pin_2. *Org. Lett.* **19**, 3283-3286 (2017).
3. Kong, W. *et al.* Base-Modulated 1,3-Regio- and Stereoselective Carboboration of Cyclohexenes. *Angew. Chem., Int. Ed.* **62**, e202308041 (2023).
4. Liu, T.-L., Ng, T. W. & Zhao, Y. Rhodium-Catalyzed Enantioselective Isomerization of Secondary Allylic Alcohols. *J. Am. Chem. Soc.* **139**, 3643-3646 (2017).
5. Sasaki, K. & Hayashi, T. Rhodium-Catalyzed Asymmetric Conjugate Addition of Arylboroxines to Borylalkenes: Asymmetric Synthesis of β-Arylalkylboranes. *Angew. Chem., Int. Ed.* **49**, 8145-8147 (2010).
6. Zhang, Y.-D. *et al.* Highly Regioselective Cobalt-Catalyzed Hydroboration of Internal Alkynes. *Angew. Chem., Int. Ed.* **61**, e202208473 (2022).
7. Ota, Y. *et al.* Cancer-Cell-Selective Targeting by Arylcyclopropylamine–Vorinostat Conjugates. *ACS Med. Chem. Lett.* **13**, 1568-1573 (2022).
8. Nitelet, A., Gérard, P., Bouche, J. & Evano, G. Total Synthesis of Conulothiazole A. *Org. Lett.* **21**, 4318-4321 (2019).
9. Sun, C., Li, Y. & Yin, G. Practical Synthesis of Chiral Allylboronates by Asymmetric 1,1-Difunctionalization of Terminal Alkenes. *Angew. Chem., Int. Ed.* **61**, e202209076 (2022).
10. Sahoo, H., Zhang, L., Cheng, J., Nishiura, M. & Hou, Z. Auto-Tandem Copper-Catalyzed Carboxylation of Undirected Alkenyl C–H Bonds with CO2 by Harnessing β-Hydride Elimination. *J. Am. Chem. Soc.* **144**, 23585-23594 (2022).
11. Polášek, J. *et al.* Stereoselective Bromoboration of Acetylene with Boron Tribromide: Preparation and Cross-Coupling Reactions of (Z)-Bromovinylboronates. *J. Org. Chem.* **85**, 6992-7000 (2020).
12. Huang, C., Wu, D., Li, Y. & Yin, G. Asymmetric anti-Selective Borylalkylation of Terminal Alkynes by Nickel Catalysis. *J. Am. Chem. Soc.* **145**, 18722-18730 (2023).
13. M. J. Frisch, G. W. T., H. B. Schlegel, G. E. Scuseria, M. A. Robb, J. R. Cheeseman, *et al. Fox Gaussian 16, Revision B.01; Gaussian, Inc.: Wallingford, CT, .* (2019).
14. Lee, C., Yang, W. & Parr, R. G. Development of the Colle-Salvetti correlation-energy formula into a functional of the electron density. *Phys.Rev.B.* **37**, 785-789 (1988).
15. Becke, A. D. Density‐functional thermochemistry. III. The role of exact exchange. *J.Chem.Phys.* **98**, 5648-5652 (1993).
16. Grimme, S., Antony, J., Ehrlich, S. & Krieg, H. A consistent and accurate ab initio parametrization of density functional dispersion correction (DFT-D) for the 94 elements H-Pu. *J.Chem.Phys.* **132**, 154104 (2010).
17. Weigend, F. & Ahlrichs, R. Balanced basis sets of split valence, triple zeta valence and quadruple zeta valence quality for H to Rn: Design and assessment of accuracy. *Phys. Chem. Chem. Phys.* **7**, 3297-3305 (2005).

18. Zhao, Y. & Truhlar, D. G. The M06 suite of density functionals for main group thermochemistry, thermochemical kinetics, noncovalent interactions, excited states, and transition elements: two new functionals and systematic testing of four M06-class functionals and 12 other functionals. *Theor. Chem. Acc.* **120**, 215-241 (2008).
19. Schäfer, A., Huber, C. & Ahlrichs, R. Fully optimized contracted Gaussian basis sets of triple zeta valence quality for atoms Li to Kr. *J.Chem.Phys.* **100**, 5829-5835 (1994).
20. Marenich, A. V., Cramer, C. J. & Truhlar, D. G. Universal Solvation Model Based on Solute Electron Density and on a Continuum Model of the Solvent Defined by the Bulk Dielectric Constant and Atomic Surface Tensions. *J. Phys. Chem. B.* **113**, 6378-6396 (2009).
21. CYLview20; Legault, C. Y., Université de. *Sherbrooke.* L10-CP17 (2020).
22. Lu, T. & Chen, F. Multiwfn: A multifunctional wavefunction analyzer. *J.Comput.Chem.* **33**, 580-592 (2012).
23. Huang, C. *et al.* Epoxide Electroreduction. *J. Am. Chem. Soc.* **144**, 1389-1395 (2022).
24. Bickelhaupt, F. M. & Houk, K. N. Analyzing Reaction Rates with the Distortion/Interaction-Activation Strain Model. *Angew. Chem., Int. Ed.* **56**, 10070-10086 (2017).
25. Bannwarth, C. C., E.; Ehlert, S.; Hansen, A.; Pracht, P.; Seibert, J.; Spicher, S.; Grimme, S., . Extended tight-binding quantum chemistry methods. . *WIREs Comput. Mol. Sci.* **11**, e01493 (2020).
26. Lu, T. *Molclus program, Version 1.12,* http://www.keinsci.com/research/ molclus.html (accessed Aug 23, 2023).
